# Pyrimidine Nucleosides Syntheses by Late-Stage Base Heterocyclization Reactions

**DOI:** 10.1021/acs.orglett.2c03152

**Published:** 2022-11-04

**Authors:** Elfie S. Cavalli, Thomas Mies, Henry S. Rzepa, Andrew J. P. White, Philip J. Parsons, Anthony G.M. Barrett

**Affiliations:** Department of Chemistry, Molecular Sciences Research Hub, White City Campus, Imperial College London, 82 Wood Lane, London W12 0BZ, England

## Abstract

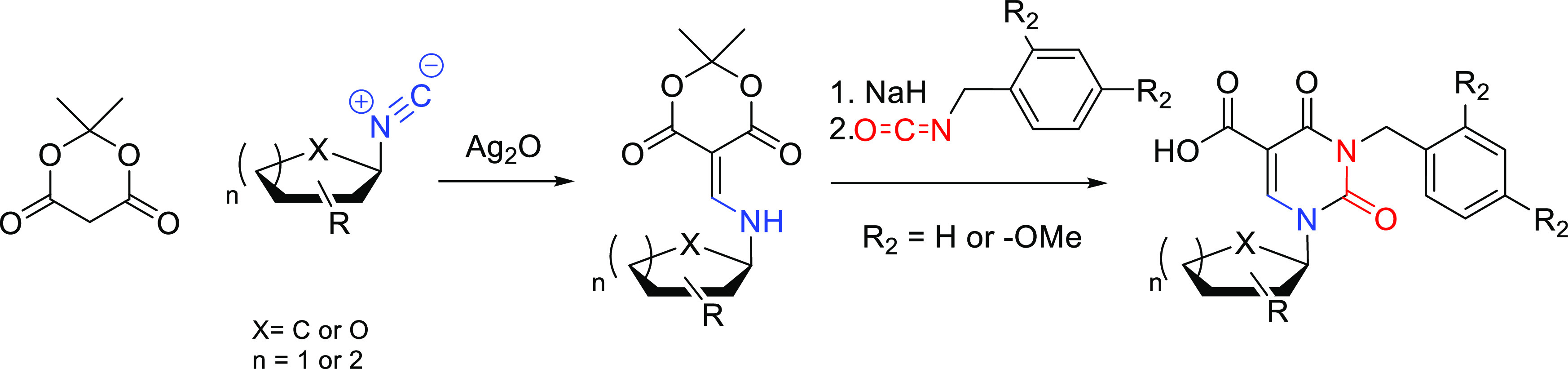

An efficient two-step procedure for the syntheses of pyrimidine nucleosides is presented. A series of glycosyl 5-(aminomethylene)-1,3-dioxane-4,6-dione derivatives were prepared from β-anomeric isonitriles by reaction with Meldrum’s acid or by allowing aminomethylene Meldrum’s acid to react with an 1-aldofuranosyl halide or acetate. The resultant 5-(aminomethylene)-1,3-dioxane-4,6-dione derivatives underwent reaction with benzyl- or 2,4-dimethoxybenzyl isocyanate via transacylation to provide uridine-5-carboxylic acid derivatives and related nucleosides. These nucleoside carboxylic acids were converted into other C-5 derivatives by bromo-decarboxylation with *N*-bromosuccinimide.

Many antiviral and anticancer drugs have been developed based on modification of the essential nucleosides in the furanosyl ring or base residue. Examples include gemcitabine (**1**), used for the treatment of ovarian, small lung, pancreatic, bladder, and breast cancers, and sofosbuvir (**2**) an effective medicine used in combination therapies to treat hepatitis C (Figure 1).^1,2^

**Figure 1:**
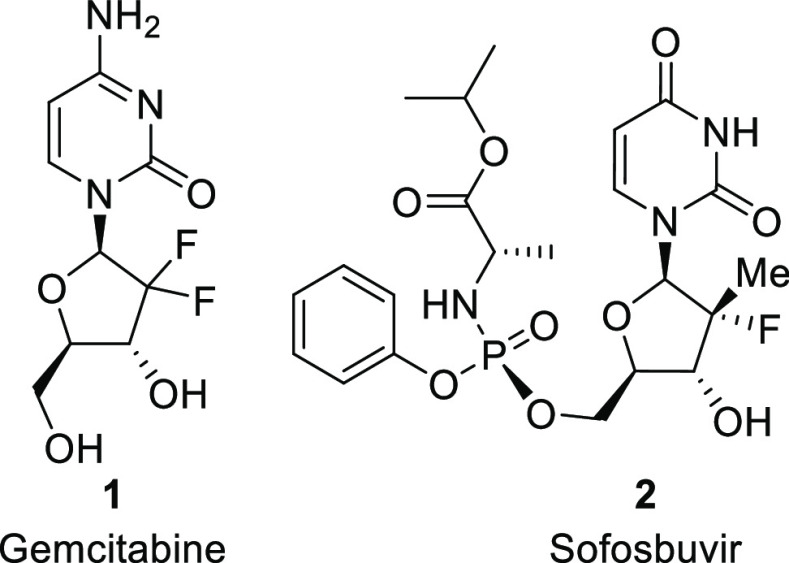
Structures of gemcitabine (**1**) and sofosbuvir (**2**).

There are three distinct strategies for the synthesis of nucleosides and their analogues: (1) condensation of an activated aldofuranose derivative with a base; (2) nucleoside modification by selective reaction of one or more groups in the ribofuranose unit or the base or both; and (3) derivatization of a β-aldofuranosyl amine with base construction via a heterocyclization reaction (Scheme 1). The most widely applied method (1), the Vorbrüggen reaction, involves a Lewis acid-catalyzed condensation reaction of protected and activated aldofuranose derivative, such as **3**, with persilylated nucleoside bases, like pyrimidine **4**.^3^ In general, these reactions with *O*-acylated ribofuranosyl derivates are highly β-selective due to neighboring group participation by the C-2 ester group via an acyloxonium ion. Second, method 2 has been used for the synthesis of non-natural nucleosides by means of selective protection and transformations of unprotected functional groups. For example, gemcitabine (**1**) has been synthesized by late-stage reaction of C-2′-ketone **6** using diethylaminosulfur trifluoride (DAST).^4^ An example of method 3 is shown with the reaction of β-d-ribosofuranosyl amine (**8**) with ethyl (*E*)-(3-ethoxy-2-cyanoacryloyl)carbamate (**9**) to produce the nucleoside (**10**) via a heterocyclization reaction.^5,6^ Each method has distinct advantages and disadvantages and usage of each method depends on the desired strategy of early- or late-stage modifications.

**Scheme 1:**
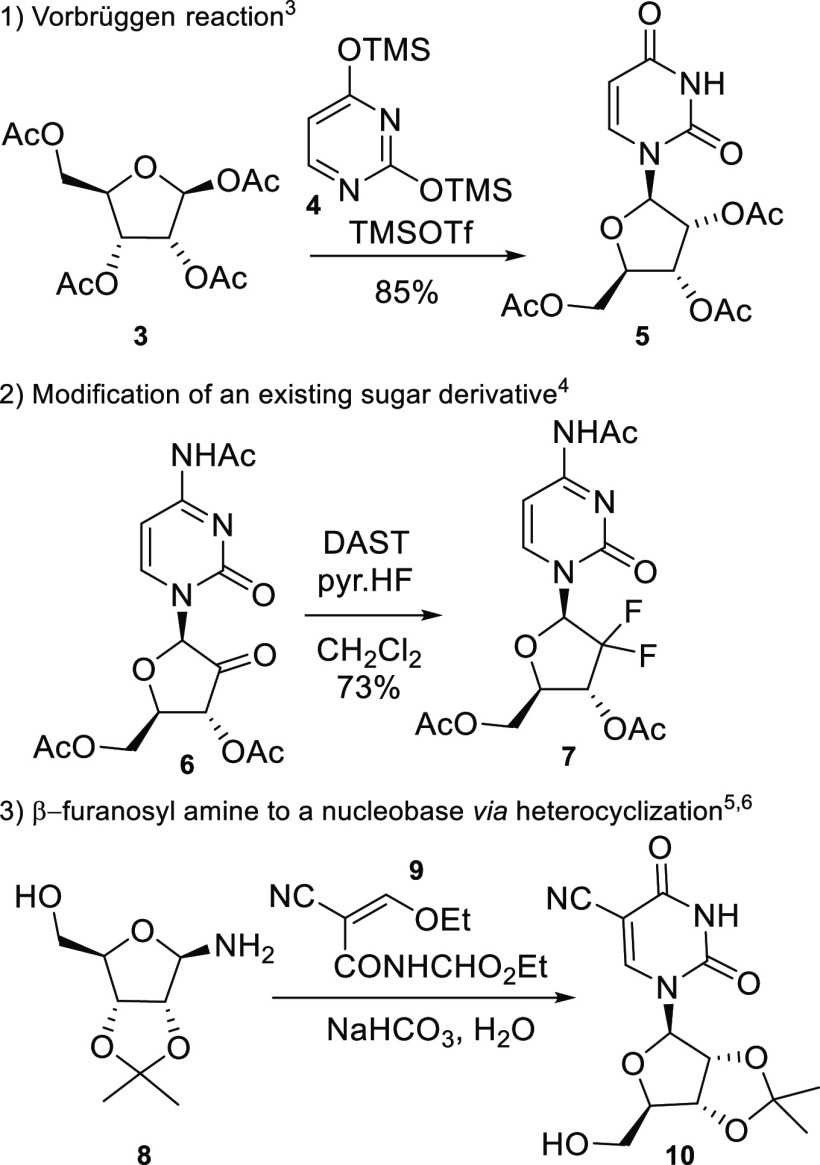
Three Distinct Strategies for the Synthesis of Modified Nucleosides

Herein we describe a new, complementary method for the synthesis of uracil and analogues, from furanosyl 5-(aminomethylene)-1,3-dioxane-4,6-dione derivatives by reactions with benzyl- or 2,4-dimethoxybenzyl isocyanates.

A series of aldofuranosyl isonitriles **11b** to **11f** were synthesized from the corresponding anomeric bromides or formamides respectively by reaction with silver cyanide or dehydration with triphosgene.^,^^7,8^ The corresponding precursors were synthesized according to adaptations of known literature procedures.^7−10^ Full methods are described in the experimental details. Reactions of the isonitriles **11a**–**11f** with Meldrum’s acid (**13**) provided the corresponding enamides (**15a**–**15f**) (Scheme 2). This reaction was known for alkyl isonitrile^11−13^ but had not been applied to anomeric sugar isonitriles. Based on known isonitrile reactivities, it is reasonable to speculate that the silver(I) cation binds to the isonitrile carbon thereby activating it toward reaction with the Meldrum’s acid enol resulting in α-addition.^14^ Overall yields were generally good (43–79%) with the exception of cyclohexyl isonitrile (**15a**) (30%). This α-addition reaction was applied to a variety of sugars (Scheme 2). Attempted application to deoxyribofuranose failed since the isonitrile **11g** could not be synthesized. Instead, Hoffer’s chlorosugar **12g** (X = Cl) was coupled with aminomethylene Meldrum’s acid (**14**) in the presence of sodium hydride to obtain the corresponding enamide **15g**. This procedure was applicable to aldofuranoses **12e** (X = Br) and **12g** (X = OAc); however, displacements with fluoro glycofuranoses **12c** and **12d** were unsuccessful. When present, undesired α-epimers of the 5-(aminomethylene)-1,3-dioxane-4,6-dione derivatives **15** were easily removed by chromatography. The structure of adduct **15d** was confirmed by single crystal X-ray structure determination (see Supporting Information).

**Scheme 2:**
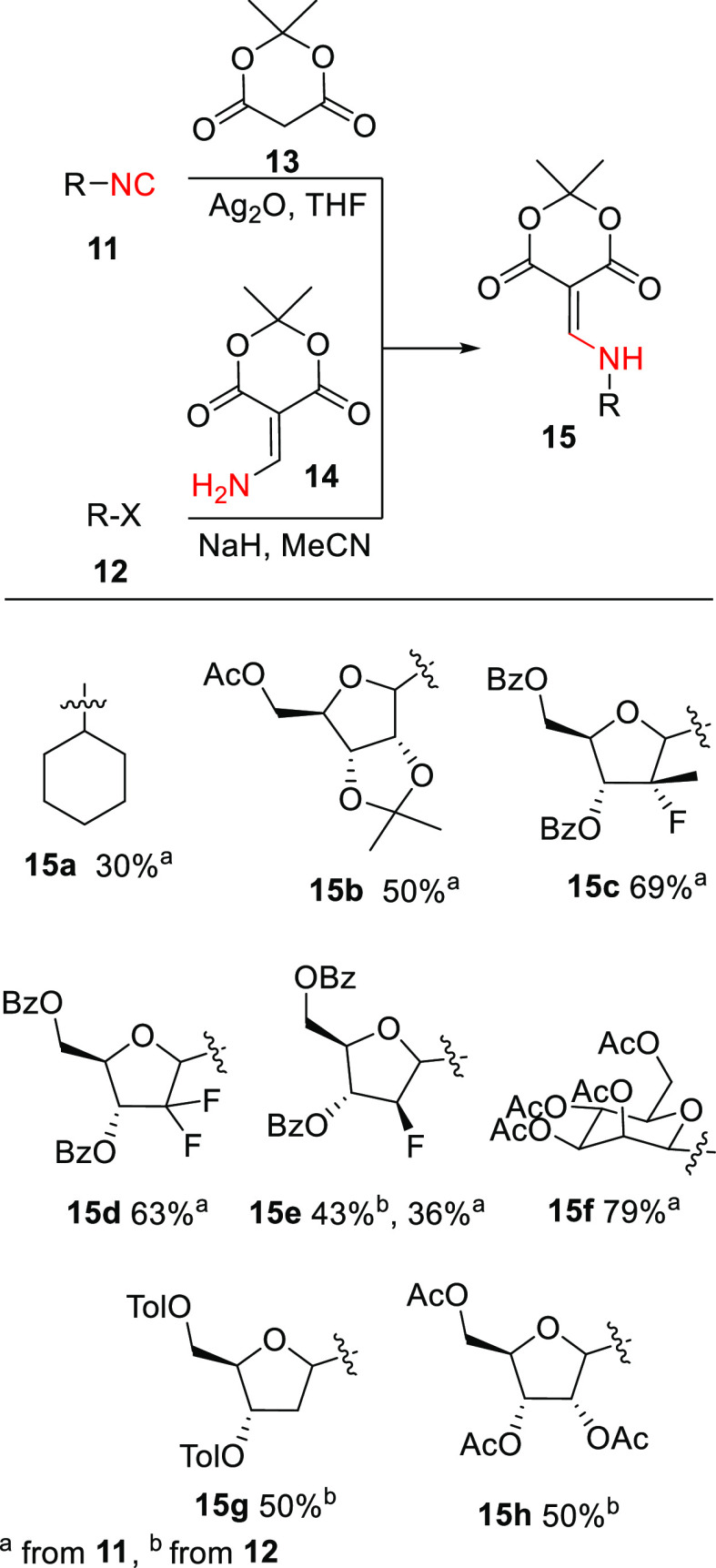
Enamide **15** Syntheses

The anomeric enamides **15** were allowed to react with benzyl isocyanate in the presence of sodium hydride in THF at 60 °C to give the corresponding nucleoside carboxylic acids **16** (Scheme 3). ^1^H NMR monitoring of the reactions was consistent with complete consumption of starting material and conversion to uracils **16** on overnight reaction with excess isocyanate; however, difficulties in purification (urea formation and H-bonding with carboxylic acid entity) complicated preparative scale synthesis and were only appropriate on a small scale for full structural analysis.

**Scheme 3:**
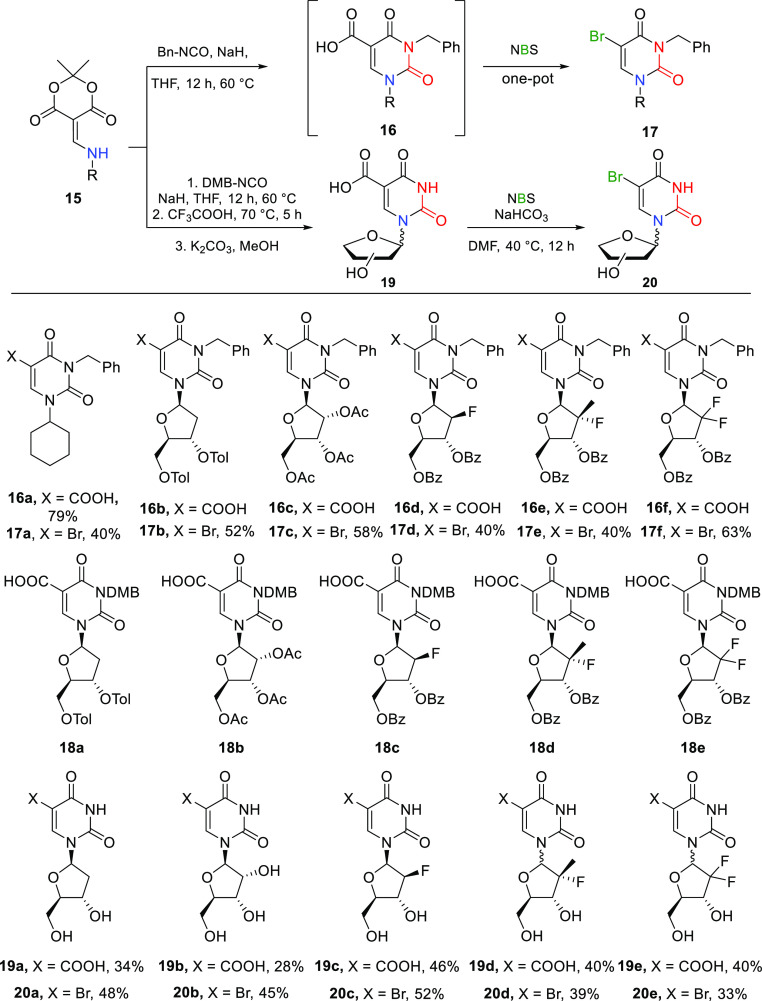
Heterocyclization with Benzyl Isocyanate and Subsequent Bromodecarboxylation Using NBS and Cyclization with 2,4-Dimethoxybenzyl Isocyanate and Derivatization of Nucleoside Core

This limitation was overcome by direct bromo-decarboxylation of the crude products **16** using *N*-bromosuccinimide (NBS) to provide the bromo-nucleosides **17a**–**17f** (40–63% over two steps). Isolation was possible for carboxylic acid **16a** only, for which additional halodecarboxylations were performed with NIS and NCS (see experimental details). Attempted deprotection of the base *N*-benzyl groups was not achieved, and such difficulties with related C–N bond cleavage reactions have precedent.^15^ Alternatively, heterocyclizations using 2,4-dimethoxybenzyl (DMB) isocyanate under equivalent basic conditions were examined (Scheme 3). These reactions gave the corresponding nucleosides **18a**–**18e** on a small scale for full structural analysis. Direct, 2,4-dimethoxybenzyl group deprotection of the crude products using trifluoroacetic acid at 70 °C followed by saponification with potassium carbonate in methanol (removal of acetate, benzoate, p-toluoyl groups) gave the nucleoside carboxylic acids **19a**–**19e** (32–58% over two steps). The structures of adducts **19a** and **19e** were confirmed by single crystal X-ray structure determinations (see Supporting Information). When present, undesired α-epimers were removed by chromatography. Related 5-carboxynucleosides have been shown to be involved in the regulation of gene expression.^16−18^ The carboxylic acid group of the nucleosides **19** was derivatized by decarboxylative halogenation using *N*-bromosuccinimide (NBS) in DMF at 40 °C, presumably via stepwise halogenation followed by decarboxylation, giving bromo-nucleosides **20a**–**20e** in 33%–52% yield. Related bromo-uridine derivatives undergo palladium-catalyzed coupling reactions.^19,20^

At this stage it is germane to further comment on the mechanism of the isocyanate reactions. Computational studies using density functional theory (B3LYP+GD3-BJ/Def2-SVP and Def2-TZVPP/SCRF = thf) gave the free energies at key points on the reaction profile. A model was constructed which also included Na^+^ coordinated with two additional explicit THF solvent molecules. Although the presence of a Meldrum’s acid entity on the enamides **15a**–**15g** is consistent with the possibility of a formal [4 + 2] cycloaddition via ketene formation, the calculations revealed a high energy barrier of 45 kcal for such a pathway. A transacylation mechanism was shown to be more probable (Scheme 4). Such a mechanistic pathway involves three distinct transition states and two intermediates. Scheme 4 displays a representation of the three transition states, all of which have lower, thermally accessible, free energy barriers (<27 kcal/mol). The first transition state (TS1) refers to the addition of the nitrogen anion on the carbon center of the isocyanate (18.5 kcal/mol). The second transition state (TS2) involves the urea nitrogen anion adding to the carbonyl group of Meldrum’s acid (26.8 kcal/mol). The last transition state (TS3) corresponds to the release of acetone and formation of the carboxylate (23.1 kcal/mol), with the final product being exoenergic by −17.6 kcal/mol with respect to reactants. Further calculations using different substituted riboses demonstrated that TS2 and TS3 can be similar in free energy and either can be the overall rate-limiting step for the cyclization.

**Scheme 4:**
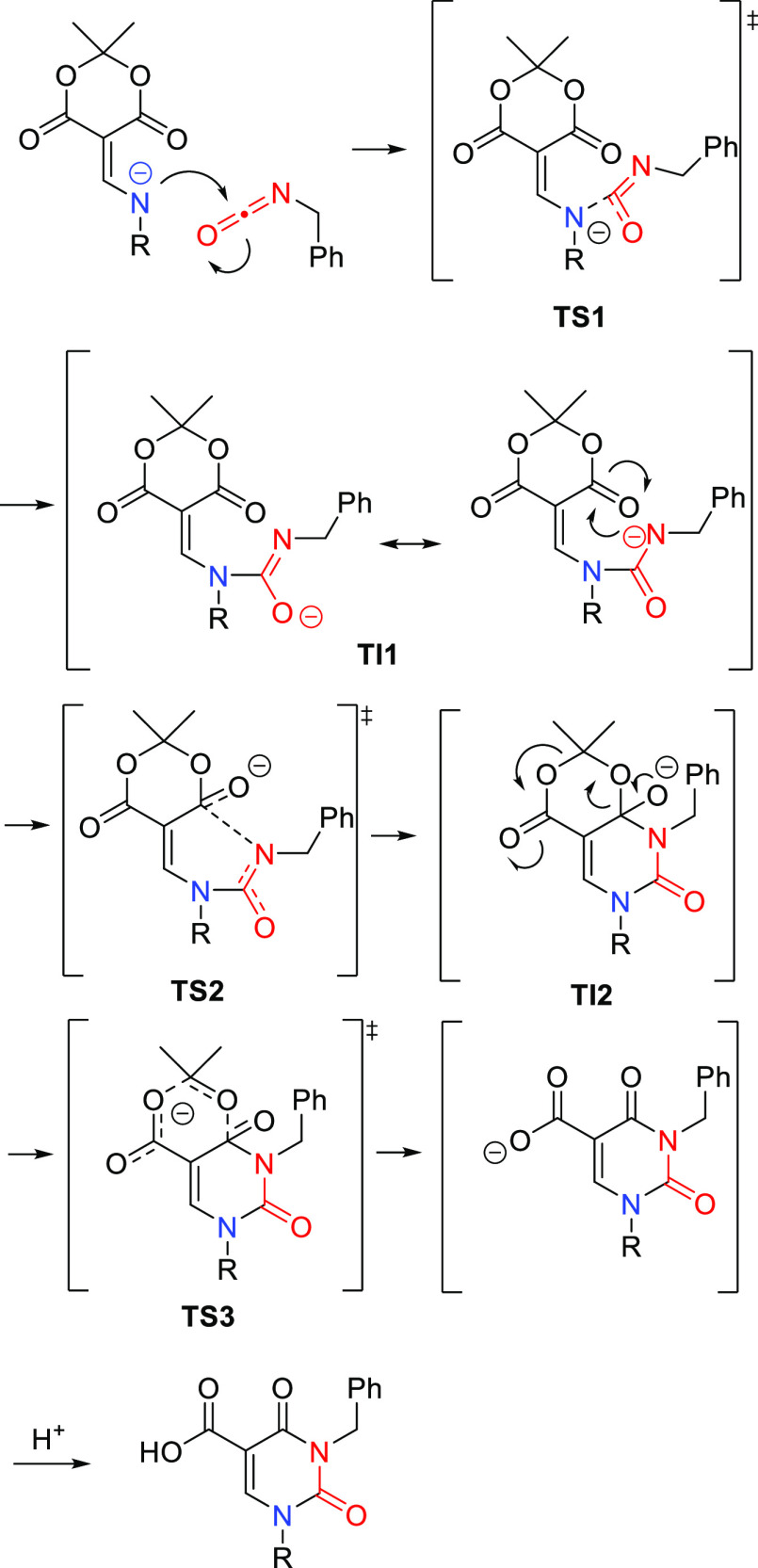
Calculated Transition States in the Heterocyclization Reaction

In conclusion, a novel pyrimidine nucleoside synthesis was developed involving the stepwise cyclization of 5-(aminomethylene)-1,3-dioxane-4,6-dione derivatives with benzyl isocyanate or 2,4-dimethoxylbenzyl isocyanate via transacylation to provide the 5-carboxyuracil nucleosides.

## Data Availability

The data underlying this study are available in the published article, in its online Supporting Information and openly available in the Spiral repository at 10.14469/hpc/6268 and 10.14469/hpc/6287.

## Supporting Information Available

Experimental details (PDF)
NMR spectra and crystal data (PDF)
