# Supplementary material for: Pyrimidine Nucleosides Syntheses by Late-Stage Base Heterocyclization Reactions
Source: Org Lett. 2022 Nov 4;24(49):8931–5. doi: 10.1021/acs.orglett.2c03152 (PMC9764413; doi:10.1021/acs.orglett.2c03152)
Supplement: Supplementary file 1 — ol2c03152_si_001.pdf [file ol2c03152_si_001.pdf]

Pyrimidine Nucleosides Syntheses by Late-Stage Base Heterocyclization Reactions.

Elfie S. Cavalli, Thomas Mies, Henry S. Rzepa, Andrew J. P. White, Philip J. Parsons, Anthony G.M. Barrett*

Department of Chemistry, Imperial College, London, W12 0BZ, England

Supporting Information (SI)

Table of Contents

I. General Information 2

II. Syntheses of isonitrile ribofuranoses....3

1. Synthesis of 2,3,4,6-tetra-*O*-acetyl-α-*D*-mannopyranosyl isonitrile....3
2. Synthesis of 5-*O*-acetyl-2,3-di-*O*-iso-propylidene-β-*D*-ribofuranosyl isonitrile ...5
3. Synthesis of 3,5-di-*O*-benzoyl-2-deoxy-2,2-difluoro-β-*D*-ribofuranosyl isonitrile6
4. Synthesis of 3,5-Di-*O*-benzoyl-2-deoxy-2-fluoro-2-methyl-*D*-ribofuranosyl isonitrile 10
5. Synthesis of 3,5-di-*O*-benzoyl-2-deoxy-2-fluoro-β-*D*-arabinofuranosyl isonitrile 13

III. Synthesis of 5-(Aminomethylene)-1,3-dioxane-4,6-dione derivatives 15

1. Synthesis of 5-((Cyclohexylamino)methylene)-2,2-dimethyl-1,3-dioxane-4,6-dione 15
2. Synthesis 5-(5-*O*-acetyl-2,3-di-*O*-iso-propylidene-β-*D*-ribofuranosylamino-methylene)-2,2-dimethyl-1,3-dioxa-4,6-dione 16
3. Synthesis of 5-(3,5-di-*O*-benzoyl-2-deoxy-2-fluoro-2-methyl-*D*-ribofuranosylamino-methylene)-2,2-dimethyl-1,3-dioxa-4,6-dione 17
4. Synthesis of 5-(3,5-di-*O*-benzoyl-2-deoxy-2,2-difluoro-*D*-ribofuranosylamino-methylene)-2,2-dimethyl-1,3-dioxa-4,6-dione 18
5. Synthesis of 5-(2,3,4,6-tetra-*O*-acetyl-α-*D*-mannopyranosylamino-methylene)-2,2-dimethyl-1,3-dioxa-4,6-dione....19
6. Synthesis of 5-(2,3,5-tri-*O*-acetyl-β-*D*-ribofuranosylamino-methylene)-2,2-dimethyl-1,3-dioxa-4,6-dione 20
7. Synthesis of 5-(3,5-di-*O*-benzoyl-2-deoxy-2-fluoro-β-*D*-arabinofuranosylamino-methylene)-2,2-dimethyl-1,3-dioxa-4,6-dione 23
8. Synthesis of 5-(3,5-di-*O*-(4-methylbenzoyl)-2-deoxy-β-*D*-ribofuranosylamino-methylene)-2,2-dimethyl-1,3-dioxa-4,6-dione 24

IV. Cyclisation with Benzyl Isocyanate and Decarboxylative Bromination....25

V. Cyclization with 2,4-Dimethoxybenzyl isocyanate and Full Deprotection 36

VI. Bromo-decarboxylation Reactions of Uridine-5-carboxylic Acid Derivatives 45

I. General Information

Solvents used for extraction and purification were HPLC grade. Unless otherwise indicated, all reactions were run in oven-dried glassware under an inert atmosphere of nitrogen or argon using anhydrous conditions. Anhydrous chemicals were obtained commercially as such. Cyclohexyl isonitrile (**11a**) was purchased from commercial vendors and used as received. NaH refers to a 60% dispersion in mineral oil unless otherwise stated. Unless stated to the contrary, reactions were carried out at room temperature, when reaction temperatures refer to the external bath temperature. In all cases, DrySyn heating mantels were used for reactions at elevated temperatures. All reactions were monitored by thin-layer chromatography (TLC) using pre-coated silica gel plates (250 mm, 60 F_{254}). Spots were visualized using 254 nm ultraviolet light or by staining with acidic vanillin, potassium permanganate, ninhydrin or bromocresol green, as appropriate. Chromatographic purifications were performed on silica gel (particle size 32-63 microns) using the solvent systems described in the experimental procedures. Pentane refers to the alkane fraction with a boiling point range of 40 and 60 °C. Infrared (IR) spectra were recorded neat with absorptions given in wavenumber (cm^{-1}). ^{1}H-, $^{13}C\{^{1}H\}$- and $^{19}F\{^{1}H\}$-NMR spectra were recorded at 400 or 500 MHz, in an appropriate deuterated solvent. Chemical shifts are reported relative to internal solvent in ppm (parts per million). Carbon multiplicities were determined using DEPT experiments. The signal multiplicity reported in the experimental is quoted as follows: coupling constant (*J*), singlet (s), doublet (d), triplet (t), quadruplet (q), quintuplet (quin), sextet (sext), septet (sep), broad (br), multiplet (m), double doublet (dd), double double doublet (ddd), doublet of double quartet (ddq), quartet of triplets (qt), quartet of doublet (qd) or a combination thereof. High resolution mass spectra (HRMS) were recorded by the Imperial College Mass Spectrometry Service under conditions of electrospray ionization (ESI), chemical ionization (CI), or electron ionization (EI). Melting points were obtained using a melting point hot stage and are uncorrected. Optical rotation values were recorded in $CHCl_3$, MeOH or H_2O, as appropriate. When appropriate, purification was performed by C18 reverse phase chromatography.

II. Syntheses of isonitrile ribofuranoses

1. Synthesis of 2,3,4,6-tetra-*O*-acetyl-α-*D*-mannopyranosyl isonitrile

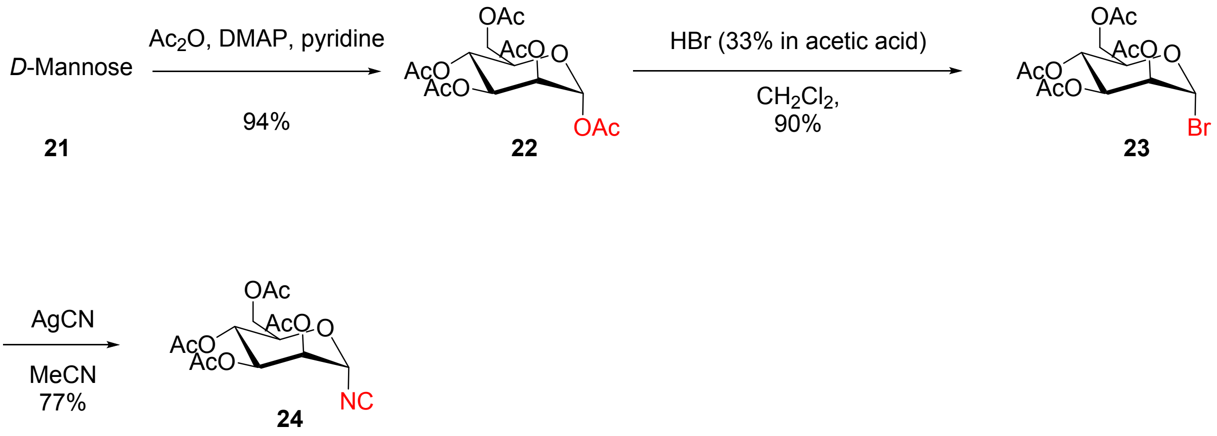

2,3,4,6-Tetra-*O*-acetyl-α-*D*-mannopyranosyl acetate 22

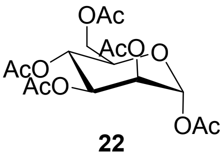

22

Ac_2O (17.7 mL, 187 mmol, 5.50 equiv), DMAP (415 mg, 3.40 mmol, 0.1 equiv) and pyridine (70 mL) were added sequentially with stirring to D-mannose **21** (6.13 g, 34.0 mmol, 1.0 equiv) at 0 °C and the mixture was allowed to warm up to 25 °C. After stirring the mixture overnight, reaction was quenched with H_2O (40 mL) and saturated aqueous $CuSO_4$ (40 mL), when Et_2O (80 mL) was added, and the organic layer was washed with saturated aqueous $CuSO_4$ (4 × 40 mL) and dried ($MgSO_4$). The remaining solvent was removed *in vacuo* to leave the penta-acetate **22** (12.6 g, 32.3 mmol, 94%) as a white solid containing only the α-anomer **22**: R_f 0.34 (EtOAc : pentane, 4:6); m.p. (Et_2O) 63.0 - 66.1 °C (lit.[1] 60 - 61 °C)**;** $[\alpha]_D^{21.7}$+ 43.4 (*c* 1.0, $CHCl_3$); IR ν_{max} 1747, 1434, 1368, 1215, 1148 cm^{-1}; ^1H-NMR (400 MHz, $CDCl_3$) δ 6.08 (d, *J* = 2.6 Hz, 1H), 5.38-5.32 (m, 2H), 5.27-5.25 (t, *J* = 2.3 Hz, 1H), 4.28 (dd, *J* = 12.4, 4.9 Hz, 1H), 4.14-4.03 (m, 2H), 2.18 (s, 3H), 2.17 (s, 3H), 2.09 (s, 3H), 2.05 (s, 3H), 2.01 (s, 3H); $^{13}C\{^1H\}$-NMR (101 MHz, $CDCl_3$) δ 170.6, 169.8, 169.7, 169.5, 168.0, 90.6, 70.6, 68.7, 68.3, 65.5, 62.1, 20.8, 20.78, 20.73, 20.6, 20.6; HRMS (MS ES-ToF) *m/z* $[M + Na]^+$: Calcd for $(C_{16}H_{22}O_{11}Na)^+$: 413.1060 Found 413.1068; Analytical data (^1H NMR, ^{13}C NMR) were in good agreement with reported values.[1]

2,3,4,6-Tetra-*O*-acetyl-α-*D*-mannopyranosyl bromide 23

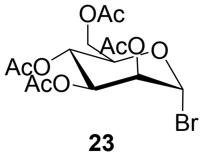

23

HBr (33% in AcOH; 25 mL) was added with stirring to the mannopyranose **22** (3.30 g, 8.45 mmol, 1 equiv) in dry CH_2Cl_2 (20 mL) at 0 °C and the reaction mixture was allowed to warm up to 25 °C. After 2.5 h, reaction was quenched with H_2O (50 mL) and the mixture was extracted with CH_2Cl_2 (2 x 50 mL). The combined organic extracts were washed with saturated aqueous $NaHCO_3$ (50 mL, exothermic) and brine (50 mL), dried ($MgSO_4$), filtered, and concentrated *in vacuo*. Chromatography (EtOAc : pentane, 1 : 1) gave α-bromide **23** (2.88 g, 7.00 mmol, 90%) as a white foam: R_f 0.46 (pentane : EtOAc, 7:3); ^{1}H-NMR (400 MHz, $CDCl_3$) δ 6.28 (d, *J* = 1.7 Hz, 1H), 5.70 (dd, *J* = 9.9, 3.4 Hz, 1H), 5.43 (dd, *J* = 3.4, 1.9 Hz, 1H), 5.36 (t, *J* = 10.1 Hz, 1H), 4.32 (dd, *J* = 12.3, 4.9 Hz, 1H), 4.21 (ddt, *J* = 13.0, 5.0, 2.4 Hz, 1H), 4.12 (dd, *J* = 12.5, 2.2 Hz, 1H), 2.17 (s, 3H), 2.10 (d, *J* = 1.6 Hz, 3H), 2.06 (s, 3H), 2.00 (s, 3H); ^{13}C{^{1}H}-NMR (101 MHz, $CDCl_3$) δ 170.5, 169.7, 169.5, 83.0, 72.8, 72.1, 67.9, 65.3, 61.4, 20.7, 20.6, 20.6, 20.5; Analytical data (^{1}H NMR, ^{13}C NMR) were in good agreement with reported values.[2] Bromide **23** was directly converted into isonitrile **24** without further purification.

2,3,4,6-Tetra-*O*-acetyl-α-*D*-mannopyranosyl isonitrile 24

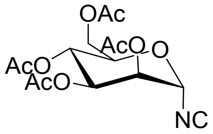

24

AgCN (531 mg, 3.96 mmol, 3 equiv) was added with stirring to bromide **23** (548 mg, 1.32 mmol, 1 equiv) in dry MeCN (12 mL). The mixture was left stirring overnight at 82 °C, after which it was filtered to remove the silver salt and concentrated *in vacuo*. Chromatography (Et_2O : pentane; 7 : 3) gave isocyanide **24** (364 mg. 1.01 mmol, 77%) as a colorless oil: R_f 0.71 (pentane : EtOAc, 7:3); $[\alpha]_D^{24.4}$+ 53.4 (*c* 1.0, $CHCl_3$); ^{1}H-NMR (400 MHz, $CDCl_3$) δ 5.44 – 5.24 (m, 4H), 4.33 (dd, *J* = 12.7, 5.1 Hz, 1H), 4.19 – 4.08 (m, 2H), 2.18 (s, 3H), 2.10 (s, 3H), 2.07 (s, 3H), 2.01 (s, 3H); ^{13}C{^{1}H}-NMR (101 MHz, $CDCl_3$) δ 170.4, 169.5, 169.5, 166.5, 78.9, 71.9, 69.1, 67.8, 64.9, 61.6, 20.7, 20.6, 20.5; IR v_{max} cm^{-1} 2122, 1740, 1431, 1367, 1205. HRMS (APCI) *m/z*: $[M + H]^+$ Calcd for $(C_{15}H_{20}NO_9)^+$: 358.1133; Found 358.1120.

2. Synthesis of 5-*O*-acetyl-2,3-di-*O*-iso-propylidene-β-*D*-ribofuranosyl isonitrile

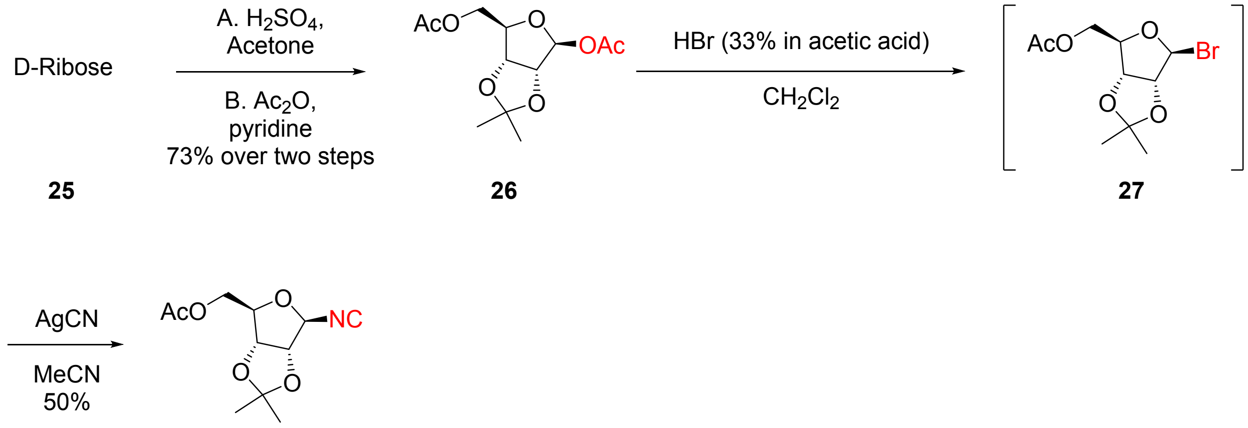

28

5-*O*-Acetyl-2,3-di-*O*-iso-propylidene-β-*D*-ribofuranosyl acetate[3] (26)

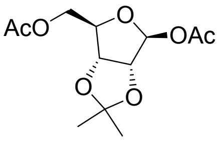

26

H_2SO_4 (0.15 mL) was added dropwise with stirring to D-ribose **25** (5.00 g, 33.3 mmol, 1 equiv) in Me_2CO (50 mL). After 2h at 25 °C, reaction was quenched with saturated aqueous $NaHCO_3$ (20 mL), and the resultant mixture filtered through celite and concentrated *in vacuo*. Ac_2O (17.3 mL) was added with stirring to the crude residue in pyridine (60 mL) at 0 °C and the reaction mixture was allowed to warm up to 25 °C. After stirring overnight, reaction was quenched with saturated aqueous $CuSO_4$ (100 mL) and the mixture was extracted with EtOAc (2 x 50 mL). The combined organic extracts were washed with saturated aqueous $CuSO_4$ (50 mL), dried ($MgSO_4$), filtered, and concentrated *in vacuo.* Chromatography (pentane : EtOAc : 1 : 1) gave ribose **26** (6.67 g. 24.3 mmol, 73%) as a colorless oil containing only the *β*-anomer: R_f 0.62 (pentane : EtOAc, 7:3); $[\alpha]_D^{22.3} = -48.4$ (*c* 1.0, $CHCl_3$); ^{1}H-NMR (400 MHz, $CDCl_3$) δ 6.20 (s, 1H), 4.70 (s, 2H), 4.45 (t, *J* = 6.7 Hz, 1H), 4.11 (dd, *J* = 6.9, 3.5 Hz, 2H), 2.08 (s, 3H), 2.04 (s, 3H), 1.48 (s, 3H), 1.32 (s, 3H); ^{13}C{^{1}H}-NMR (101 MHz, $CDCl_3$) δ 170.5, 169.3, 113.2, 102.1, 85.4, 85.1, 81.6, 64.1, 26.4, 25.1, 21.2, 20.8; IR ν_{max} 1738, 1371, 1207 cm^{-1}; HRMS (ESI) *m/z*: $[M + Na]^+$ Calcd for $(C_{12}H_{18}O_7Na)^+$: 297.0950; Found 297.0941. Analytical data (^{1}H NMR, ^{13}C NMR, IR and HRMS) were in good agreement with reported values.[3]

5-*O*-Acetyl-2,3-di-*O*-iso-propylidene-β-*D*-ribofuranosyl isonitrile (28)

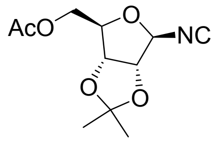

28

HBr (33% in AcOH, 2 mL) was added with stirring to ribofuranosyl acetate **26** (438 mg, 1.60 mmol) in dry CH_2Cl_2 (20 mL) at 0 °C and the reaction mixture was allowed to warm up to 25 °C. After 20 min, reaction was quenched with H_2O (20 mL) and the mixture was extracted with CH_2Cl_2 (2 x 15 mL). The combined organic extracts were washed with saturated aqueous $NaHCO_3$ (10 mL) and brine (10 mL), dried ($MgSO_4$), filtered, and concentrated *in vacuo*. AgCN (239 mg, 0.815 mmol, 1 equiv) was added with stirring to the crude bromide **27** in MeCN (7 mL). After stirring overnight, the solution was concentrated *in vacuo* and chromatography of the residue (Et_2O: pentane : 4 : 6) gave isocyanide **28** (78.0 mg, 3.23 mmol, 28%) as a colorless oil: R_f 0.58 (pentane : EtOAc, 7 : 3); $[\alpha]_D^{25.9} = -39.4$ (*c* 1.0, $CHCl_3$); ^{1}H-NMR (500 MHz, $CDCl_3$) δ 5.32 – 5.26 (m, 1H), 4.93 (dt, *J* = 5.9, 0.7 Hz, 1H), 4.84 (ddd, *J* = 5.8, 1.3, 0.5 Hz, 1H), 4.53 (td, *J* = 5.0, 1.3 Hz, 1H), 4.34 – 4.19 (m, 2H), 2.14 (s, 3H), 1.48 (d, *J* = 0.8 Hz, 3H), 1.34 (d, *J* = 0.8 Hz, 3H); $^{13}C\{^1H\}$-NMR (101 MHz, $CDCl_3$) δ 170.3, 162.3, 114.0, 88.3, 87.4, 86.4, 81.9, 63.6, 26.46, 25.0, 21.0; IR ν_{max} 2128, 1744, 1375, 1231 cm^{-1}; HRMS (APCI) *m/z*: $[M - NC]^+$ Calcd for $(C_{11}H_{15}O_5)^+$: 215.0914; Found 215.0915.

3. Synthesis of 3,5-di-*O*-benzoyl-2-deoxy-2,2-difluoro-β-*D*-ribofuranosyl isonitrile

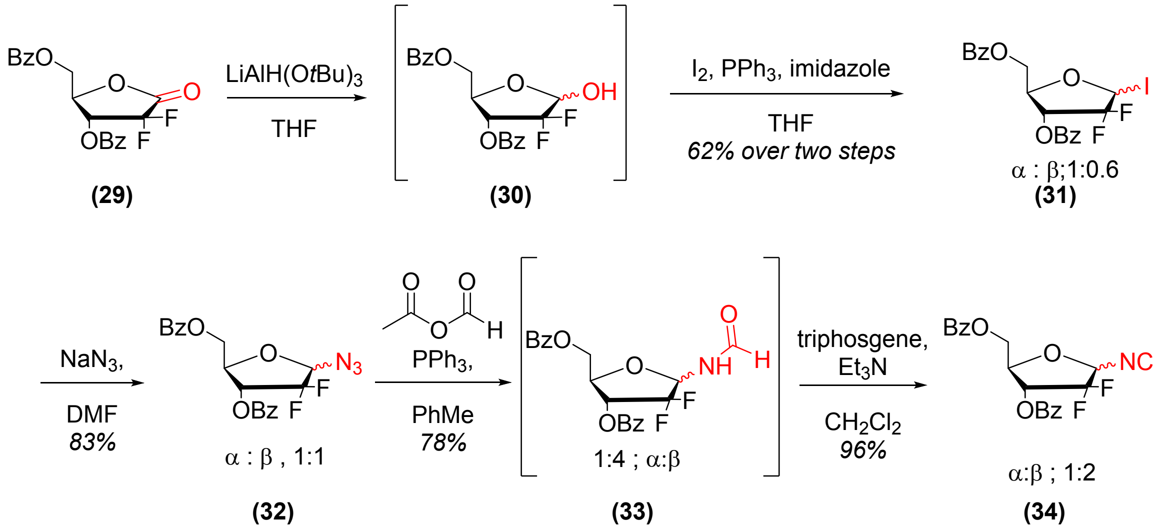

3,5-Di-*O*-benzoyl-2-deoxy-2,2-difluoro-β-*D*-ribofuranosyl iodide [4] (31)

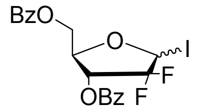

(31)

LiAlH(O-*t*-Bu)$_3$ (134 mg, 0.527 mmol, 2 equiv) was added portion-wise with stirring to lactone (**29)** (100 mg, 0.265 mmol, 1 equiv) in Et_2O and THF (1 : 1; 10 mL) at 0 °C. After 1h, reaction was quenched with MeOH (5 mL) and water (15 mL), and the mixture was extracted with Et_2O (2 x 20 mL). The combined organic extracts were washed with aqueous HCl (1M), saturated aqueous $NaHCO_3$ (5 mL) and saturated aqueous Rochelle salt (5 mL), dried ($MgSO_4$), filtered and concentrated *in vacuo* to leave a colorless oil containing the crude alcohol **30**. PPh_3 (69.0 mg, 0.265 mmol, 1 equiv), I_2 (68.0 mg, 0.265 mmol, 1 equiv) and imidazole (18.0 mg, 0.265 mmol, 1 equiv) were added sequentially with stirring to this oil in CH_2Cl_2 (5 mL) at 25 °C. After 3h, the mixture was concentrated *in vacuo* and chromatography of the residue (EtOAc : pentane 1:9) gave iodide **31** (81.0 mg, 0.165 mmol, 62%, α : β, 1:0.6) as a colorless oil. Partial epimerization of **31** occurred over time leading to a 1:1 ratio of anomers: R_f 0.8 (10% EtOAc : pentane 1 : 9); $[\boldsymbol{\alpha}]_{\boldsymbol{D}}^{22.1} = +73.6$ (*c* 1.0, $CHCl_3$); ^{1}H-NMR For *α*-anomer (400 MHz, $CDCl_3$) δ 8.16 (dd, *J* = 8.4, 1.4 Hz, 2H), 8.11 – 8.00 (m, 2H), 7.65 (s, 1H), 7.58 (s, 1H), 7.55 – 7.47 (m, 2H), 7.47 – 7.41 (m, 2H), 6.92 (dd, *J* = 11.1, 1.2 Hz, 1H), 5.57 (ddd, *J* = 17.2, 4.7, 1.2 Hz, 1H), 4.85 – 4.62 (m, 3H); ^{1}H NMR For *β*-anomer (400 MHz, $CDCl_3$) δ 8.12 – 8.00 (m, 3H), 7.69 – 7.54 (m, 2H), 7.54 – 7.47 (m, 2H), 7.47 – 7.37 (m, 3H), 6.73 (dd, *J* = 11.1, 2.0 Hz, 1H), 6.26 – 6.11 (m, 1H), 4.86 – 4.61 (m, 3H); ^{13}C{^{1}H}-NMR Mix of *α- and β-* (101 MHz, $CDCl_3$) δ 165.9, 165.1, 134.1, 133.5, 130.3, 129.8, 129.2, 128.7, 128.5, 128.12, 121.4, 83.0, 72.0, 71.8, 71.6, 71.5, 66.4, 66.1, 65.7, 61.7; ^{19}F{^{1}H}-NMR For *α*- anomer (377 MHz, $CDCl_3$) δ -100.24, -100.87, -104.82, -105.45; ^{19}F{^{1}H}-NMR For *β*- anomer (377 MHz, $CDCl_3$) δ -105.96, -106.56, -120.91, -121.51, -121.51; IR v_{max} 1724, 1264, 1059, 707 cm^{-1}; HRMS (APCI) *m/z*: $[M + H]^+$ Calcd for $(C_{19}H_{16}F_2IO_5)^+$: 489.0005; Found 489.0005. Analytical data (^{1}H NMR, ^{13}C NMR, ^{19}F NMR and HRMS) were in good agreement with reported values.[4]

3,5-Di-*O*-benzoyl-2-deoxy-2,2-difluoro-β-*D*-ribofuranosyl azide (32)

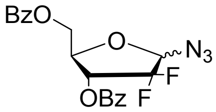

(32)

NaN_3 (121 mg, 0.186 mmol, 2 equiv) was added with stirring to the ribofuranosyl iodide **31** (454 mg, 0.931 mmol, 1 equiv) in DMF (22 mL). The mixture was left stirring overnight at 50

°C and was quenched with H_2O (25 mL), when LiCl (200 mg) was added to facilitate phase separation and the resultant mixture was extracted with EtOAc (2 x 25 mL). The combined organic extracts were washed with saturated aqueous $NaHCO_3$, dried ($MgSO_4$), filtered and concentrated *in vacuo.* Chromatography (Et_2O : pentane 1 : 4) gave azide **32** as a mixture of anomers (312 mg, 0.772 mmol, 83%, α : β, 1 : 1). Separation of the anomers was carried out by re-chromatography (Et_2O : pentane 1 : 9) giving the α- anomer as a colorless oil, and the β-anomer as a white solid: R_f α- anomer 0.6 (Et_2O : pentane 1 : 4); R_f β- anomer 0.4 (Et_2O : pentane 1 : 4); m.p. β-anomer (Et_2O/pentane) (62.5 - 63.4 °C); α- anomer $[\boldsymbol{\alpha}]_{\boldsymbol{D}}^{25.6} = +143$ (*c* 1.0, $CHCl_3$); β- anomer $[\boldsymbol{\alpha}]_{\boldsymbol{D}}^{25.6} = -67.1$ (*c* 1.0, $CHCl_3$); ^{1}H-NMR α- anomer (400 MHz, $CDCl_3$) δ 8.07 (ddd, *J* = 14.1, 8.3, 1.3 Hz, 4H), 7.66 – 7.54 (m, 2H), 7.53 – 7.39 (m, 4H), 5.59 – 5.48 (m, 2H), 4.76 (dt, *J* = 8.9, 3.1 Hz, 2H), 4.63 (dd, *J* = 13.1, 5.3 Hz, 1H); ^{1}H-NMR β- anomer (400 MHz, $CDCl_3$) δ 8.13 – 7.98 (m, 4H), 7.67 – 7.52 (m, 2H), 7.45 (dddd, *J* = 19.1, 7.5, 6.4, 1.7 Hz, 4H), 5.76 (td, *J* = 9.9, 6.3 Hz, 1H), 5.27 (dd, *J* = 7.8, 4.3 Hz, 1H), 4.74 (ddd, *J* = 12.0, 3.7, 1.1 Hz, 1H), 4.62 – 4.45 (m, 2H); $^{13}C\{^1H\}$-NMR α- anomer (101 MHz, $CDCl_3$) δ 166.0, 165.0, 134.0, 133.4, 130.1, 129.8, 129.3, 128.7, 128.5, 128.2, 124.3, 121.8, 121.5, 119.1, 90.9, 90.6, 90.4, 90.2, 81.2, 81.2, 81.2, 71.9, 71.7, 71.6, 71.4, 62.8; $^{13}C\{^1H\}$-NMR β- anomer (101 MHz, $CDCl_3$) δ 166.0, 164.8, 134.1, 134.1, 133.4, 130.1, 129.8, 129.8, 129.3, 128.7, 128.5, 128.1, 123.9, 121.3, 121.2, 118.7, 89.5, 89.2, 89.1, 88.9, 78.3, 78.6, 71.1, 70.9, 70.8, 70.7, 63.1; $^{19}F\{^1H\}$-NMR α- anomer (377 MHz, $CDCl_3$) δ -105.44, -106.11, -122.38, -123.05; $^{19}F\{^1H\}$-NMR β- anomer (377 MHz, $CDCl_3$) δ -119.53, -120.16, -121.51, -122.15; IR v_{max} α- anomer 2117, 1718, 1244, 704 cm^{-1}; IR v_{max} β- anomer 2119, 1722, 1244, 704 cm^{-1}; HRMS (APCI) *m/z*: [M + H]$^+$ Calcd for $(C_{19}H_{16}F_2N_3O_5)^+$: 404.1053; Found 404.1043. The assignment of anomers was based on a comparison with literature data [5]

3,5-Di-*O*-benzoyl-2-deoxy-2,2-difluoro-β-*D*-ribofuranosyl isonitrile (34)

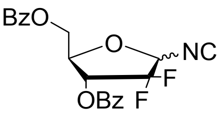

(34)

PPh_3 (718 mg, 2.74 mmol, 1.1 equiv) and AcOCHO (0.438 mL, 4.98 mmol, 2.2 equiv), freshly prepared from heating Ac_2O (2.68 mL) and HCO_2H (1.6 mL) at reflux for 4 h, were added sequentially with stirring to the ribofuranosyl azide **32** (1.00 g, 2.49 mmol, 1 equiv) in PhMe (50 mL). After 3 h at 40 °C, the mixture was concentrated *in vacuo* and chromatographed (Et_2O : pentane 1 : 4) to give ribofuranosyl formamide **33** (825 mg, 2.04 mmol, 82%) as a colorless oil. NEt_3 (2.8 mL, 19.9 mmol, 8 equiv) and triphosgene (1.48 g, 4.98 mmol, 2 equiv)

were added sequentially with stirring to formamide **33** in CH_2Cl_2 (30 mL). After 1h, the mixture was concentrated *in vacuo* and the residue chromatographed (Et_2O : pentane 1 : 4) to give isocyanide **34** (931 mg, 2.40 mmol, 96%, 1:2 α : β) as mixture of anomers. The α–anomer was obtained as a colorless oil, whereas the β-anomer was obtained as a white solid: R_f α 0.45 (Et_2O : pentane 1 : 4); R_f β 0.27 (Et_2O : pentane 1 : 4); m.p. β (Et_2O/pentane) (126.2 - 127.4 °C); α $[\alpha]_D^{25.1} = +34.0$ (*c* 1.0, $CHCl_3$); β $[\alpha]_D^{25} = -3.1$ (*c* 1.0, $CHCl_3$); ^{1}H-NMR α (400 MHz, $CDCl_3$) δ 8.22-7.95 (m.4H), 7.71 – 7.57 (m, 2H), 7.57 – 7.43 (m, 4H), 5.63 (ddd, *J* = 15.9, 4.5, 0.8 Hz, 1H), 5.52 – 5.41 (m, 1H), 4.88 (q, *J* = 4.1 Hz, 1H), 4.84 – 4.65 (m, 2H); ^{1}H-NMR β (400 MHz, $CDCl_3$) δ 8.13 – 8.01 (m, 4H), 7.67 – 7.53 (m, 2H), 7.52 – 7.37 (m, 4H), 5.96 – 5.80 (m, 1H), 5.38 (dd, *J* = 7.4, 2.5 Hz, 1H), 4.79 – 4.66 (m, 1H), 4.66 – 4.51 (m, 2H); ^{13}C{^{1}H}-NMR α (101 MHz, $CDCl_3$) δ 167.2, 165.9, 164.9, 134.3, 133.5, 130.2, 130.1, 129.8, 128.8, 128.7, 128.5, 128.5, 128.4, 82.6, 82.4, 82.2, 81.9, 79.9, 79.8, 71.4, 71.2, 71.0, 70.8, 62.3, 62.3; ^{13}C{^{1}H}-NMR β (101 MHz, $CDCl_3$) δ 167.8, 166.0, 164.6, 134.3, 133.5, 130.1, 129.9, 129.1, 128.7, 128.5, 127.8, 81.7, 81.5, 81.3, 81.1, 79.6, 79.5, 77.4, 70.4, 70.3, 70.1, 70.0, 62.5; ^{19}F{^{1}H}-NMR α (377 MHz, $CDCl_3$) δ -107.30, -107.96, -120.33, -120.99; ^{19}F{^{1}H}-NMR β (377 MHz, $CDCl_3$) δ -116.17, -116.80, -121.71, -122.34; IR ν_{max} α 2127, 1726, 708 cm^{-1}; IR ν_{max} β 2130, 1729, 707 cm^{-1}; HRMS (APCI) *m/z*: [M - NC]$^+$ Calcd for $(C_{19}H_{15}F_2O_5)^+$: 361.0882; Found 361.0844.

4. Synthesis of 3,5-Di-*O*-benzoyl-2-deoxy-2-fluoro-2-methyl-*D*-ribofuranosyl isonitrile

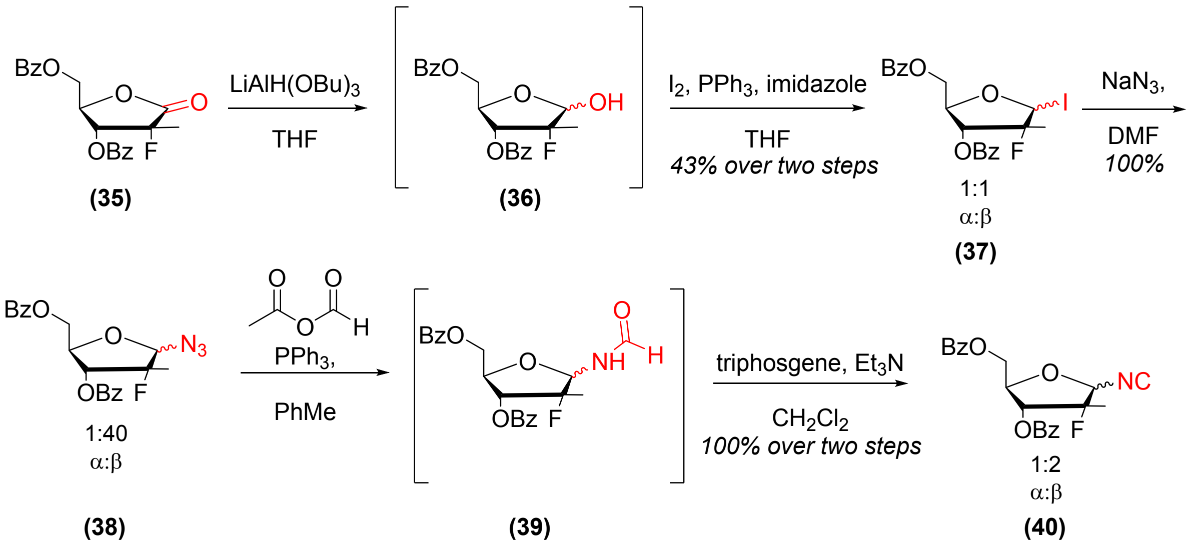

3,5-Di-*O*-benzoyl-2-deoxy-2-fluoro-2-methyl-*D*-ribofuranosyl iodide (37)

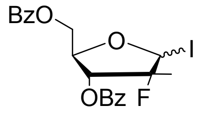

(37)

$LiAlH(O\text{-}t\text{-}Bu)_3$ (946 mg, 3.72 mmol, 2 equiv) was added portion-wise with stirring to lactone **35** (694 mg, 1.86 mmol, 1 equiv) in Et_2O and THF (1:1, 20 mL) at 0 °C. After 1h, reaction was quenched with H_2O (20 mL) and the mixture was extracted with Et_2O (2 x 20 mL). The combined organic extracts were washed with aqueous HCl (1M), saturated aqueous $NaHCO_3$ (10 mL) and saturated aqueous Rochelle salt (10 mL), dried ($MgSO_4$, twice), filtered, and concentrated *in vacuo* as a colorless oil containing the crude alcohol **36**. I_2 (946 mg, 3.72 mmol, 2 equiv), PPh_3 (977 mg, 3.72 mmol, 2 equiv) and imidazole (152 mg, 2.23 mmol, 1.2 equiv) were added sequentially with stirring to this oil in CH_2Cl_2 (20 mL). After stirring overnight, the mixture was concentrated *in vacuo* and the residue chromatographed (EtOAc : pentane 1 : 9) to give iodide **37** (388 mg, 0.802 mmol, 43%, α : β, 1:1) as a colorless oil. Partial epimerization of iodide **37** occurred over time leading to a 1:1 ratio of anomers: R_f 0.85 (EtOAc : pentane 1 : 9); R_f 0.74 (EtOAc : pentane 1 : 9); $[\alpha]_D^{24.1}$= + 43.4 (*c* 1.0, $CHCl_3$); ^{1}H-NMR (400 MHz, $CDCl_3$) δ 8.24 – 8.14 (m, 2H), 8.14 – 8.00 (m, 6H), 7.68 – 7.57 (m, 3H), 7.57 – 7.42 (m, 7H), 7.41 – 7.33 (m, 2H), 6.83 (d, *J* = 14.3 Hz, 1H), 6.73 (dd, *J* = 4.7, 0.8 Hz, 1H), 6.19 (dd, *J* = 22.7, 8.0 Hz, 1H), 5.34 (dd, *J* = 5.6, 4.5 Hz, 1H), 4.88 – 4.72 (m, 4H), 4.72 – 4.57 (m, 2H), 1.88 (d, *J* = 22.2 Hz, 3H), 1.69 (d, *J* = 21.1 Hz, 3H); $^{13}C\{^1H\}$-NMR (101 MHz, $CDCl_3$) δ 166.0, 165.9, 165.7, 165.5, 133.9, 133.7, 133.4, 133.2, 130.1, 130.1, 129.9, 129.7, 129.5, 129.3, 128.8, 128.6, 128.6, 128.5, 128.3, 102.9, 101.1, 95.7, 93.6, 81.9, 80.7, 75.4, 75.2, 73.7, 73.6, 73.5, 73.4, 71.5, 71.3, 62.5, 62.1, 22.2, 21.9, 20.5, 20.3; $^{19}F\{^1H\}$-NMR (377 MHz, $CDCl_3$) δ -138.27, -138.30, -158.06, -158.08; IR v_{max} 1718, 1260, 704 cm^{-1}; HRMS (APCI) *m/z*: $[M – I]^+$ Calcd for $(C_{20}H_{18}FO_5)^+$: 357.1133; Found 357.113. Analytical data were in good agreement with reported values.[6]

3,5-Di-*O*-benzoyl-2-deoxy-2-fluoro-2-methyl-*D*-ribofuranosyl azide[2] (38)

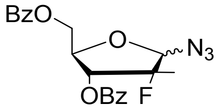

(38)

NaN_3 (35.0 mg, 0.536 mmol, 2 equiv) was added with stirring to ribofuranosyl iodide **37** (130 mg, 0.268 mmol, 1 equiv) in DMF (10 mL). After stirring overnight at 50 °C, reaction was quenched with H_2O (20 mL) and the mixture was extracted with Et_2O (2 x 20 mL). The combined organic extracts were washed with saturated aqueous $NaHCO_3$, dried ($MgSO_4$),

filtered, and concentrated *in vacuo*. Chromatography (Et_2O : pentane 3 : 7) gave azide **38** (107 mg, 0.268 mmol, 100%, 40 : 1 β : α) as a colorless oil: R_f -β 0.81 (Et_2O : pentane 3 : 7); R_f -α 0.62 (Et_2O : pentane 3 : 7): -β $[\boldsymbol{\alpha}]_{\boldsymbol{D}}^{25.6} = -142$ (*c* 1.0, $CHCl_3$); -α $[\boldsymbol{\alpha}]_{\boldsymbol{D}}^{26.1} = +159$ (*c* 1.0, $CHCl_3$); ^{1}H-NMR -β anomer (400 MHz, $CDCl_3$) δ 8.06 (ddt, *J* = 10.9, 7.1, 1.4 Hz, 4H), 7.64 – 7.50 (m, 2H), 7.42 (td, *J* = 7.5, 6.2, 1.7 Hz, 4H), 5.61 (dd, *J* = 23.3, 8.3 Hz, 1H), 5.46 (d, *J* = 12.3 Hz, 1H), 4.72 (dd, *J* = 12.1, 3.7 Hz, 1H), 4.64 (dt, *J* = 8.1, 4.0 Hz, 1H), 4.49 (dd, *J* = 12.1, 4.4 Hz, 1H), 1.51 (d, *J* = 22.5 Hz, 3H); ^{1}H-NMR -α anomer (400 MHz, $CDCl_3$) δ 8.16 – 7.95 (m, 4H), 7.68 – 7.53 (m, 2H), 7.53 – 7.38 (m, 4H), 5.45 (dd, *J* = 16.0, 7.7 Hz, 1H), 5.02 (d, *J* = 12.0 Hz, 1H), 4.79 (dddd, *J* = 7.9, 4.5, 3.6, 0.9 Hz, 1H), 4.72 (dd, *J* = 12.2, 3.6 Hz, 1H), 4.53 (dd, *J* = 12.2, 4.5 Hz, 1H), 1.65 (s, 2H), 1.59 (s, 2H); $^{13}C\{^1H\}$-NMR -β anomer (101 MHz, $CDCl_3$) δ 166.1, 165.6, 133.8, 133.2, 130.0, 129.6, 129.6, 128.7, 128.6, 128.4, 100.8, 98.9, 94.7, 94.4, 78.5, 73.8, 73.7, 63.6, 16.8, 16.6; $^{13}C\{^1H\}$-NMR α-anomer (101 MHz, $CDCl_3$) δ 166.0, 165.6, 133.8, 133.3, 130.1, 129.7, 129.4, 128.6, 128.5, 98.8, 96.8, 93.1, 92.9, 78.8, 77.3, 77.2, 77.0, 76.7, 74.2, 74.0, 63.3, 19.6, 19.4; $^{19}F\{^1H\}$-NMR β-anomer (377 MHz, $CDCl_3$) δ -165.93, -165.98; $^{19}F\{^1H\}$-NMR α-anomer (377 MHz, $CDCl_3$) δ -172.74, -172.78; IR v_{max} β-anomer 2113, 1722, 1244, 704 cm^{-1}; IR v_{max} α-anomer 2114, 1723, 1264, 708 cm^{-1}; HRMS (ESI) *m/z*: $[M + H]^+$ Calcd for $(C_{20}H_{19}FN_3O_5)^+$: 400.1303; Found 400.1302. Analytical data for the β- anomer (^{1}H, ^{13} NMR) were in good agreement with reported values.[7]

3,5-Di-*O*-benzoyl-2-deoxy-2-fluoro-2-methyl-*D*-ribofuranosyl isonitrile (40)

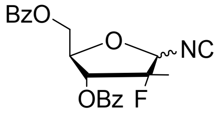

(40)

PPh_3 (563 mg, 6.40 mmol, 2 equiv) and AcOCHO (0.839 mL, 3.52 mmol, 1.1 equiv), freshly prepared from heating Ac_2O (2.68 mL) and HCO_2H (1.6 mL) at reflux for 4 h, were added sequentially with stirring to ribofuranosyl azide **38** (1.27 g, 3.20 mmol, 1 equiv) in PhMe (40 mL). After stirring at 40 °C for 3h, the mixture was concentrated *in vacuo* and the residue chromatographed (EtOAc : pentane 1 : 1) to give ribofuranosyl formamide **39** (984 mg, 2.45 mmol, 77%) as a colorless oil. NEt_3 (2.76 mL, 19.6 mmol, 8 equiv) and triphosgene (1.45 g, 4.90 mmol, 2 equiv) were added sequentially with stirring to formamide **39** (984 mg, 2.45 mmol, 1 equiv) in CH_2Cl_2 (30 mL). After 2h, the mixture was concentrated *in vacuo* and the residue chromatographed (Et_2O : pentane 1 : 4) to give ribofuranosyl isocyanide **40** (940 mg, 2.45 mmol, 100%, β : α 2:1) as a colorless oil. Anomeric stereochemistry was tentatively assigned: R_f β-anomer 0.65 (Et_2O : pentane 3 : 7); R_f α-anomer 0.45 (Et_2O : pentane 3:7); β-

anomer $[\alpha]_D^{25.9}$ = + 47.2 (*c* 1.0, $CHCl_3$); α-anomer $[\alpha]_D^{25.7}$ = + 45.5 (*c* 1.0, $CHCl_3$); ^{1}H-NMR α-anomer (400 MHz, $CDCl_3$) δ 8.17 – 8.04 (m, 2H), 8.04 – 7.96 (m, 2H), 7.64 – 7.53 (m, 2H), 7.52 – 7.38 (m, 4H), 5.32 (td, *J* = 5.7, 0.5 Hz, 1H), 5.19 (dt, *J* = 3.0, 0.4 Hz, 1H), 4.83 (dddt, *J* = 5.8, 4.5, 3.4, 0.5 Hz, 1H), 4.75 (ddd, *J* = 12.5, 3.4, 0.5 Hz, 1H), 4.57 (dd, *J* = 12.5, 4.6 Hz, 1H), 1.70 (d, *J* = 21.9 Hz, 3H); ^{1}H-NMR β-anomer (400 MHz, $CDCl_3$) δ 8.12 – 8.03 (m, 4H), 7.66 – 7.59 (m, 1H), 7.58 – 7.51 (m, 1H), 7.51 – 7.43 (m, 2H), 7.43 – 7.36 (m, 2H), 5.75 (dd, *J* = 22.4, 8.6 Hz, 1H), 5.31 (d, *J* = 11.4 Hz, 1H), 4.71 (dd, *J* = 12.3, 3.6 Hz, 1H), 4.68 – 4.58 (m, 1H), 4.53 (dd, *J* = 12.3, 4.2 Hz, 1H), 1.73 (d, *J* = 22.1 Hz, 3H); ^{13}C{^{1}H}-NMR α-anomer (101 MHz, $CDCl_3$) δ 165.9, 165.6, 164.9, 133.9, 133.5, 130.1, 129.7, 129.2, 128.6, 128.5, 128.4, 95.1, 93.4, 85.8, 85.7, 80.9, 73.5, 73.4, 62.6, 21.7, 21.5; ^{13}C{^{1}H}-NMR β-anomer (101 MHz, $CDCl_3$) δ 166.1, 165.3, 165.3, 134.0, 133.3, 130.1, 129.8, 129.7, 129.3, 128.6, 128.5, 128.4, 128.3, 100.6, 99.1, 86.6, 86.3, 79.2, 73.0, 72.9, 62.7, 17.3, 17.1; ^{19}F{^{1}H}-NMR β-anomer (377 MHz, $CDCl_3$) δ -168.02; ^{19}F{^{1}H}-NMR α-anomer (377 MHz, $CDCl_3$) δ -165.50; IR ν_{max} α-anomer 2128, 1723, 1264, 708 cm^{-1}; IR ν_{max} β-anomer 2126, 1723, 1265, 708 cm^{-1}; HRMS (ESI) *m/z*: $[M + H]^+$ Calcd for $(C_{21}H_{19}FNO_5)^+$: 384.1242; Found 384.1236.

5. Synthesis of 3,5-di-*O*-benzoyl-2-deoxy-2-fluoro-β-*D*-arabinofuranosyl isonitrile

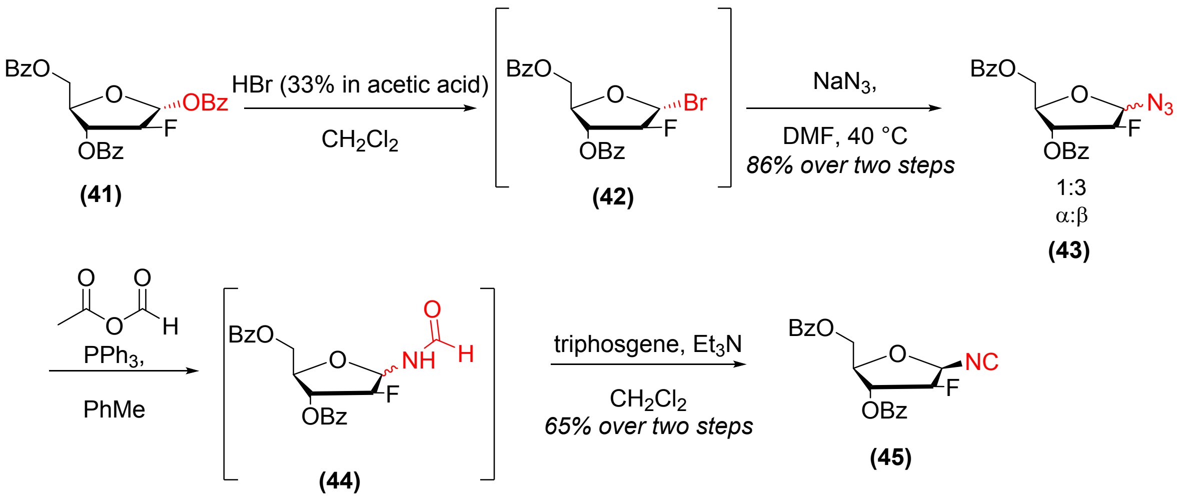

3,5-Di-*O*-benzoyl-2-deoxy-2-fluoro-*D*-arabinofuranosyl azide (43).[8,9]

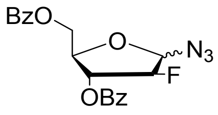

(43)

HBr (33% in AcOH, 10 mL) was added to benzoate **41** (337 mg, 0.725 mmol, 1 equiv) in CH_2Cl_2 (20 mL) at 0 °C and the reaction mixture was allowed to warm up to 25 °C. After 1h, reaction was quenched with H_2O (40 mL) and the mixture was extracted with CH_2Cl_2 (2 x 50

mL). The combined organic extracts were washed with saturated aqueous $NaHCO_3$, dried ($MgSO_4$), filtered, and concentrated *in vacuo.* NaN_3 (125 mg, 1.930 mmol, 2.6 equiv) was added to the crude bromide **42** in DMF (15 mL). After 3h, reaction was quenched with H_2O, LiCl (0.5 g) was added to the mixture to facilitate phase separation, and the mixture was extracted with Et_2O (2 x 50 mL). The combined organic extracts were washed with saturated aqueous $NaHCO_3$, dried ($MgSO_4$), filtered, and concentrated *in vacuo*. Chromatography (Et_2O : pentane 1 : 4) gave α-azide **43** (58.0 mg, 0.150 mmol, 21%) as a colorless oil and β-azide **43** (0.181 g, 0.469 mmol, 65%) as a white solid: R_f α-anomer 0.47 (Et_2O : pentane 1 : 4); R_f β-anomer 0.23 (Et_2O : pentane 1 : 4); m.p. (Et_2O/pentane) for β-anomer (85.8 - 86.8 °C)(lit.[8] 89.0 - 90.5 °C); α-anomer $[\alpha]_D^{25.3}$ + 174 (*c* 1.0, $CHCl_3$); β-anomer $[\alpha]_D^{25.4}$ – 54.5 (*c* 1.0, $CHCl_3$); ^{1}H-NMR α-anomer (400 MHz, $CDCl_3$) δ 8.13 – 8.00 (m, 4H), 7.66 – 7.53 (m, 2H), 7.52 – 7.39 (m, 4H), 5.76 (dd, *J* = 12.6, 0.9 Hz, 1H), 5.54 (ddt, *J* = 20.5, 3.7, 0.9 Hz, 1H), 5.05 (dd, *J* = 48.9, 0.7 Hz, 1H), 4.76 (dd, *J* = 11.4, 3.5 Hz, 1H), 4.74 – 4.62 (m, 2H); ^{1}H-NMR β-anomer (400 MHz,) δ 8.13 – 7.98 (m, 4H), 7.65 – 7.51 (m, 2H), 7.51 – 7.39 (m, 4H), 5.74 – 5.63 (m, 1H), 5.37 – 5.14 (m, 2H), 4.76 – 4.61 (m, 2H), 4.44 (dt, *J* = 5.5, 4.3 Hz, 1H); ^{13}C{^{1}H}-NMR α-anomer (101 MHz, $CDCl_3$) δ 166.2, 165.5, 133.9, 133.2, 129.9, 129.8, 129.5, 128.7, 128.6, 128.4, 98.7, 96.8, 94.1, 93.7, 83.4, 63.4; ^{13}C{^{1}H}-NMR β-anomer (101 MHz, $CDCl_3$) δ 166.2, 165.2, 133.9, 133.2, 129.9, 129.8, 129.6, 128.7, 128.5, 128.4, 94.9, 92.9, 89.5, 89.3, 80.3, 80.2, 76.3, 76.0, 64.0; ^{19}F{^{1}H}-NMR α-anomer (377 MHz, $CDCl_3$) δ -187.07; ^{19}F{^{1}H}-NMR β-anomer (377 MHz, $CDCl_3$) δ -201.98; IR v_{max} α-anomer 2109, 1718, 1258, 704 cm^{-1}; IR v_{max} β-anomer 2115, 1717, 1258, 704 cm^{-1}; HRMS (ESI) *m/z*: $[M + H]^+$ Calcd for $(C_{19}H_{17}FN_3O_5)^+$: 386.1147; Found 386.1136. Analytical data for the β- anomer (^{1}H NMR, ^{13}C NMR, ^{19}F NMR) were in good agreement with reported values.[8,9] Analytical data for the α- anomer (^{1}H NMR, ^{13}C NMR, ^{19}F NMR) were in good agreement with reported values.[9]

3,5-Di-*O*-benzoyl-2-deoxy-2-fluoro-β-*D*-arabinofuranosyl isonitrile (45).

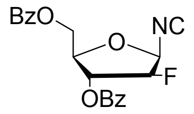

(45)

PPh_3 (97.0 mg, 0.372 mmol, 1 equiv) and AcOCHO (0.064 mL, 0.744 mmol, 2 equiv), freshly prepared from heating Ac_2O (2.68 mL) and HCO_2H (1.6 mL) at reflux for 4 h, were added sequentially with stirring to azide **43** (143 mg, 0.372 mmol, 1 equiv) in PhMe (10 mL). After 1h at 40 °C, the mixture was concentrated *in vacuo* and chromatography of the residue (EtOAc : pentane 1 : 1) gave formamide **44** (418 mg, 0.86 mmol, 83%) as a white foam. Et_3N (0.418 mL, 2.97 mmol, 8 equiv) and triphosgene (220 mg, 0.744 mmol, 2 equiv) were added

sequentially with stirring to the crude formamide **44** in CH_2Cl_2 (10 mL). After 20 min, the mixture was concentrated *in vacuo* and chromatography of the residue (Et_2O : pentane 2 : 3) gave mainly the β–isocyanide **45** (0.088 g, 0.240 mmol, 65%) as a white solid and only analytical traces of the α–isocyanide **45** : R_f 0.41 (Et_2O : pentane 2 : 3); m.p. β–anomer (Et_2O/pentane) (106.2 - 107.4 °C); β–anomer $[\boldsymbol{\alpha}]_{\boldsymbol{D}}^{25.1}$- 15.4 (*c* 1.0, $CHCl_3$); ^{1}H-NMR β–anomer (400 MHz, $CDCl_3$) δ 8.17 – 7.95 (m, 4H), 7.68 – 7.51 (m, 2H), 7.46 (dtd, *J* = 14.1, 7.5, 7.1, 1.7 Hz, 4H), 5.79 (dt, *J* = 15.6, 4.1 Hz, 1H), 5.54 (dd, *J* = 8.3, 4.5 Hz, 1H), 5.30 (dt, *J* = 50.9, 4.2 Hz, 1H), 4.70 (qdd, *J* = 12.1, 5.2, 0.8 Hz, 2H), 4.50 (dt, *J* = 5.7, 4.5 Hz, 1H); ^{1}H-NMR α-anomer (400 MHz, $CDCl_3$) δ 8.16 – 8.00 (m, 4H), 7.68 – 7.52 (m, 2H), 7.54 – 7.36 (m, 4H), 5.67 – 5.52 (m, 2H), 5.38 (d, *J* = 48.2 Hz, 1H), 4.77 (dt, *J* = 10.1, 3.5 Hz, 2H), 4.66 (dd, *J* = 12.8, 5.5 Hz, 1H); ^{13}C{^{1}H}-NMR β–anomer (101 MHz, $CDCl_3$) δ 166.1, 165.1, 134.1, 133.3, 129.9, 129.4, 128.7, 128.4, 128.2, 92.7, 90.7, 81.3, 81.3, 75.7, 75.4, 63.4; ^{13}C{^{1}H}-NMR α-anomer (101 MHz, $CDCl_3$) δ 166.1, 165.3, 164.7, 134.1, 133.3, 130.0, 129.8, 129.4, 128.8, 128.5, 128.2, 98.7, 96.9, 84.2, 76.3, 76.0, 62.9; ^{19}F{^{1}H}-NMR β–anomer (377 MHz, $CDCl_3$) δ -197.82; ^{19}F{^{1}H}-NMR α-anomer (377 MHz, $CDCl_3$) δ -184.00; IR ν_{max} 2133, 1727, 1267, 710 cm^{-1}; HRMS (ESI) *m/z*: [M - NC]$^+$ Calcd for $(C_{20}H_{16}FO_5)^+$: 343.0976; Found 343.0997.

III. Synthesis of 5-(Aminomethylene)-1,3-dioxane-4,6-dione derivatives

1. Synthesis of 5-((Cyclohexylamino)methylene)-2,2-dimethyl-1,3-dioxane-4,6-dione

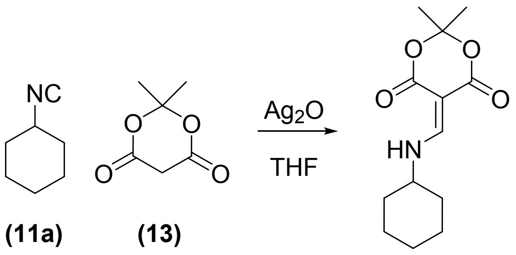

(11a) **(13)** **(15a)**

5-((Cyclohexylamino)methylene)-2,2-dimethyl-1,3-dioxane-4,6-dione (15a).

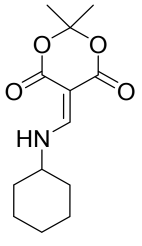

(15a)

2,2-Dimethyl-1,3-dioxane-4,6-dione (Meldrum's acid) (**13**) (5.23 g, 36.3 mmol, 1.1 equiv) and Ag_2O (7.65 g, 33.0 mmol, 1 equiv) were added sequentially with stirring to cyclohexyl isonitrile

(**11a**) (4.51 mL, 33.0 mmol, 1 equiv) in THF (50 mL). The reaction mixture was left stirring overnight at 70 °C, cooled to room temperature, filtered to remove the silver salts and concentrated *in vacuo*. Chromatography (pentane : CH_2Cl_2: Me_2CO, 20 : 19 : 1) gave enamide **15a** (1.21 g, 4.81 mmol, 15%) as a white solid: R_f 0.74 (pentane : EtOAc, 3:2); m.p. (CH_2Cl_2/pentane) (150.5 – 153.0 °C)(lit.[1] 152-153 °C); IR ν_{max} 3243, 3205, 1723, 1674, 1621, 1448 cm^{-1}; ^{1}H-NMR (400 MHz, $CDCl_3$) δ 9.56 (s, br, 1H), 8.14 (dd, *J* = 4 Hz, 1H), 3.38-3.31 (m, 1H), 2.02-1.98 (m, 2H), 1.85-1.80 (m, 2H), 1.70 (m, 6H), 1.49-1.41 (m, 2H), 1.41-1.33 (m, 2H), 1.29-1.19 (m, 2H); ^{13}C{^{1}H}-NMR (101 MHz, $CDCl_3$) δ 165.7, 164.1, 157.5, 104.6, 84.1, 59.0, 33.4, 26.8, 24.8, 24.3; HRMS (ESI) *m/z*: $[M + H]^+$ Calcd for $(C_{13}H_{20}NO_4)^+$: 254.1387; Found 254.1400. Spectroscopic data (^{1}H, ^{13}C NMR) were in good agreement with reported values.[11,12]

2. Synthesis 5-(5-*O*-acetyl-2,3-di-*O*-iso-propylidene-β-*D*-ribofuranosylamino-methylene)-2,2-dimethyl-1,3-dioxa-4,6-dione

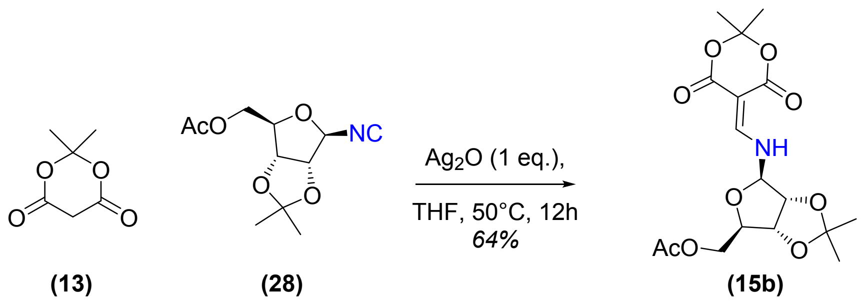

5-(5-*O*-Acetyl-2,3-di-*O*-iso-propylidene-β-*D*-ribofuranosylamino-methylene)-2,2-dimethyl-1,3-dioxa-4,6-dione (15b)

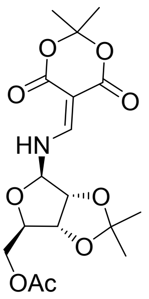

(15b)

2,2-Dimethyl-1,3-dioxane-4,6-dione (Meldrum's acid) (**13**) (91.0 mg, 0.633 mmol, 1.2 equiv) and Ag_2O (122 mg, 0.527 mmol, 1 equiv) were added sequentially with stirring to ribofuranosyl isonitrile **28** (127 mg, 0.527 mmol, 1 equiv) in THF (20 mL). After stirring overnight at 50 °C, the mixture was filtered to remove the silver salt and concentrated *in vacuo*. Chromatography

(pentane : EtOAc, 7 : 3) gave enamide **15b** (130 mg, 0.337 mmol, 64%) as a white solid: R_f 0.61 (pentane : EtOAc, 1 : 1); $[\alpha]_D^{26.2} = -28.8$ (*c* 1.0, $CHCl_3$); m.p. (Et_2O) (146.0 - 148.7 °C); ^{1}H-NMR (400 MHz, $CDCl_3$) δ 9.79 (t, *J* = 12.0 Hz, 1H), 8.21 (d, *J* = 14.2 Hz, 1H), 5.22 (d, *J* = 9.4 Hz, 1H), 4.73 (d, *J* = 0.9 Hz, 2H), 4.55 (t, *J* = 2.7 Hz, 1H), 4.38 (dd, *J* = 12.4, 2.9 Hz, 1H), 4.10 (dd, *J* = 12.4, 2.4 Hz, 1H), 2.26 (s, 3H), 1.70 (d, *J* = 1.0 Hz, 6H), 1.55 (s, 3H), 1.38 – 1.31 (m, 3H); $^{13}C\{^1H\}$-NMR (101 MHz, $CDCl_3$) δ 170.5, 165.1, 163.4, 158.1, 114.1, 105.0, 97.6, 86.4, 86.2, 84.1, 82.0, 65.2, 27.1, 26.9, 26.8, 25.1, 20.9; IR ν_{max} 1735, 1679, 1604, 1265 cm^{-1}; HRMS (ESI) *m/z*: $[M - H]^-$ Calcd for $(C_{17}H_{22}NO_9)^-$: 384.1300; Found 384.1300.

3. Synthesis of 5-(3,5-di-*O*-benzoyl-2-deoxy-2-fluoro-2-methyl-*D*-ribofuranosylamino-methylene)-2,2-dimethyl-1,3-dioxa-4,6-dione

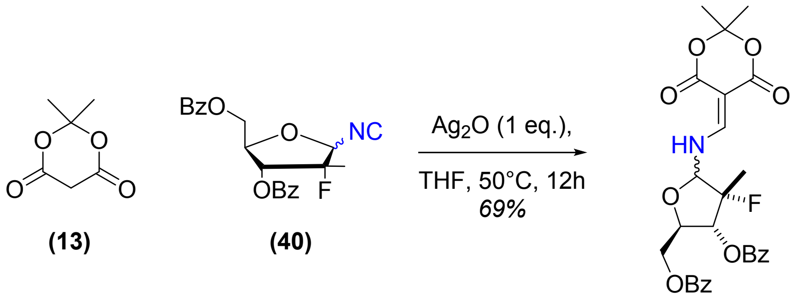

(15c)

5-(3,5-Di-*O*-benzoyl-2-deoxy-2-fluoro-2-methyl-*D*-ribofuranosylamino-methylene)-2,2-dimethyl-1,3-dioxa-4,6-dione (15c)

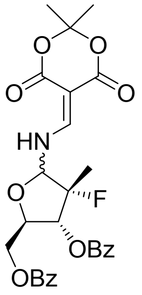

(15c)

2,2-Dimethyl-1,3-dioxane-4,6-dione (Meldrum's acid) (**13**) (388 mg, 2.69 mmol, 1.05 equiv) and Ag_2O (594 mg, 2.56 mmol, 1 equiv) were added sequentially with stirring to isonitrile **40** (984 mg, 2.56 mmol, 1 equiv) in THF (40 mL). After stirring overnight at 45 °C and cooling to room temperature, the mixture was filtered to remove the silver salts, and concentrated *in vacuo*. Chromatography (EtOAc : pentane 1 : 4) gave enamide **15c** (956 mg, 1.77 mmol, 69%, α:β; 1:2.3) as a white solid. Anomeric stereochemistry was tentatively assigned: R_f β-anomer 0.31 (EtOAc : pentane 1 : 4); R_f α-anomer 0.20 (EtOAc : pentane 1 : 4); β-anomer $[\alpha]_D^{25.2}$ –32.7 (*c* 1.0, $CHCl_3$); α-anomer $[\alpha]_D^{24.9}$ +28.7 (*c* 1.0, $CHCl_3$); m.p. (Et_2O) for α (68.5 - 75.3 °C);

m.p. (Et_2O) for β (94.9 - 97.9 °C); ^{1}H-NMR α-anomer (400 MHz, $CDCl_3$) δ 10.03 – 9.82 (m, 1H), 8.28 (dd, *J* = 14.0, 1.4 Hz, 1H), 8.03 (dd, *J* = 8.2, 1.4 Hz, 4H), 7.65 – 7.49 (m, 2H), 7.51 – 7.33 (m, 4H), 5.57 (dd, *J* = 18.9, 8.1 Hz, 1H), 5.34 – 5.19 (m, 1H), 4.72 (dddd, *J* = 8.2, 4.6, 3.5, 1.0 Hz, 1H), 4.57 (ddd, *J* = 70.8, 12.2, 4.1 Hz, 2H), 1.70 (d, *J* = 2.2 Hz, 6H), 1.63 (s, 3H); ^{1}H-NMR β-anomer (400 MHz, $CDCl_3$) δ 9.78 (dd, *J* = 13.7, 8.7 Hz, 1H), 8.27 (d, *J* = 13.6 Hz, 1H), 8.12 – 7.96 (m, 4H), 7.70 – 7.50 (m, 2H), 7.53 – 7.37 (m, 4H), 5.44 (dd, *J* = 17.2, 8.1 Hz, 1H), 5.28 (dd, *J* = 15.5, 8.7 Hz, 1H), 4.67 (dd, *J* = 12.2, 3.2 Hz, 1H), 4.59 (dt, *J* = 7.5, 3.6 Hz, 1H), 4.52 (dd, *J* = 12.2, 4.4 Hz, 1H), 1.70 (d, *J* = 10.7 Hz, 6H), 1.65-1.59 (d, *J* = 13.6 Hz, 3H); ^{13}C{^{1}H}-NMR α-anomer (101 MHz, $CDCl_3$) δ 165.9, 165.5, 164.9, 163.4, 158.9, 134.0, 133.4, 130.1, 129.7, 129.3, 128.7, 128.5, 128.3, 105.1, 97.8, 95.9, 92.2, 92.1, 87.9, 78.1, 77.3, 74.2, 74.1, 63.3, 27.2, 27.0, 18.8, 18.6; ^{13}C{^{1}H}-NMR β-anomer (101 MHz, $CDCl_3$) δ 166.1, 165.4, 165.3, 162.9, 157.8, 133.9, 133.3, 130.1, 129.8, 129.2, 129.0, 128.6, 128.5, 128.4, 128.2, 105.3, 99.2, 97.3, 93.5, 93.2, 87.7, 77.6, 77.2, 73.4, 73.3, 62.7, 27.2, 27.1, 18.2, 17.9; ^{19}F{^{1}H}-NMR α-anomer (377 MHz, $CDCl_3$) δ -173.90; ^{19}F{^{1}H}-NMR β-anomer (377 MHz, $CDCl_3$) δ -161.53; IR ν_{max} β-anomer 1723, 1677, 1610, 1264, 710 cm^{-1}; IR ν_{max} α-anomer 1723, 1679, 1603, 1261, 707 cm^{-1}; HRMS (APCI) *m/z*: $[M - H]^-$ Calcd for $(C_{27}H_{25}FNO_9)^-$: 526.1519; Found 526.1524.

4. Synthesis of 5-(3,5-di-*O*-benzoyl-2-deoxy-2,2-difluoro-*D*-ribofuranosylamino-methylene)-2,2-dimethyl-1,3-dioxa-4,6-dione

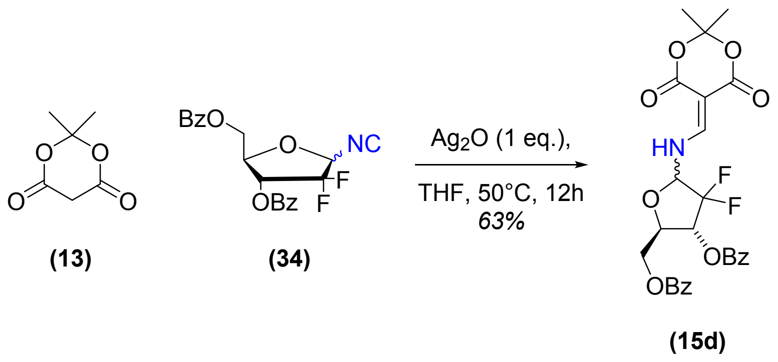

5-(3,5-Di-*O*-benzoyl-2-deoxy-2,2-difluoro-*D*-ribofuranosylamino-methylene)-2,2-dimethyl-1,3-dioxa-4,6-dione (15d)

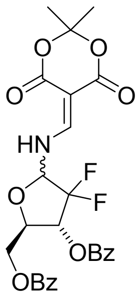

(15d)

2,2-Dimethyl-1,3-dioxane-4,6-dione (Meldrum's acid) (**13**) (16.0 mg, 0.112 mmol, 1.2 equiv) and Ag_2O (21.0 mg, 0.093 mmol, 1 equiv) were added sequentially with stirring to isonitrile **34** (36.0 mg, 0.093 mmol, 1 equiv) in THF (10 mL). The reaction mixture was left stirring overnight at 45 °C, allowed to cool to room temperature, filtered to remove the silver salts, and concentrated *in vacuo*. Chromatography (EtOAc : pentane 1:4) gave enamide **15d** (31.0 mg, 0.058 mmol, 63%) as a white solid: R_f β 0.40 (EtOAc : pentane 3 : 7); m.p. β (Et_2O /pentane) (174.0 - 175.0 °C); m.p. α (Et_2O/pentane) (93.05-94.57 °C); β $[\alpha]_D^{22.3} = -15.4\,°$ (*c* 1.0, $CHCl_3$); α $[\alpha]_D^{22.3} = +35.1$ (*c* 0.3, $CHCl_3$); ^1H-NMR β (400 MHz, $CDCl_3$) δ 9.83 (s, 1H), 8.27 (s, 1H), 8.15 – 7.99 (m, 4H), 7.68 – 7.53 (m, 2H), 7.53 – 7.40 (m, 4H), 5.59 (ddd, *J* = 14.2, 5.0, 1.9 Hz, 1H), 5.33 – 5.23 (m, 1H), 4.71 – 4.58 (m, 2H), 4.52 (qd, *J* = 4.5, 1.2 Hz, 1H), 1.73 (d, *J* = 2.7 Hz, 6H); ^1H-NMR α (400 MHz, $CDCl_3$) δ 10.07 (dd, *J* = 13.5, 8.2 Hz, 1H), 8.30 (d, *J* = 13.5 Hz, 1H), 8.23 – 8.03 (m, 4H), 7.72 – 7.57 (m, 2H), 7.51 (dt, *J* = 19.8, 7.8 Hz, 4H), 5.75 (dt, *J* = 12.6, 3.2 Hz, 1H), 5.48 (t, *J* = 8.0 Hz, 1H), 4.76 (t, *J* = 4.2 Hz, 1H), 4.68 (qd, *J* = 12.3, 4.5 Hz, 2H), 1.75 (d, *J* = 4.3 Hz, 6H); $^{13}C\{^1H\}$-NMR β (101 MHz, $CDCl_3$) δ 166.0, 164.9, 164.6, 162.9, 158.3, 134.3, 133.5, 130.07, 129.8, 129.1, 128.8, 128.6, 127.9, 105.4, 89.2, 88.7, 88.5, 88.3, 88.1, 78.2, 71.9, 71.7, 71.5, 71.4, 62.7, 27.2, 27.2; $^{13}C\{^1H\}$-NMR α (101 MHz, $CDCl_3$) δ 166.0, 165.1, 164.7, 163.0, 158.0, 134.4, 133.5, 130.3, 129.8, 129.1, 129.1, 128.8, 128.6, 127.6, 105.4, 89.0, 88.8, 88.7, 88.6, 88.4, 80.9, 72.0, 71.8, 71.7, 71.5, 62.9, 27.2; $^{19}F\{^1H\}$-NMR β (377 MHz, $CDCl_3$) δ -116.31, -116.97, -121.49, -122.15; $^{19}F\{^1H\}$-NMR α (377 MHz, $CDCl_3$) δ -109.60, -110.27, -123.26, -123.93; IR v_{max} (neat) β 1733, 1686, 1614, 1269 cm^{-1}; IR v_{max} (neat) α 1730, 1685, 1612, 1269 cm^{-1}; HRMS (APCI) *m/z*: $[M - H]^-$ Calcd for $(C_{26}H_{22}F_2NO_9)^-$: 530.1268; Found 530.1254; Anal. Calcd for $C_{26}H_{23}F_2NO_9$: C, 58.76; H, 4.36; N, 2.64; Found: C, 58.58; H, 4.32; N, 2.60.

5. Synthesis of 5-(2,3,4,6-tetra-*O*-acetyl-α-*D*-mannopyranosylamino-methylene)-2,2-dimethyl-1,3-dioxa-4,6-dione

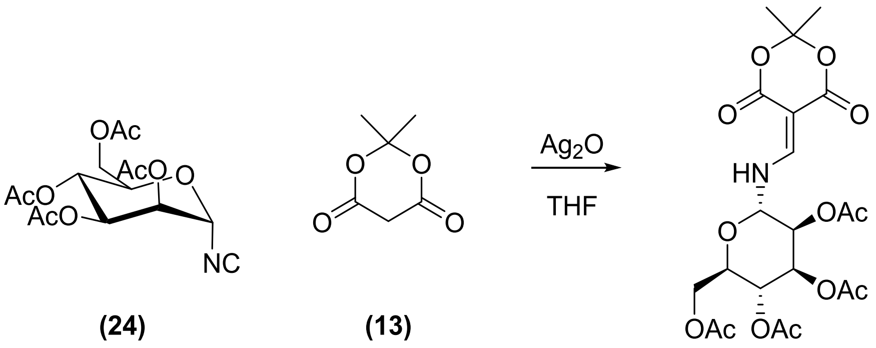

(24) **(13)** **(15f)**

5-(2,3,4,6-Tetra-*O*-acetyl-α-*D*-mannopyranosylamino-methylene)-2,2-dimethyl-1,3-dioxa-4,6-dione (15f).

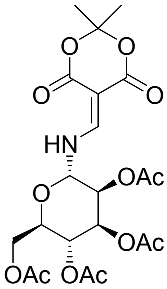

(15f)

2,2-Dimethyl-1,3-dioxane-4,6-dione (Meldrum's acid) (**13**) (78.6 mg, 0.545 mmol, 1 equiv) and Ag_2O (126 mg, 0.545 mmol, 1 equiv) were added sequentially with stirring to isonitrile **24** (195 mg, 0.545 mmol, 1 equiv) in THF (27 mL). The reaction mixture was left stirring overnight at 70 °C. After cooling to room temperature, the mixture was filtered to remove the silver salt and concentrated *in vacuo*. Chromatography (pentane : EtOAc, 1 : 1) gave enamide **15f** (226 mg, 0.405 mmol, 79%) as a white solid: R_f 0.4 (pentane : EtOAc, 1:1); m.p. (CH_2Cl_2/pentane) (68.3 - 77.0 °C) (single point at 69.3 °C); $[\alpha]_D^{24.7}$+ 29.7 (*c* 1.0, $CHCl_3$); ^{1}H-NMR (400 MHz, $CDCl_3$) δ 9.80 (dd, *J* = 13.8, 7.1 Hz, 1H), 8.28 (d, *J* = 13.7 Hz, 1H), 5.37 – 5.25 (m, 2H), 5.22 (dd, *J* = 7.0, 5.5 Hz, 1H), 5.11 (t, *J* = 5.9 Hz, 1H), 4.70 – 4.59 (m, 1H), 4.16 – 4.00 (m, 2H), 2.18 – 2.09 (m, 12H), 1.72 (d, *J* = 1.7 Hz, 6H). ^{13}C{^{1}H}-NMR (101 MHz, $CDCl_3$) δ 170.8, 169.5, 169.4, 169.1, 165.2, 163.1, 158.4, 105.2, 88.1, 81.6, 73.3, 68.0, 67.8, 66.7, 60.7, 60.4, 27.1, 21.1, 20.8, 20.7, 20.6; IR ν_{max} cm^{-1} 1739, 1681, 1608, 1201; HRMS (ESI) *m/z*: [M + Na]$^+$ Calcd for $(C_{21}H_{27}NO_{13}Na)^+$: 524.1300; Found 524.1300. Anal. Calcd for $C_{21}H_{27}NO_{13}$.0.5 H_2O: C, 49.41; H, 5.53; N, 2.74. Found: C, 49.36; H, 5.08; N, 2.88.

6. **Synthesis of 5-(2,3,5-tri-*O*-acetyl-β-*D*-ribofuranosylamino-methylene)-2,2-dimethyl-1,3-dioxa-4,6-dione**

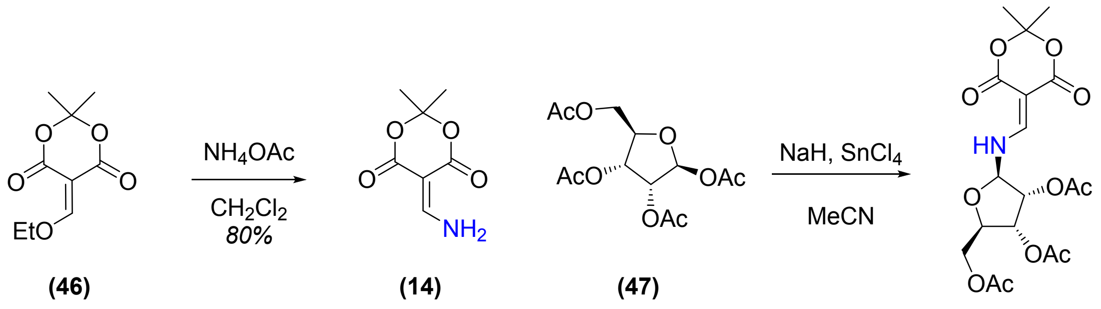

(46) **(14)** **(47)** **(15h)**

(5-(Ethoxymethylene)-2,2-dimethyl-1,3-dioxane-4,6-dione (46)

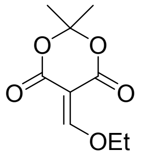

(46)

Meldrum's acid **13** (265 mg, 1.83 mmol, 1 equiv) and $(MeO)_3CH$ (0.612 mL, 3.68 mmol, 2 equiv) were heated at 70 °C for 3 h. The reaction was quenched with cold pentane (20 mL) and left to stir for another 1 h which resulted in precipitation. Filtration and washing with cold pentane gave product **46** (238 mg, 1.18 mmol, 65%) as a yellow solid: R_f 0.63 (MeOH : CH_2Cl_2 1 : 19); m.p. 61.5-62.4 °C (pentane); ^{1}H-NMR (400 MHz, $CDCl_3$) δ 8.23 (s, 1H), 4.51 (q, *J* = 7.2 Hz, 2H), 1.73 (s, 6H), 1.53 (t, *J* = 7.2 Hz, 3H); ^{13}C{^{1}H}-NMR (101 MHz, $CDCl_3$) δ 173.7, 163.5, 158.7, 104.8, 96.7, 76.3, 27.4, 15.4; IR ν_{max} (neat) 2989, 1750, 1714, 1591, 1404, 1259, 1192, 1133 cm^{-1}; HRMS (APCI) *m/z*: $[M - C_2H_5]^-$ Calcd for $(C_7H_7O_5)^-$: 171.0299; Found 171.0289. Analytical data (^{1}H NMR, ^{13}C NMR) were in good agreement with reported values.[10]

5-(Aminomethylene)-2,2-dimethyl-1,3-dioxane-4,6-dione (14)

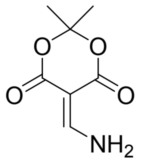

(14)

Ammonium acetate (577 mg, 7.49 mmol, 1.5 equiv) was added with stirring to the Meldrum's acid ethyl enol ether **46** (1.00 g, 4.99 mmol, 1 equiv) in CH_2Cl_2 (20 mL) at room temperature. After 5 h, evaporation and chromatography of the residue (EtOAc : pentane 1 : 1) gave enamide **14** (686 mg, 4.01 mmol, 80%) as a yellow solid: R_f 0.29 (50% EtOAc : pentane 1 : 1); m.p. 204.59-206.54 °C (pentane); ^{1}H-NMR (400 MHz, $CDCl_3$) δ 9.15 (s, 1H), 8.26 (dd, J = 16.0, 8.6 Hz, 1H), 6.56 (s, 1H), 1.72 (s, 6H); ^{13}C{^{1}H}-NMR (101 MHz, $CDCl_3$) δ 165.2, 163.9, 158.9, 104.9, 87.0, 27.0; IR ν_{max} (neat) 3404, 3263, 1722, 1693, 1636 cm^{-1}; HRMS (APCI) *m/z*: $[M + H]^+$ Calcd for $(C_7H_{10}NO_4)^+$: 172.0604; Found 172.0610.

5-(2,3,5-Tri-*O*-acetyl-β-*D*-ribofuranosylamino-methylene)-2,2-dimethyl-1,3-dioxa-4,6-dione (15h)

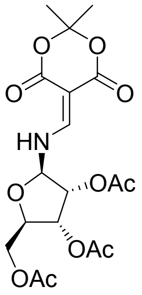

(15h)

Meldrum's acid amine **14** (591 mg, 3.46 mmol, 1.1 equiv), NaH (226 mg, 9.43 mmol, 3 equiv) and $SnCl_4$ in DCM (1M, 0.015 mL, 0.1 equiv) were added to tetraacetate **47** (1.00 g, 3.14 mmol, 1 equiv) in MeCN (20 mL). The mixture was left to stir overnight at room temperature, evaporated and the residue chromatographed (EtOAc : pentane 3 : 7) to give exclusively the *β*-epimer (495 mg, 1.12 mmol, 37%) as a colorless oil: R_f 0.24 (pentane : EtOAc 1 : 1); $[\alpha]_D^{26.3}$+ 121.5 ° (*c* 1.0, $CHCl_3$); ^{1}H-NMR (400 MHz,) δ 10.19 (dd, J = 14.1, 9.4 Hz, 1H), 8.15 (d, J = 14.1 Hz, 1H), 5.48 – 5.44 (m, 1H), 5.44 – 5.40 (m, 1H), 5.31 (t, J = 5.4 Hz, 1H), 4.44 (dd, J = 3.7, 1.8 Hz, 1H), 4.21 (dd, J = 10.5, 3.8 Hz, 2H), 2.25 (s, 3H), 2.12 (s, 3H), 2.10 (s, 3H), 1.73 – 1.70 (m, 6H); ^{13}C{^{1}H}-NMR (101 MHz, $CDCl_3$) δ 170.3, 169.8, 169.3, 165.3, 163.4, 158.9, 105.1, 87.8, 87.1, 80.7, 71.3, 70.1, 63.3, 27.2, 27.0, 20.8, 20.6, 20.3; IR ν_{max} (neat) 1744, 1681, 1605, 1209 cm^{-1}; HRMS (ESI) *m/z*: $[M + H]^+$ Calcd for $(C_{18}H_{24}NO_{11})^+$: 430.1344; Found 430.1360.

7. Synthesis of 5-(3,5-di-*O*-benzoyl-2-deoxy-2-fluoro-β-*D*-arabinofuranosylamino-methylene)-2,2-dimethyl-1,3-dioxa-4,6-dione

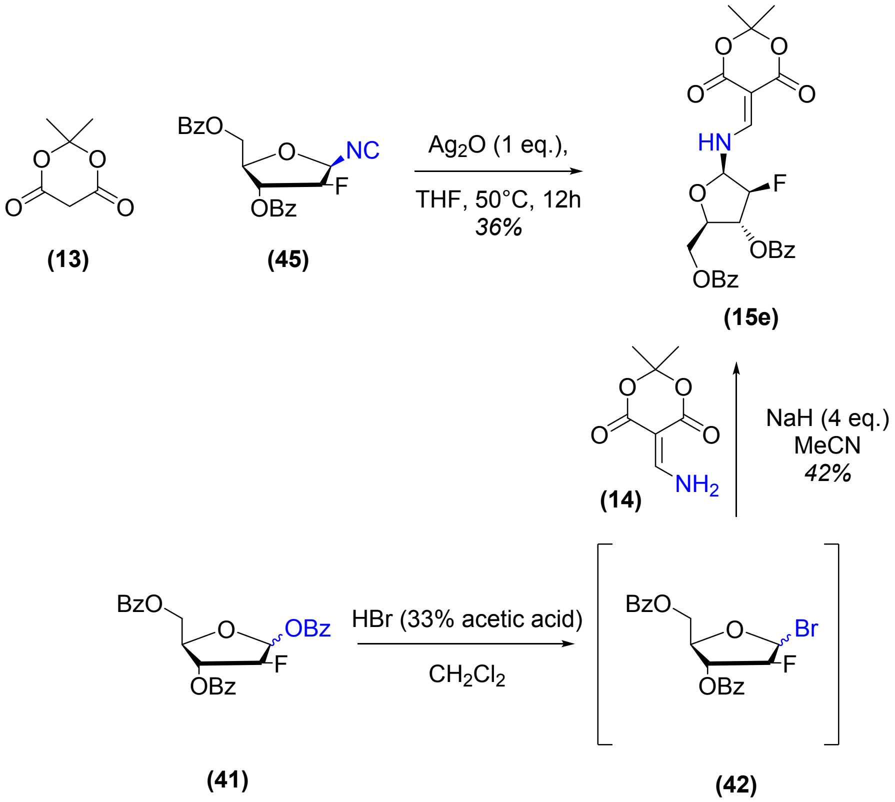

5-(3,5-Di-*O*-benzoyl-2-deoxy-2-fluoro-β-*D*-arabinofuranosylamino-methylene)-2,2-dimethyl-1,3-dioxa-4,6-dione (15e)

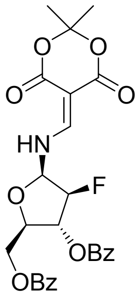

(15e)

<u>**Isocyanide coupling**</u>: 2,2-Dimethyl-1,3-dioxane-4,6-dione (Meldrum's acid) (**13**) (34.0 mg, 0.238 mmol, 1.1 equiv) and Ag_2O (50.0 mg, 0.216 mmol, 1 equiv) were added sequentially with stirring to isonitrile **45** (80.0 mg, 0.216 mmol, 1 equiv) in THF (8 mL). After stirring overnight at 45 °C and allowing the mixture to cool to room temperature, the mixture was filtered to remove the silver salts, and the filtrate was concentrated *in vacuo*. Chromatography (CH_2Cl_2 : pentane : Me_2CO 5 : 5 : 0.2) gave exclusively *β*-enamide **15e** (40.0 mg, 0.078 mmol, 36%) as a white solid.

Anomeric displacement: HBr (33% in AcOH, 2.0 mL) was added to the arabinofuranose derivative (**41**) (500 mg, 1.08 mmol, 1 equiv) in CH_2Cl_2 (20 mL) at 0 °C and the reaction mixture was allowed to warm up to 25 °C. After 1h, reaction was quenched with H_2O (40 mL) and the mixture was extracted with CH_2Cl_2 (2 x 50 mL). The combined organic extracts were washed with saturated aqueous $NaHCO_3$, dried ($MgSO_4$), filtered, and concentrated *in vacuo.* NaH (103 mg, 4.31 mmol, 4 equiv) and Meldrum's acid derivative (**14**) (276 mg, 1.61 mmol, 1.5 equiv) were added to the crude bromide **42** in MeCN (15 mL). Evaporation and chromatography of the residue (EtOAc : pentane 3 : 7) gave exclusively *β*–enamide **15e** (233 mg, 0.453 mmol, 42%) as a white solid: R_f 0.21 (EtOAc : pentane 3 : 7); m.p. (Et_2O) (160.0 - 163.9 °C); $[\alpha]_D^{25.1}$- 16.0 (*c* 1.0, $CHCl_3$); ^{1}H-NMR (400 MHz, $CDCl_3$) δ 9.88 (dd, *J* = 13.8, 9.1 Hz, 1H), 8.31 (dd, *J* = 13.8, 1.5 Hz, 1H), 8.05 (dd, *J* = 7.0, 1.4 Hz, 4H), 7.70 – 7.40 (m, 6H), 5.61 (ddd, *J* = 17.1, 3.0, 1.0 Hz, 1H), 5.48 (ddd, *J* = 18.7, 9.1, 2.9 Hz, 1H), 5.32 – 5.14 (m, 1H), 4.65 (d, *J* = 5.0 Hz, 2H), 4.46 (td, *J* = 5.0, 3.0 Hz, 1H), 1.71 (s, 6H); ^{13}C{^{1}H}-NMR (101 MHz, $CDCl_3$) δ 166.2, 165.0, 164.8, 163.3, 158.6, 134.2, 133.3, 129.9, 129.8, 129.8, 129.4, 128.7, 128.5, 128.5, 128.4, 128.2, 105.2, 94.0, 92.1, 89.2, 89.0, 88.2, 80.7, 63.5, 27.2, 27.1; ^{19}F NMR (377 MHz, $CDCl_3$) δ -201.65, -201.67; IR v_{max} 1725, 1684, 1612, 1269, 710 cm^{-1}; HRMS (ESI) *m/z*: $[M - H]^-$ Calcd for $(C_{26}H_{23}FNO_9)^-$: 512.1362; Found 512.1364.

8. Synthesis of 5-(3,5-di-*O*-(4-methylbenzoyl)-2-deoxy-β-*D*-ribofuranosylamino-methylene)-2,2-dimethyl-1,3-dioxa-4,6-dione

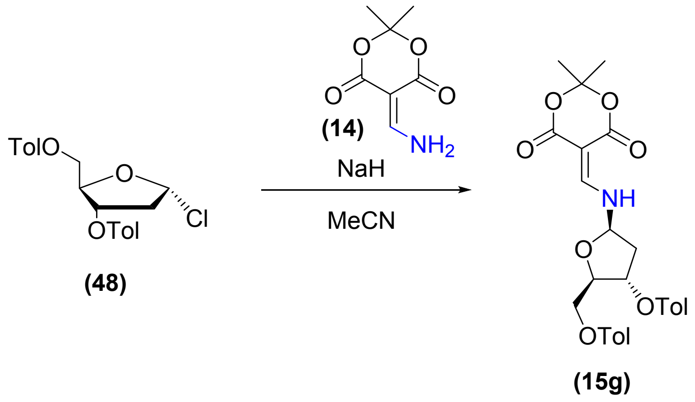

5-(3,5-Di-*O*-(4-methylbenzoyl)-2-deoxy-β-*D*-ribofuranosylamino-methylene)-2,2-dimethyl-1,3-dioxa-4,6-dione (15g)

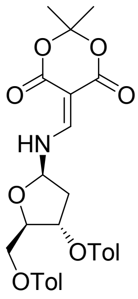

(15g)

Meldrum's acid amine **14** (136 mg, 0.794 mmol, 1.3 equiv) and NaH (43.0 mg, 1.93 mmol, 3 equiv) were added to Hoffer's chlorosugar **48** (237 mg, 0.611 mmol, 1 equiv) in MeCN (8.0 mL). The mixture was left to stir for 2 h at room temperature, evaporated and the residue was chromatographed (EtOAc : pentane 3 : 7) to give β-enamide **15g** (200 mg, 0.382 mmol, 62 %, β ; α 3:1) as a white solid: R_f β 0.43 (EtOAc : pentane 3 : 7): R_f α 0.14 (EtOAc : pentane 3 : 7); m.p. β 160.7-162.0 °C (Et_2O); m.p. α 81.7-82.3 °C (Et_2O); b $[\alpha]_D^{26.2}$ – 58.3 ° (*c* 1.0, $CHCl_3$); α $[\alpha]_D^{26.2}$ 13.1° (*c* 1.0, $CHCl_3$); ^{1}H-NMR β (400 MHz, $CDCl_3$) δ 9.69 (dd, *J* = 14.0, 8.8 Hz, 1H), 8.29 (d, *J* = 14.0 Hz, 1H), 7.93 (dd, *J* = 14.8, 8.0 Hz, 4H), 7.27 (d, *J* = 7.7 Hz, 4H), 5.54 (dd, *J* = 8.3, 5.9 Hz, 2H), 4.66 (dd, *J* = 11.7, 3.7 Hz, 1H), 4.56 – 4.41 (m, 2H), 2.71 – 2.54 (m, 1H), 2.42 (d, *J* = 7.9 Hz, 7H), 1.70 (d, *J* = 3.0 Hz, 6H); ^{1}H-NMR α (400 MHz, $CDCl_3$) δ 10.01 (dd, *J* = 14.3, 8.2 Hz, 1H), 8.31 (d, *J* = 14.2 Hz, 1H), 8.04 (d, *J* = 7.9 Hz, 2H), 7.92 (d, *J* = 8.0 Hz, 3H), 7.26 (dd, *J* = 15.5, 7.8 Hz, 5H), 5.76 – 5.51 (m, 2H), 4.70 (d, *J* = 4.4 Hz, 1H), 4.51 (t, *J* = 3.6 Hz, 2H), 2.79 (dt, *J* = 13.8, 6.6 Hz, 1H), 2.48 (d, *J* = 14.8 Hz, 1H), 2.40 (s, 6H), 1.68 (d, *J* = 6.1 Hz, 6H); ^{13}C{^{1}H}-NMR β (101 MHz, $CDCl_3$) δ 166.2, 165.8, 165.2, 163.4, 158.2, 144.6, 144.2, 129.8, 129.4, 129.3, 126.6, 126.3, 105.0, 90.0, 86.8, 82.9, 75.1, 64.1, 39.0, 27.1, 27.0, 21.8, 21.7; ^{13}C{^{1}H}-NMR α (101 MHz, $CDCl_3$) δ 166.1, 166.1, 165.0, 163.6, 158.0, 144.6, 144.1, 130.1, 129.7, 129.7, 129.4, 129.3, 126.7, 125.9, 104.8, 90.2, 86.0, 84.1, 75.1, 64.0, 38.4, 27.1, 26.9, 21.7; β IR v_{max} (neat) 1715, 1676, 1611, 1287 cm^{-1}; α IR v_{max} (neat) 1718, 1681, 1610, 1267 cm^{-1}; HRMS (APCI) *m/z*: $[M + H]^+$ Calcd for $(C_{28}H_{30}NO_9)^+$: 524.1915; Found 524.1921.

IV. Cyclisation with Benzyl Isocyanate and Decarboxylative Bromination

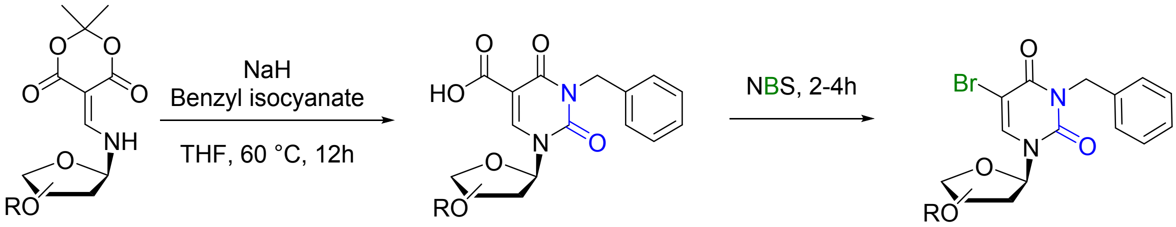

3-Benzyl-1-cyclohexyl-2,4-dioxo-1,2,3,4-tetrahydropyrimidine-5-carboxylic Acid (16a).

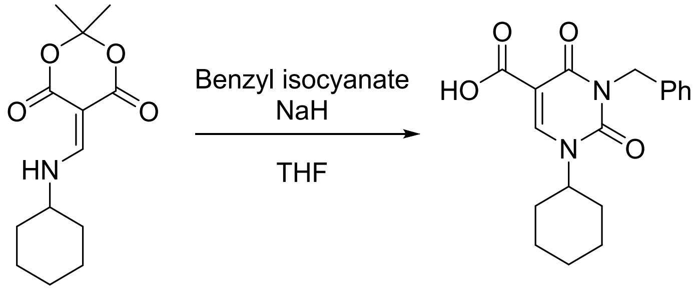

(15a) **(16a)**

Benzyl Isocyanate (50 μL, 0.41 mmol, 1.5 equiv) and enamide **15a** (69.0 mg, 0.27 mmol) in THF (1 mL) were added with stirring to NaH (60% in oil; 39.0 mg, 1.63 mmol, 6 equiv). After stirring overnight, reaction was quenched with H_2O and the mixture extracted with EtOAc. The aqueous phase was acidified with aqueous HCl (1M) and further extracted with EtOAc (5 mL x 2). The combined organic phases, after acidification, were dried ($MgSO_4$), filtered and evaporated to dryness. The residue was purified by preparative TLC (MeOH : CH_2Cl_2 , 1: 19) which gave carboxylic acid **16a** (70.0 mg, 0.21 mmol, 79%) as a white solid: R_f 0.52 (MeOH : CH_2Cl_2, 1:9); m.p. (CH_2Cl_2/pentane) (142.4 - 144.0 °C); IR ν_{max} 1748, 1710, 1626, 1426, 1237 cm^{-1}; ^{1}H-NMR (400 MHz, $CDCl_3$) δ 12.73 (s, 1H), 8.44 (s, 1H), 7.54 – 7.42 (m, 2H), 7.40 – 7.26 (m, 3H), 5.18 (s, 2H), 4.54 (tt, *J* = 11.9, 3.4 Hz, 1H), 2.02 – 1.84 (m, 4H), 1.82 – 1.72 (m, 1H), 1.52 – 1.37 (m, 4H), 1.31 – 1.12 (m, 1H); ^{13}C{^{1}H}-NMR (101 MHz, $CDCl_3$) δ 164.6, 163.6, 150.0, 147.4, 135.2, 129.4, 128.7, 128.3, 101.6, 57.7, 45.4, 32.2, 25.6, 24.9; HRMS (ESI) *m/z*: $[M + H]^+$ Calcd for $(C_{18}H_{21}N_2O_4)^+$: 329.1496; Found 329.1512. Anal. Calcd for $C_{18}H_{20}N_2O_4$**.**1 H_2O**.**1 MeOH: C, 63.32; H, 6.71; N, 7.77. Found: C, 63.39; H, 6.11; N, 7.85.

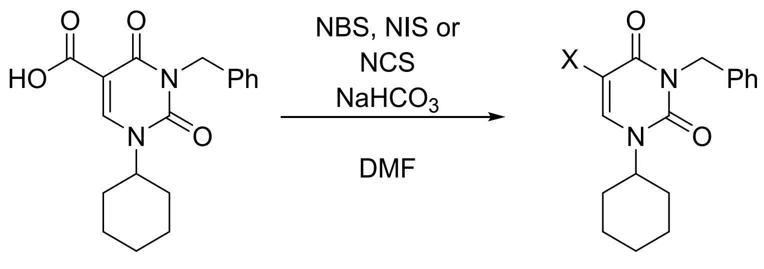

(16a)

17a, X = Br
49, X = Cl
50, X= I

3-Benzyl-5-bromo-1-cyclohexylpyrimidine-2,4(1*H*,3*H*)-dione (17a).

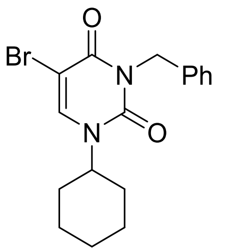

(17a)

$NaHCO_3$ (27.0 mg, 0.327 mmol, 3 equiv) and NBS (19.0 mg, 0.109 mmol, 1 equiv) were added sequentially with stirring to carboxylic acid **16a** (31.2 mg, 0.109 mmol, 1 equiv) in dry DMF (1 mL). The reaction mixture was left stirring overnight at 25 °C, when reaction was quenched with H_2O (2 mL) and the mixture extract with EtOAc (5 mL x 2). The combined organic extracts were dried ($MgSO_4$), filtered, and concentrated *in vacuo*. Chromatography (pentane : EtOAc 3 : 2) gave bromide **17a** (16.0 mg, 0.044 mmol, 40%) as a brown oil: R_f 0.73 (pentane : EtOAc 3 : 2); ^{1}H-NMR (400 MHz, $CDCl_3$) δ 7.51 (d, *J* = 7.3 Hz, 3H), 7.35 – 7.26 (m, 3H), 5.17 (s, 2H), 4.48 (ddq, *J* = 11.5, 6.8, 3.4 Hz, 1H), 1.91 (ddd, *J* = 11.0, 6.1, 3.0 Hz, 4H), 1.79 – 1.66 (m, 1H), 1.42 (ddd, *J* = 12.2, 9.3, 6.0 Hz, 4H), 1.16 (qt, *J* = 12.6, 3.0 Hz, 1H); ^{13}C{^{1}H}-NMR (101 MHz, $CDCl_3$) δ 158.9, 150.8, 138.6, 136.4, 129.5, 128.4, 127.8, 96.1, 56.4, 46.0, 32.2, 25.6, 25.1; IR υ_{max} 1705, 1657, 1625, 1440 cm^{-1}; HRMS (ESI) *m/z*: $[M+H]^+$ Calcd for $(C_{17}H_{20}BrN_2O_2)^+$: 363.0703; Found 363.0715.

3-Benzyl-5-chloro-1-cyclohexylpyrimidine-2,4(1*H*,3*H*)-dione (49).

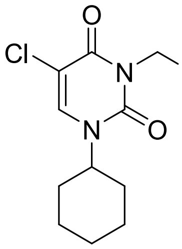

(49)

$NaHCO_3$ (38.0 mg, 0.456 mmol, 3 equiv) and NCS (30.0 mg, 0.228 mmol, 1.5 equiv) were added sequentially with stirring to the carboxylic acid **16a** (50.0 mg, 0.152 mmol, 1 equiv) in DMF (1 mL). The mixture was left stirring at 40 °C overnight, when reaction was quenched with H_2O (2 mL) and the mixture extracted with EtOAc (5 mL x 2). The combined organic extracts were dried ($MgSO_4$), filtered, and concentrated *in vacuo*. Chromatography (pentane : EtOAc 5 : 1) gave chloride **49** (30.0 mg, 0.094 mmol, 63%) as a white solid: R_f 0.63 (EtOAc : pentane 1 : 5); m.p. 98.9 – 100.9 °C (Et_2O); ^{1}H-NMR (400 MHz, $CDCl_3$) δ 7.53 – 7.48 (m, 2H), 7.41 (s, 1H), 7.34 – 7.27 (m, 3H), 5.16 (s, 2H), 4.49 (tt, *J* = 8.6, 3.5 Hz, 1H), 1.98 – 1.82 (m, 4H), 1.78 – 1.66 (m, 2H), 1.42 (m, 4H), 1.19 (m, 1H); $^{13}C\{^1H\}$-NMR (101 MHz, $CDCl_3$) δ 158.9, 150.5, 136.3, 135.9, 129.4, 128.4, 127.8, 127.5, 127.8, 108.2, 56.3, 45.8, 32.1, 25.6, 25.1; IR ν_{max} 1708, 1662, 1448 cm^{-1}; HRMS (APCI) *m/z*: [M + H]$^+$ Calcd for $(C_{17}H_{20}ClN_2O_2)^+$: 319.1208; Found 319.1210. Anal. Calcd for $C_{17}H_{19}ClN_2O_2$.0.25 H_2O: C, 63.16; H, 6.08; N, 8.66. Found: C, 63.12; H, 6.10; N, 8.49.

3-Benzyl-5-iodo-1-cyclohexylpyrimidine-2,4(1*H*,3*H*)-dione (50).

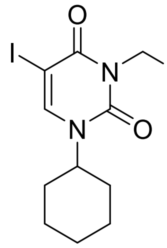

(50)

$NaHCO_3$ (38.0 mg, 0.456 mmol, 3 equiv) and NIS (40.0 mg, 0.228 mmol, 1.5 equiv) were added sequentially with stirring to carboxylic acid **16a** (50.0 mg, 0.152 mmol, 1 equiv) in dry DMF (2 mL). After stirring overnight at 50 °C, reaction was quenched with H_2O (2 mL) and the mixture extracted with EtOAc (5 mL x 2). The combined organic extracts were dried ($MgSO_4$), filtered, and concentrated *in vacuo*. Chromatography (pentane : EtOAc 5 : 1) gave iodine **50** (62.0 mg, 0.152 mmol, 100%) as an off-white solid: R_f 0.63 (EtOAc : pentane 1 : 5); m.p. 163-169.57 °C (Et_2O); ^{1}H-NMR (400 MHz, $CDCl_3$) δ 7.61 (s, 1H), 7.56 – 7.45 (m, 2H), 7.36 – 7.21 (m, 3H), 5.17 (s, 2H), 4.46 (dt, *J* = 11.8, 6.5 Hz, 1H), 1.96 – 1.79 (m, 4H), 1.79 – 1.66 (m, 1H), 1.41 (dt, *J* = 9.6, 3.0 Hz, 4H), 1.23 – 1.10 (m, 1H); $^{13}C\{^1H\}$-NMR (101 MHz, $CDCl_3$) δ 159.7,

151.1, 143.7, 136.5, 129.5, 128.4, 127.8, 67.6, 56.4, 46.3, 32.2, 25.6, 25.1; IR v_{max} (neat) 1696, 1649, 1611, 1436, 1331, 1233 cm^{-1}; HRMS (APCI) *m/z*: $[M + H]^+$ Calcd for $(C_{17}H_{20}IN_2O_2)^+$: 411.0564; Found 411.0576.

1-(3,5-Di-*O*-(4-methylbenzoyl)-2-deoxy-β-*D*-ribofuranosyl)-3-benzyl-2,4-dioxo-1,2,3,4-tetrahydropyrimidine-5-carboxylic acid (16b)

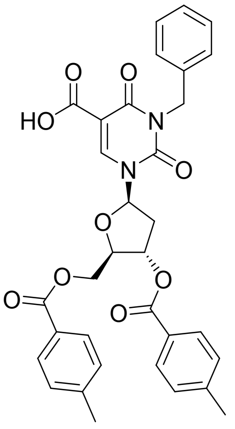

(16b)

Benzyl isocyanate (**51**) (55.0 μL, 0.450 mmol, 4 equiv) and enamide **15g** (59.0 mg, 0.112 mmol, 1 equiv) in THF (6.0 mL) were added with stirring to NaH (60% in oil; 16.0 mg, 0.676 mmol, 6 equiv). After stirring overnight, reaction was quenched with AcOH (3 mL) and the mixture was concentrated *in vacuo*. The crude ^{1}H NMR spectrum was consistent with 100% conversion to the product **16b**. Chromatography (CH_2Cl_2 : pentane 1 : 1 to MeOH : CH_2Cl_2 : pentane 1 : 5 :4) only gave small quantities of the nucleoside **16b** as a white solid: R_f 0.26 (MeOH: CH_2Cl_2 1 : 24); m.p. 121.04-122.7 °C (Et_2O); $[\alpha]_D^{25.9}$= -23.9 (*c* 1.0, $CHCl_3$); ^{1}H-NMR (400 MHz, $CDCl_3$) δ 12.49 (s, 1H), 8.82 (s, 1H), 7.93 (dd, *J* = 17.6, 8.0 Hz, 4H), 7.50 (dd, *J* = 7.3, 2.3 Hz, 2H), 7.39 – 7.30 (m, 4H), 7.19 (t, *J* = 7.5 Hz, 3H), 6.32 (dd, *J* = 8.0, 5.6 Hz, 1H), 5.60 (dt, *J* = 6.7, 1.9 Hz, 1H), 5.26 – 5.04 (m, 2H), 4.86 (dd, *J* = 11.9, 3.0 Hz, 1H), 4.73 – 4.54 (m, 2H), 2.92 (ddd, *J* = 14.5, 5.7, 1.7 Hz, 1H), 2.46 (s, 3H), 2.39 (s, 3H), 2.38 – 2.27 (m, 1H); $^{13}C\{^1H\}$-NMR (101 MHz, $CDCl_3$) δ 166.1, 165.8, 164.6, 162.6, 149.2, 145.8, 144.7, 144.4, 135.0, 129.8, 129.7, 129.5, 129.3, 129.1, 128.8, 128.5, 128.2, 126.3, 126.2, 102.2, 88.1, 84.2, 74.8, 63.7, 45.2, 39.4, 21.8, 21.7; IR v_{max} (neat) 1750, 1718, 1631, 1610, 1268 cm^{-1}; HRMS (ESI) *m/z*: $[M + H]^+$ Calcd for $(C_{33}H_{31}N_2O_9)^+$: 599.2024; Found 599.2043.

1-(2,3,5-Tri-*O*-acetyl-β-*D*-ribofuranosyl)-3-benzyl-2,4-dioxo-1,2,3,4-tetrahydropyrimidine-5-carboxylic acid (16c)

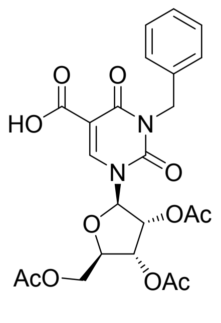

(16c)

Benzyl isocyanate (**51**) (86.0 μL, 0.698 mmol, 6 equiv) and enamide **15h** (50.0 mg, 0.116 mmol, 1 equiv) in THF (1.0 mL) were added with stirring to NaH (60% in oil; 11.0 mg, 0.465 mmol, 4 equiv). After stirring overnight, reaction was quenched with AcOH (3 mL) and the mixture was concentrated *in vacuo*. The crude ^{1}H NMR spectrum was consistent with a 100% conversion to the product. Chromatography (CH_2Cl_2 : pentane 1 : 1 to MeOH; CH_2Cl_2 : pentane 1 : 5 : 4) only gave small quantities of nucleoside **16c** as a colorless foam: R_f 0.23 (MeOH : CH_2Cl_2 1 : 24); $[\alpha]_D^{25.7}$= +19.9 (*c* 1.0, $CHCl_3$); ^{1}H-NMR (400 MHz, $CDCl_3$) δ 12.56 (s, 1H), 8.66 (s, 1H), 7.50 – 7.42 (m, 2H), 7.32 (dd, *J* = 4.9, 2.6 Hz, 3H), 6.43 (d, *J* = 5.6 Hz, 1H), 5.77 (t, *J* = 5.4 Hz, 1H), 5.38 (dd, *J* = 5.2, 3.2 Hz, 1H), 5.26 – 5.01 (m, 2H), 4.73 (q, *J* = 3.4 Hz, 1H), 4.38 – 4.15 (m, 2H), 2.15 (s, 3H), 2.07 (s, 3H), 1.73 (s, 3H); $^{13}C\{^1H\}$-NMR (101 MHz, $CDCl_3$) δ 170.1, 169.4, 168.4, 164.7, 163.1, 149.2, 147.0, 135.0, 129.3, 128.7, 128.5, 101.3, 85.6, 82.0, 71.0, 70.0, 63.1, 45.1, 20.8, 20.4, 19.9; IR υ_{max} (neat) 1749, 1718, 1636, 1423, 1222 cm^{-1}; HRMS (ESI) *m/z*: $[M + H]^+$ Calcd for $(C_{23}H_{25}N_2O_{11})^+$: 505.1453; Found 505.1469.

1-(3,5-Di-*O*-benzoyl-2-deoxy-2-fluoro-β-*D*-arabinofuranosyl)-3-benzyl-2,4-dioxo-1,2,3,4-tetrahydropyrimidine-5-carboxylic acid (16d)

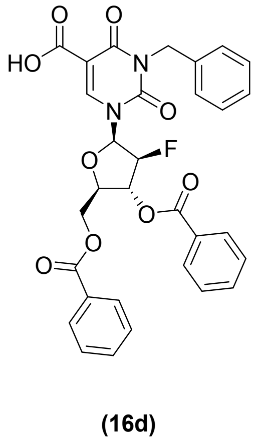

(16d)

Benzyl isocyanate (**51**) (72.0 μL, 0.584 mmol, 6 equiv) and enamide **15e** (50.0 mg, 0.097 mmol, 1 equiv) in THF (1.0 mL) were added with stirring to NaH (60% in oil; 9.0 mg, 0.389 mmol, 4 equiv). After stirring overnight, reaction was quenched with AcOH (3 mL) and the

mixture was concentrated *in vacuo*. The crude ^{1}H NMR spectrum was consistent with 100% conversion to the product **16d**. Chromatography (CH_2Cl_2 : pentane 1 : 1 to MeOH: CH_2Cl_2 : pentane 1 : 5 :4) only gave small quantities of the nucleoside **16d** as a white solid: R_f 0.31 (MeOH : CH_2Cl_2 1 : 24); $[\alpha]_D^{25.5}$= - 37.2 (*c* 1.0, $CHCl_3$); m.p. 79.0-80.9 °C (Et_2O); ^{1}H-NMR (400 MHz, $CDCl_3$) δ 12.45 (s, 1H), 8.82 (d, *J* = 1.2 Hz, 1H), 8.16 – 7.97 (m, 4H), 7.60 (d, *J* = 7.4 Hz, 2H), 7.52 – 7.40 (m, 6H), 7.34 (d, *J* = 6.4 Hz, 3H), 6.31 (dd, *J* = 19.9, 2.7 Hz, 1H), 5.62 (dd, *J* = 16.6, 2.7 Hz, 1H), 5.40 (dd, *J* = 49.8, 2.8 Hz, 1H), 5.27 – 5.04 (m, 2H), 4.80 (ddd, *J* = 57.3, 12.3, 4.4 Hz, 2H), 4.63 (q, *J* = 3.9 Hz, 1H); $^{13}C\{^{1}H\}$-NMR (101 MHz, $CDCl_3$) δ 166.2, 165.0, 164.6, 162.5, 149.1, 147.1, 134.8, 134.3, 133.5, 130.0, 129.2, 128.8, 128.6, 128.5, 102.4, 93.1, 91.2, 86.7, 86.6, 82.3, 76.4, 62.8, 45.3; ^{19}F NMR (377 MHz, $CDCl_3$) δ -200.24; IR υ_{max} (neat) 1723, 1636, 1451, 1267, 711 cm^{-1}; HRMS (ESI) *m/z*: $[M + H]^+$ Calcd for $(C_{31}H_{26}FN_2O_9)^+$: 589.1617; Found 589.1614.

1-(3,5-Di-*O*-benzoyl-2-deoxy-2-fluoro-2-methyl-β-*D*-ribofuranosyl)-3-benzyl-2,4-dioxo-1,2,3,4-tetrahydropyrimidine-5-carboxylic acid (16e)

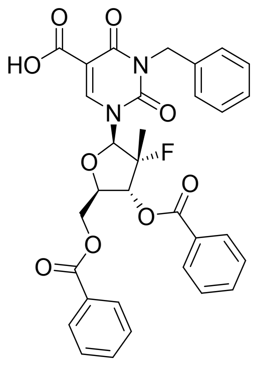

(16e)

Benzyl isocyanate (**51**) (0.51 mL, 0.417 mmol, 4 equiv) and enamide **15c** (55.0 mg, 0.104 mmol, 1 equiv) in THF (1.0 mL) were added with stirring to NaH (60% in oil; 10.0 mg, 0.417 mmol, 4 equiv). After stirring overnight, reaction was quenched with AcOH (3 mL) and the mixture was concentrated *in vacuo*. The crude ^{1}H NMR spectrum was consistent with 100% conversion to the product **16e**. Chromatography (CH_2Cl_2 : pentane 1 : 1 to MeOH : CH_2Cl_2 : pentane 1 : 5 : 4) only gave small quantities of nucleoside **16e** as a white solid with tentatively assignment of anomeric stereochemistry: R_f α 0.52 (MeOH : CH_2Cl_2 1 : 24); R_f β 0.37 (MeOH : CH_2Cl_2 1 : 24); m.p. β 134.4 ° C (Et_2O); m.p. α 117.7 ° C; β $[\alpha]_D^{25.9} = -2.6$ (*c* 1, $CHCl_3$); α $[\alpha]_D^{25.9} = +17.5$ (*c* 1, $CHCl_3$); ^{1}H-NMR β (500 MHz, $CDCl_3$) δ 12.22 (s, 1H), 8.58 (s, 1H), 8.14 – 7.97 (m, 4H), 7.64 – 7.48 (m, 2H), 7.49 – 7.35 (m, 6H), 7.37 – 7.26 (m, 3H), 6.17 (d, *J* = 18.9 Hz, 1H), 5.59 (s, 1H), 5.28 – 5.09 (m, 2H), 4.82 – 4.65 (m, 3H), 1.43 (d, *J* = 22.4 Hz, 3H); $^{13}C\{^{1}H\}$-NMR β (126 MHz, $CDCl_3$) δ 166.1, 165.3, 164.4, 162.1, 149.2, 134.8, 133.7, 133.3,

130.1, 129.9, 129.3, 129.2, 128.8, 128.6, 128.5, 103.2, 100.0, 98.5, 78.4, 73.5, 73.3, 62.5, 45.4, 18.0, 17.8.; ^{19}F{^{1}H}-NMR β (377 MHz, 328K, $CDCl_3$) δ -171.86; ^{1}H-NMR α (400 MHz, $CDCl_3$) δ 12.54 (s, 1H), 8.63 (t, *J* = 2.2 Hz, 1H), 8.04 (ddt, *J* = 27.8, 8.4, 1.6 Hz, 4H), 7.68 – 7.53 (m, 2H), 7.52 – 7.38 (m, 5H), 7.38 – 7.29 (m, 3H), 6.42 (dd, *J* = 17.5, 1.7 Hz, 1H), 5.71 (ddd, *J* = 21.4, 8.7, 1.8 Hz, 1H), 5.18 (ddd, *J* = 57.8, 13.9, 1.7 Hz, 2H), 4.93 (dt, *J* = 8.7, 3.3 Hz, 1H), 4.82 – 4.46 (m, 2H), 1.60 (d, *J* = 22.2 Hz, 3H); ^{13}C{^{1}H}-NMR α (101 MHz, $CDCl_3$) δ 165.9, 165.3, 164.6, 162.8, 149.7, 147.5, 147.5, 134.8, 134.2, 133.4, 130.1, 129.7, 129.2, 128.8, 128.6, 128.5, 128.5, 128.0, 102.4, 99.0, 97.1, 88.6, 88.5, 79.2, 74.0, 73.9, 62.8, 45.4, 18.5, 18.3; ^{19}F{^{1}H}-NMR α (377 MHz, $CDCl_3$) δ -172.31; IR v_{max} (neat) β 1750, 1718, 1635 cm^{-1}; IR v_{max} (neat) α 1714, 1635, 1600 cm^{-1}; HRMS (ES) *m/z*: $[M + H]^+$ Calcd for $(C_{32}H_{28}FN_2O_9)^+$: 603.1773; Found 603.1773.

1-(3,5-Di-*O*-benzoyl-2-deoxy-2,2-difluoro-β-*D*-ribofuranosyl)-3-benzyl-2,4-dioxo-1,2,3,4-tetrahydropyrimidine-5-carboxylic acid (16f)

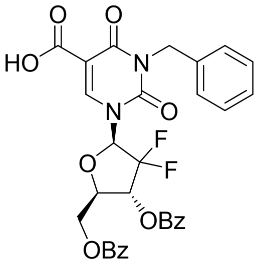

(16f)

Benzyl isocyanate (**51**) (0.14 mL, 1.14 mmol, 6 equiv) and enamide **15d** (101 mg, 0.190 mmol, 1 equiv) in THF (10 mL) were added with stirring to NaH (60% in oil; 27.0 mg, 1.14 mmol, 6 equiv). After stirring overnight, reaction was quenched with AcOH (3 mL) and the mixture was concentrated *in vacuo*. The crude ^{1}H NMR spectrum was consistent with 100% conversion to the product **16f**. Chromatography (CH_2Cl_2 : pentane 1 : 1 to MeOH : CH_2Cl_2 : pentane 1 : 5 : 4) only gave small quantities of the nucleoside **16f** as a white solid: R_f 0.53 (MeOH : CH_2Cl_2 1 : 24); m.p. 64.4 – 64.7 ° C (Et_2O); $[\alpha]_D^{26.3}$= - 57.0 (*c* 1.0, $CHCl_3$); ^{1}H-NMR (400 MHz, $CDCl_3$) δ 12.41 (s, 1H), 8.67 (s, 1H), 8.11 (td, *J* = 7.8, 1.4 Hz, 4H), 7.73 – 7.57 (m, 2H), 7.50 (dq, *J* = 11.1, 7.8 Hz, 5H), 7.37 (dd, *J* = 5.6, 1.9 Hz, 4H), 6.44 (t, *J* = 8.3 Hz, 1H), 5.68 (d, *J* = 13.0 Hz, 1H), 5.36 – 5.06 (m, 2H), 4.89 (dd, *J* = 12.5, 4.3 Hz, 1H), 4.77 – 4.63 (m, 2H); ^{13}C{^{1}H}-NMR (101 MHz, $CDCl_3$) δ 166.1, 164.6, 164.3, 162.1, 149.1, 146.6, 134.6, 134.3, 133.7, 130.1, 129.9, 129.4, 128.8, 128.7, 128.6, 103.3, 79.2, 71.1, 62.2, 45.6, 43.8; ^{19}F{^{1}H}-NMR (377 MHz, $CDCl_3$) δ -114.15, -114.81;IR v_{max} (neat) 1723, 1640, 1267, 1092, 710 cm^{-1}; HRMS (ES) *m/z*: $[M + H]^+$ Calcd for $(C_{31}H_{25}F_2N_2O_9)^+$: 607.1523; Found 607.1518.

1-(3,5-Di-*O*-(4-methylbenzoyl)-2-deoxy-β-*D*-ribofuranosyl)-3-benzyl-5-bromo-2,4-dioxo-1,2,3,4-tetrahydropyrimidine (17b)

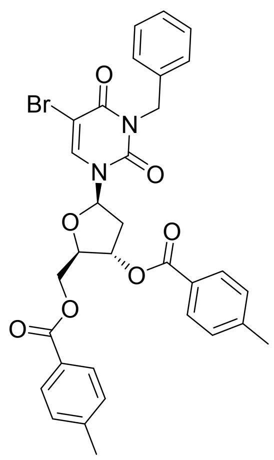

(17b)

Benzyl isocyanate (**51**) (0.25 mL, 1.90 mmol, 6 equiv) and enamide **15g** (165 mg, 0.316 mmol, 1 equiv) in THF (10 mL) were added with stirring to NaH (60% in oil; 45.0 mg, 1.90 mmol, 6 equiv). After stirring overnight at 60 °C, NBS (168 mg, 0.945 mmol, 3 equiv) was added with stirring and left to react for an additional 3 h. Reaction was quenched with AcOH (3 mL) and the mixture was concentrated *in vacuo*. Chromatography of the residue (EtOAc : pentane 1 : 4) gave nucleoside **17b** (104 mg, 0.164 mmol, 52%) as a white solid: R_f 0.58 (EtOAc : pentane 1 : 4); m.p. 72.2- 72.8 °C (Et_2O); $[\alpha]_D^{26.2}$= -73.1 (*c* 1.0, $CHCl_3$); ^{1}H-NMR (400 MHz, $CDCl_3$) δ 7.92 (t, *J* = 8.6 Hz, 4H), 7.87 (s, 1H), 7.53 – 7.46 (m, 2H), 7.33 – 7.27 (m, 5H), 7.24 (d, *J* = 8.0 Hz, 2H), 6.40 (dd, *J* = 8.6, 5.4 Hz, 1H), 5.64 – 5.53 (m, 1H), 5.21 – 5.05 (m, 2H), 4.81 – 4.63 (m, 2H), 4.56 (q, *J* = 2.7 Hz, 1H), 2.84 – 2.70 (m, 1H), 2.42 (d, *J* = 11.6 Hz, 6H), 2.23 (ddd, *J* = 14.7, 8.7, 6.4 Hz, 1H); ^{13}C{^{1}H}-NMR (101 MHz, $CDCl_3$) δ 166.1, 166.0, 158.7, 150.1, 144.7, 144.6, 136.6, 136.0, 129.8, 129.6, 129.5, 129.3, 129.1, 128.6, 128.5, 128.0, 126.3, 126.2, 97.3, 86.5, 83.4, 74.9, 64.0, 45.8, 38.8, 21.8, 21.7; IR v_{max} (neat) 1716, 1664, 1610, 1446, 1266, 752 cm^{-1}; HRMS (ESI) *m/z*: $[M + H]^+$ Calcd for $(C_{32}H_{30}BrN_2O_7)^+$: 633.1231; Found 633.1224.

1-(2,3,5-Tri-*O*-acetyl-β-*D*-ribofuranosyl)-3-benzyl-5-bromo-2,4-dioxo-1,2,3,4-tetrahydropyrimidine (17c)

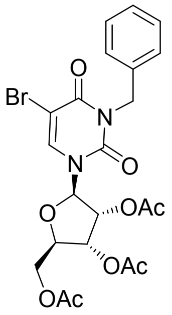

(17c)

Benzyl isocyanate (**51**) (0.31 mL, 2.46 mmol, 6 equiv) and enamide **15h** (176 mg, 0.410 mmol, 1 equiv) in THF (10 mL) were added with stirring to NaH (60% in oil; 59.0 mg, 2.46 mmol, 6 equiv). After stirring overnight at 60 °C, NBS (218 mg, 1.23 mmol, 3 equiv) was added with stirring and left to react for an additional 3 h. Reaction was quenched with AcOH (3 mL) and and the mixture was concentrated *in vacuo*. Chromatography of the residue (EtOAc : pentane 1 : 4) gave nucleoside **17c** (129 mg, 0.239 mmol, 58%) as a colorless oil: R_f 0.68 (EtOAc : pentane 1 : 1); $[\alpha]_D^{25.7}$= +6.1 (*c* 1.0, $CHCl_3$); ^{1}H-NMR (400 MHz, $CDCl_3$) δ 7.75 (s, 1H), 7.51 – 7.42 (m, 2H), 7.27 (s, 3H), 6.37 (d, *J* = 5.3 Hz, 1H), 5.71 (t, *J* = 5.2 Hz, 1H), 5.37 (dd, *J* = 5.1, 3.8 Hz, 1H), 5.20 – 5.03 (m, 2H), 4.61 (q, *J* = 3.7 Hz, 1H), 4.24 (ddd, *J* = 52.7, 12.4, 3.6 Hz, 2H), 2.13 (d, 6H), 1.76 (s, 3H); ^{13}C{^{1}H}-NMR (101 MHz, $CDCl_3$) δ 170.2, 169.2, 168.5, 158.8, 149.9, 137.9, 136.1, 129.4, 128.5, 128.0, 95.7, 85.0, 80.9, 71.0, 70.0, 63.2, 45.7, 20.8, 20.5, 20.0; IR ν_{max} (neat) 1746, 1707, 1658, 1441, 1207, 728, 698 cm^{-1}; HRMS (ESI) *m/z*: [M + H]$^+$ Calcd for $(C_{22}H_{24}BrN_2O_9)^+$: 539.0660; Found 539.0676.

1-(3,5-Di-*O*-benzoyl-2-deoxy-2-fluoro-β-*D*-arabinofuranosyl)-3-benzyl-5-bromo-2,4-dioxo-1,2,3,4-tetrahydropyrimidine (17d)

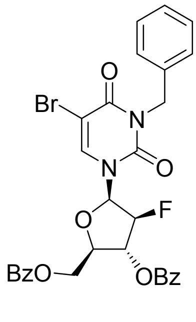

(17d)

Benzyl isocyanate (**51**) (0.20 mL, 1.64 mmol, 6 equiv) and enamide **15e** (140 mg, 0.272 mmol, 1 equiv) in THF (10 mL) were added with stirring to NaH (60% in oil; 39.0 mg, 1.64 mmol, 6 equiv). After stirring overnight at 60 °C, NBS (144 mg, 0.816 mmol, 3 equiv) was added with stirring and left to react for an additional 3 h. Reaction was quenched with AcOH (3 mL) and the mixture was concentrated *in vacuo*. Chromatography of the residue (EtOAc : pentane 1 :

4) gave nucleoside **17d** (0.067 g, 0.107 mmol, 40%) as a white solid: R_f 0.28 (EtOAc : pentane 1 : 4); m.p. (Et_2O) 58.1-58.6 °C; $[\boldsymbol{\alpha}]_{\boldsymbol{D}}^{25.4}$= -31.7 (*c* 1.0, $CHCl_3$); ^{1}H-NMR (400 MHz, $CDCl_3$) δ 8.14 – 7.95 (m, 4H), 7.90 (d, *J* = 1.9 Hz, 1H), 7.70 – 7.54 (m, 2H), 7.54 – 7.44 (m, 6H), 7.34 – 7.24 (m, 3H), 6.32 (dd, *J* = 21.3, 2.7 Hz, 1H), 5.61 (dd, *J* = 16.9, 2.7 Hz, 1H), 5.34 (dd, *J* = 50.0, 2.8 Hz, 1H), 5.25 – 5.06 (m, 2H), 4.81 (qd, *J* = 12.3, 4.1 Hz, 2H), 4.52 (dt, *J* = 4.7, 2.5 Hz, 1H); ^{19}F{^{1}H}-NMR (377 MHz, $CDCl_3$) δ -201.06; ^{13}C{^{1}H}-NMR (126 MHz, $CDCl_3$) δ 166.2, 165.1, 158.6, 150.0, 138.2, 138.2, 135.8, 134.2, 133.5, 129.9, 129.8, 129.2, 129.2, 128.7, 128.6, 128.5, 128.1, 128.0, 96.8, 93.0, 91.5, 85.8, 85.7, 81.9, 63.0, 45.8; IR v_{max} (neat) 1710, 1662, 1256, 710 cm^{-1}; HRMS (ESI) *m/z*: $[M + H]^+$ Calcd for $(C_{30}H_{25}BrFN_2O_7)^+$: 623.0824; Found 623.0852.

1-(3,5-Di-*O*-benzoyl-2-deoxy-2-fluoro-2-methyl-β-*D*-ribofuranosyl)-3-benzyl-5-bromo-2,4-dioxo-1,2,3,4-tetrahydropyrimidine (17e)

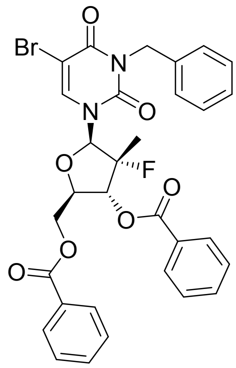

(17e)

Benzyl isocyanate (**51**) (86.0 μL, 0.682 mmol, 6 equiv) and enamide **15c** (60.0 mg, 0.113 mmol, 1 equiv) in THF (10 mL) were added with stirring to NaH (60% in oil; 12.0 mg, 0.454 mmol, 4 equiv). After stirring overnight at 60 °C, NBS (40.0 mg, 0.227 mmol, 3 equiv) was added with stirring and left to react for an additional 3 h. Reaction was quenched with AcOH (3 mL) and the mixture was concentrated *in vacuo*. Chromatography of the residue (EtOAc : pentane 1 : 4) gave nucleoside **17e** (28.0 mg, 0.045 mmol, 40%) as a white solid. Anomeric stereochemistry was tentatively assigned as the β-anomer: R_f 0.24 (Et_2O : pentane 3 : 7); $[\boldsymbol{\alpha}]_{\boldsymbol{D}}^{25.7}$= -3.3 (*c* 1.0, $CHCl_3$); ^{1}H-NMR (400 MHz, $CDCl_3$) δ 8.07 (dd, *J* = 9.5, 7.5 Hz, 4H), 7.73 (s, 1H), 7.60 (dt, *J* = 19.9, 7.5 Hz, 2H), 7.46 (dt, *J* = 12.0, 8.3 Hz, 5H), 7.37 – 7.27 (m, 4H), 6.27 (s, 1H), 5.53 (s, 1H), 5.29 – 5.00 (m, 2H), 4.85 (dd, *J* = 12.9, 2.6 Hz, 1H), 4.65 (dd, *J* = 9.6, 3.0 Hz, 1H), 4.55 (dd, *J* = 12.8, 3.7 Hz, 1H), 1.42 (d, *J* = 22.4 Hz, 3H); ^{13}C{^{1}H}-NMR (101 MHz, 328K, $CDCl_3$) δ 166.1, 165.4, 158.3, 150.2, 135.9, 134.0, 133.4, 130.1, 129.9, 129.5, 129.2, 128.7, 128.7, 128.5, 128.5, 128.0, 100.6, 98.7, 97.9, 77.7, 72.7, 72.5, 61.9, 45.9, 17.7, 17.5; ^{19}F{^{1}H}-NMR (377 MHz, 328K, $CDCl_3$) δ -168.64, -173.78; IR v_{max} (neat) 1723, 1667,

1439, 1267, 710 cm^{-1}; HRMS (ESI) *m/z*: [M + H]$^+$ Calcd for $(C_{31}H_{27}BrFN_2O_7)^+$: 637.0980; Found 637.0985.

1-(3,5-Di-*O*-benzoyl-2-deoxy-2,2-difluoro-β-*D*-ribofuranosyl)-3-benzyl-5-bromo-2,4-dioxo-1,2,3,4-tetrahydropyrimidine (17f)

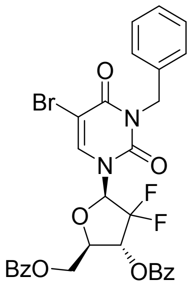

(17f)

Benzyl isocyanate (**51**) (0.28 mL, 2.26 mmol, 6 equiv) and enamide **15d** (200 mg, 0.374 mmol, 1 equiv) in THF (10 mL) were added with stirring to NaH (60% in oil; 54.0 mg, 2.26 mmol, 6 equiv). After stirring overnight at 60 °C, NBS (200 mg, 1.128 mmol, 3 equiv) was added with stirring and left to react for an additional 3 h. Reaction was quenched with AcOH (3 mL) and the mixture was concentrated *in vacuo*. Chromatography of the residue (EtOAc : pentane 1 : 4) gave nucleoside **17f** (152 mg, 0.236 mmol, 63%) as a white solid: R_f 0.33 (EtOAc : pentane 1 : 4); m.p. 142.5-143.4 °C (Et_2O); $[\alpha]_D^{25.2}$ = -52.4 (*c* 1.0, $CHCl_3$); ^{1}H-NMR (400 MHz, $CDCl_3$) δ 8.16 – 7.98 (m, 4H), 7.70 (d, *J* = 1.7 Hz, 1H), 7.68 – 7.56 (m, 2H), 7.49 (dt, *J* = 11.5, 8.1 Hz, 6H), 7.30 (q, *J* = 7.6, 6.5 Hz, 3H), 6.42 (dd, *J* = 11.5, 6.3 Hz, 1H), 5.75 – 5.53 (m, 1H), 5.30 – 5.01 (m, 2H), 4.78 (qd, *J* = 12.6, 3.5 Hz, 2H), 4.59 (q, *J* = 4.0 Hz, 1H); $^{13}C\{^1H\}$-NMR (101 MHz, $CDCl_3$*d*) δ 166.0, 164.7, 158.3, 150.1, 137.2, 135.7, 134.3, 133.7, 130.1, 129.7, 129.4, 128.9, 128.7, 128.5, 128.1, 127.7, 97.9, 78.9, 71.6, 71.1, 62.3, 46.1; $^{19}F\{^1H\}$-NMR (377 MHz, $CDCl_3$) δ -114.91, -115.56; IR v_{max} (neat) 1717, 1668, 1436, 1263, 1092, 708 cm^{-1}; HRMS (ESI) *m/z*: [M + H]$^+$ Calcd for $(C_{30}H_{24}BrF_2N_2O_7)^+$: 641.0729; Found 641.0733.

V. Cyclization with 2,4-Dimethoxybenzyl isocyanate and Full Deprotection

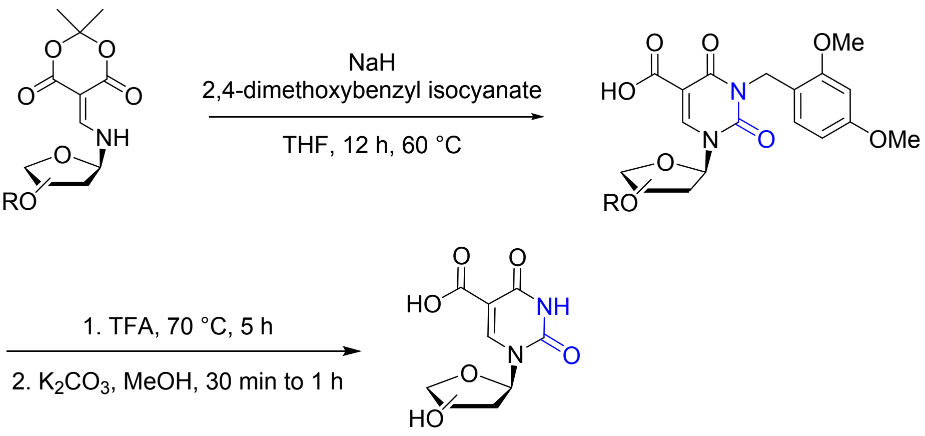

1-(3,5-Di-*O*-(4-methylbenzoyl)-2-deoxy-β-*D*-ribofuranosyl)-3-(2,4-dimethoxybenzyl)-2,4-dioxo-1,2,3,4-tetrahydropyrimidine-5-carboxylic acid (18a)

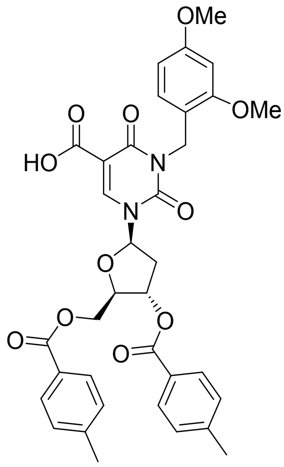

(18a)

2,4-Dimethoxybenzyl isocyanate (**52**) (0.20 mL, 1.20 mmol, 4 equiv) and enamide **15g** (157 mg, 0.299 mmol, 1 equiv) in THF (6 mL) were added with stirring to NaH (60% in oil; 43.0 mg, 1.80 mmol, 6 equiv). After stirring overnight, reaction was quenched with AcOH (3 mL) and the mixture directly loaded onto silica and chromatographed (CH_2Cl_2 : pentane 1 : 1 to MeOH : CH_2Cl_2 : pentane 1 : 5 : 4) to give the nucleoside **18a** (87.0 mg, 0.132 mmol, 44%) as a white solid: R_f 0.36 (MeOH : CH_2Cl_2 1 : 24); m.p. 123.3-123.6 (Et_2O); $[\alpha]_D^{25.5}$ = -28.7 (*c* 1.0, $CHCl_3$); ^{1}H-NMR (400 MHz, $CDCl_3$) δ 12.59 (s, 1H), 8.84 (s, 1H), 7.92 (dd, *J* = 8.0, 1.7 Hz, 4H), 7.33 – 7.16 (m, 5H), 7.07 (d, *J* = 8.1 Hz, 1H), 6.44 (d, *J* = 8.6 Hz, 2H), 6.32 (dd, *J* = 7.9, 5.6 Hz, 1H), 5.59 (d, *J* = 6.2 Hz, 1H), 5.26 – 5.02 (m, 2H), 4.86 (dd, *J* = 11.5, 2.6 Hz, 1H), 4.72 – 4.57 (m, 2H), 3.80 (d, *J* = 4.7 Hz, 6H), 2.90 (dd, *J* = 14.5, 5.6 Hz, 1H), 2.41 (d, *J* = 20.6 Hz, 6H), 2.32 (dt, *J* = 15.0, 7.4 Hz, 1H); ^{13}C{^{1}H}-NMR (126 MHz, $CDCl_3$) δ 166.1, 165.8, 164.7, 162.8, 160.8, 158.4, 149.1, 145.6, 144.6, 144.4, 129.8, 129.8, 129.3, 129.3, 115.1, 104.2, 102.1,

98.7, 87.9, 84.2, 77.3, 77.0, 76.8, 74.8, 63.7, 55.6, 55.4, 40.6, 39.3, 21.8, 21.7; IR v_{max} (neat) 1718, 1612, 1636, 1267 cm^{-1}; HRMS (ESI) *m/z*: $[M + H]^+$ Calcd for $(C_{35}H_{35}N_2O_{11})^+$: 659.2235; Found 659.2233.

1-(2-Deoxy-β-D-ribofuranosyl)-2,4-dioxo-1,2,3,4-tetrahydropyrimidine-5-carboxylic acid (19a)

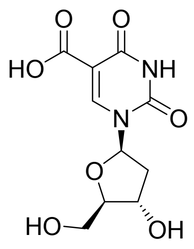

(19a)

2,4-Dimethoxybenzyl isocyanate (**52**) (1.56 mL, 1.21 mmol, 4 equiv) and enamide **15g** (1.21 g, 2.32 mmol, 1 equiv) in THF (10 mL) were added with stirring to NaH (60% in oil; 220 mg, 9.27 mmol, 4 equiv). After stirring overnight, reaction was quenched with HCO_2H (2 mL), and the mixture was filtered and concentrated *in vacuo*. CF_3CO_2H (3 mL) was added with stirring to the residue in PhMe (10 mL). After stirring overnight at 75 °C, the solution was co-evaporated with PhMe to dryness. K_2CO_3 (960 mg, 6.95 mmol, 3 equiv) was added with stirring to the crude residue in MeOH (20 mL). After stirring overnight, the solution was quenched with Dowex 50 (pH of 6-7), filtered and concentrated *in vacuo*. Purification by reverse phase C18 silica (H_2O/MeCN with 0.1% HCO_2H in MeCN : H_2O 0 : 1 to 3 : 7) gave nucleoside carboxylic acid **19a** (214 mg, 0.785 mmol, 34 %) as a white solid: R_f 0.35 (MeOH : CH_2Cl_2 1 : 4 with 0.1% HCO_2H); m.p. 168.8-170.8 °C (H_2O); $[\alpha]_D^{26.3} = +25.3°$ (*c* 1.0, H_2O); ^{1}H-NMR (400 MHz, D_2O) δ 8.86 (s, 1H), 6.20 (t, *J* = 6.1 Hz, 1H), 4.44 (dd, *J* = 7.7, 3.2 Hz, 1H), 4.10 – 4.02 (m, 1H), 3.90 – 3.74 (m, 2H), 2.50 – 2.34 (m, 2H); ^{13}C{^{1}H}-NMR (101 MHz, D_2O) δ 164.2, 150.9, 150.3, 149.0, 103.5, 87.1, 86.7, 69.9, 60.6, 39.5; IR v_{max} (neat) 3293, 1709, 1616, 1430 cm^{-1}; HRMS (ESI) *m/z*: $[M + H]^+$ Calcd for $(C_{10}H_{13}N_2O_7)^+$: 273.0717; Found 273.0721; Anal. Calcd for $C_{10}H_{12}N_2O_7$.0.5 H_2O: C, 42.71; H, 4.66; N, 9.96; Found: C, 42.78; H, 4.03; N, 9.44. NMR spectra (^{1}H NMR, ^{13}C NMR) were in reasonable agreement with literature data.[13]

1-(2,3,5-Tri-*O*-acetyl-β-*D*-ribofuranosyl)-3-(2,4-dimethoxybenzyl)-2,4-dioxo-1,2,3,4-tetrahydropyrimidine-5-carboxylic acid (18b)

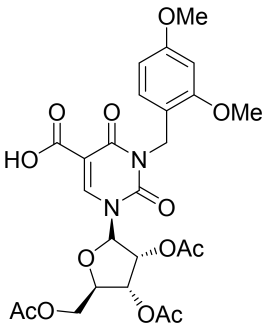

(18b)

2,4-Dimethoxybenzyl isocyanate (**52**) (0.39 mL, 2.34 mmol, 4 equiv) and enamide **15h** (252 mg, 0.586 mmol, 1 equiv) in THF (10 mL) were added with stirring to NaH (60% in oil; 84.0 mg, 3.52 mmol, 6 equiv). After stirring overnight, reaction was quenched with AcOH (3 mL) and the mixture directly loaded onto silica and chromatographed (CH_2Cl_2 : pentane 1 : 1 to MeOH; CH_2Cl_2 : pentane 1 : 5 :4) to give the nucleoside **18b** (169 mg, 0.309 mmol, 53%) as a white solid: R_f 0.2 (MeOH : CH_2Cl_2 1 : 24); m.p. (Et_2O) 66.0 °C; $[\alpha]_D^{26.3}$= +24.7 (*c* 1.0, $CHCl_3$); ^{1}H-NMR (400 MHz, $CDCl_3$) δ 12.73 (s, 1H), 8.69 (s, 1H), 7.03 (d, *J* = 8.3 Hz, 1H), 6.47 – 6.37 (m, 3H), 5.75 (t, *J* = 5.3 Hz, 1H), 5.39 (dd, *J* = 5.1, 3.7 Hz, 1H), 5.14 (s, 2H), 4.73 (q, *J* = 3.6 Hz, 1H), 4.27 (ddd, *J* = 52.8, 12.5, 3.5 Hz, 2H), 3.79 (d, *J* = 11.5 Hz, 6H), 2.12 (d, *J* = 18.8 Hz, 6H), 1.86 (s, 3H); ^{13}C{^{1}H}-NMR (101 MHz, $CDCl_3$) δ 170.1, 169.4, 168.5, 164.7, 163.3, 160.8, 158.4, 149.0, 147.0, 129.4, 115.1, 104.2, 101.2, 98.7, 85.4, 81.6, 71.0, 70.1, 63.1, 55.6, 55.4, 40.4, 20.8, 20.4, 20.0; IR ν_{max} (neat) 1750, 1722, 1632, 1209 cm^{-1}; HRMS (ESI) *m/z*: $[M + H]^+$ Calcd for $(C_{25}H_{29}N_2O_{13})^+$: 565.1664; Found 565.1676.

1-(β-*D*-Ribofuranosyl)-2,4-dioxo-1,2,3,4-tetrahydropyrimidine-5-carboxylic acid (19b)

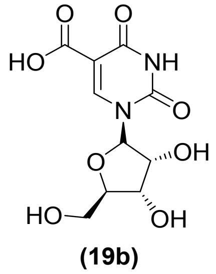

(19b)

2,4-Dimethoxybenzyl isocyanate (**52**) (1.03 mL, 6.21 mmol, 4 equiv) and enamide **15h** (667 mg, 1.55 mmol, 1 equiv) in THF (10 mL) were added with stirring to NaH (60% in oil; 223 mg, 9.32 mmol, 6 equiv). After stirring overnight, the reaction was quenched with HCO_2H (2 mL), filtered and concentrated *in vacuo*. CF_3CO_2H (3 mL) was added with stirring to the residue in

PhMe (10 mL), and, after stirring overnight at 75 °C, the solution was co-evaporated with PhMe to dryness. K_2CO_3 (644 mg, 4.65 mmol, 3 equiv) was added with stirring to the residue in MeOH (20 mL). After stirring overnight, reaction was quenched with Dowex 50 (pH of 6-7), and the mixture was filtered and concentrated *in vacuo*. Purification of the residue by reverse phase C18 silica (H_2O : MeCN with 0.1% HCO_2H in MeCN : H_2O 0 : 1 to 3 : 7) gave nucleoside carboxylic acid **19b** (127 mg, 0.44 mmol, 28%) as a white solid: R_f 0.54 (MeOH : CH_2Cl_2 1 : 4 + 0.1% HCO_2H); m.p. 119.86-120°C (H_2O); $[\alpha]_D^{25.5}$ = -5.5° (*c* 1, H_2O); ^{1}H-NMR (400 MHz, D_2O) δ 8.53 (s, 1H), 6.10 (d, *J* = 4.2 Hz, 1H), 4.42 (t, *J* = 4.4 Hz, 1H), 4.34 – 4.16 (m, 2H), 3.87 – 3.56 (m, 2H); ^{13}C{^{1}H}-NMR (101 MHz, D_2O) δ 166.2, 164.5, 150.2, 150.1, 102.0, 87.2, 83.9, 70.7, 70.1, 60.6; IR υ_{max} (neat) 3327, 1675, 1586 cm^{-1}; HRMS (ESI) *m/z*: $[M + H]^+$ Calcd for $(C_{10}H_{13}N_2O_8)^+$: 289.0666; Found 289.0682.

1-(3,5-Di-*O*-benzoyl-2-deoxy-2-fluoro-β-*D*-arabinofuranosyl)-3-(2,4-dimethoxybenzyl)-2,4-dioxo-1,2,3,4-tetrahydropyrimidine-5-carboxylic acid (18c)

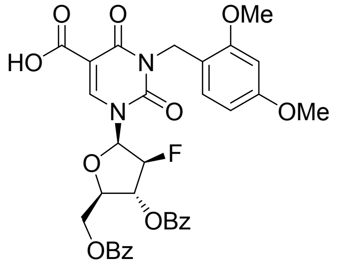

(18c)

2,4-Dimethoxybenzyl isocyanate (**52**) (0.29 mL, 1.77 mmol, 6 equiv) and enamide **15e** (155 mg, 0.293 mmol, 1 equiv) in THF (10 mL) were added with stirring to NaH (60% in oil; 42.0 mg, 1.763 mmol, 6 equiv). After stirring overnight, reaction was quenched with AcOH (3 mL) and the mixture directly loaded onto silica and chromatographed (CH_2Cl_2 : pentane 1 : 1 to MeOH; CH_2Cl_2 : pentane 1 : 5 : 4) to give the nucleoside **18c** (636 mg, 0.095 mmol, 33%) as a white solid: R_f 0.38 (4% MeOH/ CH_2Cl_2); $[\alpha]_D^{25.6}$= -6.44 (*c* 1.0, $CHCl_3$); m.p. 89.45- 91.62 °C (Et_2O); ^{1}H-NMR (400 MHz, $CDCl_3$) δ 12.81 (s, 1H), 8.84 (s, 1H), 8.07 (d, *J* = 7.7 Hz, 4H), 7.60 (m, 2H), 7.46 (dt, *J* = 16.2, 7.6 Hz, 4H), 7.06 (d, *J* = 8.1 Hz, 1H), 6.44 (s, 2H), 6.29 (d, *J* = 19.6 Hz, 1H), 5.62 (d, *J* = 16.5 Hz, 1H), 5.41 (d, *J* = 49.8 Hz, 1H), 5.26 – 5.03 (m, 2H), 4.95 – 4.71 (m, 2H), 4.63 (s, 1H), 3.79 (s, 6H); ^{13}C{^{1}H}-NMR (101 MHz, $CDCl_3$) δ 165.0, 160.8, 1 58.4, 148.9, 147.1, 134.2, 133.5, 129.9, 129.8, 129.1, 128.8, 128.6, 128.1, 114.9, 104.2, 102.0, 98.7, 93.1, 91.2, 86.8, 86.6, 82.4, 76.4, 62.8, 55.6, 55.4, 40.9; ^{19}F{^{1}H}-NMR (377 MHz, $CDCl_3$) δ -200.43; IR v_{max} (neat) 1718, 1634, 1450, 1262, 709 cm^{-1}; HRMS (ESI) *m/z*: $[M + H]^+$ Calcd for $(C_{33}H_{30}FN_2O_{11})^+$: 649.1828; Found 649.1846.

1-(2-Deoxy-2-fluoro-β-*D*-arabinofuranosyl)-2,4-dioxo-1,2,3,4-tetrahydropyrimidine-5-carboxylic acid (19c)

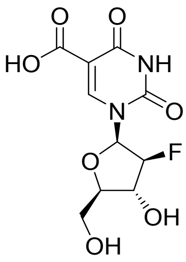

(19c)

2,4-Dimethoxybenzyl isocyanate (**52**) (0.96 mL, 5.77 mmol, 3 equiv) and enamide **15e** (989 mg, 1.93 mmol, 1 equiv) in THF (10 mL) were added with stirring to NaH (60% in oil; 184 mg, 7.70 mmol, 4 equiv). After stirring overnight, reaction was quenched with HCO_2H (2 mL), and the mixture was filtered and concentrated *in vacuo*. CF_3CO_2H (3 mL) was added with stirring to the residue in PhMe (10 mL). After further stirring overnight at 75 °C, the solution was co-evaporated with PhMe to dryness. K_2CO_3 (807 mg, 5.84 mmol, 3 equiv) was added with stirring to the residue in MeOH (20 mL), and, after further stirring overnight, reaction was quenched with Dowex 50 (pH of 6-7), and the mixture was filtered and concentrated *in vacuo*. Purification by reverse phase C18 silica (H_2O/MeCN with 0.1% HCO_2H in MeCN : H_2O 0 : 1 to 3 : 7) gave nucleoside carboxylic acid **19c** (255 mg, 0.456 mmol, 46%) as a white solid: R_f 0.48 (MeOH : CH_2Cl_2 1 : 4 with 0.1% HCO_2H); m.p. 172.25-175.2 °C ((H_2O); $[\alpha]_D^{26.3} =$ +63.0° (*c* 1, H_2O); ^{1}H-NMR (400 MHz, D_2O) δ 8.39 (d, *J* = 1.6 Hz, 1H), 6.29 (dd, *J* = 16.7, 4.0 Hz, 1H), 5.19 (dt, *J* = 51.4, 3.3 Hz, 1H), 4.44 (ddd, *J* = 18.8, 4.9, 2.7 Hz, 1H), 4.12 – 4.03 (m, 1H), 3.94 – 3.80 (m, 2H); ^{19}F{^{1}H}-NMR (377 MHz, D_2O) δ -199.54; ^{13}C{^{1}H}-NMR (101 MHz, D_2O) δ 168.9, 163.2, 150.8, 146.2, 109.6, 95.6, 93.7, 84.0, 83.9, 83.7, 73.4, 73.2, 60.4; IR ν_{max} (neat) 1718, 1634, 1450, 1262 cm^{-1}; HRMS (ESI) *m/z*: [M + H]$^+$ Calcd for $(C_{10}H_{12}FN_2O_7)^+$: 291.0623; Found 291.0627.

1-(3,5-Di-*O*-benzoyl-2-deoxy-2-fluoro-2-methyl-β-*D*-ribofuranosyl)-3-(2,4-dimethoxybenzyl)-2,4-dioxo-1,2,3,4-tetrahydropyrimidine-5-carboxylic acid (18d)

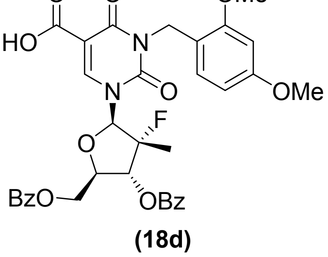

(18d)

2,4-Dimethoxybenzyl isocyanate (**52**) (0.29 mL, 1.77 mmol, 6 equiv) and enamide **15c** (155 mg, 0.293 mmol, 1 equiv) in THF (10 mL) were added with stirring to NaH (60% in oil; 43.0 mg, 1.76 mmol, 6 equiv). After stirring overnight, reaction was quenched with AcOH (3 mL) and the mixture directly loaded onto silica and chromatographed (CH_2Cl_2 : pentane 1 : 1 to 1:5:4; MeOH : CH_2Cl_2 : pentane 1 : 5 : 4) to give the nucleoside **18d** (63.0 mg, 0.095 mmol, 33%) as a white solid with the anomeric stereochemistry tentatively assigned as the β-anomer: R_f 0.38 (MeOH : CH_2Cl_2 1 : 24); m.p. 89.45-91.62 °C (Et_2O); $[\boldsymbol{\alpha}]_{\boldsymbol{D}}^{25.6}$= - 6.4 (*c* 1.0, $CHCl_3$); ^{1}H-NMR (400 MHz, $CDCl_3$) δ 12.52 (s, 1H), 8.59 (s, 1H), 8.05 (dd, *J* = 8.0, 4.5 Hz, 4H), 7.50 (ddt, *J* = 55.1, 24.6, 7.5 Hz, 6H), 7.09 (d, *J* = 8.5 Hz, 1H), 6.43 (d, *J* = 6.9 Hz, 2H), 6.31 – 5.90 (m, 1H), 5.57 (s, 1H), 5.26 – 5.00 (m, 2H), 4.71 (s, 3H), 3.79 (d, *J* = 6.5 Hz, 7H), 1.44 (d, *J* = 22.5 Hz, 3H); ^{13}C{^{1}H}-NMR (126 MHz, $CDCl_3$) δ 166.2, 165.3, 164.5, 162.6, 160.9, 158.5, 148.8, 133.9, 133.4, 130.2, 130.1, 129.9, 129.1, 128.6, 128.5, 128.4, 114.7, 104.1, 102.7, 98.7, 78.2, 73.1, 62.5, 55.6, 55.4, 41.2, 17.9, 17.7; ^{19}F{^{1}H}-NMR (377 MHz, $CDCl_3$) δ -148.59, -149.50; IR v_{max} (neat) 1722, 1638, 1587, 1271, 710 cm^{-1}; HRMS (ESI) *m/z*: $[M + H]^+$ Calcd for $(C_{34}H_{32}FN_2O_{11})^+$: 663.1985; Found 663.1996;

1-(2-Deoxy-2-fluoro-2-methyl-*D*-ribofuranosyl)-2,4-dioxo-1,2,3,4-tetrahydropyrimidine-5-carboxylic acid (19d)

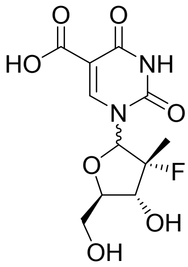

(19d)

2,4-Dimethoxybenzyl isocyanate (**52**) (0.92 mL, 5.51 mmol, 6 equiv) and enamide **15c** (484 mg, 0.917 mmol, 1 equiv) in THF (10 mL) were added with stirring to NaH (60% in oil; 132 mg, 5.51 mmol, 6 equiv). After stirring overnight, reaction was quenched with HCO_2H (2 mL), and the mixture was filtered and concentrated *in vacuo*. CF_3CO_2H (3 mL) was added with stirring to the residue in PhMe (10 mL). After stirring overnight at 75 °C, the solution was co-evaporated with PhMe to dryness, when K_2CO_3 (380 mg, 2.75 mmol, 3 equiv) was added with stirring to the residue in MeOH (20 mL). After stirring overnight, the solution was quenched with Dowex 50 (pH of 6-7), filtered and concentrated *in vacuo*. Purification by reverse phase C18 silica (H_2O/MeCN with 0.1% HCO_2H in MeCN : H_2O 0 : 1 to 3 : 7) gave nucleoside carboxylic acid **19d** (110 mg, 0.361 mmol, 40%) as a white solid. Anomeric stereochemistry was tentatively assigned: R_f 0.48 (MeOH : CH_2Cl_2 1 : 4 with 0.1% HCO_2H); m.p. β- anomer

148.6-150.8 °C (H_2O); m.p. α- anomer 174.32-175.6 °C; β- anomer $[\alpha]_D^{26.3}$ = -7.2° (*c* 1.0, H_2O); α- anomer $[\alpha]_D^{26.3}$ = +44.0° (*c* 1.0, H_2O); ^{1}H-NMR β- anomer (400 MHz, D_2O) δ 8.43 (d, *J* = 2.7 Hz, 1H), 6.28 (d, *J* = 19.7 Hz, 1H), 4.38 (ddt, *J* = 9.2, 4.4, 2.1 Hz, 1H), 4.20 (dd, *J* = 24.3, 9.5 Hz, 1H), 4.00 – 3.68 (m, 2H), 1.50 (d, *J* = 22.9 Hz, 3H); ^{1}H-NMR α- anomer (400 MHz, D_2O) δ 9.06 (s, 1H), 6.24 (dt, *J* = 17.5, 2.6 Hz, 1H), 4.18 – 4.01 (m, 3H), 3.86 (d, *J* = 13.2 Hz, 1H), 1.57 – 1.23 (m, 3H); ^{19}F{^{1}H}-NMR β- anomer (377 MHz, D_2O) δ -175.88; ^{19}F{^{1}H}-NMR α- anomer (377 MHz, 353K, D_2O) δ -159.76; ^{13}C{^{1}H}-NMR β- anomer (101 MHz, 353K, D_2O) δ 165.9, 164.2, 150.8, 149.2, 104.7, 102.6, 100.8, 90.9, 90.5, 82.5, 71.8, 71.6, 59.5, 16.5, 16.3; ^{13}C{^{1}H}-NMR α- anomer (101 MHz, 353K, D_2O) δ 165.9, 164.2, 150.8, 149.2, 104.7, 102.6, 100.8, 90.9, 90.5, 82.5, 71.8, 71.6, 59.5, 16.5, 16.3; IR v_{max} (neat) β- anomer 3262, 1714, 1674, 1615 cm^{-1}; IR v_{max} (neat) α- anomer 3438, 1718, 1647 cm^{-1}; HRMS (ESI) *m/z*: [M + H - COOH]$^+$ Calcd for $(C_{10}H_{12}FN_2O_7)^+$: 261.0881; Found 261.0881; Anal. Calcd for $C_{11}H_{13}FN_2O_7$.0.5 H_2O: C, 42.18; H, 4.51; N, 8.94; Found: C, 42.25; H, 4.23; N, 8.61.

1-(3,5-Di-*O*-benzoyl-2-deoxy-2,2-difluoro-β-*D*-ribofuranosyl)-3-(2,4-dimethoxybenzyl)-2,4-dioxo-1,2,3,4-tetrahydropyrimidine-5-carboxylic acid (18e)

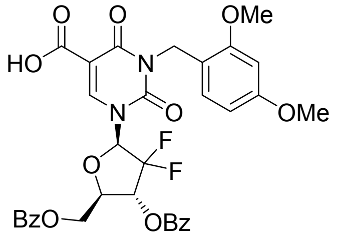

(18e)

2,4-Dimethoxybenzyl isocyanate (**52**) (0.22 mL, 1.35 mmol, 4 equiv) and enamide **15d** (177 mg, 0.333 mmol, 1 equiv) in THF (6 mL) were added with stirring to NaH (60% in oil; 47.0 mg, 1.938 mmol, 6 equiv). The mixture directly loaded onto silica and chromatographed (CH_2Cl_2 : pentane 1 : 1 to MeOH : CH_2Cl_2 : pentane 1 : 5 : 4) to give the nucleoside **18e** (58.0 mg, 0.091 mmol, 27%) as a white solid with the anomeric stereochemistry tentatively assigned as the β-anomer: R_f 0.37 (MeOH : CH_2Cl_2 1 : 24); m.p. (Et_2O) 99.7-101.9 °C; $[\boldsymbol{\alpha}]_D^{26.0}$= -44.7 (*c* 1.0, $CHCl_3$); ^{1}H-NMR (400 MHz, $CDCl_3$) δ 12.46 (s, 1H), 8.65 (s, 1H), 8.18 – 7.95 (m, 4H), 7.69 – 7.53 (m, 2H), 7.47 (dt, *J* = 11.5, 7.6 Hz, 4H), 7.18 – 7.05 (m, 1H), 6.44 (tt, *J* = 5.8, 4.1, 3.3 Hz, 3H), 5.66 (d, *J* = 11.2 Hz, 1H), 5.17 (dd, *J* = 107.7, 14.4 Hz, 2H), 4.87 (dd, *J* = 12.6, 4.3 Hz, 1H), 4.81 – 4.57 (m, 2H), 3.78 (d, *J* = 2.2 Hz, 6H); ^{19}F{^{1}H}-NMR (377 MHz, $CDCl_3$) δ -114.05, -114.70; ^{13}C{^{1}H}-NMR (101 MHz, $CDCl_3$) δ 166.1, 164.6, 164.4, 162.4, 160.9, 158.5, 148.9, 134.3, 133.7, 130.4, 130.1, 129.9, 128.8, 128.7, 127.7, 114.7, 104.1, 103.1, 98.7, 79.0, 71.1,

62.2, 55.5, 55.4, 41.5; IR v_{max} (neat) 1725, 1684, 1654, 1613, 1267, 710 cm^{-1}; HRMS (ESI) *m/z*: $[M + H]^+$ Calcd for $(C_{33}H_{29}F_2N_2O_{11})^+$: 667.1734; Found 667.1768.

1-(2-Deoxy-2,2-difluoro-*D*-ribofuranosyl)-2,4-dioxo-1,2,3,4-tetrahydropyrimidine-5-carboxylic acid (19e)

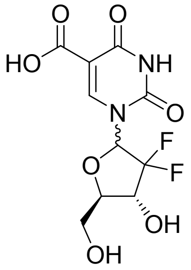

(19e)

2,4-Dimethoxybenzyl isocyanate (**52**) (0.11 mL, 4.52 mmol, 4 equiv) and enamide **15d** (600 mg, 1.13 mmol, 1 equiv) in THF (10 mL) were added with stirring to NaH (60% in oil; 752 mg, 4.52 mmol, 4 equiv). After stirring overnight, reaction was quenched with HCO_2H (2 mL), and the mixture was filtered and concentrated *in vacuo*. CF_3CO_2H (3 mL) was added with stirring to the residue in PhMe (10 mL). After stirring overnight at 75 °C, the solution was co-evaporated with PhMe to dryness. K_2CO_3 (468 mg, 3.38 mmol, 3 equiv) was added with stirring to the residue in MeOH (20 mL). After stirring overnight, the solution was quenched with Dowex 50 (pH of 6-7), filtered and concentrated *in vacuo*. Purification of the residue by reverse phase C18 silica (H_2O/MeCN with 0.1% HCO_2H in MeCN : H_2O 0 : 1 to 3 : 7) gave nucleoside carboxylic acid **19e** (0.139 g, 0.450 mmol, 40%) as a white solid: R_f 0.48 (MeOH : CH_2Cl_2 1 : 4 with 0.1% HCO_2H); m.p. β 199.67-202.2 °C (H_2O); m.p. α 206.45-208.8 °C; α $[\alpha]_D^{26.1} =$ +7.5° (*c* 0.5, H_2O); β $[\alpha]_D^{26.1} =$ -38.5° (*c* 1.0, H_2O); ^{1}H-NMR α (400 MHz, D_2O) δ 8.09 (s, 1H), 6.31 – 6.19 (m, 1H), 4.46 (q, *J* = 10.4, 9.8 Hz, 1H), 4.41 – 4.30 (m, 1H), 3.87 – 3.67 (m, 2H); ^{1}H-NMR β (400 MHz, D_2O) δ 8.20 (s, 1H), 6.13 (t, *J* = 7.7 Hz, 1H), 4.35 (td, *J* = 11.7, 8.2 Hz, 1H), 4.03 – 3.88 (m, 2H), 3.78 (dd, *J* = 13.2, 4.2 Hz, 1H); ^{19}F{^{1}H}-NMR β (377 MHz, D_2O) δ -75.64; ^{19}F{^{1}H}-NMR α (377 MHz, D_2O) δ -75.65; ^{13}C{^{1}H}-NMR β (101 MHz, D_2O) δ 169.2, 162.7, 151.0, 144.1, 124.7, 122.1, 119.5, 111.8, 84.5, 84.2, 84.1, 83.8, 80.6, 80.5, 69.2, 69.0, 68.7, 59.1; ^{13}C{^{1}H}-NMR α (101 MHz, D_2O) δ 169.3, 162.9, 151.3, 144.0, 124.8, 122.2, 122.2, 119.6, 111.4, 85.0, 84.8, 84.6, 84.4, 83.7, 83.6, 69.9, 69.8, 69.7, 69.5, 59.7; IR v_{max} (neat) 1725, 1654, 1451, 1267 cm^{-1}; HRMS (ESI) *m/z*: $[M - H]^-$ Calcd for $(C_{10}H_9F_2N_2O_7)^-$: 307.0383; Found 307.0389.

VI. Bromo-decarboxylation Reactions of Uridine-5-carboxylic Acid Derivatives

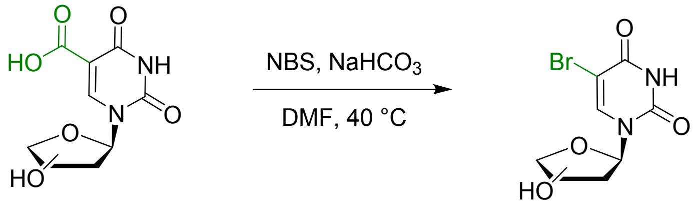

1-(2-Deoxy-β-*D*-ribofuranosyl)-5-bromo-2,4-dioxo-1,2,3,4-tetrahydropyrimidine (20a)

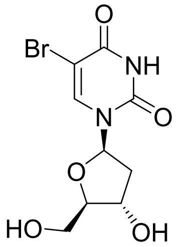

(20a)

NBS (42.0 mg, 0.238 mmol, 1.3 equiv) and $NaHCO_3$ (46.0 mg, 0.551 mmol, 3 equiv) were added with stirring to nucleoside carboxylic acid **19a** (50.0 mg, 0.183 mmol, 1 equiv) in DMF (1 mL). After stirring overnight at 50 °C, reaction was quenched with Dowex 50 (pH 6-7), and the mixture was filtered, and concentrated *in vacuo*. Purification by preparative TLC (MeOH : CH_2Cl_2 1 : 9) gave bromo-nucleoside **20a** (27.0 g, 0.088 mmol, 48%) as a white solid: R_f 0.64 (MeOH : CH_2Cl_2 1 : 5); m.p. 173.1- 174.9 °C (MeOH); $[\alpha]_D^{26.3}$ = +11.8° (*c* 1.0, MeOH); ^{1}H-NMR (400 MHz, D_2O) δ 8.11 (d, *J* = 2.5 Hz, 1H), 6.14 (t, *J* = 6.5 Hz, 1H), 4.35 (dt, *J* = 6.6, 4.3 Hz, 1H), 3.93 (q, *J* = 4.1 Hz, 1H), 3.84 – 3.60 (m, 2H), 2.38 – 2.17 (m, 2H); ^{13}C{^{1}H}-NMR (126 MHz, D_2O) δ 164.0, 152.6, 141.0, 96.8, 86.6, 85.7, 70.1, 60.9, 39.0; IR v_{max} (neat) 3371, 1675, 1448, 1270 cm^{-1}; HRMS (APCI) *m/z*: $[M - H]^-$ Calcd for $(C_9H_{10}BrN_2O_5)^-$: 306.9758; Found 306.9758.

1-(β-*D*-Ribofuranosyl)-5-bromo-2,4-dioxo-1,2,3,4-tetrahydropyrimidine (20b)

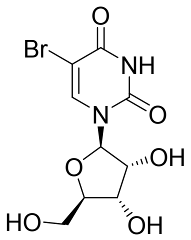

(20b)

NBS (19.0 mg, 0.104 mmol, 1 equiv) and $NaHCO_3$ (26.0 mg, 0.0312 mmol, 3 equiv) were added with stirring to nucleoside carboxylic acid **19b** (30.0 mg, 0.104 mmol, 1 equiv) in DMF (1 mL). After stirring overnight at 50 °C, reaction was quenched with Dowex 50 (pH 6-7), and the mixture was filtered, and concentrated *in vacuo*. Purification by preparative TLC (MeOH :

CH_2Cl_2 1 : 9) gave bromo-nucleoside **20b** (15.0 mg, 0.046 mmol, 45%) as a white solid: R_f 0.27 (MeOH : CH_2Cl_2 1 : 9); m.p. 192.4-193.5 (MeOH); $[\alpha]_D^{25.8} =$ +6.5° (*c* 0.5, MeOH); ^{1}H-NMR (400 MHz, D_2O) δ 8.53 (s, 1H), 6.10 (d, *J* = 4.2 Hz, 1H), 4.42 (t, *J* = 4.4 Hz, 1H), 4.34 – 4.16 (m, 2H), 3.87 – 3.56 (m, 2H); $^{13}C\{^1H\}$-NMR (101 MHz, D_2O) δ 163.2, 151.7, 142.2, 95.4, 86.9, 83.2, 70.6, 69.9, 60.7; IR v_{max} (neat) 3418, 1699, 1271 cm^{-1} ; HRMS (ESI) *m/z*: $[M - H]^-$ Calcd for $(C_9H_{10}BrN_2O_6)^-$: 320.9728; Found 320.9720.

1-(2-Deoxy-2-fluoro-β-*D*-arabinofuranosyl)-5-bromo-2,4-dioxo-1,2,3,4-tetrahydropyrimidine (20c)

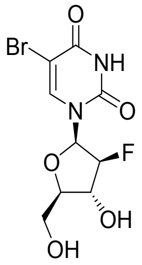

(20c)

NBS (31.0 mg, 0.172 mmol, 1 equiv) and $NaHCO_3$ (43.0 mg, 0.516 mmol, 3 equiv) were added with stirring to nucleoside carboxylic acid **19c** (50.0 mg, 0.172 mmol, 1 equiv) in DMF (1 mL). After stirring overnight at 50 °C, reaction was quenched with Dowex 50 (pH 6-7), and the mixture was filtered, and concentrated *in vacuo*. Purification by preparative TLC (MeOH : CH_2Cl_2 1 : 9) gave bromo-nucleoside **20c** (29.0 mg, 0.089 mmol, 52%) as a white solid: R_f 0.27 (MeOH : CH_2Cl_2 1 : 9); m.p. 191.2-193.5 °C **(**MeOH); $[\alpha]_D^{26.0} = +46.3°$ (*c* 1.0, MeOH); ^{1}H-NMR (400 MHz, D_2O) δ 8.09 (d, *J* = 1.4 Hz, 1H), 6.13 (dd, *J* = 16.4, 4.0 Hz, 1H), 5.16 – 4.97 (m, 1H), 4.31 (ddd, *J* = 19.2, 5.1, 2.7 Hz, 1H), 3.95 (q, *J* = 4.9 Hz, 1H), 3.82 (dd, *J* = 12.6, 3.6 Hz, 1H), 3.72 (dd, *J* = 12.6, 5.8 Hz, 1H); $^{13}C\{^1H\}$-NMR (101 MHz, D_2O) δ 163.6, 152.1, 141.3, 141.3, 96.5, 95.3, 93.7, 84.0, 83.9, 83.3, 83.3, 73.2, 73.0, 60.3; $^{19}F\{^1H\}$-NMR (377 MHz, D_2O) δ -199.81; IR v_{max} (neat) 3354, 1695, 1675 cm^{-1}; HRMS (ESI) *m/z*: $[M + H]^+$ Calcd for $(C_9H_{11}BrFN_2O_5)^+$: 324.9830; Found 324.9829.

1-(2-Deoxy-2-fluoro-2-methyl-β-*D*-ribofuranosyl)-5-bromo-2,4-dioxo-1,2,3,4-tetrahydropyrimidine (20d)

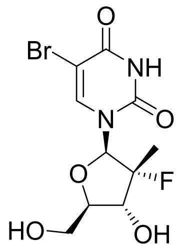

(20d)

NBS (20.0 mg, 0.111 mmol, 1.3 equiv) and $NaHCO_3$ (22.0 mg, 0.256 mmol, 3 equiv) were added with stirring to nucleoside carboxylic acid **19d** (26.0 mg, 0.085 mmol, 1 equiv) in DMF (1 mL). After stirring overnight at 50 °C, reaction was quenched with Dowex 50 (pH 6-7), and the mixture was filtered, and concentrated *in vacuo*. Purification by preparative TLC (MeOH : CH_2Cl_2 1 : 9) gave bromo-nucleoside **20d** (11.0 mg, 0.033 mmol, 39%) as a white solid. Anomeric stereochemistry was tentatively assigned: R_f 0.46 (MeOH : CH_2Cl_2 1 : 9); m.p. 294.5-295 **(**MeOH); $[\alpha]_D^{26.0} =$ -9.3° (*c* 0.5, MeOH); ^{1}H-NMR (500 MHz, D_2O) δ 8.23 (s, 1H), 6.11 (d, *J* = 18.5 Hz, 1H), 4.06 – 3.93 (m, 3H), 3.79 (dd, *J* = 13.3, 2.7 Hz, 1H), 1.30 (d, *J* = 23.2 Hz, 3H); ^{19}F{^{1}H}-NMR (471 MHz, 353K, D_2O) δ -158.84; ^{13}C{^{1}H}-NMR (126 MHz, 353K, D_2O) δ 140.7, 102.5, 101.7, 97.9, 90.6, 90.3, 82.1, 72.0, 71.9, 59.8, 16.6, 16.4; IR ν_{max} (neat) 3384, 1695 cm^{-1}; HRMS (APCI) *m/z*: $[M - H]^-$ Calcd for $(C_{10}H_{13}BrFN_2O_5)^-$: 336.9841; Found 336.9841.

1-(2-Deoxy-2,2-difluoro-β-*D*-ribofuranosyl)-5-bromo-2,4-dioxo-1,2,3,4-tetrahydropyrimidine (20e)

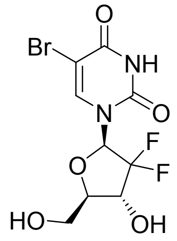

(20e)

NBS (26.0 mg, 0.015 mmol, 1.5 equiv) and $NaHCO_3$ (25.0 mg, 0.29 mmol, 3 equiv) were added with stirring to β-nucleoside carboxylic acid **19e** (30.0 mg, 0.057 mmol, 1 equiv) in DMF (1 mL). After stirring overnight at 50 °C, reaction was quenched with Dowex 50 (pH 6-7), and the mixture was filtered, and concentrated *in vacuo*. Purification by preparative TLC (MeOH : CH_2Cl_2 1 : 9) gave bromo-nucleoside **20e** (11.0 mg, 0.032 mmol, 33%) as a white solid: R_f 0.82 (MeOH : CH_2Cl_2 1 : 5); m.p.145.89-147.13 °C (MeOH); $[\alpha]_D^{26.3} =$ +4.8 ° (*c* 1.0, MeOH); ^{1}H-NMR (400 MHz, D_2O) δ 8.19 (t, *J* = 2.5 Hz, 1H), 6.24 – 6.10 (m, 1H), 4.37 (d, *J* = 11.2 Hz, 1H), 4.11 – 4.03 (m, 1H), 4.03 – 3.81 (m, 2H); ^{19}F{^{1}H}-NMR (377 MHz, D_2O) δ -117.73; ^{13}C{^{1}H}-NMR (101 MHz, D_2O) δ 160.5, 151.3, 140.4, 124.6, 122.0, 119.4, 105.8, 97.3, 84.5, 84.3,

84.1, 83.9, 80.7, 80.6, 69.1, 68.9, 68.6, 59.1; IR v_{max} (neat) 3240, 1668, 1410, 1318, 1201, 1075 cm^{-1}; HRMS (APCI) *m/z*: $[M - H]^-$ Calcd for $(C_9H_8BrF_2N_2O_5)^-$: 340.9590; Found 340.9590.

(1) Lerner, L. M.; Kohn, P. The Preparation of Nucleosides from Allose, Altrose, Gulose, Talose, and Mannose1. *J. Med. Chem.* **1964**, *7* (5), 655–658. https://doi.org/10.1021/jm00335a021.

(2) Floyd, N.; Vijayakrishnan, B.; Koeppe, J. R.; Davis, B. G. Thiyl Glycosylation of Olefinic Proteins: S-Linked Glycoconjugate Synthesis. *Angew. Chem. Int. Ed.* **2009**, *48* (42), 7798–7802. https://doi.org/10.1002/anie.200903135.

(3) A. O'Doherty, G.; J. Beuning, P.; Jo Ondrechen, M.; Ray, D. A Practical Synthesis of Glycinamide Ribonucleotide. *HETEROCYCLES* **2018**, *97* (2), 776. https://doi.org/10.3987/COM-18-S(T)50.

(4) Liu, T.; Tang, J.; Liang, J.; Chen, Y.; Wang, X.; Shen, J.; Zhao, D.; Xiong, B.; Cen, J.-D.; Chen, Y.-L. Stereoselective N-Glycosylation with N4-Acyl Cytosines and Efficient Synthesis of Gemcitabine. *Tetrahedron* **2019**, *75* (9), 1203–1213. https://doi.org/10.1016/j.tet.2019.01.027.

(5) Hertel, L. W.; Jones, C. D.; Kroin, J. S.; Mabry, T. E. Anomeric Fluororibosyl Amines. US5594155A, January 14, 1997.

(6) Chun, B.-K.; Du, J.; Rachakonda, S.; Ross, B.; Sofia, M. J.; PAMULAPATI, G. R.; Chang, W.; Zhang, H.-R.; Nagarathnam, D. Synthesis of Purine Nucleosides. US9045520B2, June 2, 2015.

(7) Yang, W.; Peng, Y.; Wang, J.; Song, C.; Yu, W.; Zhou, Y.; Jiang, J.; Wang, Q.; Wu, J.; Chang, J. Design, Synthesis, and Biological Evaluation of Novel 2′-Deoxy-2′-Fluoro-2′-C-Methyl 8-Azanebularine Derivatives as Potent Anti-HBV Agents. *Bioorg. & Med. Chem. Lett.* **2019**, *29* (11), 1291–1297. https://doi.org/10.1016/j.bmcl.2019.04.005.

(8) Liu, Y.; Peng, Y.; Lu, J.; Wang, J.; Ma, H.; Song, C.; Liu, B.; Qiao, Y.; Yu, W.; Wu, J.; Chang, J. Design, Synthesis, and Biological Evaluation of New 1,2,3-Triazolo-2′-Deoxy-2′-Fluoro- 4′-Azido Nucleoside Derivatives as Potent Anti-HBV Agents. *Eur. J. Med. Chem.* **2018**, *143*, 137–149. https://doi.org/10.1016/j.ejmech.2017.11.028.

(9) Štimac, A.; Kobe, J. Stereoselective Synthesis of 1,2-Cis- and 2-Deoxyglycofuranosyl Azides from Glycosyl Halides. *Carbohydr. Res.* **2000**, *329* (2), 317–324. https://doi.org/10.1016/S0008-6215(00)00186-5.

(10) Hametner, C.; Černuchová, P.; Milata, V.; Vo-Thanh, G.; Loupy, A. NMR Spectroscopic Data of Some 1-Alkoxy-2,2-Di(Carbonyl, Carboxyl, Cyano)-Substituted Ethylenes. *Magn. Reson. Chem.* **2005**, *43* (2), 171–173. https://doi.org/10.1002/mrc.1522.

(11) Briehl, H.; Lukosch, A.; Wentrup, C. Reactive Nitrogenous Molecules from Meldrum's Acid Derivatives, Pyrrole-2,3-Diones, and Isoxazolones. *J. Org. Chem.* **1984**, *49* (15), 2772–2779. https://doi.org/10.1021/jo00189a025.

(12) Gordon, H. J.; Martin, J. C.; McNab, H. The Formation of Enaminoenaminones from N-Alkylaminomethylene Derivatives of Meldrum's Acid. *J. Chem. Soc., Perkin Trans. 1* **1984**, No. 0, 2129–2132. https://doi.org/10.1039/P19840002129.

(13) Berthod, T.; Pétillot, Y.; Guy, A.; Cadet, J.; Molko, D. Synthesis of Oligonucleotides Containing 5-Carboxy-2‘-Deoxyuridine at Defined Sites. *J. Org. Chem.* **1996**, *61* (17), 6075–6078. https://doi.org/10.1021/jo960614f.
