# Supplementary material for: Pyrimidine Nucleosides Syntheses by Late-Stage Base Heterocyclization Reactions
Source: Org Lett. 2022 Nov 4;24(49):8931–5. doi: 10.1021/acs.orglett.2c03152 (PMC9764413; doi:10.1021/acs.orglett.2c03152)

Pyrimidine Nucleosides Syntheses by Late-Stage Base Heterocyclization Reactions.

Elfie S. Cavalli, Thomas Mies, Henry S. Rzepa, Andrew J. P. White, Philip J. Parsons, Anthony G.M. Barrett*

Department of Chemistry, Imperial College, London, W12 0BZ, England

Supporting Information (SI)

Figure S. 1 – ^{1}H-NMR Spectrum (400 MHz, $CDCl_3$) - 2,3,4,6-Tetra-*O*-acetyl-α-*D*-mannopyranosyl acetate – **22**

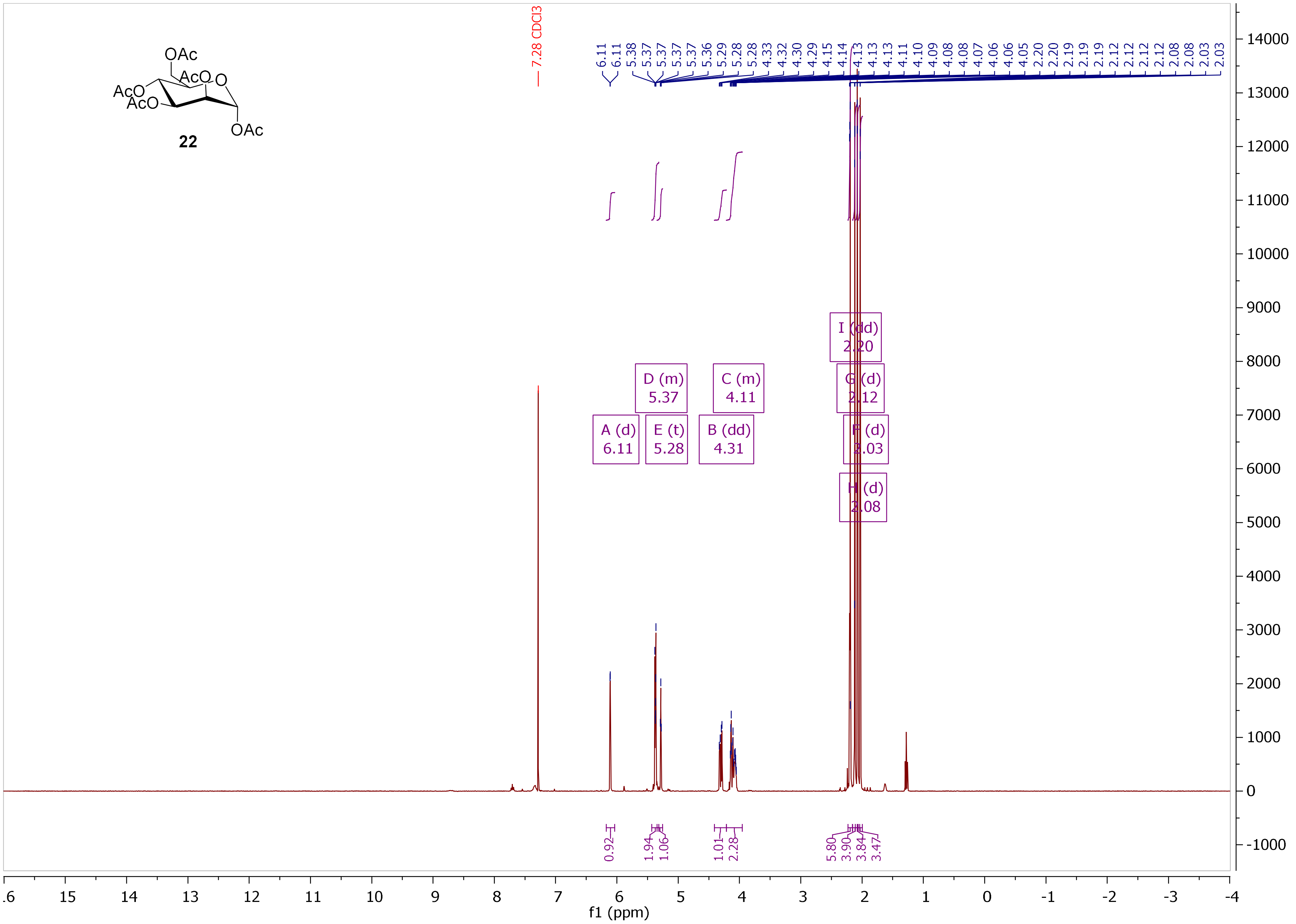

Figure S. 2 - ^{13}C NMR Spectra (101 MHz, $CDCl_3$)- 2,3,4,6-Tetra-*O*-acetyl-α-*D*-mannopyranosyl acetate – **22**

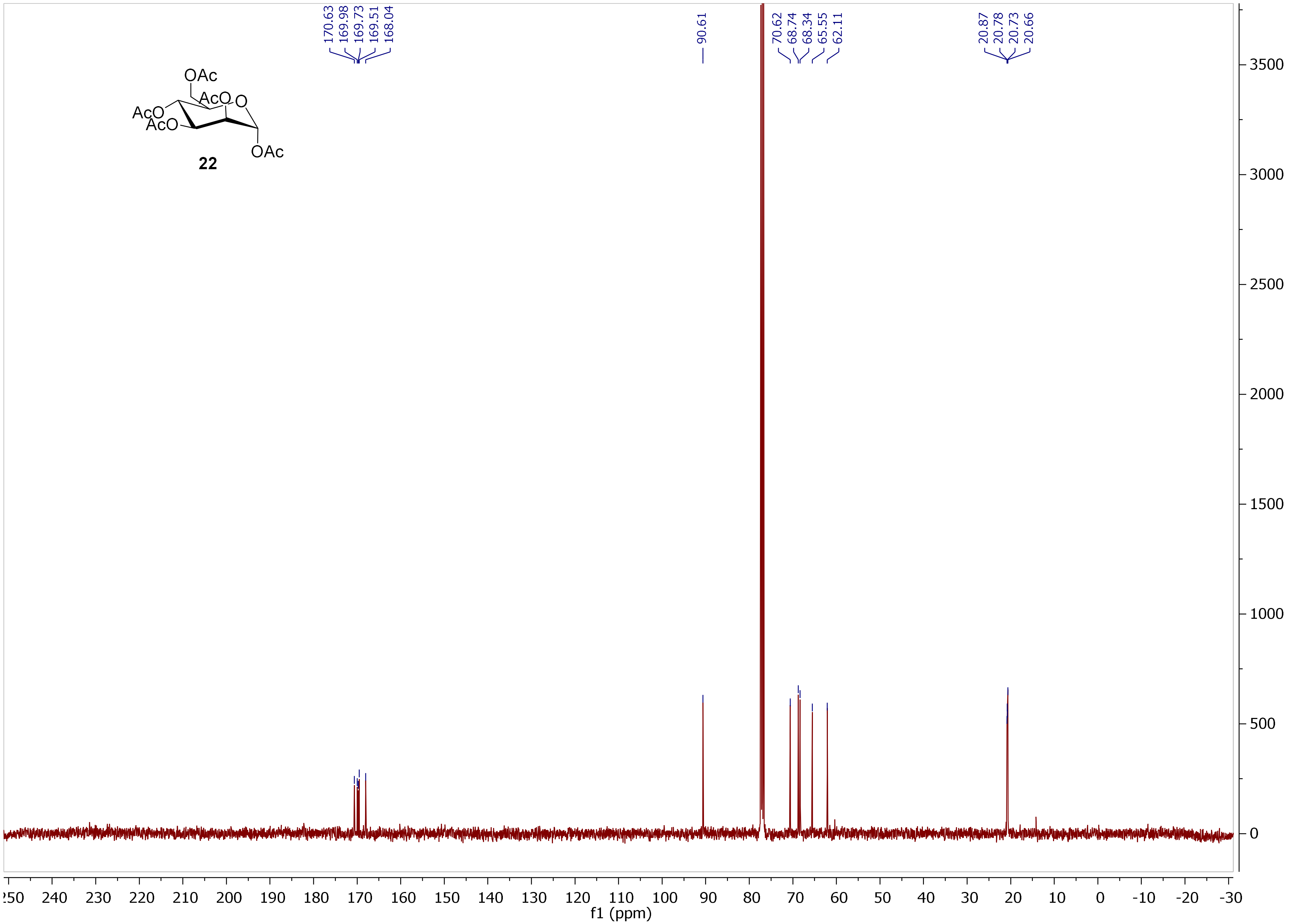

Figure S. 3 - ^{1}H-NMR Spectrum (400 MHz, $CDCl_3$) - 2,3,4,6-Tetra-*O*-acetyl-α-*D*-mannopyranosyl bromide – **23**

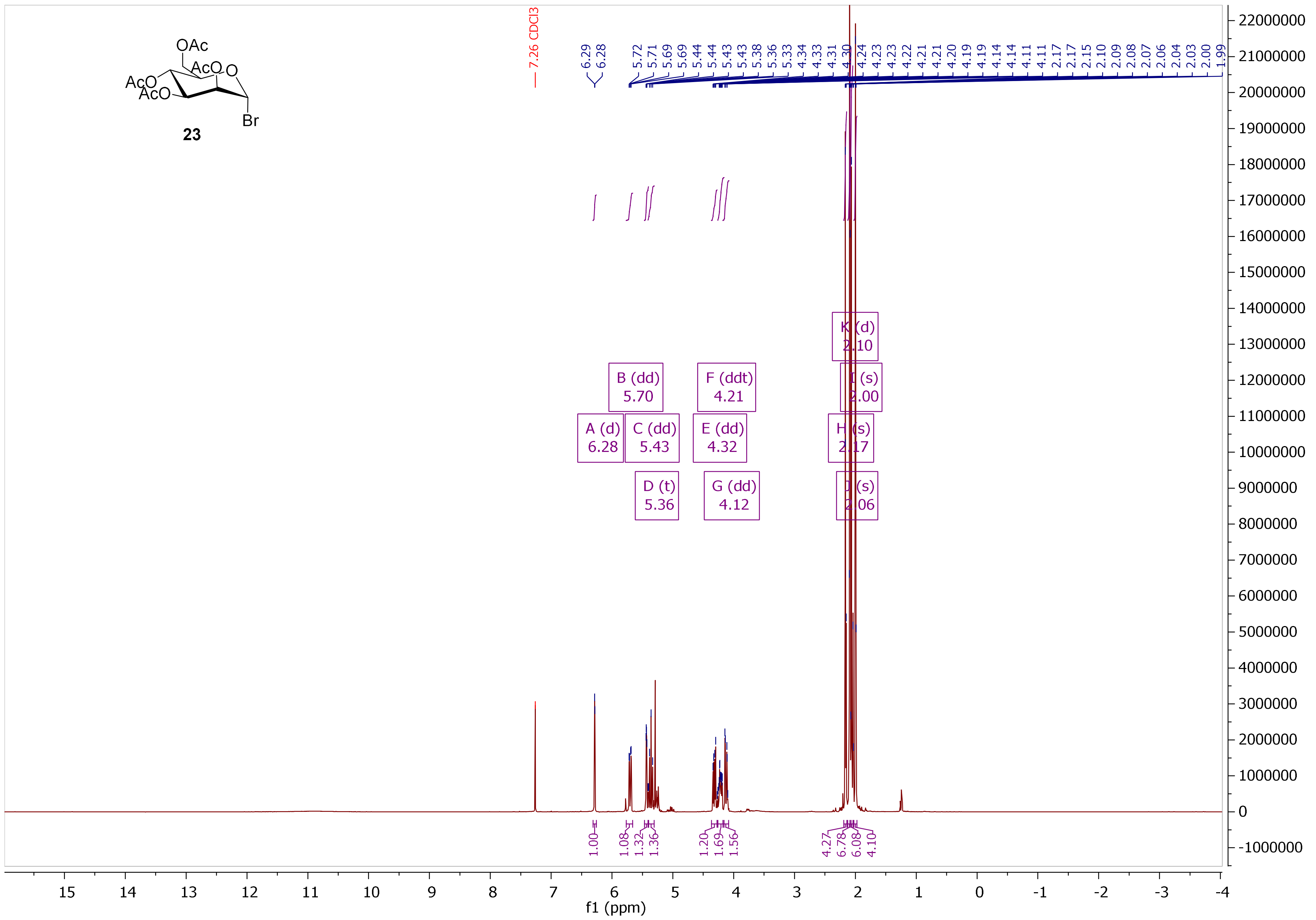

Figure S. 4 - ^{13}C NMR Spectra (101 MHz, $CDCl_3$) - 2,3,4,6-Tetra-*O*-acetyl-α-*D*-mannopyranosyl bromide – **23**

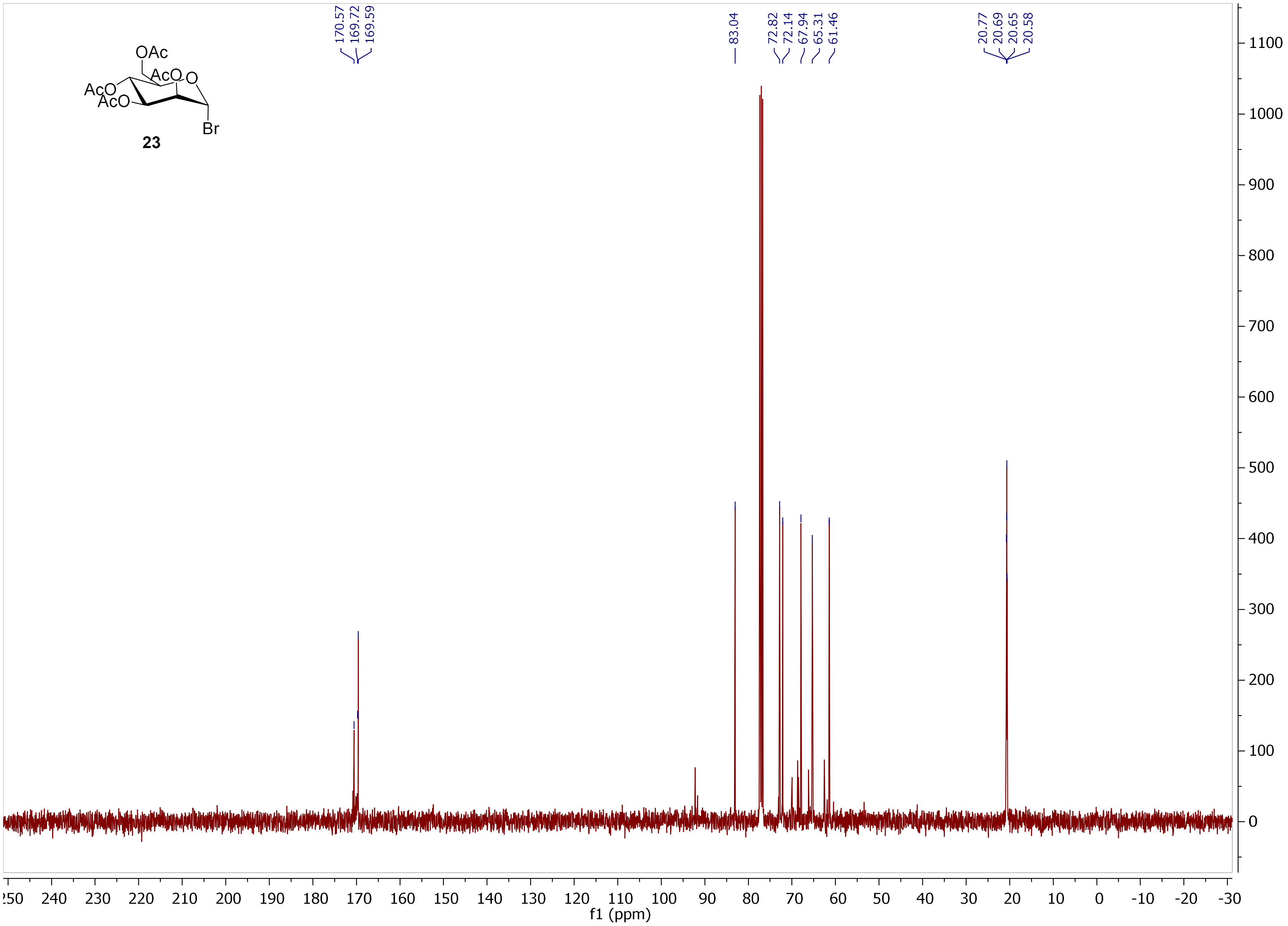

Figure S. 5 ^{1}H-NMR Spectrum (400 MHz, $CDCl_3$) - 2,3,4,6-Tetra-*O*-acetyl-α-*D*-mannopyranosyl isonitrile – **24**

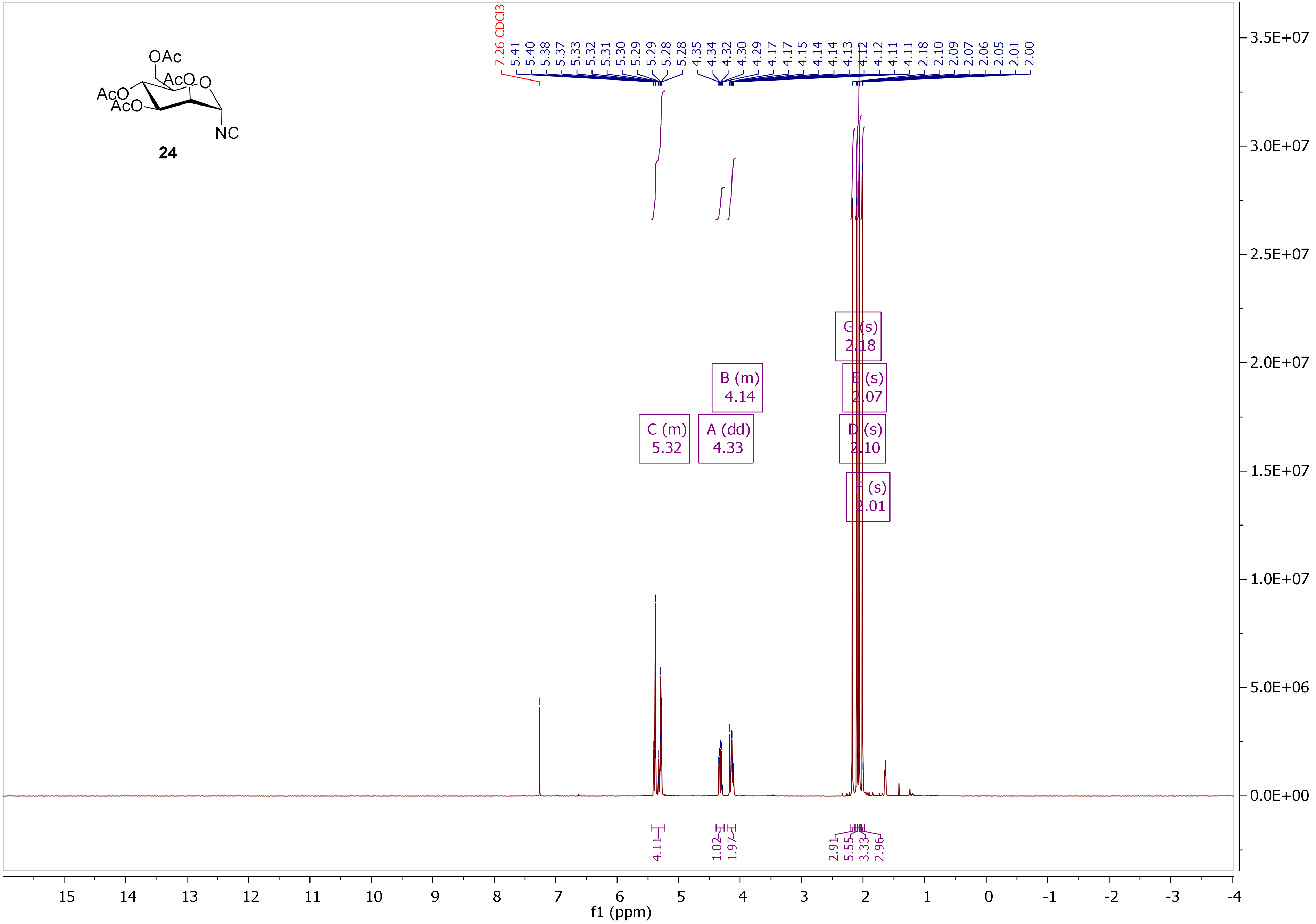

Figure S. 6 - ^{13}C NMR Spectra (101 MHz, $CDCl_3$) - 2,3,4,6-Tetra-*O*-acetyl-α-*D*-mannopyranosyl isonitrile – **24**

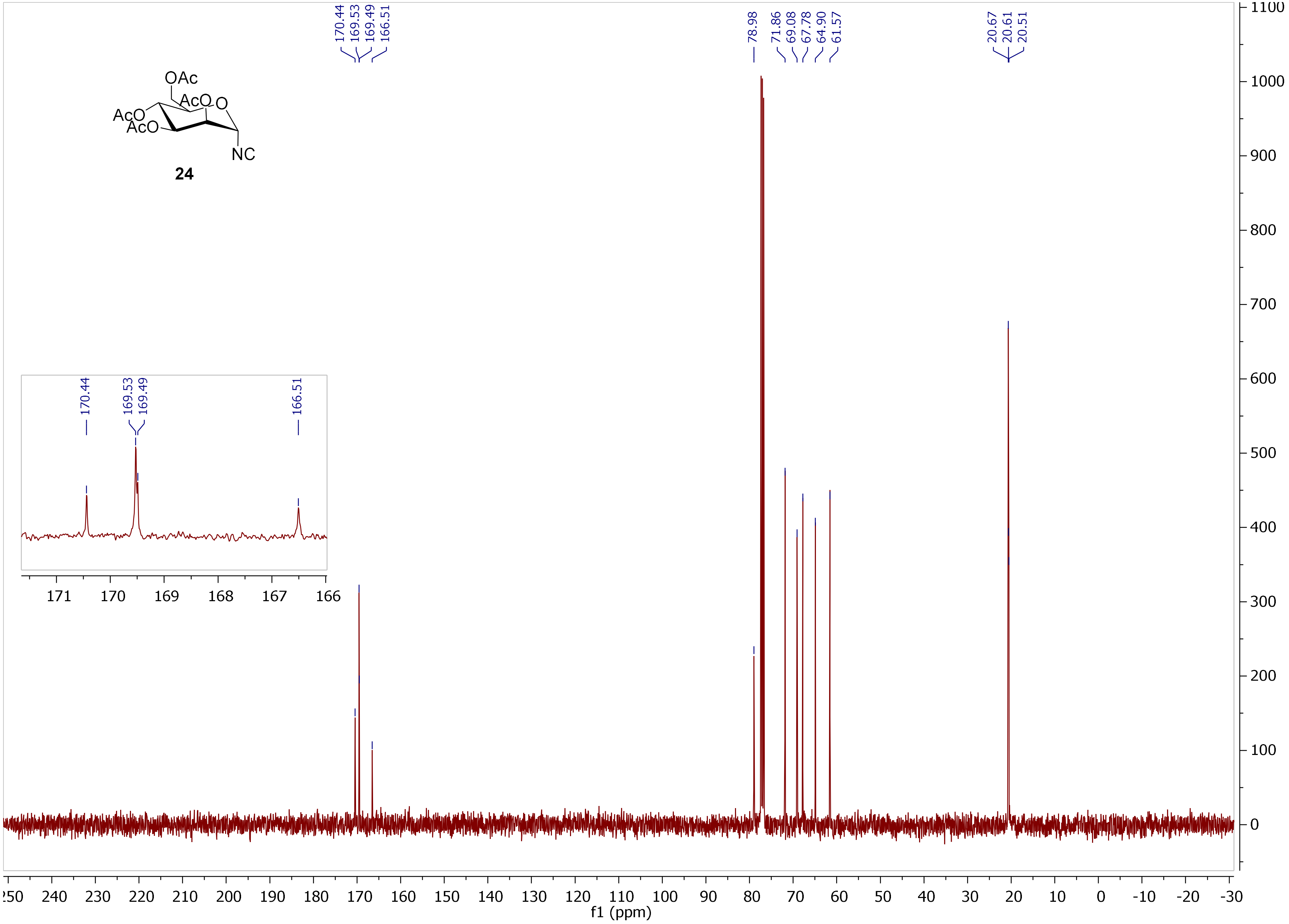

Figure S. 7. - ^{1}H-NMR Spectrum (400 MHz, $CDCl_3$) - 5-*O*-Acetyl-2,3-di-*O*-iso-propylidene-β-*D*-ribofuranosyl acetate – **26**

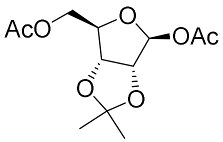

26

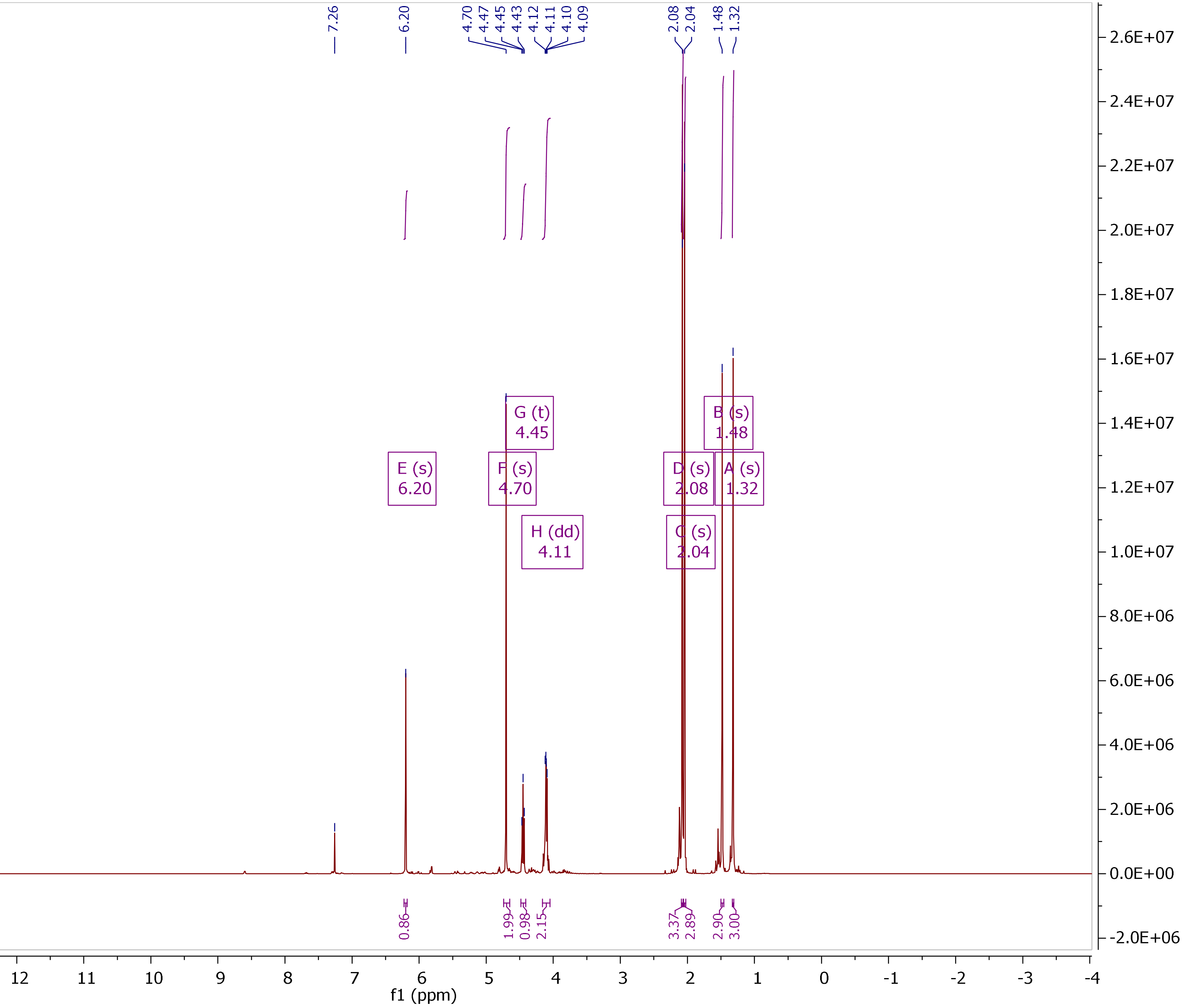

Figure S. 8 - ^{13}C NMR Spectra (101 MHz, $CDCl_3$) - 5-*O*-Acetyl-2,3-di-*O*-iso-propylidene-β-*D*-ribofuranosyl acetate –**26**

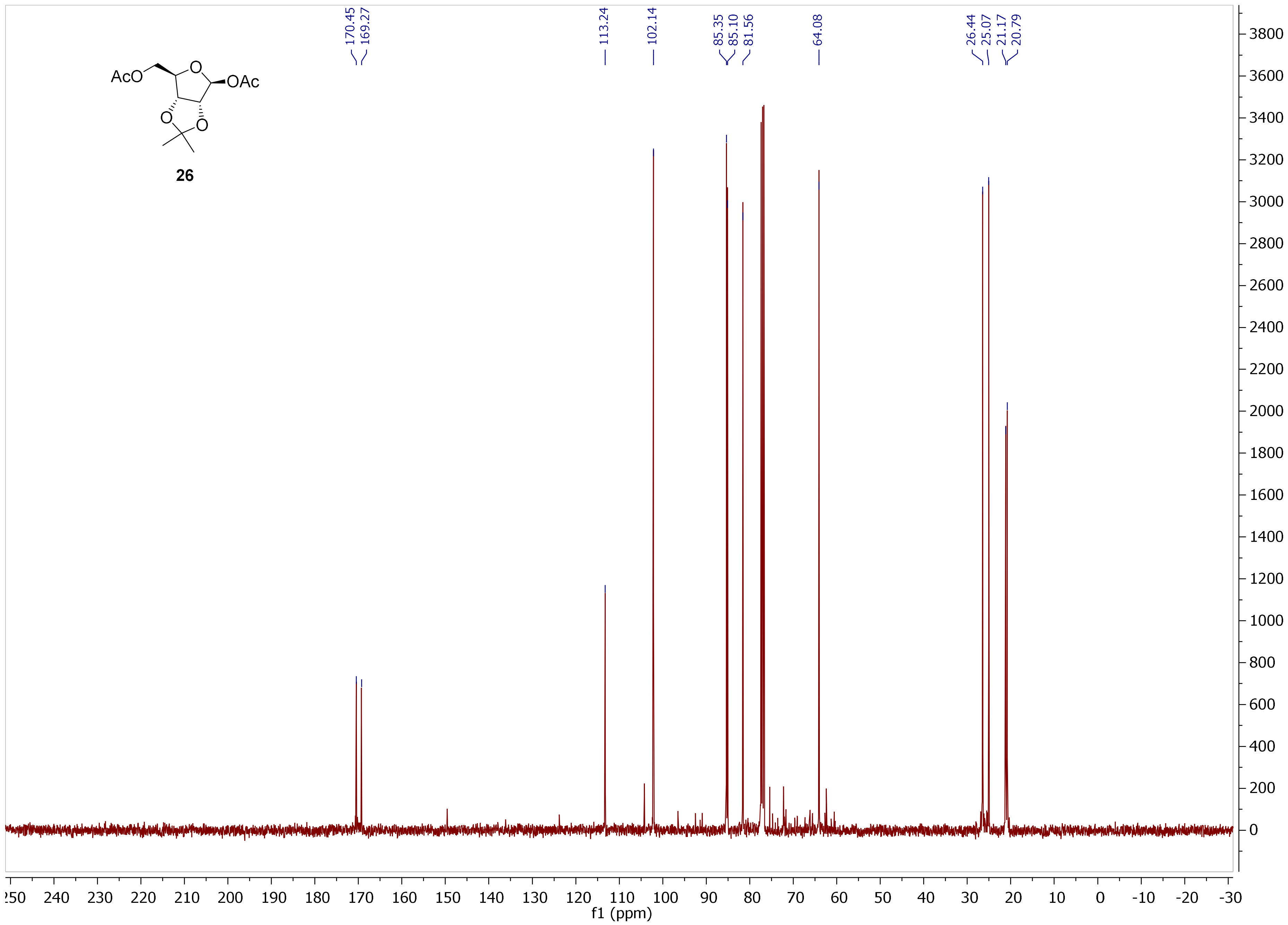

Figure S. 9 - ^{1}H-NMR Spectrum (400 MHz, $CDCl_3$) - 5-*O*-Acetyl-2,3-di-*O*-iso-propylidene-β-*D*-ribofuranosyl isontrile –**28**

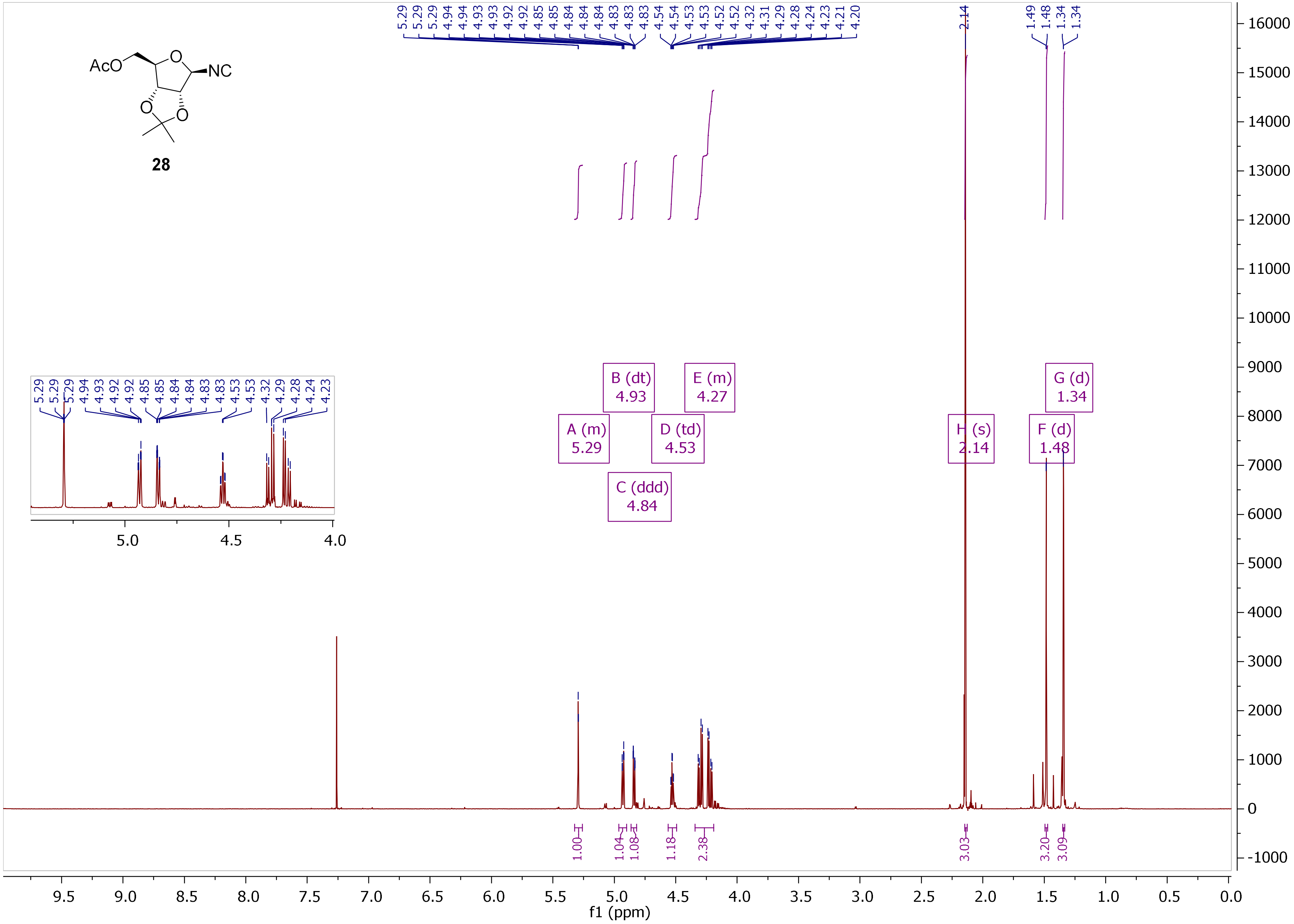

Figure S. 10 - ^{13}C NMR Spectra (101 MHz, $CDCl_3$) - 5-*O*-Acetyl-2,3-di-*O*-iso-propylidene-β-*D*-ribofuranosyl isonitrile –**28**

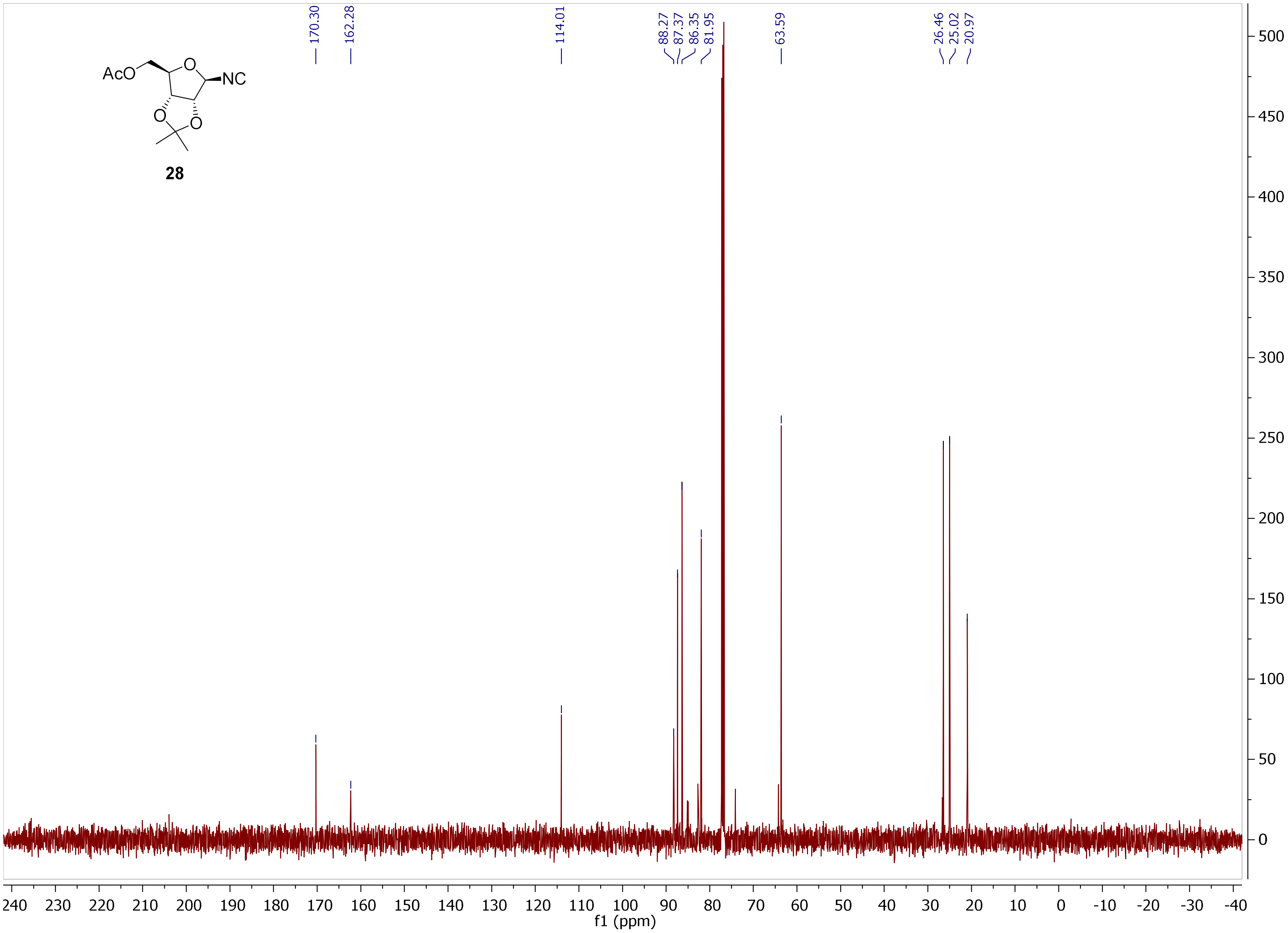

Figure S. 11 - ^{1}H-NMR Spectrum (400 MHz, $CDCl_3$) - 3,5-di-*O*-benzoyl-2-deoxy-2,2-difluoro-*D*-ribofuranosyl iodide – **31**

(31)

α + β

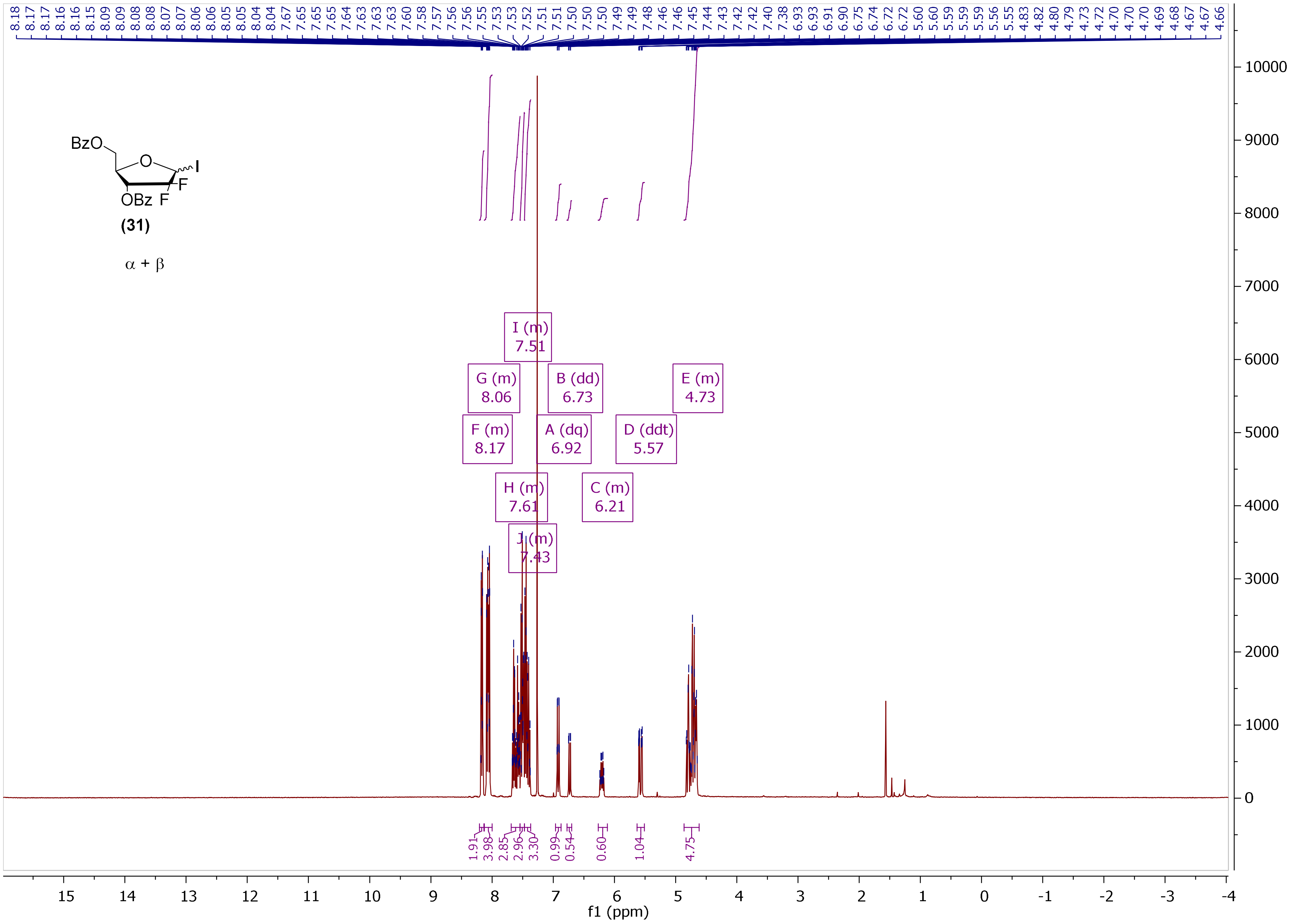

Figure S. 12 – ^{19}F NMR Spectra (377 MHz, $CDCl_3$) - 3,5-di-*O*-benzoyl-2-deoxy-2,2-difluoro-*D*-ribofuranosyl iodide – **31**

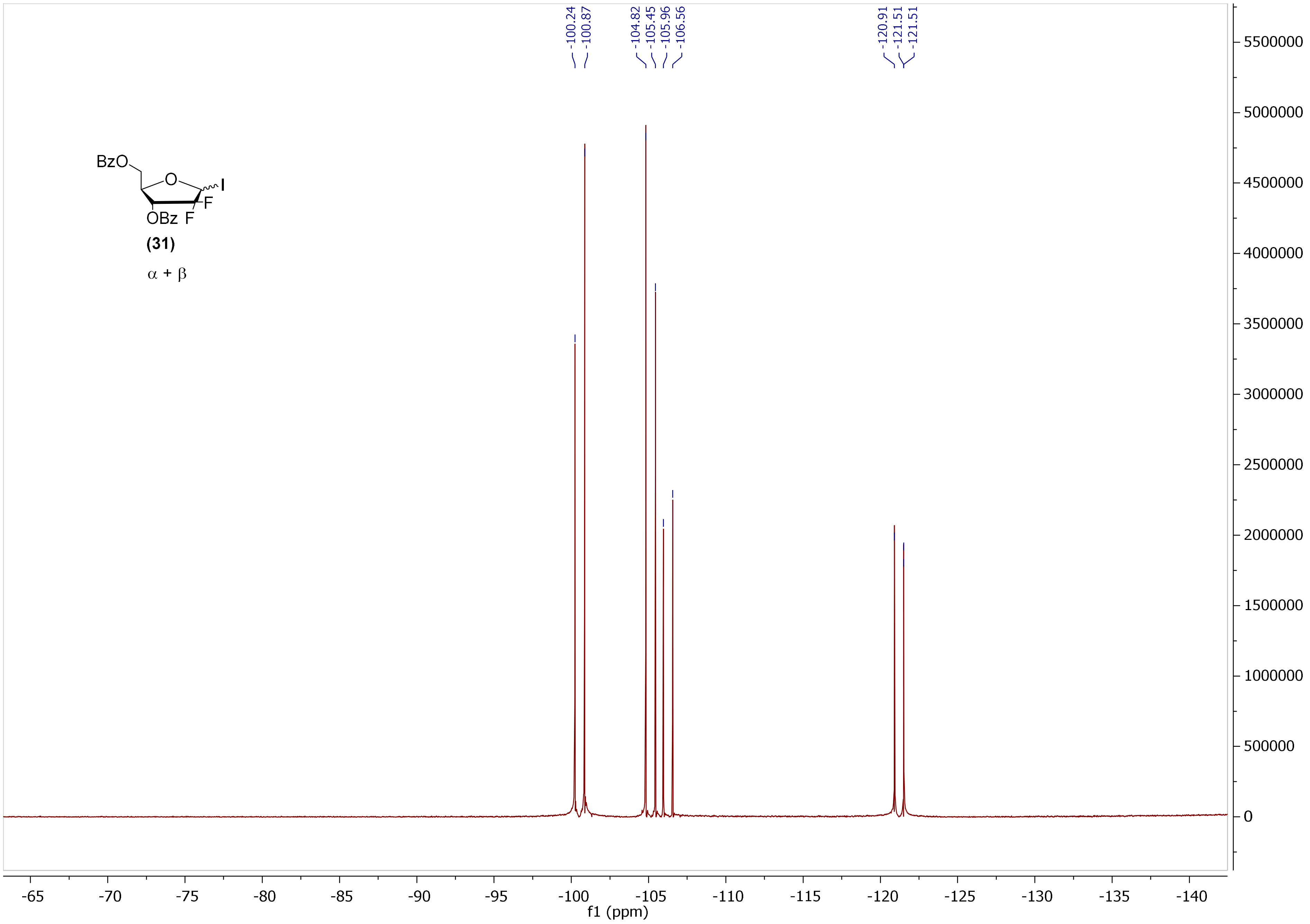

Figure S. 13 - ^{13}C NMR Spectra (101 MHz, $CDCl_3$) - 3,5-di-*O*-benzoyl-2-deoxy-2,2-difluoro-*D*-ribofuranosyl iodide – **31**

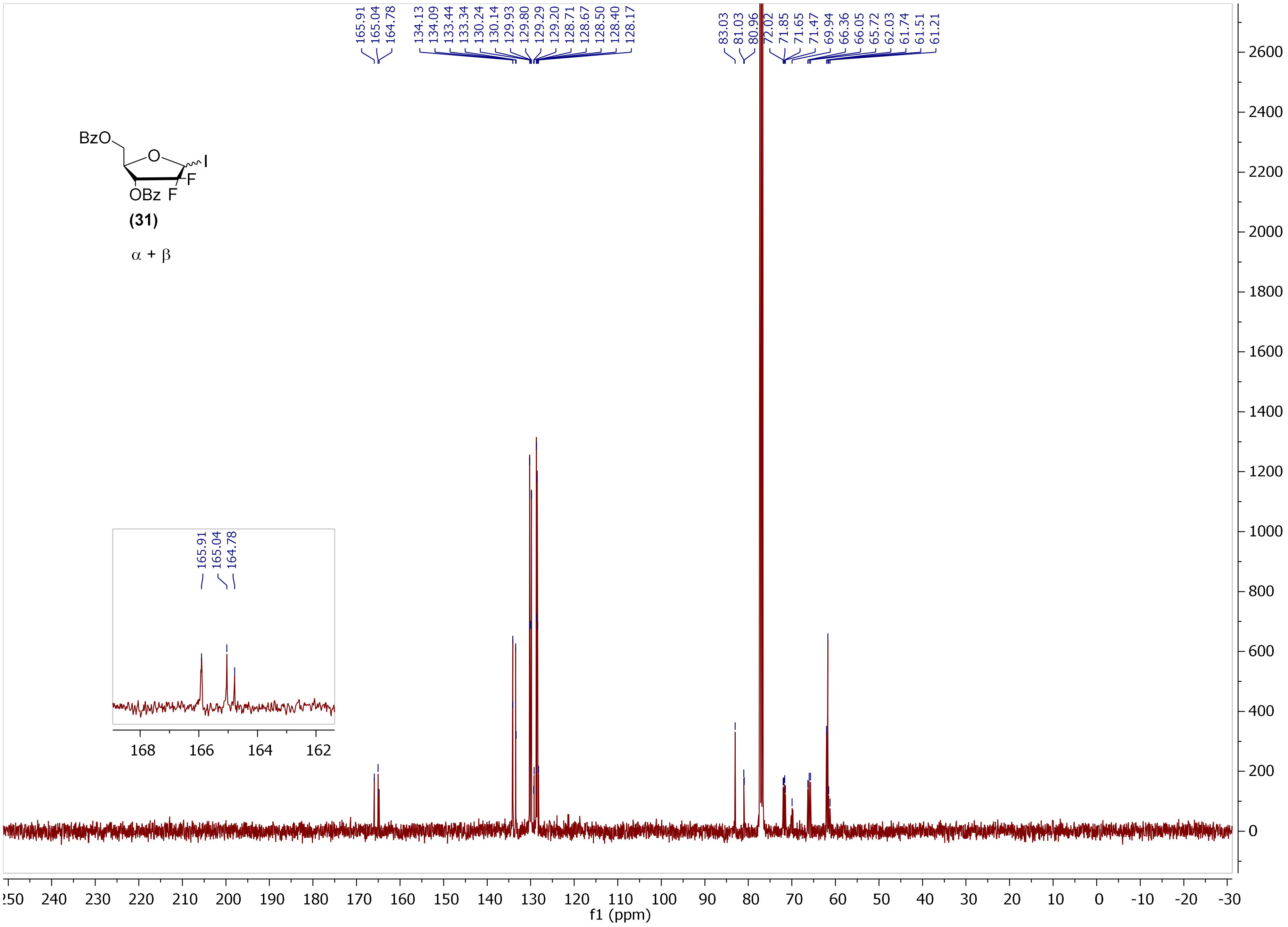

Figure S. 14 - ^{1}H-NMR Spectrum (400 MHz, $CDCl_3$) - 3,5-di-*O*-benzoyl-2-deoxy-2,2-difluoro-β-*D*-ribofuranosyl azide – **32**

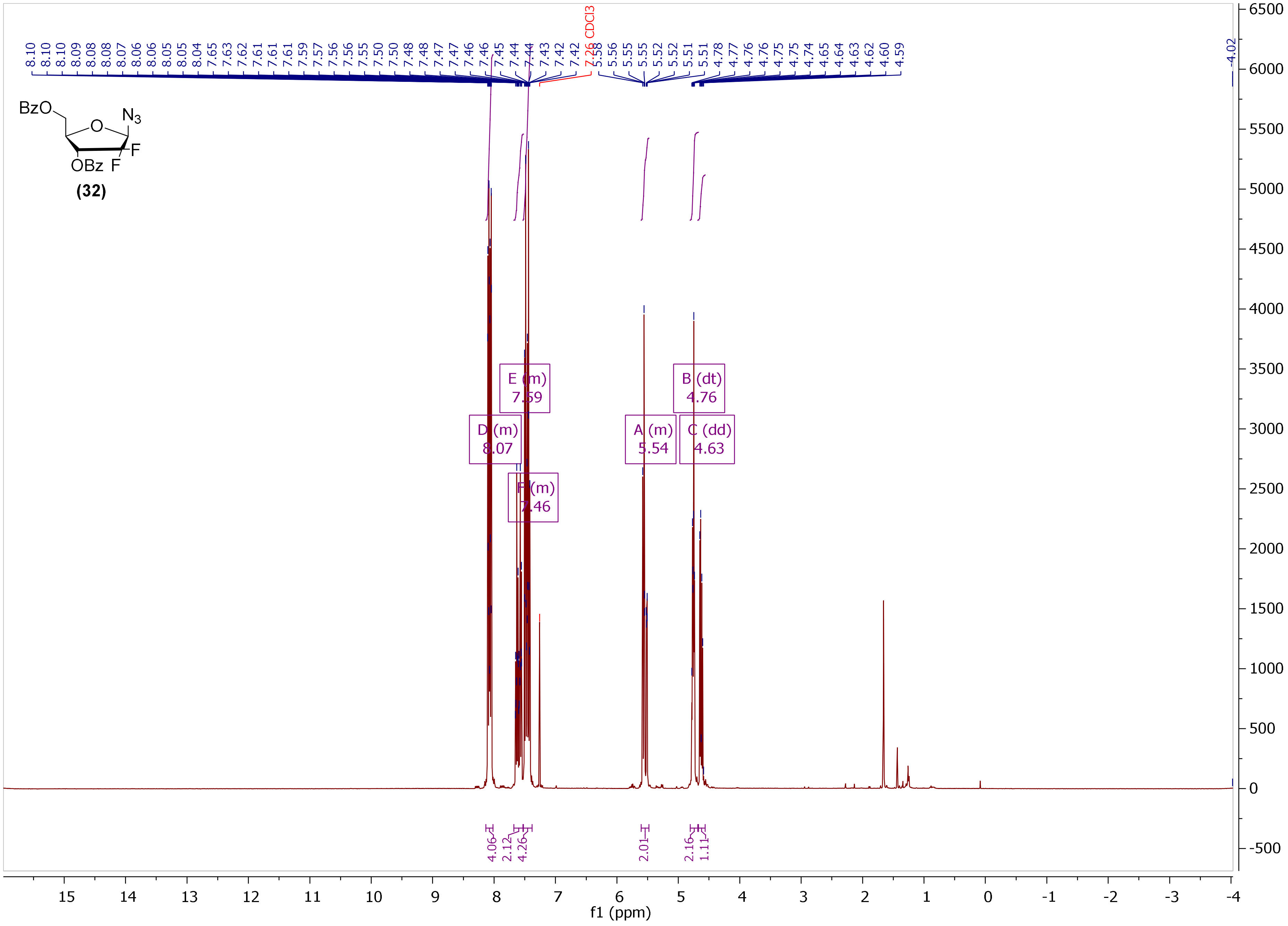

Figure S. 15 - ^{1}H-NMR Spectrum (400 MHz, $CDCl_3$) - 3,5-di-*O*-benzoyl-2-deoxy-2,2-difluoro-α-*D*-ribofuranosyl azide – (1-epi-**32**)

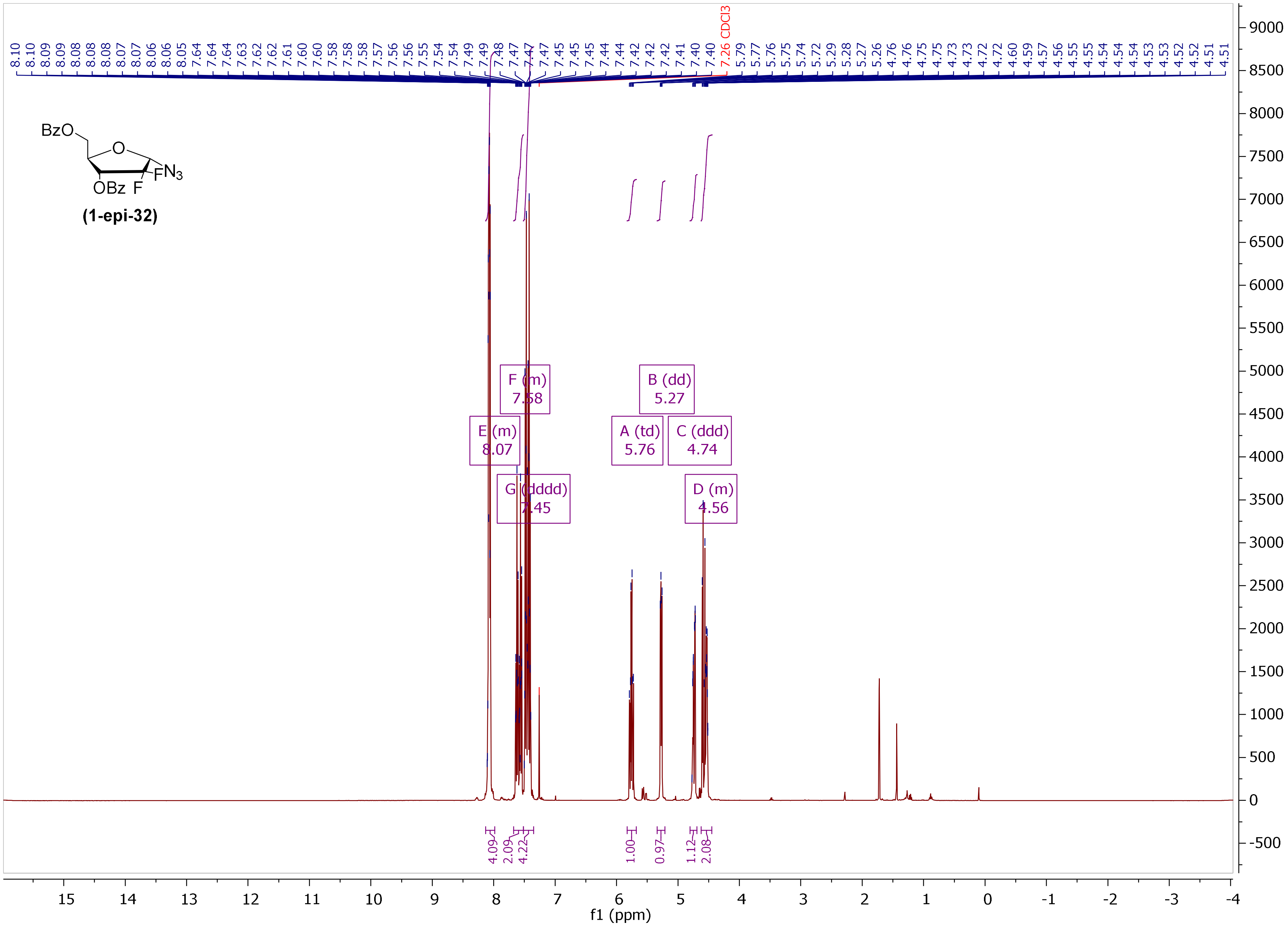

Figure S. 16 - ^{19}F NMR Spectra (377 MHz, $CDCl_3$) - 3,5-di-*O*-benzoyl-2-deoxy-2,2-difluoro-α-*D*-ribofuranosyl azide – (1-epi-**32**)

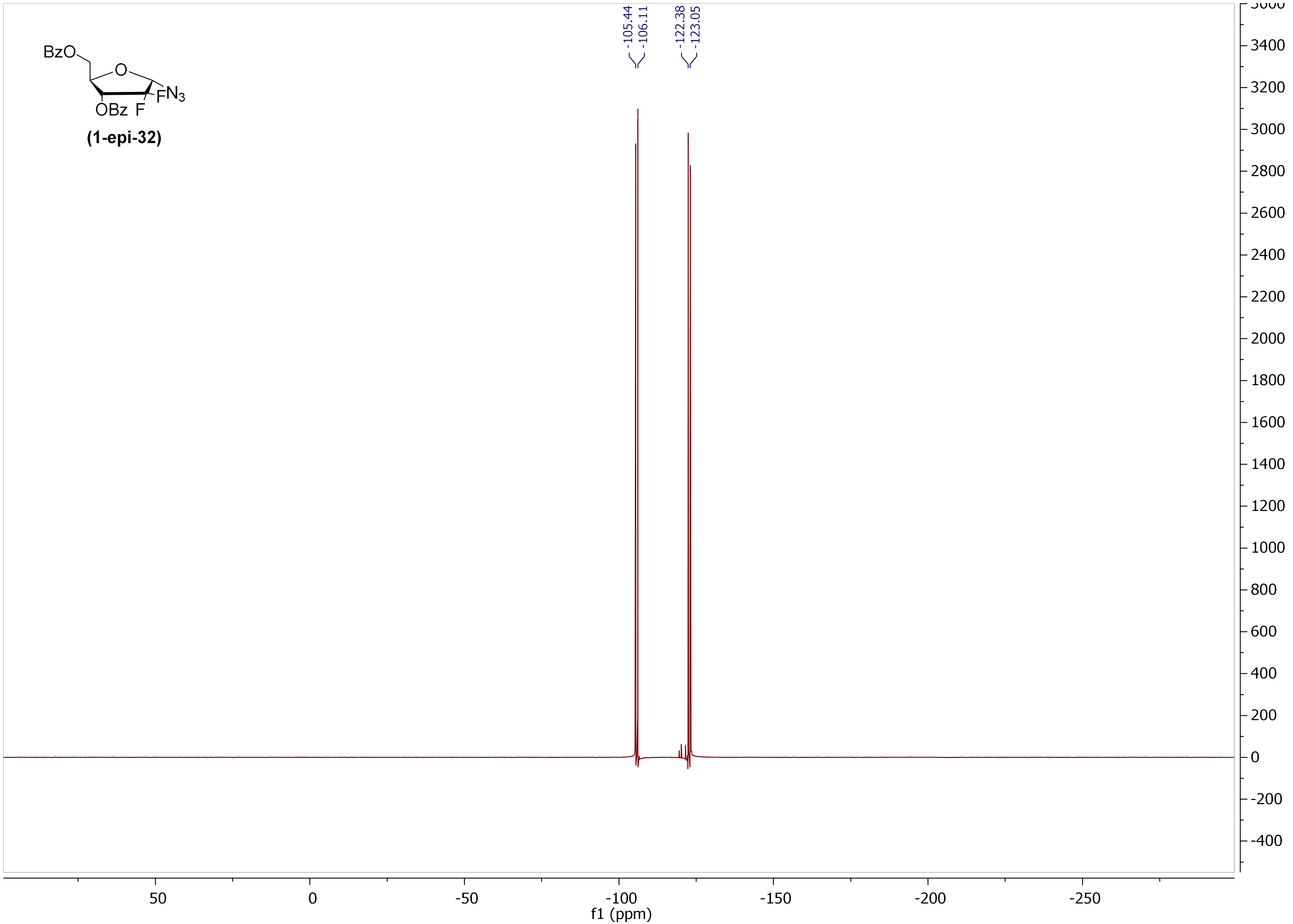

Figure S. 17 - ^{19}F NMR Spectra (377 MHz, $CDCl_3$) - 3,5-di-*O*-benzoyl-2-deoxy-2,2-difluoro-β-*D*-ribofuranosyl azide – **32**

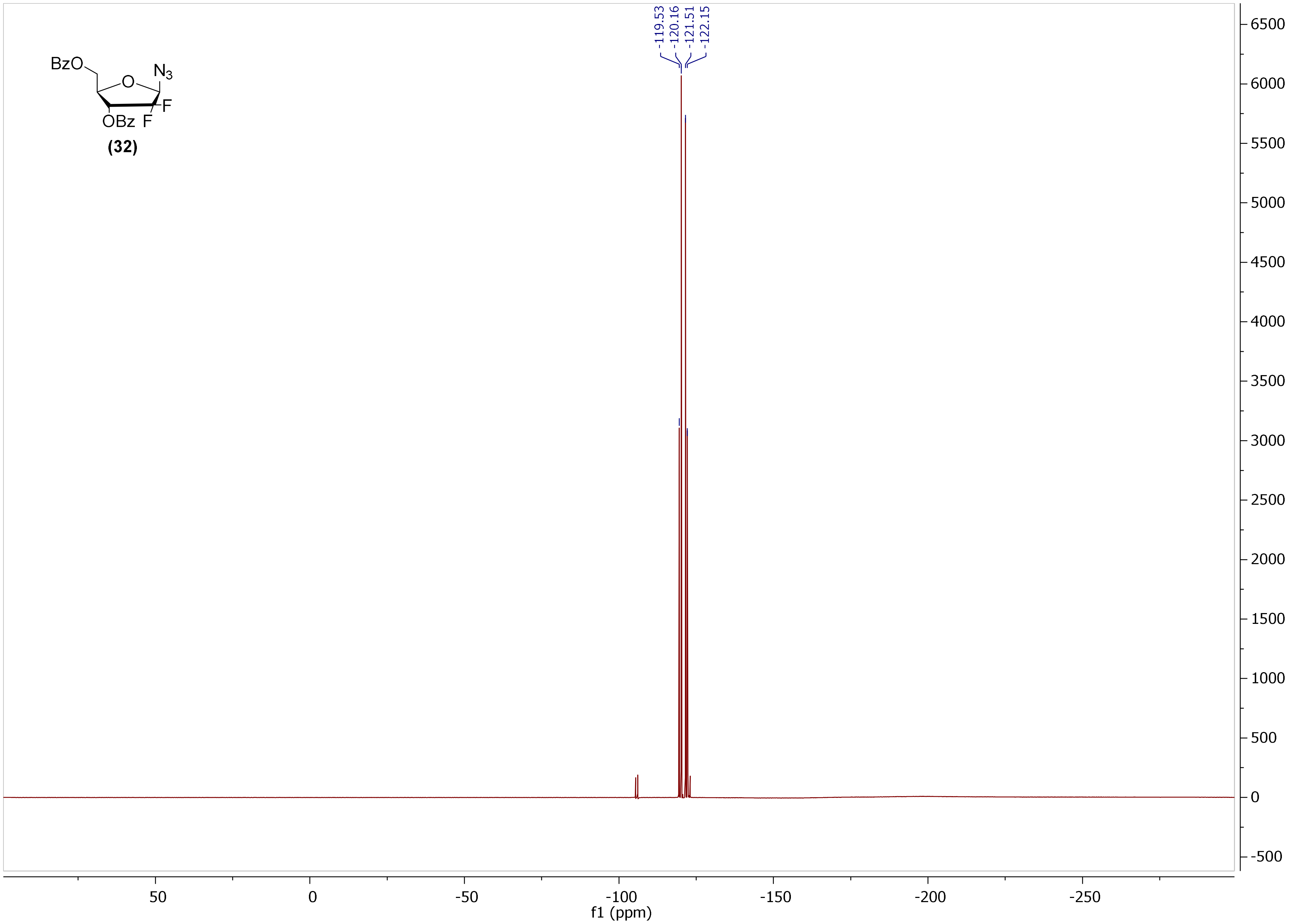

Figure S. 18 - ^{13}C NMR Spectra (101 MHz, $CDCl_3$) - 3,5-di-*O*-benzoyl-2-deoxy-2,2-difluoro-α-*D*-ribofuranosyl azide – (1-epi-**32**)

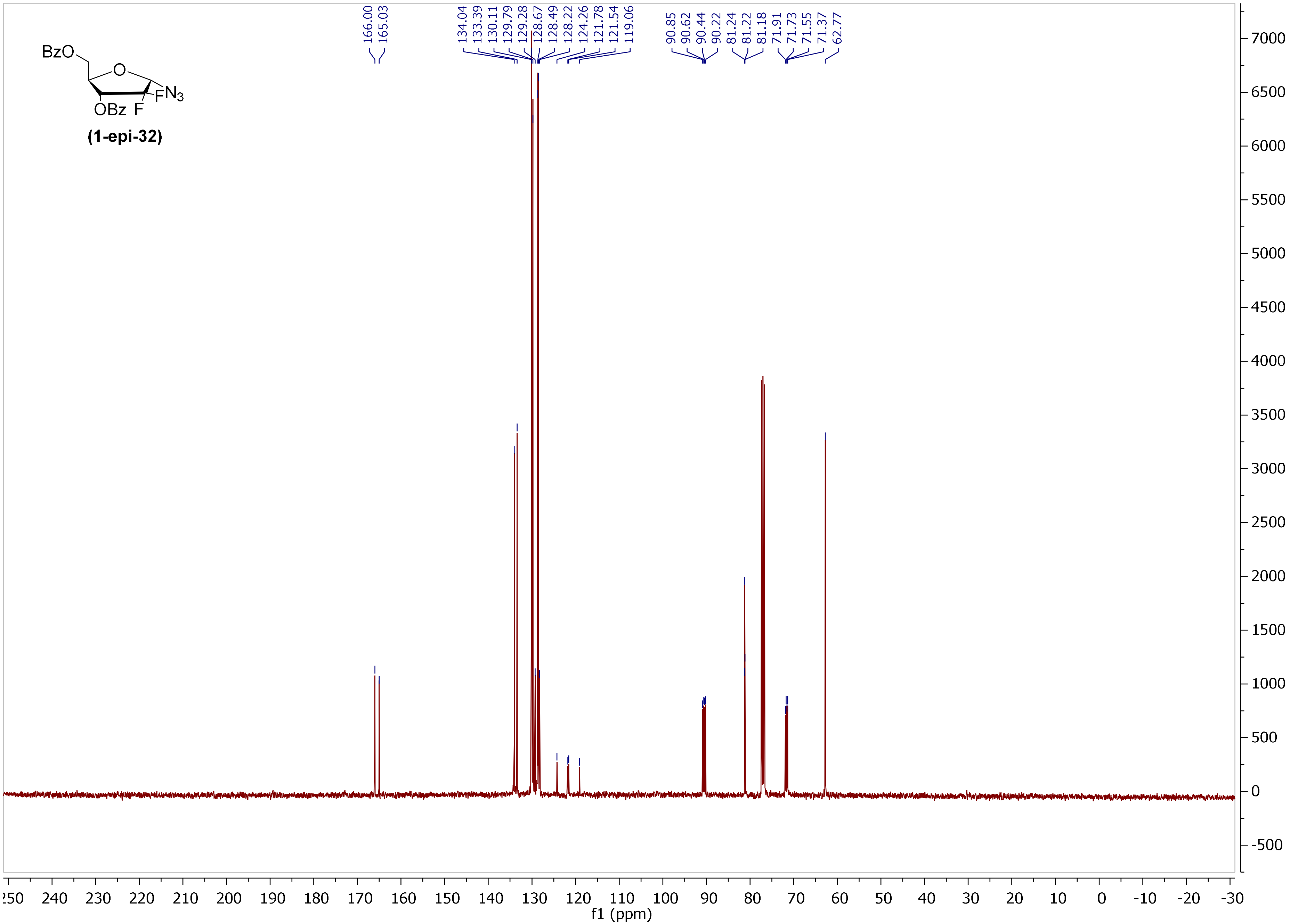

Figure S. 19 - ^{13}C NMR Spectra (101 MHz, $CDCl_3$) - 3,5-di-*O*-benzoyl-2-deoxy-2,2-difluoro-β-*D*-ribofuranosyl azide – **32**

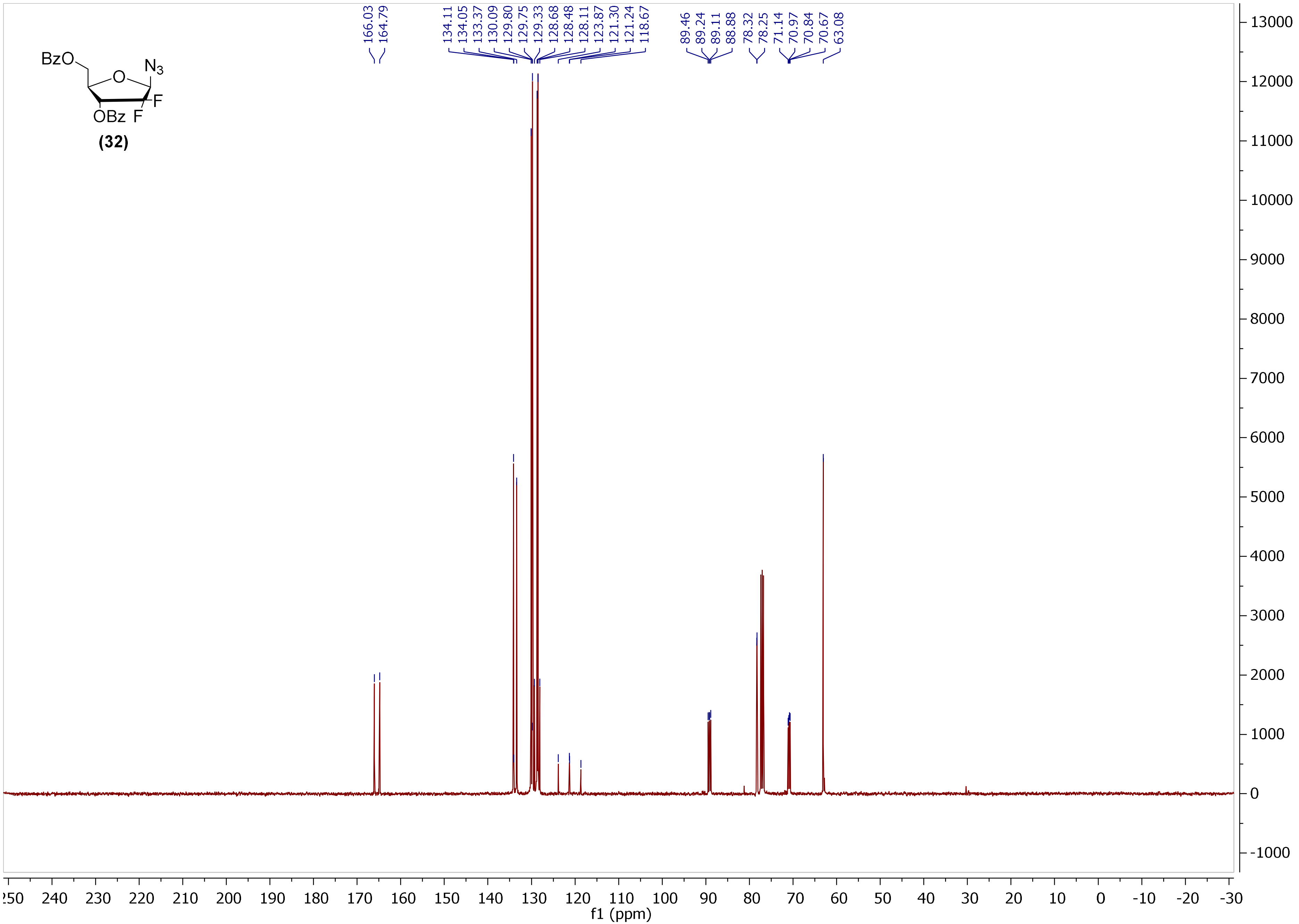

Figure S. 20 - ^{1}H-NMR Spectrum (400 MHz, $CDCl_3$) - 3,5-di-*O*-benzoyl-2-deoxy-2,2-difluoro-α-*D*-ribofuranosyl isonitrile – (1-epi-**34**) – minor anomer

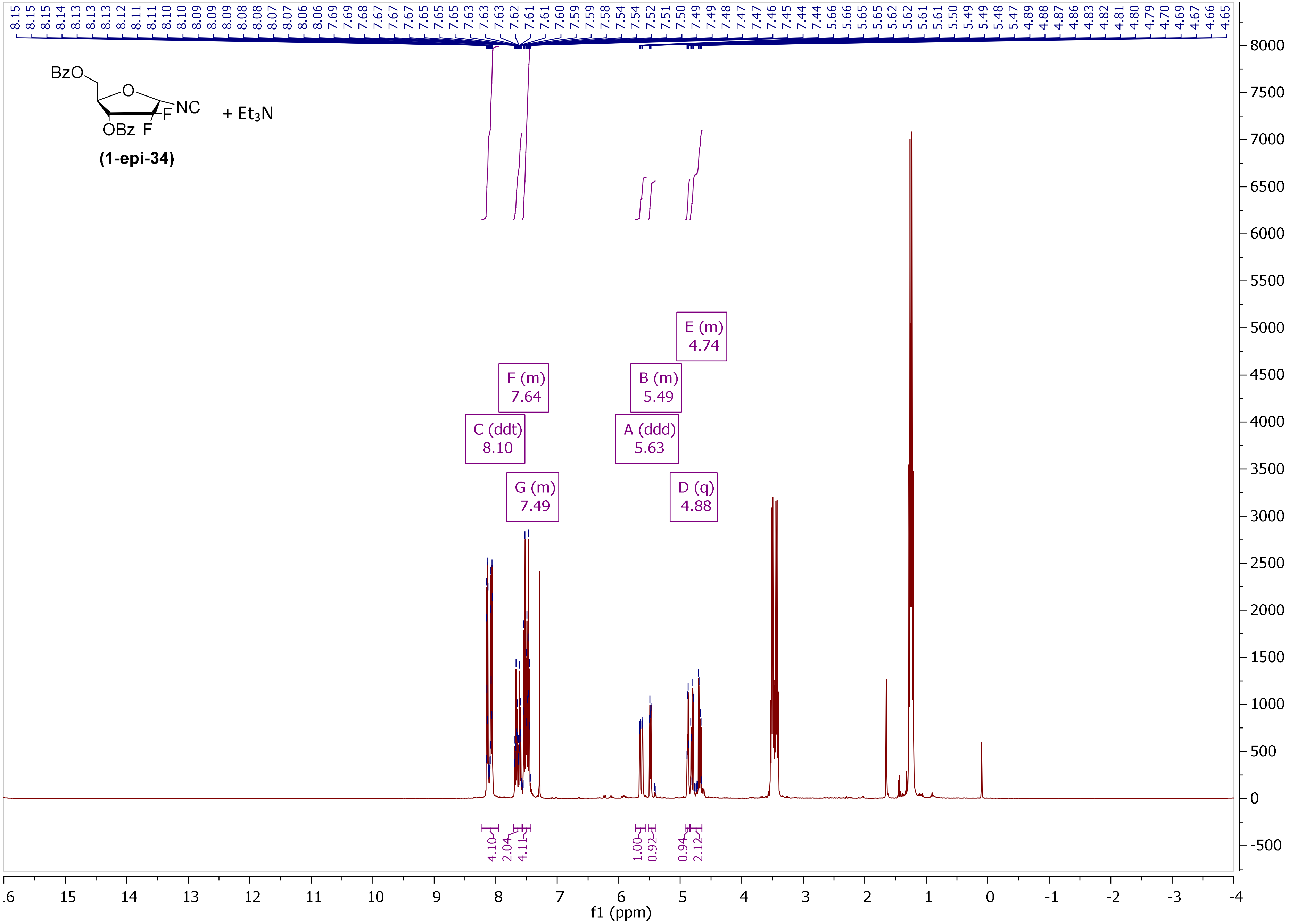

Figure S. 21 - ^{19}F NMR Spectra (377 MHz, $CDCl_3$) - 3,5-di-*O*-benzoyl-2-deoxy-2,2-difluoro-α-*D*-ribofuranosyl isonitrile– (1-epi-**34**) – minor anomer

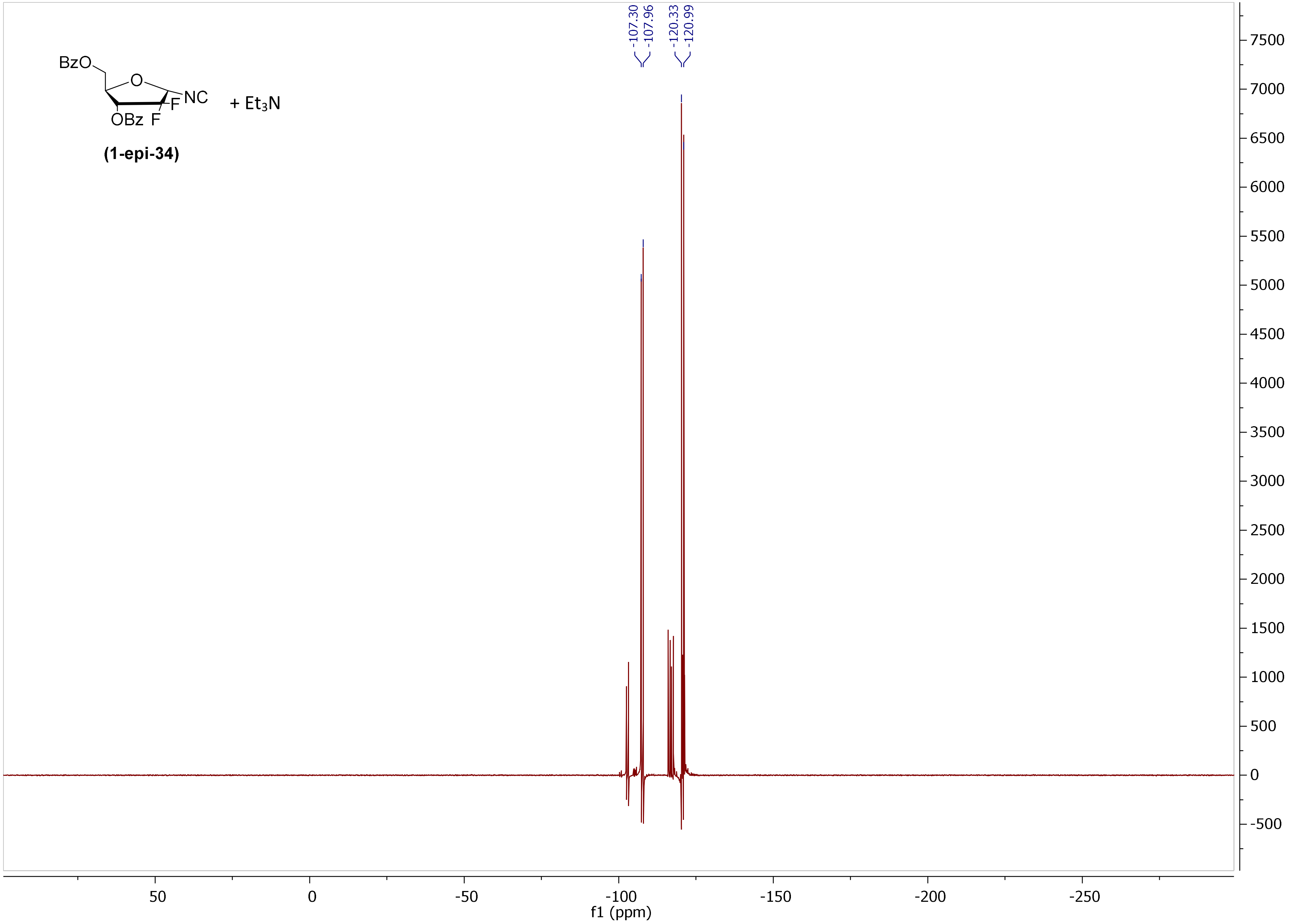

Figure S. 22 - ^{13}C NMR Spectra (101 MHz, $CDCl_3$) - 3,5-di-*O*-benzoyl-2-deoxy-2,2-difluoro-α-*D*-ribofuranosyl isonitrile – (1-epi-**34**)

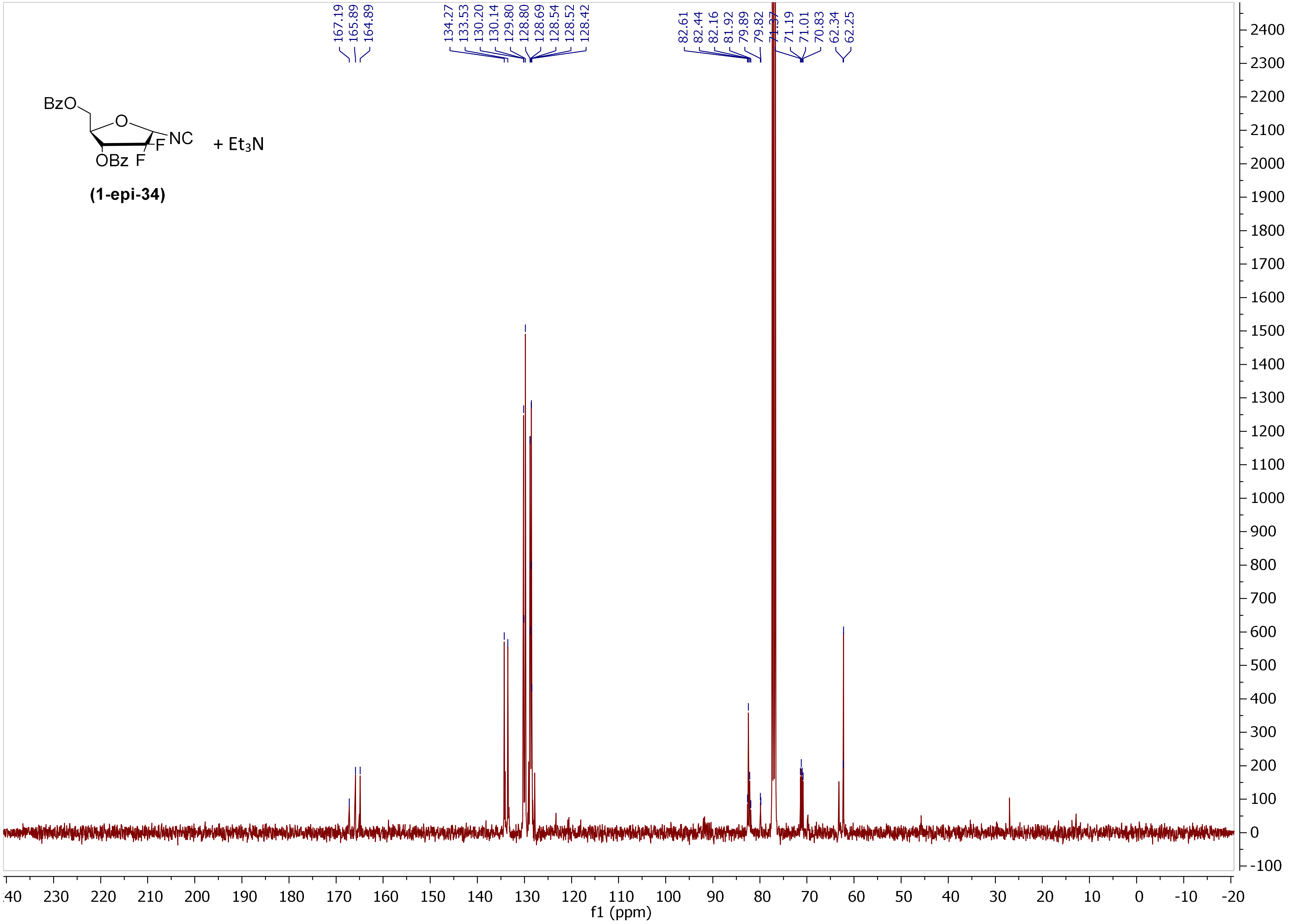

Figure S. 23 - ^{1}H-NMR Spectrum (400 MHz, $CDCl_3$) - 3,5-di-*O*-benzoyl-2-deoxy-2,2-difluoro-β-*D*-ribofuranosyl isonitrile – **34**

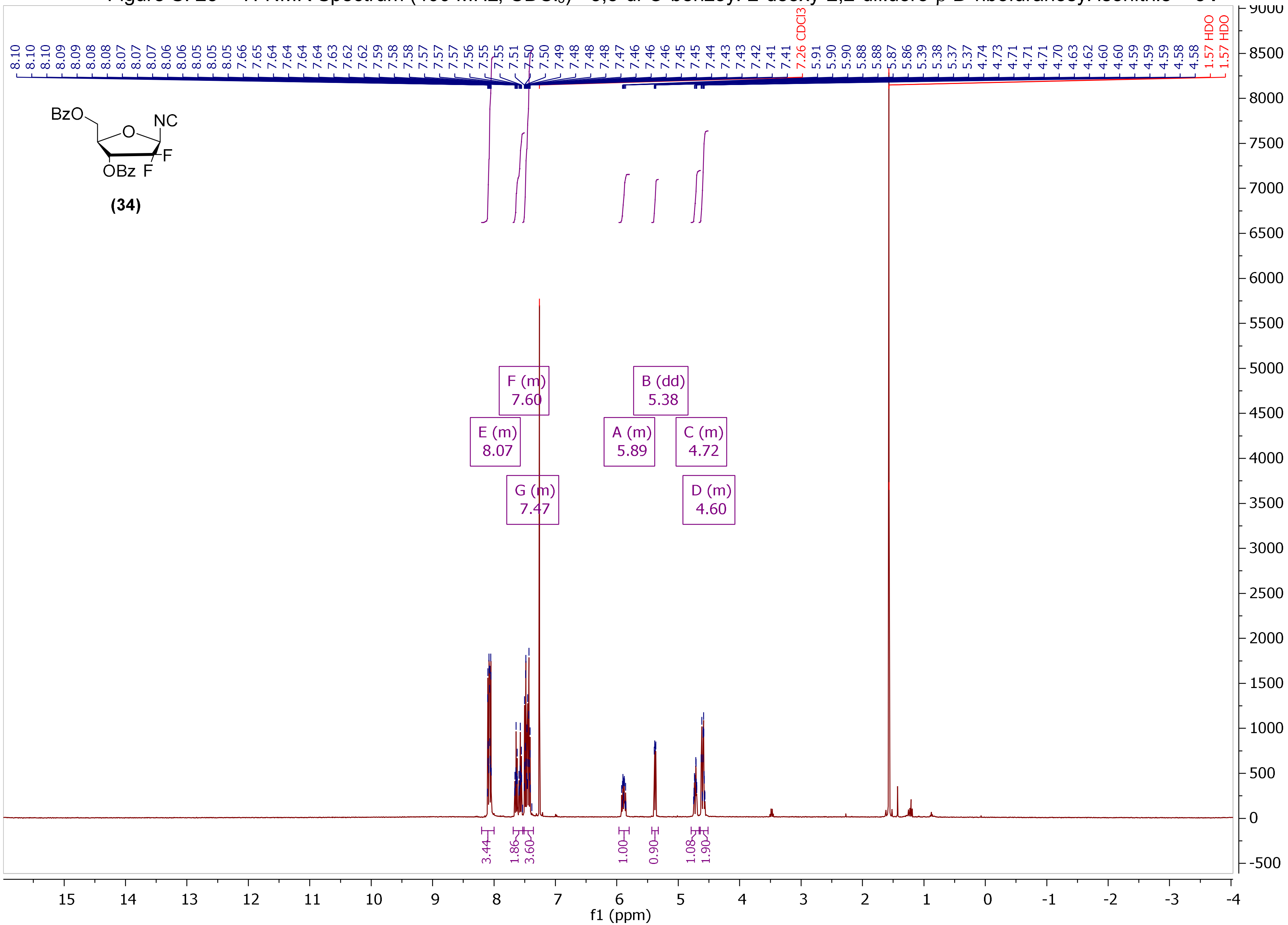

Figure S. 24 - ^{19}F NMR Spectra (377 MHz, $CDCl_3$) - 3,5-di-*O*-benzoyl-2-deoxy-2,2-difluoro-β-*D*-ribofuranosyl isonitrile – **34**

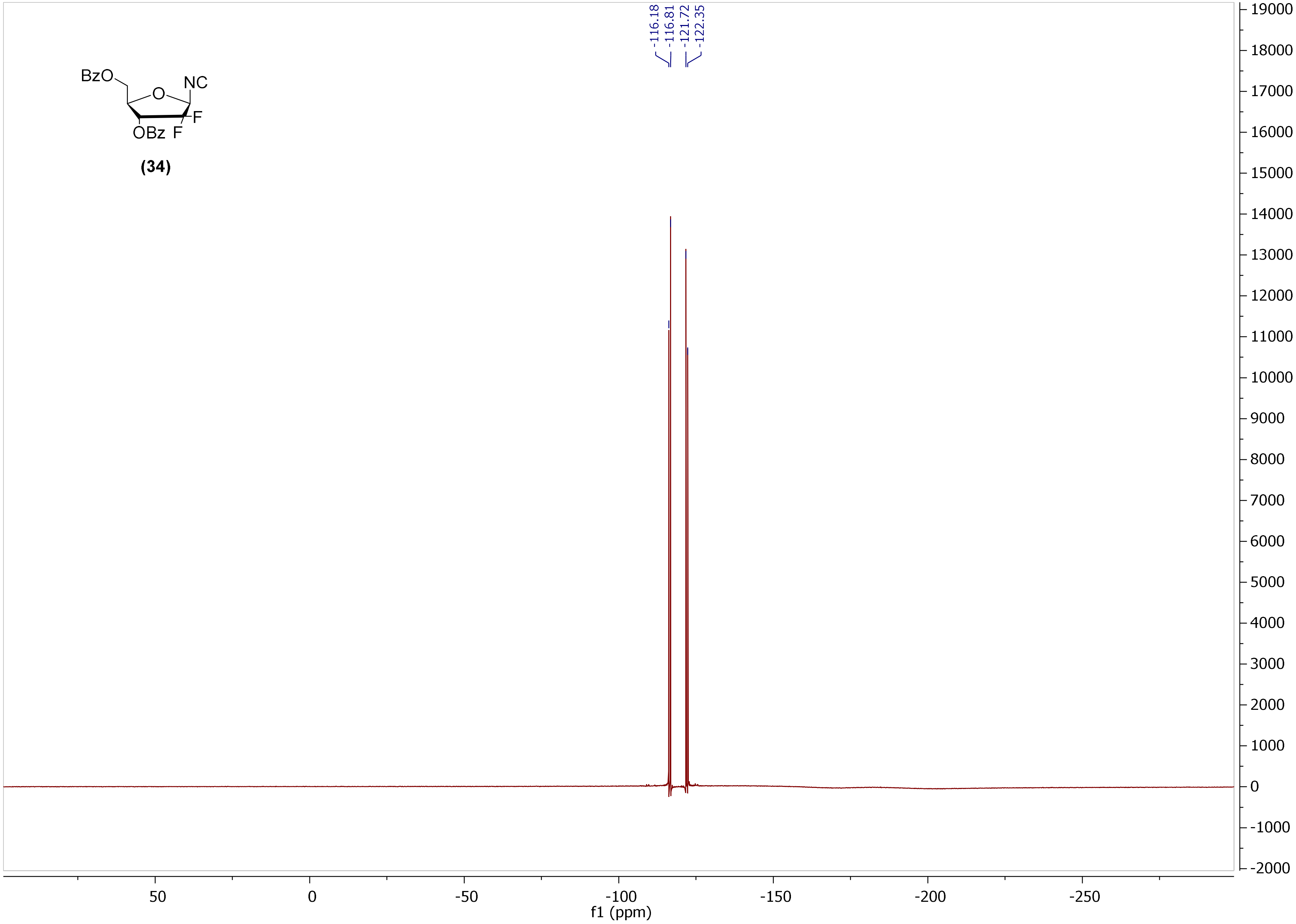

Figure S. 25 - ^{13}C NMR Spectra (101 MHz, $CDCl_3$) - 3,5-di-*O*-benzoyl-2-deoxy-2,2-difluoro-β-*D*-ribofuranosyl isonitrile – **34**

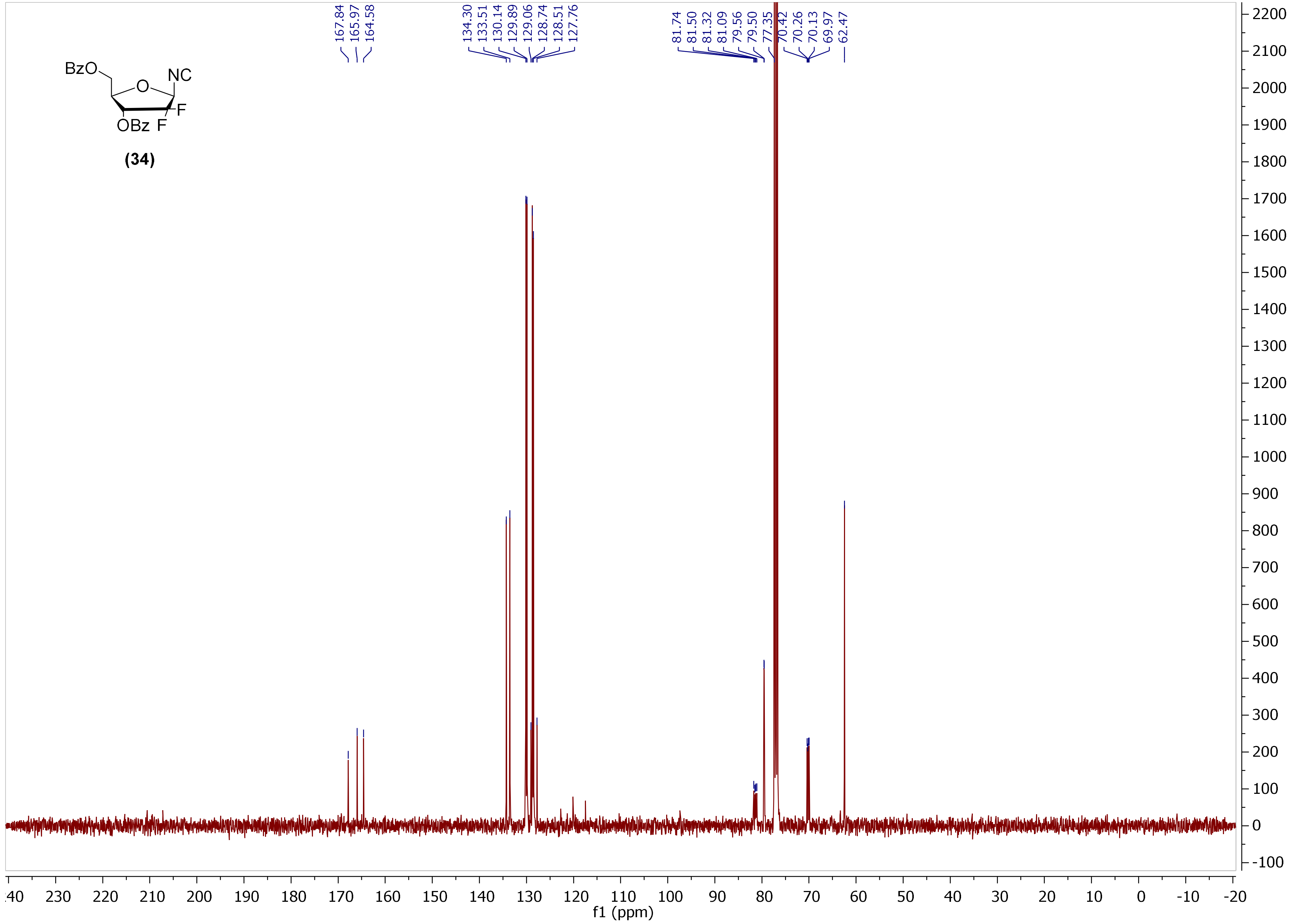

(34)

Figure S. 26 - ^{1}H-NMR Spectrum (400 MHz, $CDCl_3$) - 3,5-Di-*O*-benzoyl-2-deoxy-2-fluoro-2-methyl-*D*-ribofuranosyl iodide - **37**

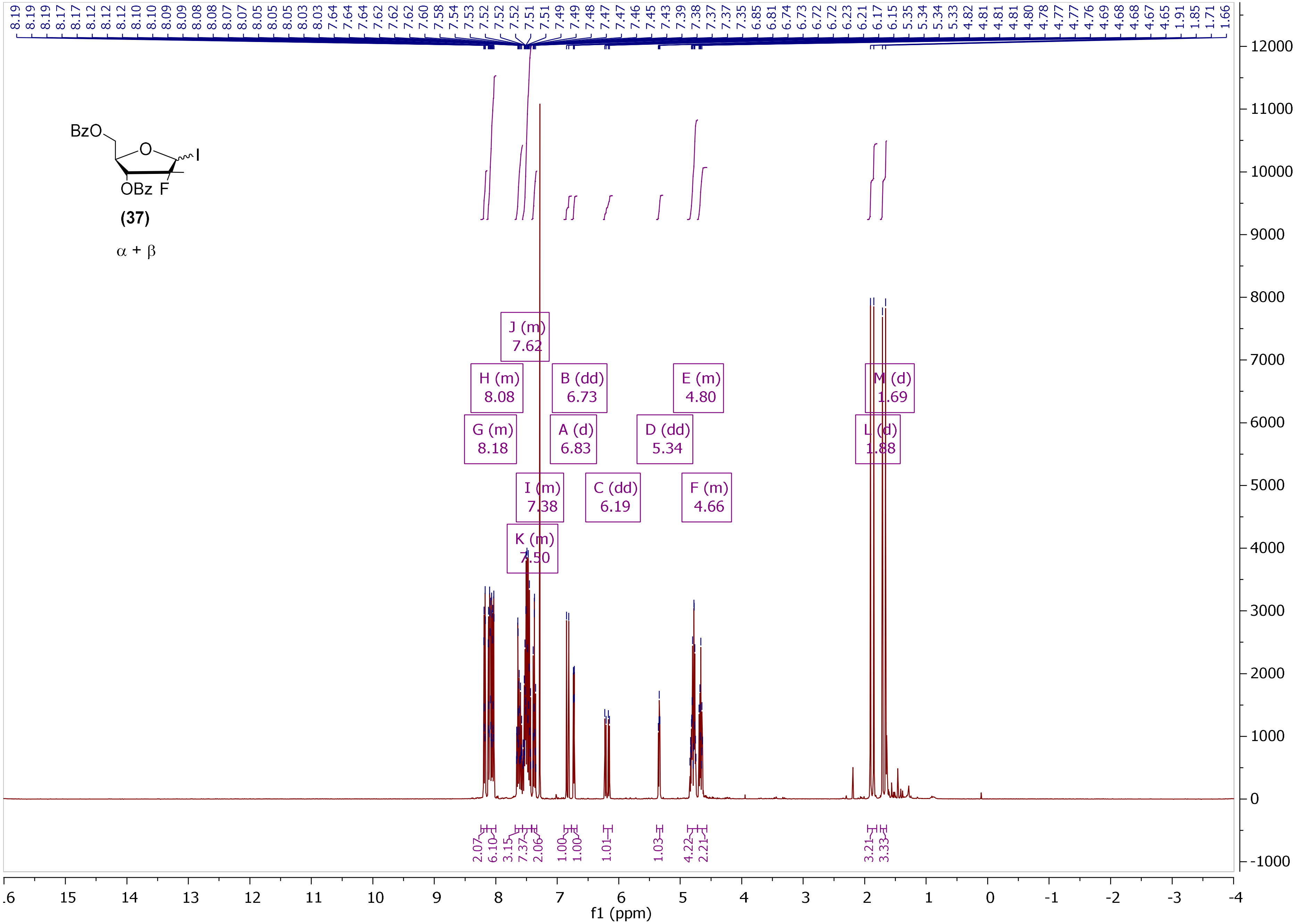

Figure S. 27 - ^{13}C NMR Spectra (101 MHz, $CDCl_3$) - 3,5-Di-*O*-benzoyl-2-deoxy-2-fluoro-2-methyl-*D*-ribofuranosyl iodide – **37**

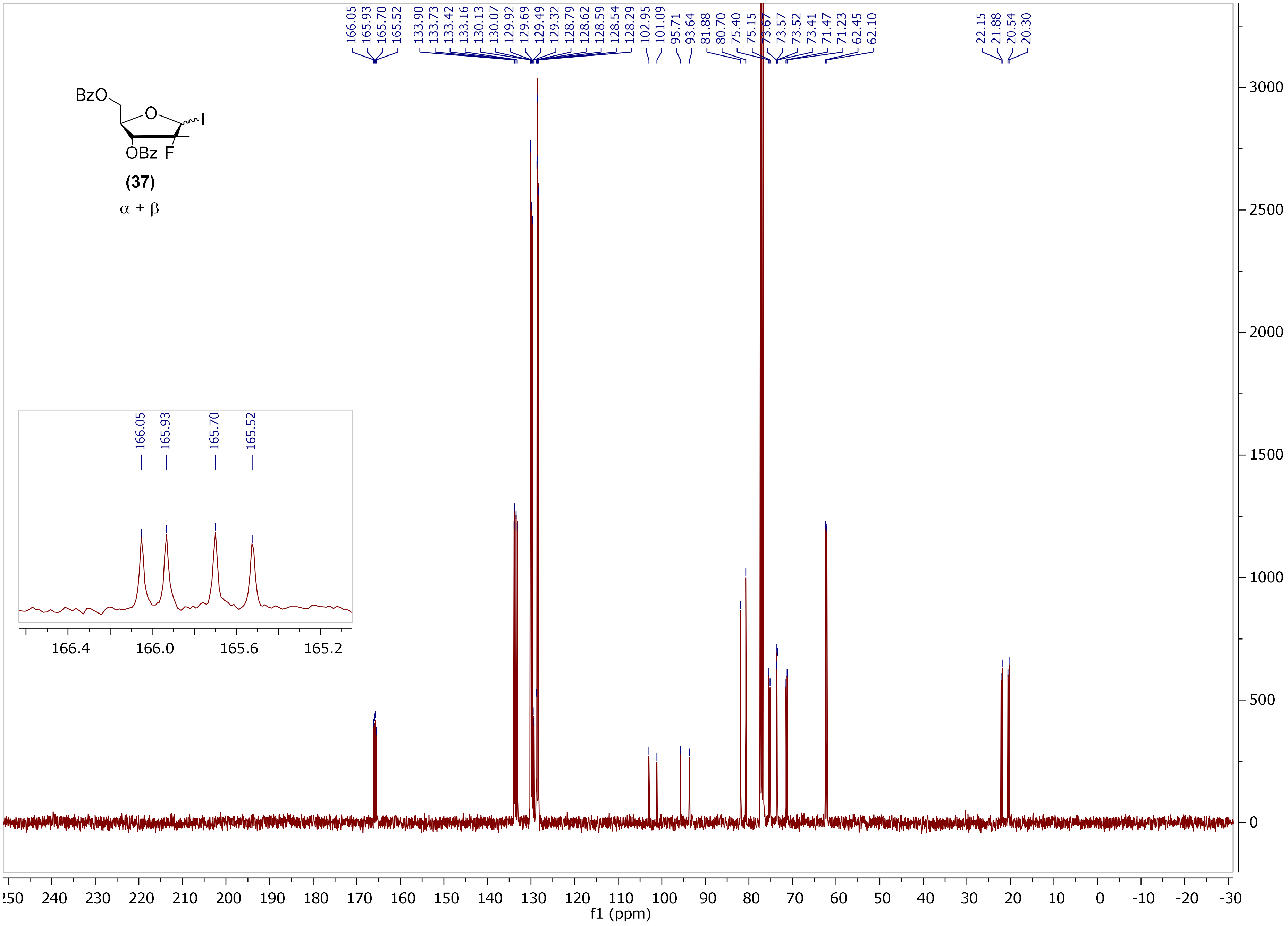

Figure S. 28 - ^{19}F NMR Spectra (377 MHz, $CDCl_3$) - 3,5-Di-*O*-benzoyl-2-deoxy-2-fluoro-2-methyl-*D*-ribofuranosyl iodide –**37**

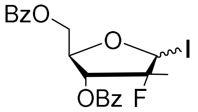

(37)

α + β

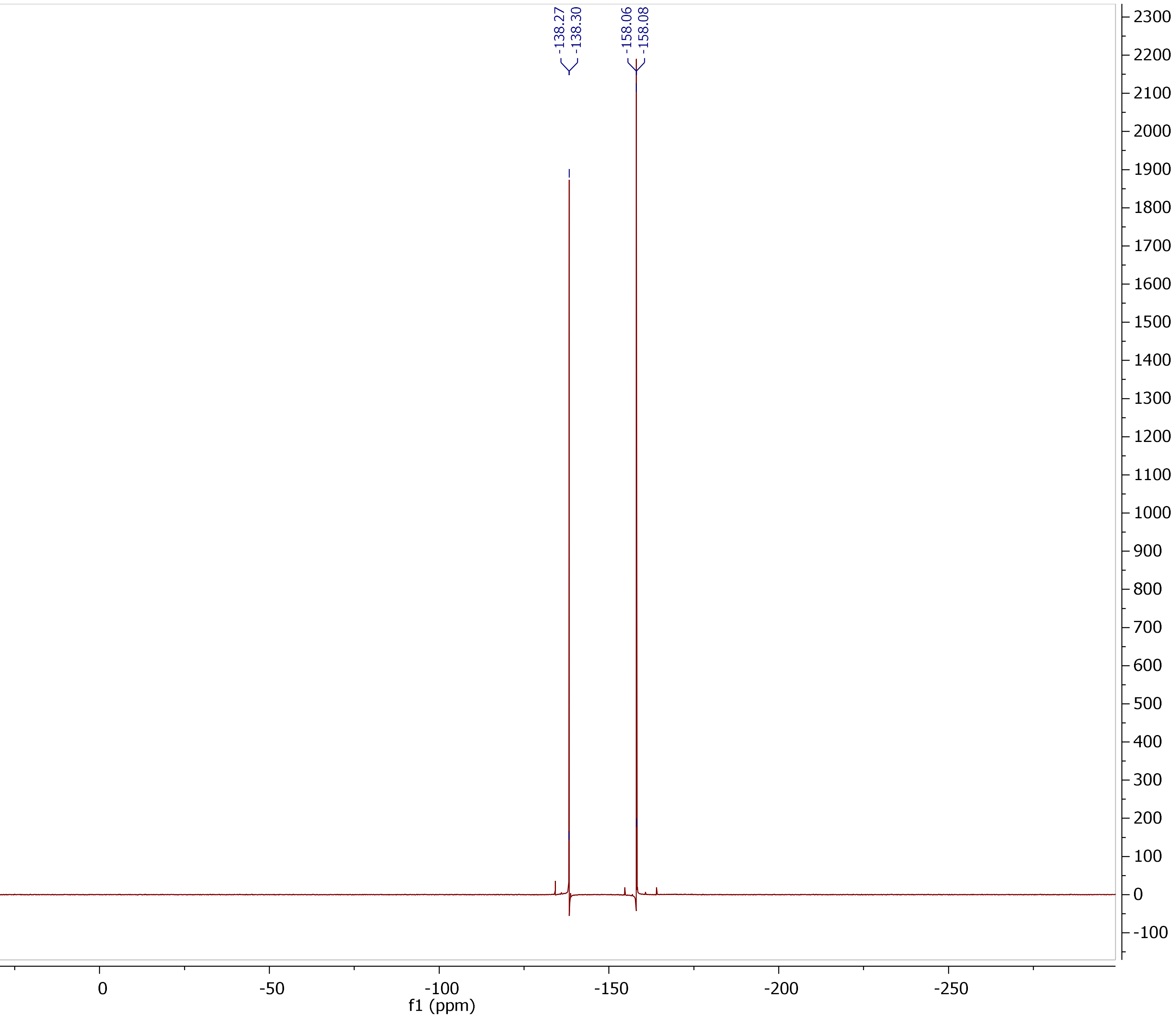

Figure S. 29 - ^{1}H-NMR Spectrum (400 MHz, $CDCl_3$) - 3,5-Di-*O*-benzoyl-2-deoxy-2-fluoro-2-methyl-β-*D*-ribofuranosyl azide – **38**

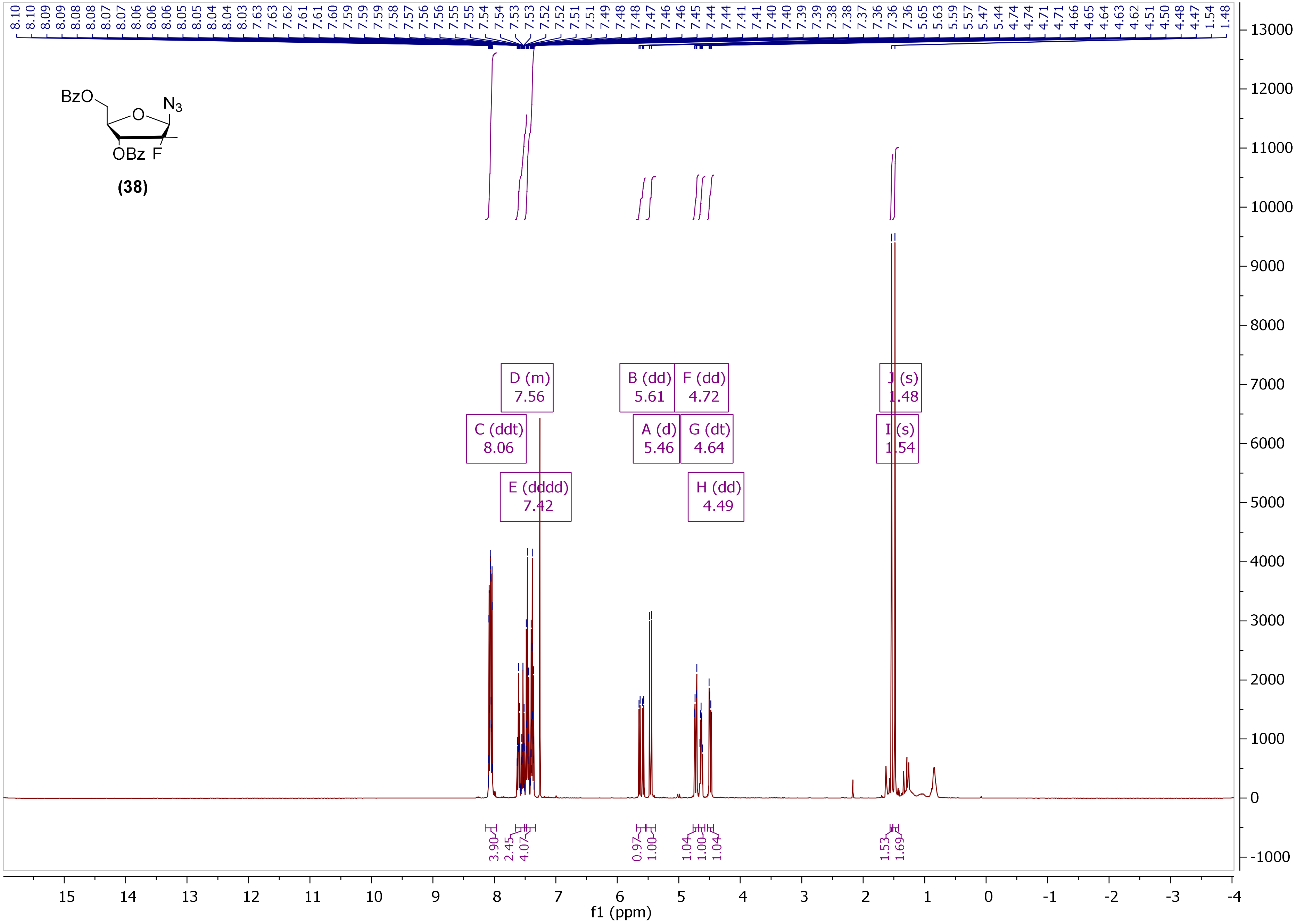

Figure S. 30 - ^{13}C NMR Spectra (101 MHz, $CDCl_3$) - 3,5-Di-*O*-benzoyl-2-deoxy-2-fluoro-2-methyl-β-*D*-ribofuranosyl azide – **38**

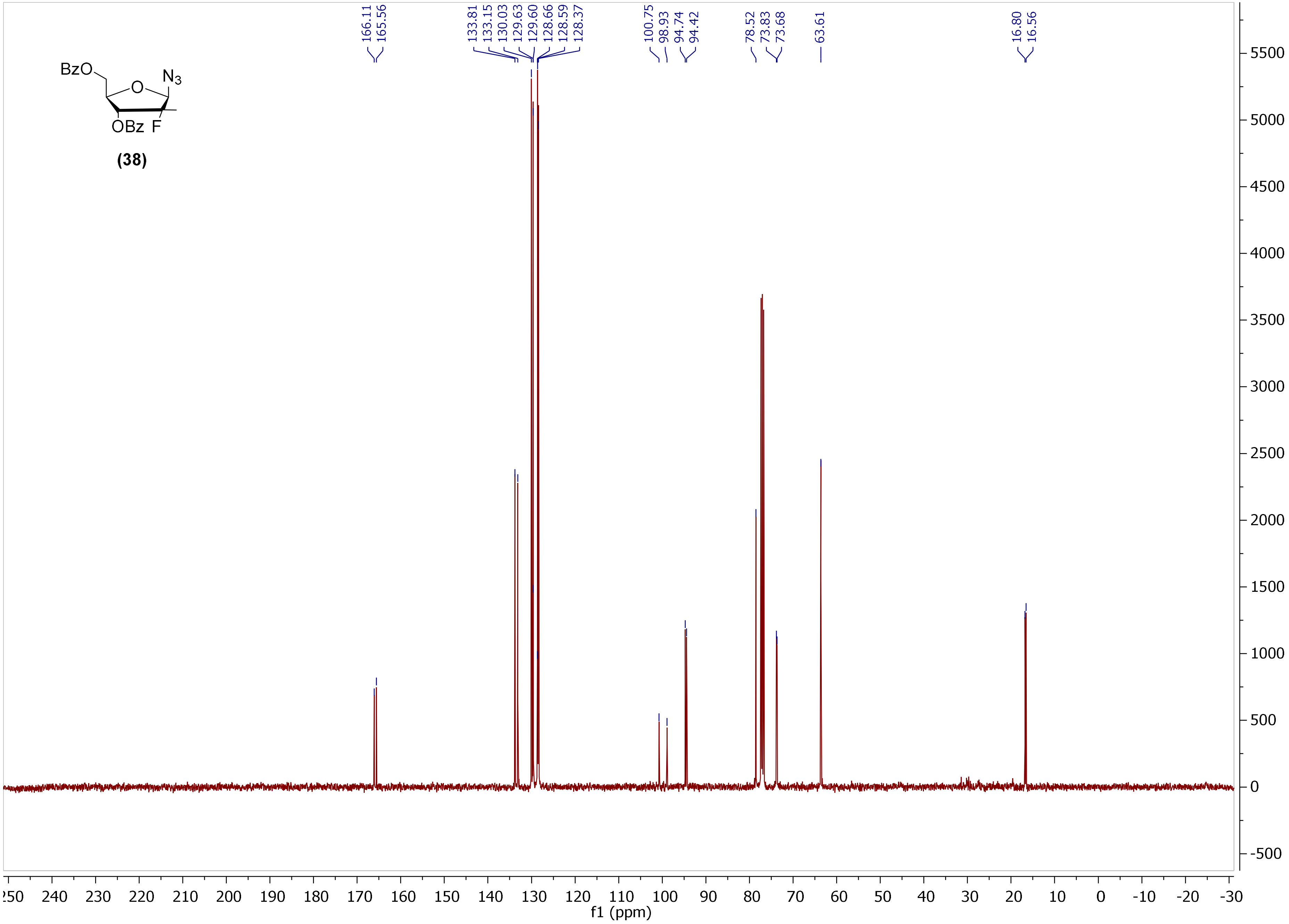

Figure S. 31 - ^{19}F NMR Spectra (377 MHz, $CDCl_3$) - 3,5-Di-*O*-benzoyl-2-deoxy-2-fluoro-2-methyl-β-*D*-ribofuranosyl azide – **38**

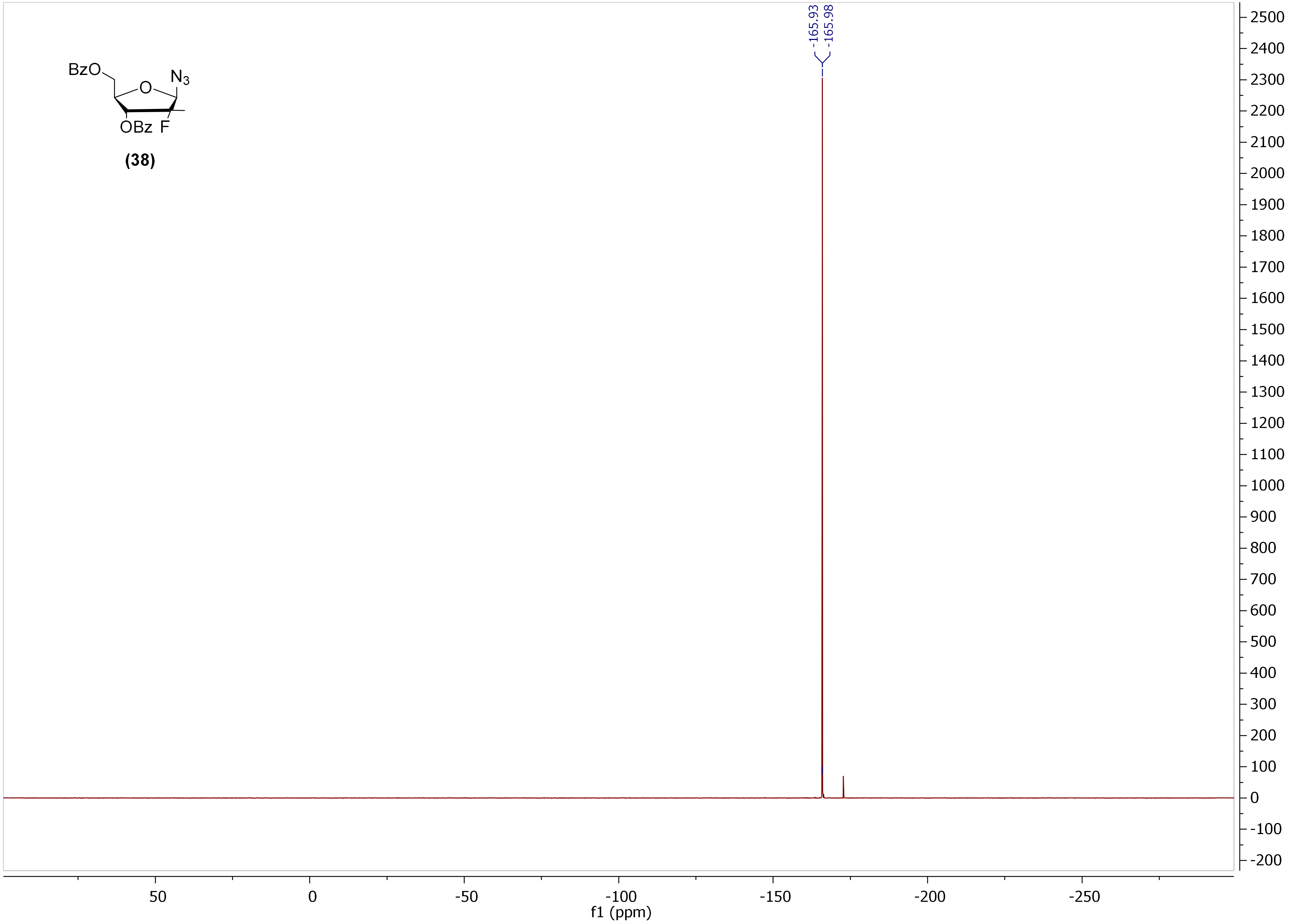

Figure S. 32 - ^{1}H-NMR Spectrum (400 MHz, $CDCl_3$) - 3,5-Di-*O*-benzoyl-2-deoxy-2-fluoro-2-methyl-α-*D*-ribofuranosyl azide – (1-epi-**38**)

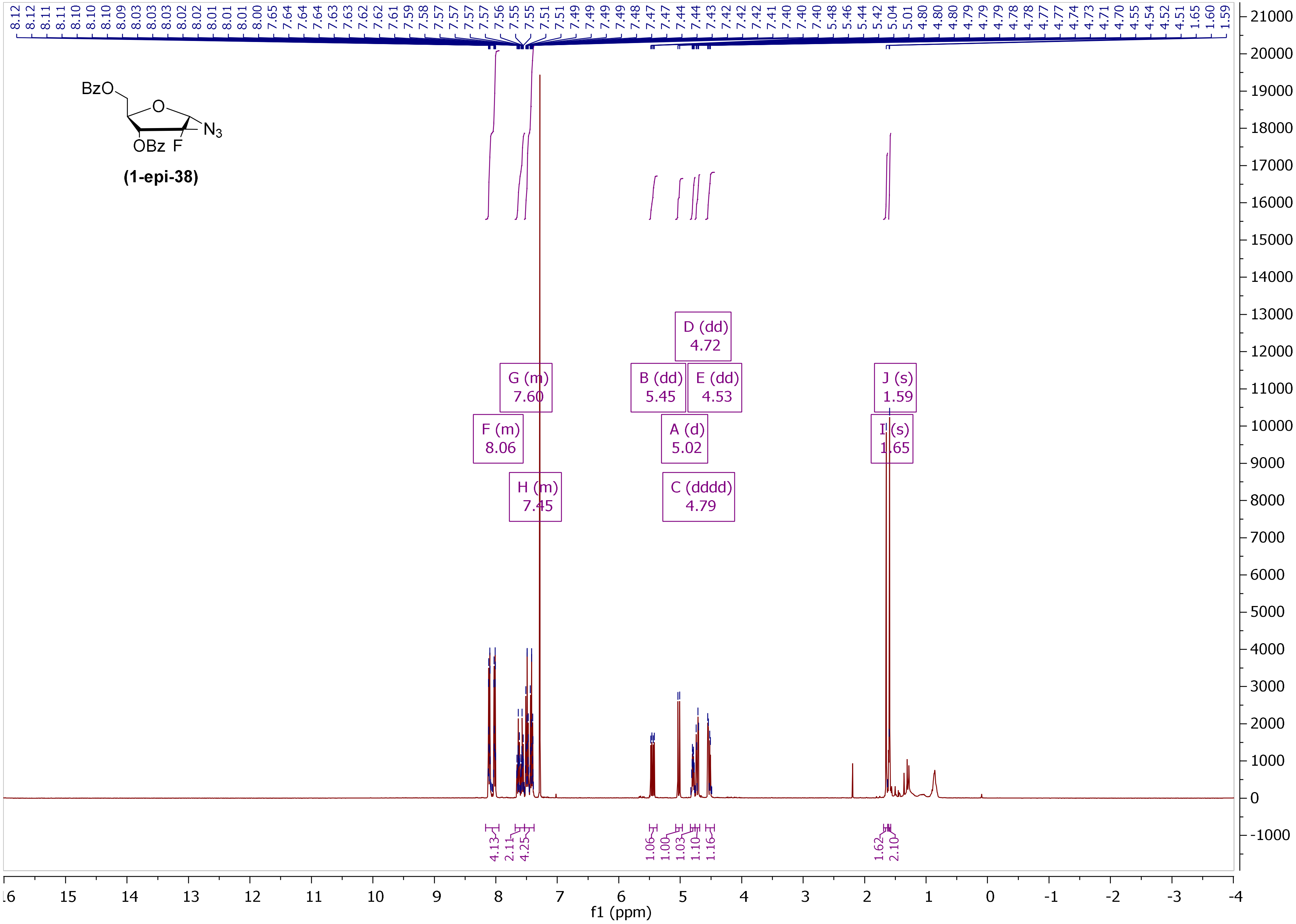

Figure S. 33 - ^{13}C NMR Spectra (101 MHz, $CDCl_3$) - 3,5-Di-*O*-benzoyl-2-deoxy-2-fluoro-2-methyl-α-*D*-ribofuranosyl azide – (1-epi-**38)**

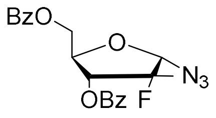

(1-epi-38)

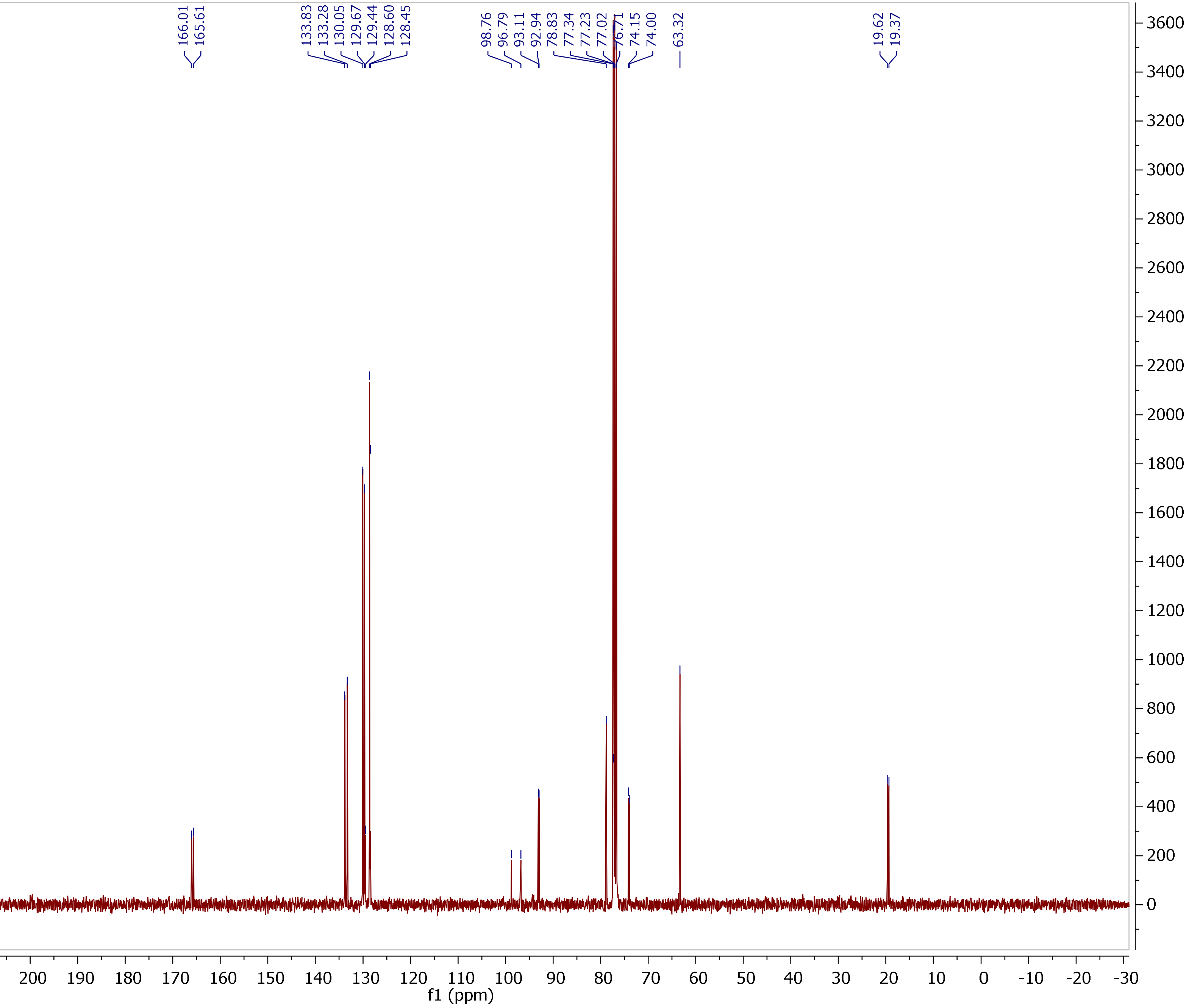

Figure S. 34 - ^{19}F NMR Spectra (377 MHz, $CDCl_3$) - 3,5-Di-*O*-benzoyl-2-deoxy-2-fluoro-2-methyl-α-*D*-ribofuranosyl azide – (1-epi-**38**)

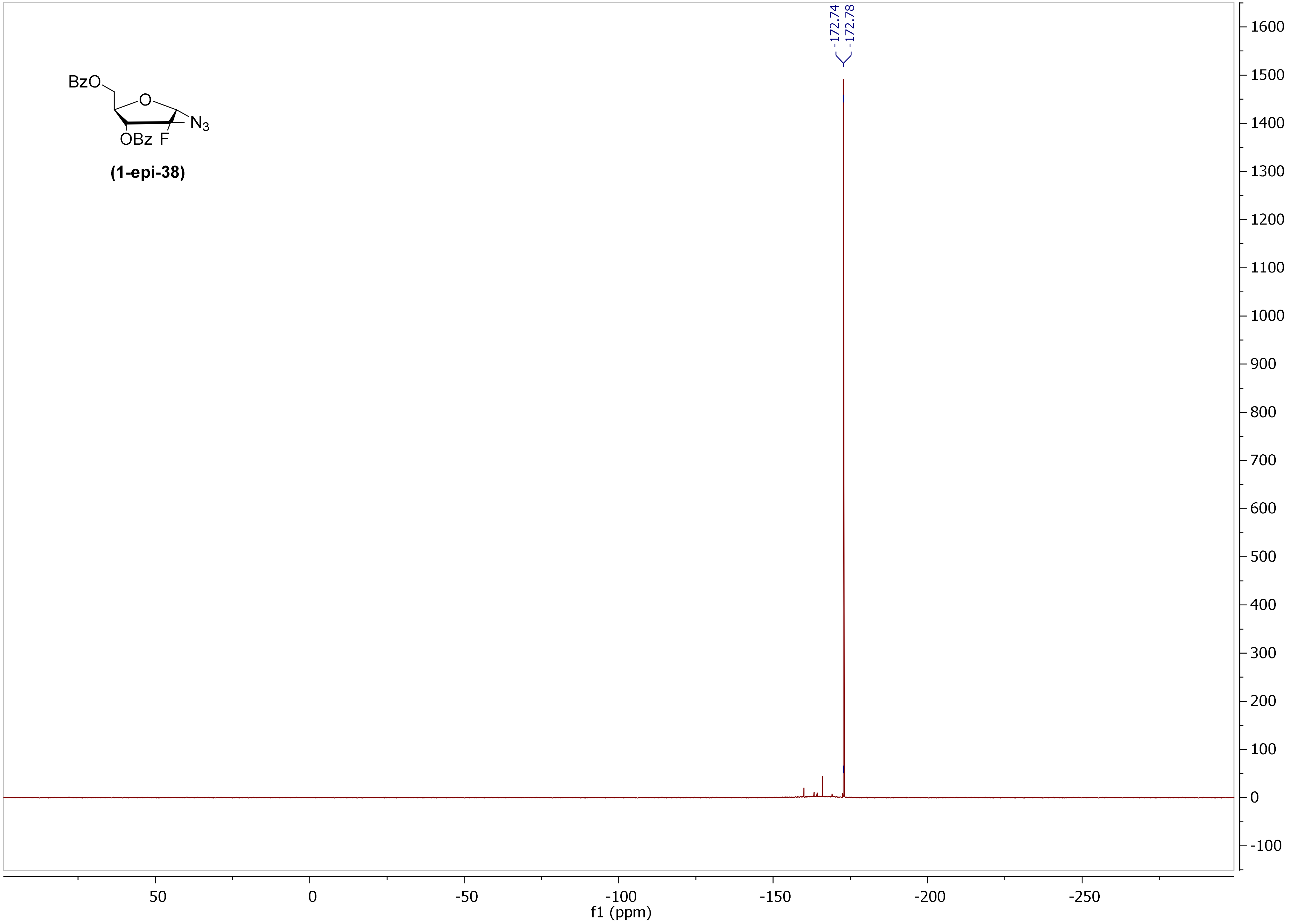

Figure S. 35 - ^{1}H-NMR Spectrum (400 MHz, $CDCl_3$) - 3,5-Di-*O*-benzoyl-2-deoxy-2-fluoro-2-methyl-*β*-*D*-ribofuranosyl isonitrile – **40**

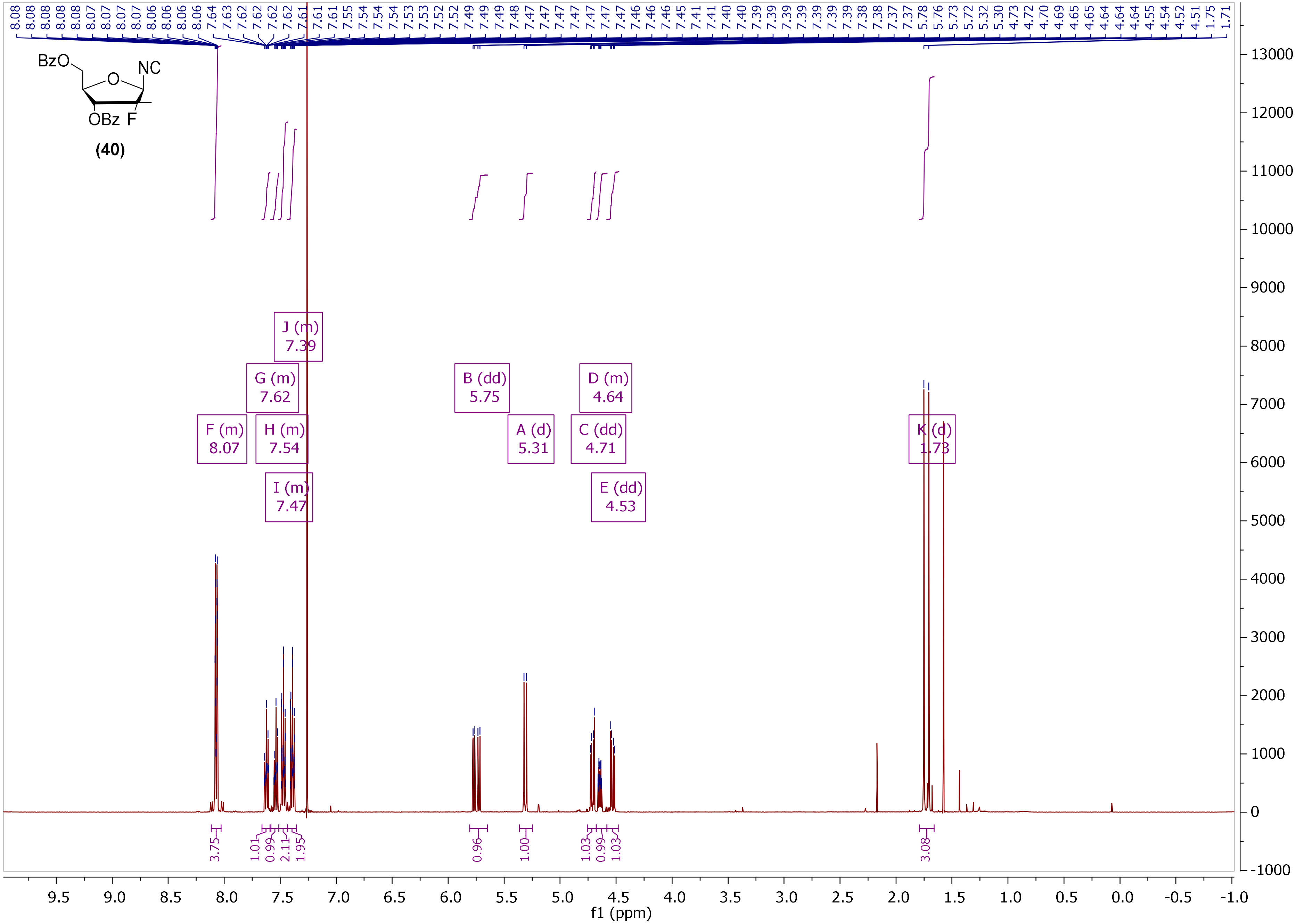

Figure S. 36 - ^{19}F NMR Spectra (377 MHz, $CDCl_3$) - 3,5-Di-*O*-benzoyl-2-deoxy-2-fluoro-2-methyl-β-*D*-ribofuranosyl isonitrile – **40**

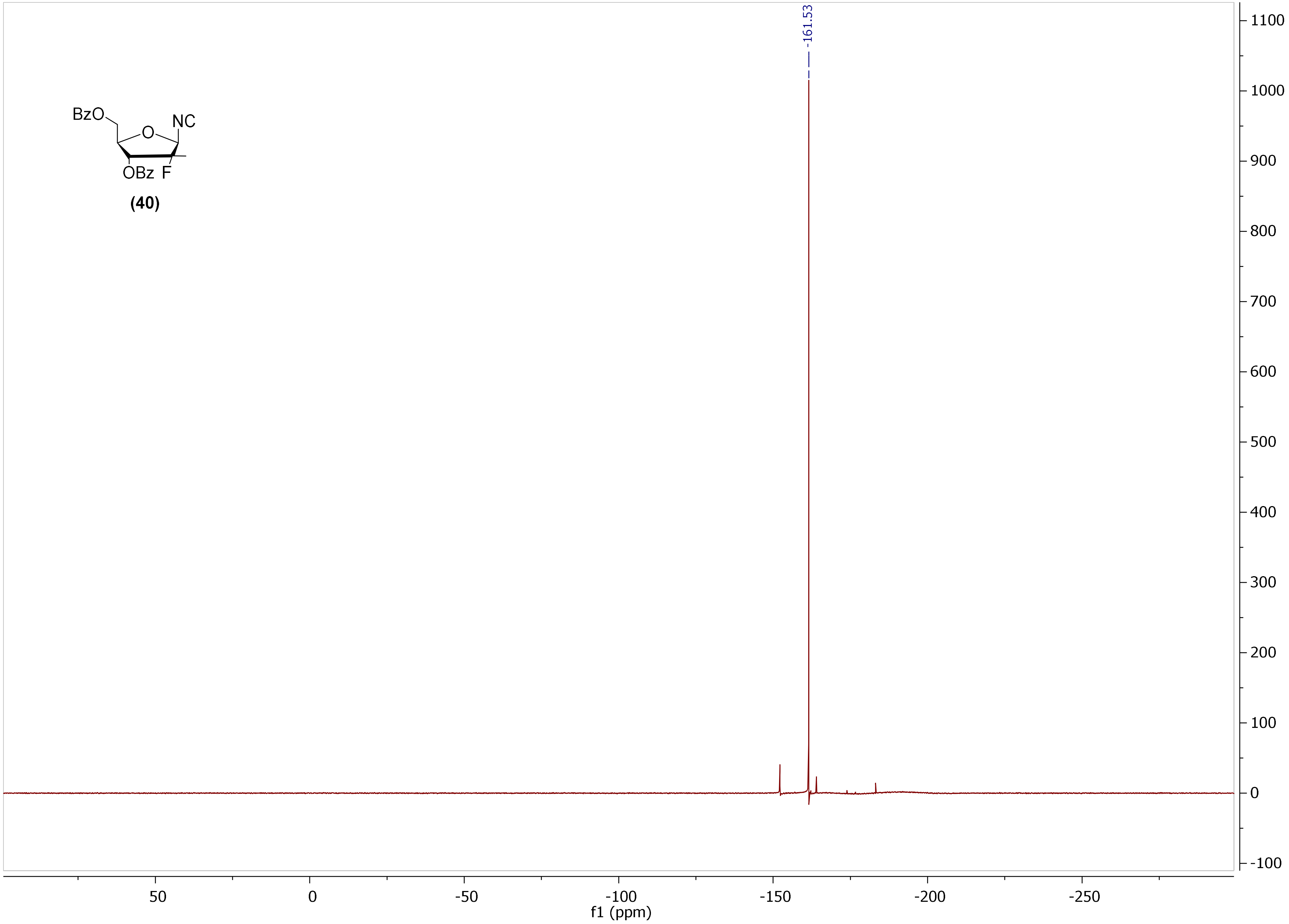

Figure S. 37 - ^{13}C NMR Spectra (101 MHz, $CDCl_3$) - 3,5-Di-*O*-benzoyl-2-deoxy-2-fluoro-2-methyl-β-*D*-ribofuranosyl isonitrile – **40**

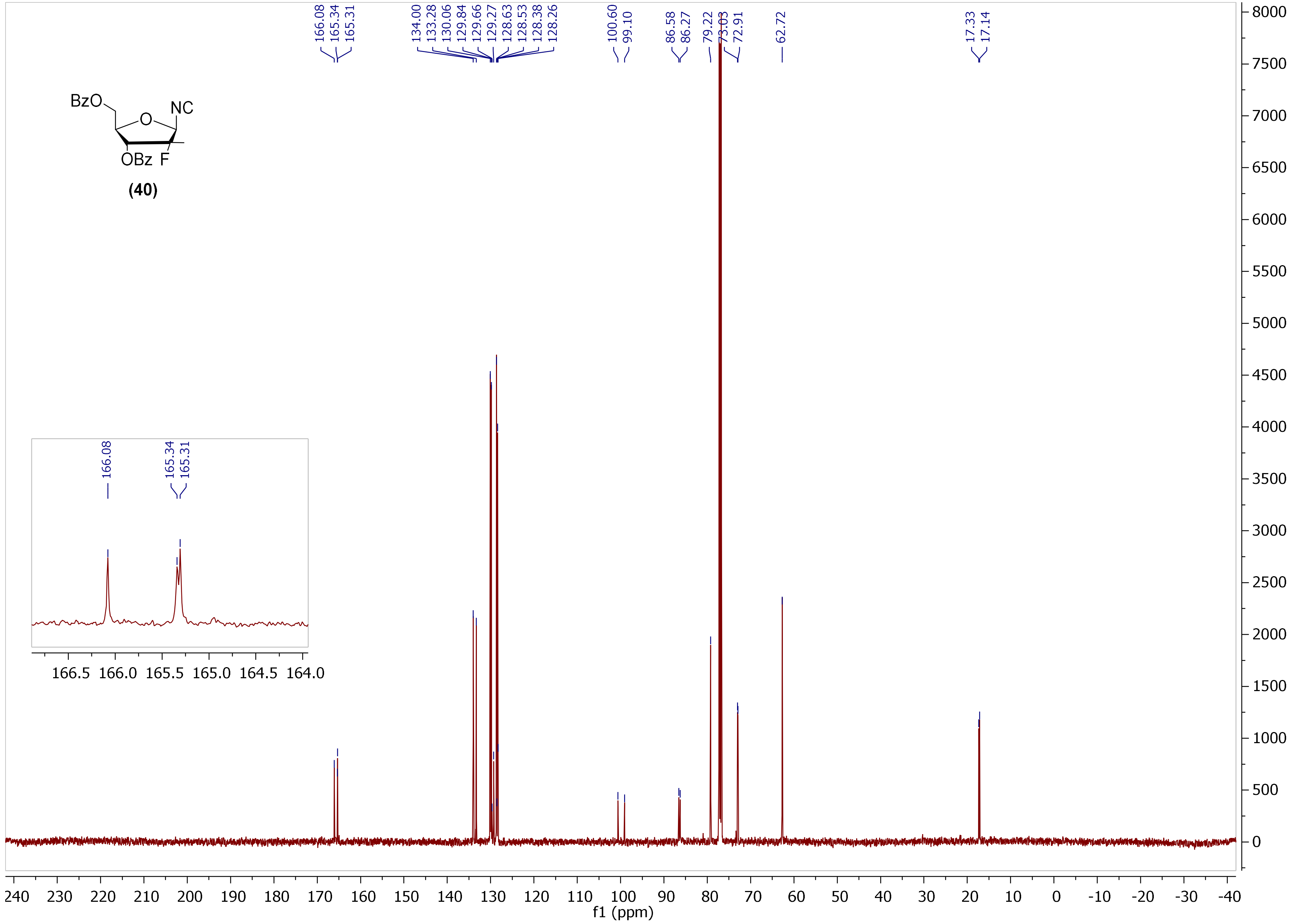

Figure S. 38 - ^{1}H-NMR Spectrum (400 MHz, $CDCl_3$) - 3,5-Di-*O*-benzoyl-2-deoxy-2-fluoro-2-methyl-α-*D*-ribofuranosyl isonitrile – (1-epi-**40**)

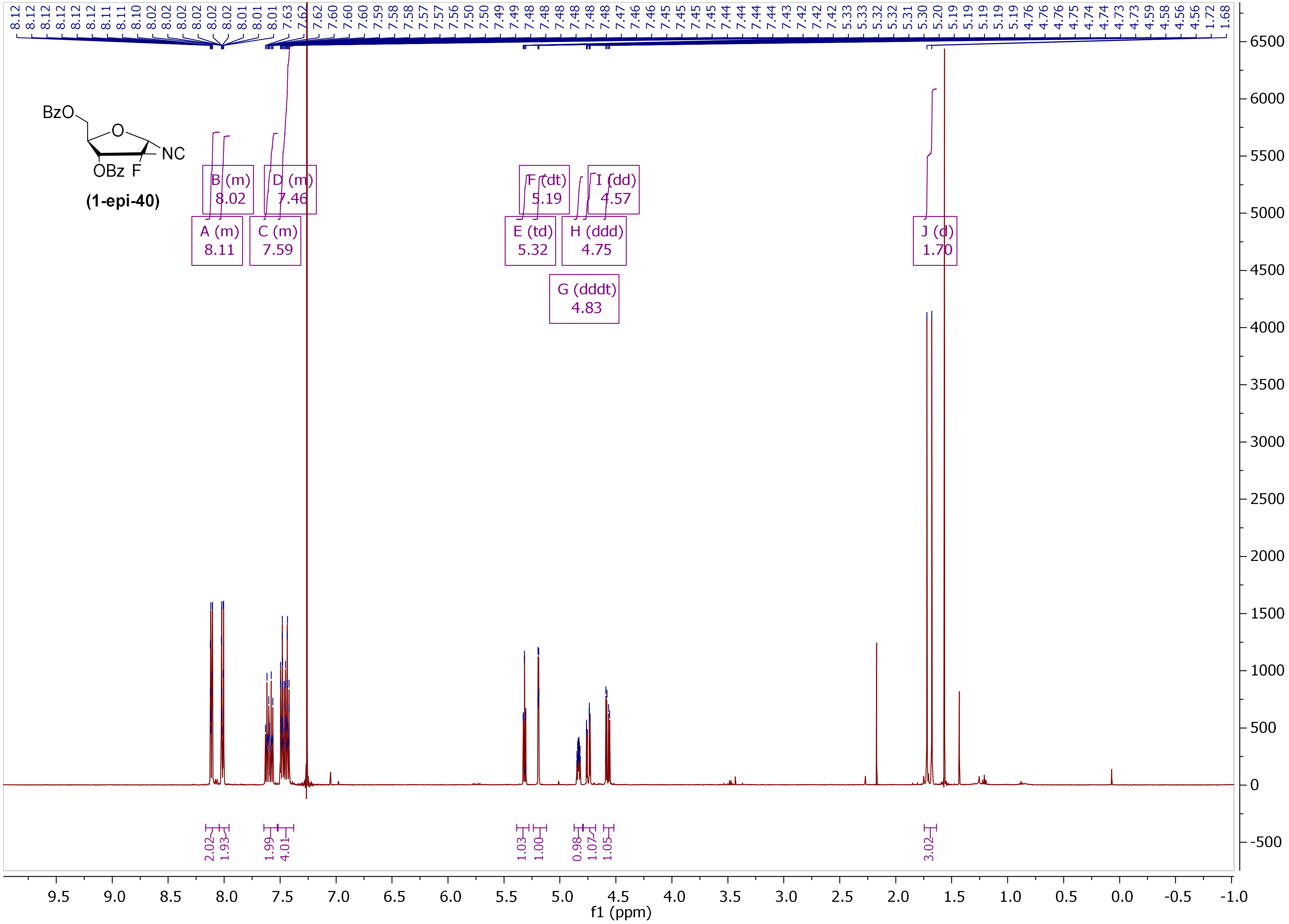

Figure S. 39 - ^{19}F NMR Spectra (377 MHz, $CDCl_3$) - 3,5-Di-*O*-benzoyl-2-deoxy-2-fluoro-2-methyl-α-*D*-ribofuranosyl isonitrile – (1-epi-**40**)

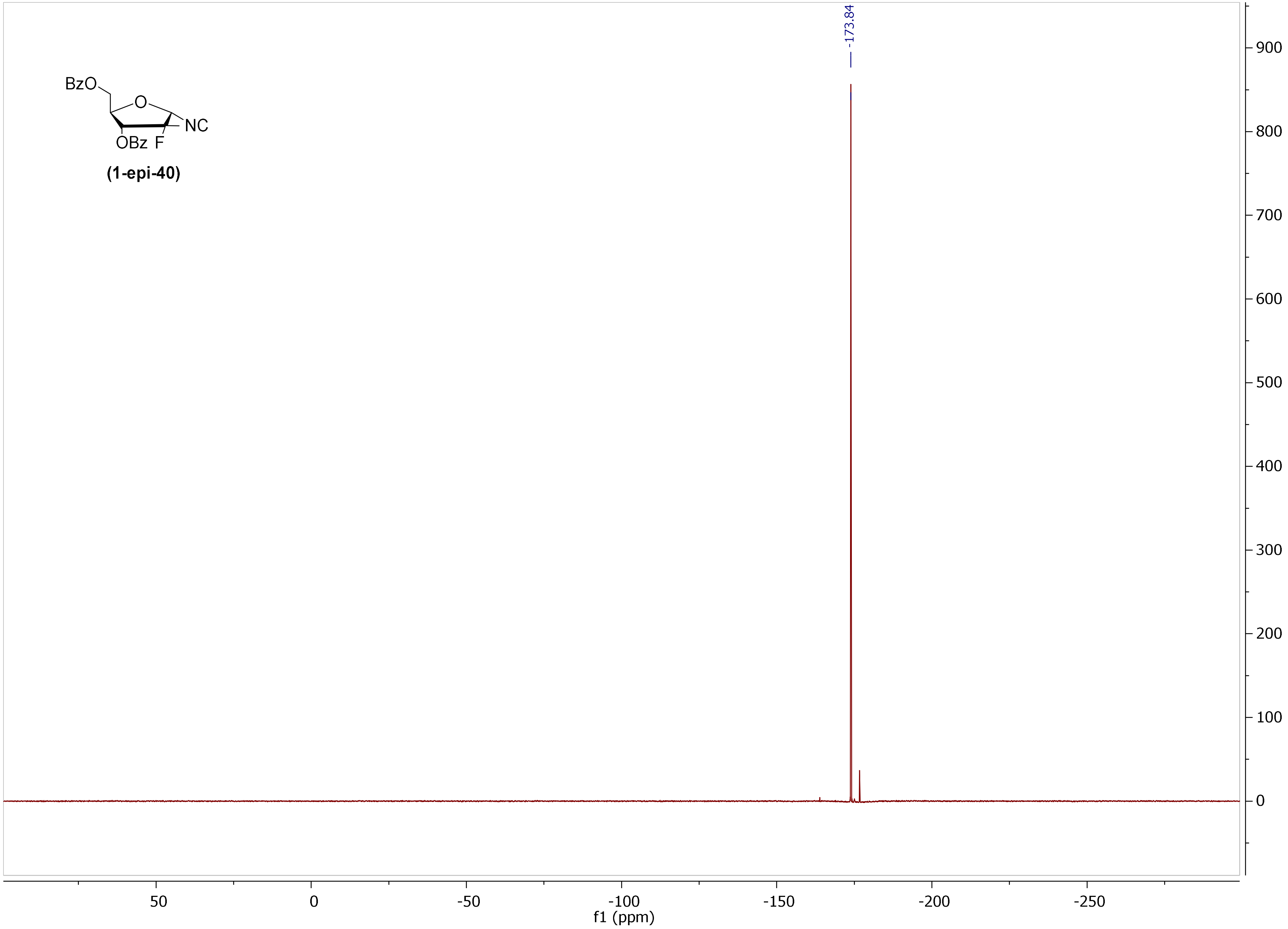

Figure S. 40 - ^{13}C NMR Spectra (101 MHz, $CDCl_3$) - 3,5-Di-*O*-benzoyl-2-deoxy-2-fluoro-2-methyl-α-*D*-ribofuranosyl isonitrile – (1-epi-**40**)

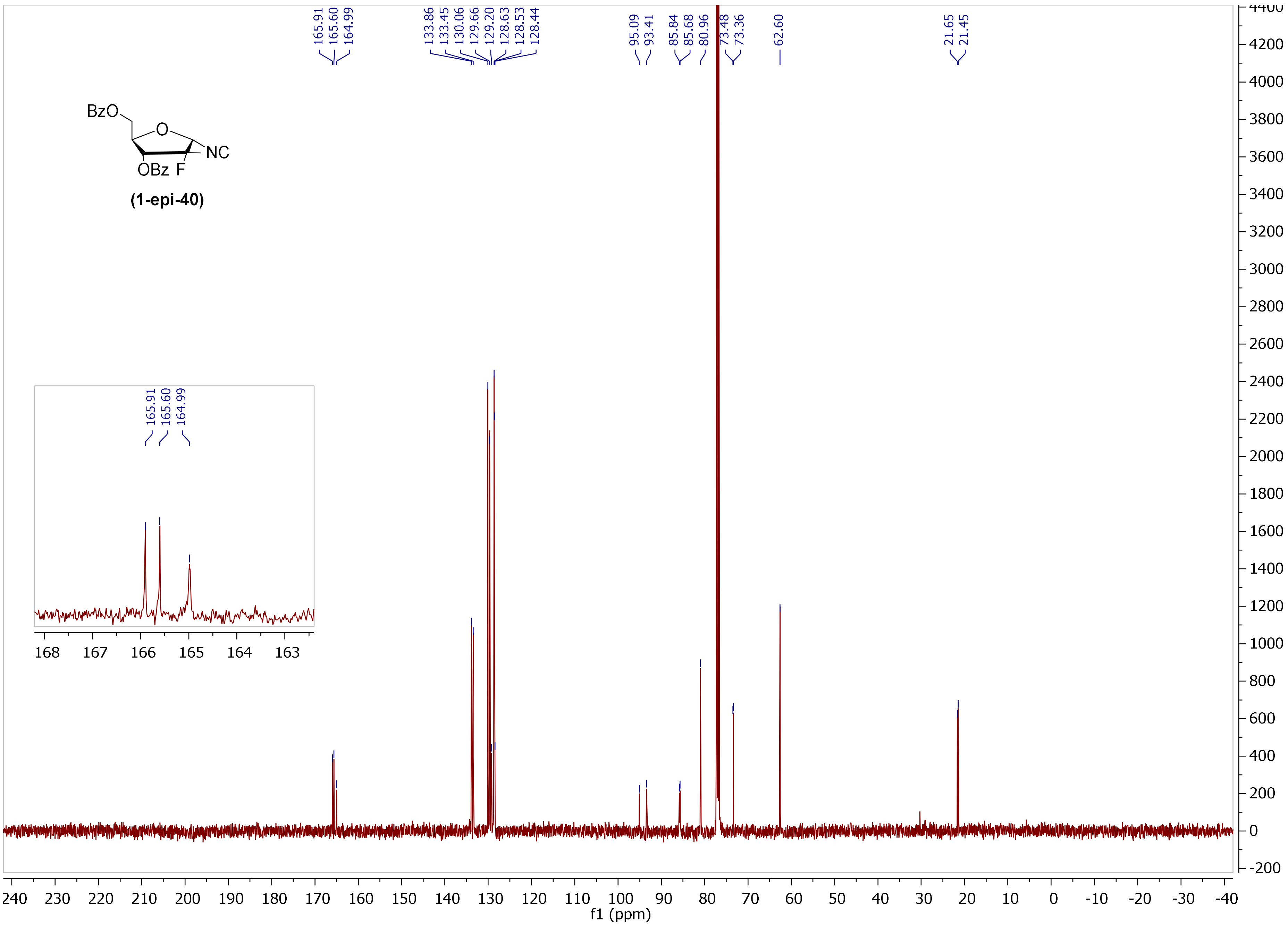

Figure S. 41 - ^{1}H-NMR Spectrum (400 MHz, $CDCl_3$) - 3,5-Di-*O*-benzoyl-2-deoxy-2-fluoro-α-*D*-arabinofuranosyl azide – (1-epi-**43**)

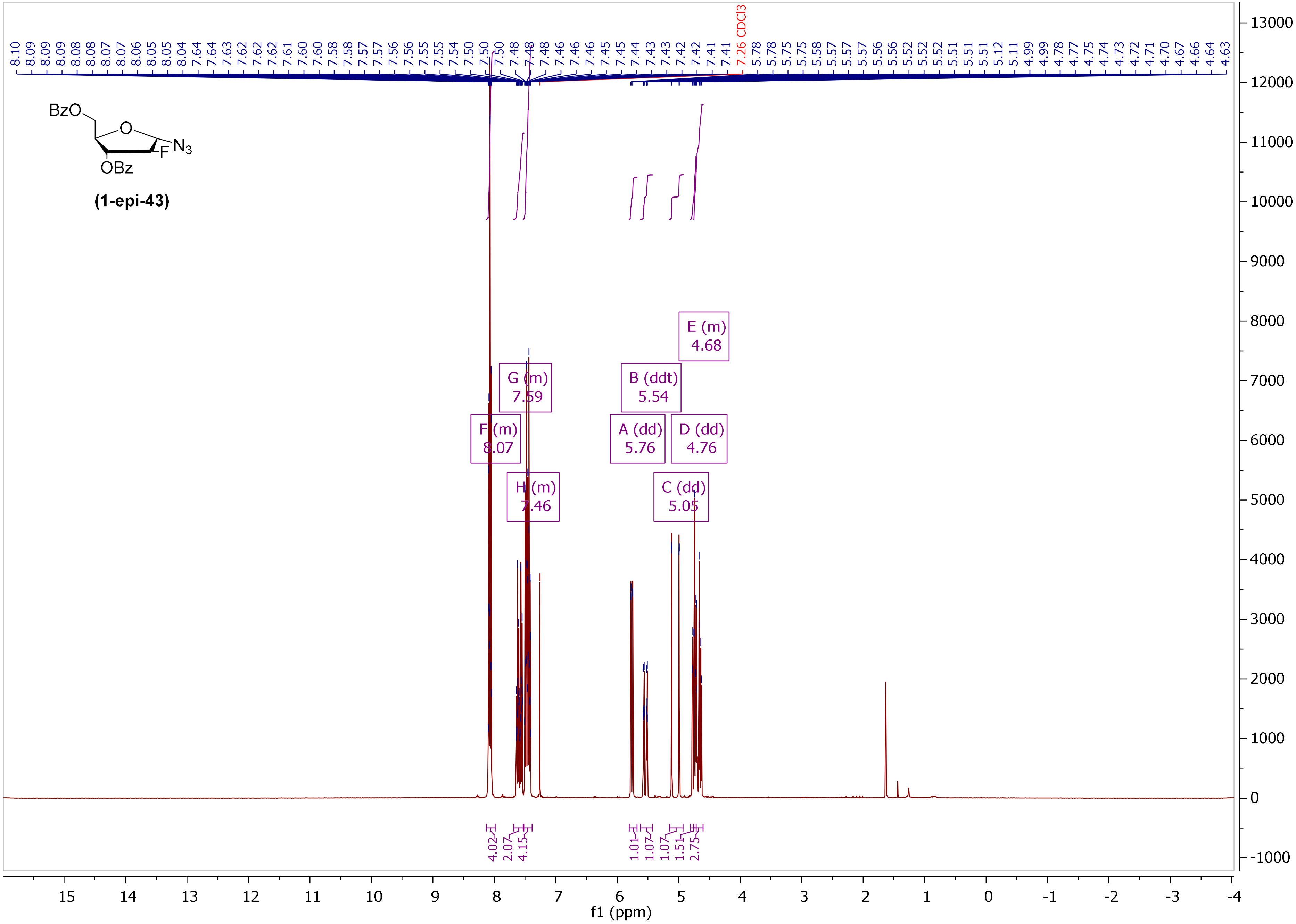

Figure S. 42 - ^{19}F NMR Spectra (377 MHz, $CDCl_3$) - 3,5-Di-*O*-benzoyl-2-deoxy-2-fluoro-α-*D*-arabinofuranosyl azide – (1-epi-**43**)

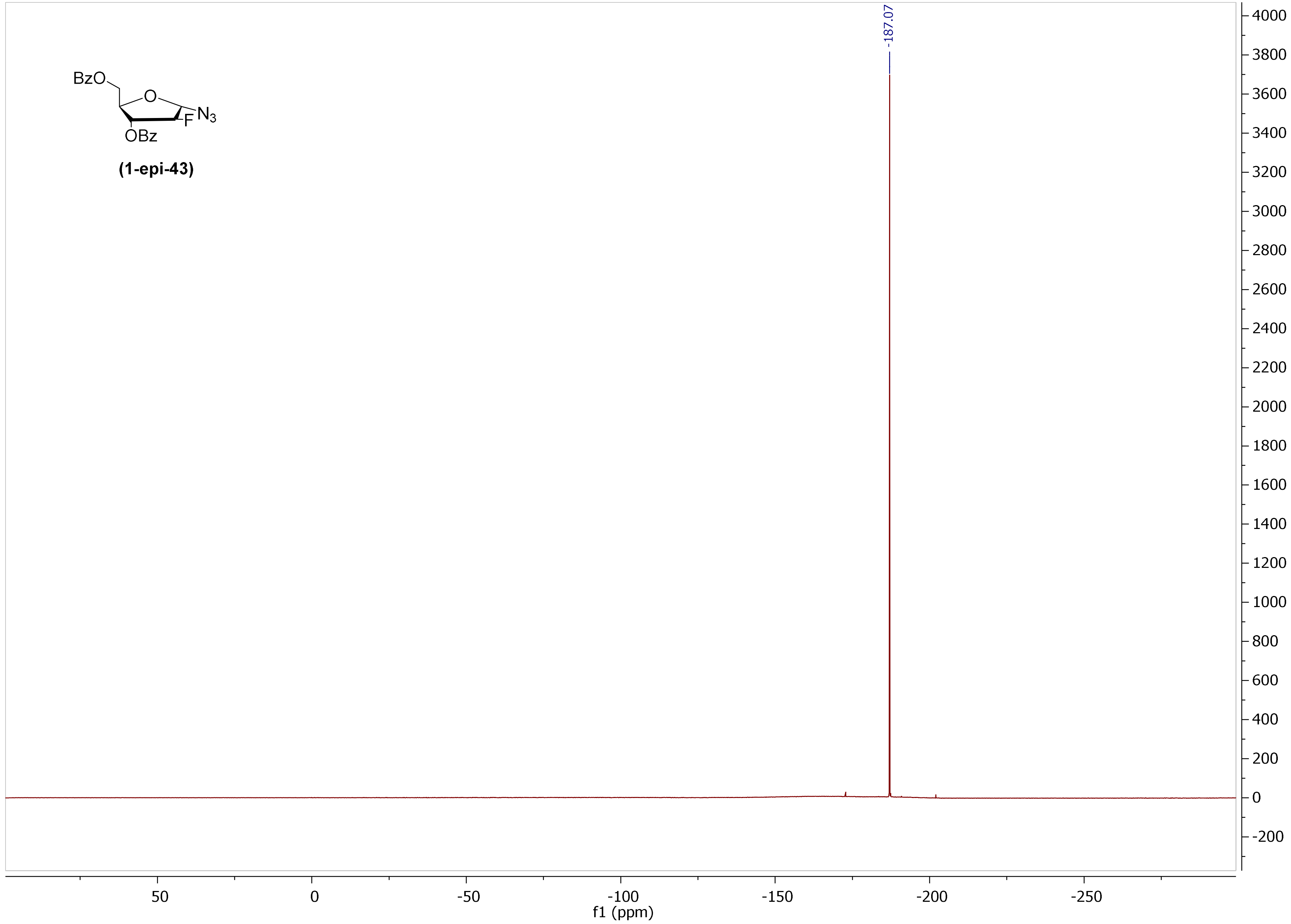

(1-epi-43)

Figure S. 43 - ^{13}C NMR Spectra (101 MHz, $CDCl_3$) - 3,5-Di-*O*-benzoyl-2-deoxy-2-fluoro-α-*D*-arabinofuranosyl azide – (1-epi-**43**)

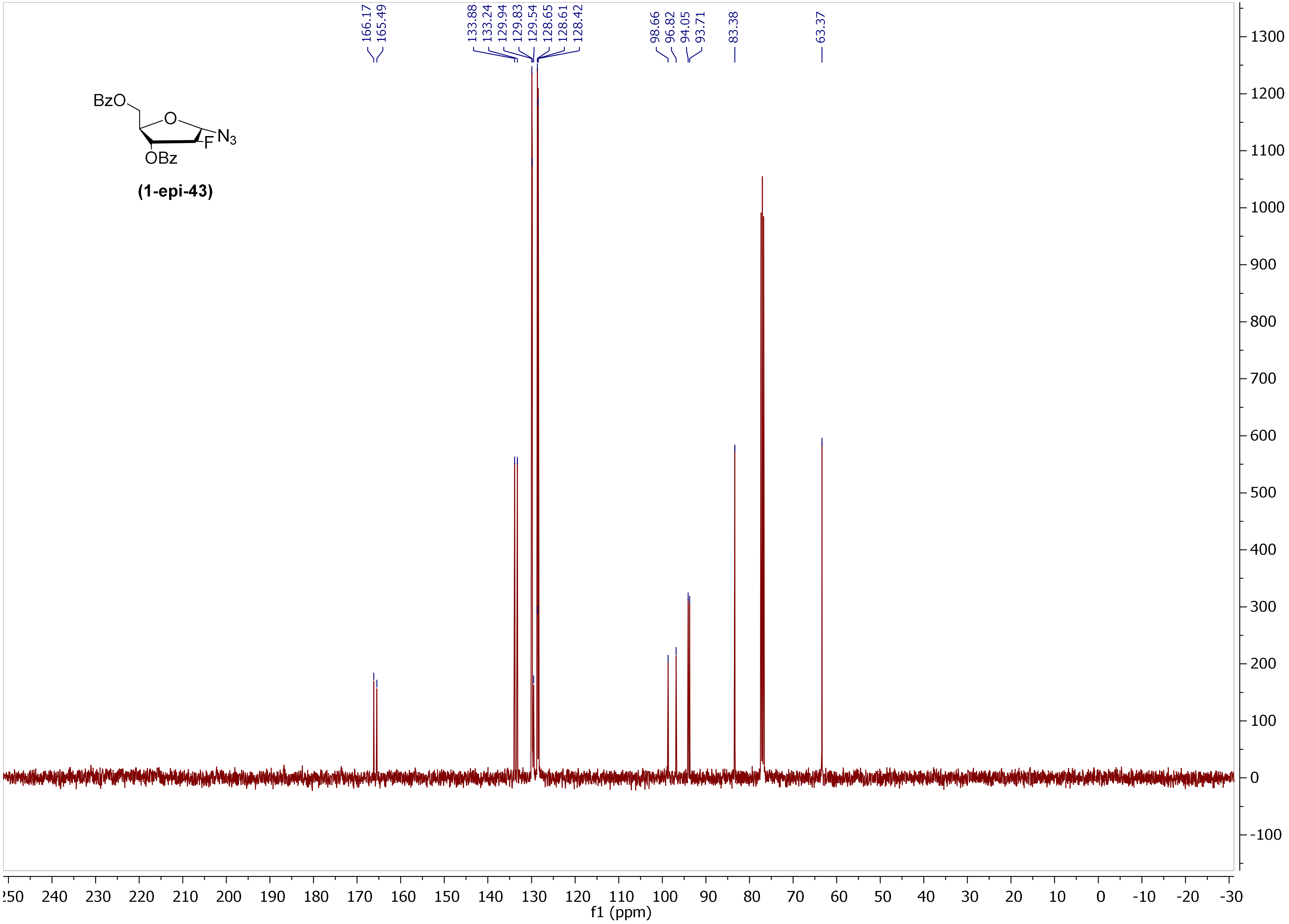

Figure S. 44 - ^{1}H-NMR Spectrum (400 MHz, $CDCl_3$) - 3,5-Di-*O*-benzoyl-2-deoxy-2-fluoro-β-*D*-arabinofuranosyl azide – **43**

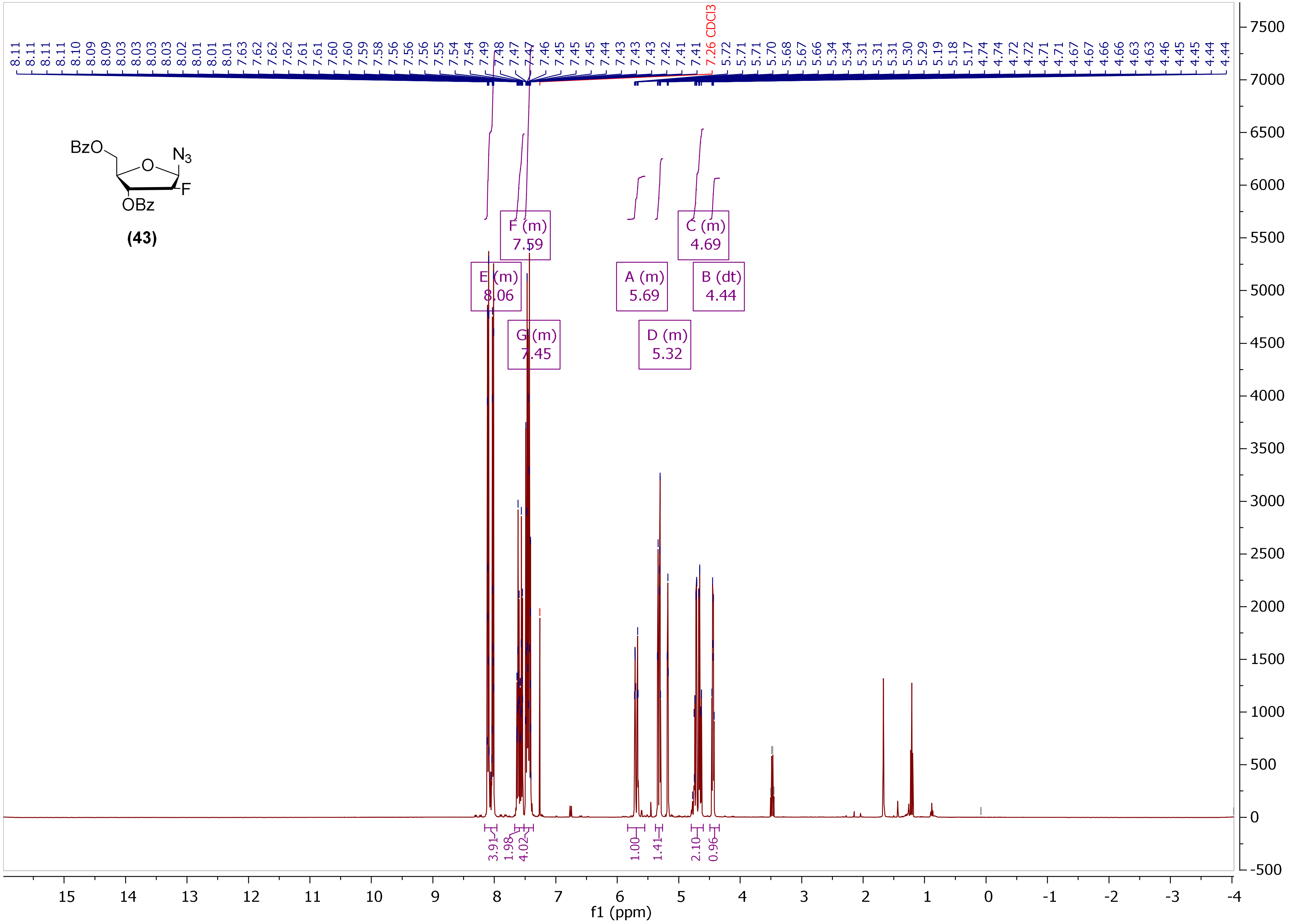

Figure S. 45 - ^{19}F NMR Spectra (377 MHz, $CDCl_3$) - 3,5-Di-*O*-benzoyl-2-deoxy-2-fluoro-β-*D*-arabinofuranosyl azide – **43**

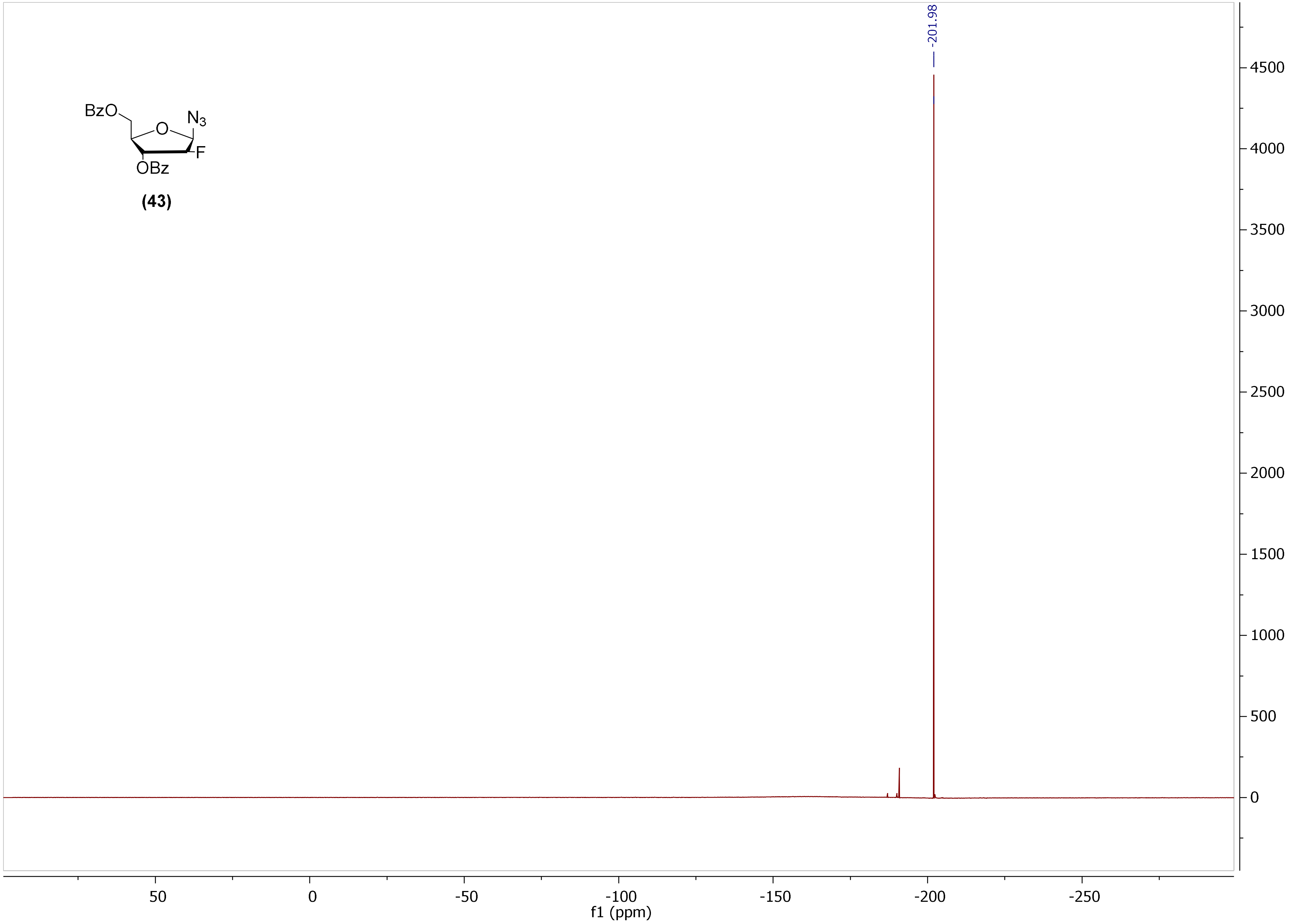

Figure S. 46 - ^{13}C NMR Spectra (101 MHz, $CDCl_3$) - 3,5-Di-*O*-benzoyl-2-deoxy-2-fluoro-β-*D*-arabinofuranosyl azide – **43**

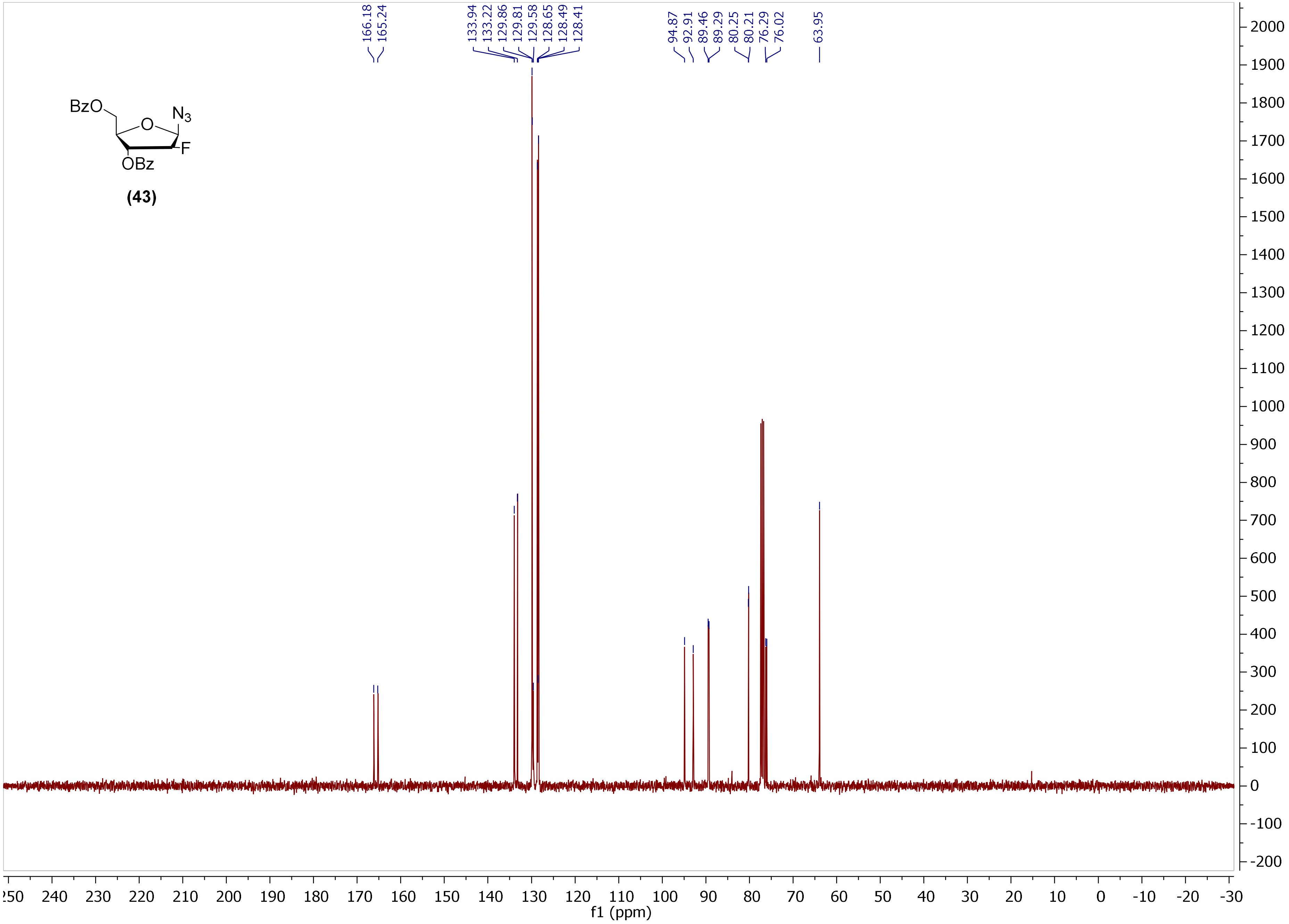

Figure S. 47- ^{1}H-NMR Spectrum (400 MHz, $CDCl_3$) - 3,5-Di-*O*-benzoyl-2-deoxy-2-fluoro-β-*D*-arabinofuranosyl isonitrile – **45**

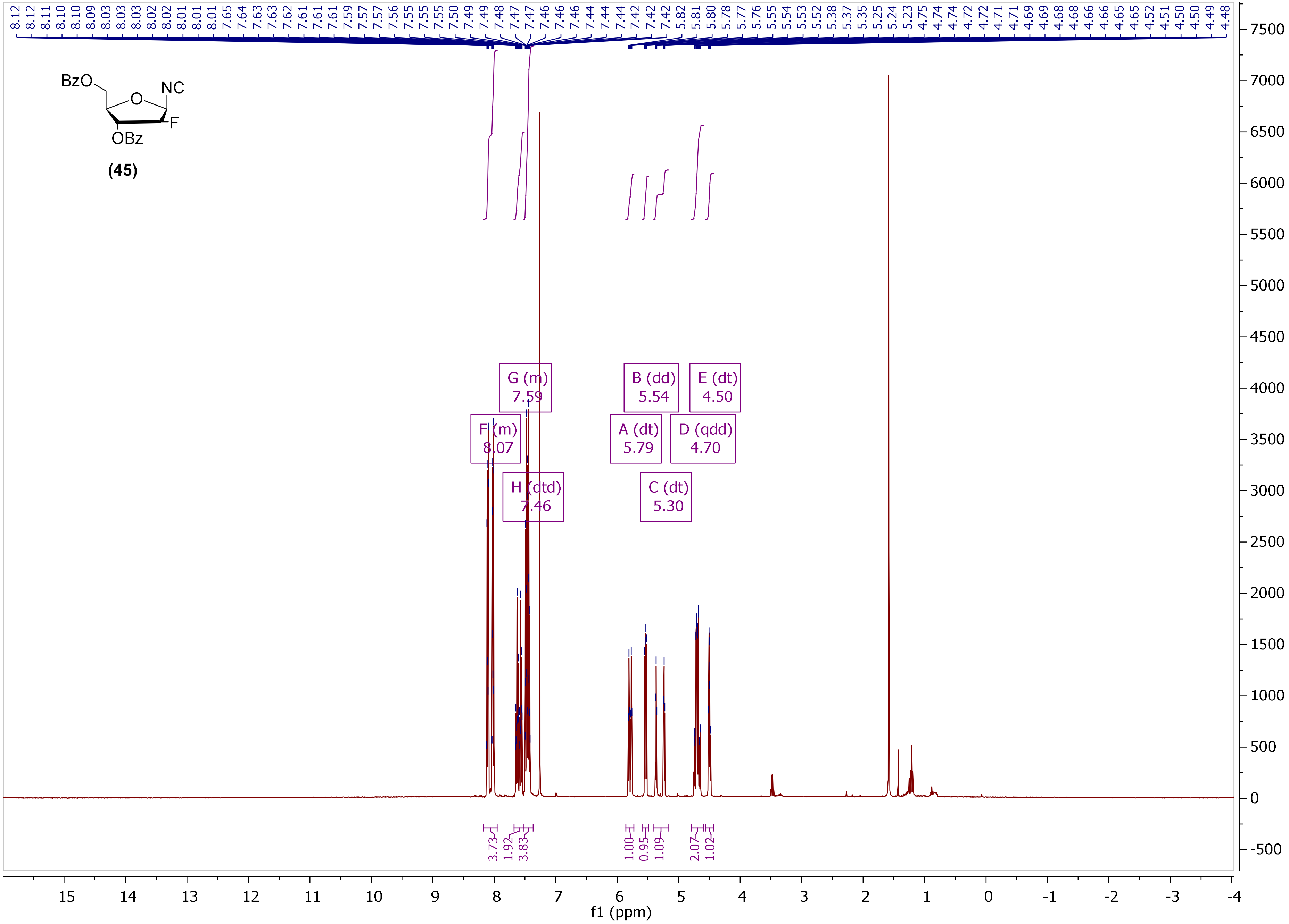

Figure S. 48 - ^{19}F NMR Spectra (377 MHz, $CDCl_3$) - 3,5-Di-*O*-benzoyl-2-deoxy-2-fluoro-β-*D*-arabinofuranosyl isonitrile –**45**

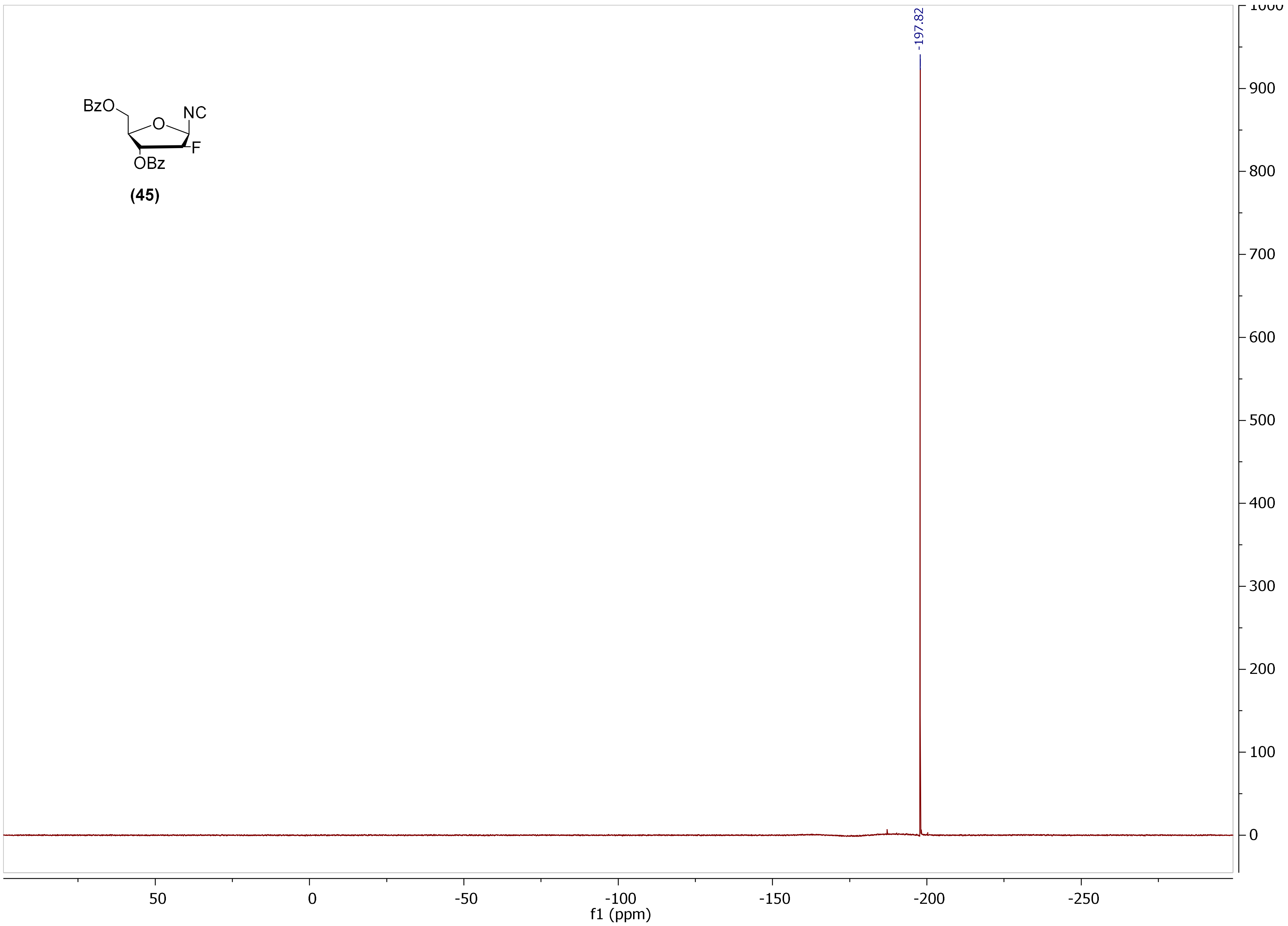

Figure S. 49 - ^{13}C NMR Spectra (101 MHz, $CDCl_3$) - 3,5-Di-*O*-benzoyl-2-deoxy-2-fluoro-β-*D*-arabinofuranosyl isonitrile – **45**

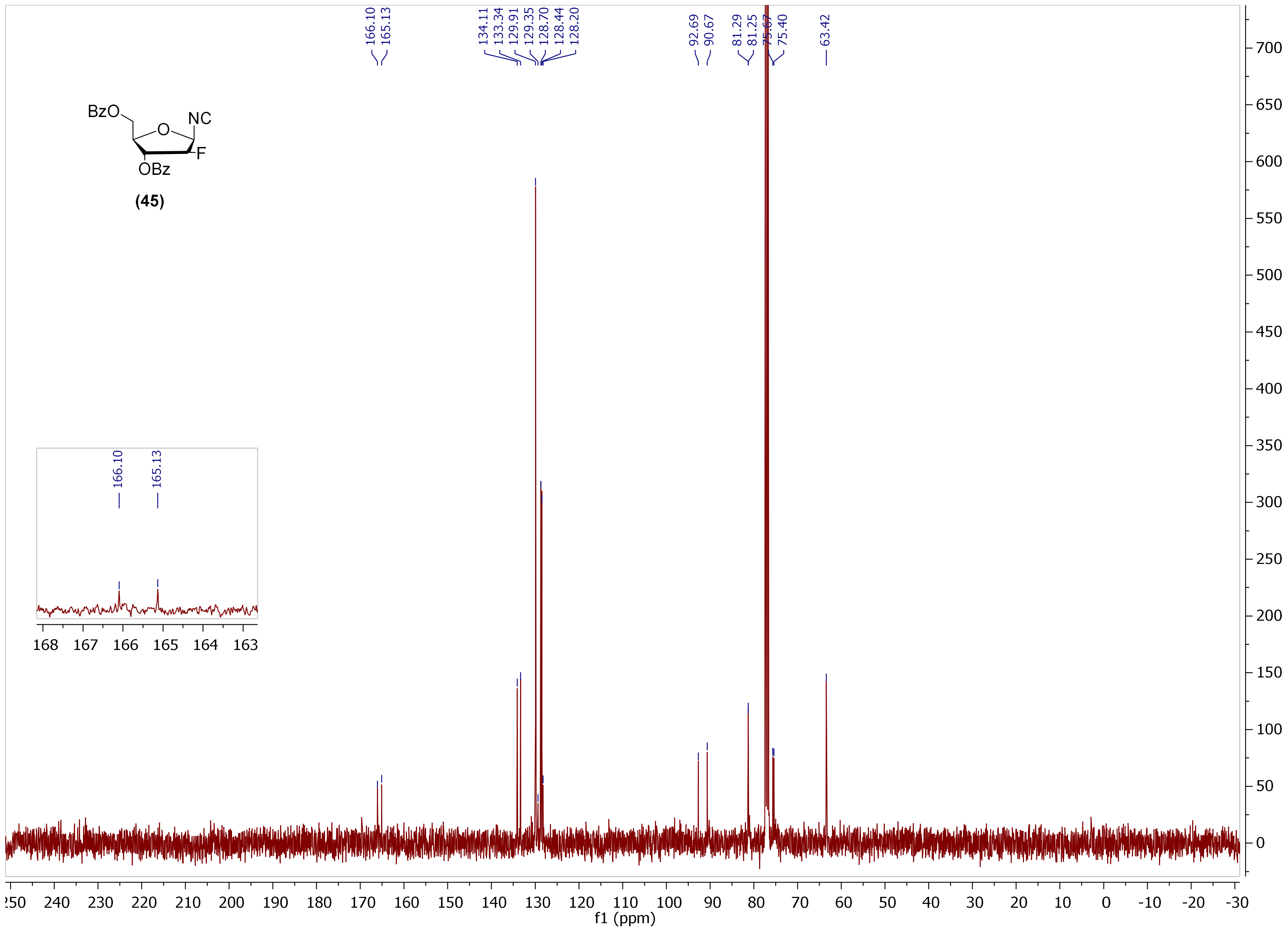

Figure S. 50 - ^{1}H-NMR Spectrum (400 MHz, $CDCl_3$) - 3,5-Di-*O*-benzoyl-2-deoxy-2-fluoro-α-*D*-arabinofuranosyl isonitrile – (1-epi-**45**)

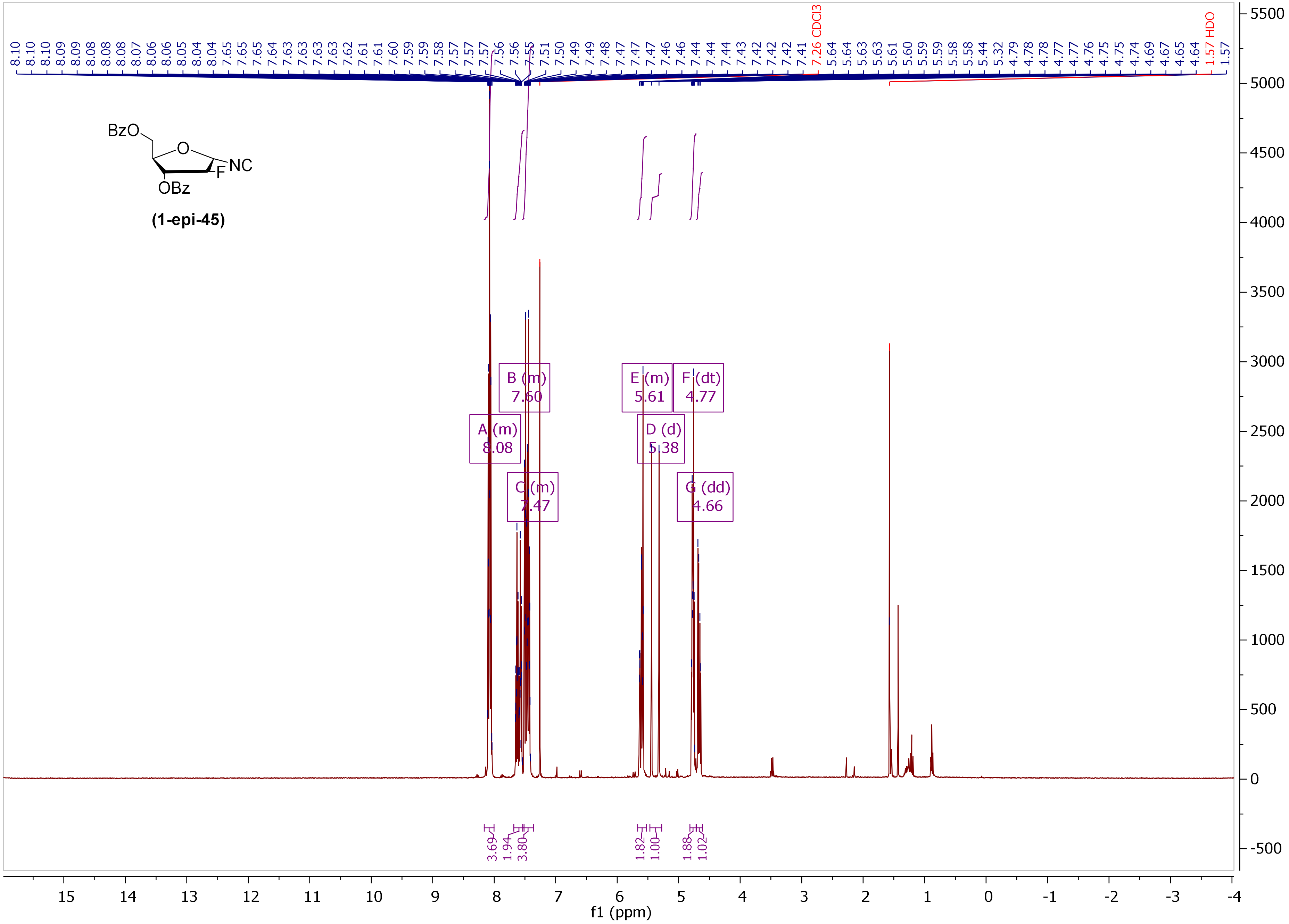

Figure S. 51 - ^{19}F NMR Spectra (377 MHz, $CDCl_3$) - 3,5-Di-*O*-benzoyl-2-deoxy-2-fluoro-α-*D*-arabinofuranosyl isonitrile – (1-epi-**45**)

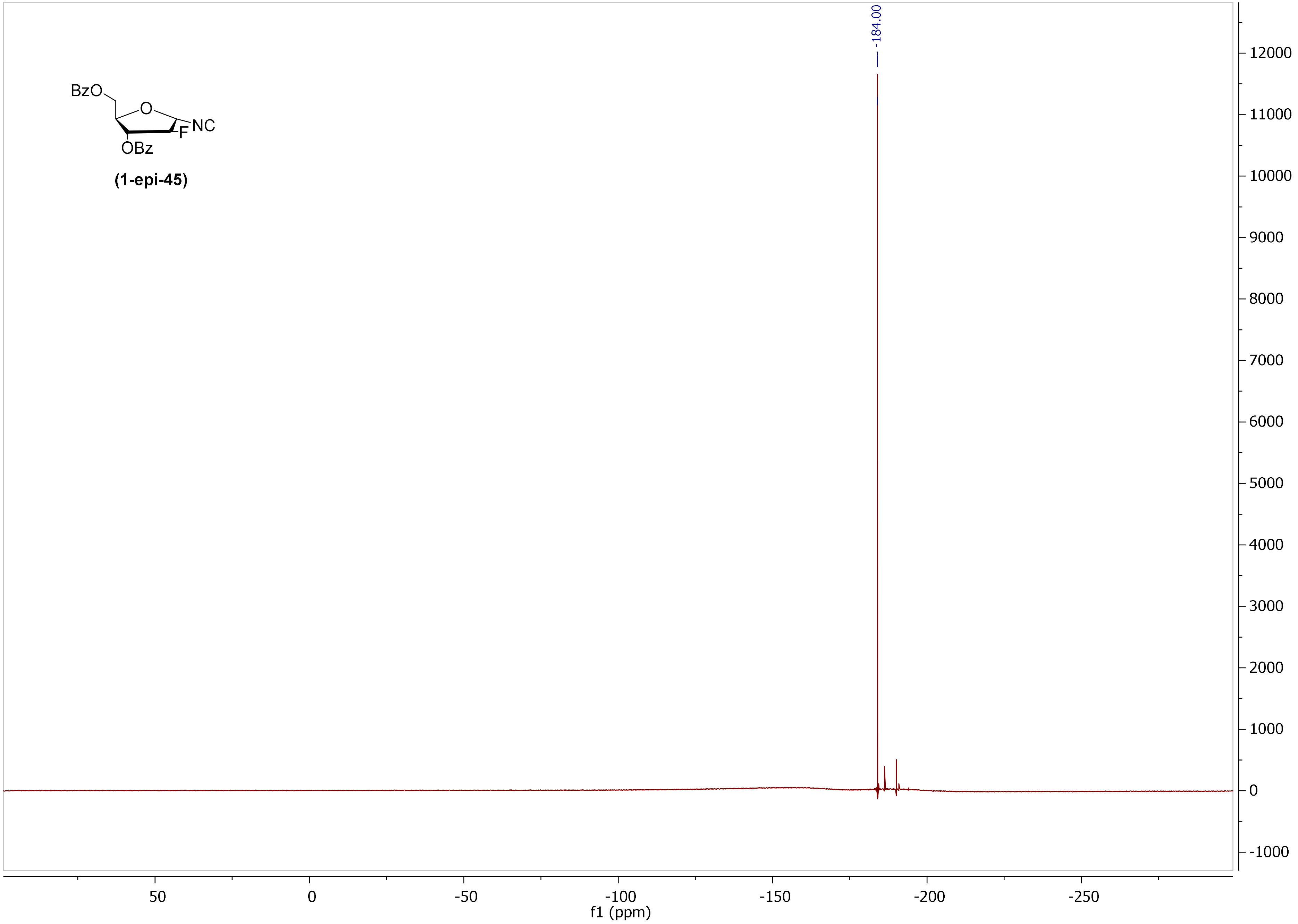

Figure S. 52 - ^{13}C NMR Spectra (101 MHz, $CDCl_3$) - 3,5-Di-*O*-benzoyl-2-deoxy-2-fluoro-α-*D*-arabinofuranosyl isonitrile – (1-epi-**45**)

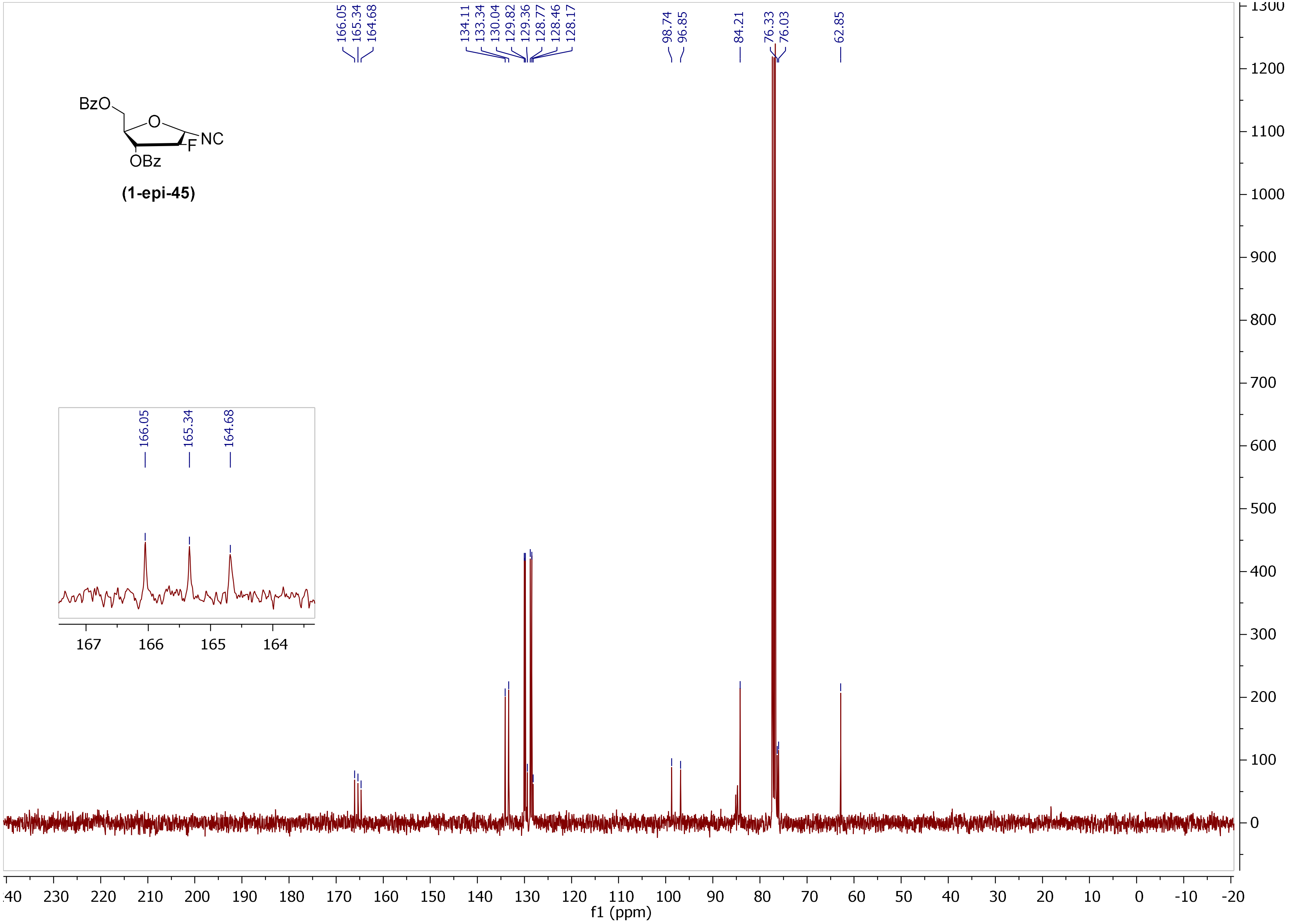

Figure S. 53 - ^{1}H-NMR Spectrum (400 MHz, $CDCl_3$) - 5-((Cyclohexylamino)methylene)-2,2-dimethyl-1,3-dioxane-4,6-dione – **15a**

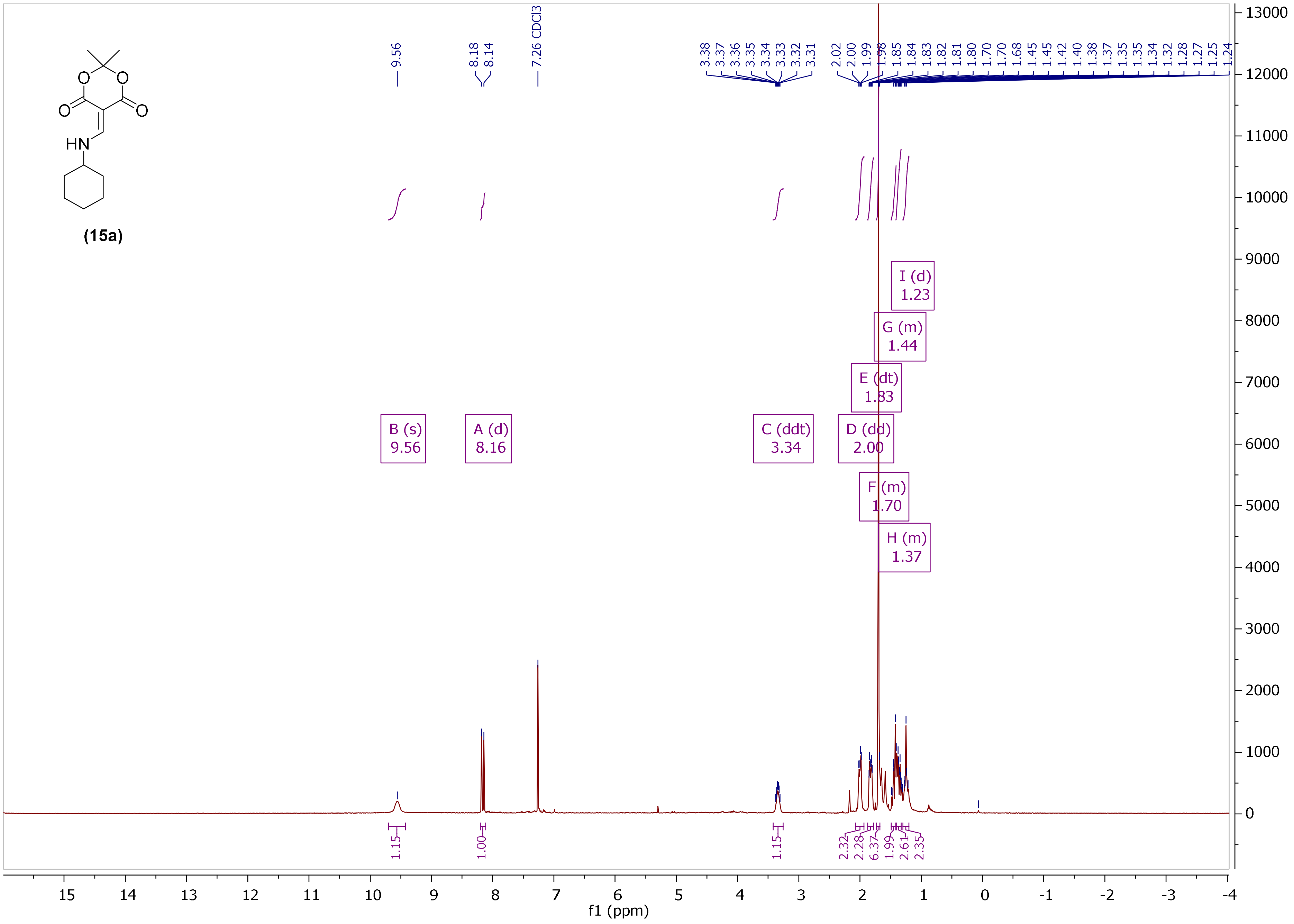

Figure S. 54 - ^{13}C NMR Spectra (101 MHz, $CDCl_3$) - 5-((Cyclohexylamino)methylene)-2,2-dimethyl-1,3-dioxane-4,6-dione – **15a**

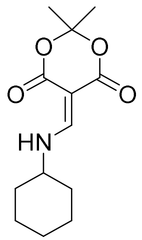

(15a)

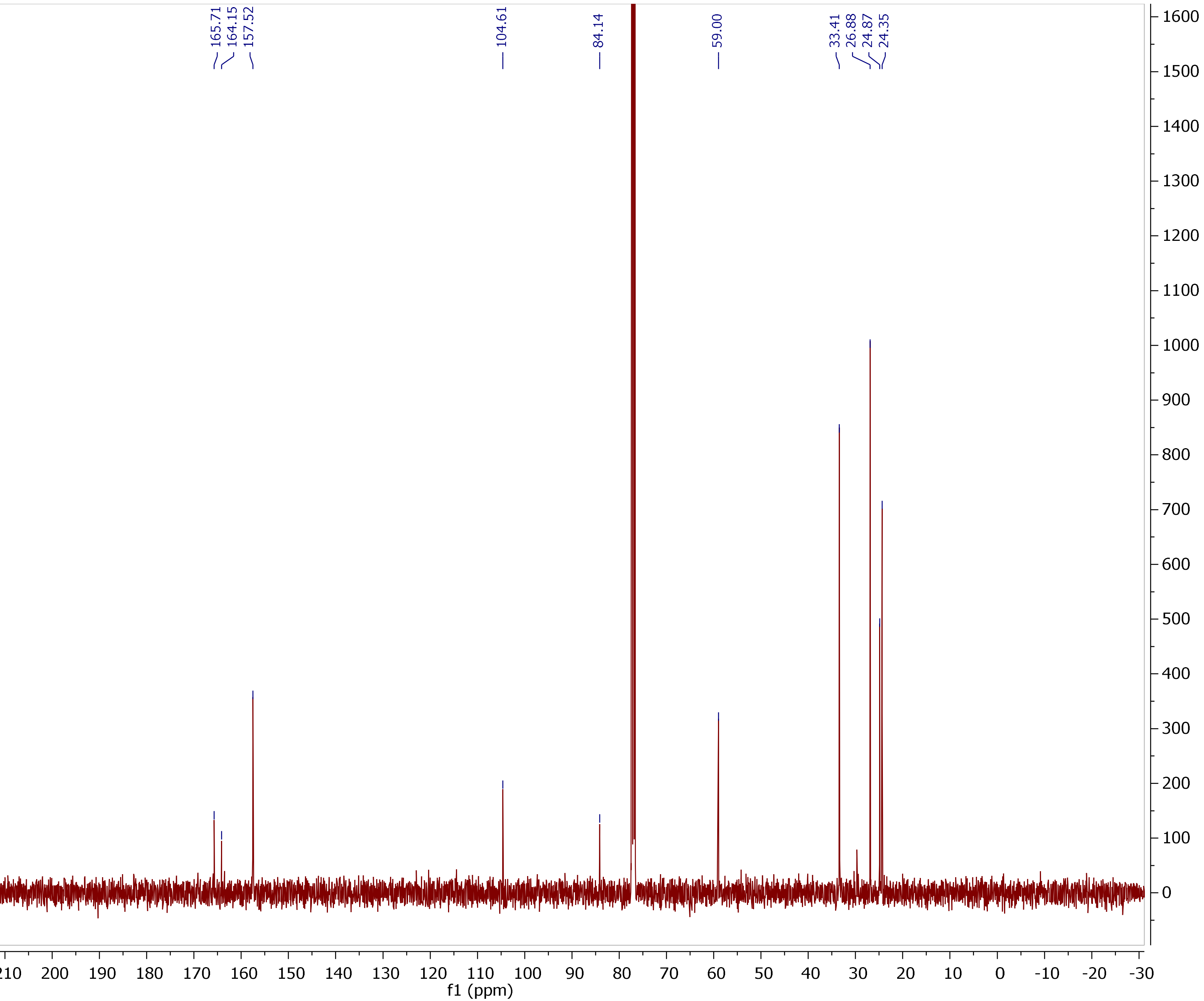

Figure S. 55 - ^{1}H-NMR Spectrum (400 MHz, $CDCl_3$) - 5-(5-*O*-acetyl-2,3-di-*O*-iso-propylidene-β-*D*-ribofuranosylamino-methylene)-2,2-dimethyl-1,3-dioxa-4,6-dione - **15b**

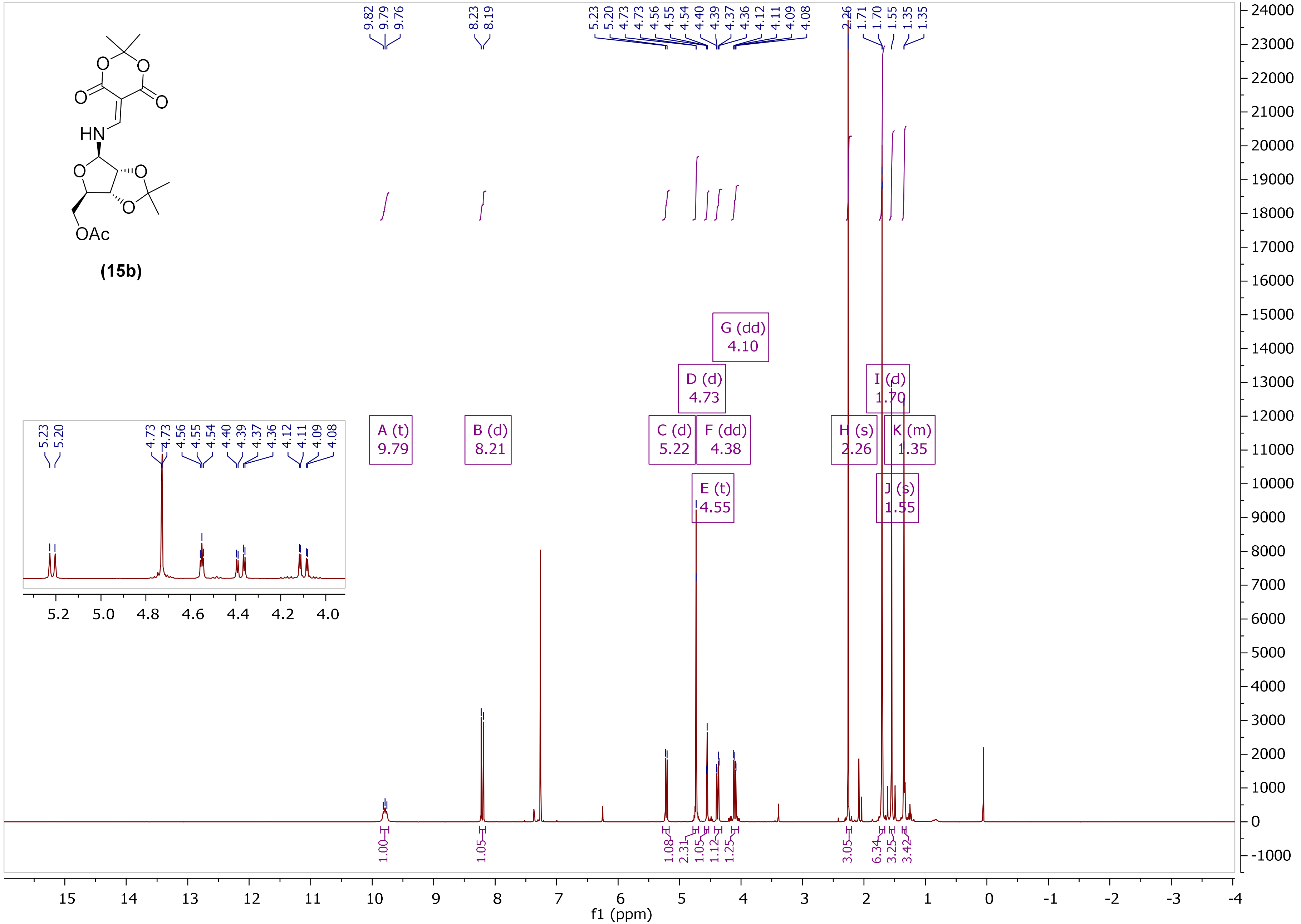

Figure S. 56 - ^{13}C NMR Spectra (101 MHz, $CDCl_3$) - 5-(5-*O*-acetyl-2,3-di-*O*-iso-propylidene-β-*D*-ribofuranosylamino-methylene)-2,2-dimethyl-1,3-dioxa-4,6-dione - **15b**

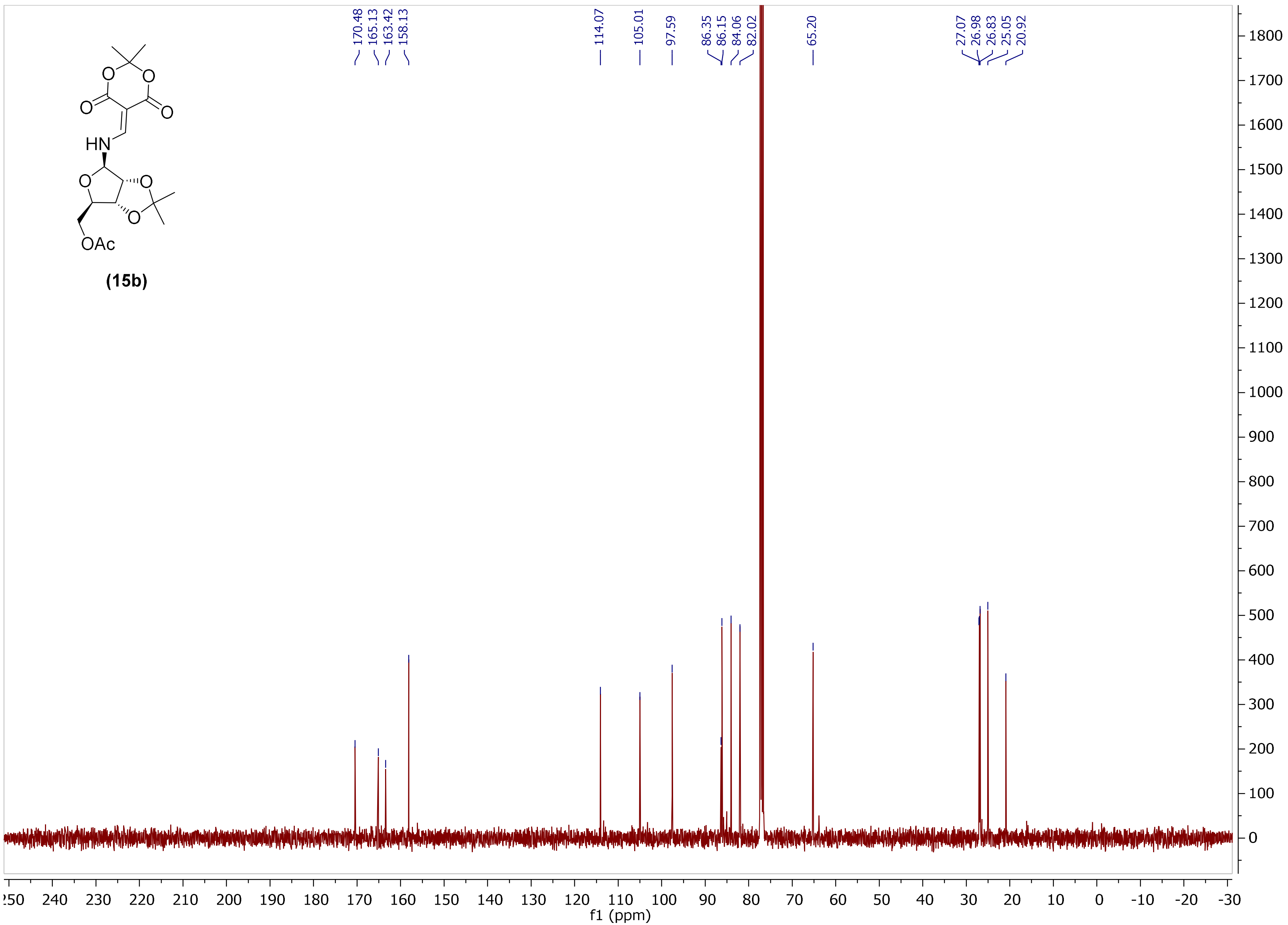

Figure S. 57 - ^{1}H-NMR Spectrum (400 MHz, $CDCl_3$) - 5-(3,5-di-*O*-benzoyl-2-deoxy-2-fluoro-2-methyl-β-*D*-ribofuranosylamino-methylene)-2,2-dimethyl-1,3-dioxa-4,6-dione - **15c**

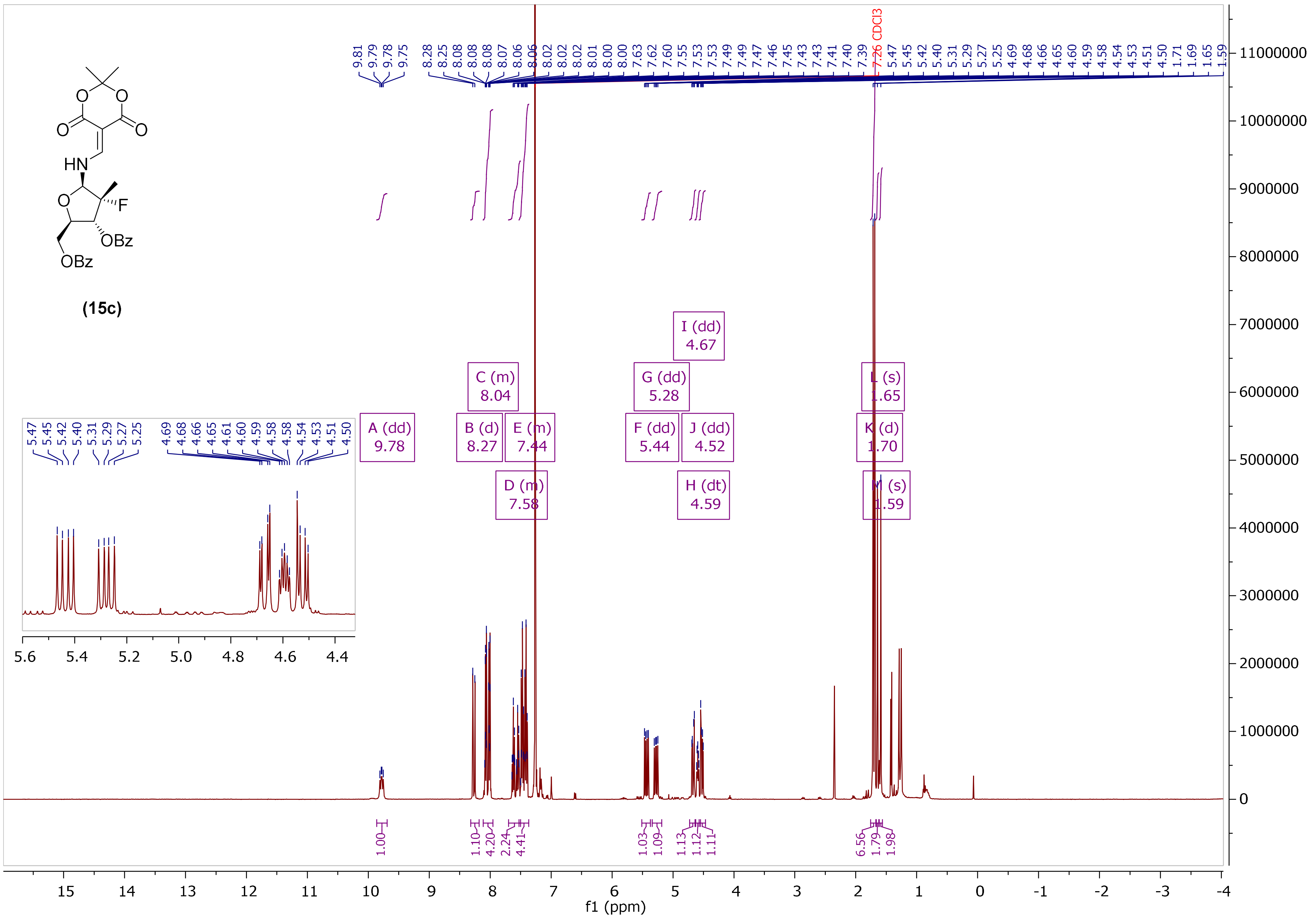

Figure S. 58 - ^{19}F NMR Spectra (377 MHz, $CDCl_3$) - 5-(3,5-di-*O*-benzoyl-2-deoxy-2-fluoro-2-methyl-β-*D*-ribofuranosylamino-methylene)-2,2-dimethyl-1,3-dioxa-4,6-dione **- 15c**

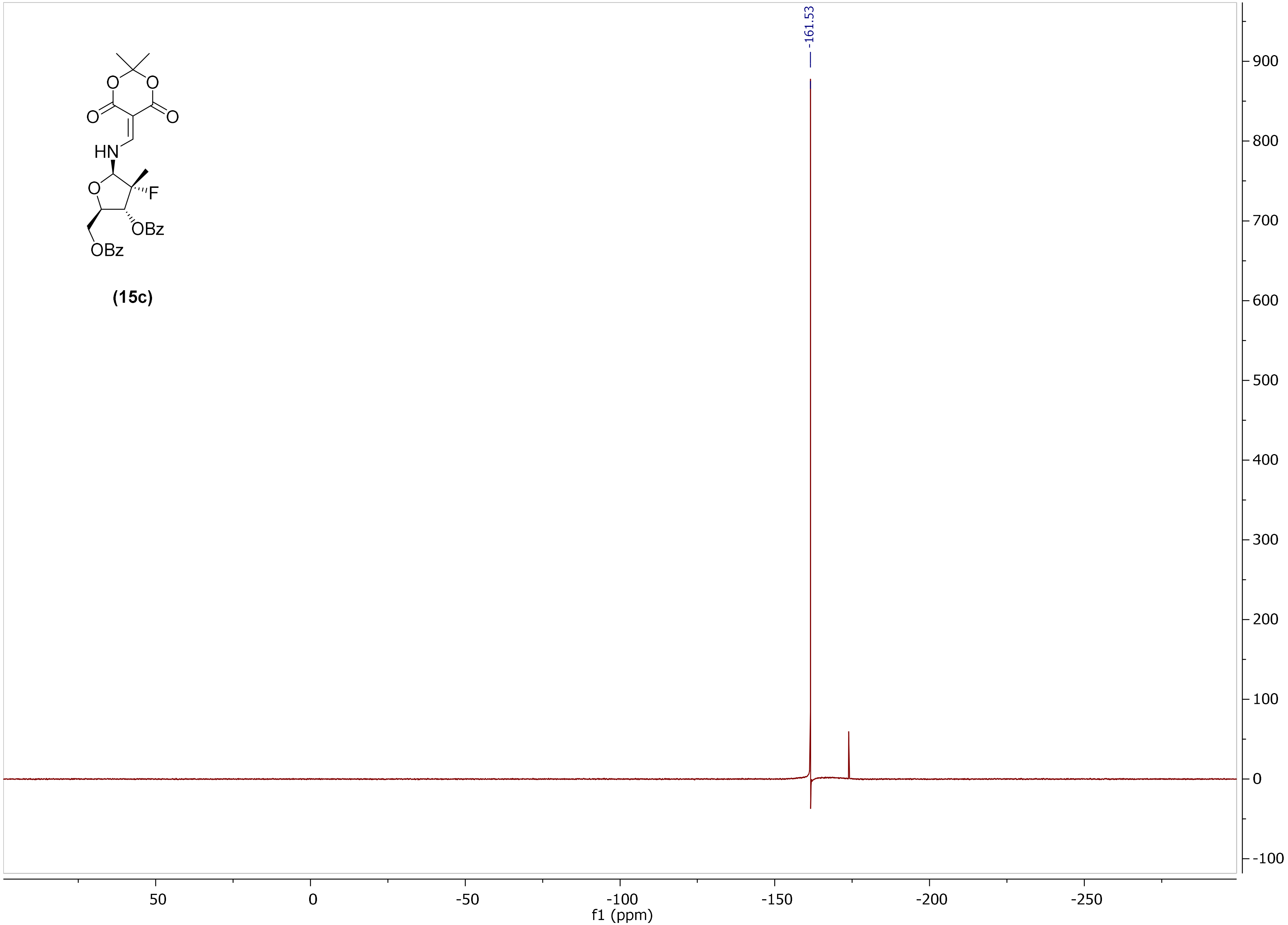

Figure S. 59 - ^{13}C NMR Spectra (101 MHz, $CDCl_3$) - 5-(3,5-di-*O*-benzoyl-2-deoxy-2-fluoro-2-methyl-β-*D*-ribofuranosylamino-methylene)-2,2-dimethyl-1,3-dioxa-4,6-dione - **15c**

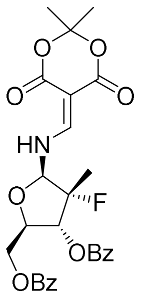

(15c)

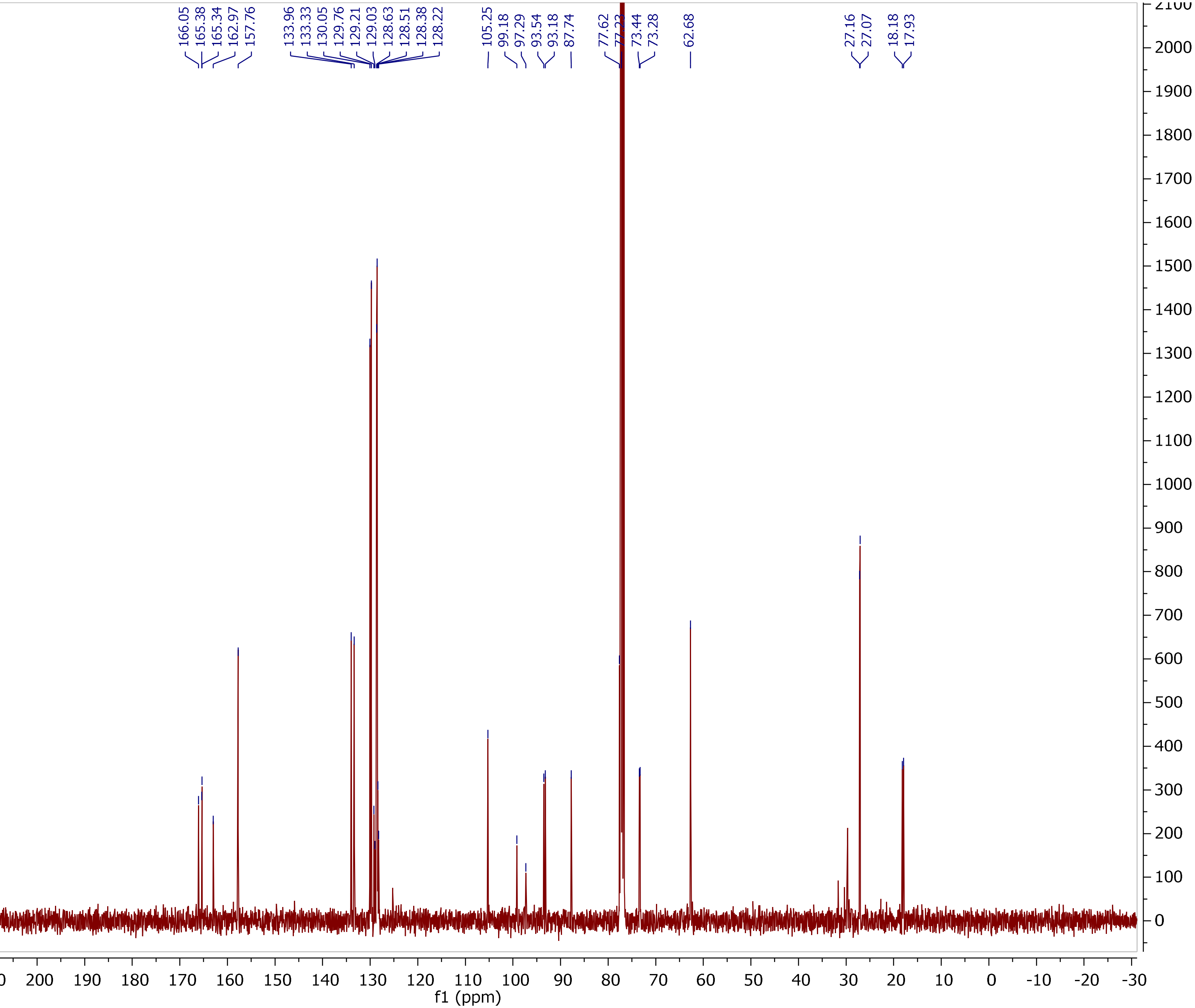

Figure S. 60 - ^{1}H NMR Spectra (400 MHz- $CDCl_3$) - 5-(3,5-di-*O*-benzoyl-2-deoxy-2-fluoro-2-methyl-α-*D*-ribofuranosylamino-methylene)-2,2-dimethyl-1,3-dioxa-4,6-dione **–** (1-epi-**15c)**

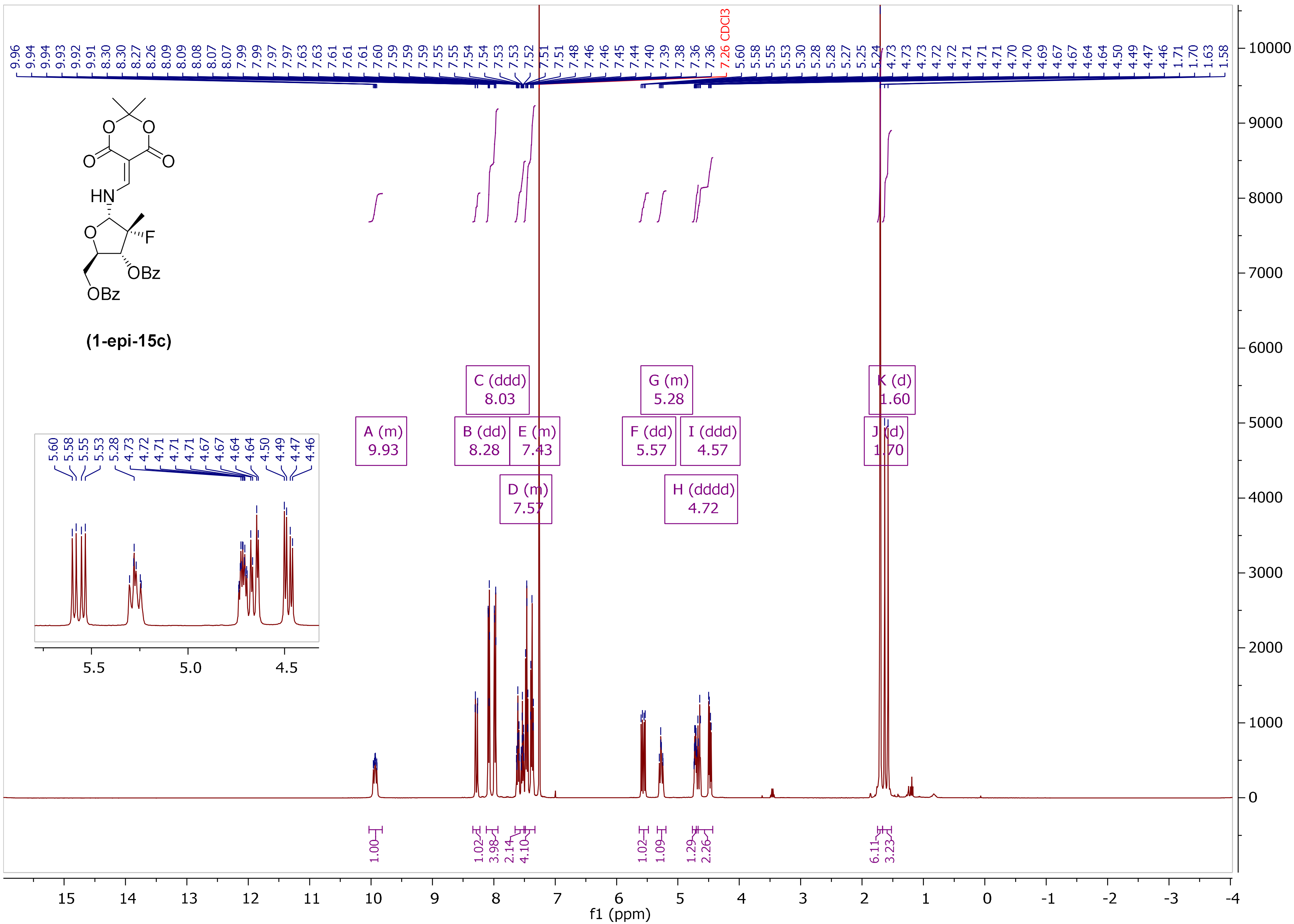

Figure S. 61 - ^{19}F NMR Spectra (377 MHz, $CDCl_3$) - 5-(3,5-di-*O*-benzoyl-2-deoxy-2-fluoro-2-methyl-α-*D*-ribofuranosylamino-methylene)-2,2-dimethyl-1,3-dioxa-4,6-dione **–** (1-epi-**15c)**

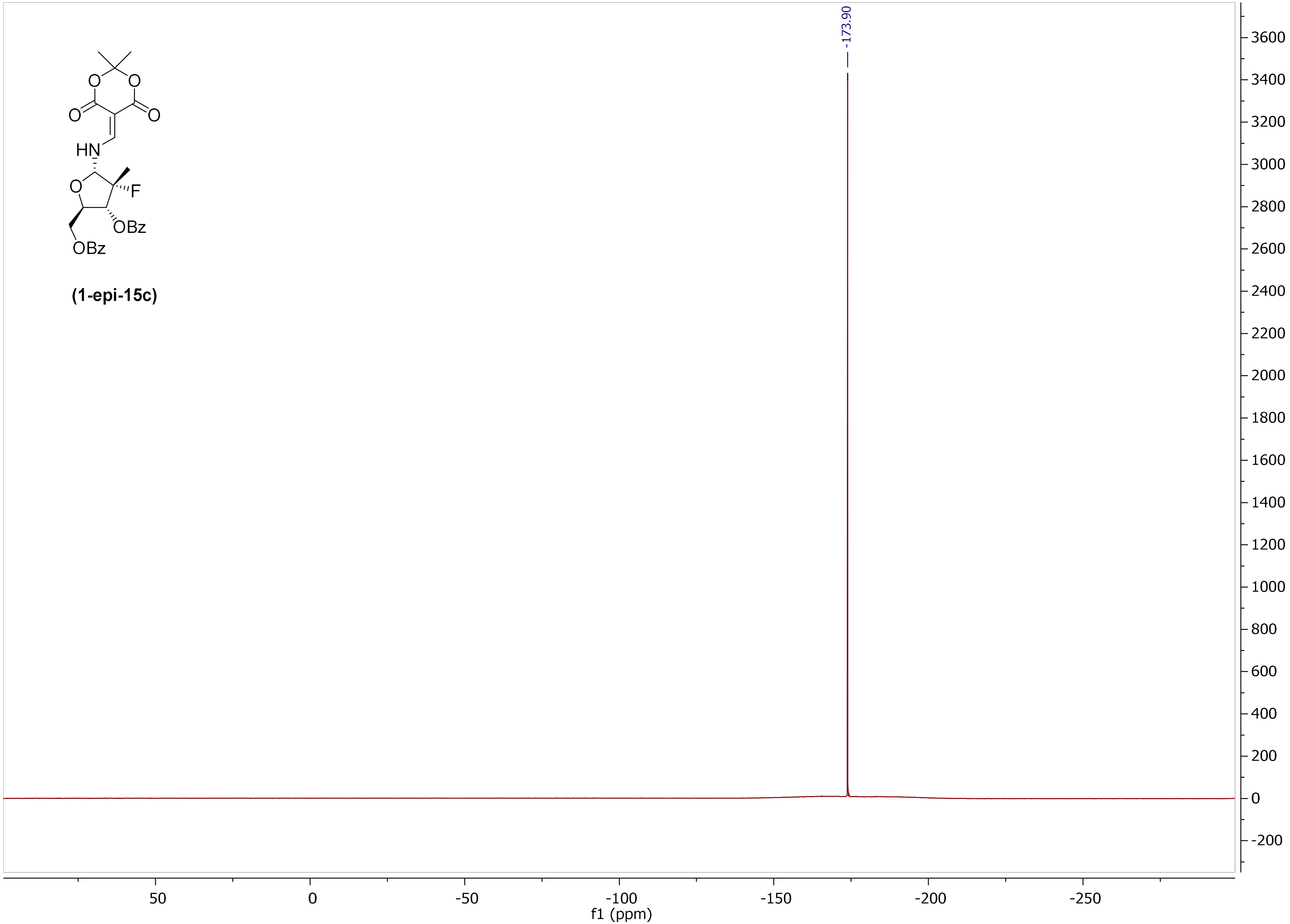

Figure S. 62 - ^{13}C NMR Spectra (101 MHz, $CDCl_3$) - 5-(3,5-di-*O*-benzoyl-2-deoxy-2-fluoro-2-methyl-α-*D*-ribofuranosylamino-methylene)-2,2-dimethyl-1,3-dioxa-4,6-dione **–** (1-epi-**15c)**

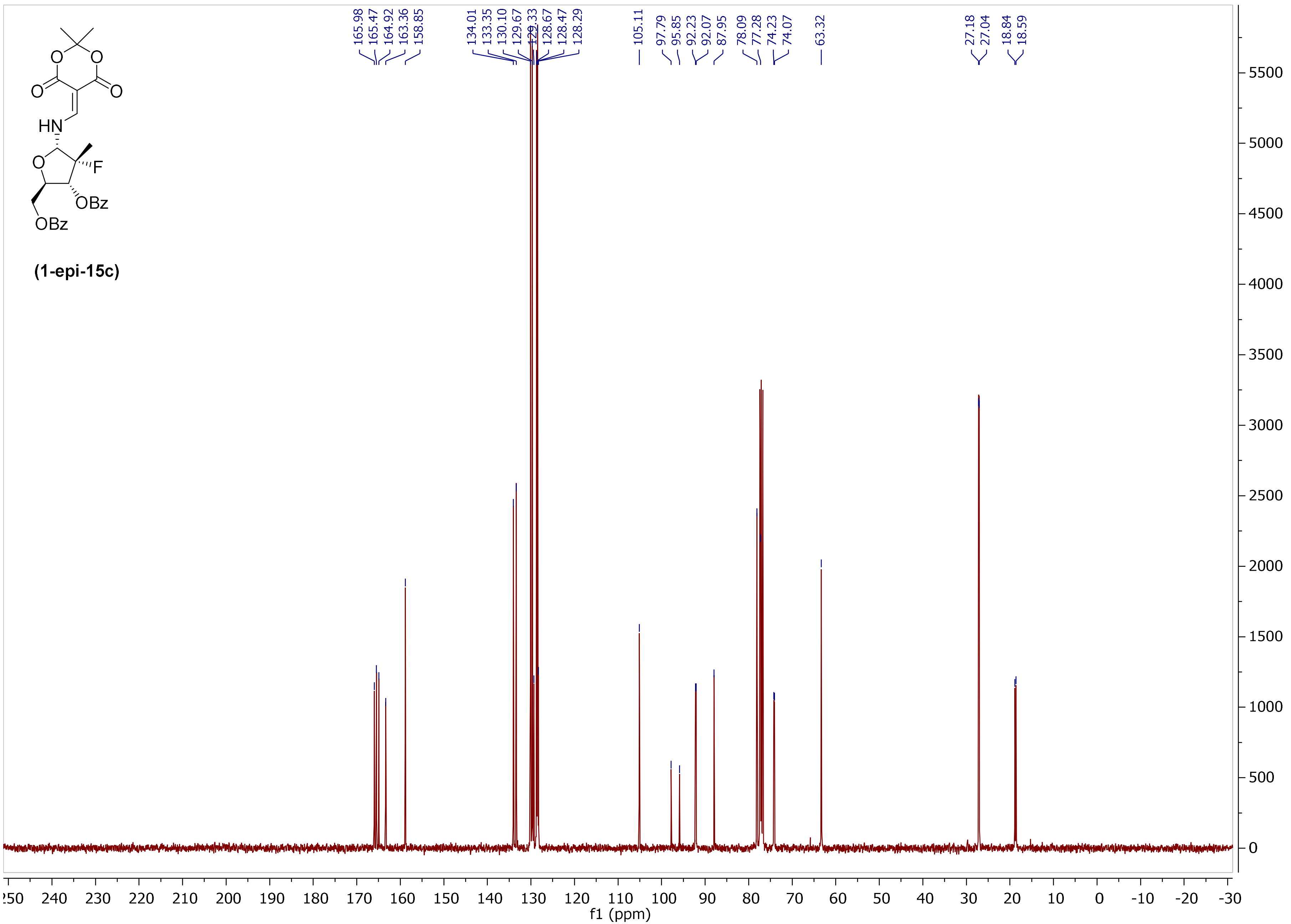

Figure S. 63 - ^{1}H-NMR Spectrum (400 MHz, $CDCl_3$) - 5-(3,5-Di-*O*-benzoyl-2-deoxy-2,2-difluoro-β-*D*-ribofuranosylamino-methylene)-2,2-dimethyl-1,3-dioxa-4,6-dione - **15d**

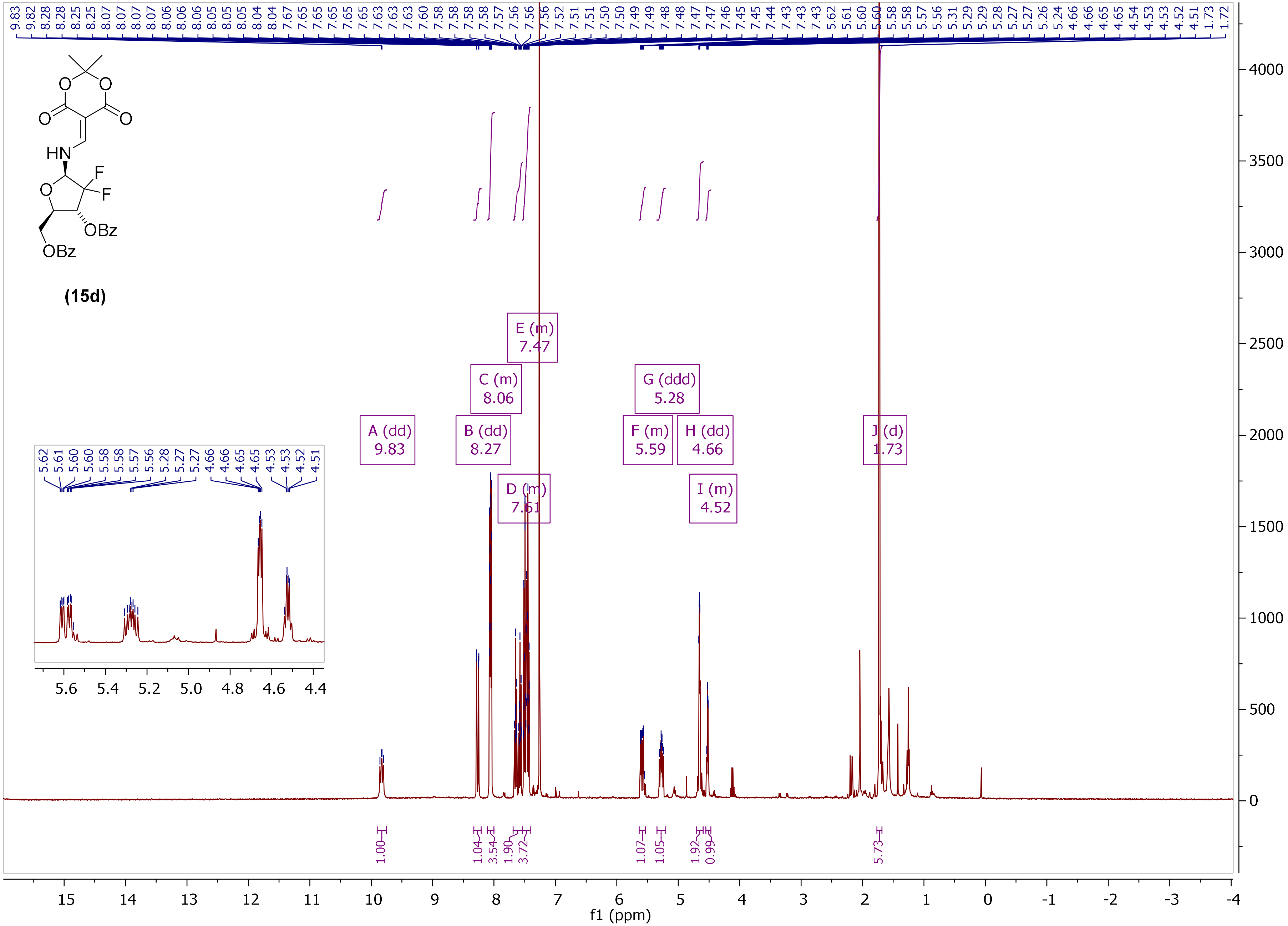

Figure S. 64 - ^{19}F NMR Spectra (377 MHz, $CDCl_3$) - 5-(3,5-Di-*O*-benzoyl-2-deoxy-2,2-difluoro-β-*D*-ribofuranosylamino-methylene)-2,2-dimethyl-1,3-dioxa-4,6-dione -**15d**

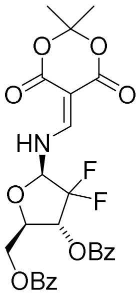

(15d)

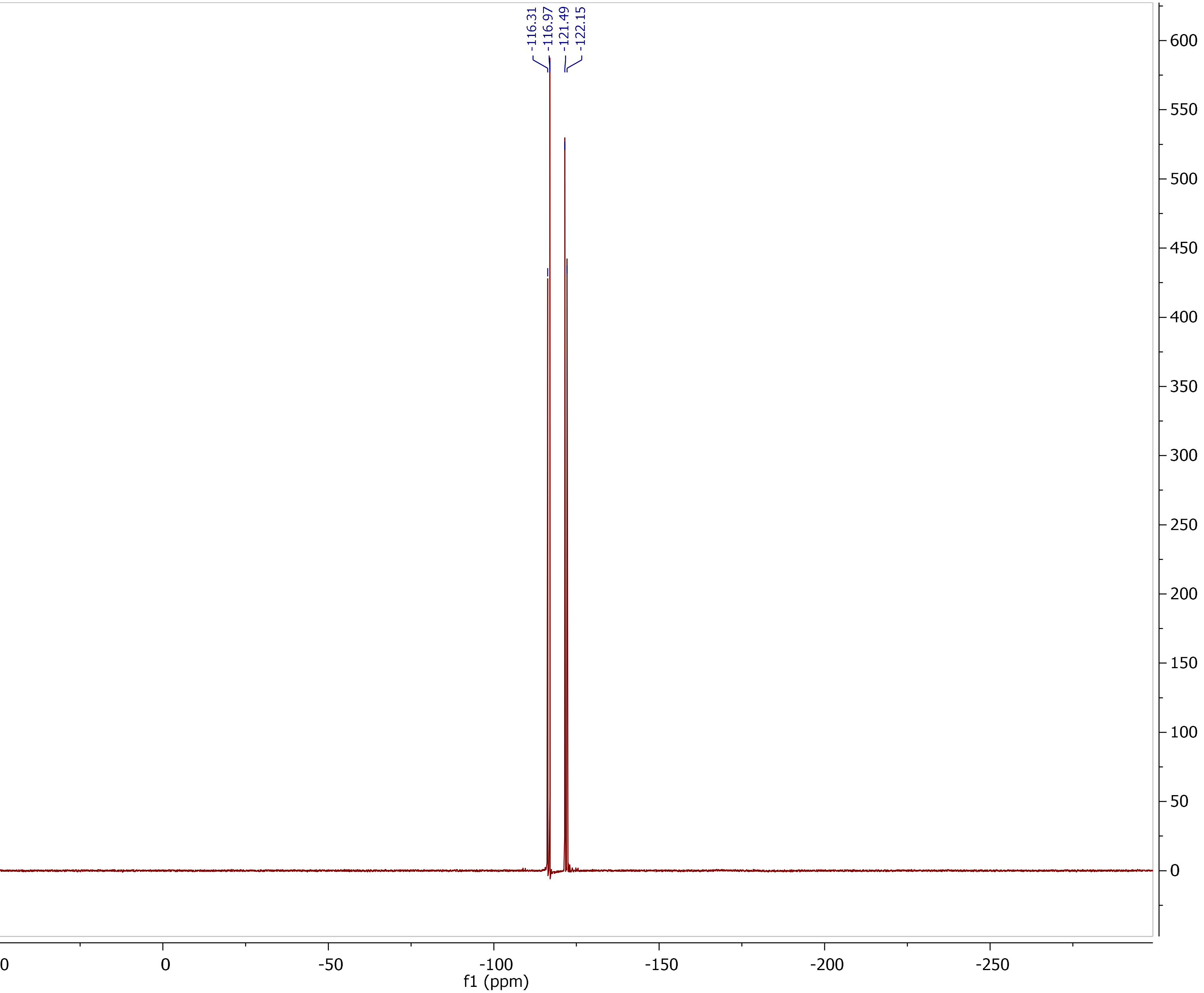

Figure S. 65 - ^{13}C NMR Spectra (101 MHz, $CDCl_3$) - 5-(3,5-Di-*O*-benzoyl-2-deoxy-2,2-difluoro-β-*D*-ribofuranosylamino-methylene)-2,2-dimethyl-1,3-dioxa-4,6-dione - **15d**

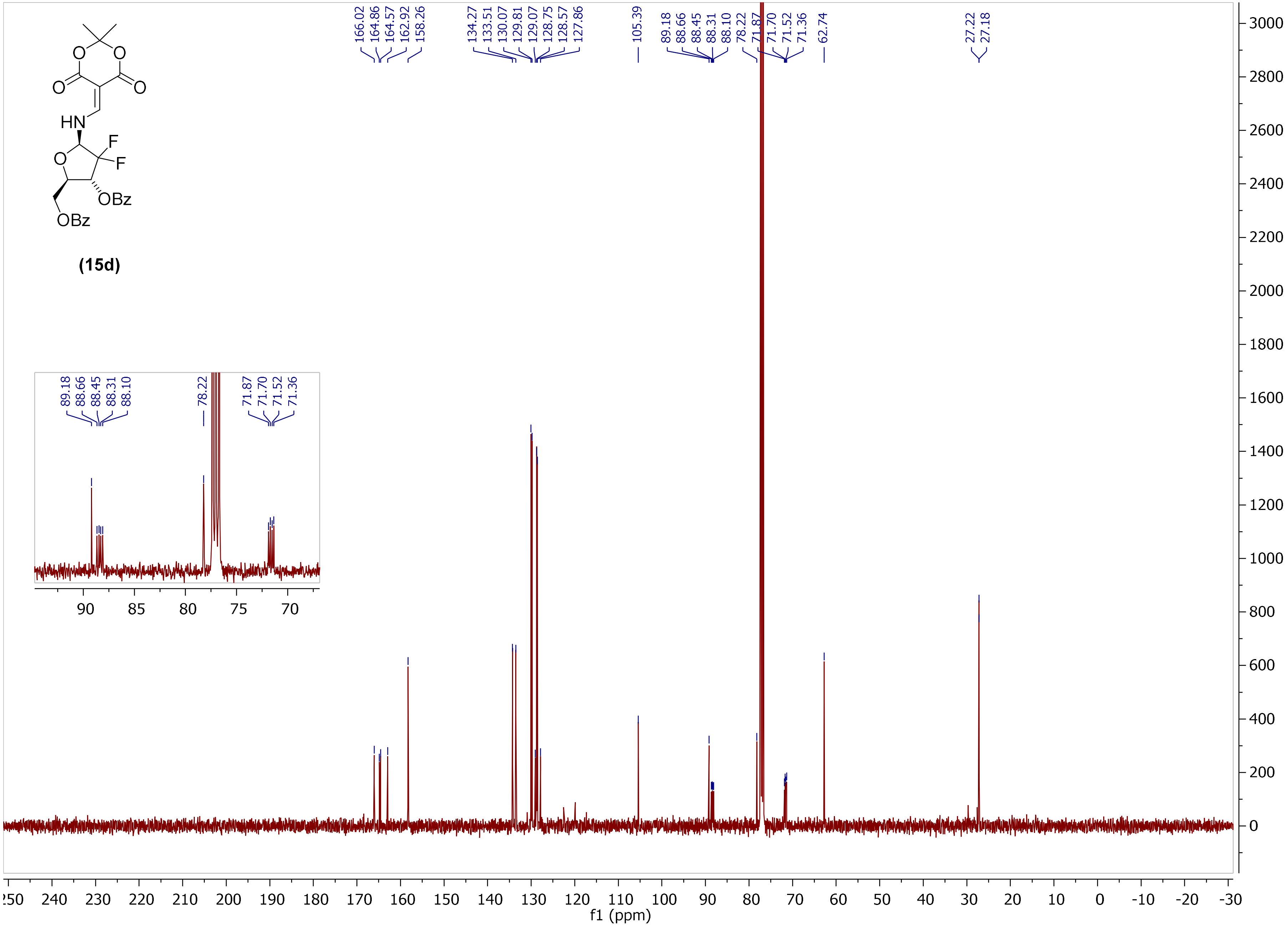

Figure S. 66 - ^{1}H-NMR Spectrum (400 MHz, $CDCl_3$) - 5-(3,5-Di-*O*-benzoyl-2-deoxy-2,2-difluoro-α-*D*-ribofuranosylamino-methylene)-2,2-dimethyl-1,3-dioxa-4,6-dione –(1-epi-**15d)**

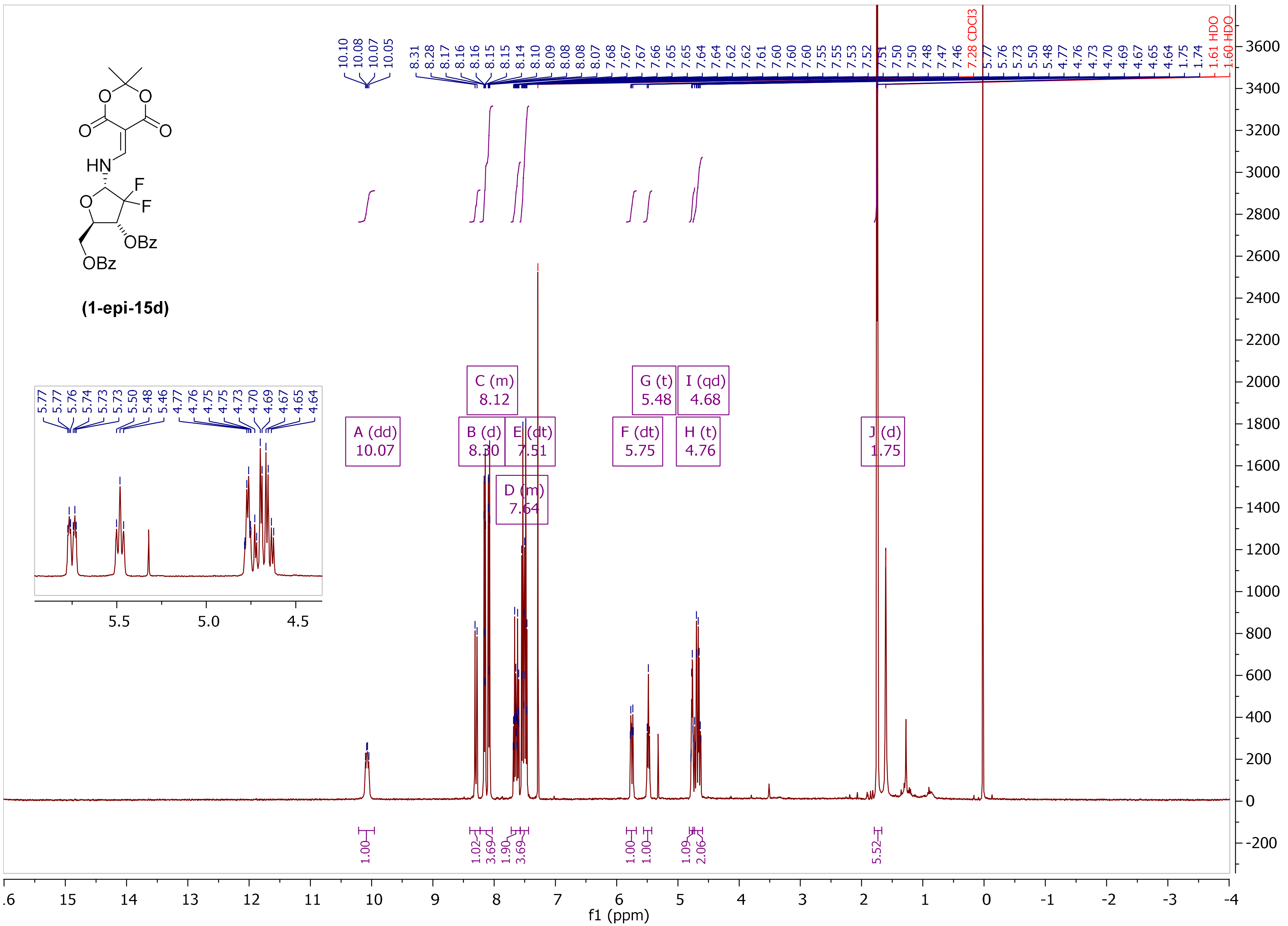

Figure S. 67 - ^{19}F NMR Spectra (377 MHz, $CDCl_3$) - 5-(3,5-Di-*O*-benzoyl-2-deoxy-2,2-difluoro-α-*D*-ribofuranosylamino-methylene)-2,2-dimethyl-1,3-dioxa-4,6-dione – (1-epi-**15d)**

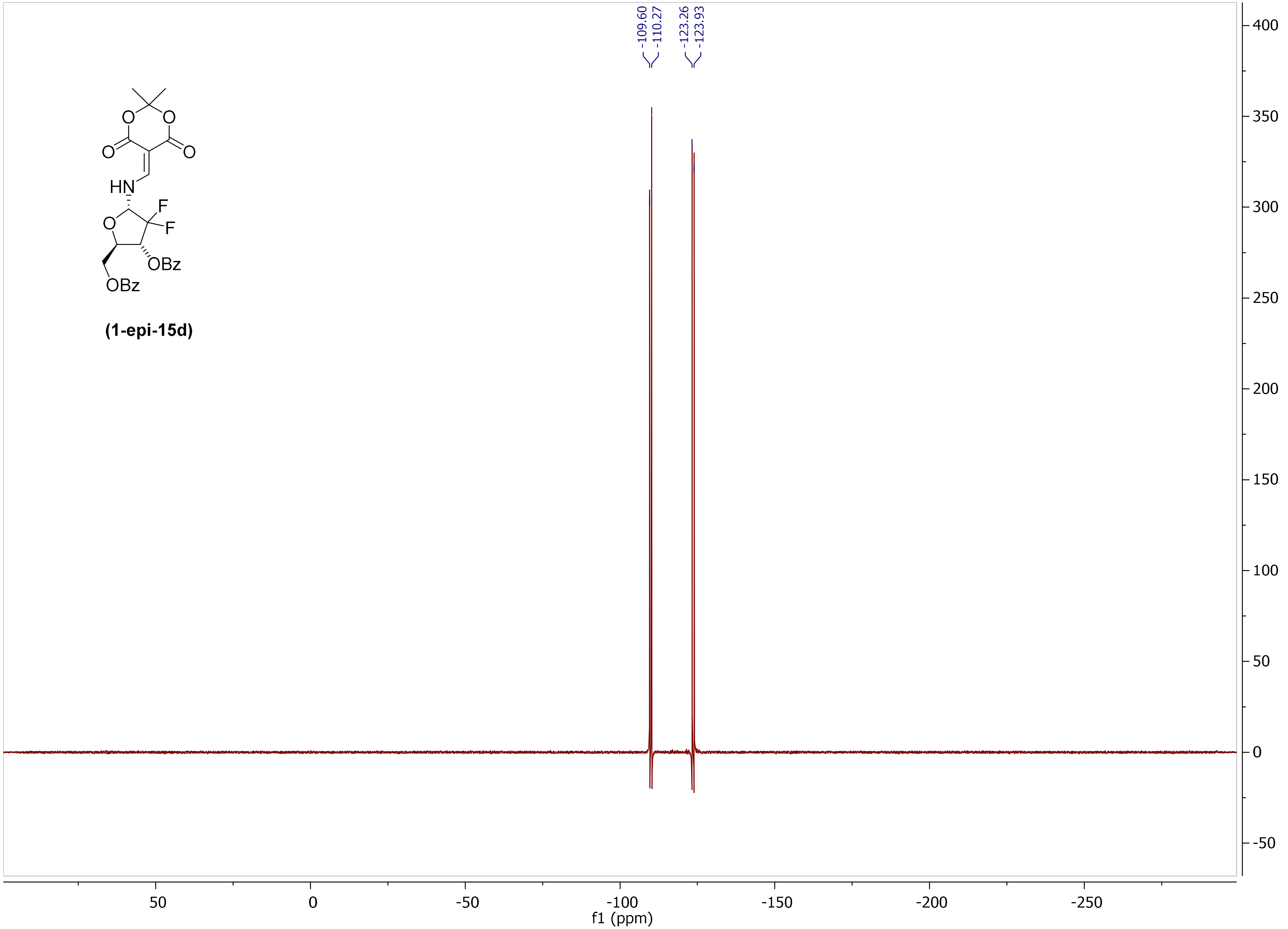

Figure S. 68 - ^{13}C NMR Spectra (101 MHz, $CDCl_3$) - 5-(3,5-Di-*O*-benzoyl-2-deoxy-2,2-difluoro-α-*D*-ribofuranosylamino-methylene)-2,2-dimethyl-1,3-dioxa-4,6-dione – (1-epi-**15d)**

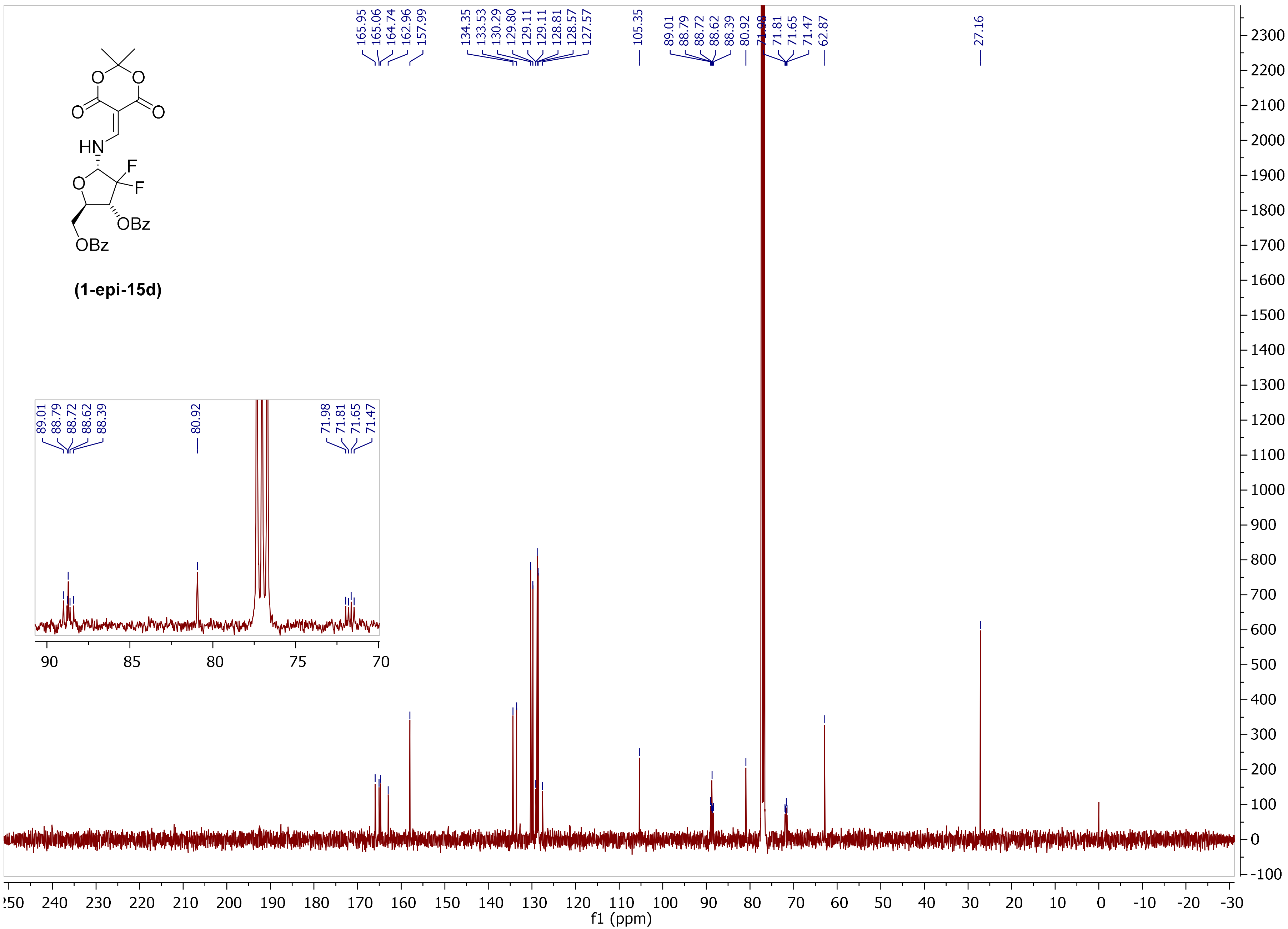

Figure S. 69 - ^{1}H-NMR Spectrum (400 MHz, $CDCl_3$) - 5-(2,3,4,6-tetra-*O*-acetyl-α-*D*-mannopyranosylamino-methylene)-2,2-dimethyl-1,3-dioxa-4,6-dione – **15f**

(15f)

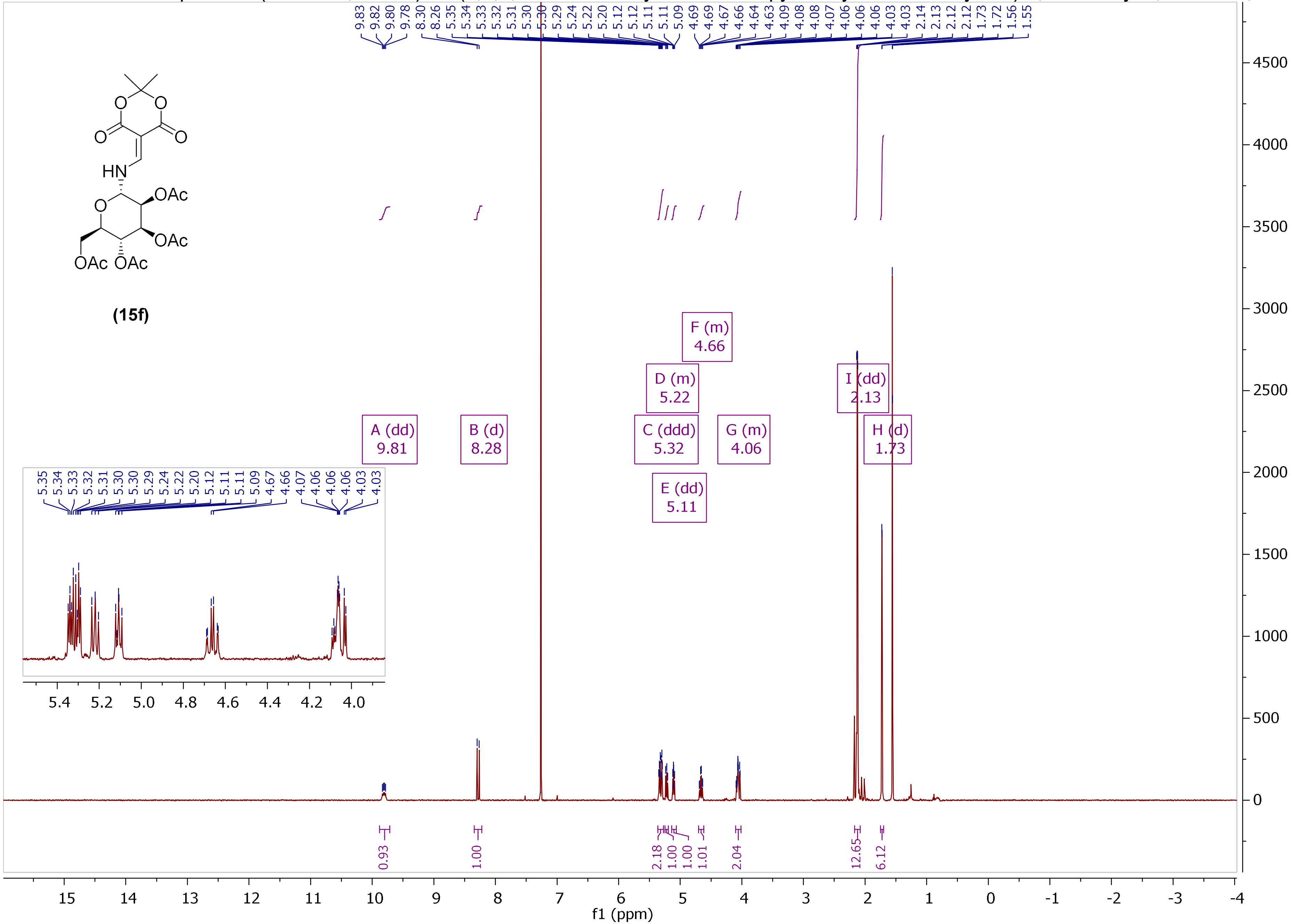

Figure S. 70 - ^{13}C NMR Spectra (101 MHz, $CDCl_3$) - 5-(2,3,4,6-tetra-*O*-acetyl-α-*D*-mannopyranosylamino-methylene)-2,2-dimethyl-1,3-dioxa-4,6-dione – **15f**

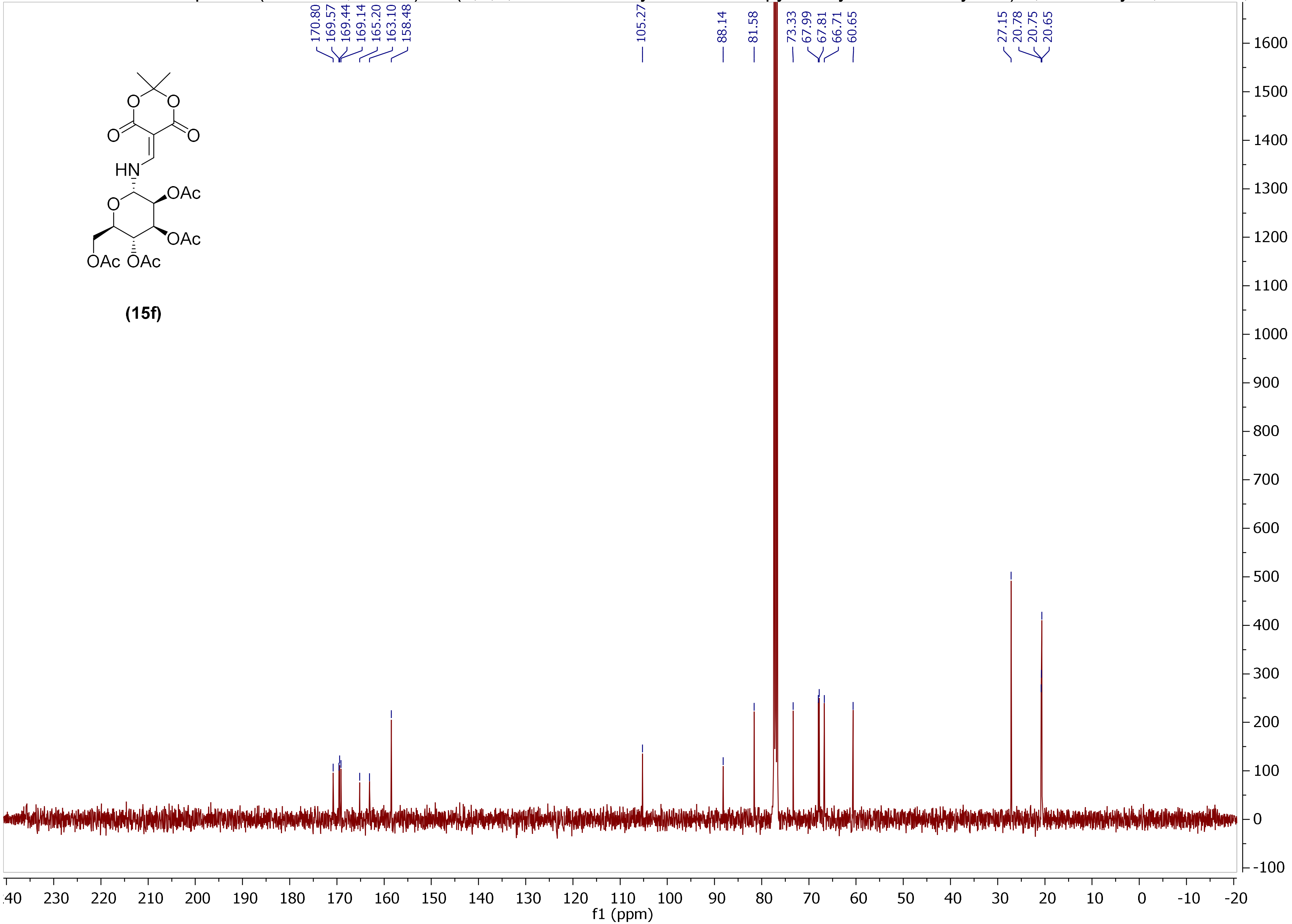

(15f)

Figure S. 71 - ^{1}H-NMR Spectrum (400 MHz, $CDCl_3$) - (5-(ethoxymethylene)-2,2-dimethyl-1,3-dioxane-4,6-dione – **46**

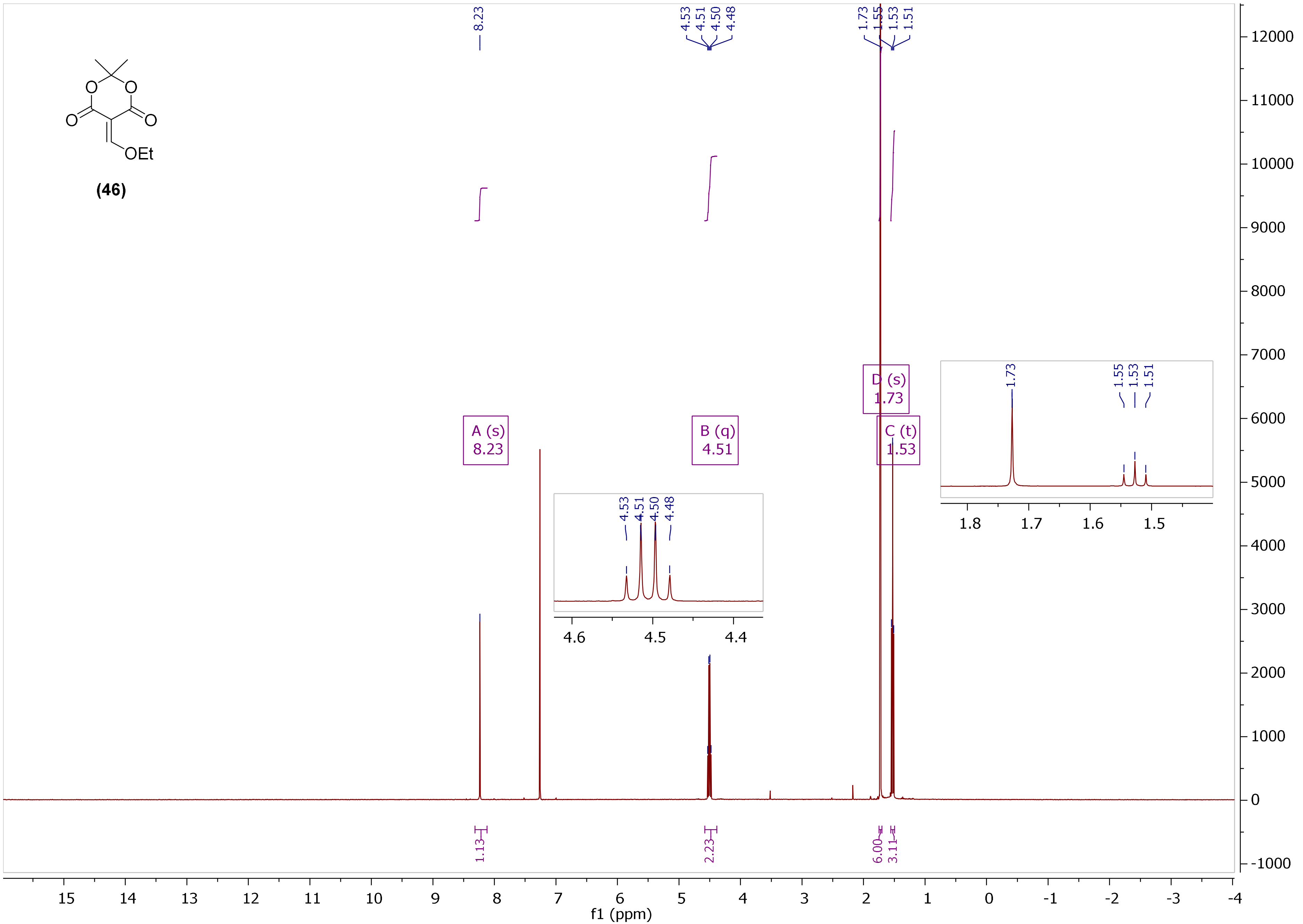

Figure S. 72 - ^{13}C NMR Spectra (101 MHz, $CDCl_3$) - (5-(ethoxymethylene)-2,2-dimethyl-1,3-dioxane-4,6-dione – **46**

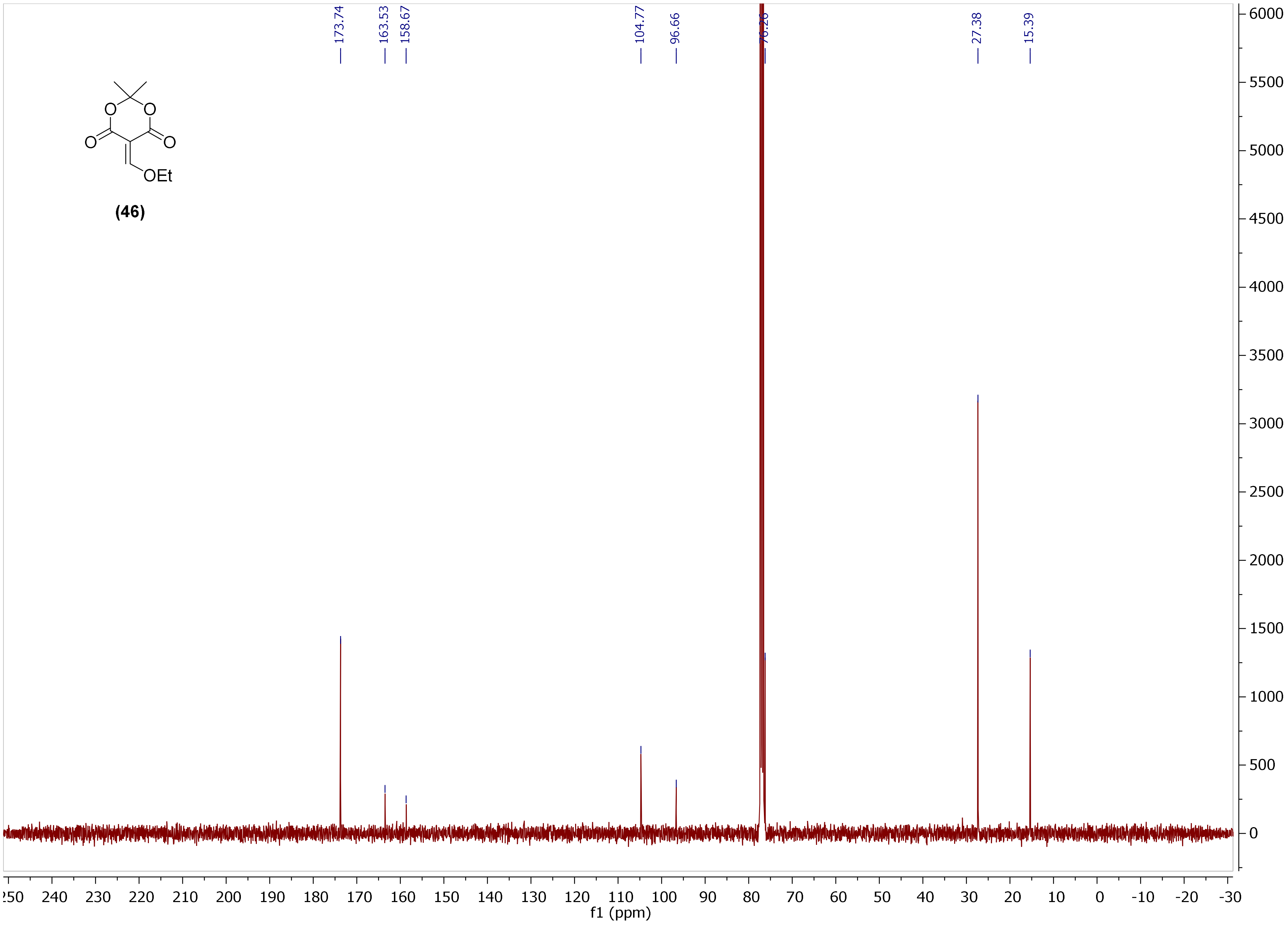

Figure S. 73 - ^{1}H-NMR Spectrum (400 MHz, $CDCl_3$) - 5-(aminomethylene)-2,2-dimethyl-1,3-dioxane-4,6-dione – **14**

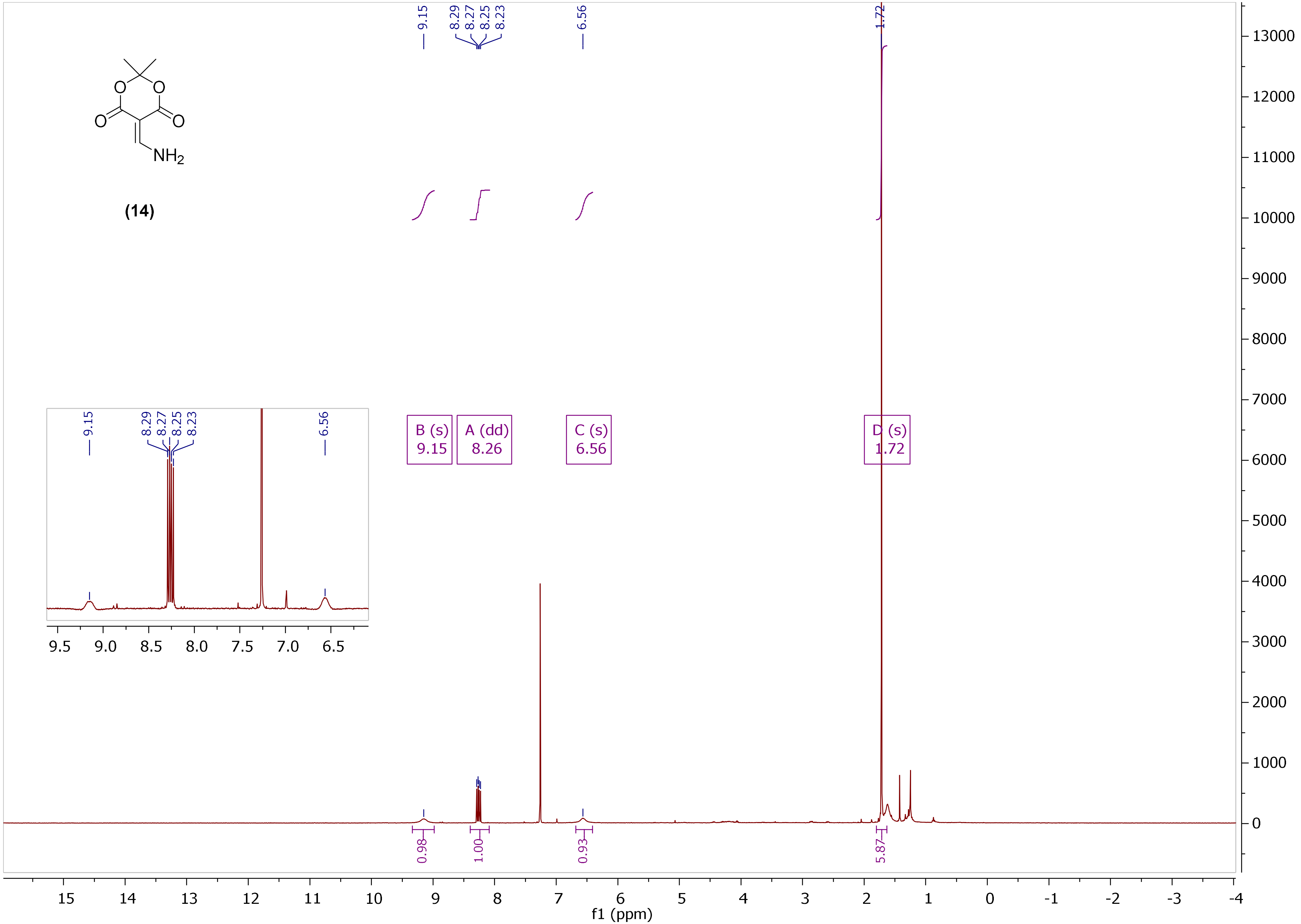

Figure S. 74 - ^{13}C NMR Spectra (101 MHz, $CDCl_3$) - 5-(aminomethylene)-2,2-dimethyl-1,3-dioxane-4,6-dione – **14**

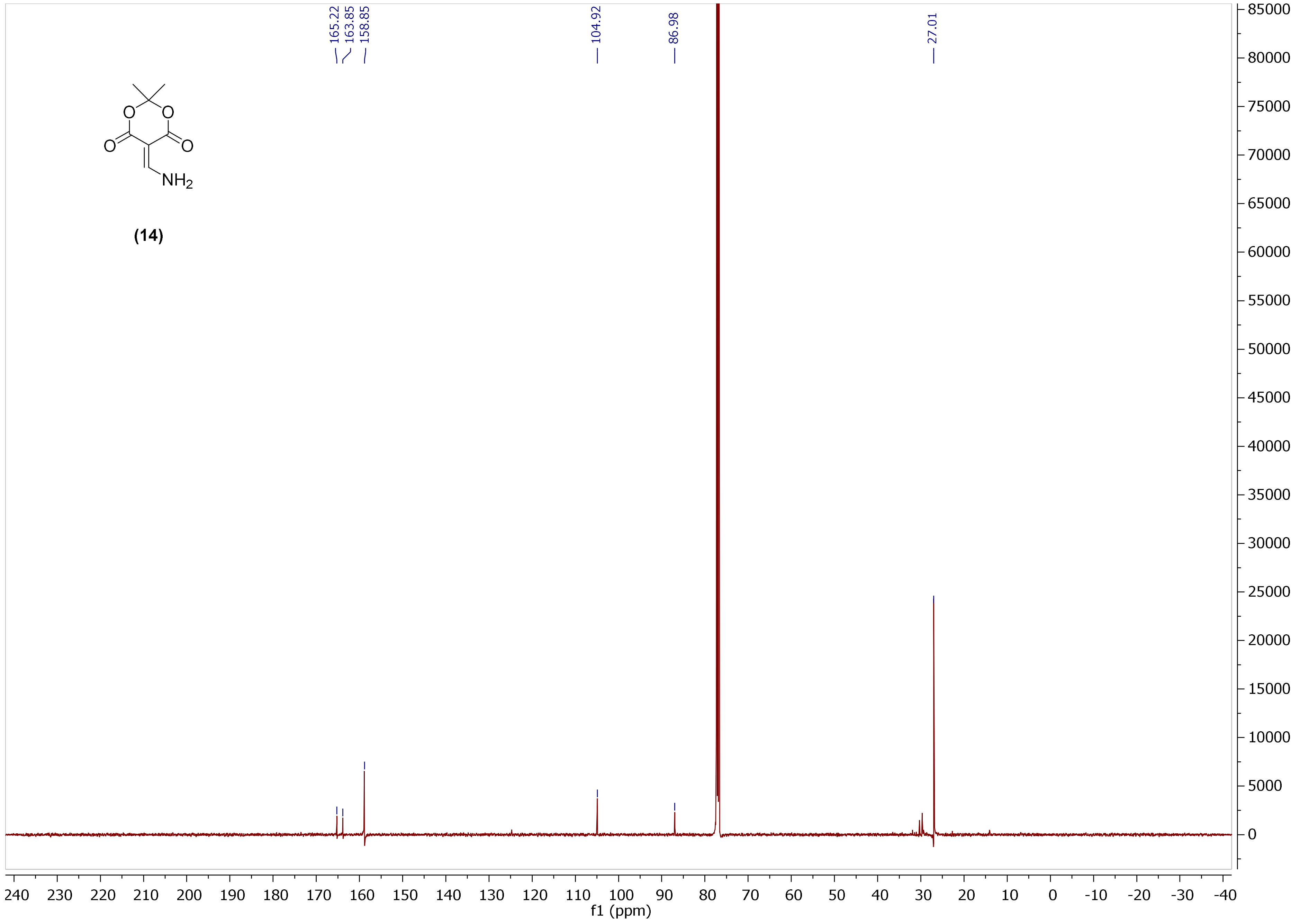

Figure S. 75 - ^{1}H-NMR Spectrum (400 MHz, $CDCl_3$) - 5-(2,3,5-tri-*O*-acetyl-β-*D*-ribofuranosylamino-methylene)-2,2-dimethyl-1,3-dioxa-4,6-dione – **15h**

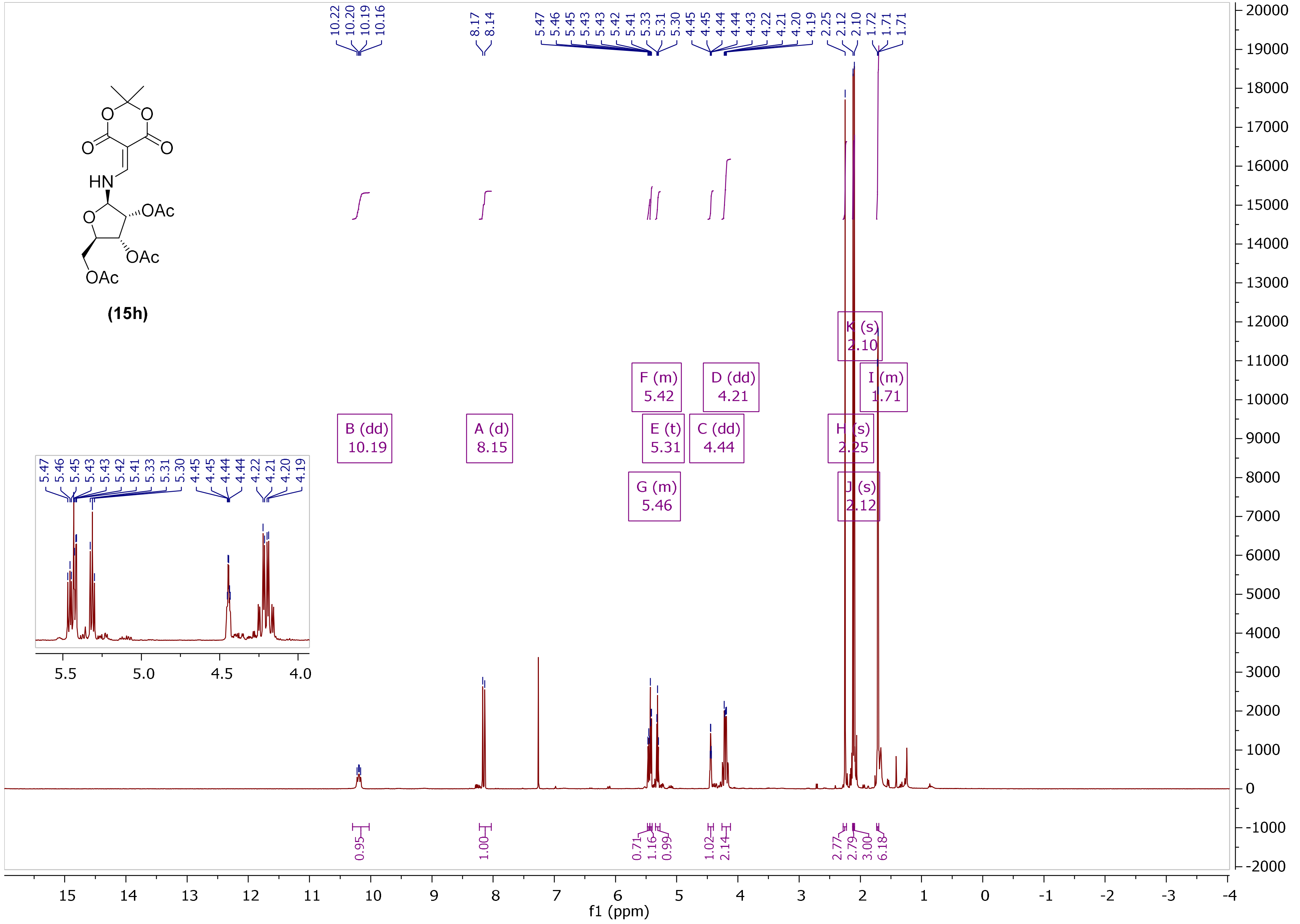

Figure S. 76 - ^{13}C NMR Spectra (101 MHz, $CDCl_3$) - 5-(2,3,5-tri-*O*-acetyl-β-*D*-ribofuranosylamino-methylene)-2,2-dimethyl-1,3-dioxa-4,6-dione – **15h**

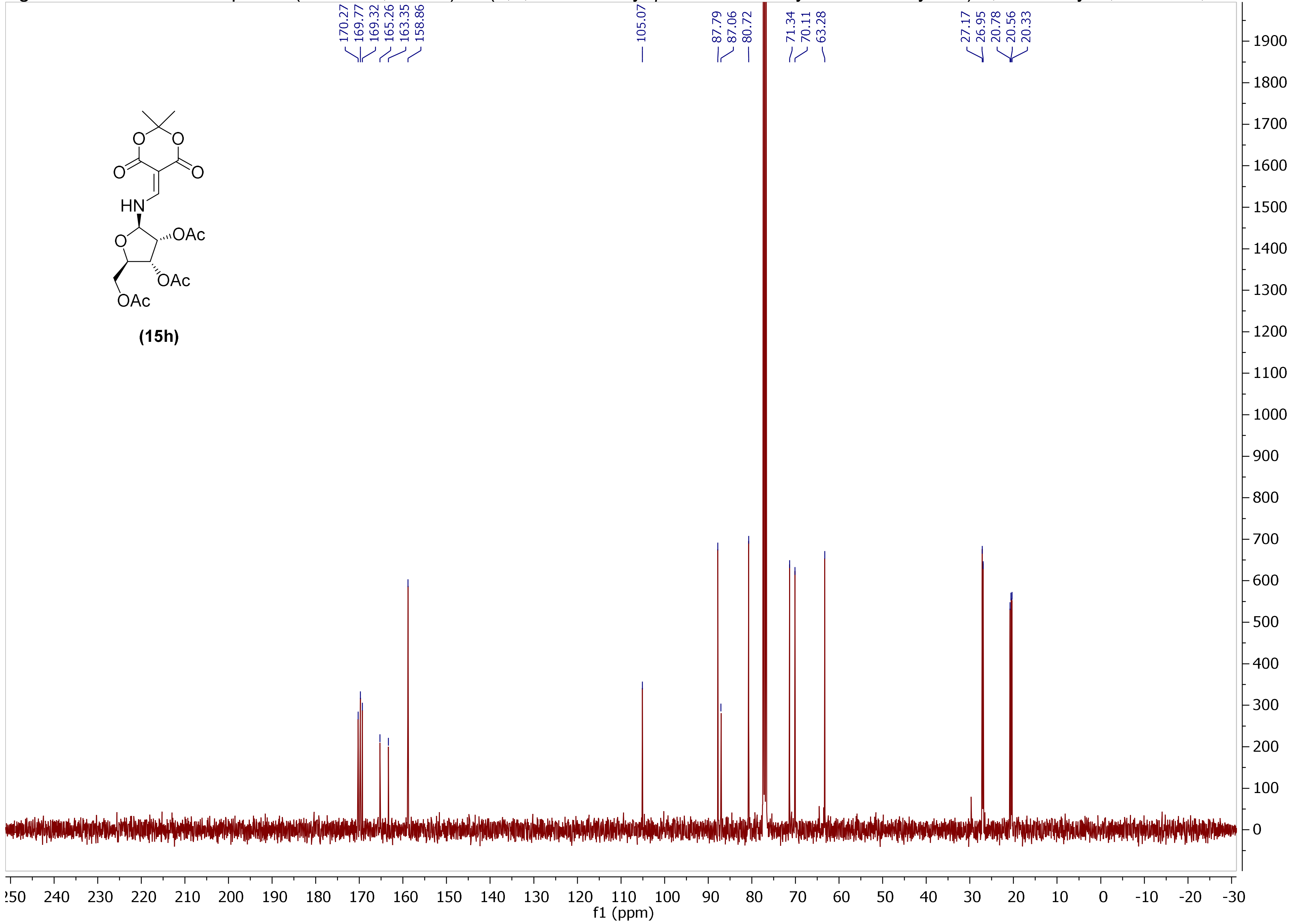

Figure S. 77 - ^{1}H-NMR Spectrum (400 MHz, $CDCl_3$) - 5-(3,5-Di-*O*-benzoyl-2-deoxy-2-fluoro-β-*D*-arabinofuranosylamino-methylene)-2,2-dimethyl-1,3-dioxa-4,6-dione - **15e**

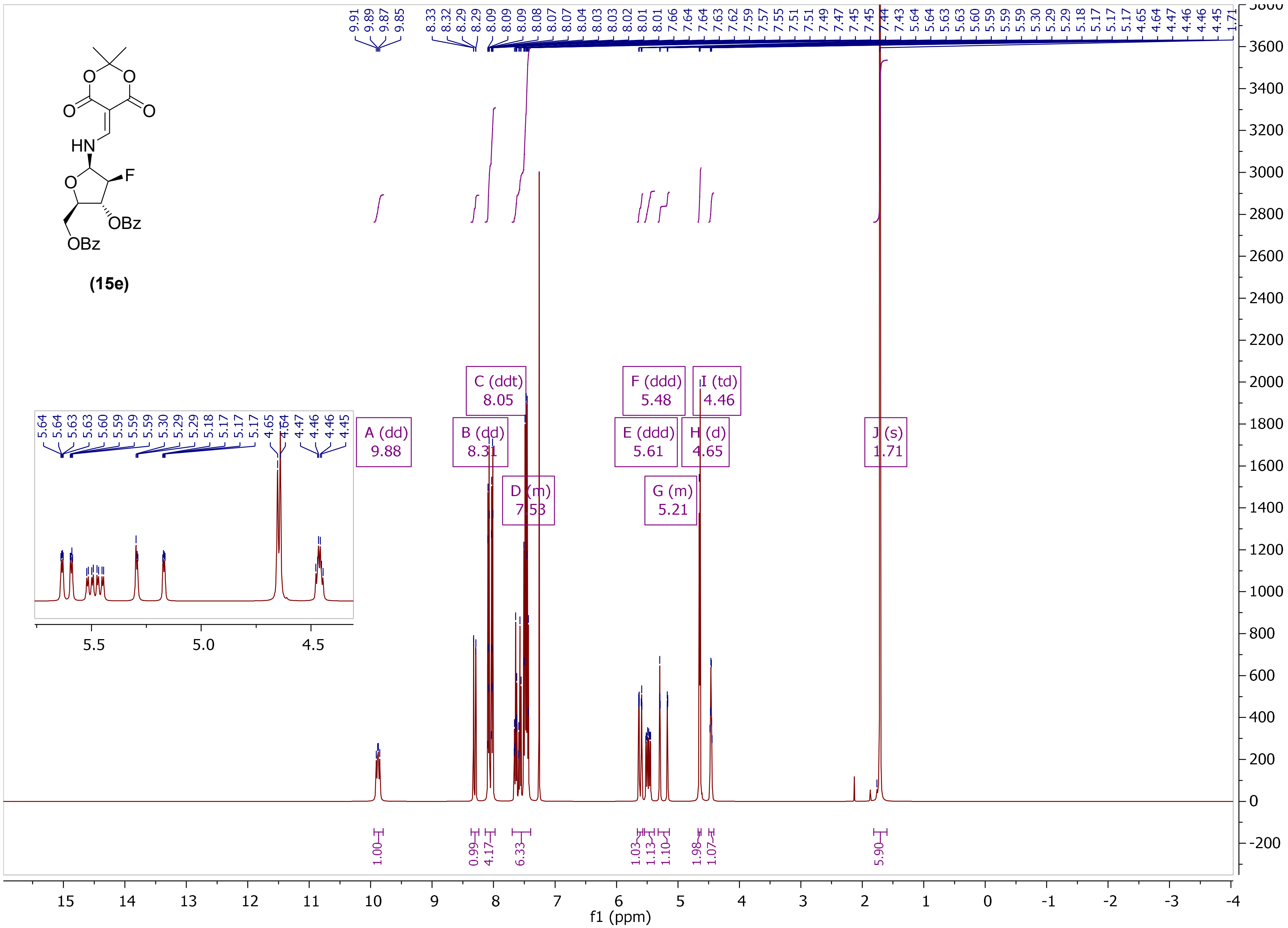

Figure S. 78 - ^{19}F NMR Spectra (377 MHz, $CDCl_3$) - 5-(3,5-Di-*O*-benzoyl-2-deoxy-2-fluoro-β-*D*-arabinofuranosylamino-methylene)-2,2-dimethyl-1,3-dioxa-4,6-dione - **15e**

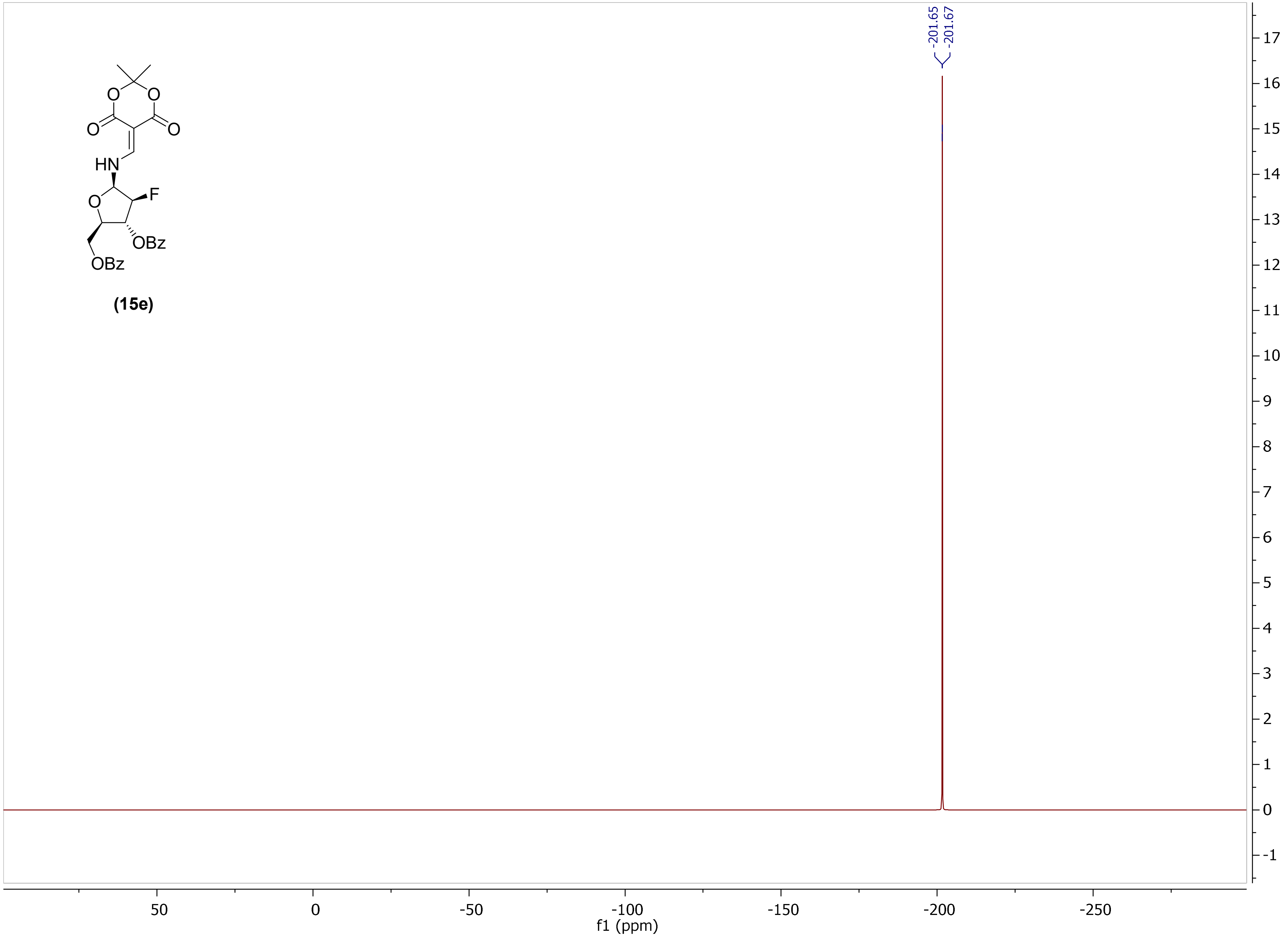

Figure S. 79 - ^{13}C NMR Spectra (101 MHz, $CDCl_3$) - 5-(3,5-Di-*O*-benzoyl-2-deoxy-2-fluoro-β-*D*-arabinofuranosylamino-methylene)-2,2-dimethyl-1,3-dioxa-4,6-dione - **15e**

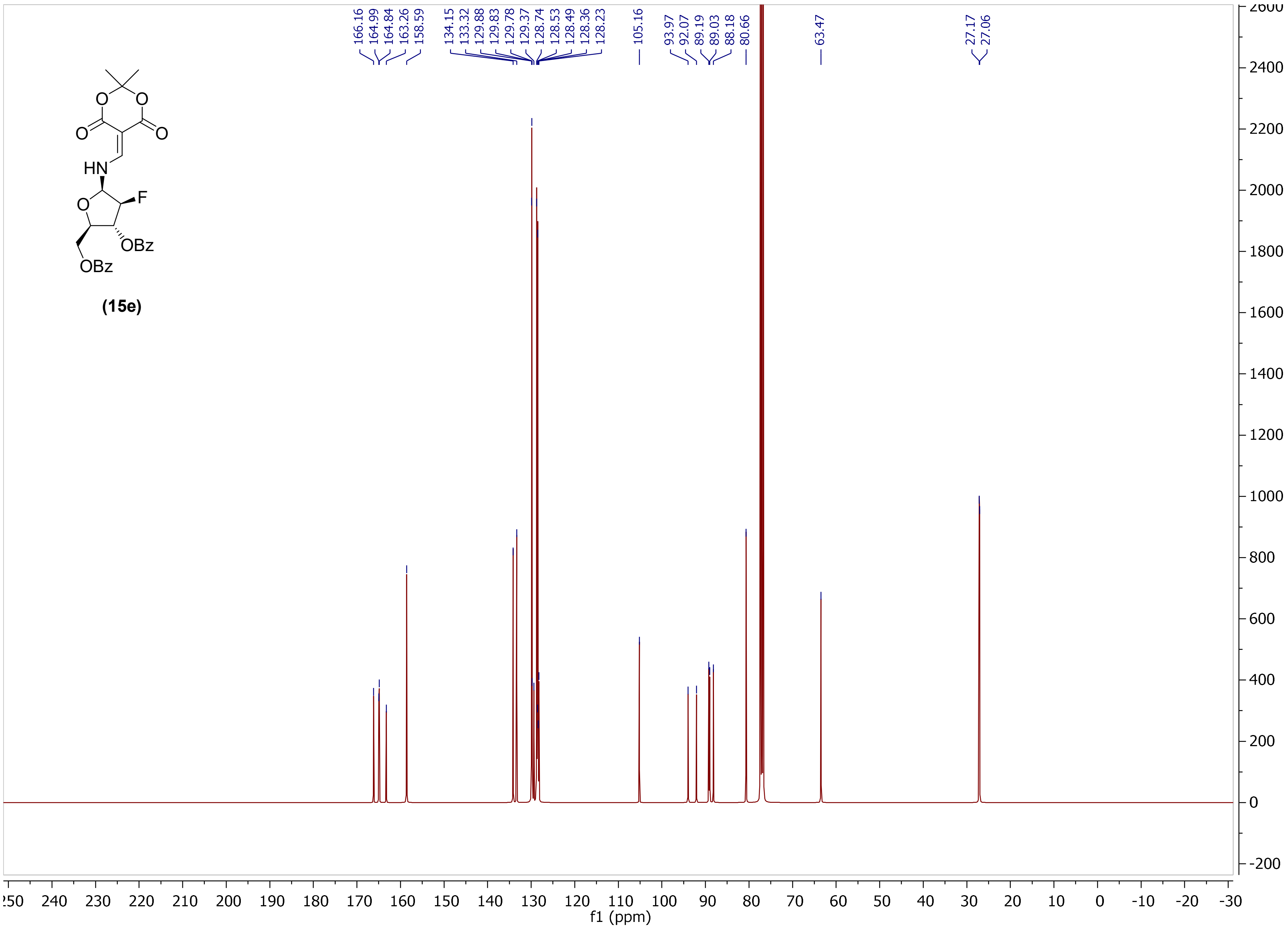

Figure S. 80 - ^{1}H-NMR Spectrum (400 MHz, $CDCl_3$) - 5-(3,5-di-*O*-(4-methylbenzoyl)-2-deoxy-β-*D*-ribofuranosylamino-methylene)-2,2-dimethyl-1,3-dioxa-4,6-dione – **15g** – major anomer

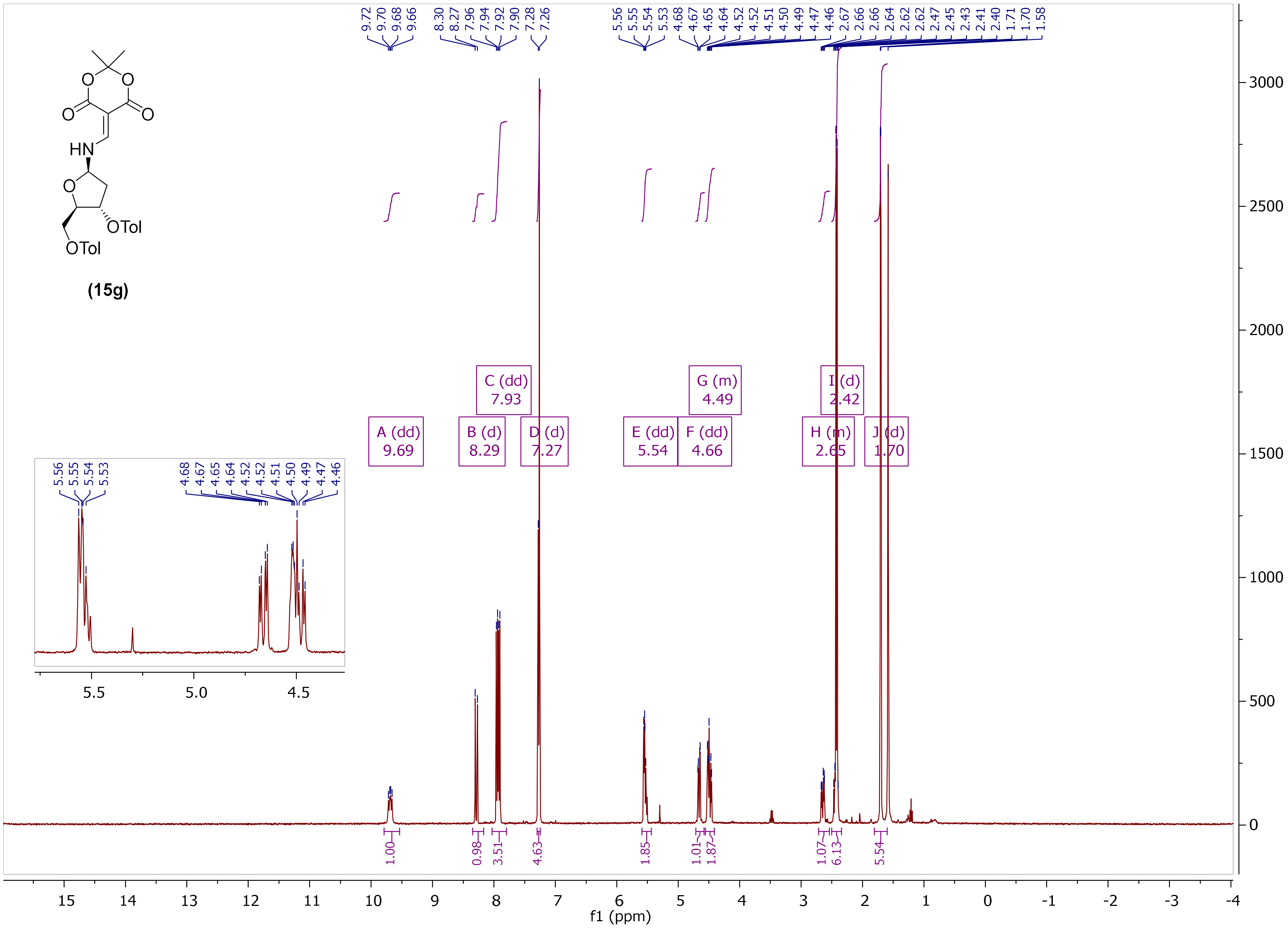

Figure S. 81 - ^{13}C NMR Spectra (101 MHz, $CDCl_3$) - 5-(3,5-di-*O*-(4-methylbenzoyl)-2-deoxy-β-*D*-ribofuranosylamino-methylene)-2,2-dimethyl-1,3-dioxa-4,6-dione – **15g** – major anomer

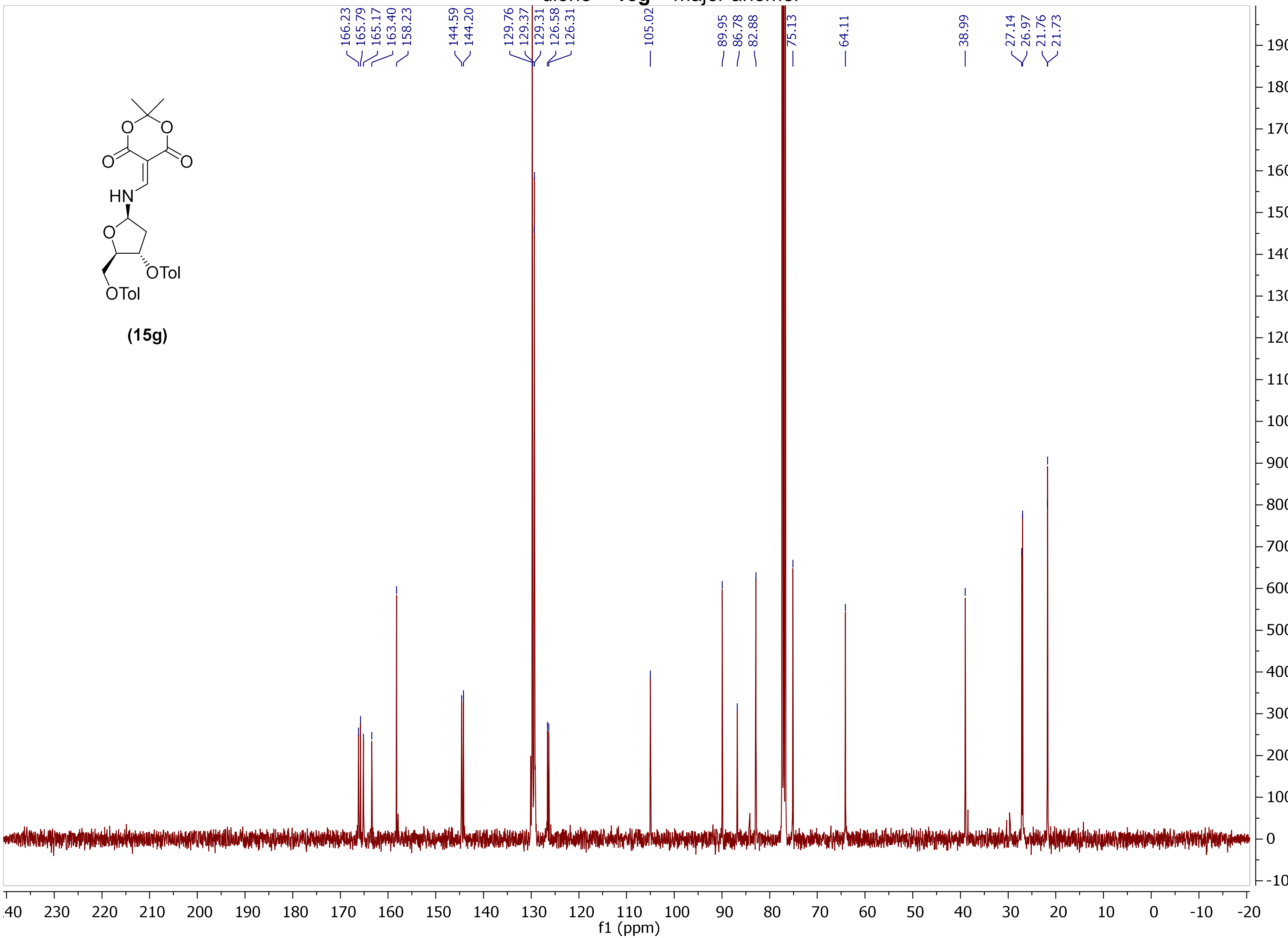

Figure S. 82 - ^{1}H-NMR Spectrum (400 MHz, $CDCl_3$) - 5-(3,5-di-*O*-(4-methylbenzoyl)-2-deoxy-α-*D*-ribofuranosylamino-methylene)-2,2-dimethyl-1,3-dioxa-4,6-dione – (1-epi-**15g**) – minor anomer

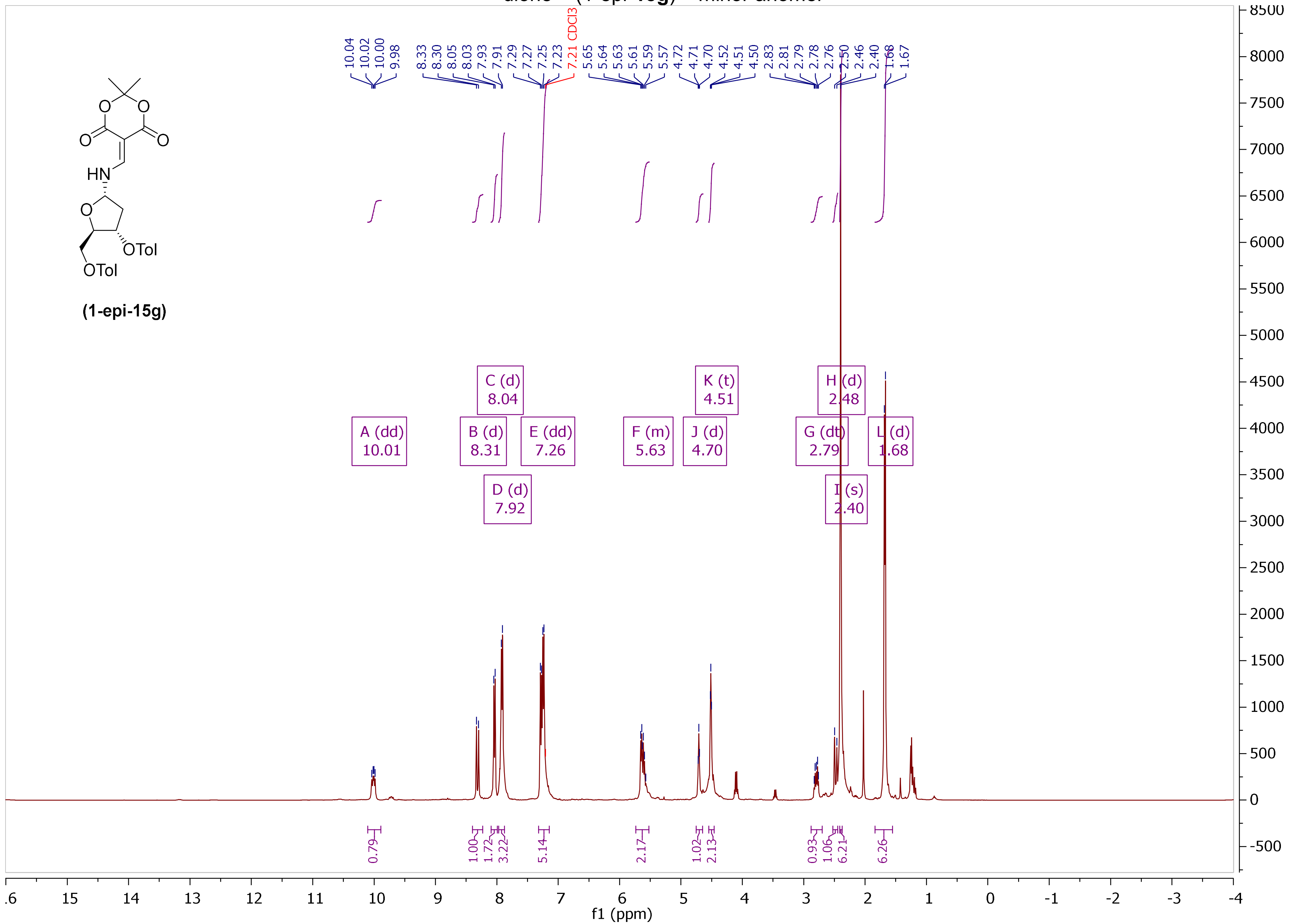

Figure S. 83 - ^{13}C NMR Spectra (101 MHz, $CDCl_3$) - 5-(3,5-di-*O*-(4-methylbenzoyl)-2-deoxy-α-*D*-ribofuranosylamino-methylene)-2,2-dimethyl-1,3-dioxa-4,6-dione – (1-epi-**15g**) – minor anomer

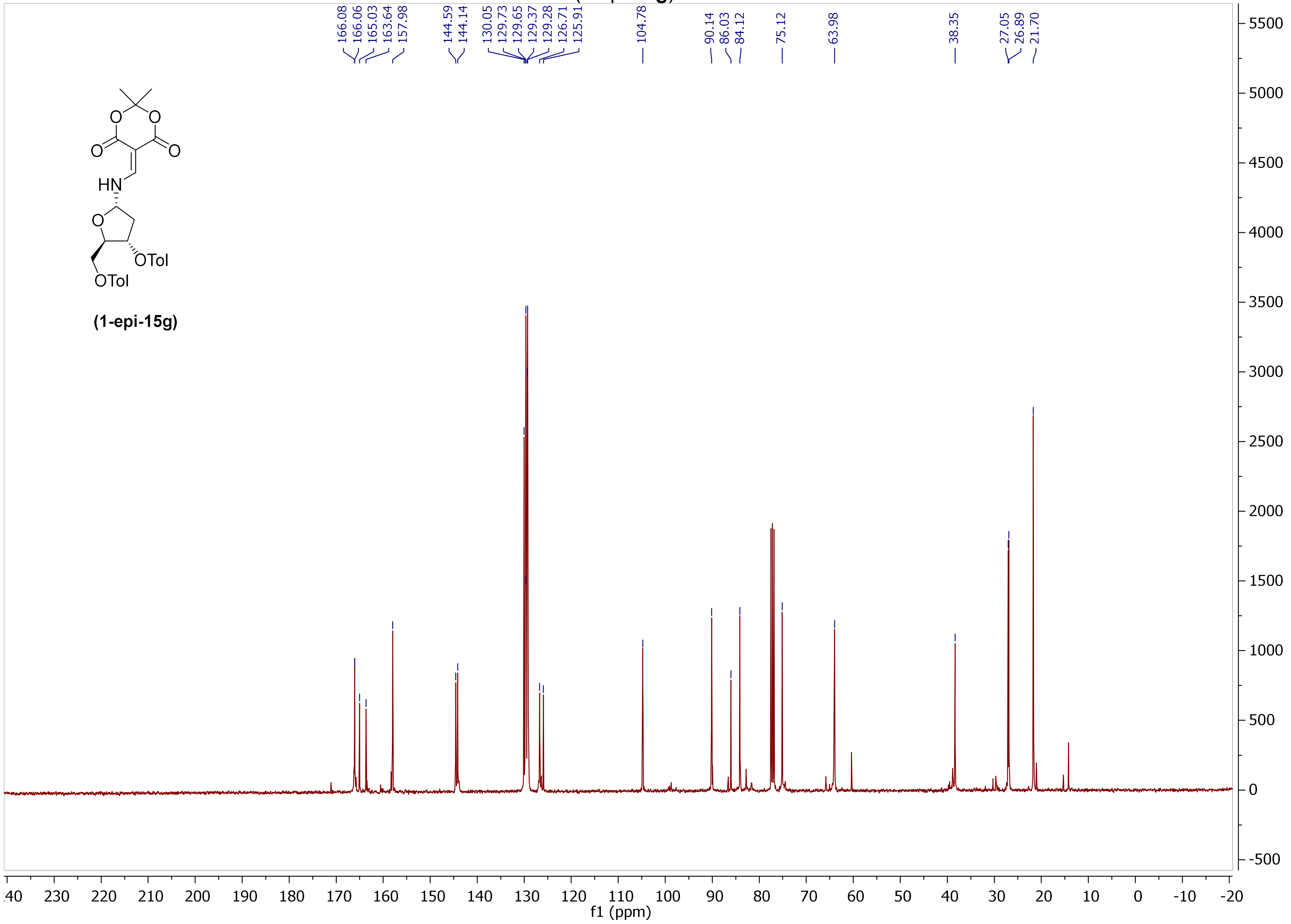

Figure S. 84 - ^{1}H-NMR Spectrum (400 MHz, $CDCl_3$) - 3-Benzyl-1-cyclohexyl-2,4-dioxo-1,2,3,4-tetrahydropyrimidine-5-carboxylic Acid – **16a**

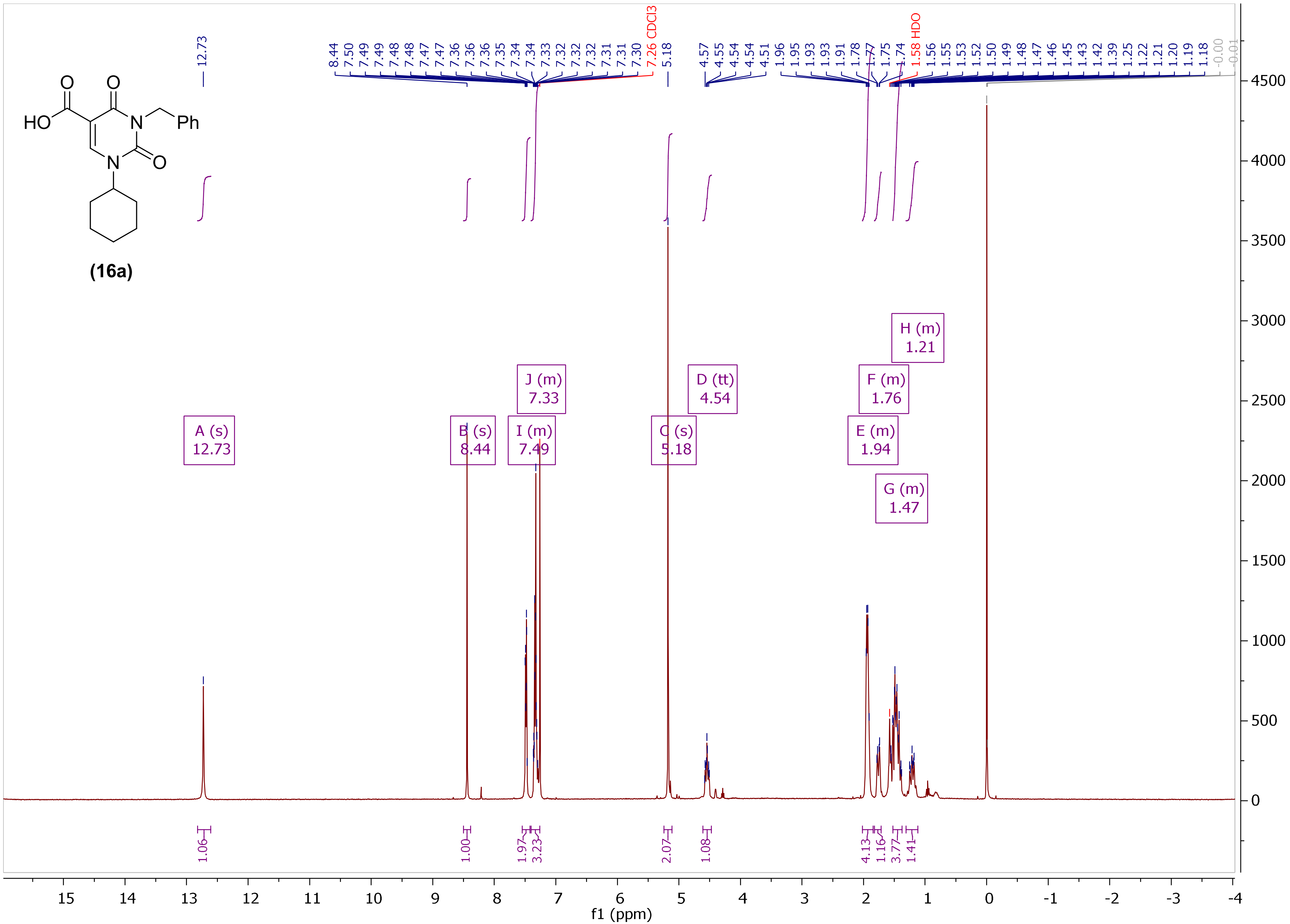

Figure S. 85 - ^{13}C NMR Spectra (101 MHz, $CDCl_3$) - 3-Benzyl-1-cyclohexyl-2,4-dioxo-1,2,3,4-tetrahydropyrimidine-5-carboxylic Acid – **16a**

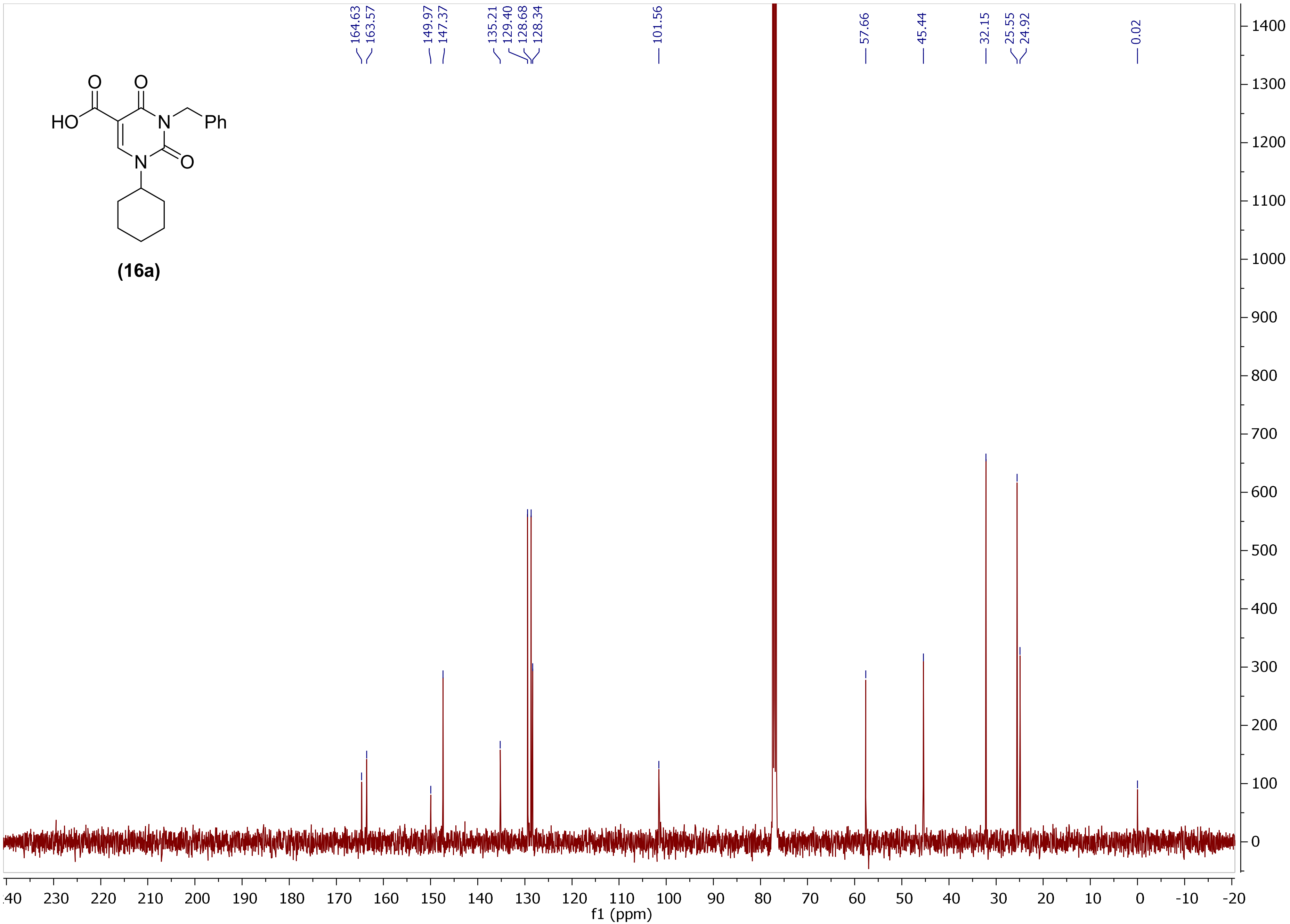

(16a)

Figure S. 86 - ^{1}H-NMR Spectrum (400 MHz, $CDCl_3$) - 3-Benzyl-5-bromo-1-cyclohexylpyrimidine-2,4(1H,3H)-dione – **17a**

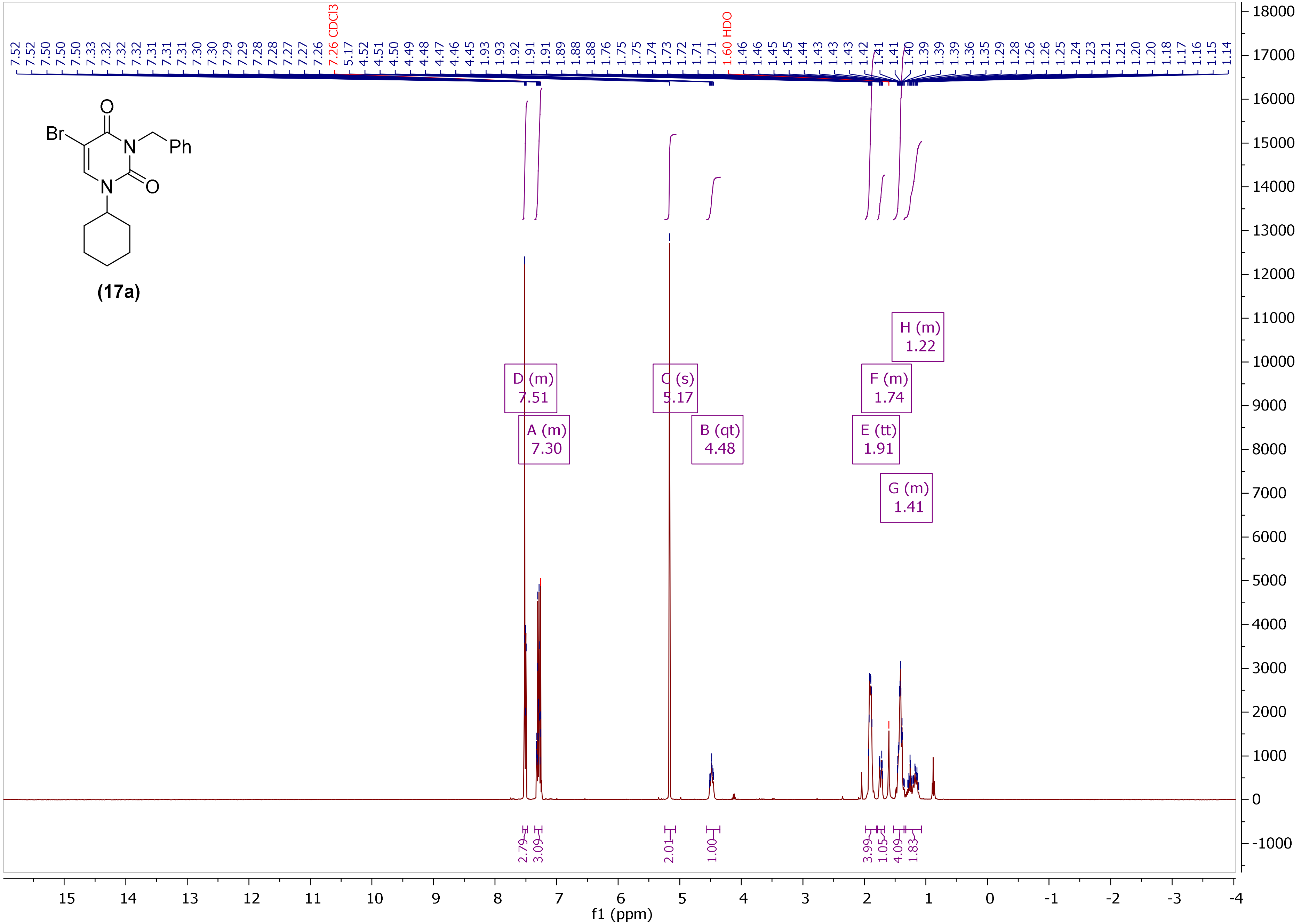

Figure S. 87 - ^{13}C NMR Spectra (101 MHz, $CDCl_3$) - 3-Benzyl-5-bromo-1-cyclohexylpyrimidine-2,4(1H,3H)-dione – **17a**

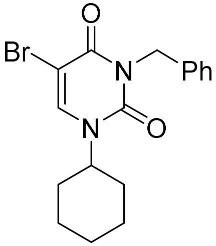

(17a)

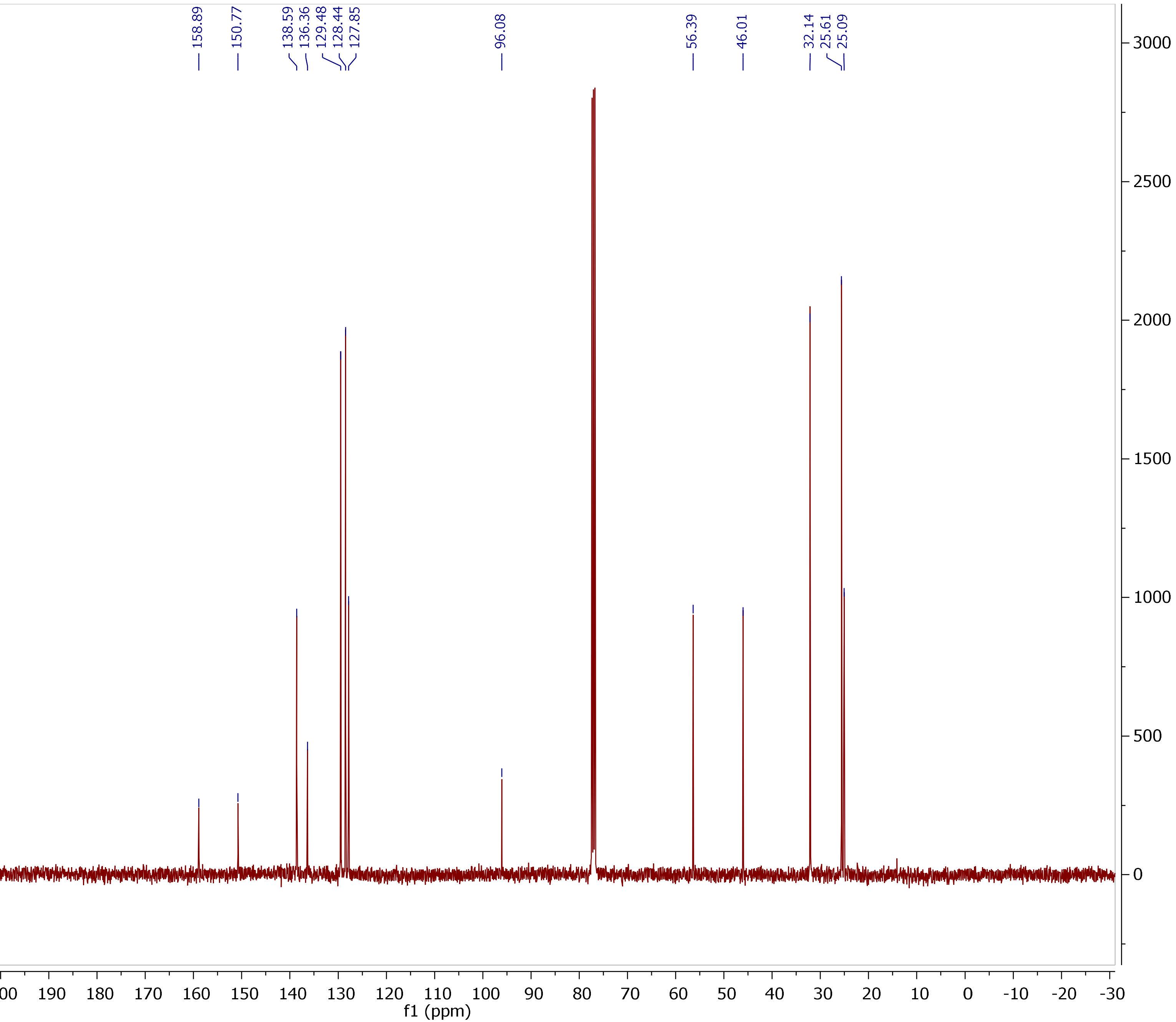

Figure S. 88 - ^{1}H-NMR Spectrum (400 MHz, $CDCl_3$) - 3-benzyl-5-chloro-1-cyclohexylpyrimidine-2,4(1H,3H)-dione – **49**

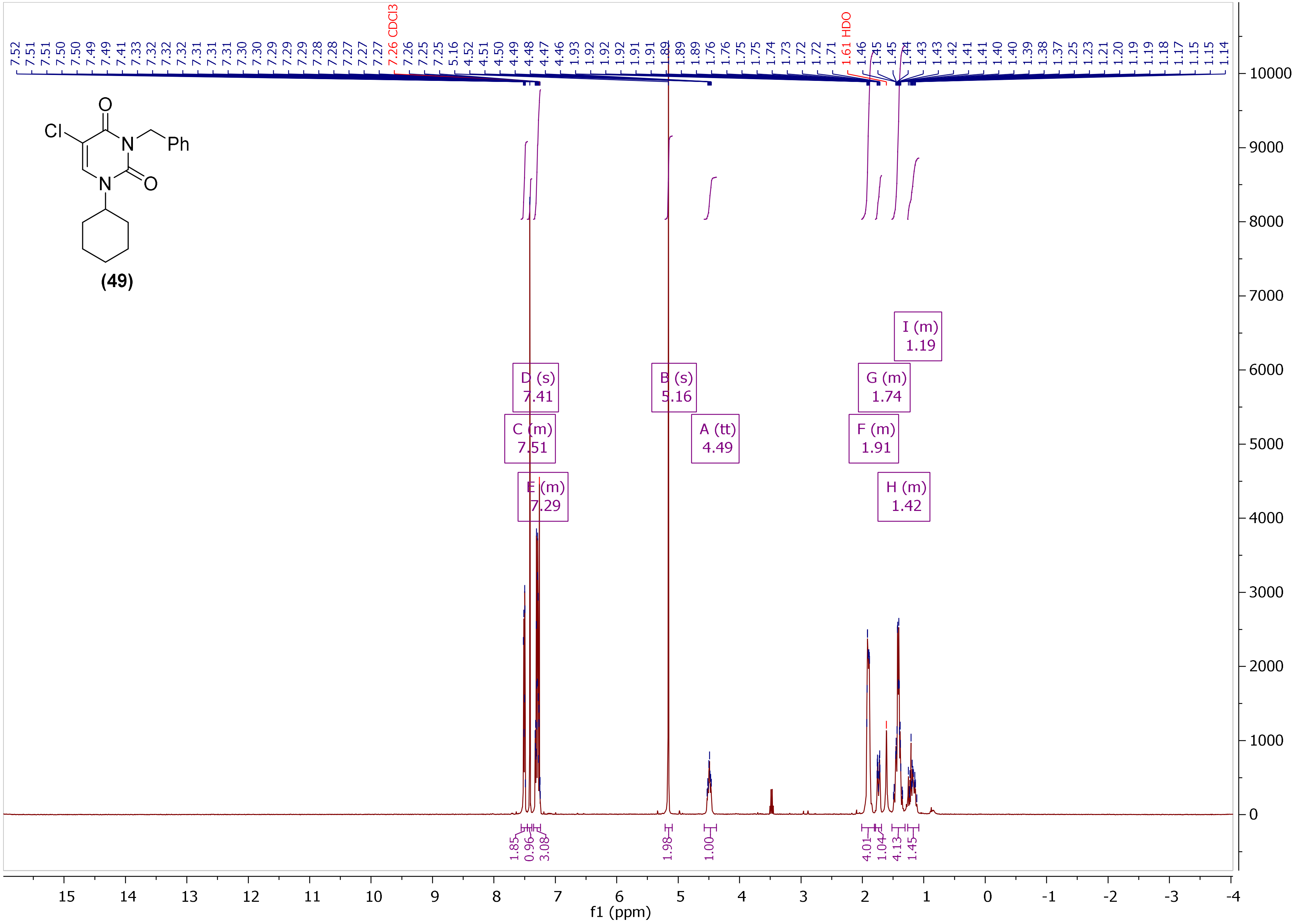

Figure S. 89 - ^{13}C NMR Spectra (101 MHz, $CDCl_3$) - 3-benzyl-5-chloro-1-cyclohexylpyrimidine-2,4(1H,3H)-dione – **49**

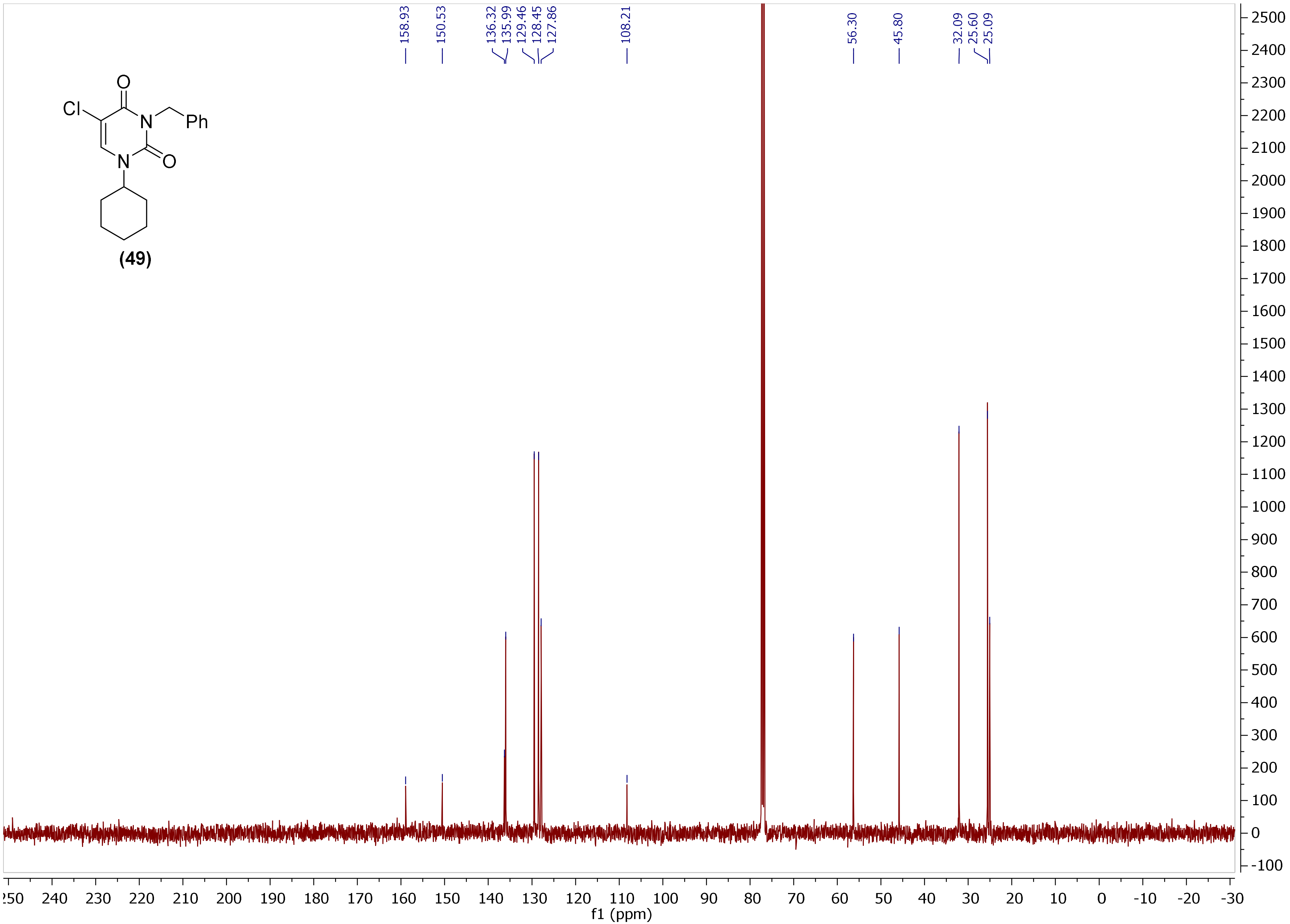

Figure S. 90 - ^{1}H-NMR Spectrum (400 MHz, $CDCl_3$) - 3-benzyl-5-iodo-1-cyclohexylpyrimidine-2,4(1H,3H)-dione - **50**

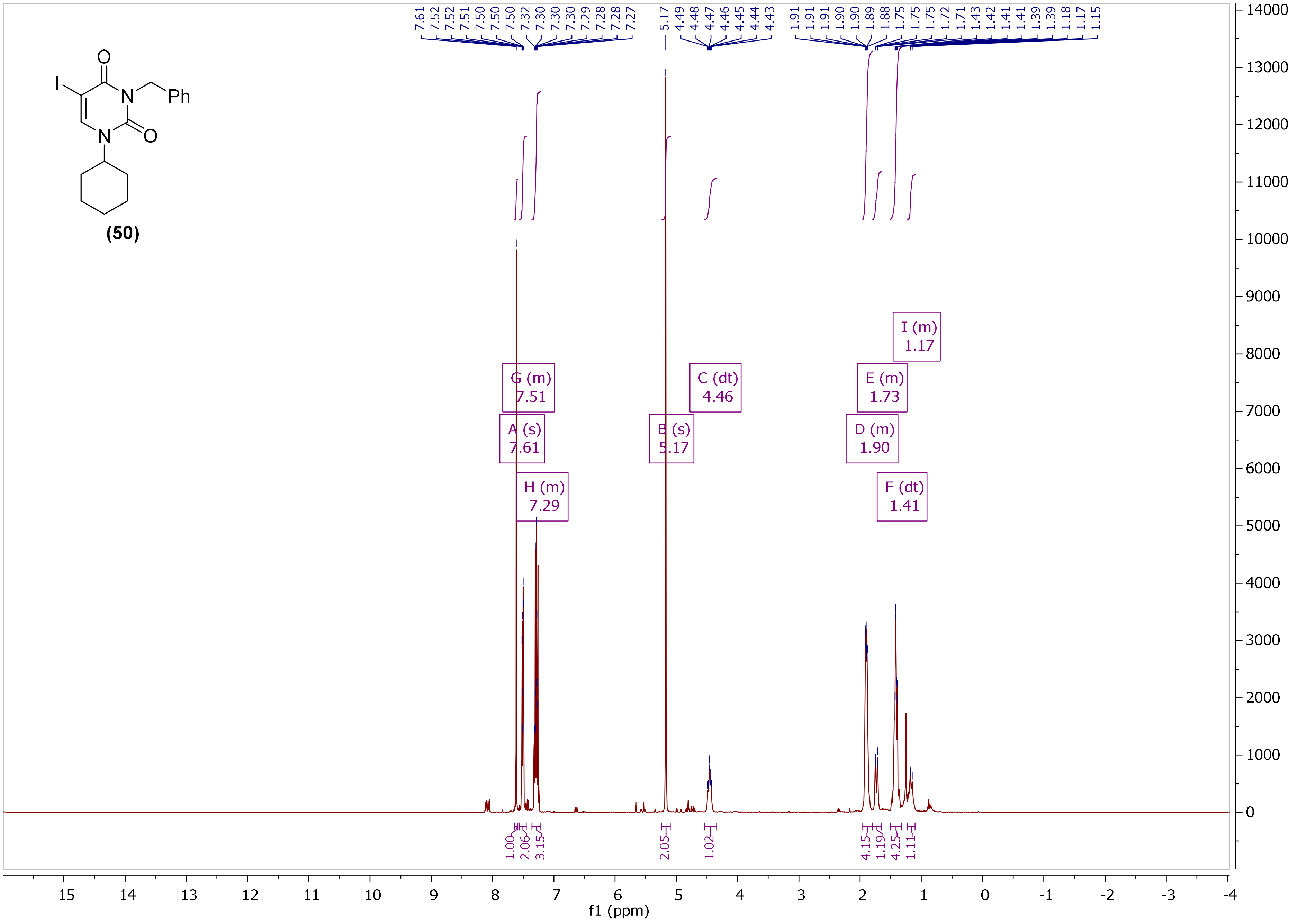

Figure S. 91 - ^{13}C NMR Spectra (101 MHz, $CDCl_3$) - 3-benzyl-5-chloro-1-cyclohexylpyrimidine-2,4(1H,3H)-dione – **50**

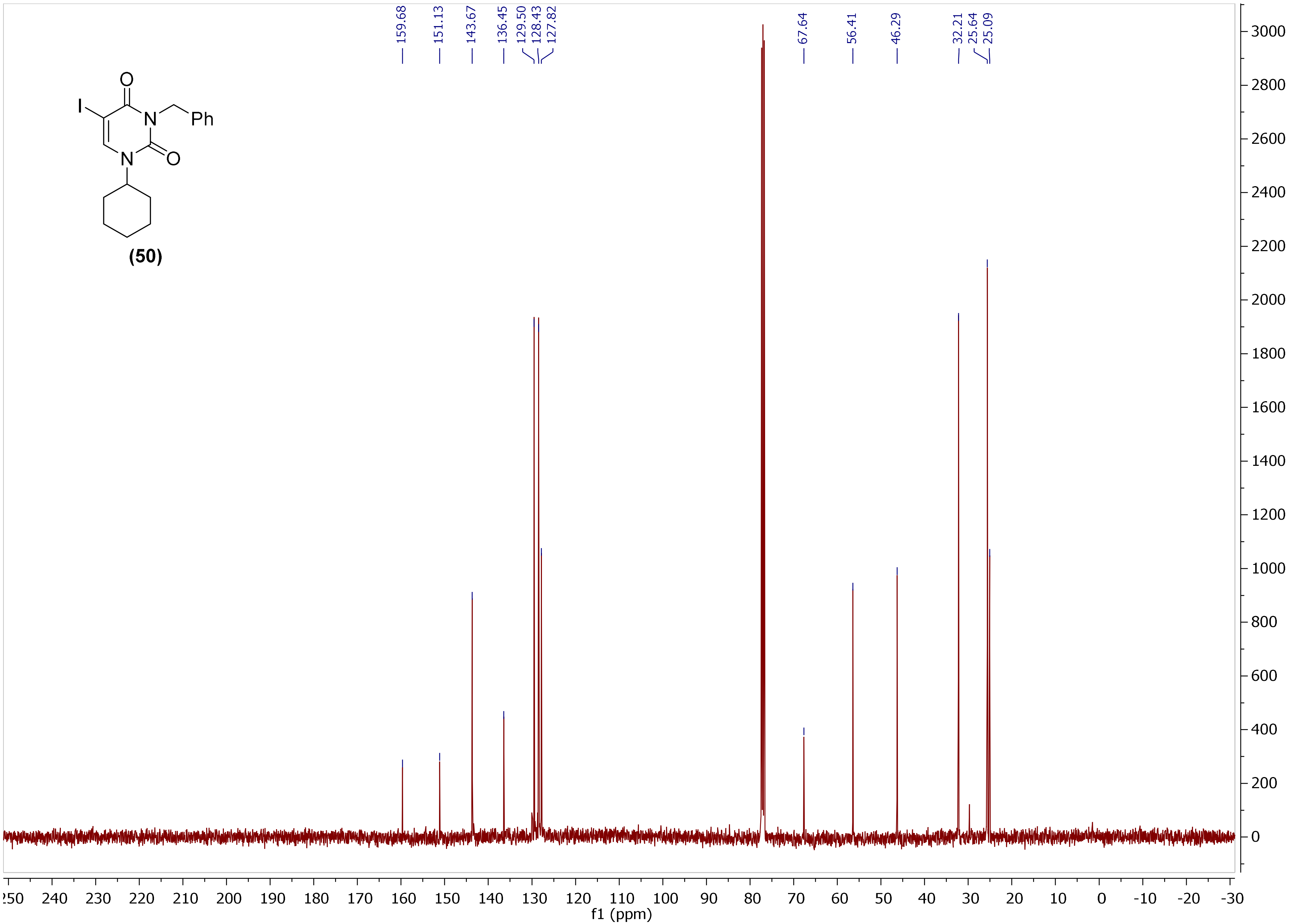

Figure S. 92 - ^{1}H-NMR Spectrum (400 MHz, $CDCl_3$) - 1-(3,5-Di-*O*-(4-methylbenzoyl)-2-deoxy-β-*D*-ribofuranosyl)-3-benzyl-2,4-dioxo-1,2,3,4-tetrahydropyrimidine-5-carboxylic acid – **16b**

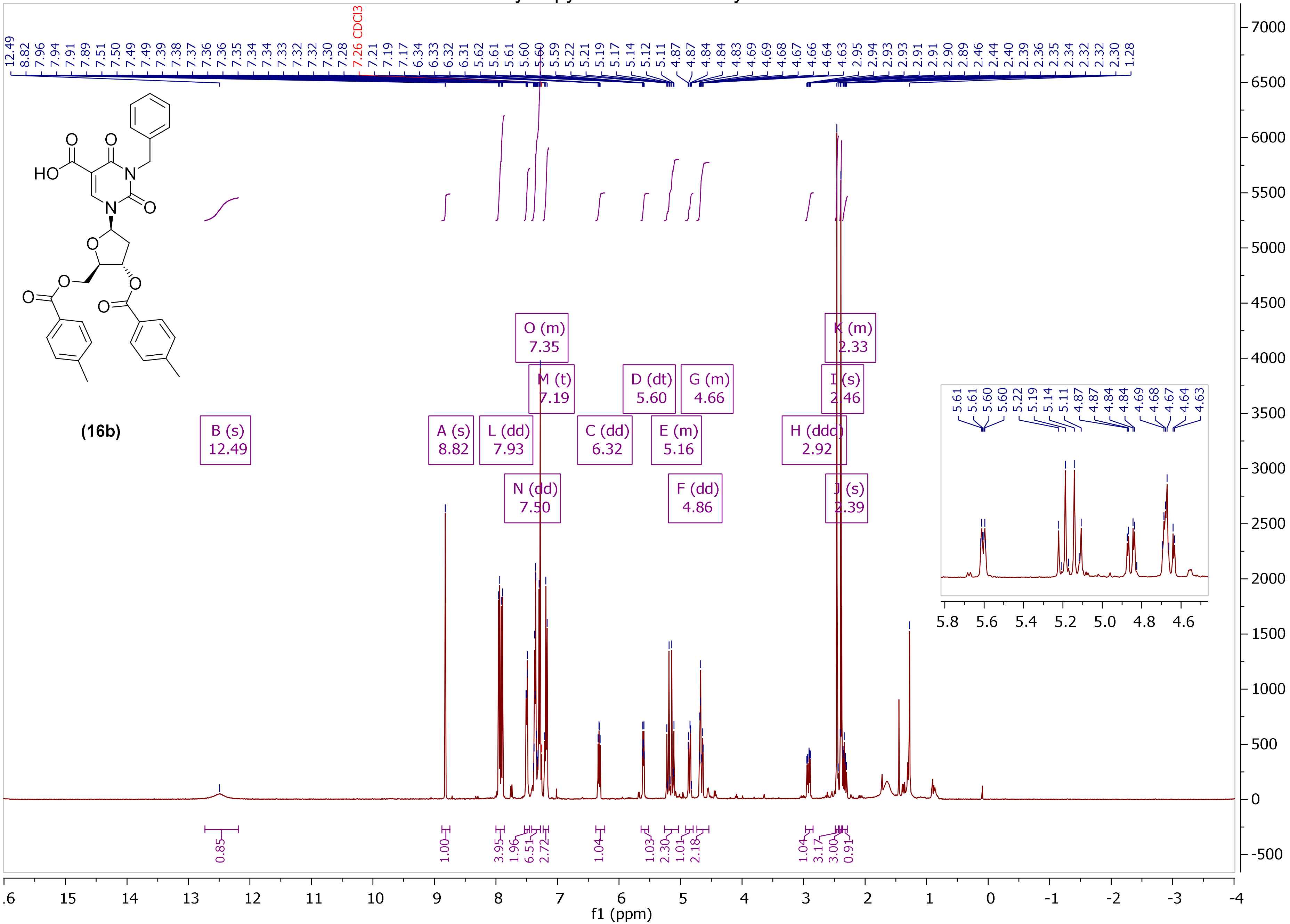

Figure S. 93 - ^{13}C NMR Spectra (101 MHz, $CDCl_3$) - 1-(3,5-Di-*O*-(4-methylbenzoyl)-2-deoxy-β-*D*-ribofuranosyl)-3-benzyl-2,4-dioxo-1,2,3,4-tetrahydropyrimidine-5-carboxylic acid – **16b**

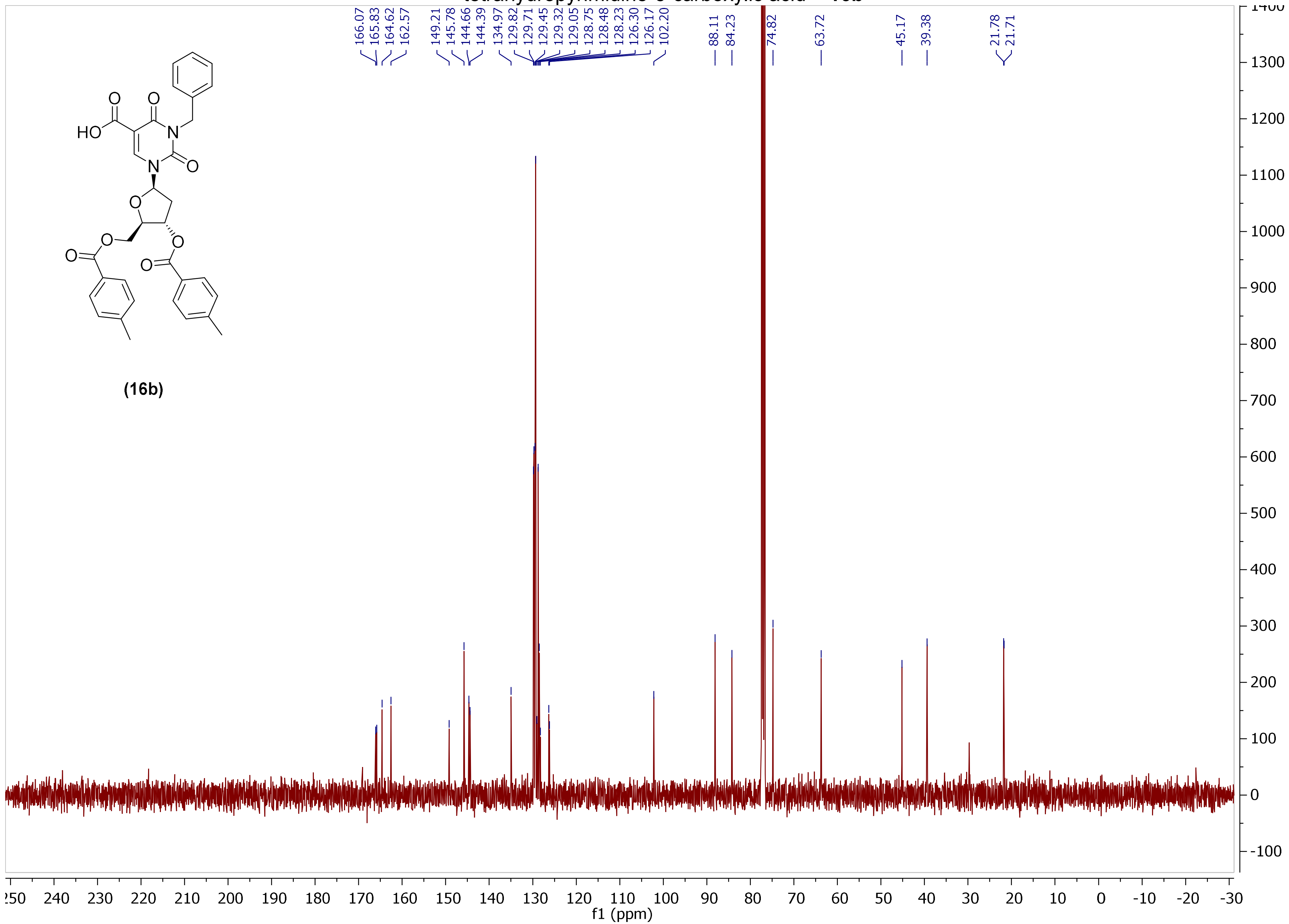

Figure S. 94 - ^{1}H-NMR Spectrum (400 MHz, $CDCl_3$) - 1-(2,3,5-Tri-*O*-acetyl-β-*D*-ribofuranosyl)-3-benzyl-2,4-dioxo-1,2,3,4-tetrahydropyrimidine-5-carboxylic acid - **16c**

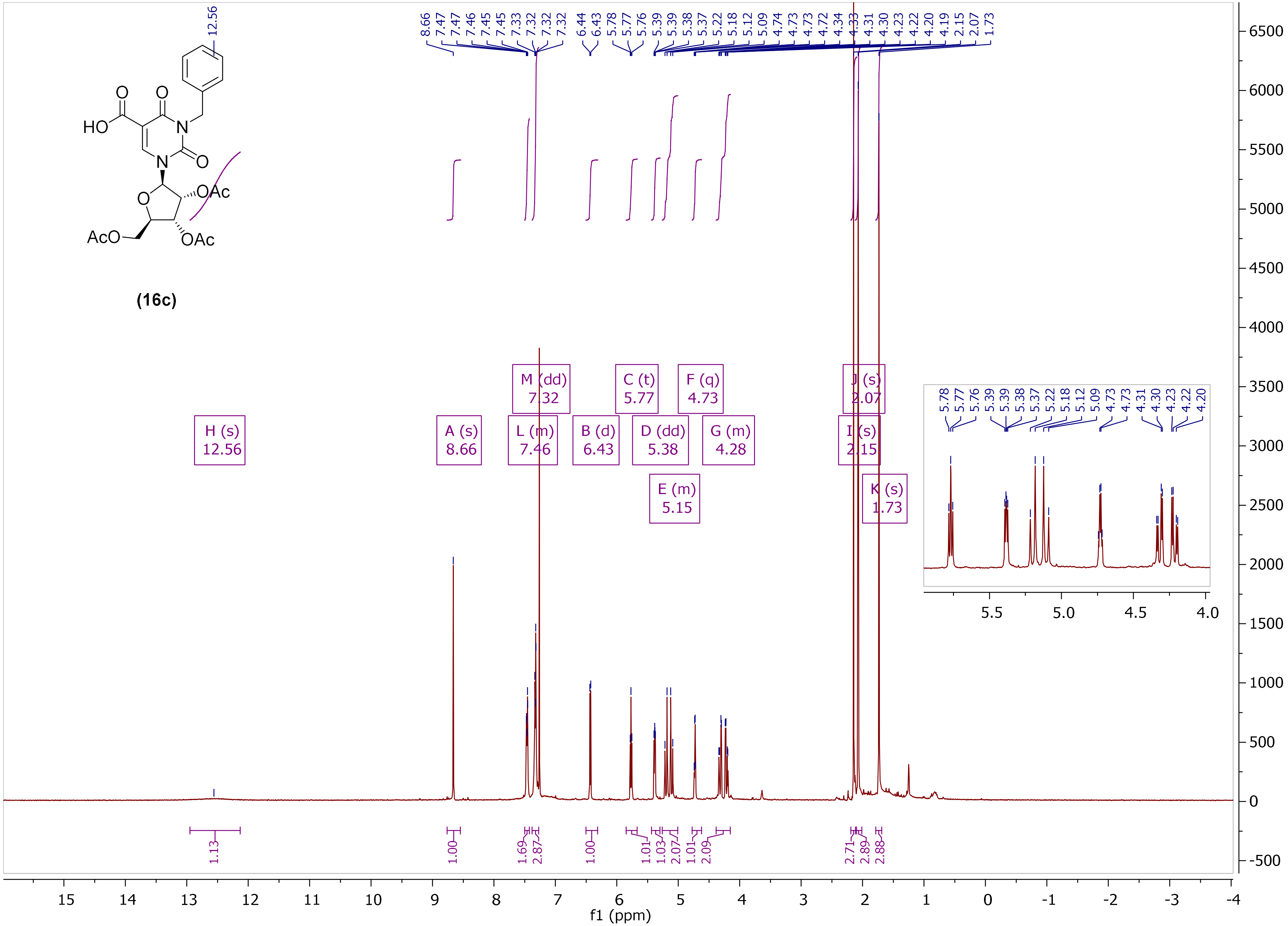

Figure S. 95 - ^{13}C NMR Spectra (101 MHz, $CDCl_3$) - 1-(2,3,5-Tri-*O*-acetyl-β-*D*-ribofuranosyl)-3-benzyl-2,4-dioxo-1,2,3,4-tetrahydropyrimidine-5-carboxylic acid – **16c**

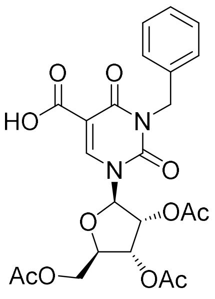

(16c)

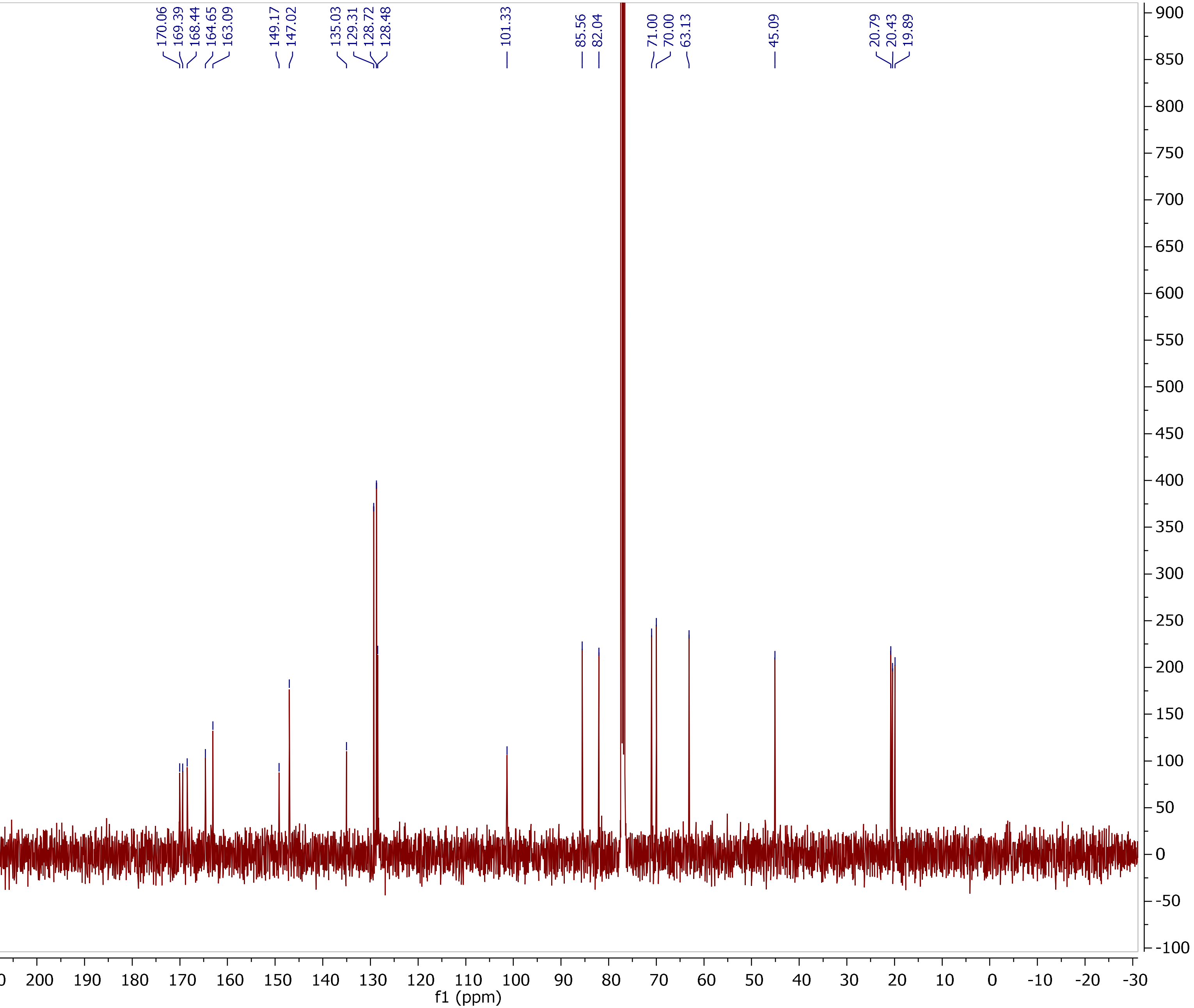

Figure S. 96 - ^{1}H-NMR Spectrum (400 MHz, $CDCl_3$) - 1-(3,5-Di-*O*-benzoyl-2-deoxy-2-fluoro-β-*D*-arabinofuranosyl)-3-benzyl-2,4-dioxo-1,2,3,4-tetrahydropyrimidine-5-carboxylic Acid – **16d**

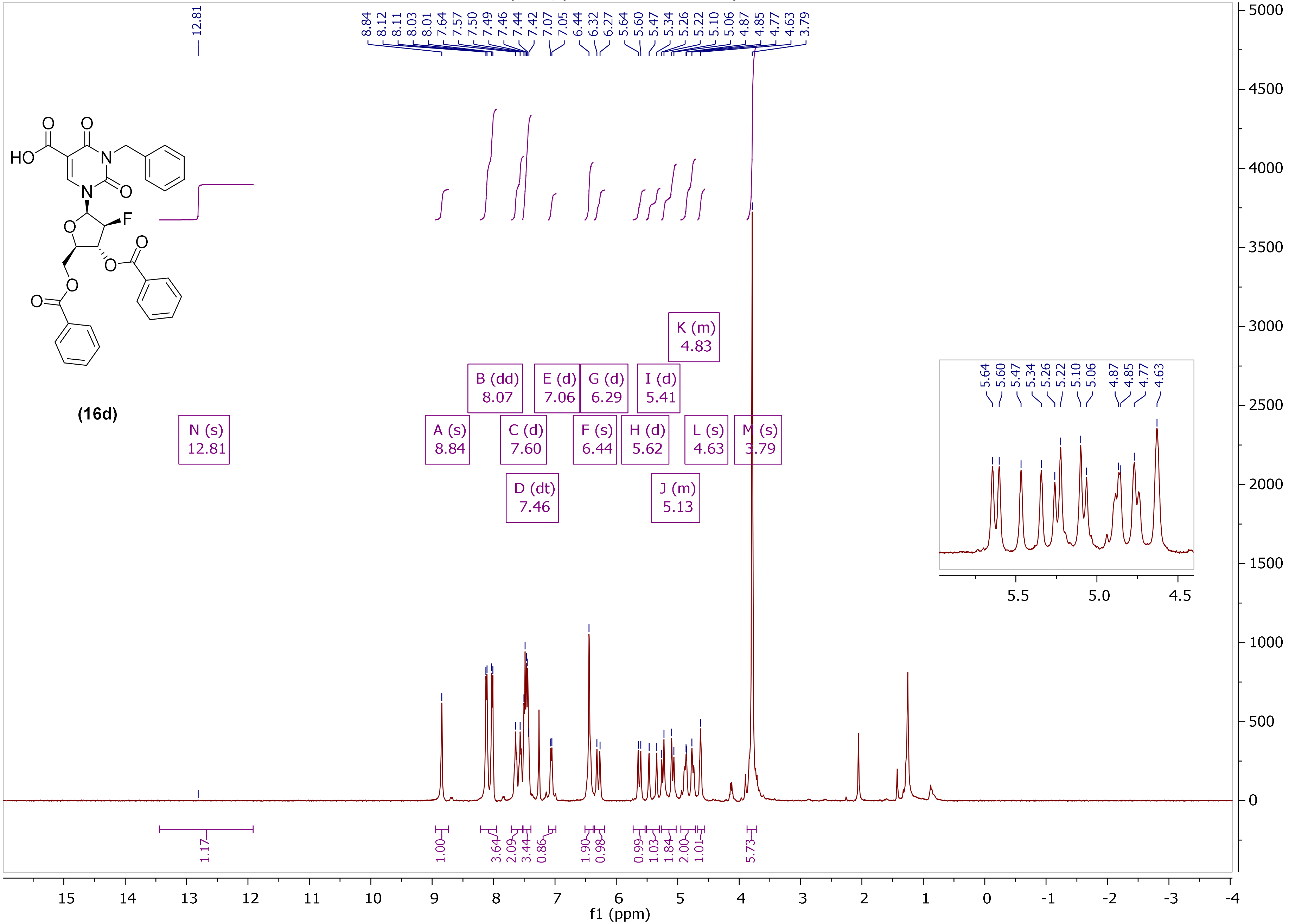

Figure S. 97 - ^{19}F NMR Spectra (377 MHz, $CDCl_3$) - 1-(3,5-Di-*O*-benzoyl-2-deoxy-2-fluoro-β-*D*-arabinofuranosyl)-3-benzyl-2,4-dioxo-1,2,3,4-tetrahydropyrimidine-5-carboxylic Acid – **16d**

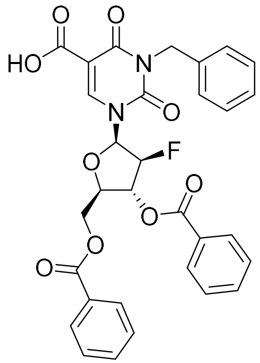

(16d)

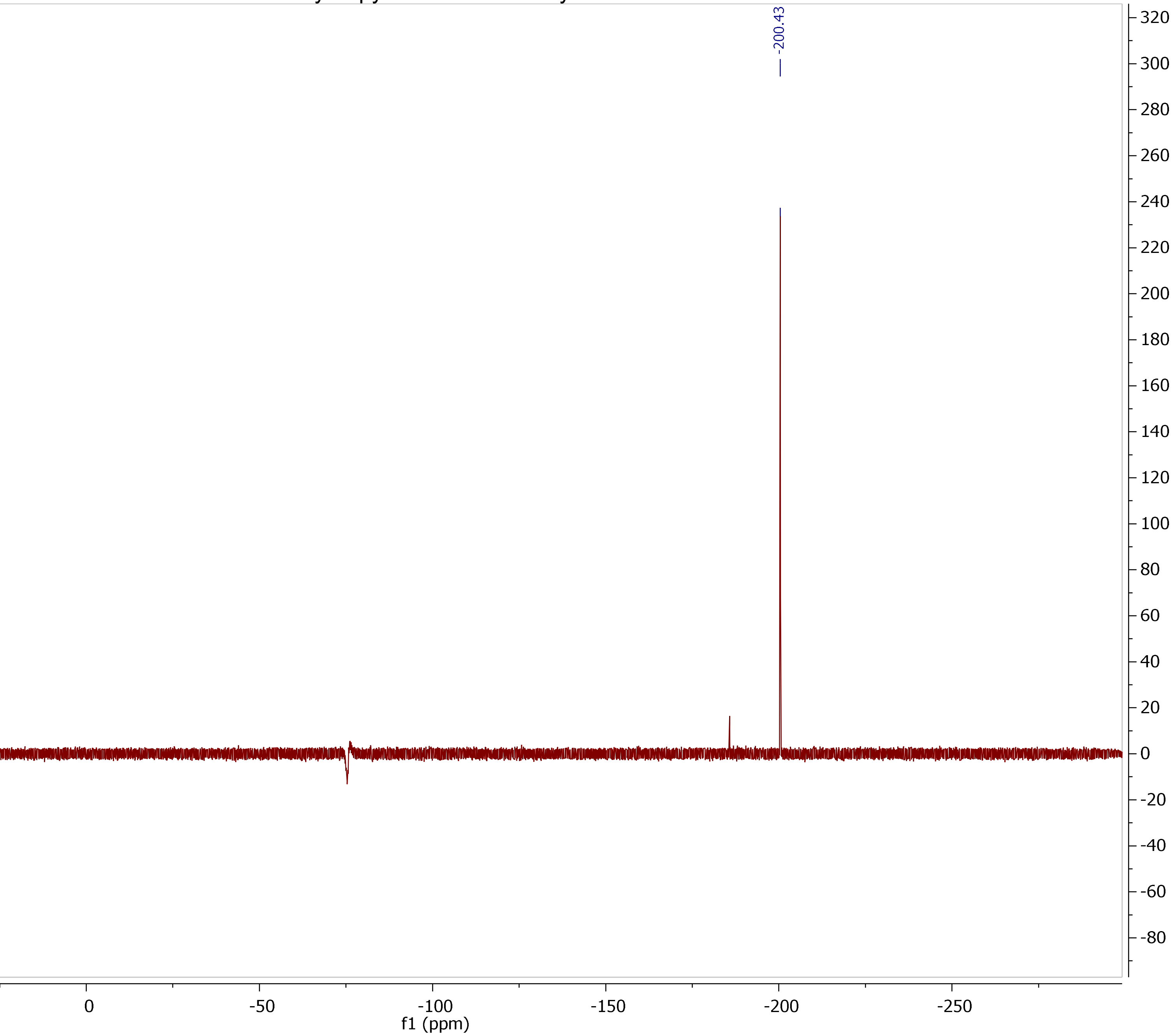

Figure S. 98 - ^{13}C NMR Spectra (101 MHz, $CDCl_3$) - 1-(3,5-Di-*O*-benzoyl-2-deoxy-2-fluoro-β-*D*-arabinofuranosyl)-3-benzyl-2,4-dioxo-1,2,3,4-tetrahydropyrimidine-5-carboxylic Acid – **16d**

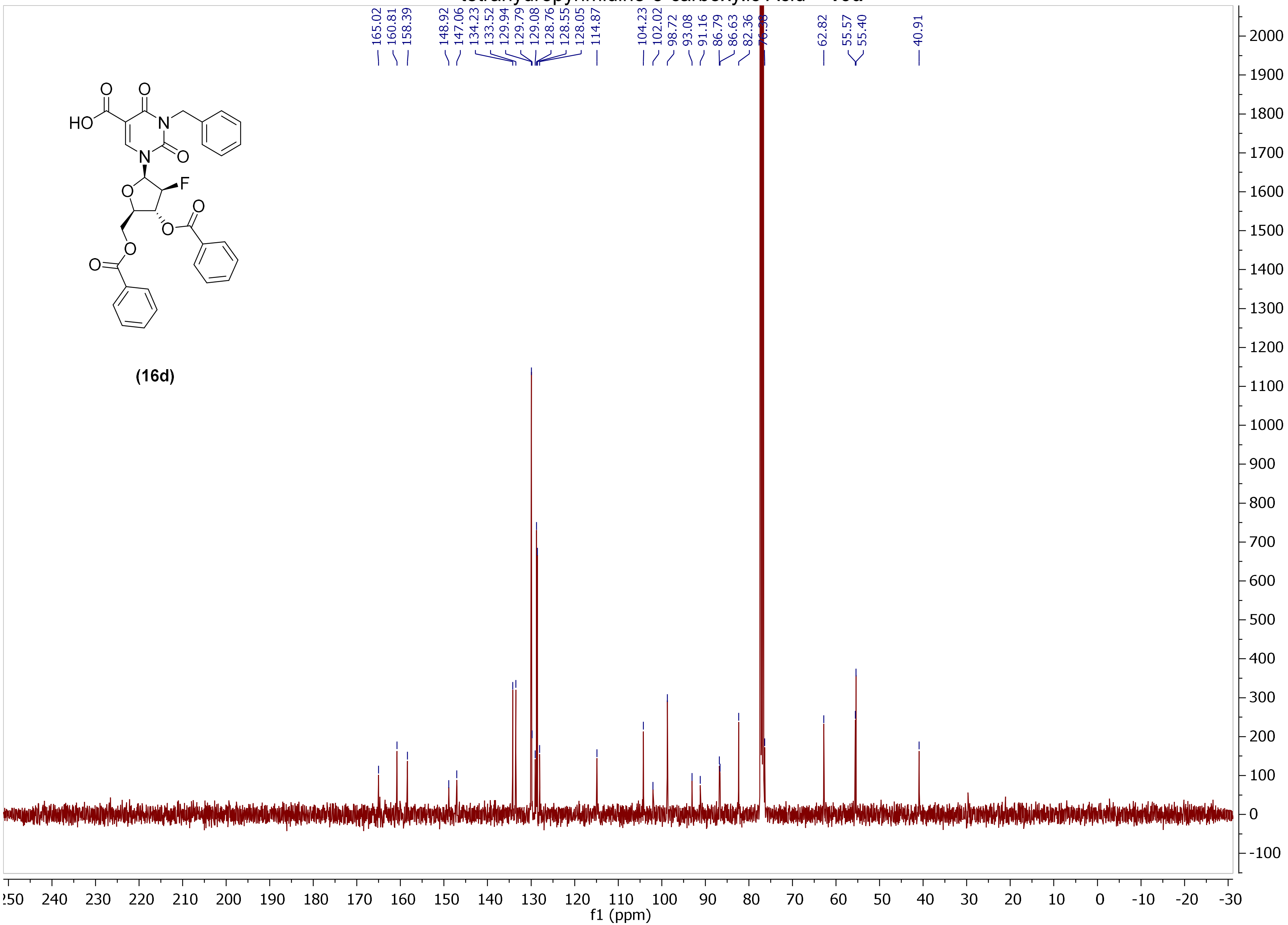

Figure S. 99 - ^{1}H-NMR Spectrum (400 MHz, $CDCl_3$) - 1-(3,5-Di-*O*-benzoyl-2-deoxy-2-fluoro-2-methyl-β-*D*-ribofuranosyl)-3-benzyl-2,4-dioxo-1,2,3,4-tetrahydropyrimidine-5-carboxylic acid – **16e**

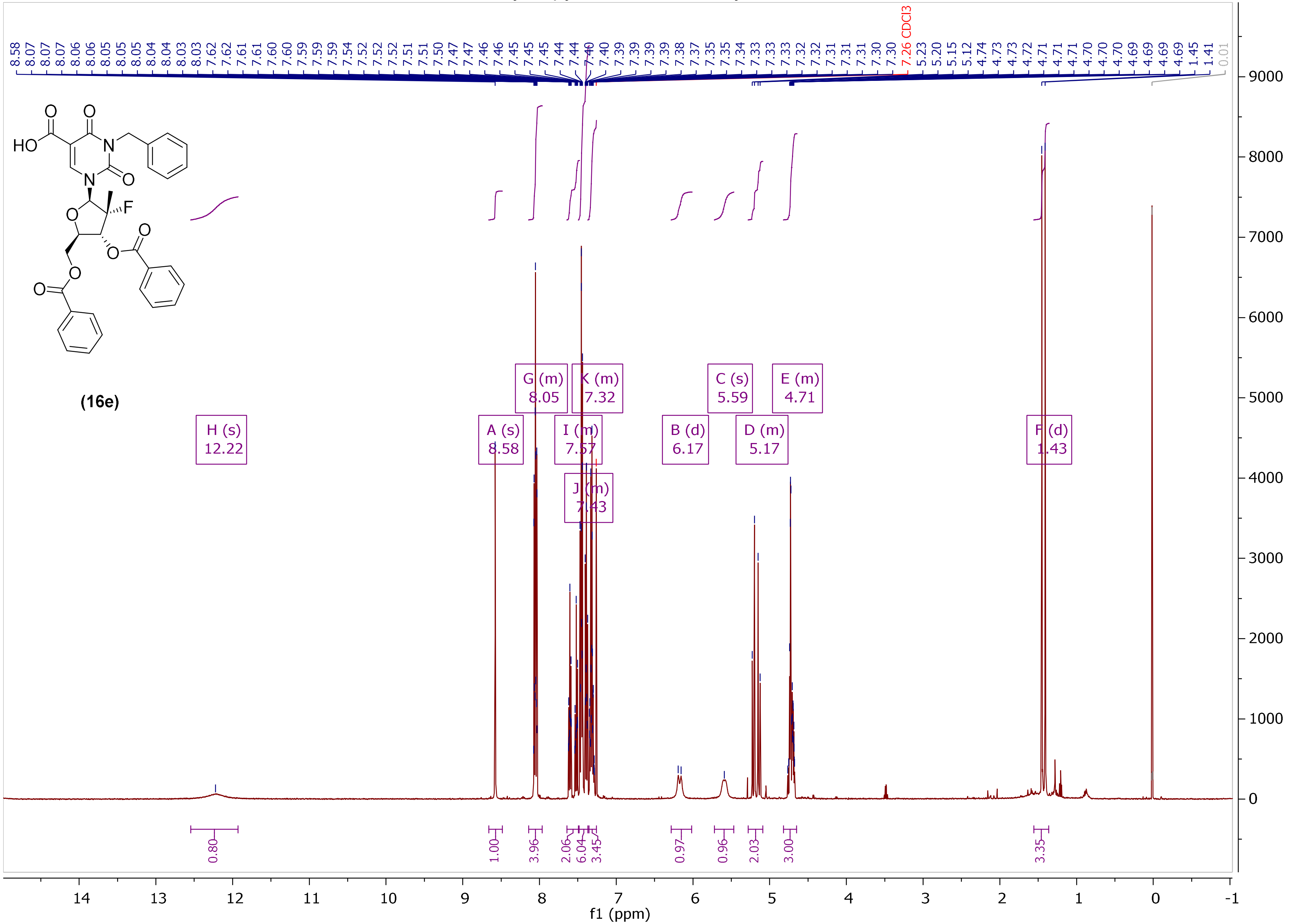

Figure S. 100 - ^{19}F NMR Spectra (377 MHz, $CDCl_3$) - 1-(3,5-Di-*O*-benzoyl-2-deoxy-2-fluoro-2-methyl-β-*D*-ribofuranosyl)-3-benzyl-2,4-dioxo-1,2,3,4-tetrahydropyrimidine-5-carboxylic acid – **16e**

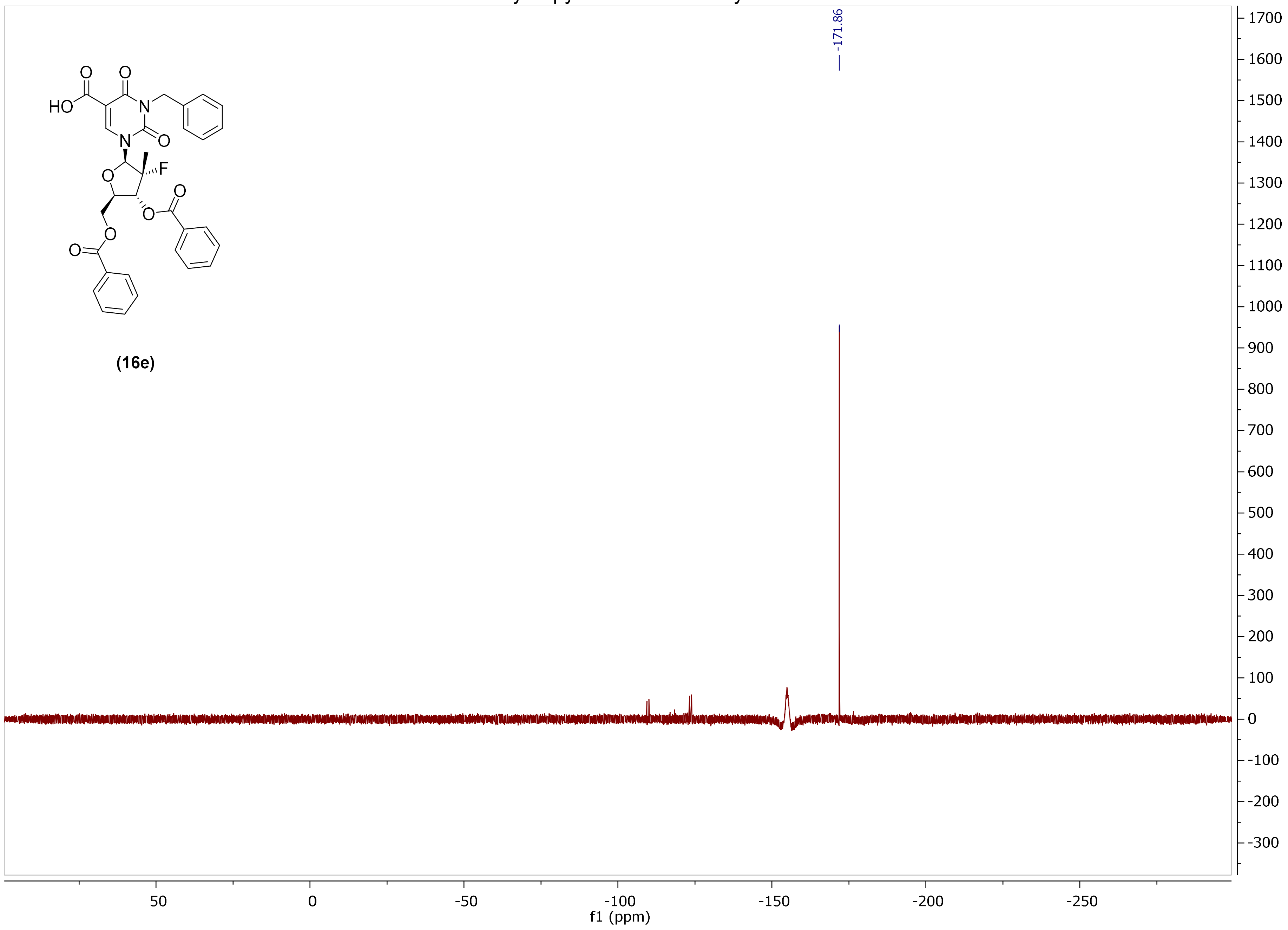

(16e)

Figure S. 101 – ^{13}C NMR Spectra (101 MHz, $CDCl_3$) - 1-(3,5-Di-*O*-benzoyl-2-deoxy-2-fluoro-2-methyl-β-*D*-ribofuranosyl)-3-benzyl-2,4-dioxo-1,2,3,4-tetrahydropyrimidine-5-carboxylic acid – **16e**

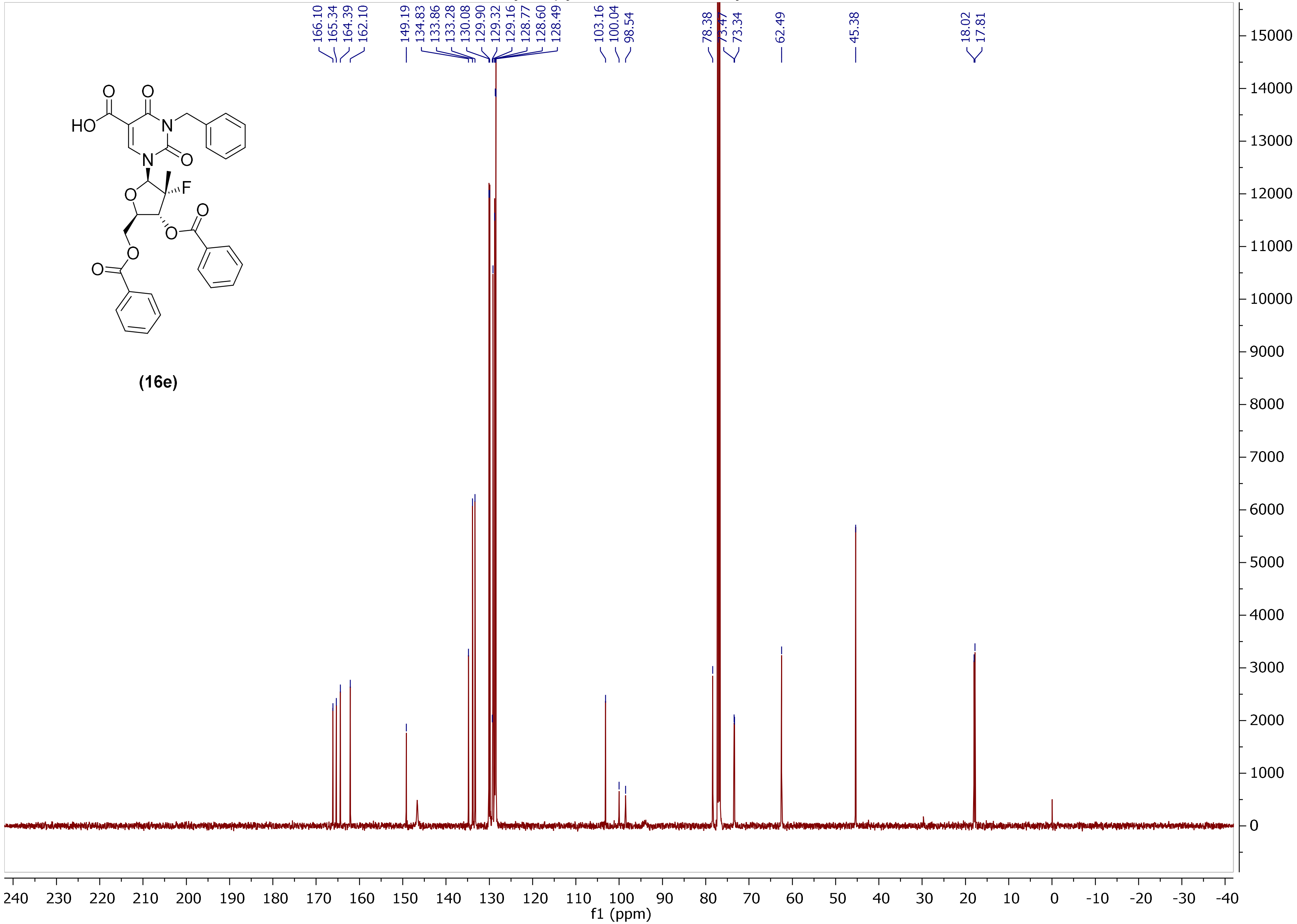

(16e)

Figure S. 102 - ^{1}H-NMR Spectrum (400 MHz, $CDCl_3$) - 1-(3,5-Di-*O*-benzoyl-2-deoxy-2-fluoro-2-methyl-α-*D*-ribofuranosyl)-3-benzyl-2,4-dioxo-1,2,3,4-tetrahydropyrimidine-5-carboxylic acid – (1-epi-**16e**)

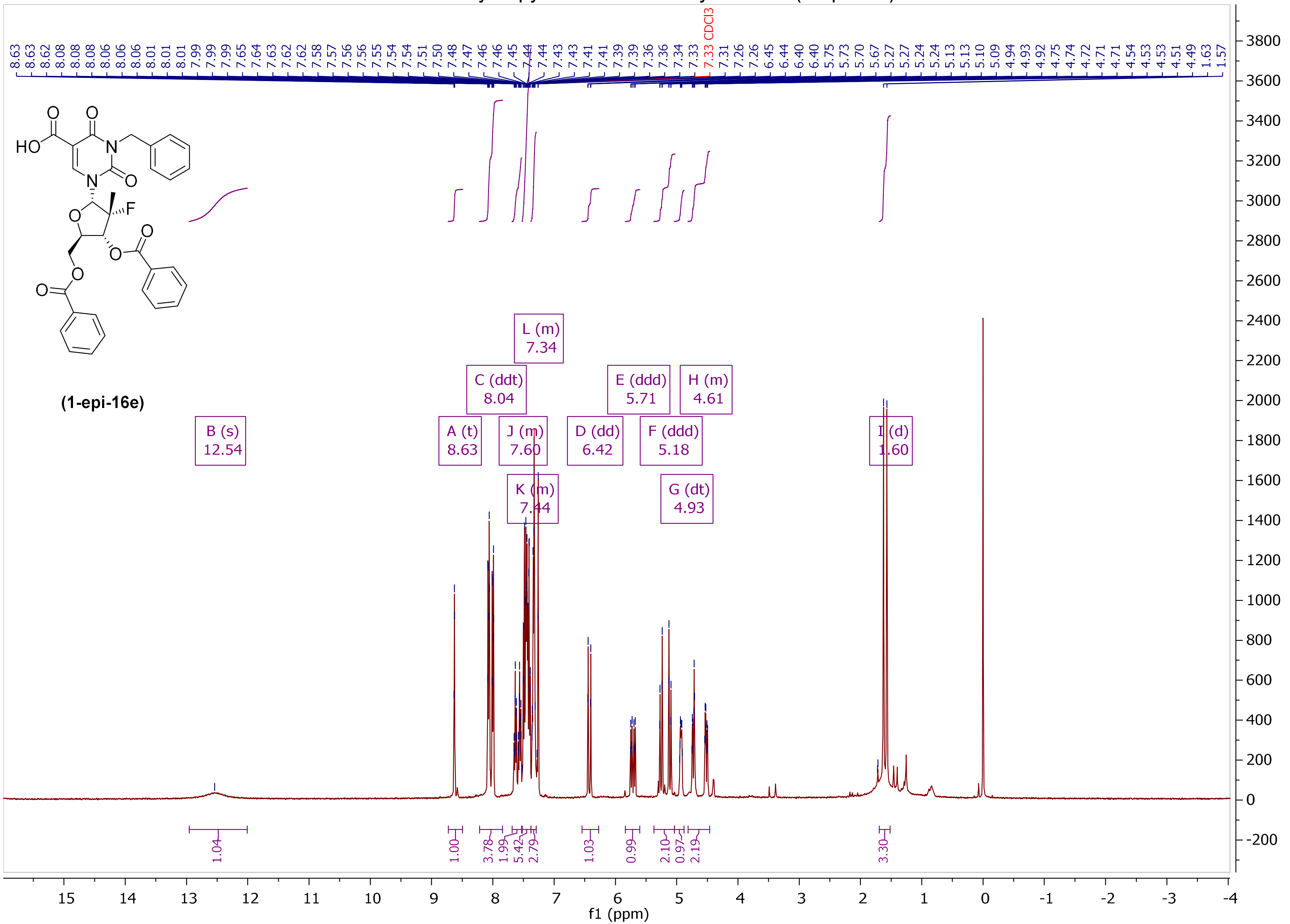

Figure S. 103 - Figure S. 104 - ^{19}F NMR Spectra (377 MHz, $CDCl_3$) - 1-(3,5-Di-*O*-benzoyl-2-deoxy-2-fluoro-2-methyl-α-*D*-ribofuranosyl)-3-benzyl-2,4-dioxo-1,2,3,4-tetrahydropyrimidine-5-carboxylic acid – (1-epi-**16e**)

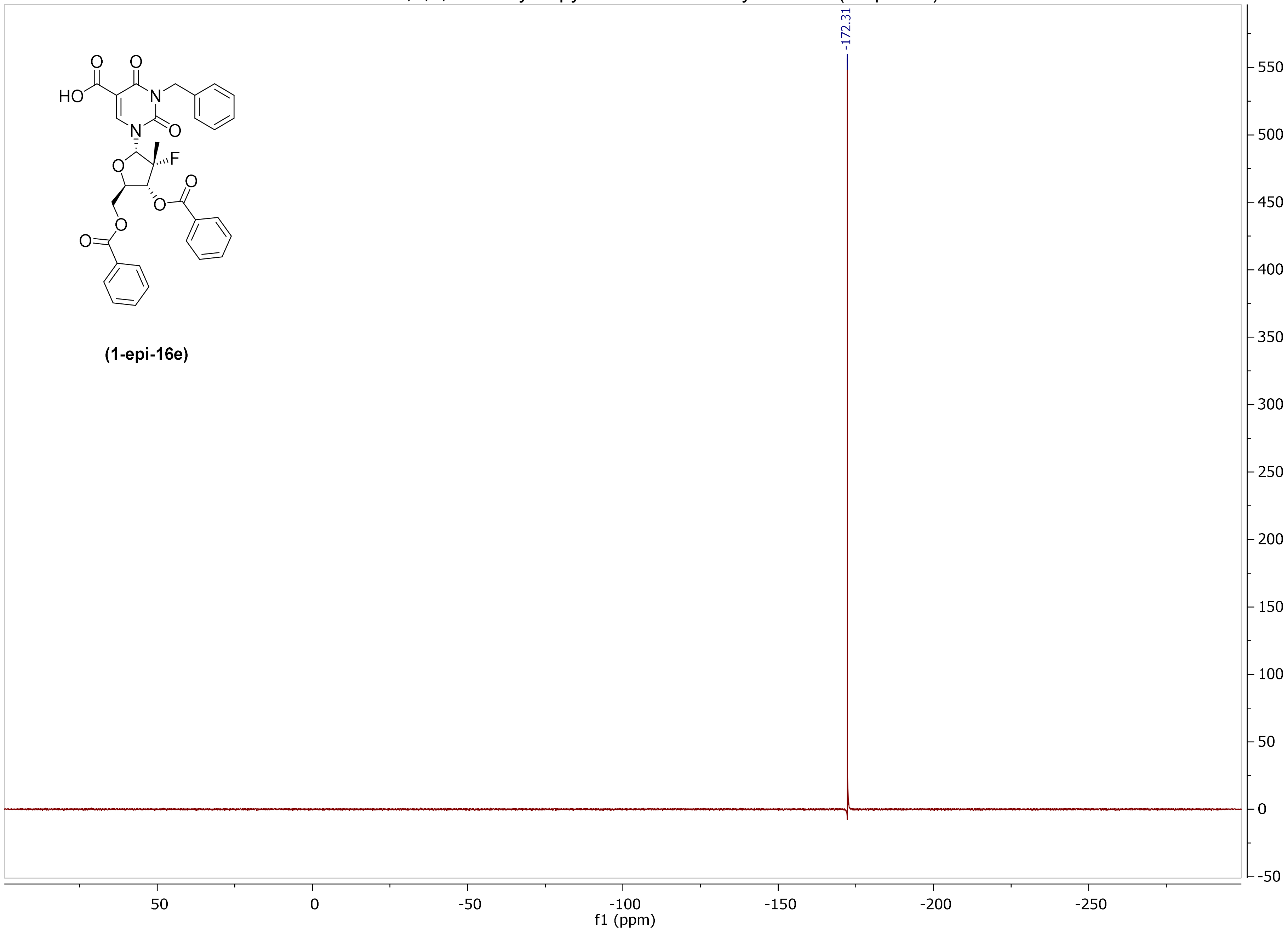

Figure S. 105 – ^{13}C NMR Spectra (101 MHz, $CDCl_3$) - 1-(3,5-Di-*O*-benzoyl-2-deoxy-2-fluoro-2-methyl-α-*D*-ribofuranosyl)-3-benzyl-2,4-dioxo-1,2,3,4-tetrahydropyrimidine-5-carboxylic acid – (1-epi-**16e**)

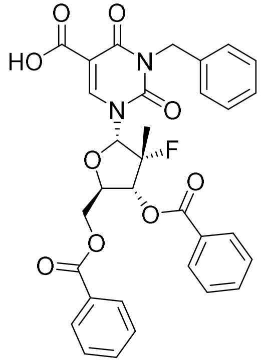

(1-epi-16e)

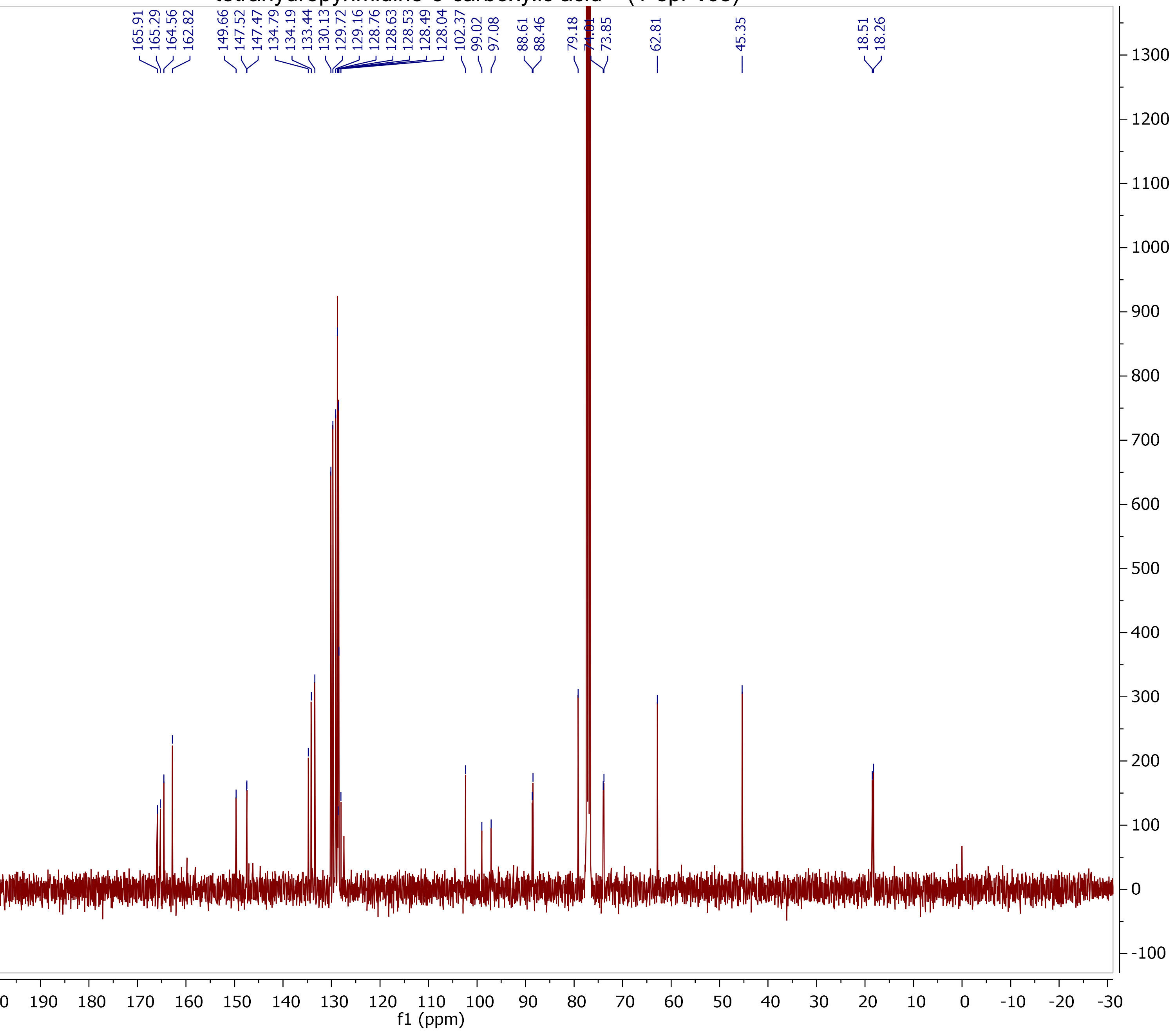

Figure S. 106 - ^{1}H-NMR Spectrum (400 MHz, $CDCl_3$) - 1-(3,5-Di-*O*-benzoyl-2-deoxy-2,2-difluoro-β-*D*-ribofuranosyl)-3-benzyl-2,4-dioxo-1,2,3,4-tetrahydropyrimidine-5-carboxylic acid – **16f**

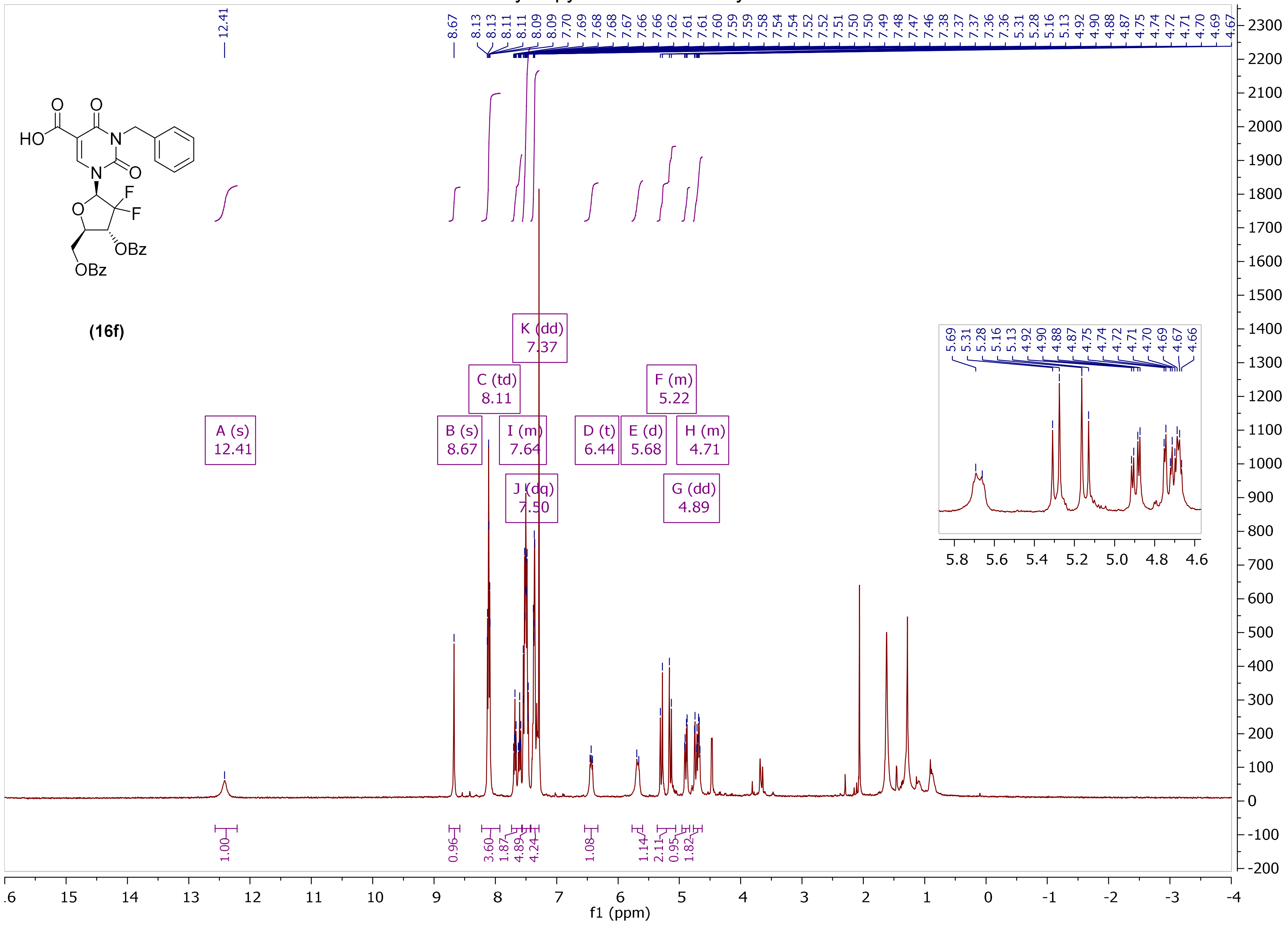

Figure S. 107 - ^{19}F NMR Spectra (377 MHz, $CDCl_3$) - 1-(3,5-Di-*O*-benzoyl-2-deoxy-2,2-difluoro-β-*D*-ribofuranosyl)-3-benzyl-2,4-dioxo-1,2,3,4-tetrahydropyrimidine-5-carboxylic acid – **16f**

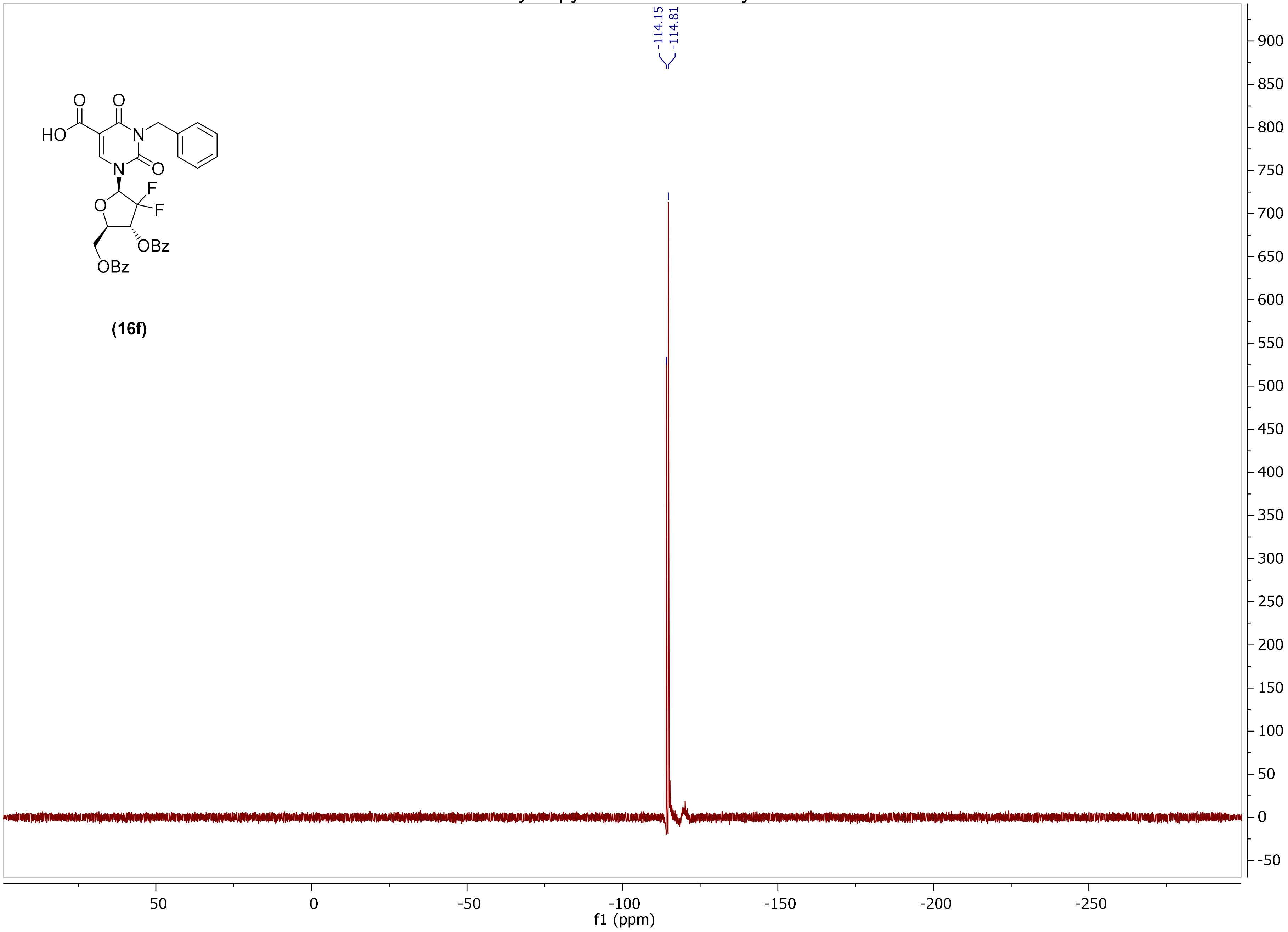

Figure S. 108 - ^{13}C NMR Spectra (101 MHz, $CDCl_3$) - 1-(3,5-Di-*O*-benzoyl-2-deoxy-2,2-difluoro-β-*D*-ribofuranosyl)-3-benzyl-2,4-dioxo-1,2,3,4-tetrahydropyrimidine-5-carboxylic acid – **16f**

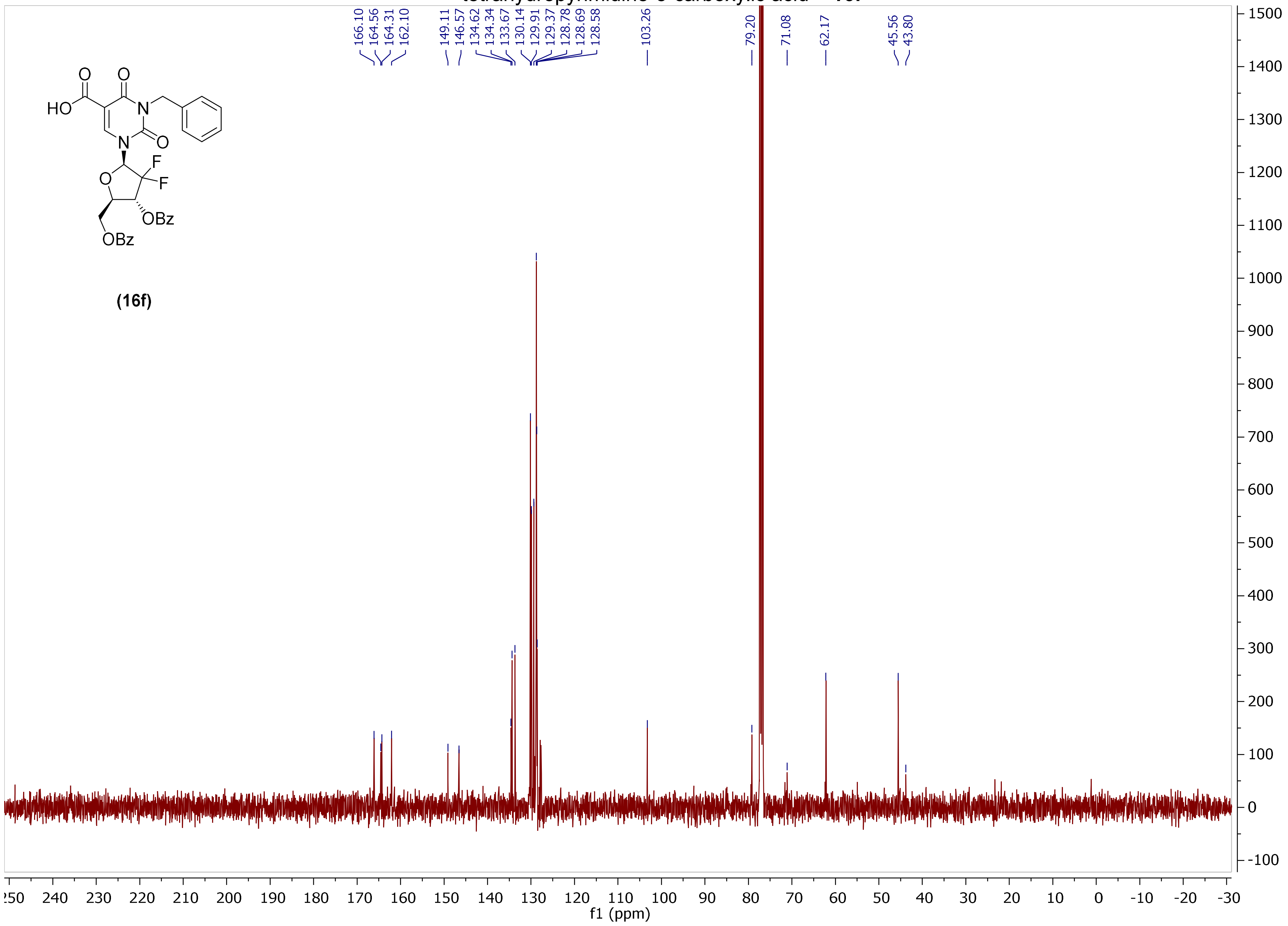

(16f)

Figure S. 109 - ^{1}H-NMR Spectrum (400 MHz, $CDCl_3$) - 1-(3,5-Di-*O*-(4-methylbenzoyl)-2-deoxy-β-*D*-ribofuranosyl)-3-benzyl-5-bromo-2,4-dioxo-1,2,3,4-tetrahydropyrimidine - **17b**

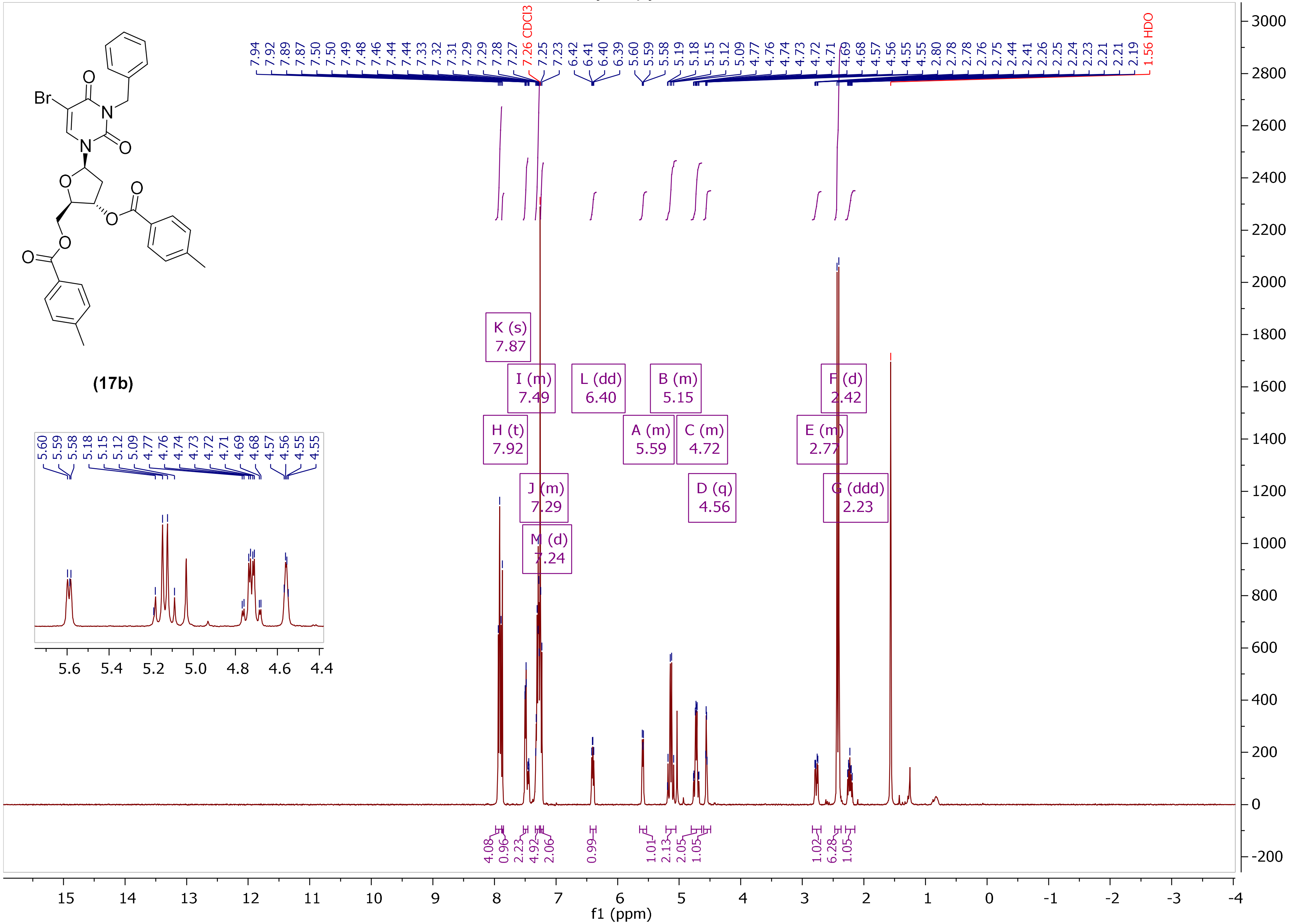

Figure S. 110 - ^{13}C NMR Spectra (101 MHz, $CDCl_3$) - 1-(3,5-Di-*O*-(4-methylbenzoyl)-2-deoxy-β-*D*-ribofuranosyl)-3-benzyl-5-bromo-2,4-dioxo-1,2,3,4-tetrahydropyrimidine - **17b**

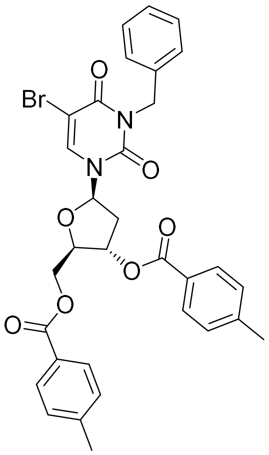

(17b)

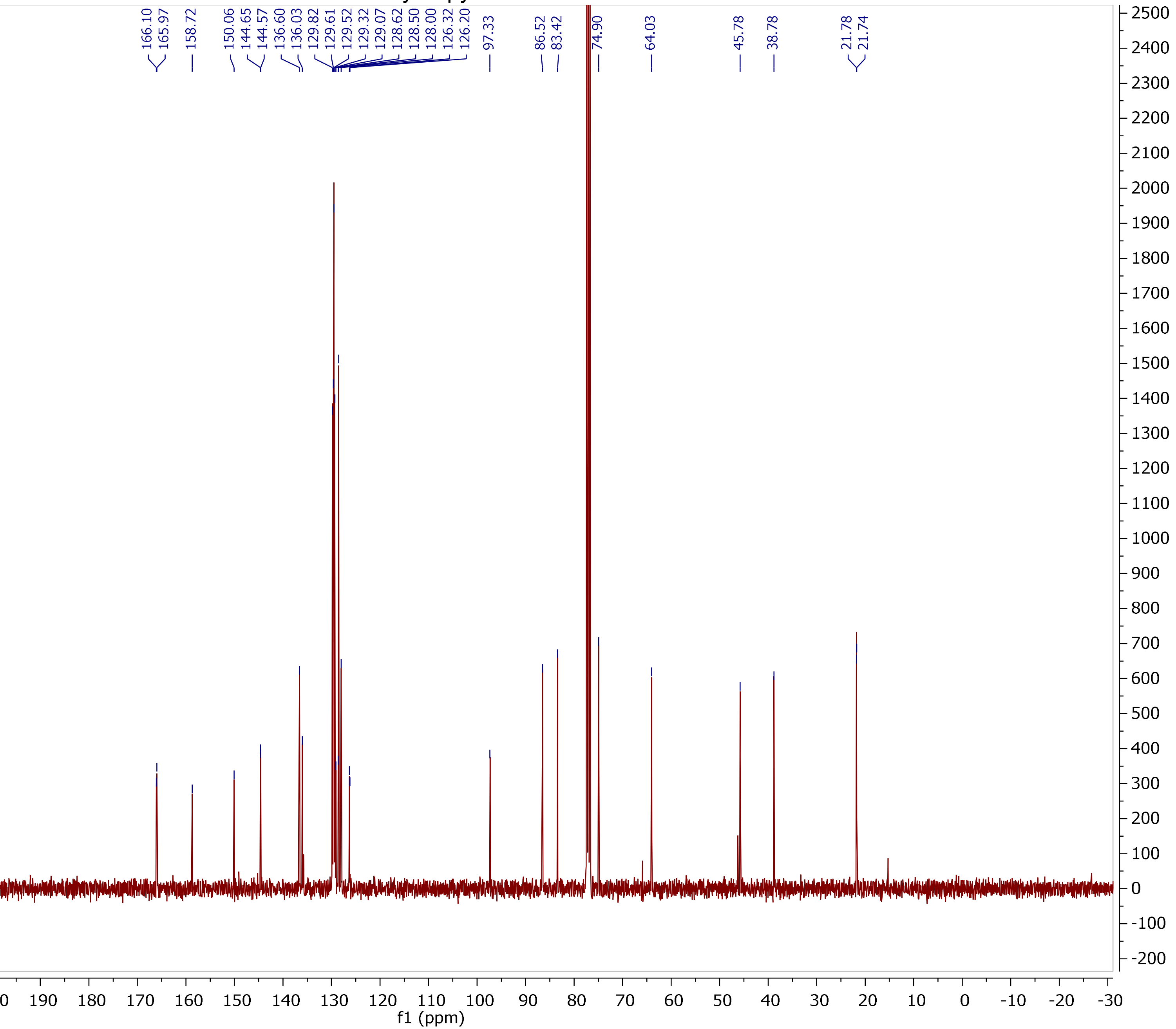

Figure S. 111 - ^{1}H-NMR Spectrum (400 MHz, $CDCl_3$) - 1-(2,3,5-Tri-*O*-acetyl-β-*D*-ribofuranosyl)-3-benzyl-5-bromo-2,4-dioxo-1,2,3,4-tetrahydropyrimidine – **17c**

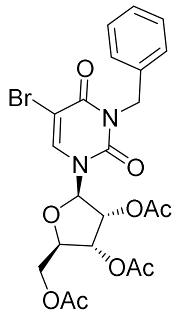

(17c)

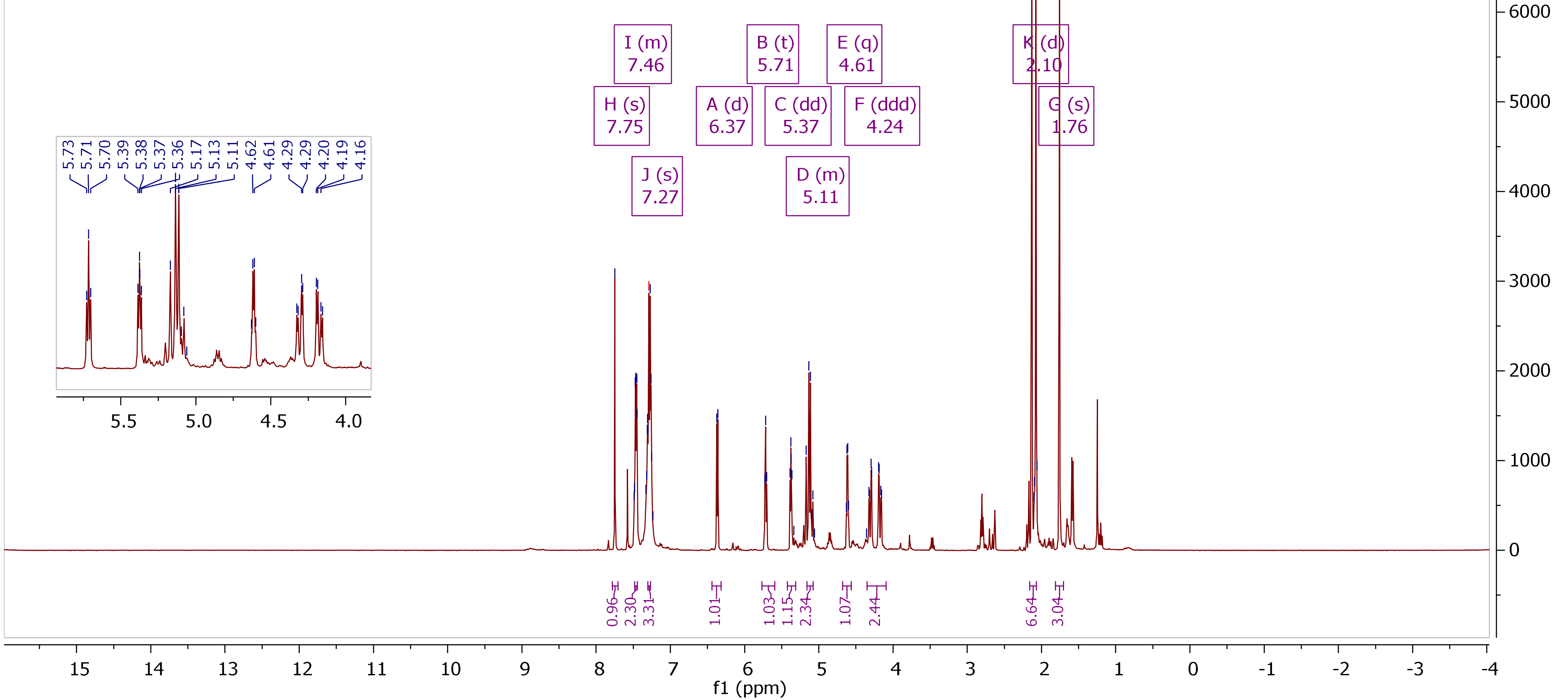

Figure S. 112 - ^{13}C NMR Spectra (101 MHz, $CDCl_3$) - 1-(2,3,5-Tri-*O*-acetyl-β-*D*-ribofuranosyl)-3-benzyl-5-bromo-2,4-dioxo-1,2,3,4-tetrahydropyrimidine – **17c**

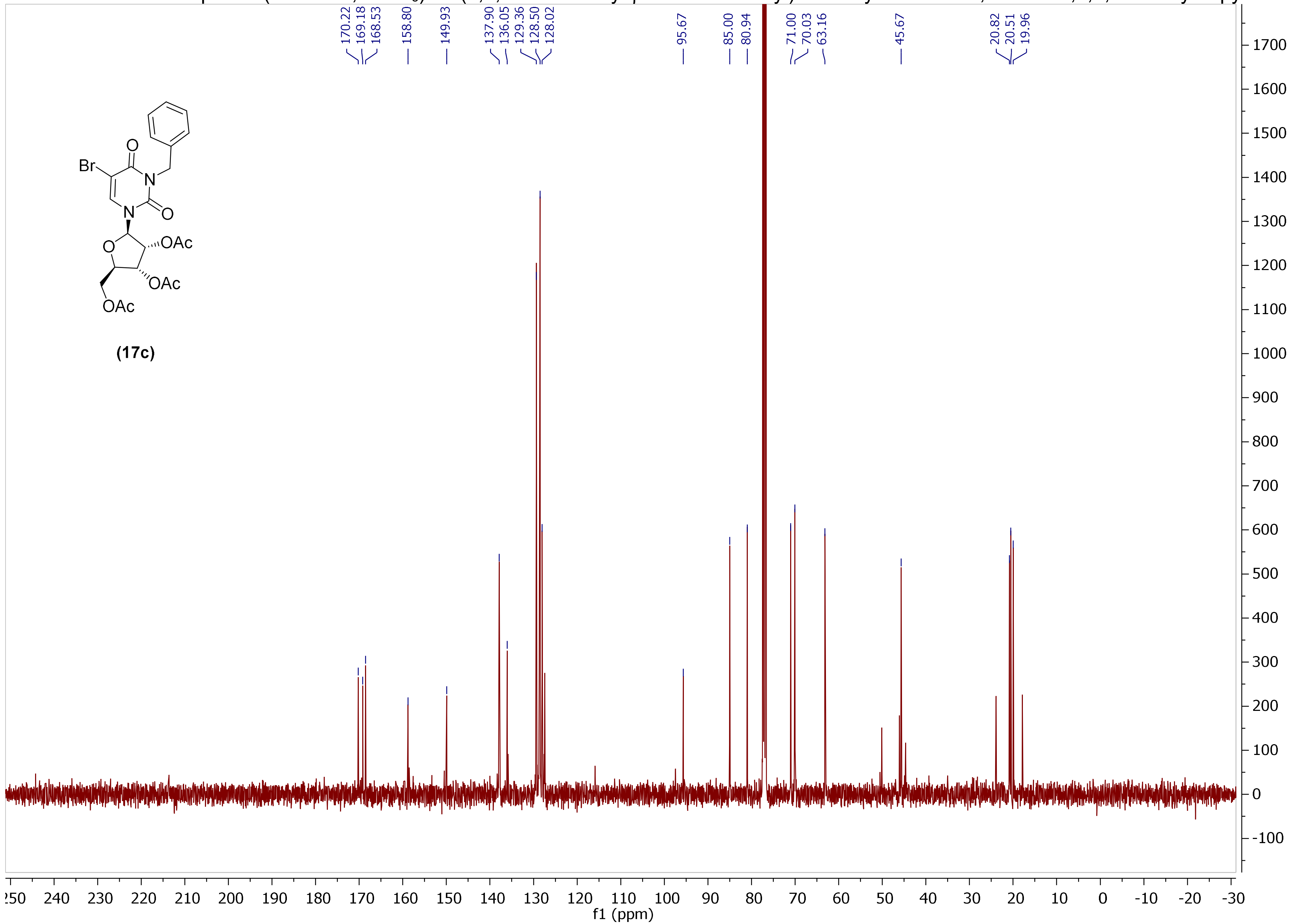

Figure S. 113 - ^{1}H-NMR Spectrum (400 MHz, $CDCl_3$) - 1-(3,5-Di-*O*-benzoyl-2-deoxy-2-fluoro-β-*D*-arabinofuranosyl)-3-benzyl-5-bromo-2,4-dioxo-1,2,3,4-tetrahydropyrimidine – **17d**

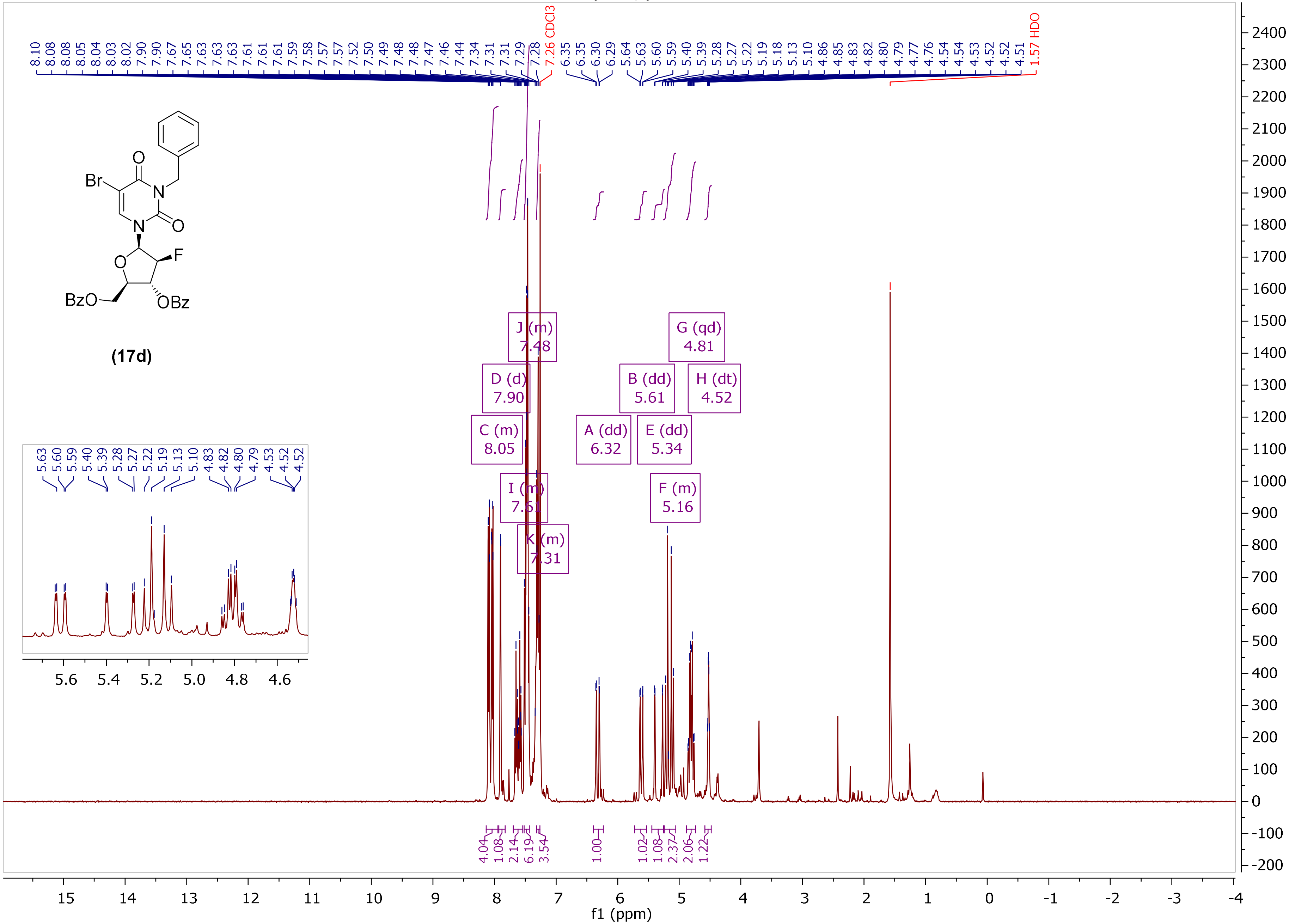

Figure S. 114 - ^{19}F NMR Spectra (377 MHz, $CDCl_3$) - 1-(3,5-Di-*O*-benzoyl-2-deoxy-2-fluoro-β-*D*-arabinofuranosyl)-3-benzyl-5-bromo-2,4-dioxo-1,2,3,4-tetrahydropyrimidine – **17d**

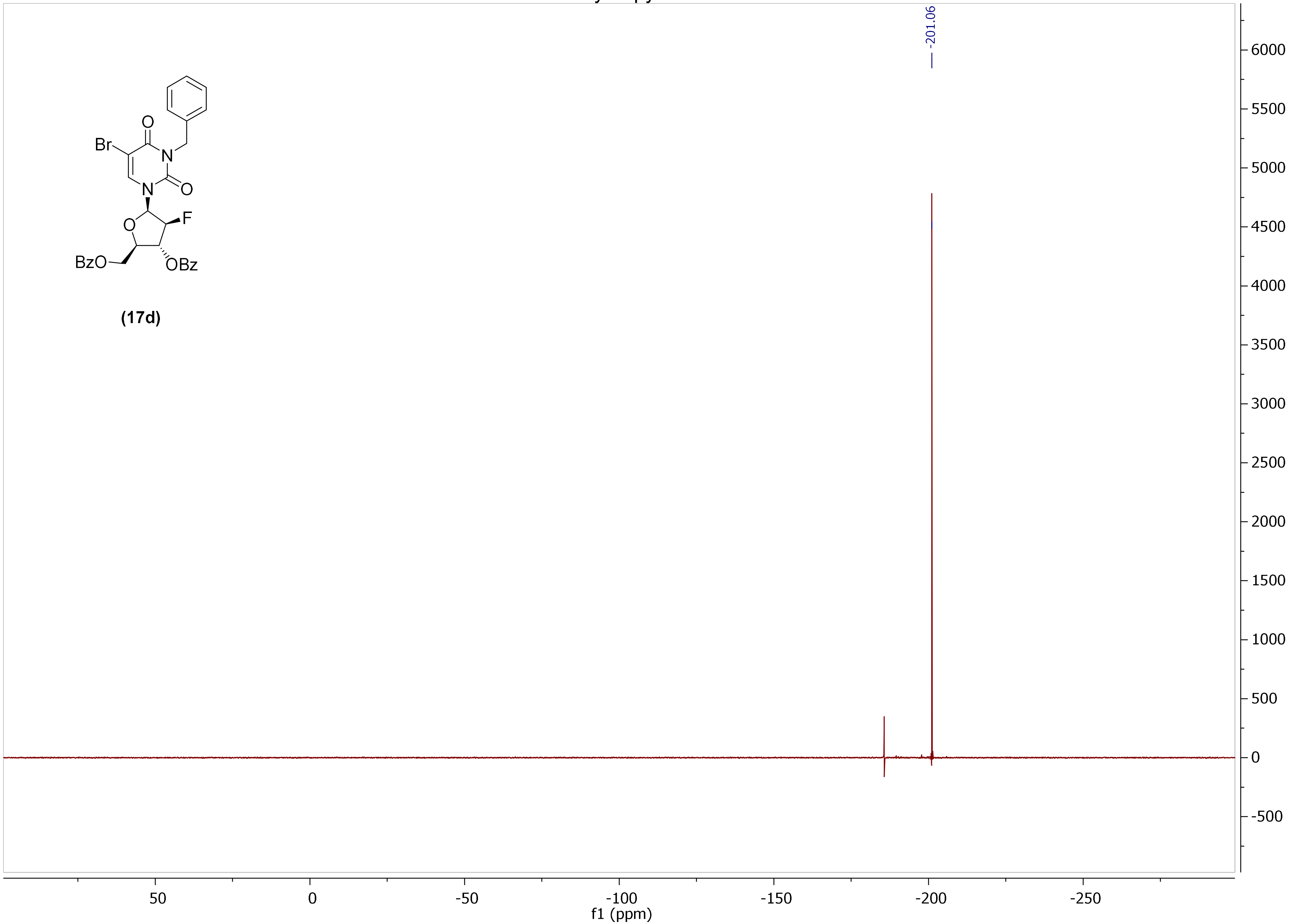

Figure S. 115 - ^{13}C NMR Spectra (101 MHz, $CDCl_3$) - 1-(3,5-Di-*O*-benzoyl-2-deoxy-2-fluoro-β-*D*-arabinofuranosyl)-3-benzyl-5-bromo-2,4-dioxo-1,2,3,4-tetrahydropyrimidine – **17d**

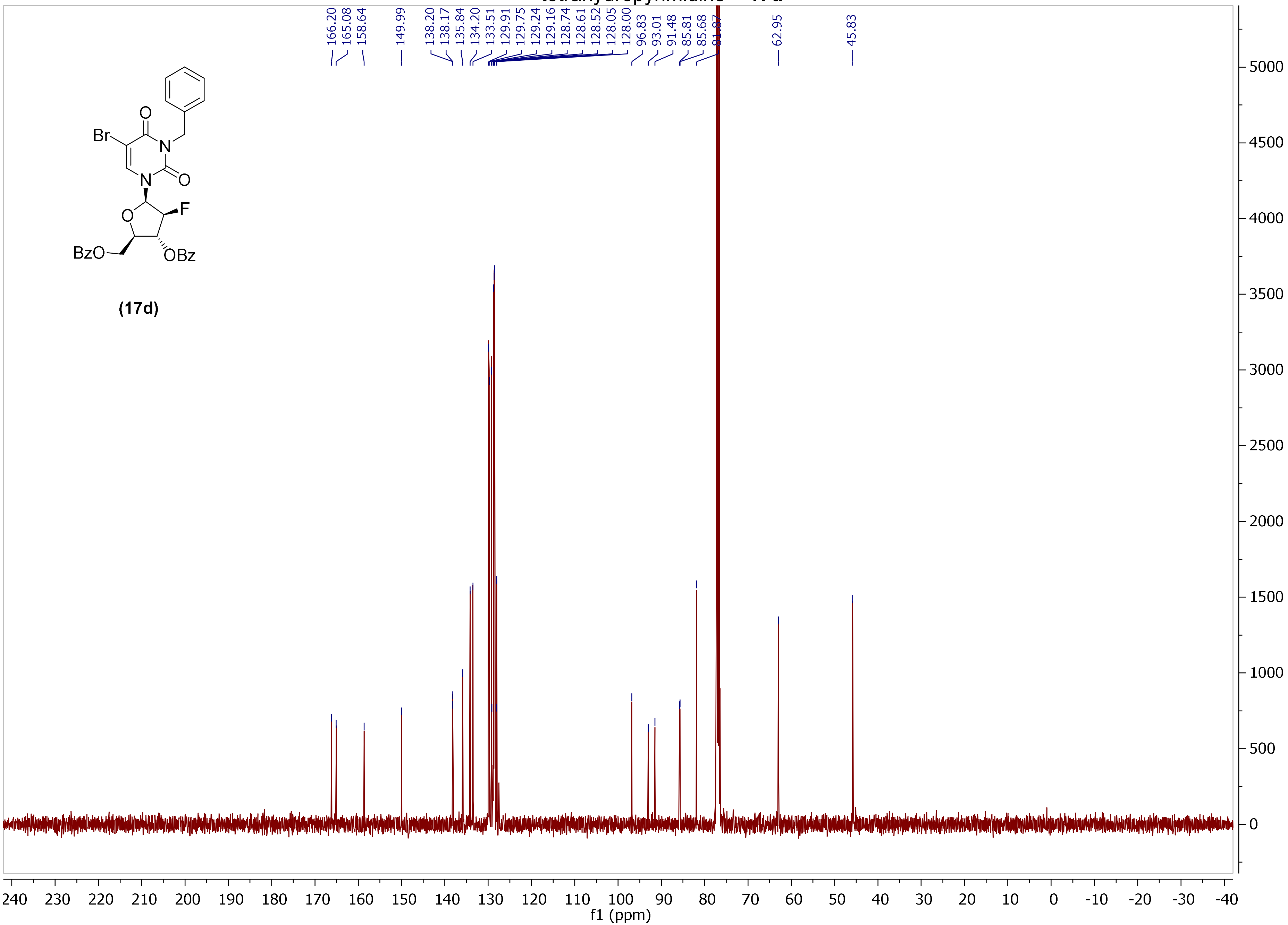

Figure S. 116 - ^{1}H-NMR Spectrum (400 MHz, $CDCl_3$) - 1-(3,5-Di-*O*-benzoyl-2-deoxy-2-fluoro-2-methyl-β-*D*-ribofuranosyl)-3-benzyl-5-bromo-2,4-dioxo-1,2,3,4-tetrahydropyrimidine – **17e**

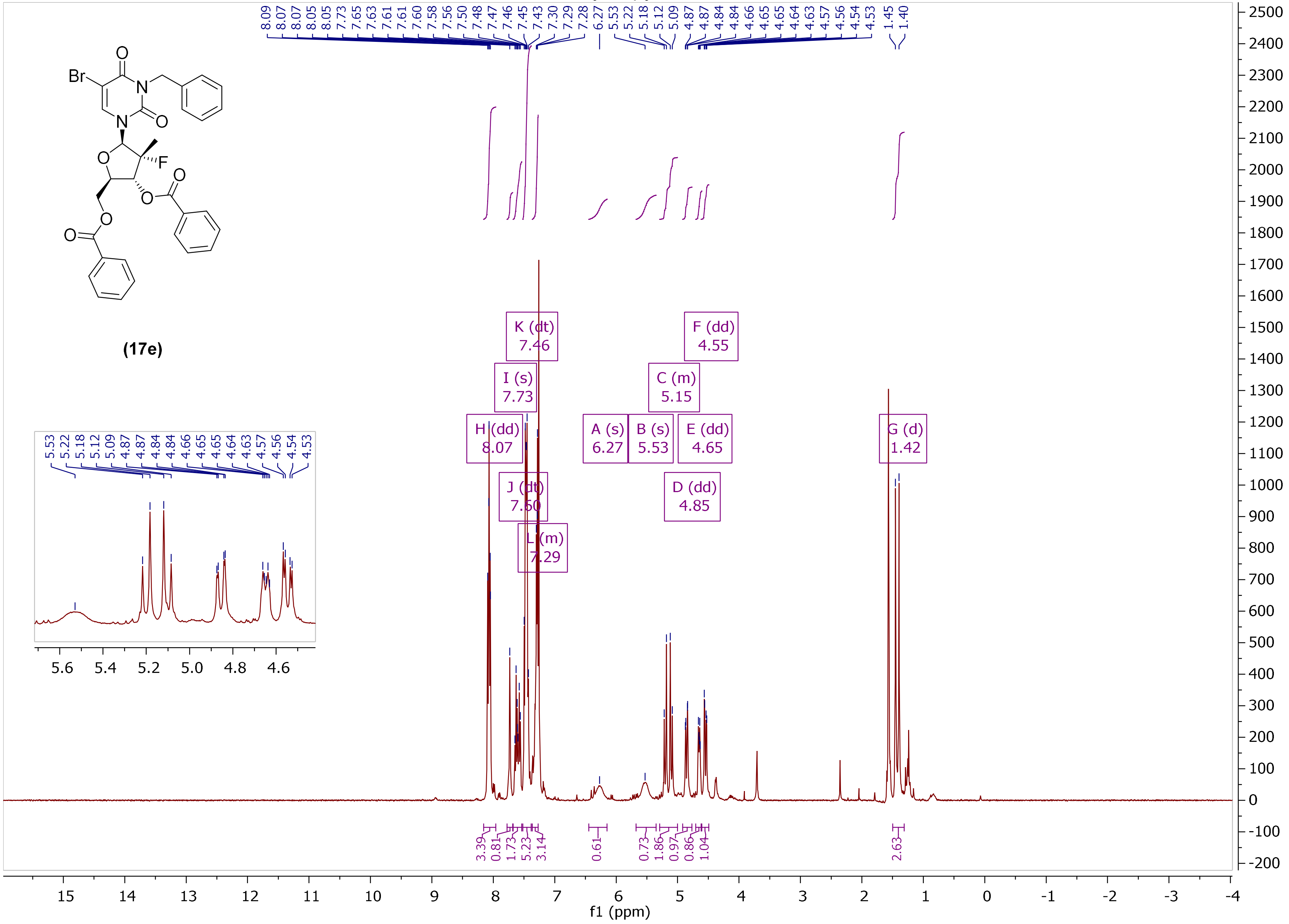

Figure S. 117 - ^{19}F NMR Spectra (377 MHz, $CDCl_3$) - 1-(3,5-Di-*O*-benzoyl-2-deoxy-2-fluoro-2-methyl-β-*D*-ribofuranosyl)-3-benzyl-5-bromo-2,4-dioxo-1,2,3,4-tetrahydropyrimidine – **17e**

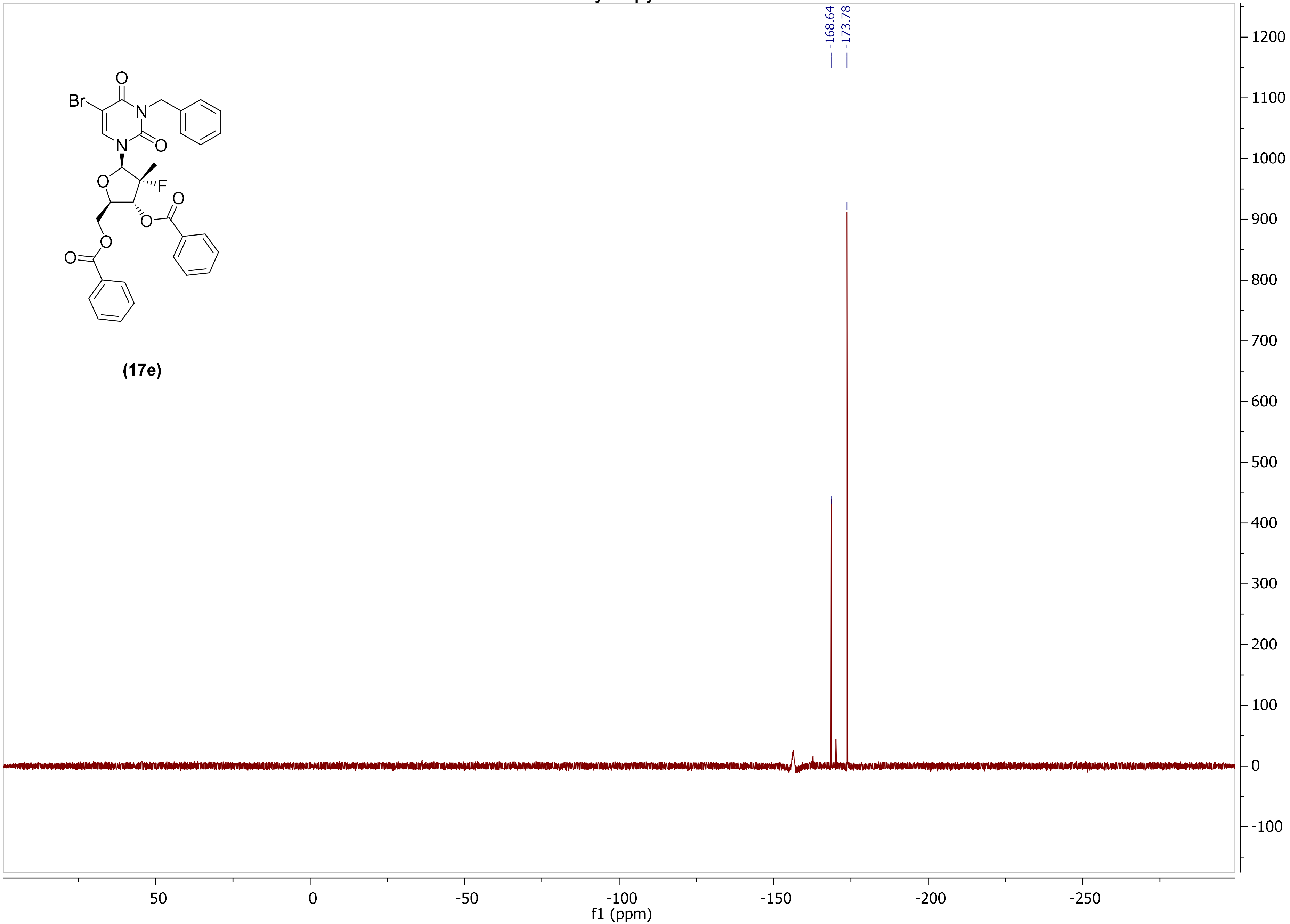

Figure S. 118 - ^{13}C NMR Spectra (101 MHz, $CDCl_3$) - 1-(3,5-Di-*O*-benzoyl-2-deoxy-2-fluoro-2-methyl-β-*D*-ribofuranosyl)-3-benzyl-5-bromo-2,4-dioxo-1,2,3,4-tetrahydropyrimidine – **17e**

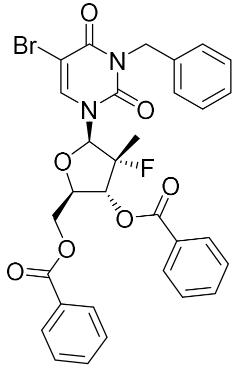

(17e)

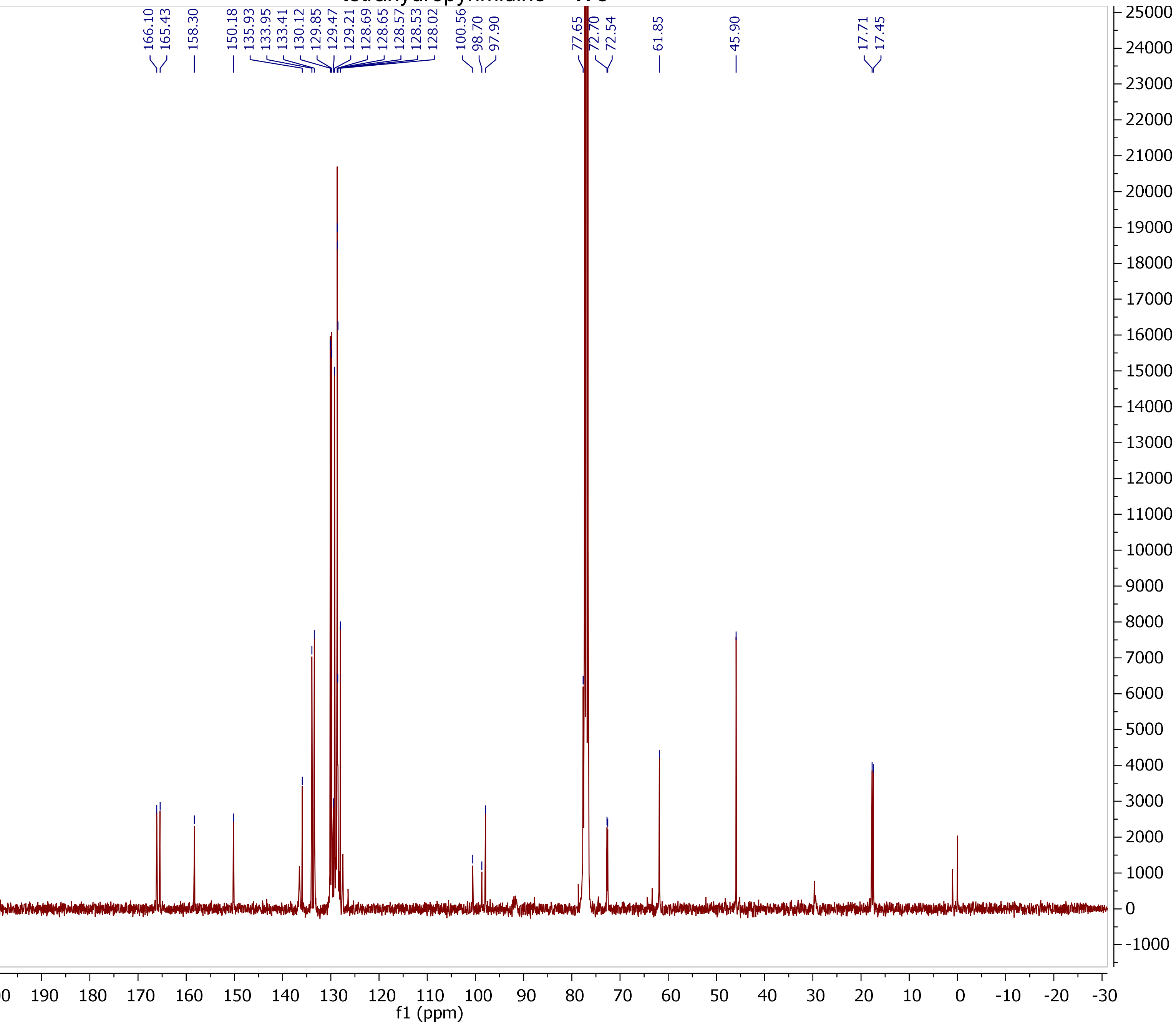

Figure S. 119 - ^{1}H-NMR Spectrum (400 MHz, $CDCl_3$) - 1-(3,5-Di-*O*-benzoyl-2-deoxy-2,2-difluoro-β-*D*-ribofuranosyl)-3-benzyl-5-bromo-2,4-dioxo-1,2,3,4-tetrahydropyrimidine - **17f**

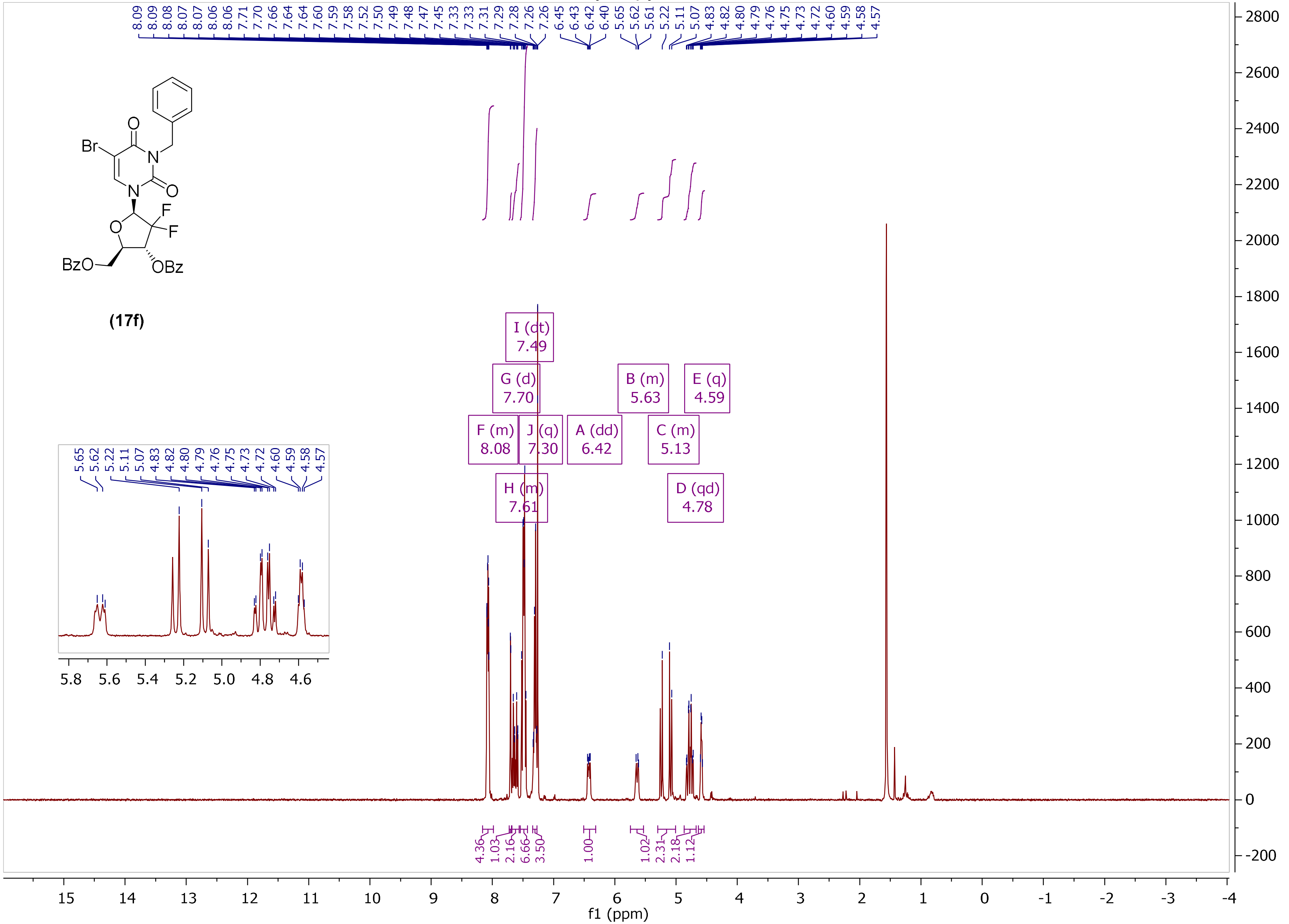

Figure S. 120 - ^{19}F NMR Spectra (377 MHz, $CDCl_3$) - 1-(3,5-Di-*O*-benzoyl-2-deoxy-2,2-difluoro-β-*D*-ribofuranosyl)-3-benzyl-5-bromo-2,4-dioxo-1,2,3,4-tetrahydropyrimidine - **17f**

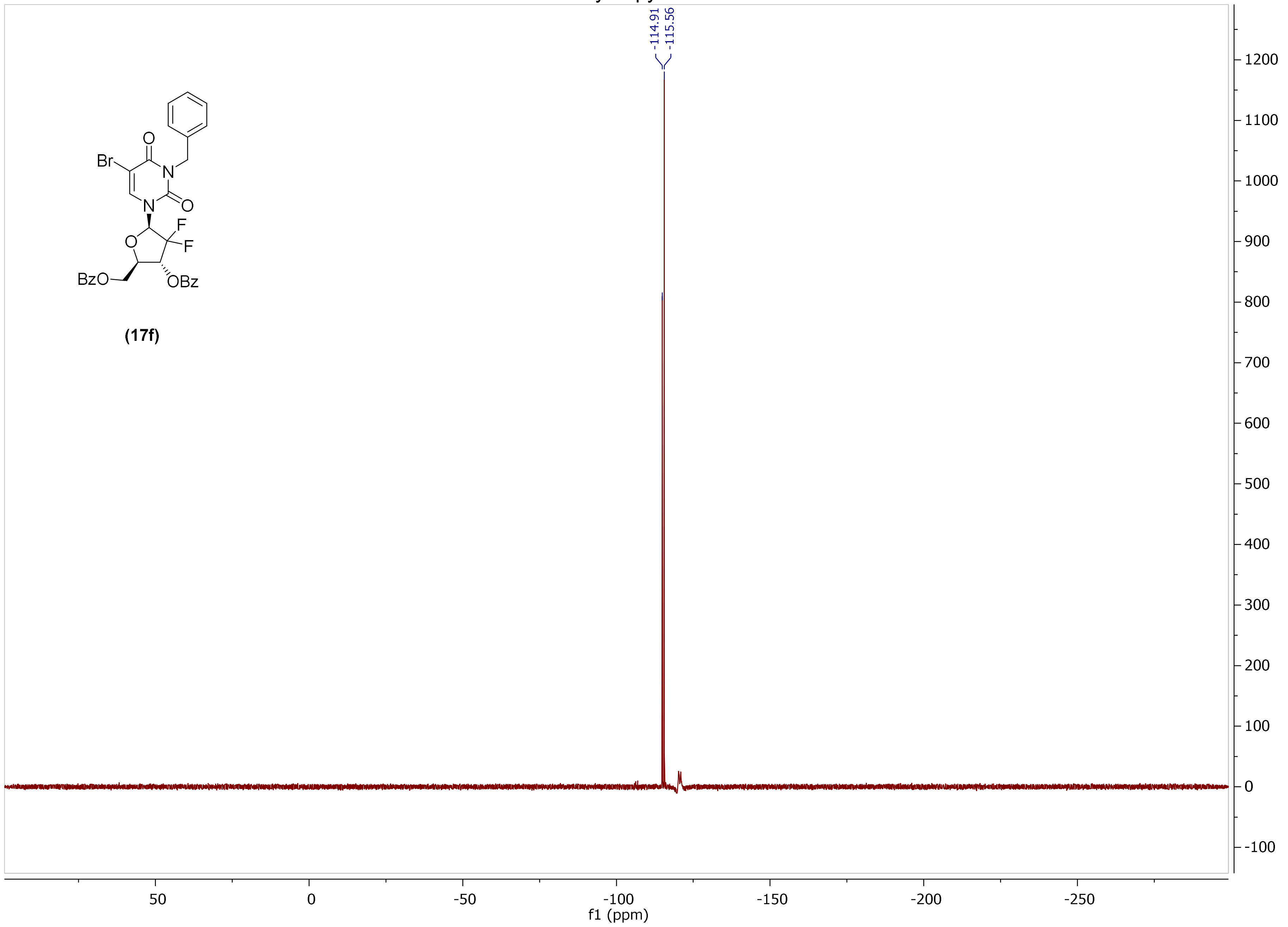

Figure S. 121 - ^{13}C NMR Spectra (101 MHz, $CDCl_3$) - 1-(3,5-Di-*O*-benzoyl-2-deoxy-2,2-difluoro-β-*D*-ribofuranosyl)-3-benzyl-5-bromo-2,4-dioxo-1,2,3,4-tetrahydropyrimidine – **17f**

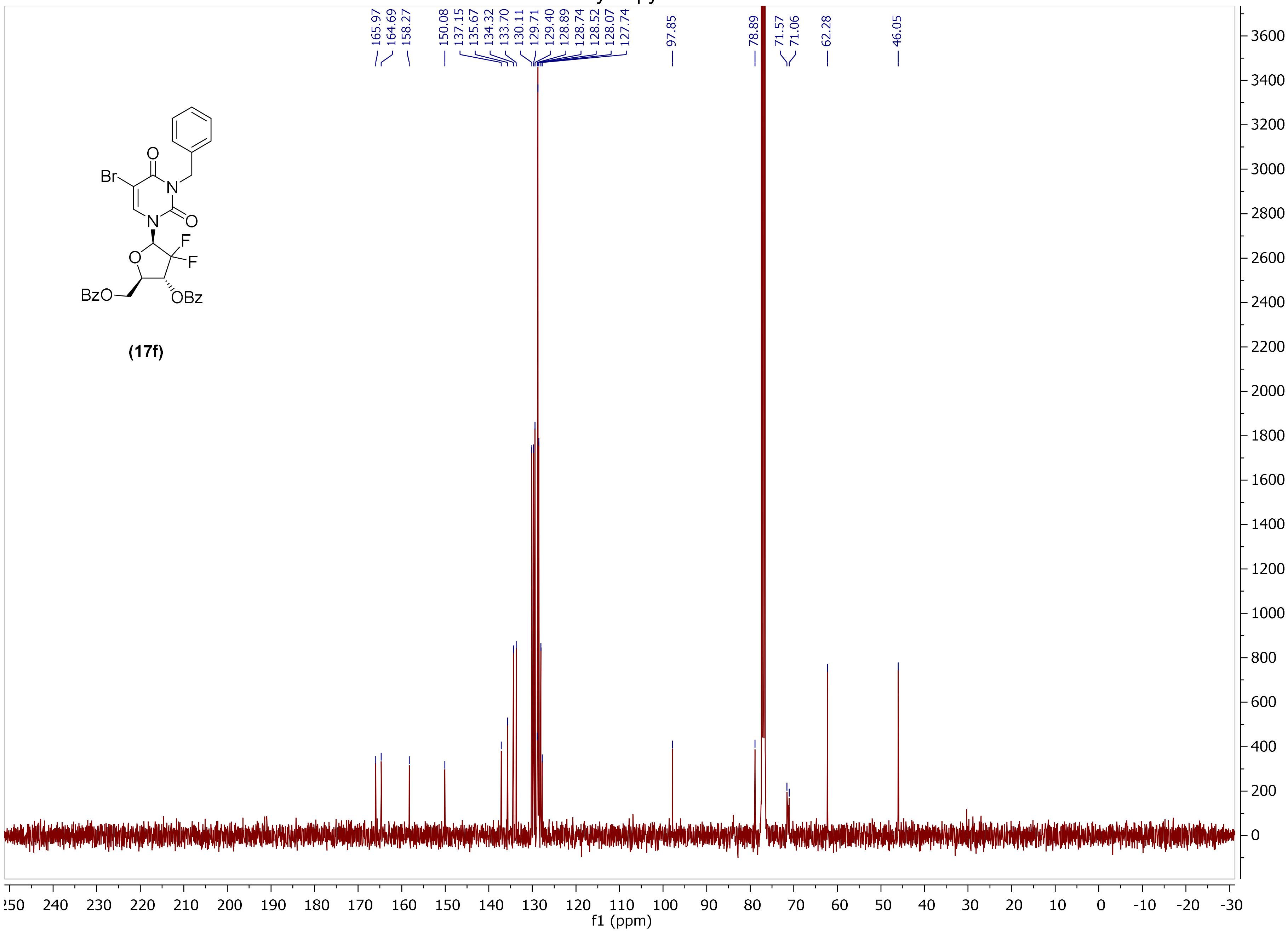

Figure S. 122 - ^{1}H-NMR Spectrum (400 MHz, $CDCl_3$) - 1-(3,5-Di-*O*-(4-methylbenzoyl)-2-deoxy-β-*D*-ribofuranosyl)-3-(2,4-dimethoxybenzyl)-2,4-dioxo-1,2,3,4-tetrahydropyrimidine-5-carboxylic acid - **18a**

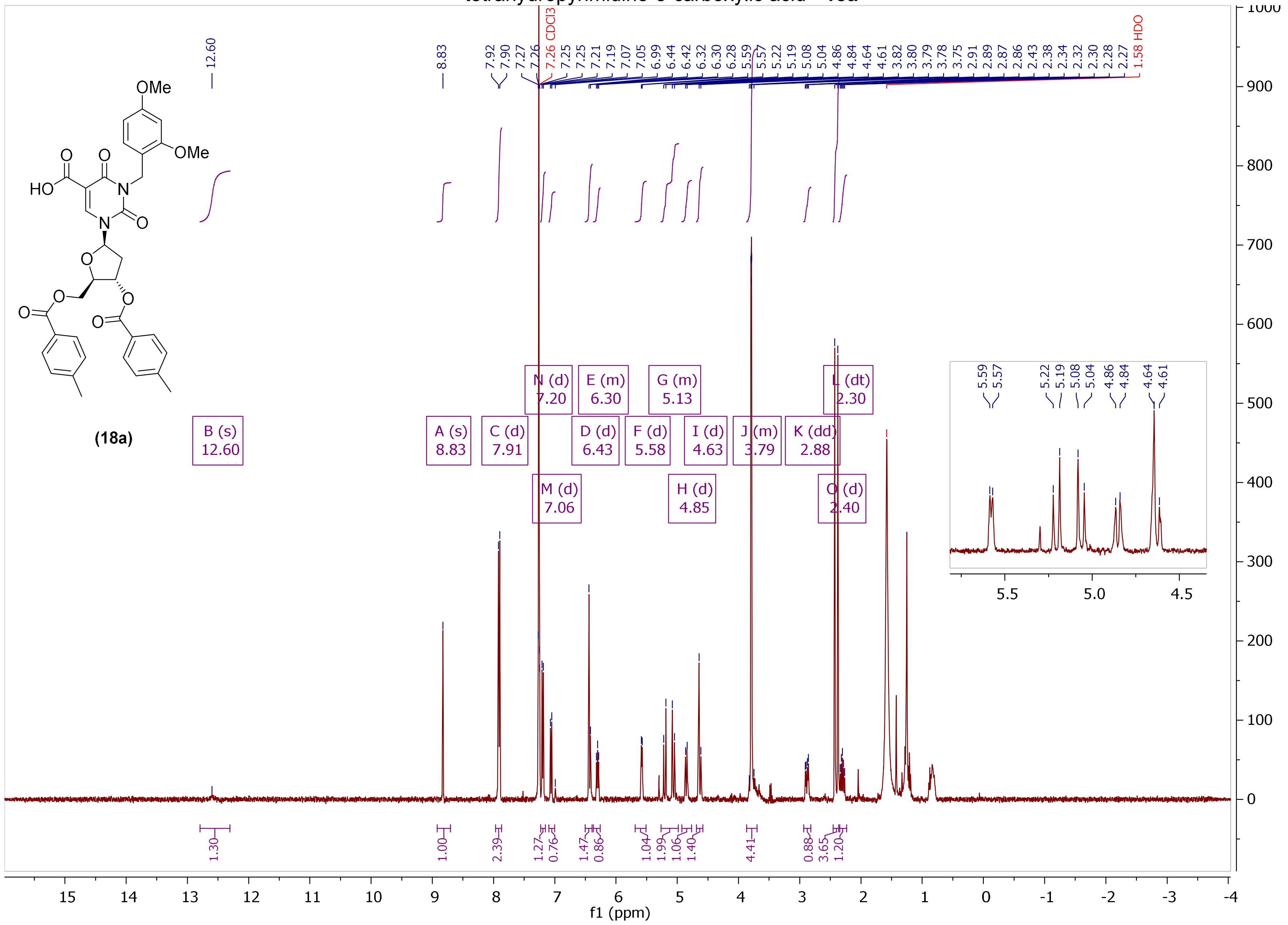

Figure S. 123 - ^{13}C NMR Spectra (101 MHz, $CDCl_3$) -1 -(3,5-Di-*O*-(4-methylbenzoyl)-2-deoxy-β-*D*-ribofuranosyl)-3-(2,4-dimethoxybenzyl)-2,4-dioxo-1,2,3,4-tetrahydropyrimidine-5-carboxylic acid – **18a**

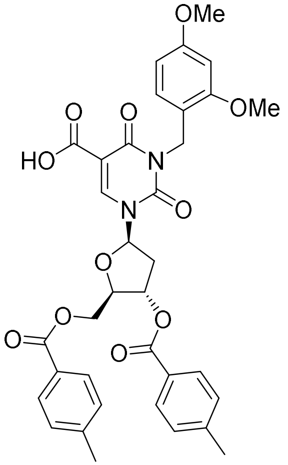

(18a)

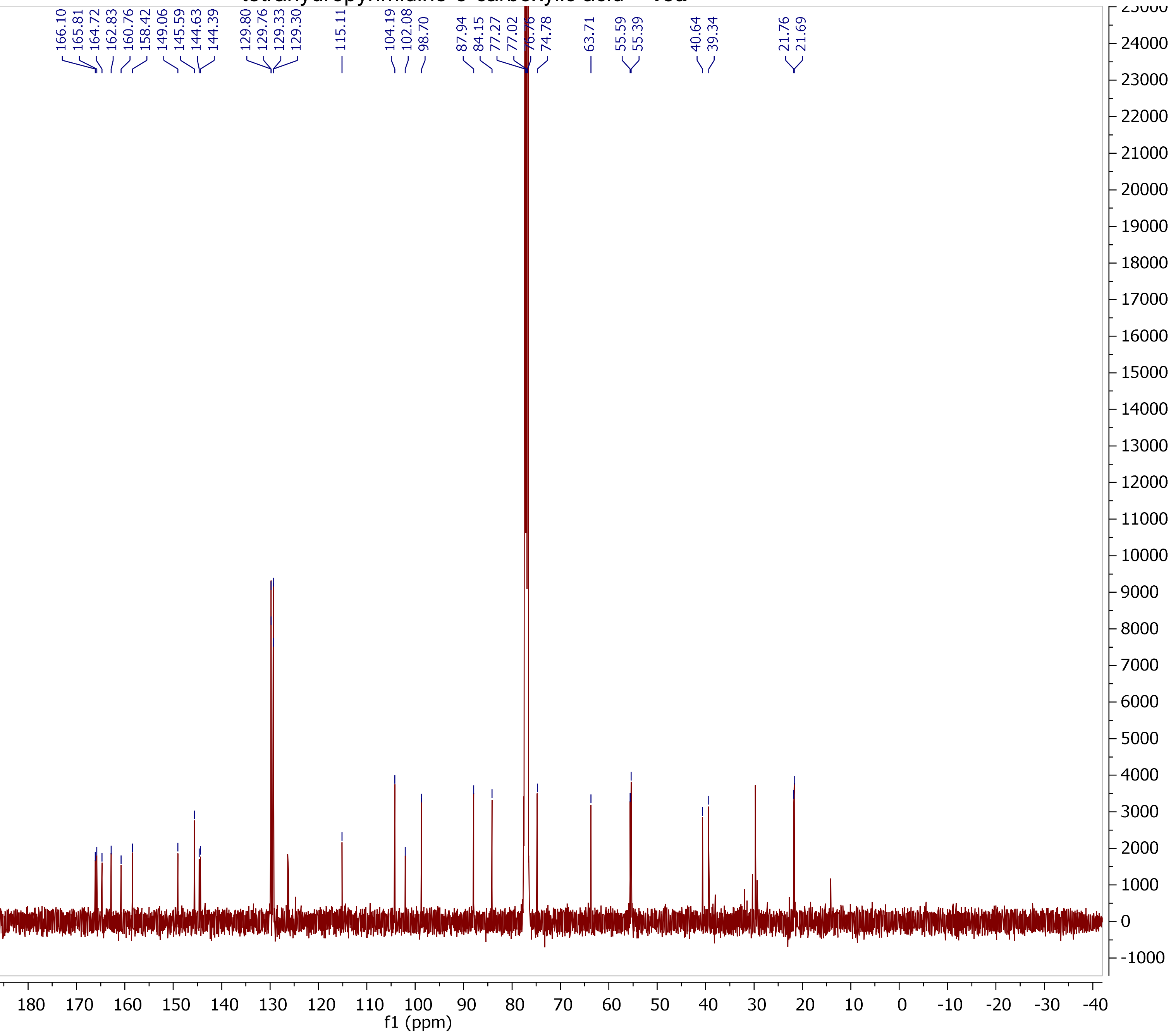

Figure S. 124 - ^{1}H-NMR Spectrum (400 MHz, D_2O) - 1-(2-Deoxy-β-*D*-ribofuranosyl)-2,4-dioxo-1,2,3,4-tetrahydropyrimidine-5-carboxylic acid – **19a**

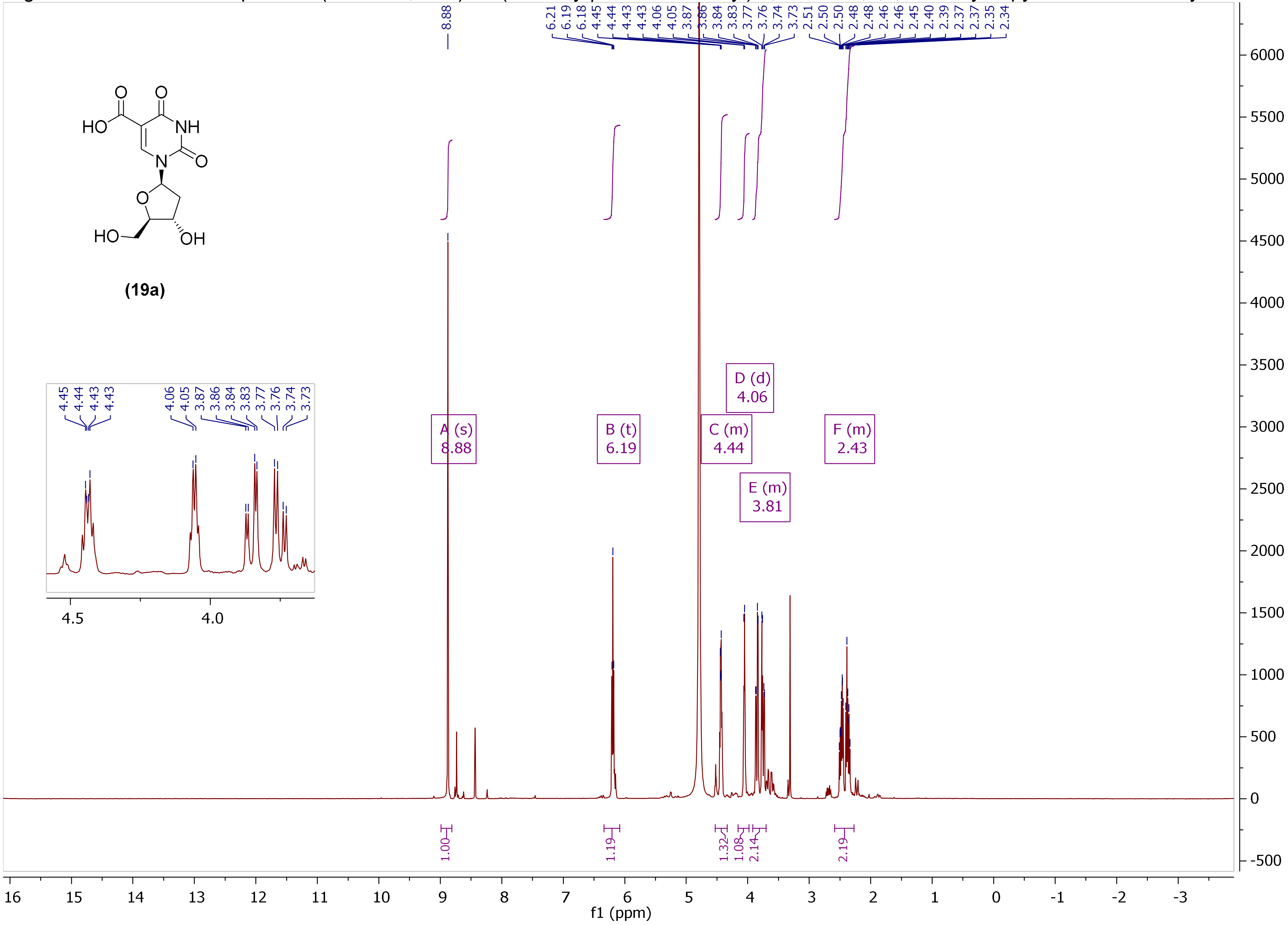

Figure S. 125 - ^{13}C NMR Spectra (101 MHz, D_2O) - 1-(2-Deoxy-β-*D*-ribofuranosyl)-2,4-dioxo-1,2,3,4-tetrahydropyrimidine-5-carboxylic acid – **19a**

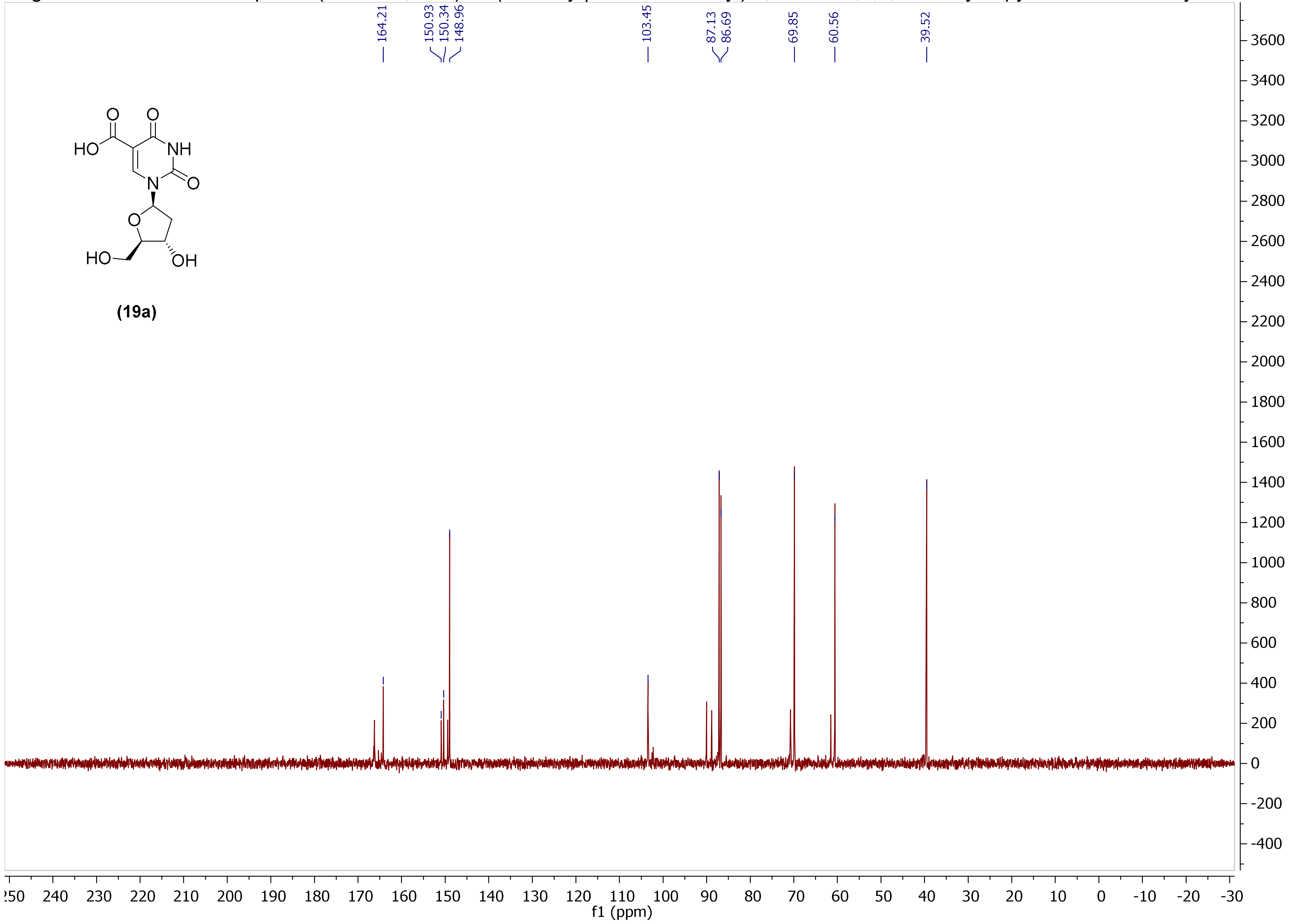

Figure S. 126 - ^{1}H-NMR Spectrum (400 MHz, $CDCl_3$) - 1-(2,3,5-Tri-*O*-acetyl-β-*D*-ribofuranosyl)-3-(2,4-dimethoxybenzyl)-2,4-dioxo-1,2,3,4-tetrahydropyrimidine-5-carboxylic acid – **18b**

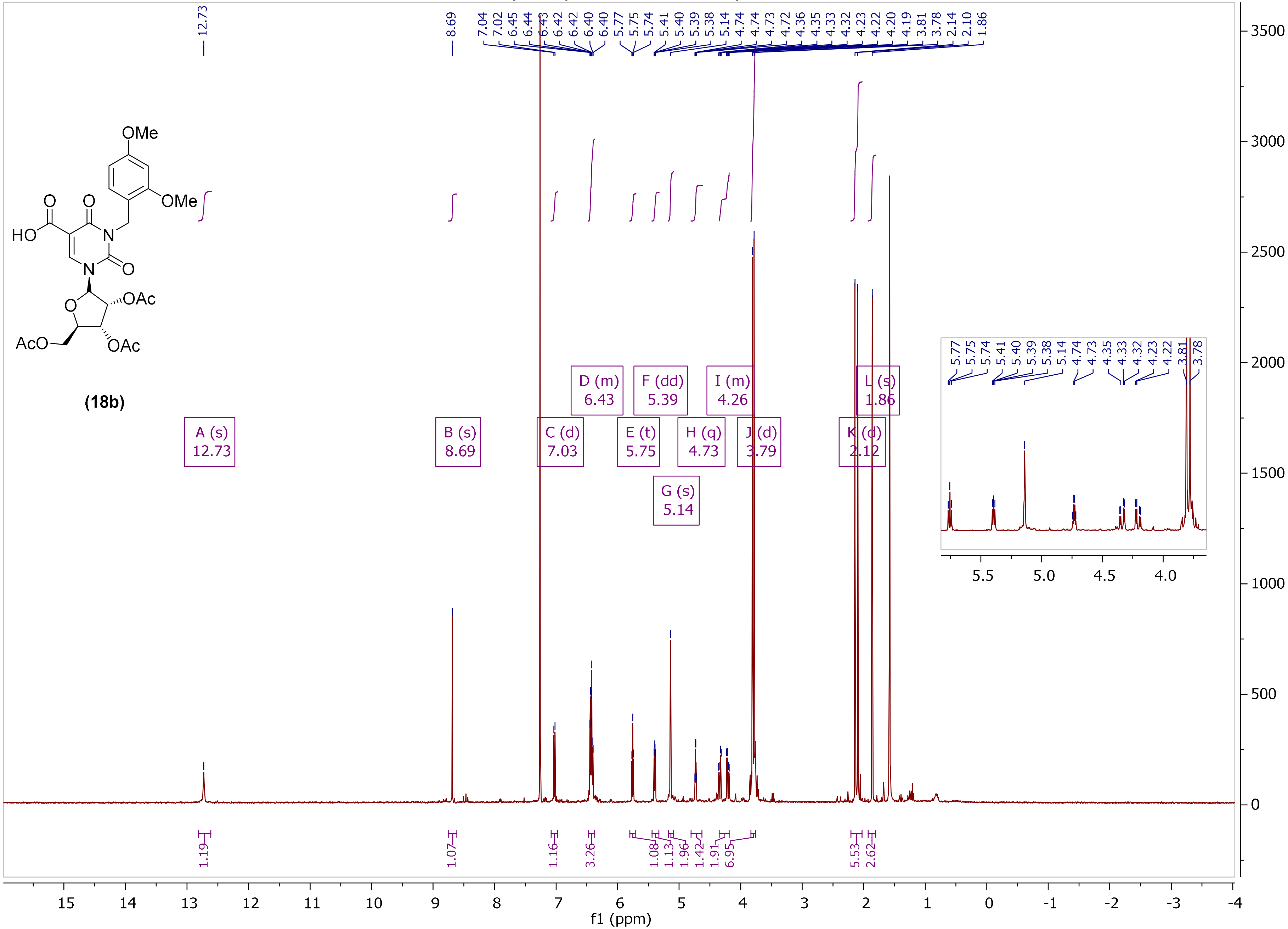

Figure S. 127 - ^{13}C NMR Spectra (101 MHz, $CDCl_3$) - 1-(2,3,5-Tri-*O*-acetyl-β-*D*-ribofuranosyl)-3-(2,4-dimethoxybenzyl)-2,4-dioxo-1,2,3,4-tetrahydropyrimidine-5-carboxylic acid - **18b**

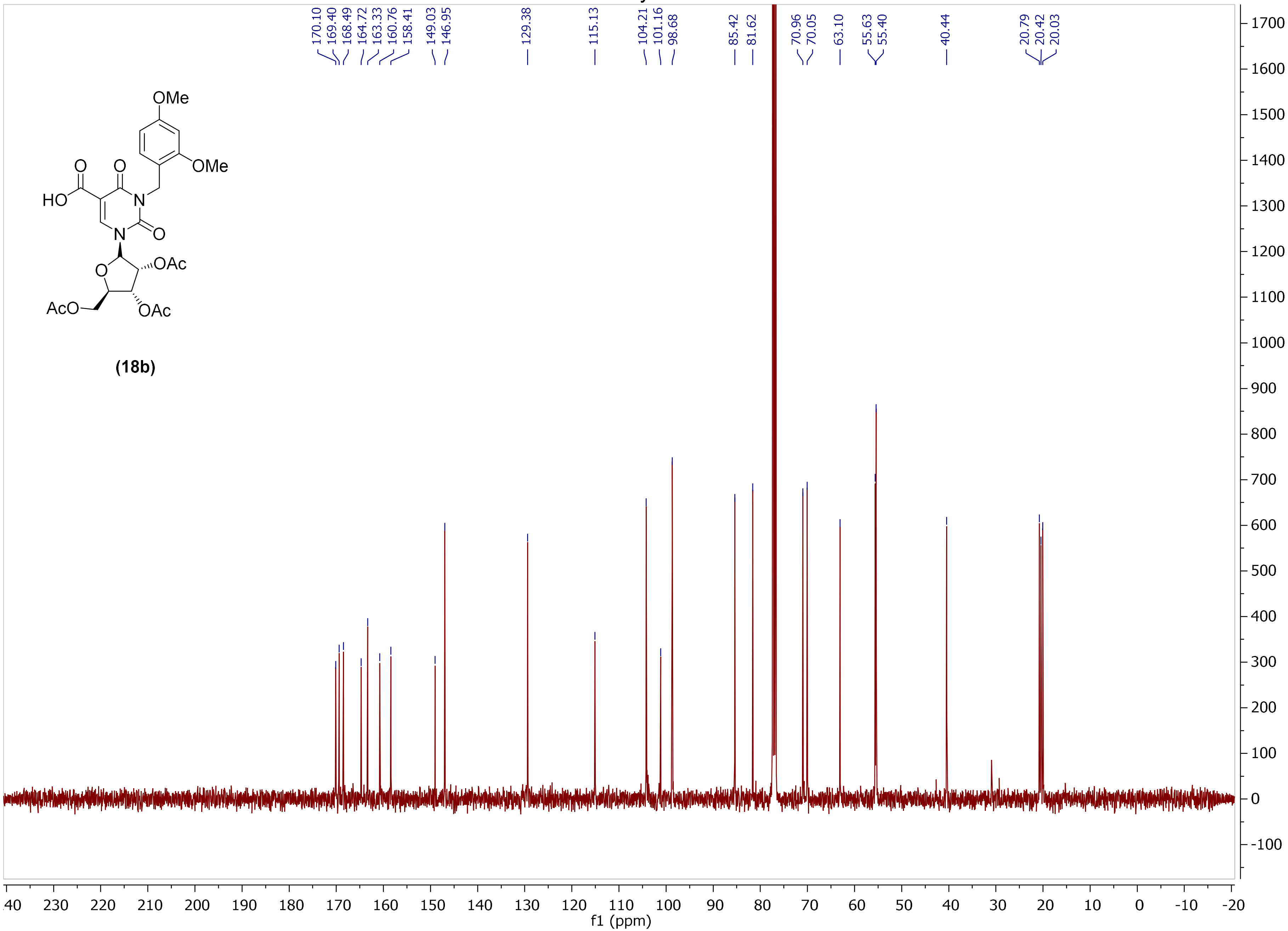

Figure S. 128 - ^{1}H-NMR Spectrum (400 MHz, D_2O) -1-(β-*D*-Ribofuranosyl)-2,4-dioxo-1,2,3,4-tetrahydropyrimidine-5-carboxylic acid – **19b**

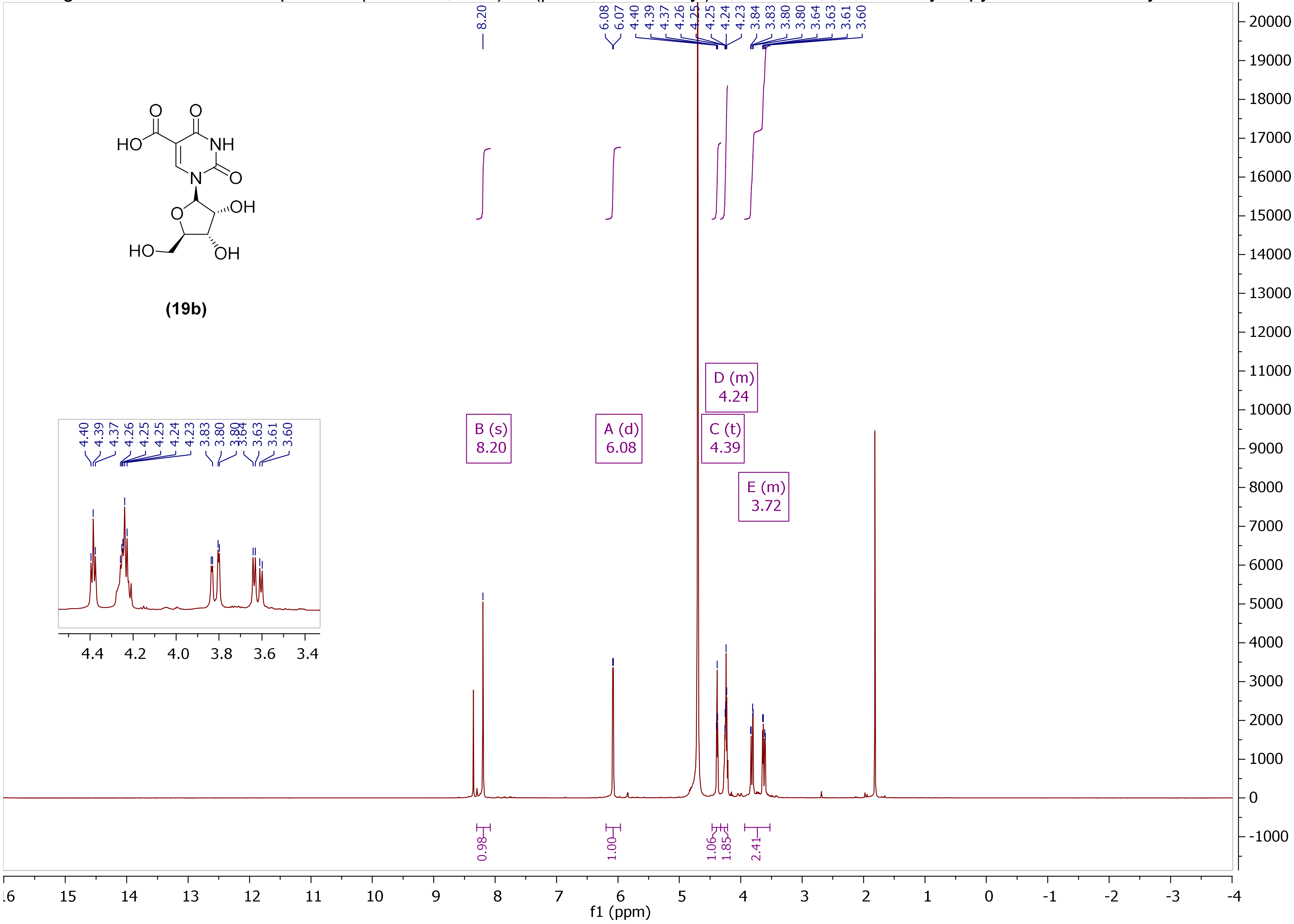

Figure S. 129 - ^{13}C NMR Spectra (101 MHz, D_2O) - 1-(β-*D*-Ribofuranosyl)-2,4-dioxo-1,2,3,4-tetrahydropyrimidine-5-carboxylic acid – **19b**

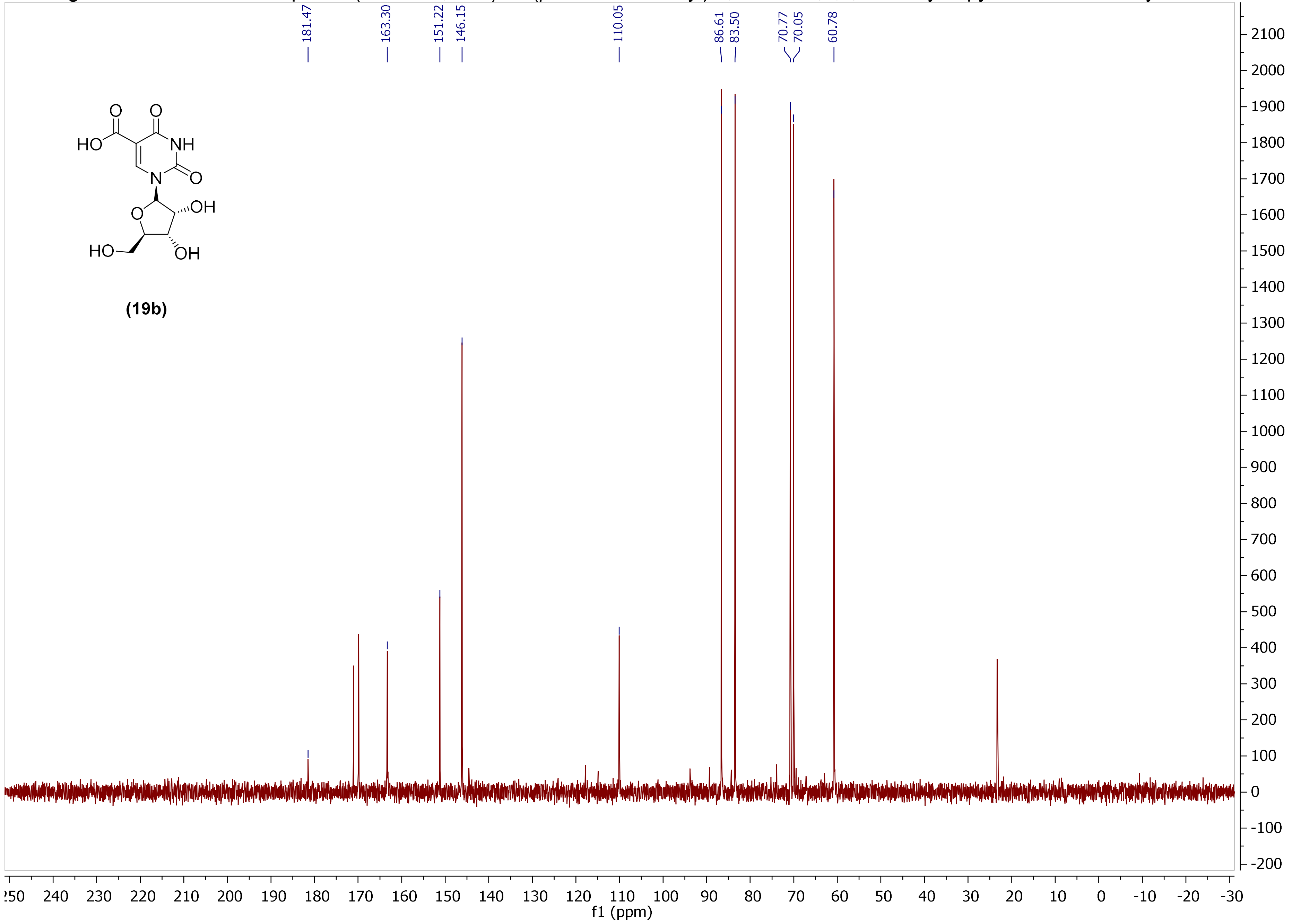

Figure S. 130 - ^{1}H-NMR Spectrum (400 MHz, $CDCl_3$) - 1-(3,5-Di-*O*-benzoyl-2-deoxy-2-fluoro-β-*D*-arabinofuranosyl)-3-(2,4-dimethoxybenzyl)-2,4-dioxo-1,2,3,4-tetrahydropyrimidine-5-carboxylic acid – **18c**

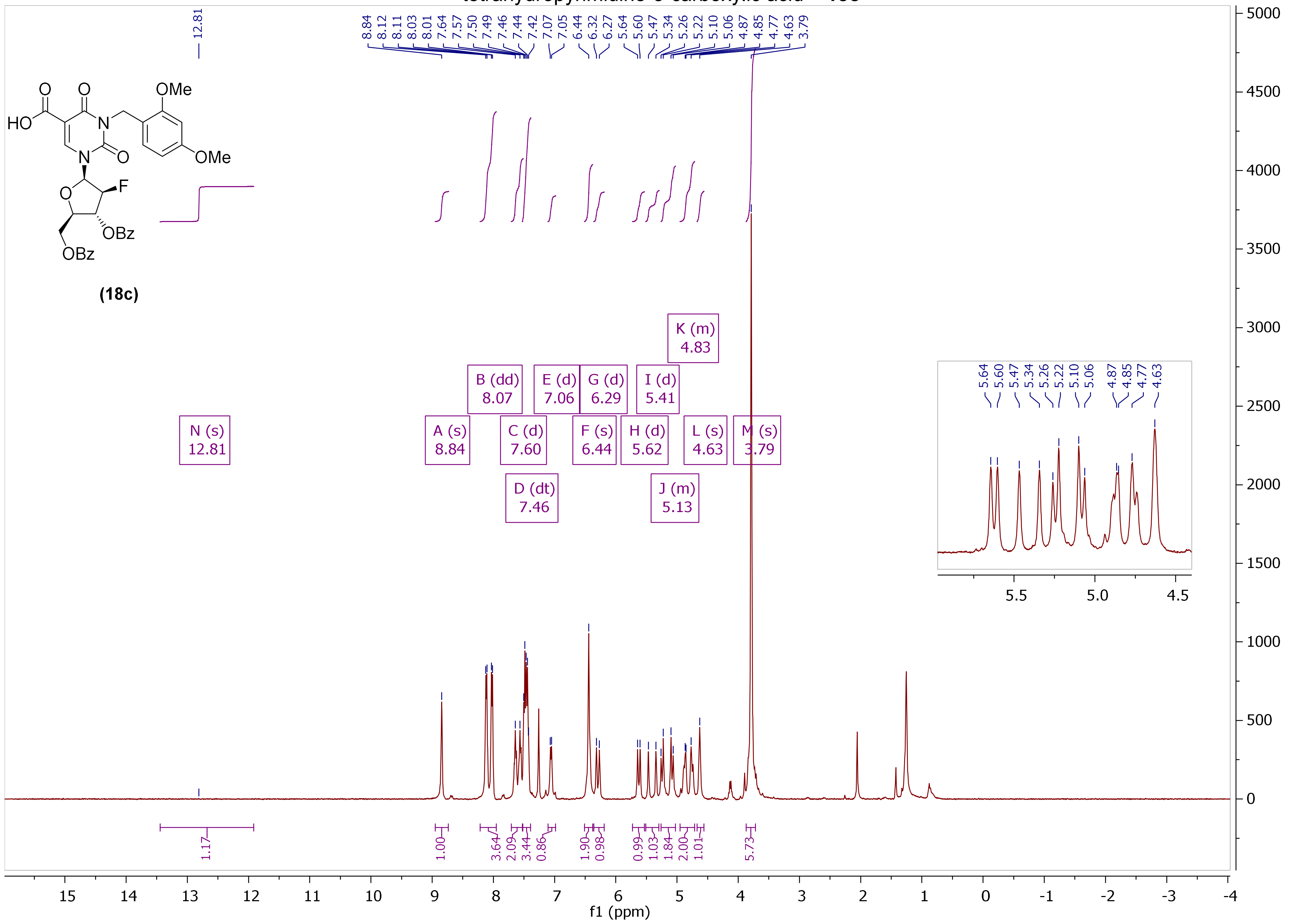

Figure S. 131 - ^{19}F NMR Spectra (377 MHz, $CDCl_3$) - 1-(3,5-Di-*O*-benzoyl-2-deoxy-2-fluoro-β-*D*-arabinofuranosyl)-3-(2,4-dimethoxybenzyl)-2,4-dioxo-1,2,3,4-tetrahydropyrimidine-5-carboxylic acid - **18c**

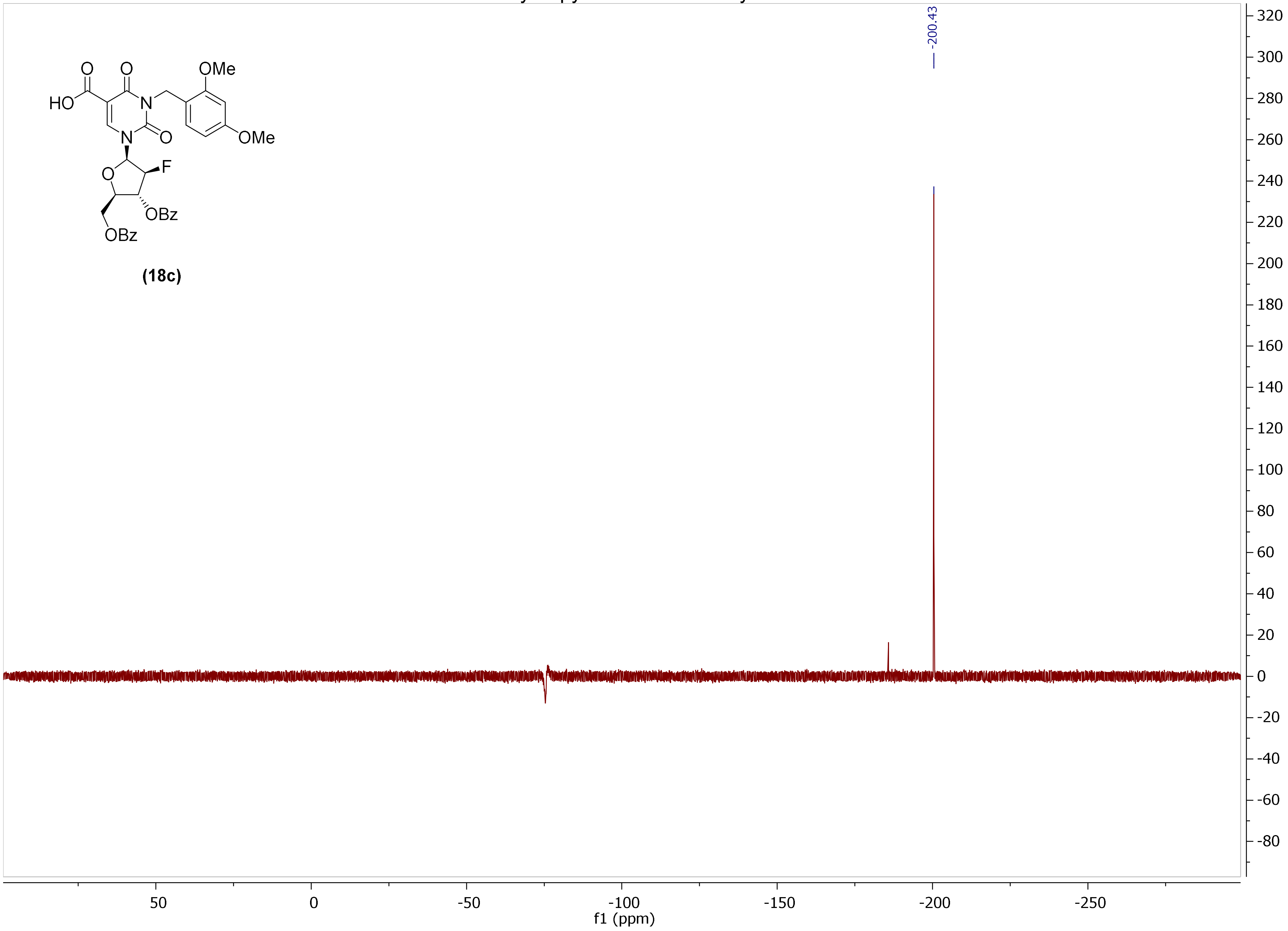

Figure S. 132 - ^{13}C NMR Spectra (101 MHz, $CDCl_3$) - 1-(3,5-Di-*O*-benzoyl-2-deoxy-2-fluoro-β-*D*-arabinofuranosyl)-3-(2,4-dimethoxybenzyl)-2,4-dioxo-1,2,3,4-tetrahydropyrimidine-5-carboxylic acid – **18c**

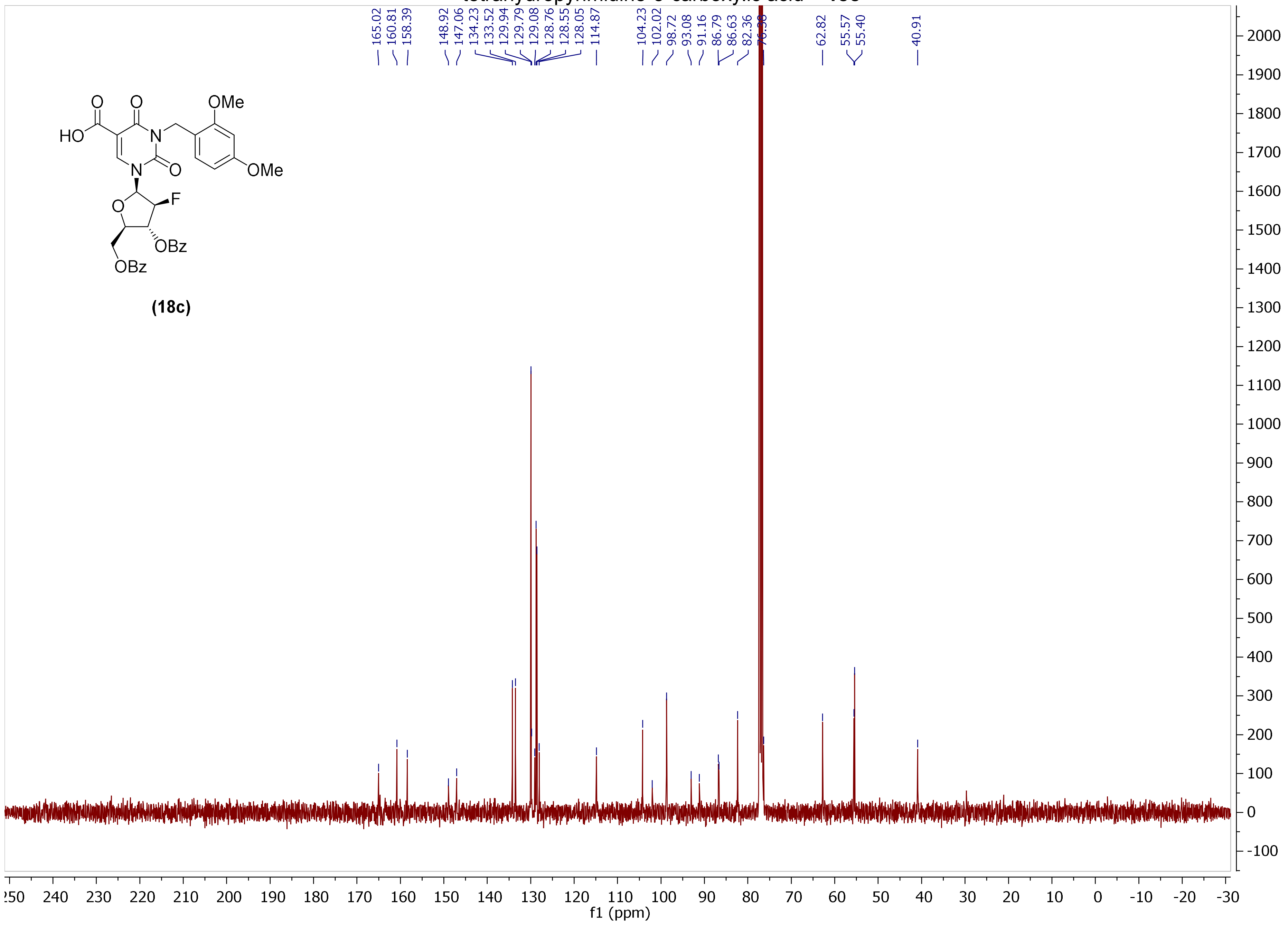

Figure S. 133 - ^{1}H-NMR Spectrum (400 MHz, D_2O) - 1-(2-Deoxy-2-fluoro-β-*D*-arabinofuranosyl)-2,4-dioxo-1,2,3,4-tetrahydropyrimidine-5-carboxylic acid – **19c**

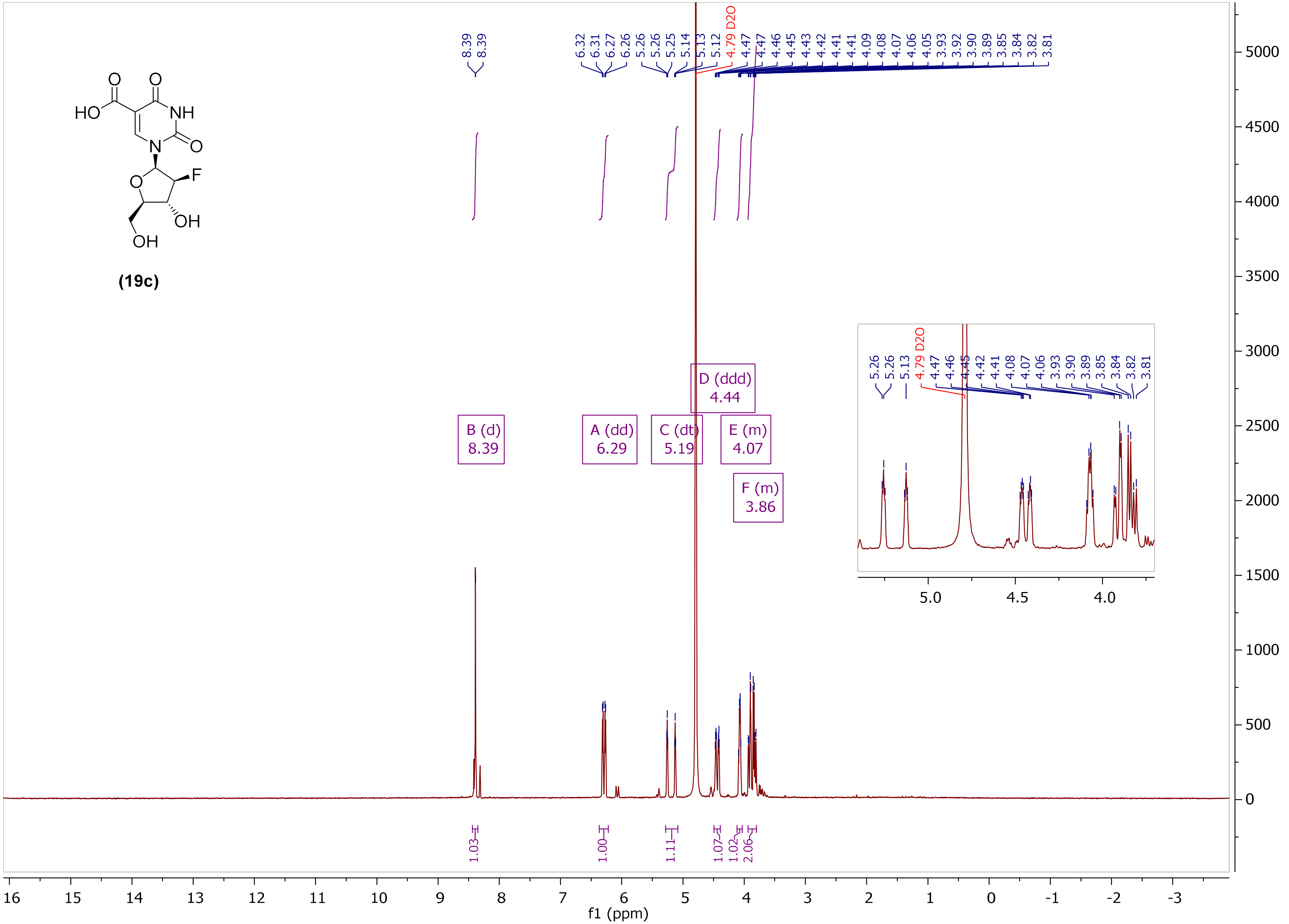

Figure S. 134 - ^{13}C NMR Spectra (101 MHz, D_2O) - 1-(2-Deoxy-2-fluoro-β-*D*-arabinofuranosyl)-2,4-dioxo-1,2,3,4-tetrahydropyrimidine-5-carboxylic acid – **19c**

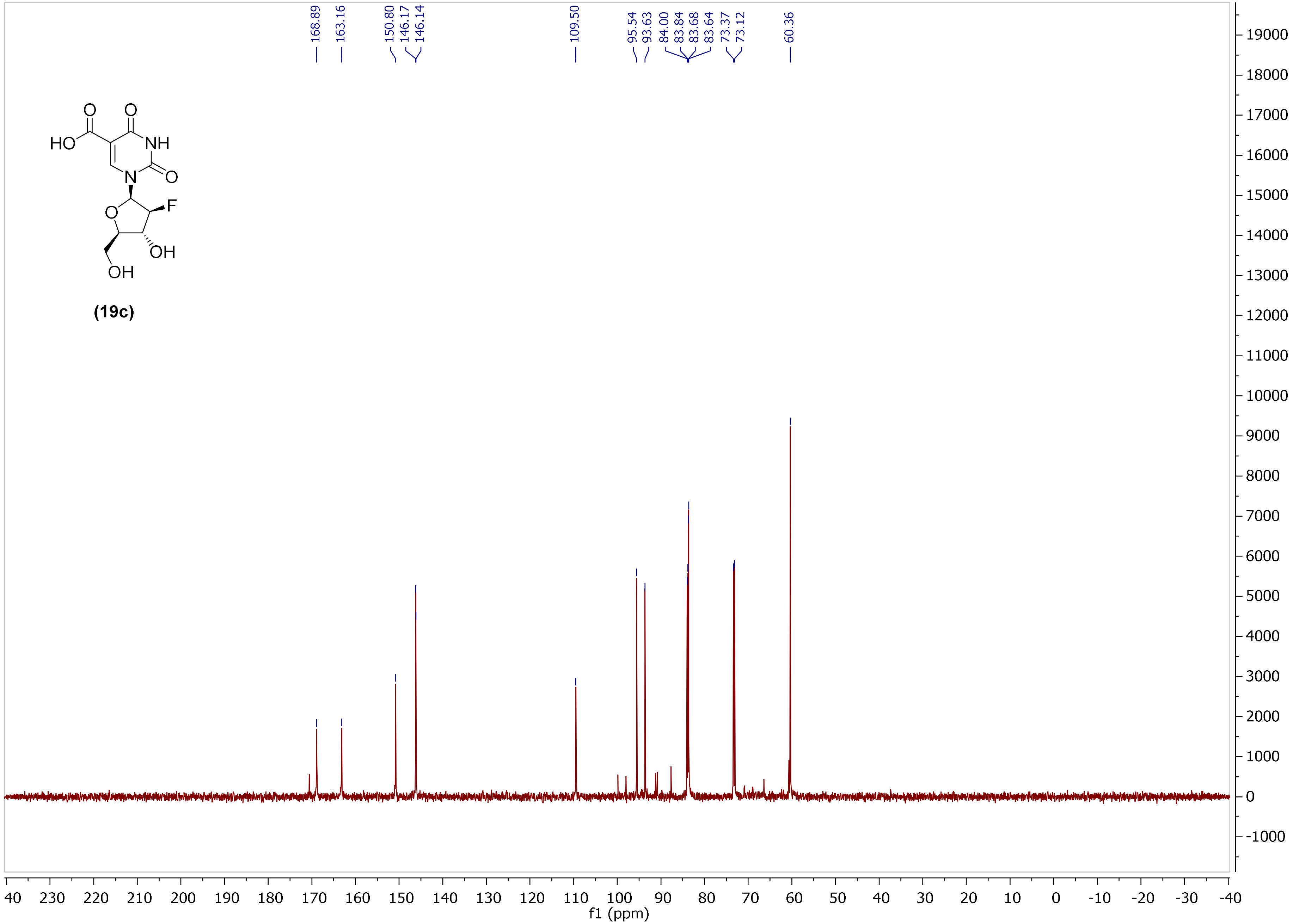

Figure S. 135 - ^{1}H-NMR Spectrum (400 MHz, $CDCl_3$) - 1-(3,5-Di-*O*-benzoyl-2-deoxy-2-fluoro-2-methyl-β-*D*-ribofuranosyl)-3-(2,4-dimethoxybenzyl)-2,4-dioxo-1,2,3,4-tetrahydropyrimidine-5-carboxylic acid – **18d**

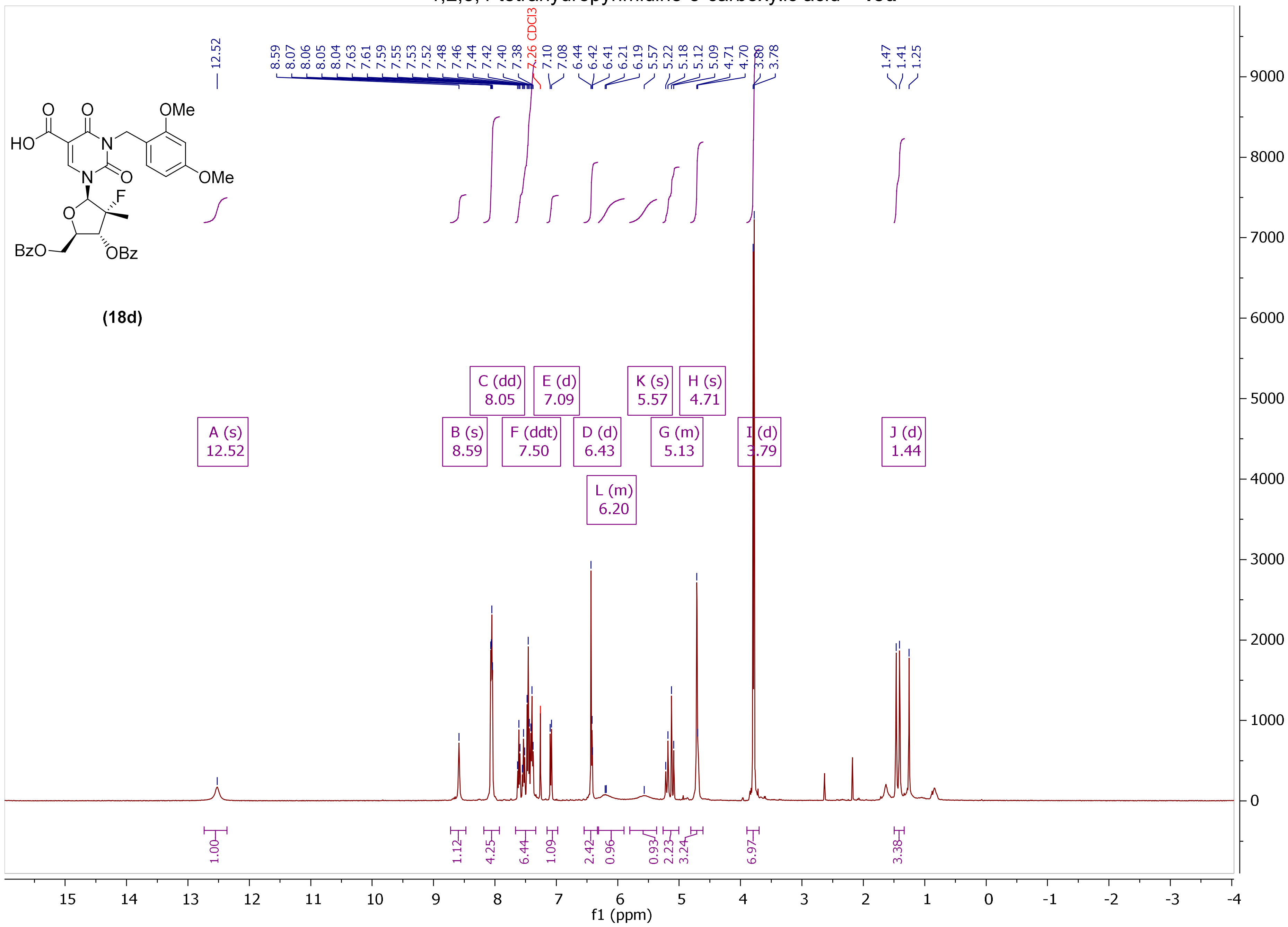

Figure S. 136 - ^{13}C NMR Spectra (101 MHz, $CDCl_3$) - 1-(3,5-Di-*O*-benzoyl-2-deoxy-2-fluoro-2-methyl-β-*D*-ribofuranosyl)-3-(2,4-dimethoxybenzyl)-2,4-dioxo-1,2,3,4-tetrahydropyrimidine-5-carboxylic acid – **18d**

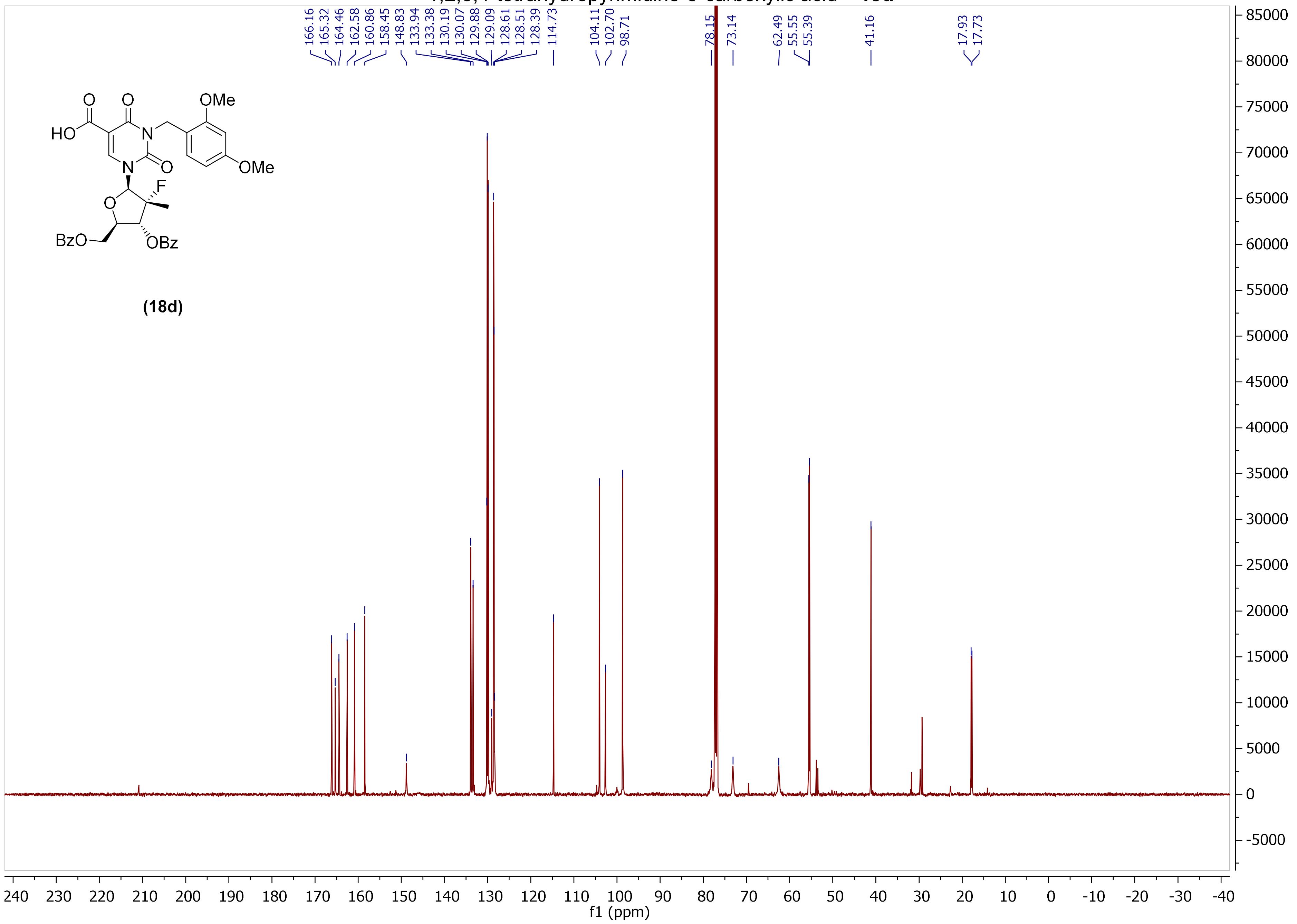

Figure S. 137 - ^{1}H-NMR Spectrum (400 MHz, D_2O) - 1-(2-Deoxy-2-fluoro-2-methyl-β-*D*-ribofuranosyl)-2,4-dioxo-1,2,3,4-tetrahydropyrimidine-5-carboxylic acid – **19d**

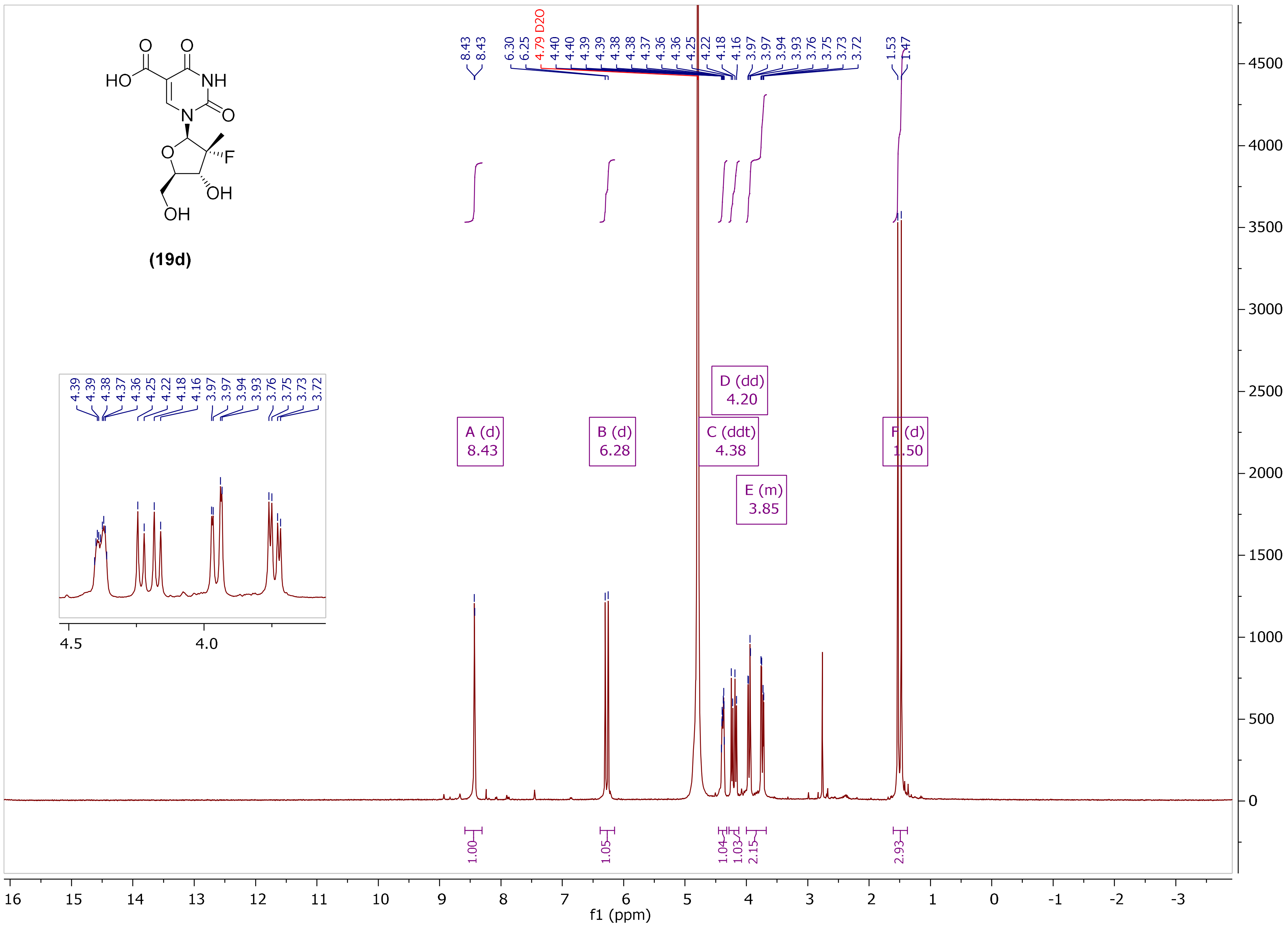

Figure S. 138 -^{1}H-NMR Spectrum (400 MHz, D_2O) - 1-(2-Deoxy-2-fluoro-2-methyl-α-*D*-ribofuranosyl)-2,4-dioxo-1,2,3,4-tetrahydropyrimidine-5-carboxylic acid – (1-epi-**19d**)

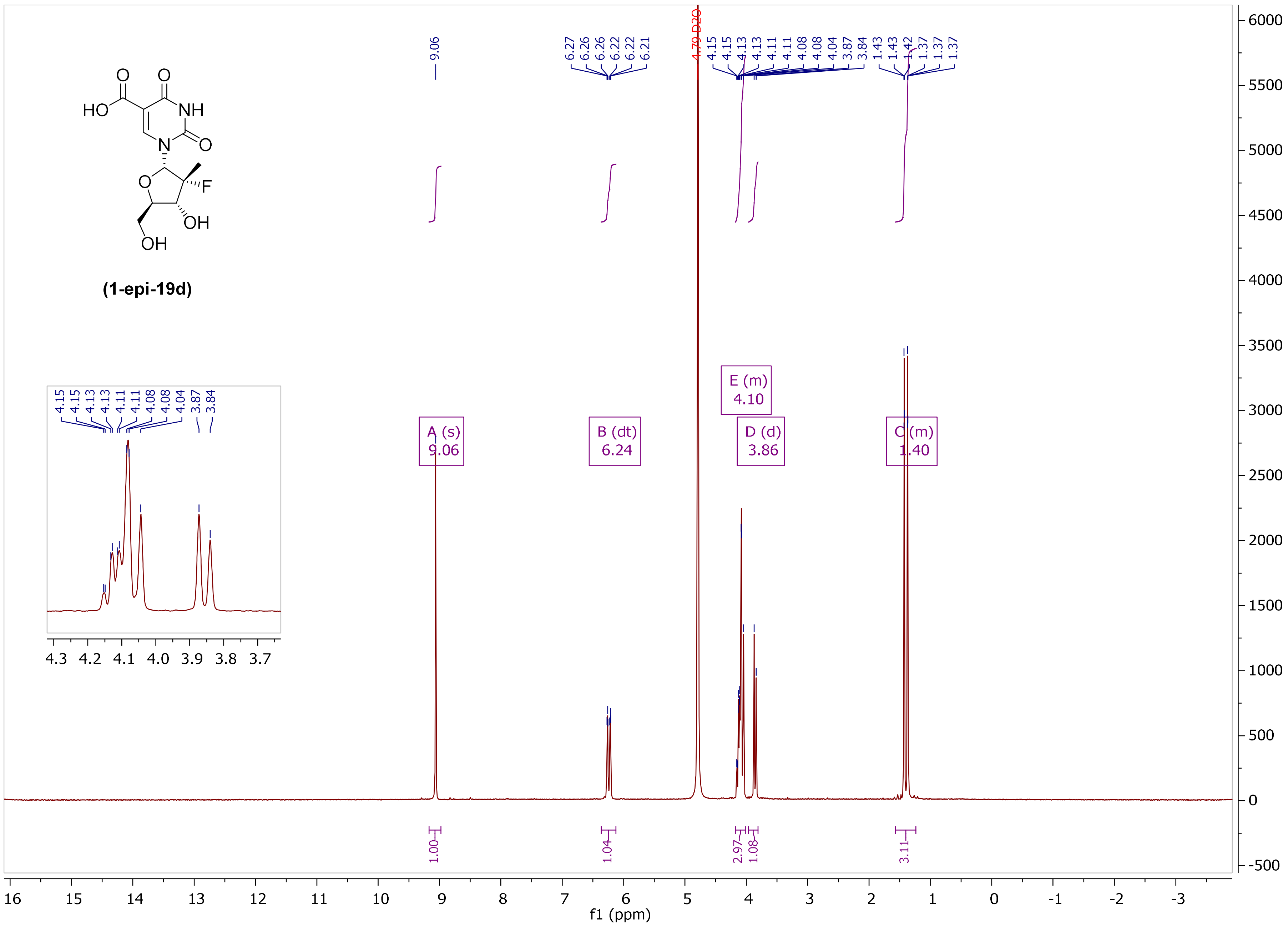

Figure S. 139 - ^{19}F NMR Spectra (377 MHz, D_2O) - 1-(2-Deoxy-2-fluoro-2-methyl-β-*D*-ribofuranosyl)-2,4-dioxo-1,2,3,4-tetrahydropyrimidine-5-carboxylic acid – **19d**

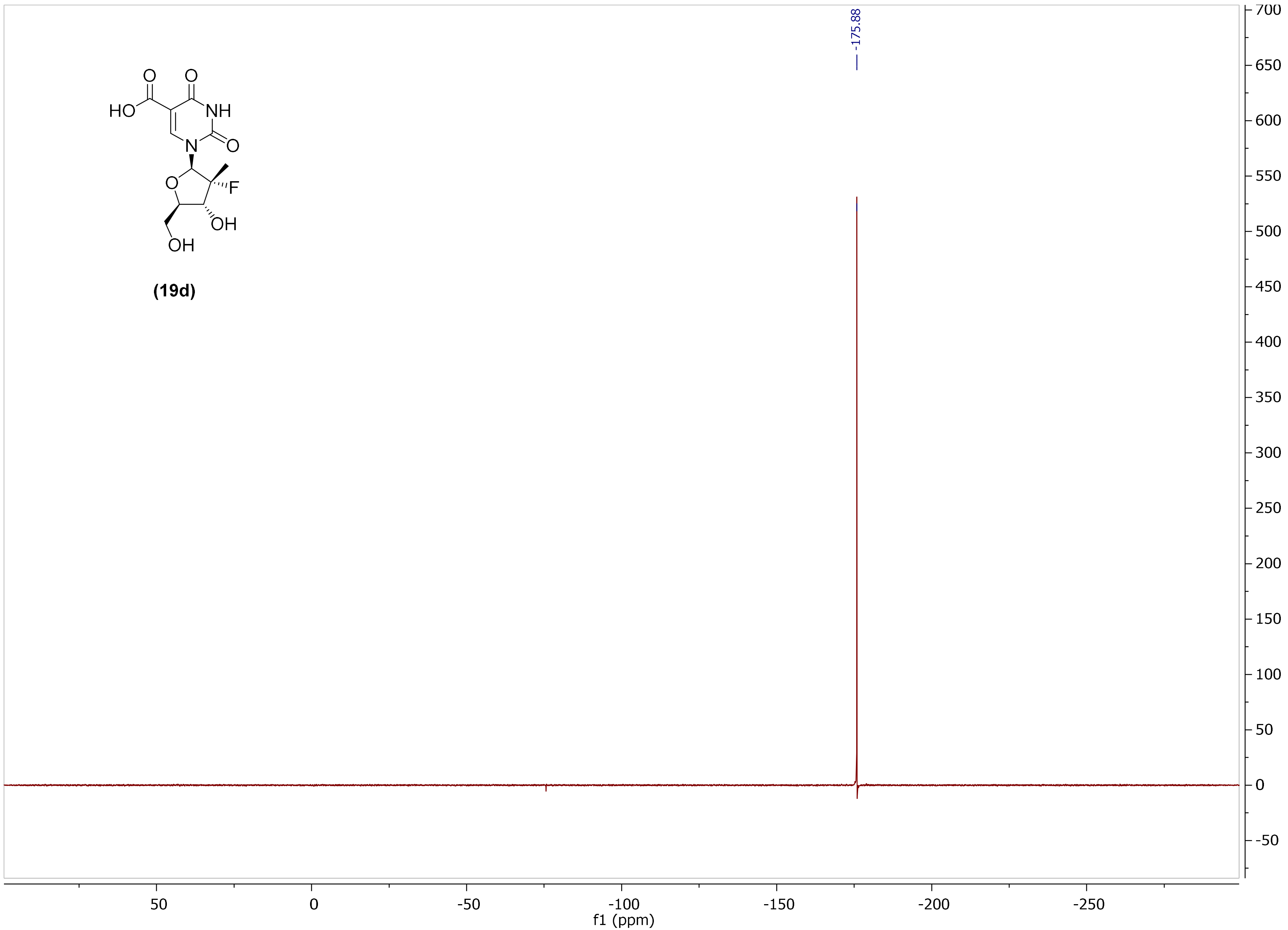

Figure S. 140- ^{19}F NMR Spectra (377 MHz, D_2O) - 1-(2-Deoxy-2-fluoro-2-methyl-α-*D*-ribofuranosyl)-2,4-dioxo-1,2,3,4-tetrahydropyrimidine-5-carboxylic acid – (1-epi-**19d**)

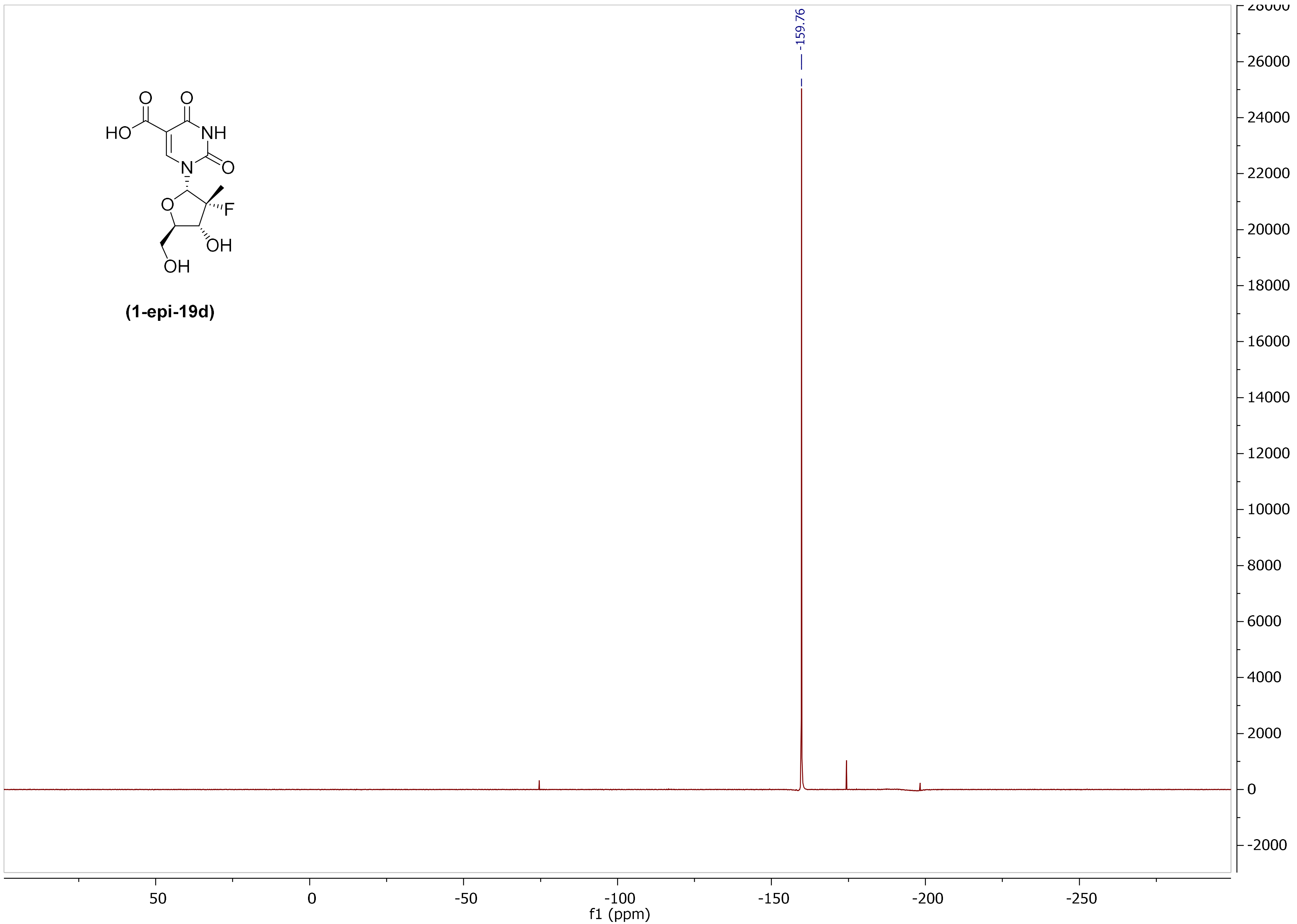

(1-epi-19d)

Figure S. 141.- ^{13}C NMR Spectra (101 MHz, D_2O) - 1-(2-Deoxy-2-fluoro-2-methyl-β-*D*-ribofuranosyl)-2,4-dioxo-1,2,3,4-tetrahydropyrimidine-5-carboxylic acid – **19d**

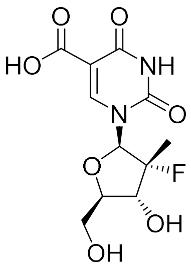

(19d)

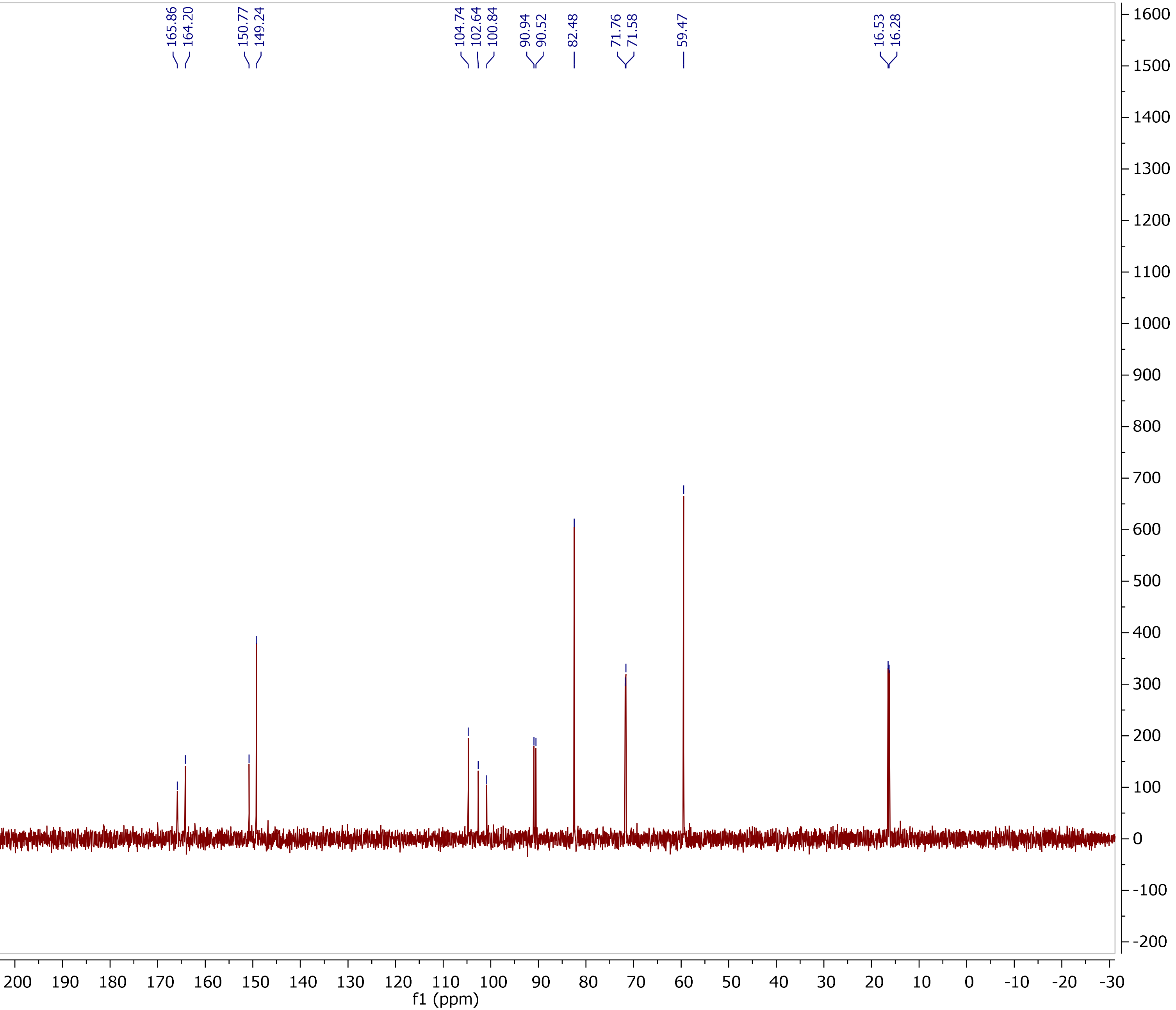

Figure S. 142 - ^{13}C NMR Spectra (101 MHz, D_2O) - 1-(2-Deoxy-2-fluoro-2-methyl-α-*D*-ribofuranosyl)-2,4-dioxo-1,2,3,4-tetrahydropyrimidine-5-carboxylic acid – (1-epi-**19d**)

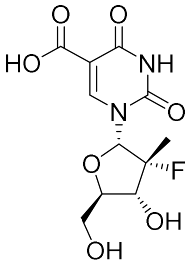

(1-epi-19d)

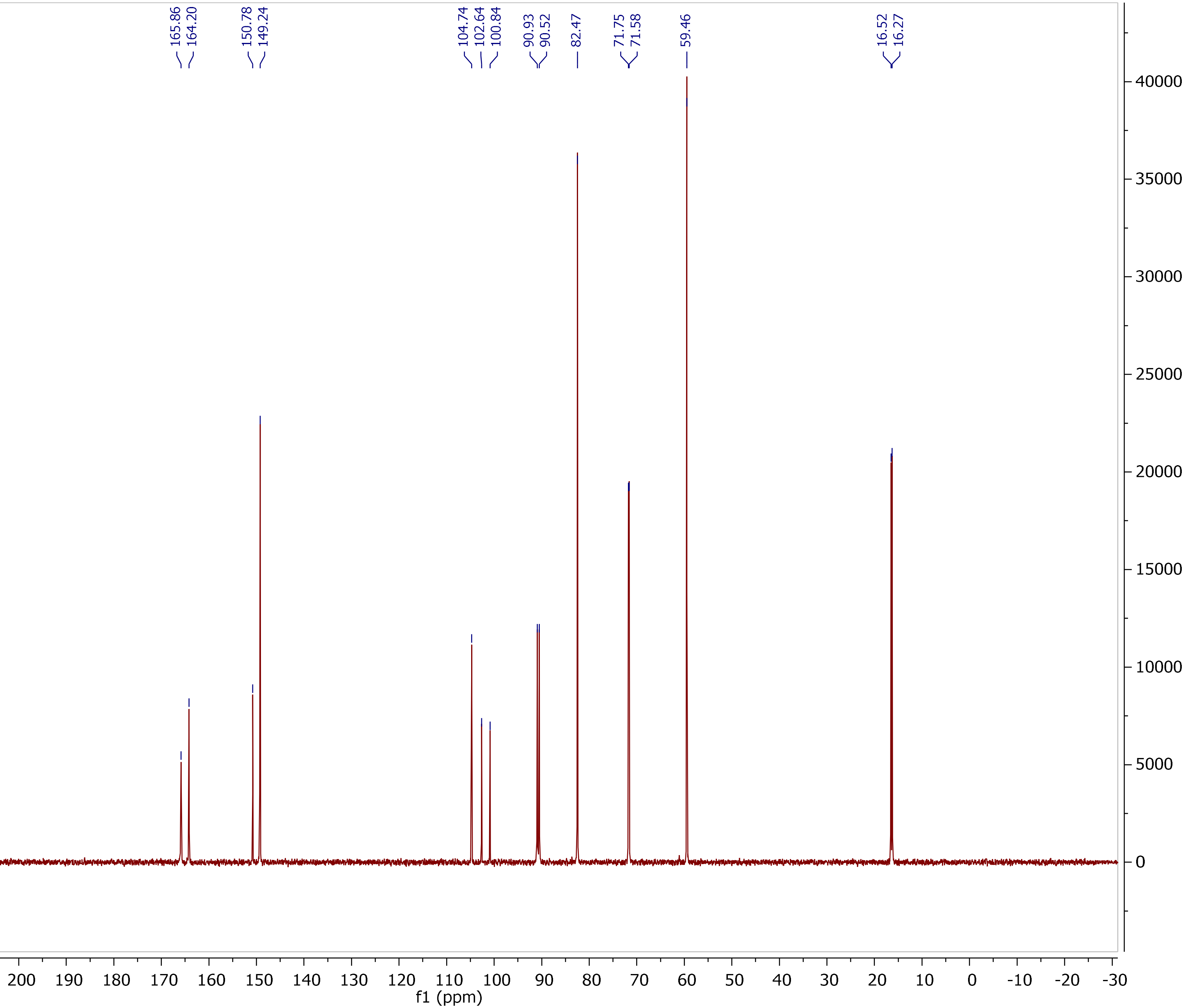

Figure S. 143 - ^{1}H-NMR Spectrum (400 MHz, $CDCl_3$) - 1-(3,5-Di-*O*-benzoyl-2-deoxy-2,2-difluoro-β-*D*-ribofuranosyl)-3-(2,4-dimethoxybenzyl)-2,4-dioxo-1,2,3,4-tetrahydropyrimidine-5-carboxylic acid – **18e**

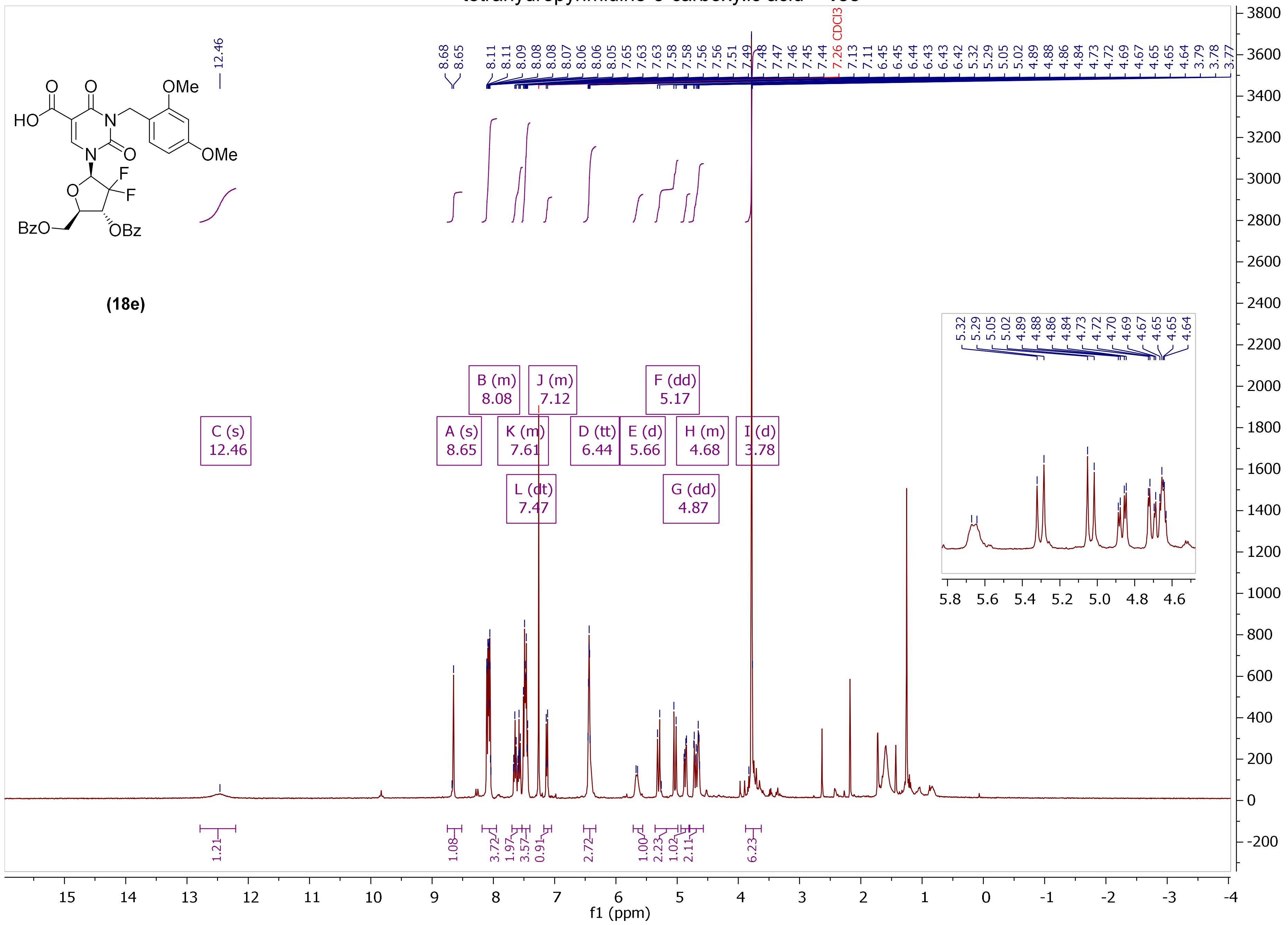

Figure S. 144 - ^{19}F NMR Spectra (377 MHz, $CDCl_3$) - 1-(3,5-Di-*O*-benzoyl-2-deoxy-2,2-difluoro-β-*D*-ribofuranosyl)-3-(2,4-dimethoxybenzyl)-2,4-dioxo-1,2,3,4-tetrahydropyrimidine-5-carboxylic acid – **18e**

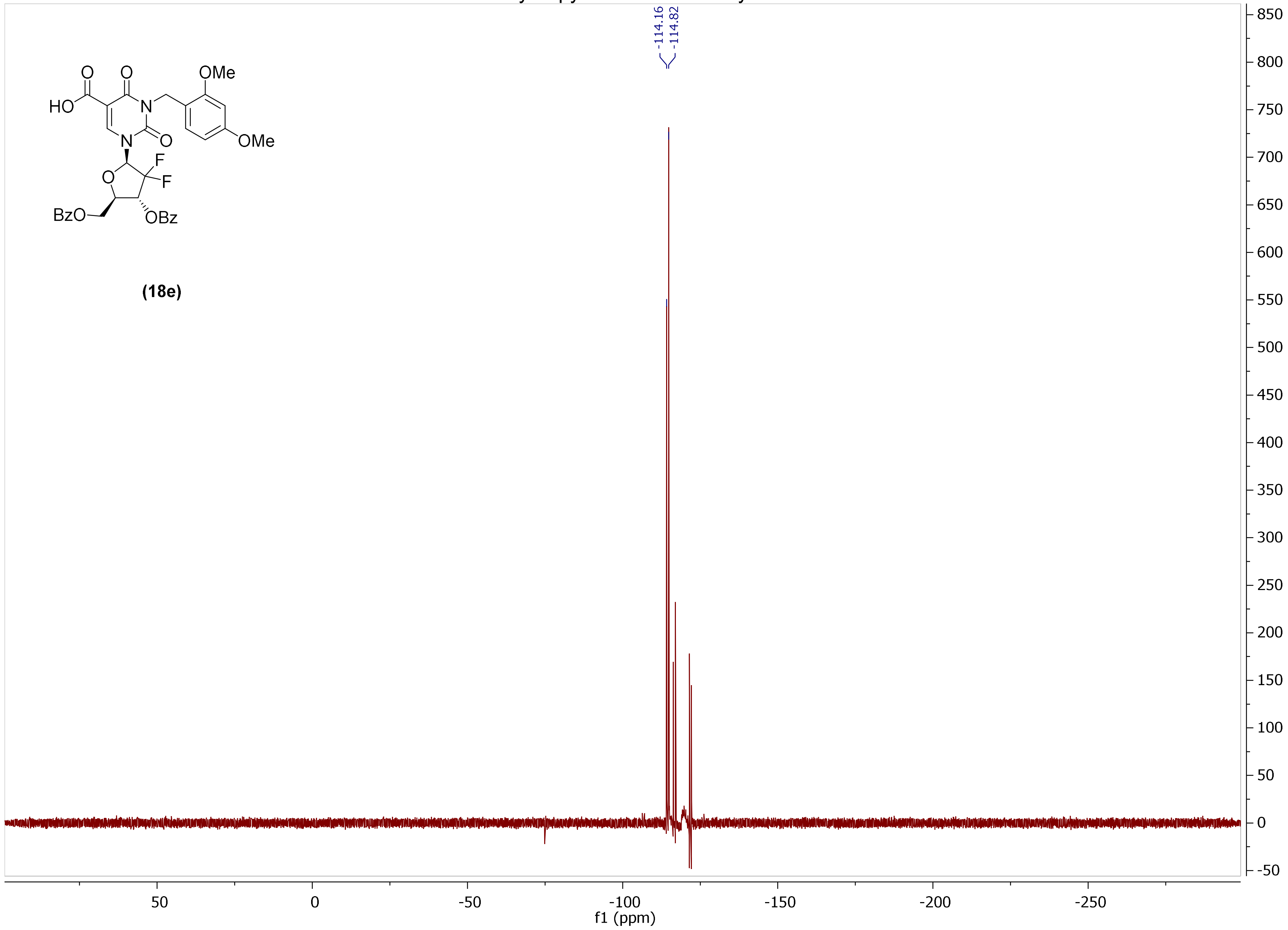

Figure S. 145 - ^{13}C NMR Spectra (101 MHz, $CDCl_3$) - 1-(3,5-Di-*O*-benzoyl-2-deoxy-2,2-difluoro-β-*D*-ribofuranosyl)-3-(2,4-dimethoxybenzyl)-2,4-dioxo-1,2,3,4-tetrahydropyrimidine-5-carboxylic acid – **18e**

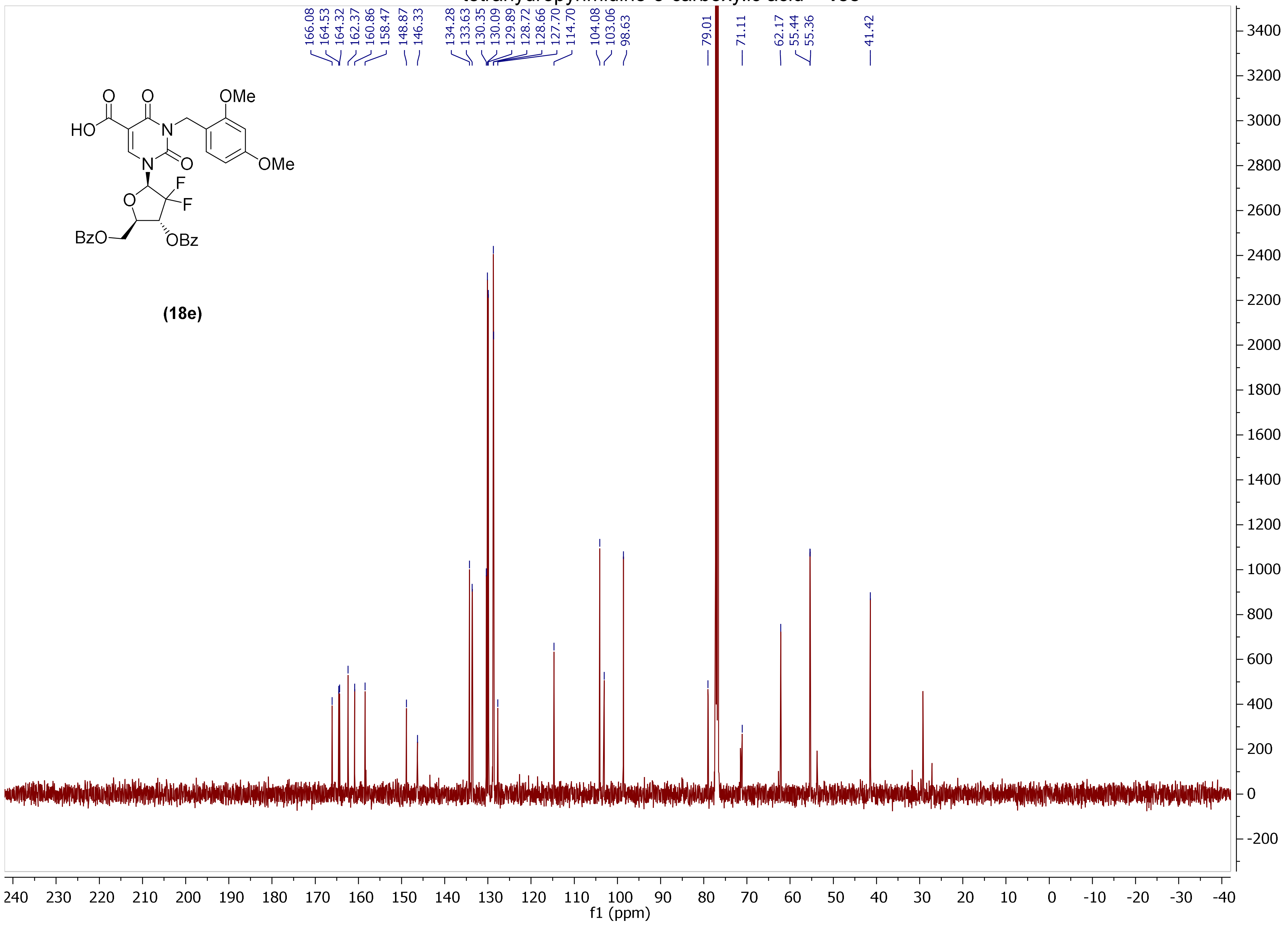

Figure S. 146 - ^{1}H-NMR Spectrum (400 MHz, D_2O) - 1-(2-Deoxy-2,2-difluoro-β-*D*-ribofuranosyl)-2,4-dioxo-1,2,3,4-tetrahydropyrimidine-5-carboxylic acid – **19e**

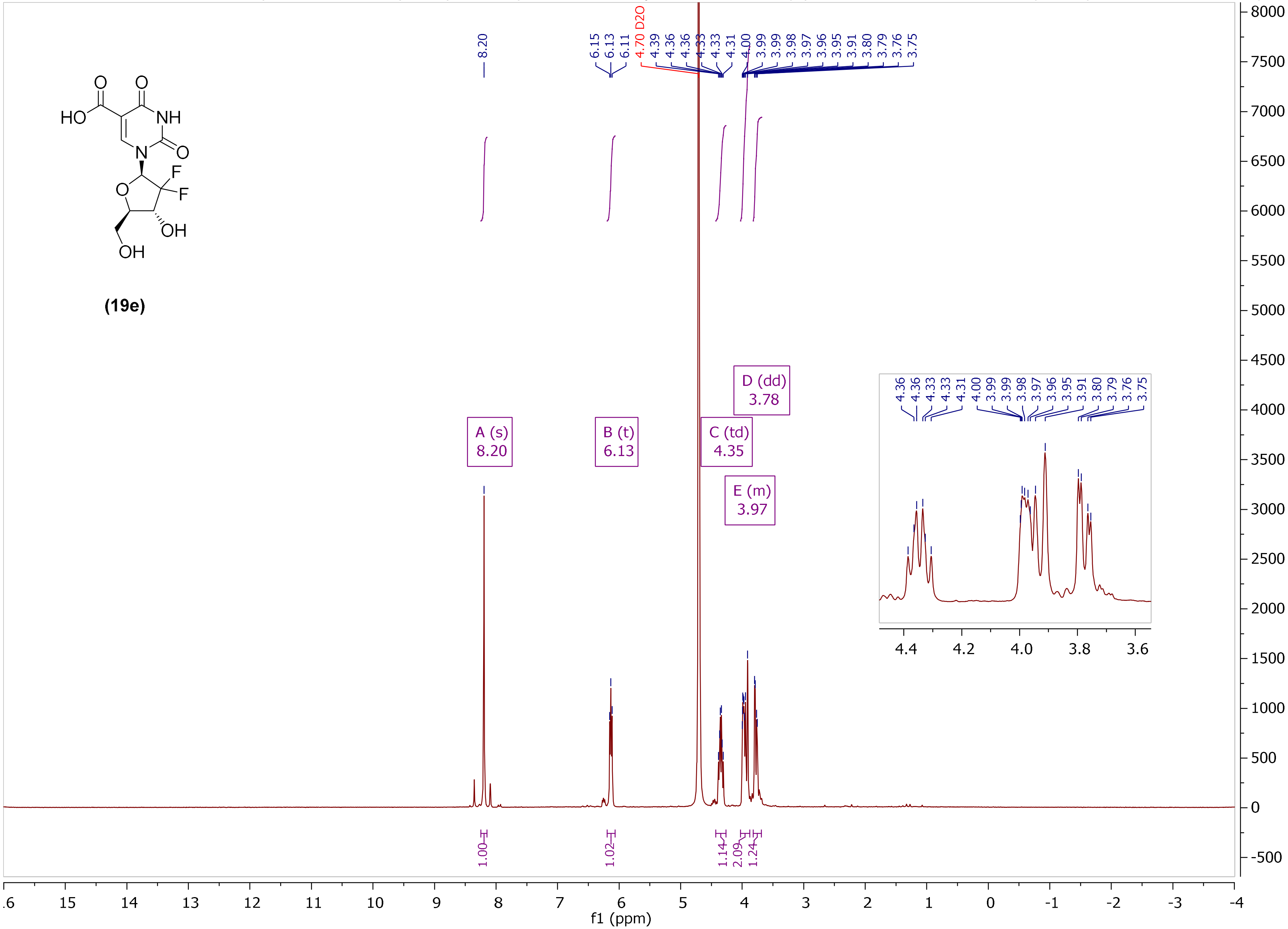

Figure S. 147 - ^{19}F NMR Spectra (377 MHz, D_2O) - 1-(2-Deoxy-2,2-difluoro-β-*D*-ribofuranosyl)-2,4-dioxo-1,2,3,4-tetrahydropyrimidine-5-carboxylic acid – **19e**

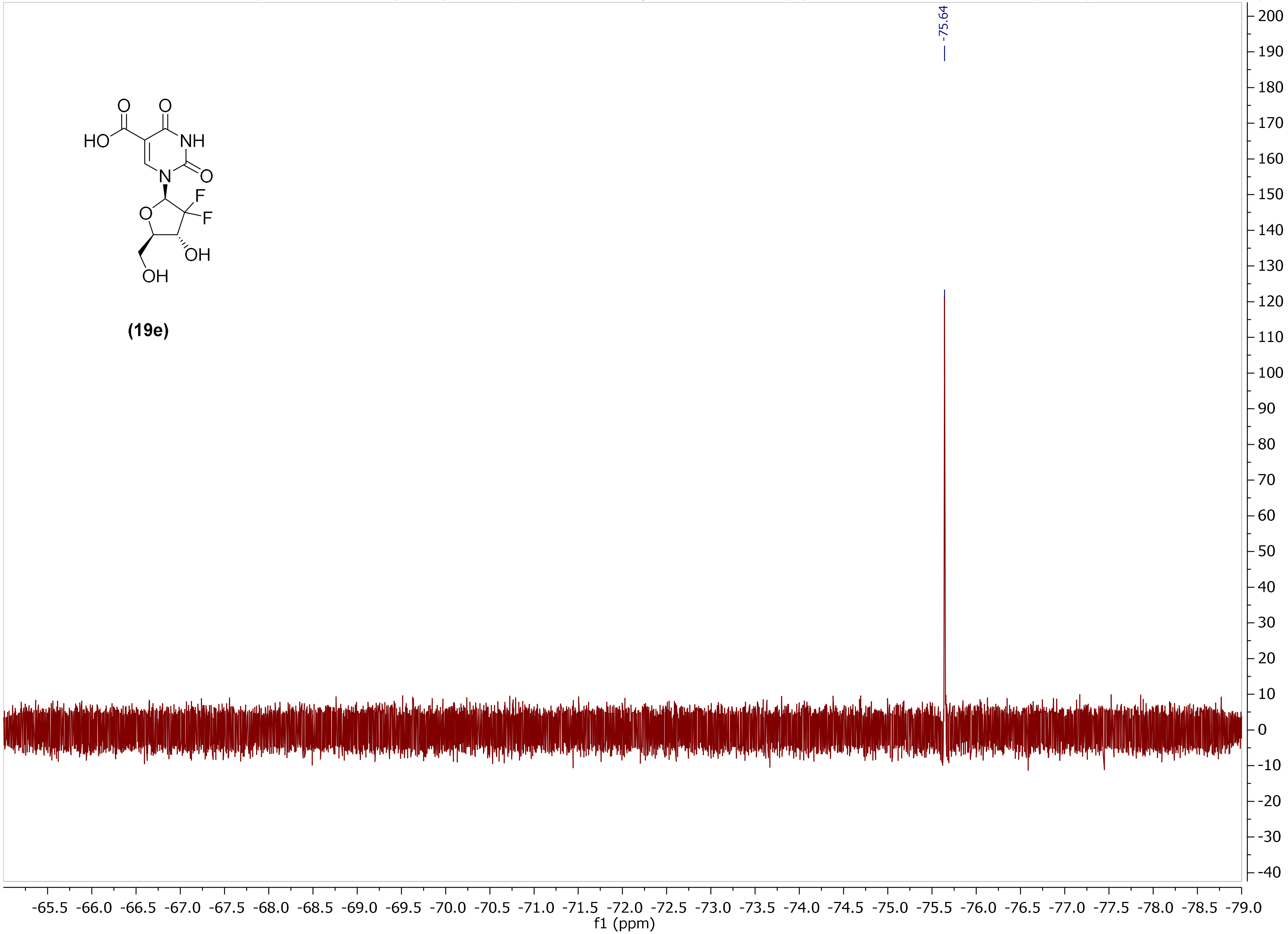

Figure S. 148 - ^{13}C NMR Spectra (101 MHz, D_2O) - 1-(2-Deoxy-2,2-difluoro-β-*D*-ribofuranosyl)-2,4-dioxo-1,2,3,4-tetrahydropyrimidine-5-carboxylic acid – **19e**

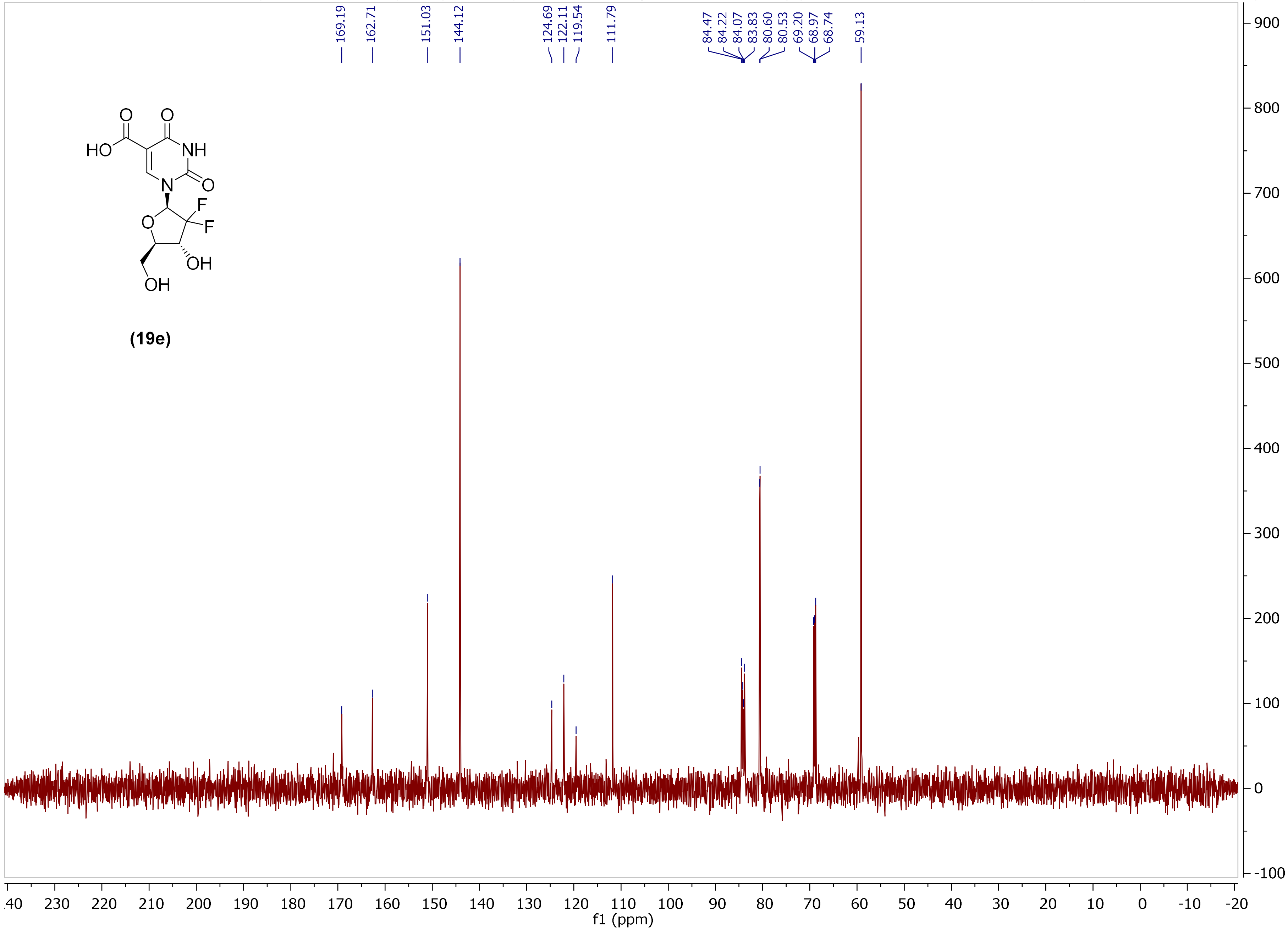

Figure S. 149 - ^{1}H-NMR Spectrum (400 MHz, D_2O) - 1-(2-Deoxy-2,2-difluoro-α-*D*-ribofuranosyl)-2,4-dioxo-1,2,3,4-tetrahydropyrimidine-5-carboxylic acid – (1-epi-**19e**)

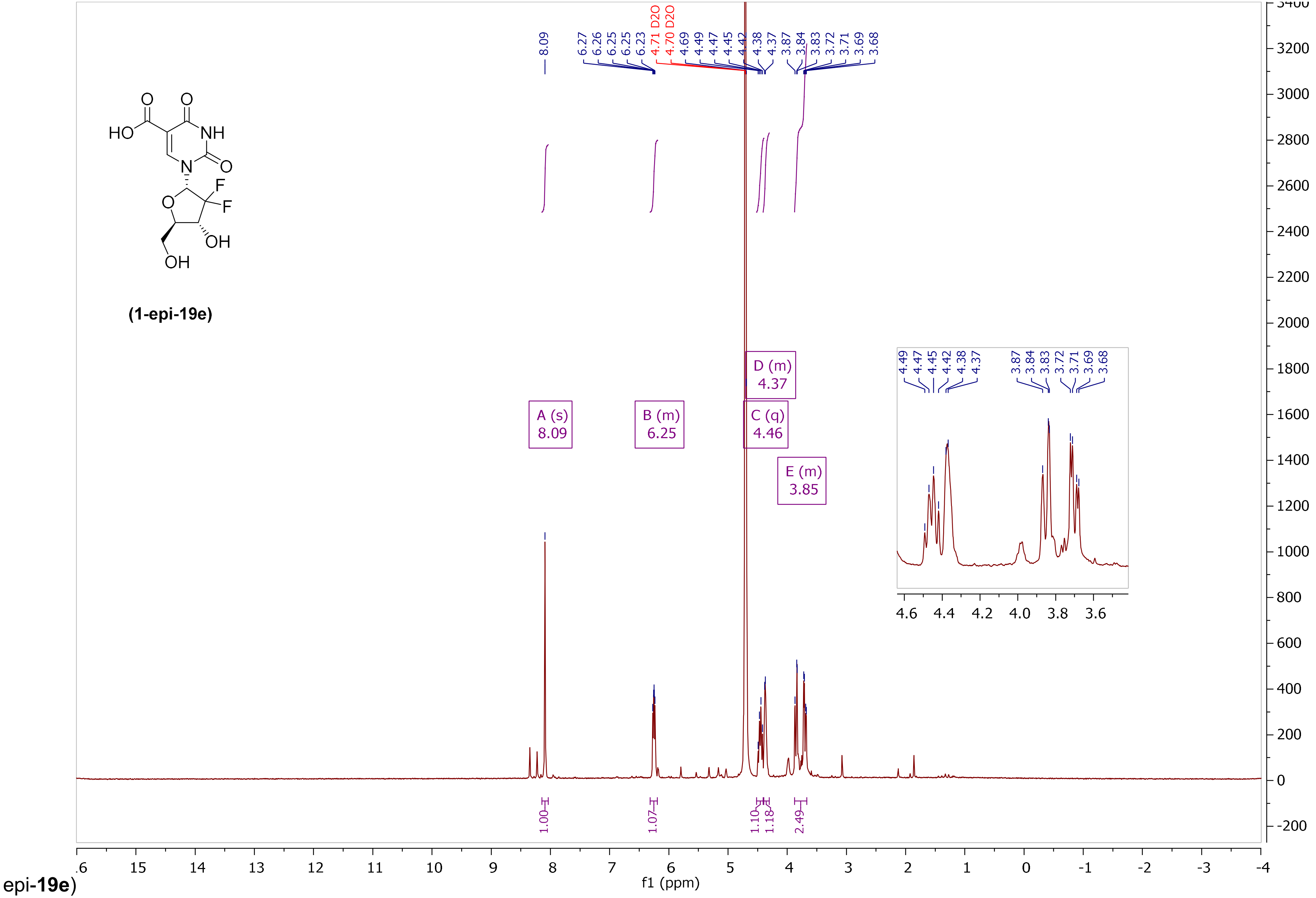

Figure S. 150 - ^{19}F NMR Spectra (377 MHz, D_2O) - 1-(2-Deoxy-2,2-difluoro-α-*D*-ribofuranosyl)-2,4-dioxo-1,2,3,4-tetrahydropyrimidine-5-carboxylic acid – (1-epi-**19e**)

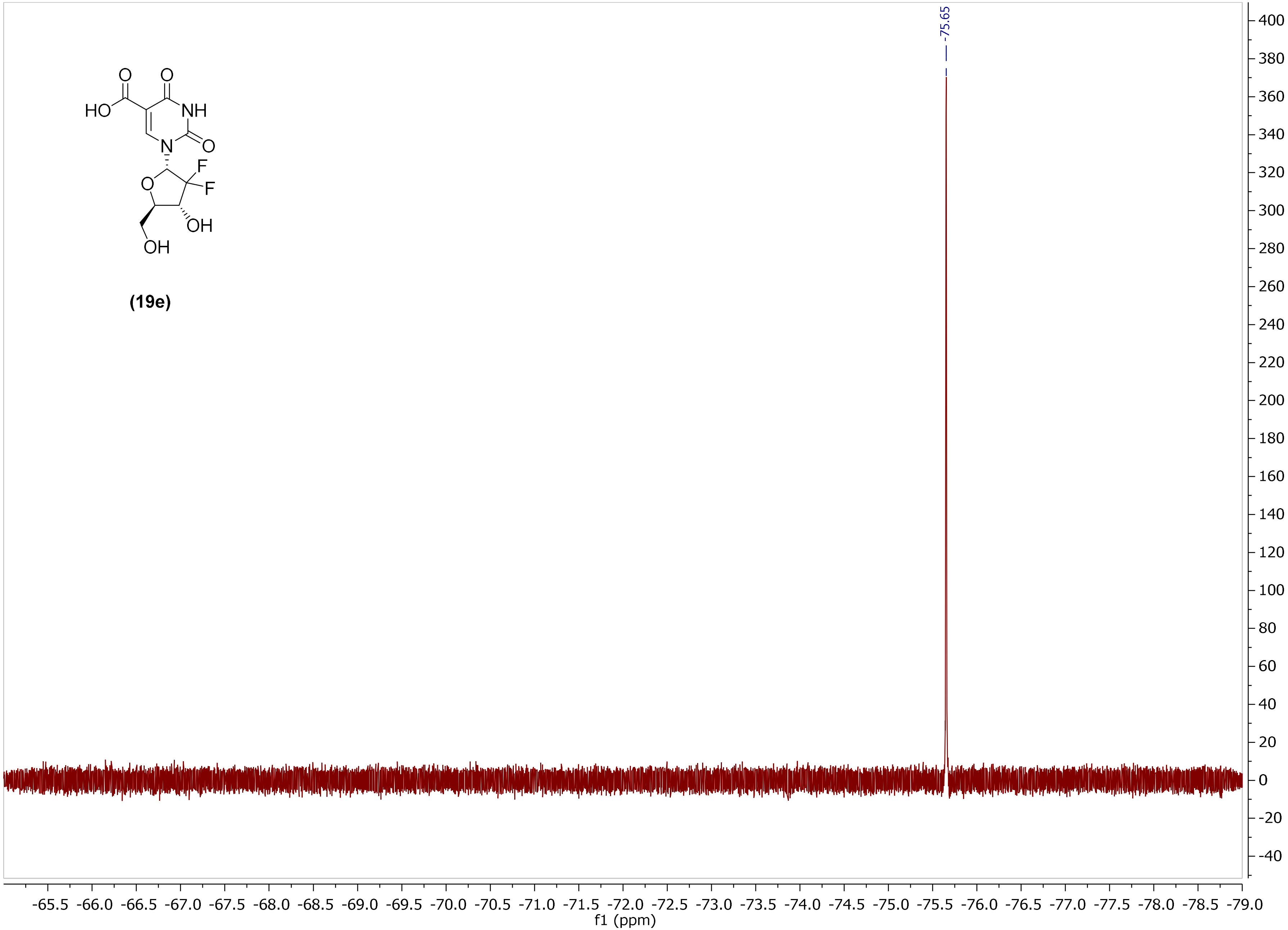

Figure S. 151 - ^{13}C NMR Spectra (101 MHz, D_2O) - 1-(2-Deoxy-2,2-difluoro-α-*D*-ribofuranosyl)-2,4-dioxo-1,2,3,4-tetrahydropyrimidine-5-carboxylic acid – (1-epi-**19e**)

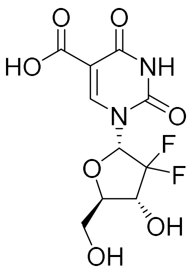

(1-epi-19e)

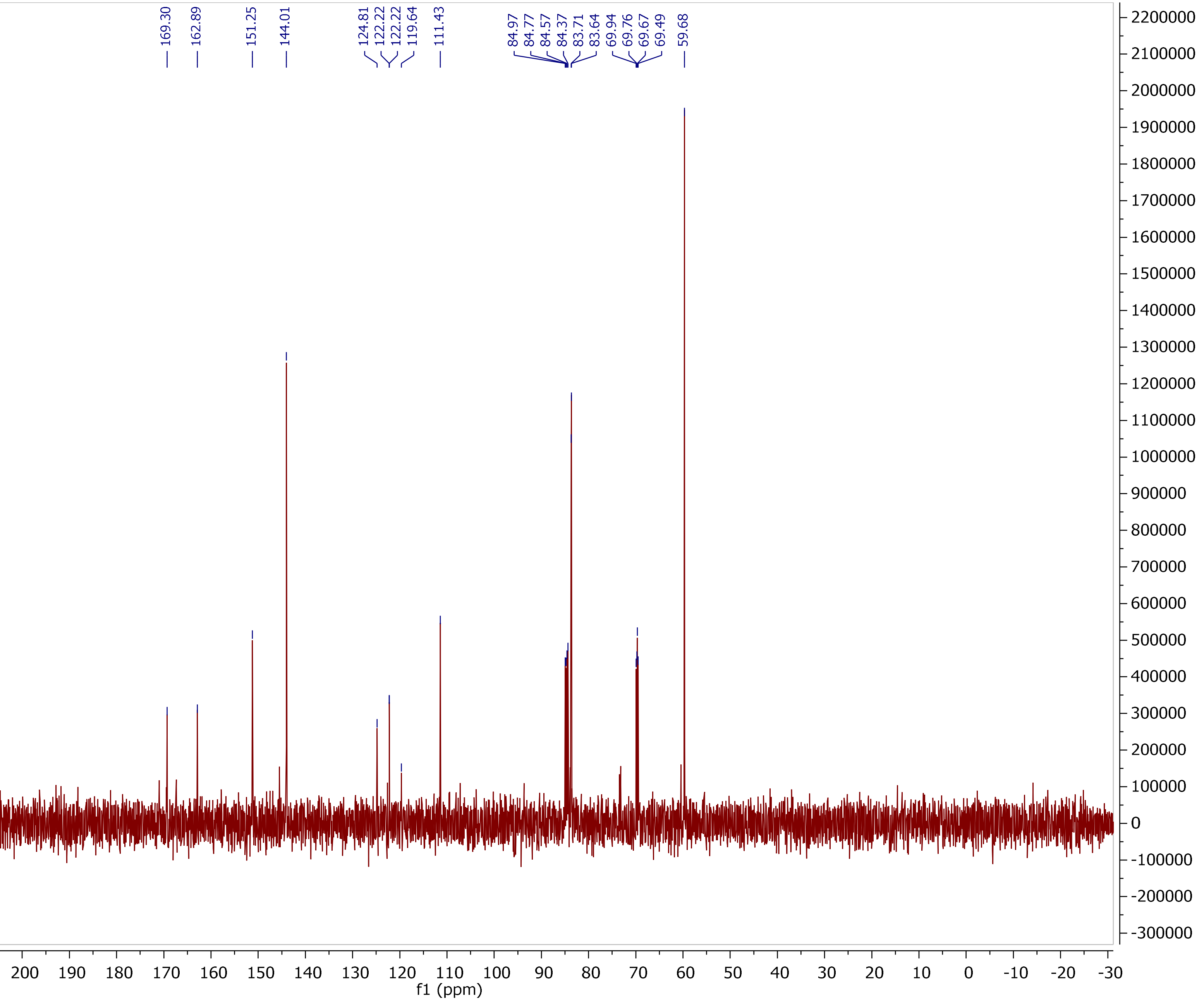

Figure S. 152 - ^{1}H-NMR Spectrum (400 MHz, D_2O) - 1-(2-Deoxy-β-*D*-ribofuranosyl)-5-bromo-2,4-dioxo-1,2,3,4-tetrahydropyrimidine – **20a**

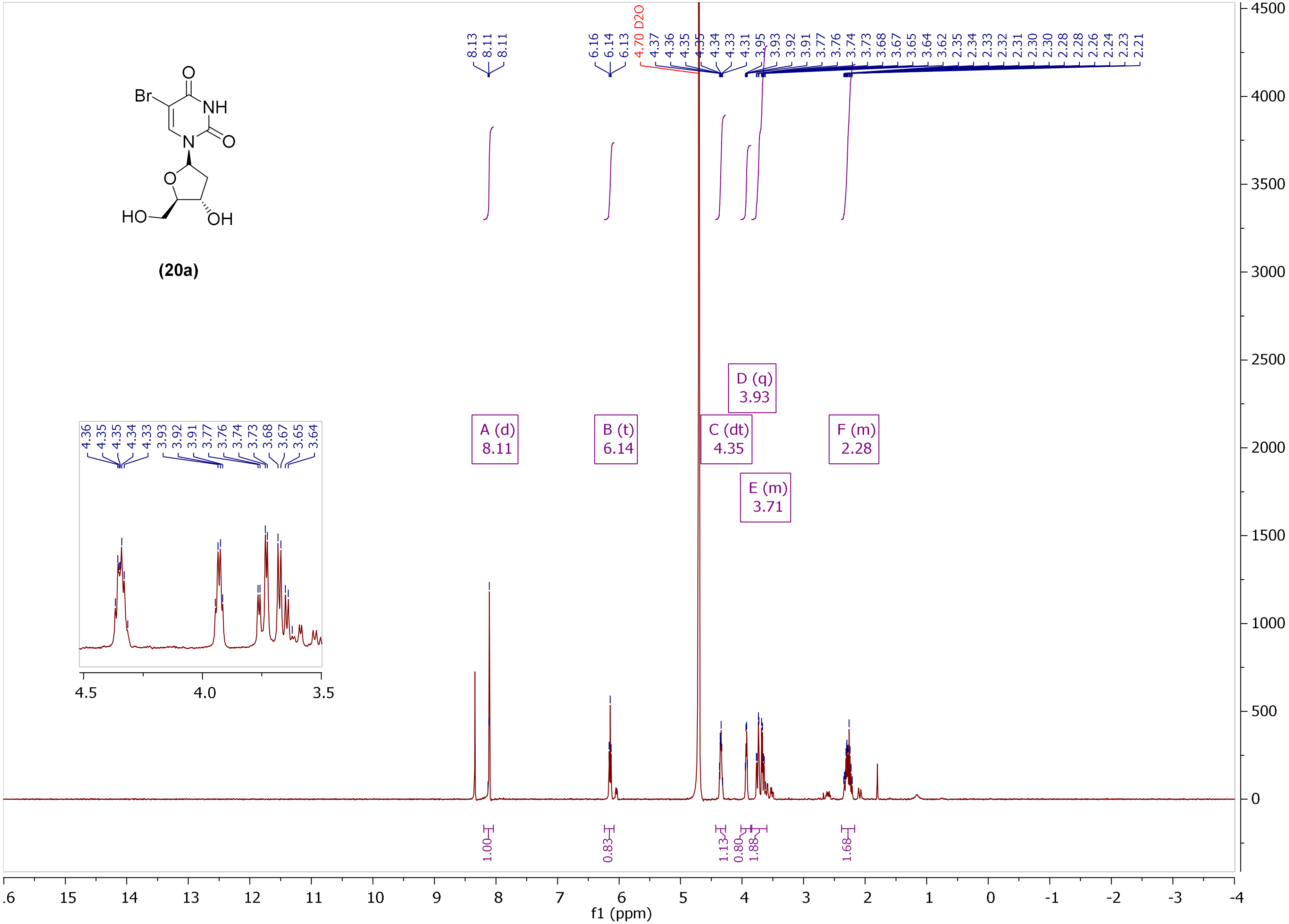

Figure S. 153 - ^{13}C NMR Spectra (101 MHz, D_2O) - 1-(2-Deoxy-β-*D*-ribofuranosyl)-5-bromo-2,4-dioxo-1,2,3,4-tetrahydropyrimidine – **20a**

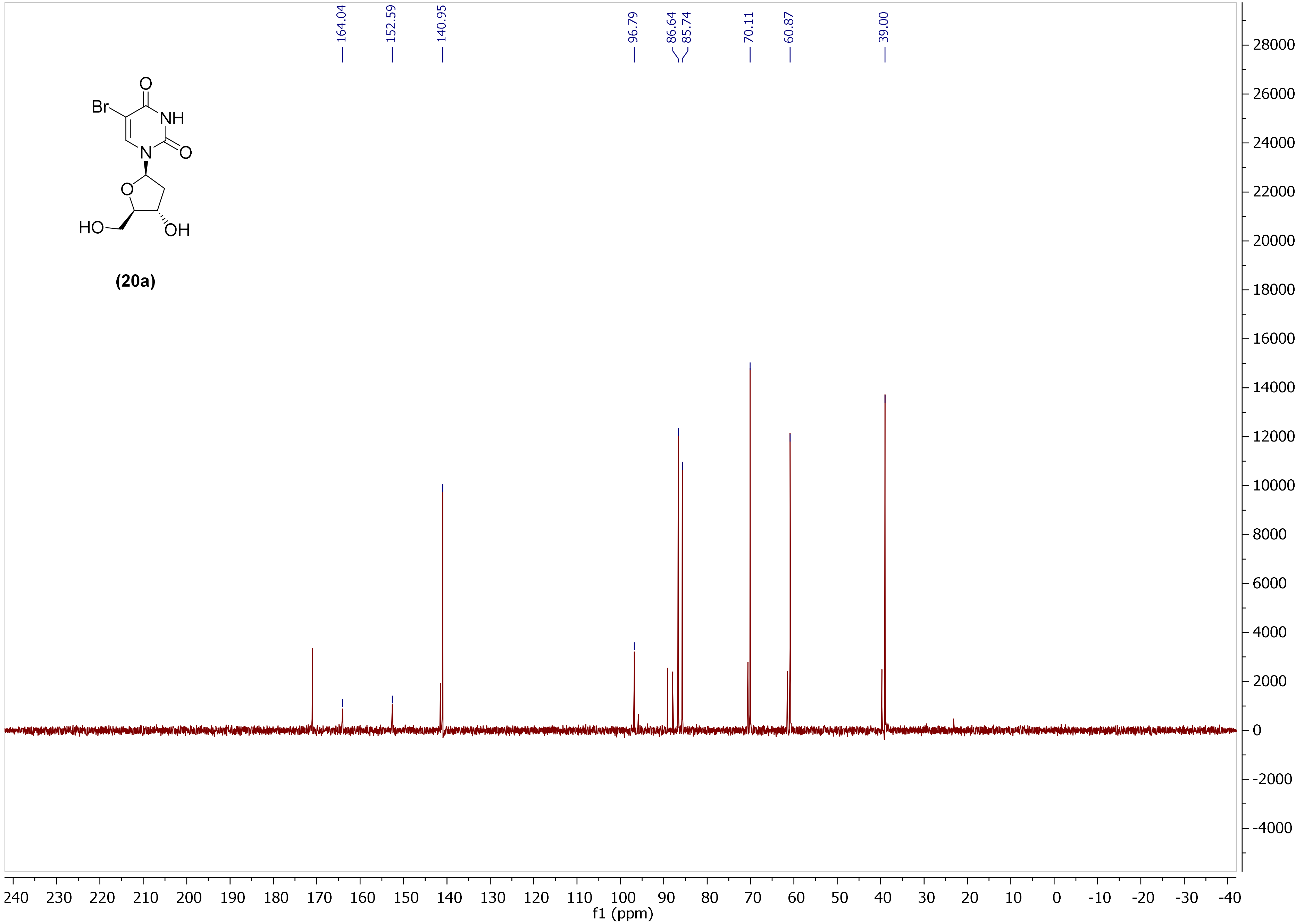

Figure S. 154. - ^{1}H-NMR Spectrum (400 MHz, D_2O) - 1-(β-*D*-Ribofuranosyl)-5-bromo-2,4-dioxo-1,2,3,4-tetrahydropyrimidine – **20b**

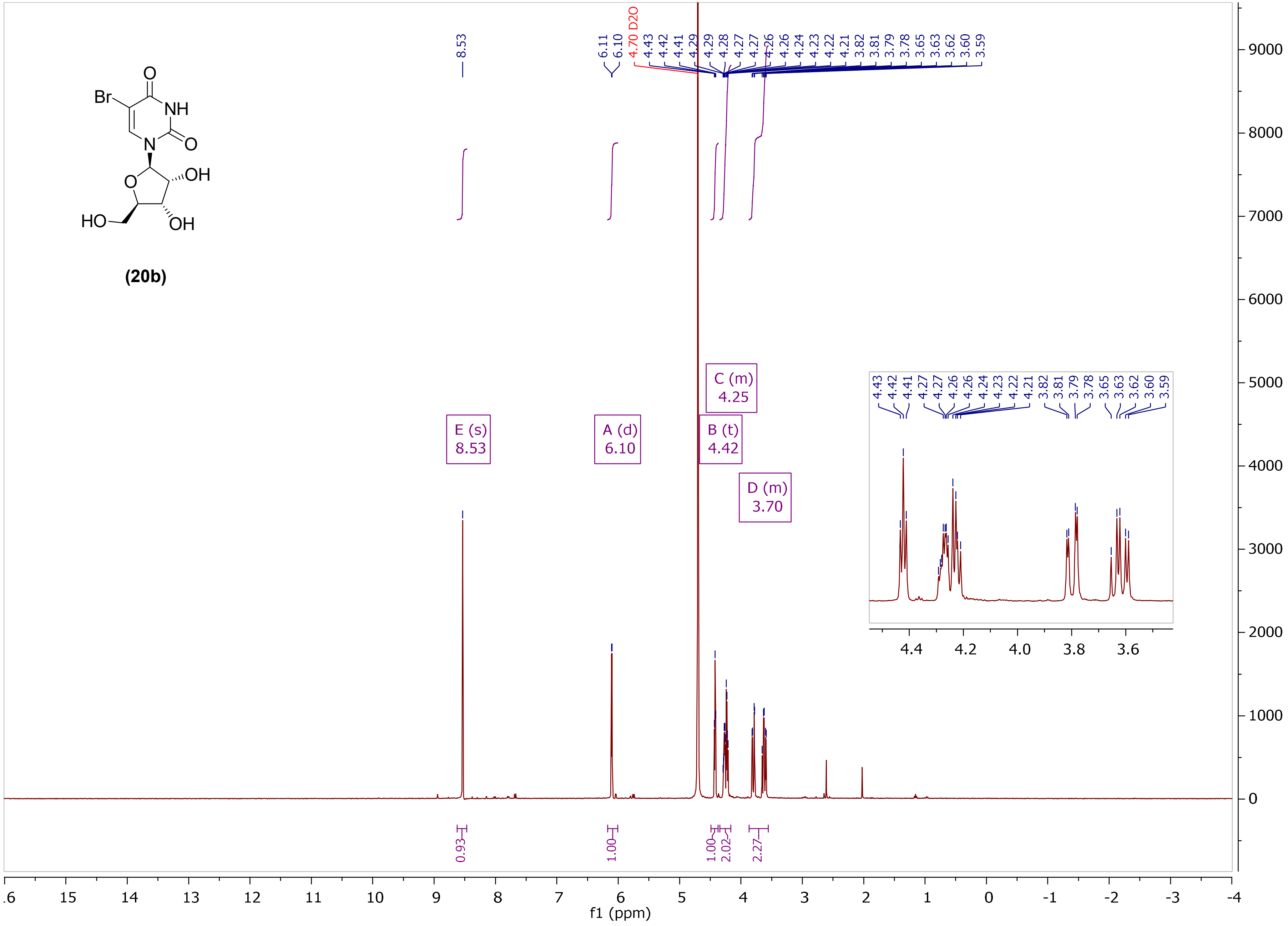

Figure S. 155 - ^{13}C NMR Spectra (101 MHz, D_2O) - 1-(β-*D*-Ribofuranosyl)-5-bromo-2,4-dioxo-1,2,3,4-tetrahydropyrimidine – **20b**

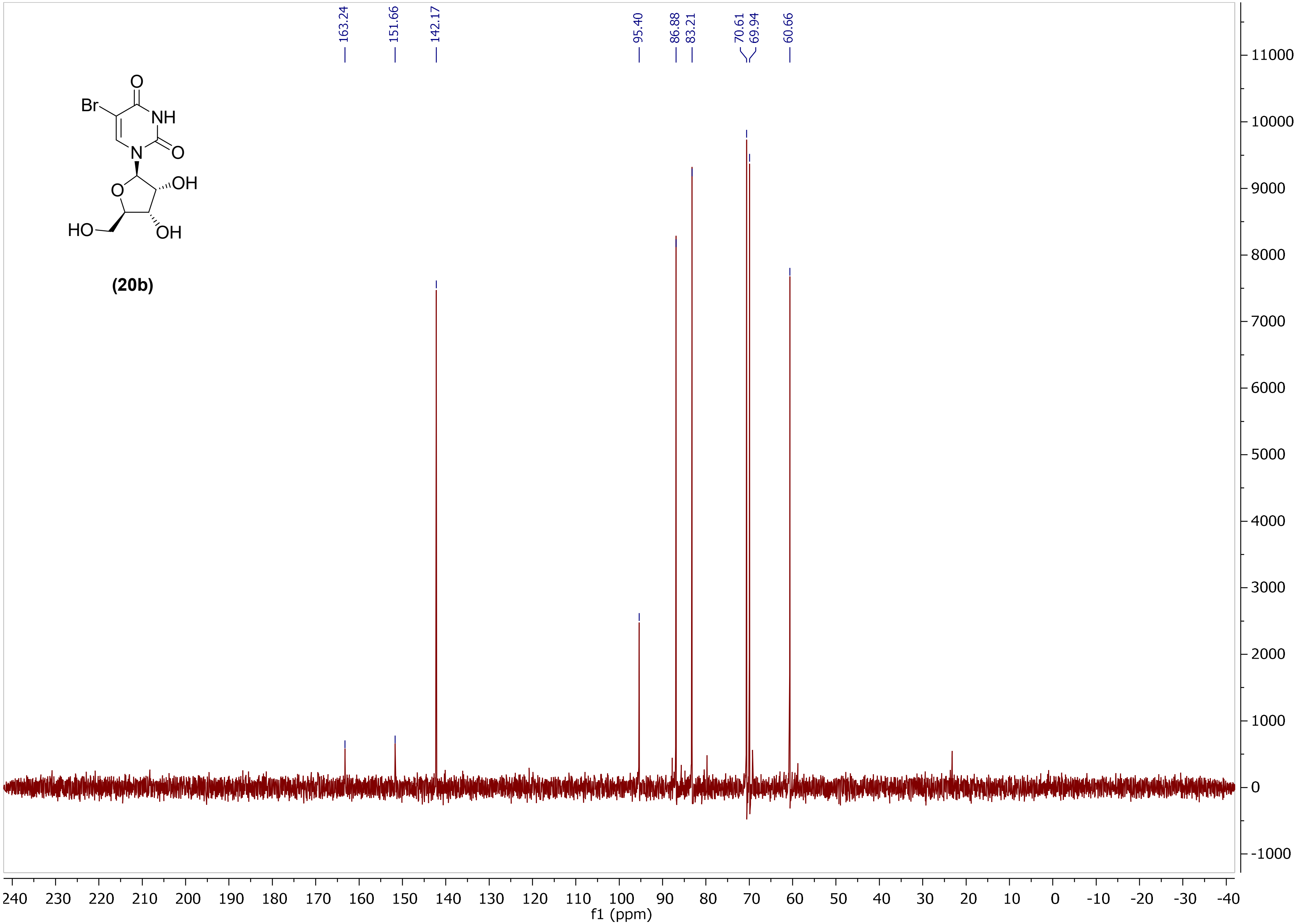

Figure S. 156 - ^{1}H-NMR Spectrum (400 MHz, D_2O) - 1-(2-Deoxy-2-fluoro-β-*D*-arabinofuranosyl)-5-bromo-2,4-dioxo-1,2,3,4-tetrahydropyrimidine – **20c**

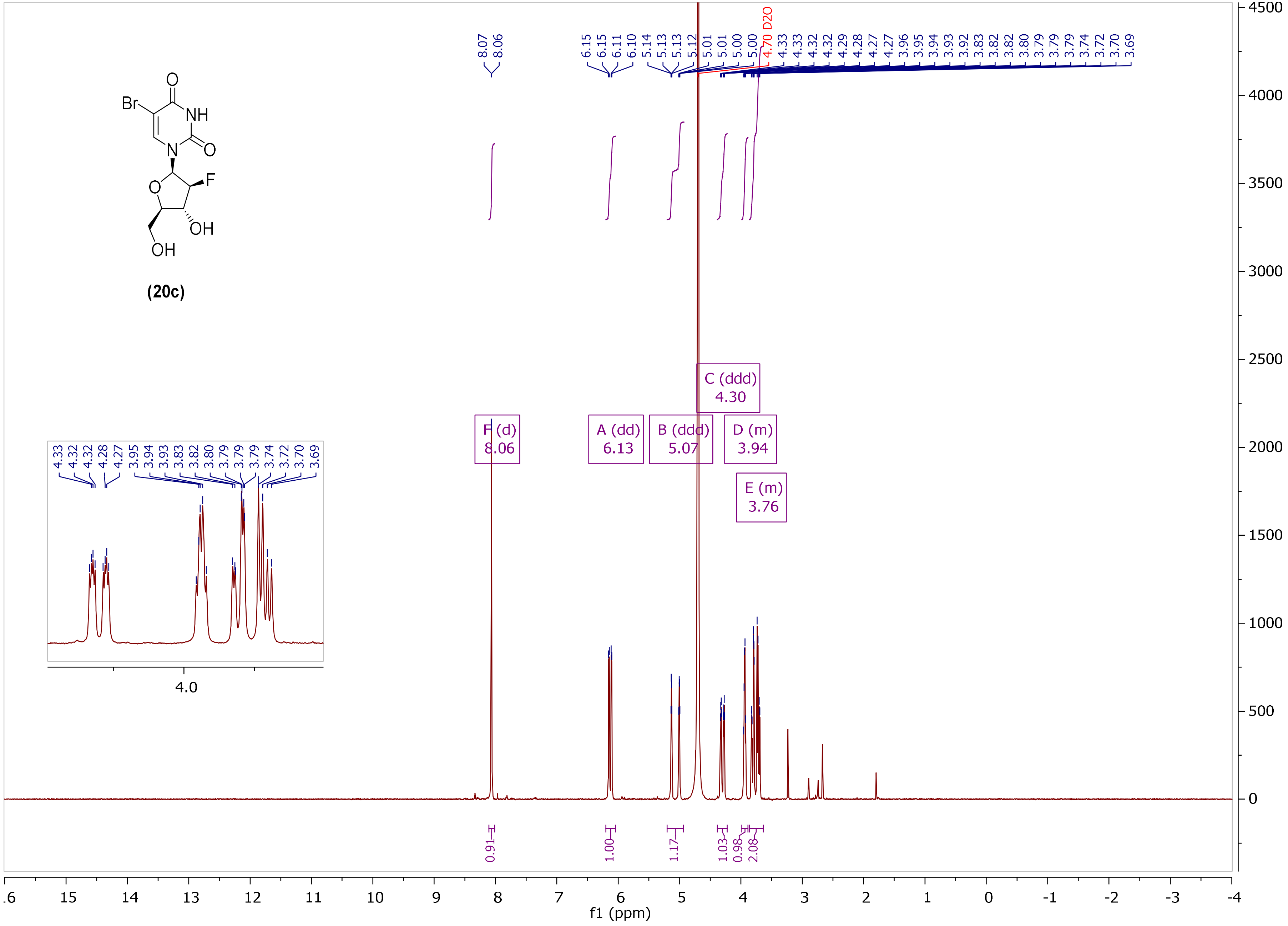

Figure S. 157 - ^{19}F NMR Spectra (377 MHz, D_2O) - 1-(2-Deoxy-2-fluoro-β-*D*-arabinofuranosyl)-5-bromo-2,4-dioxo-1,2,3,4-tetrahydropyrimidine – **20c**

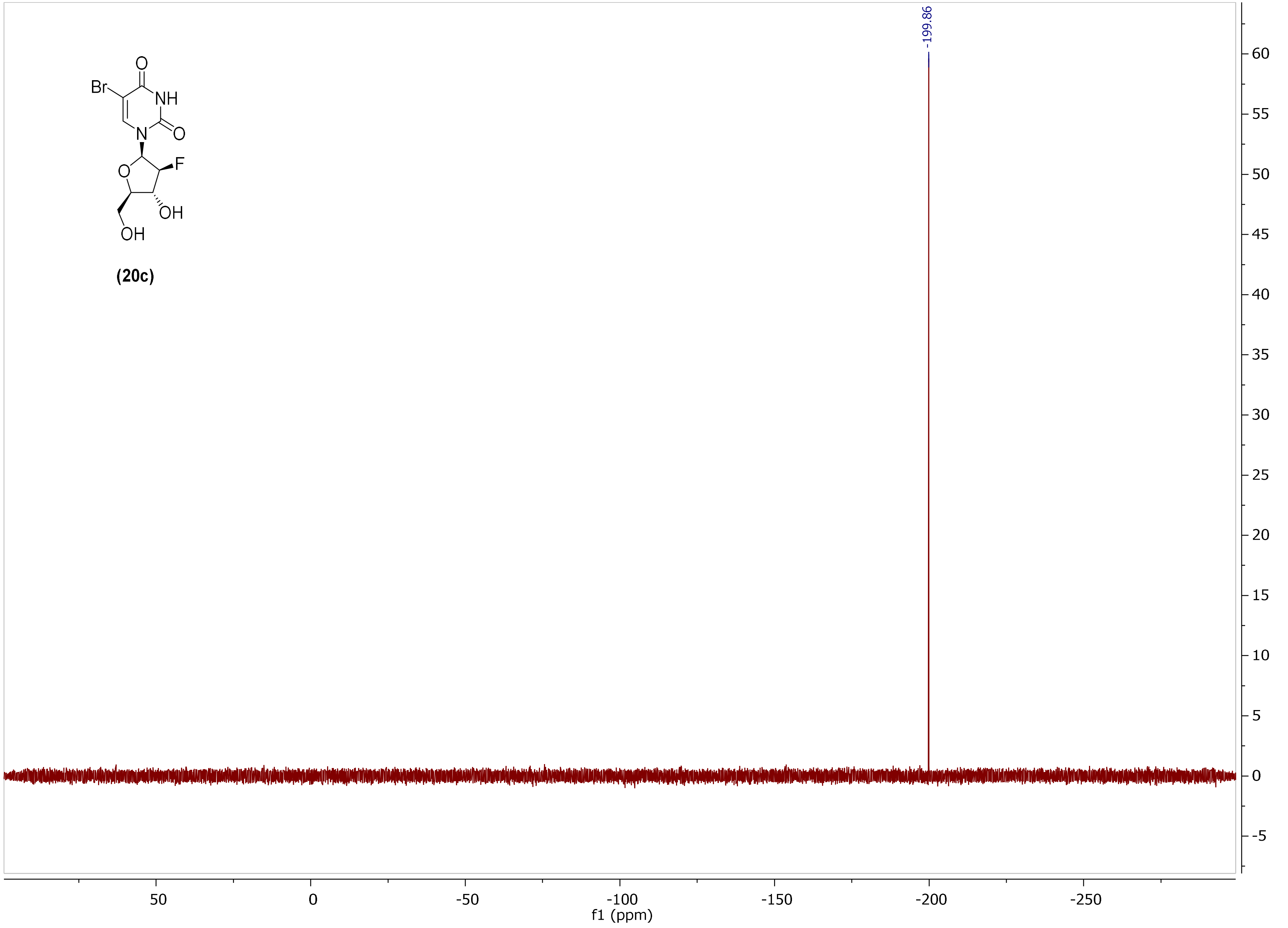

Figure S. 158 - ^{13}C NMR Spectra (101 MHz, D_2O) - 1-(2-Deoxy-2-fluoro-β-*D*-arabinofuranosyl)-5-bromo-2,4-dioxo-1,2,3,4-tetrahydropyrimidine – **20c**

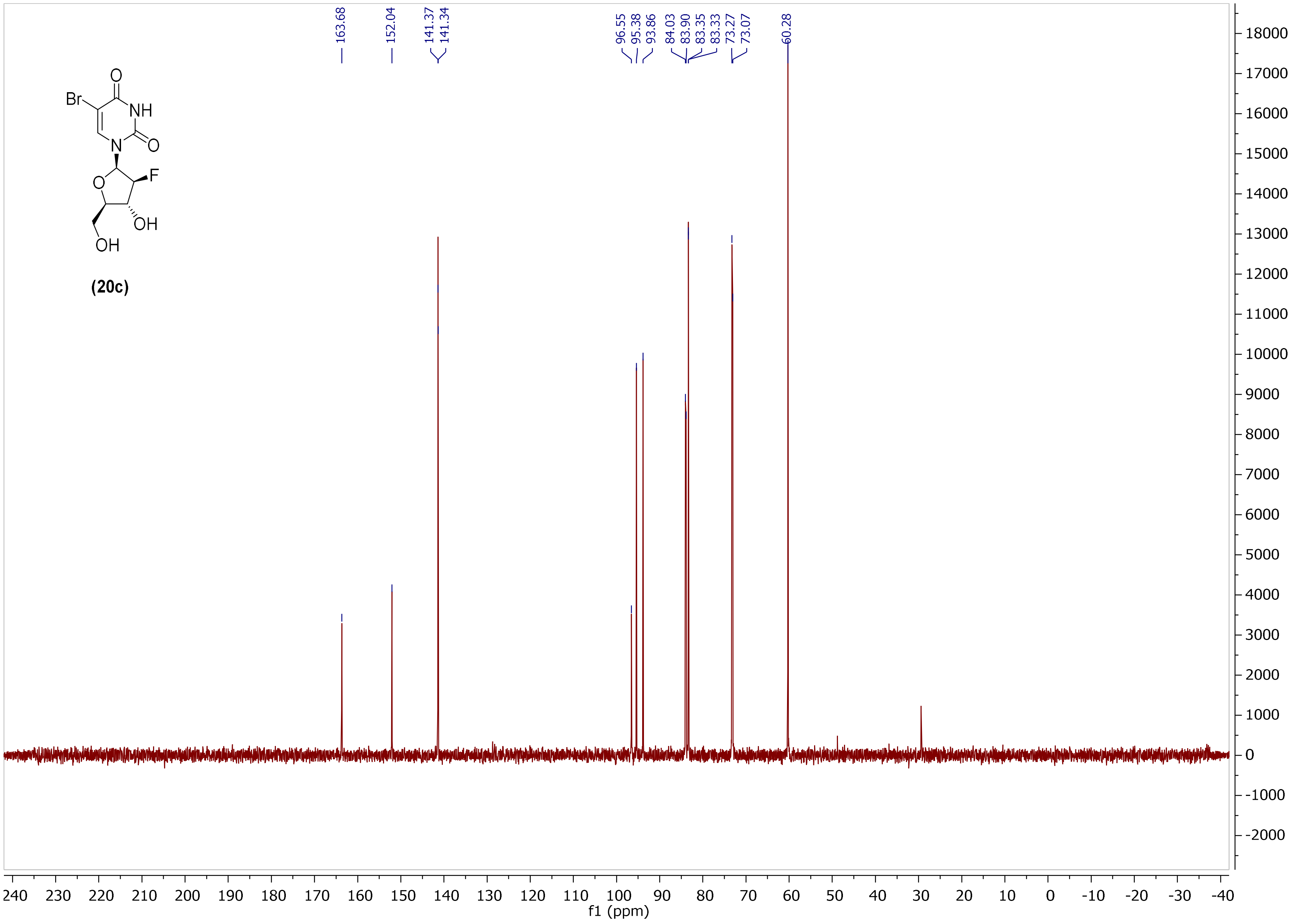

Figure S. 159 - ^{1}H-NMR Spectrum (400 MHz, D_2O) - 1-(2-Deoxy-2-fluoro-2-methyl-β-*D*-ribofuranosyl)-5-bromo-2,4-dioxo-1,2,3,4-tetrahydropyrimidine – **20d**

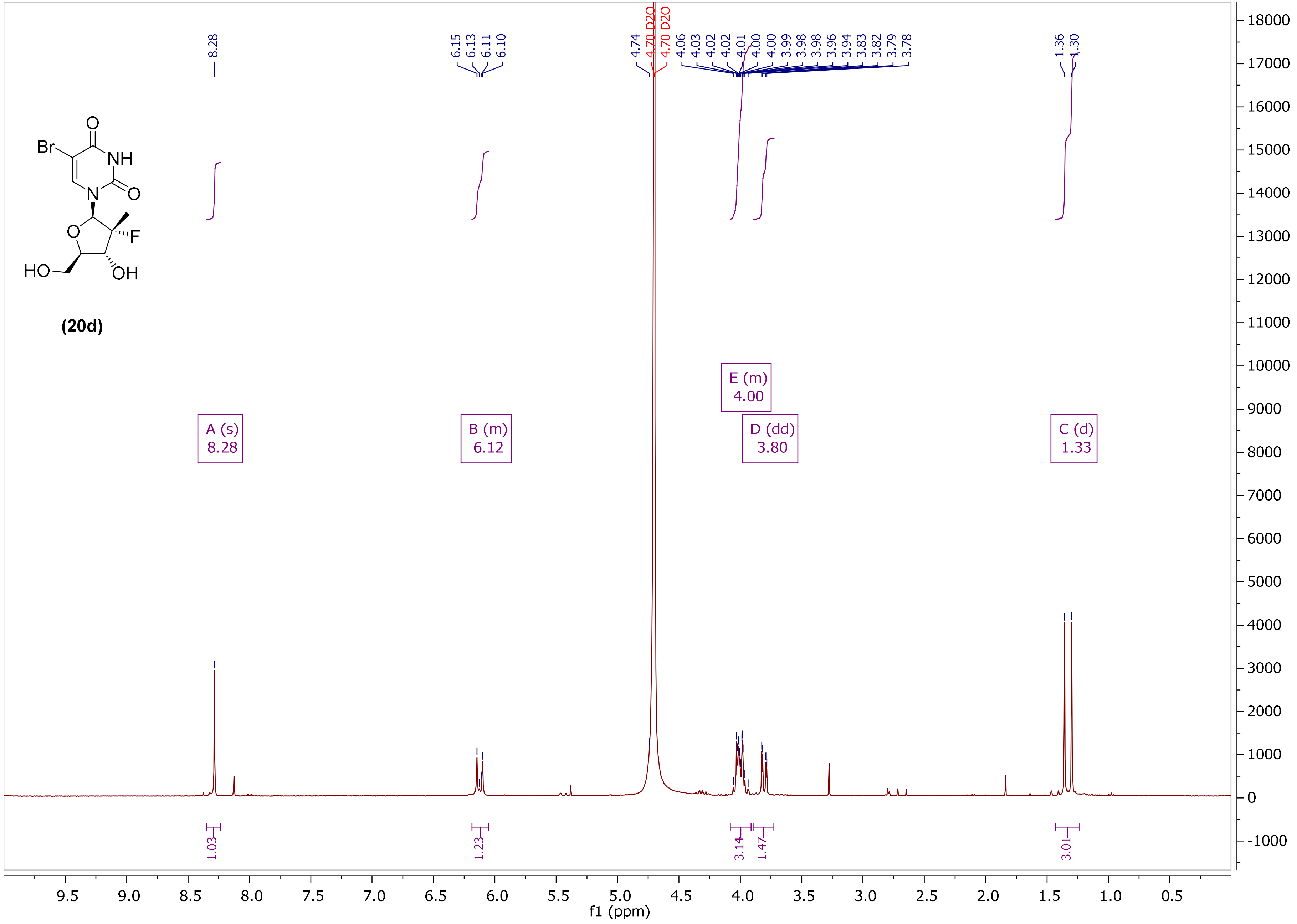

Figure S. 160 - ^{13}C NMR Spectra (101 MHz, D_2O) - 1-(2-Deoxy-2-fluoro-2-methyl-β-*D*-ribofuranosyl)-5-bromo-2,4-dioxo-1,2,3,4-tetrahydropyrimidine – **20d**

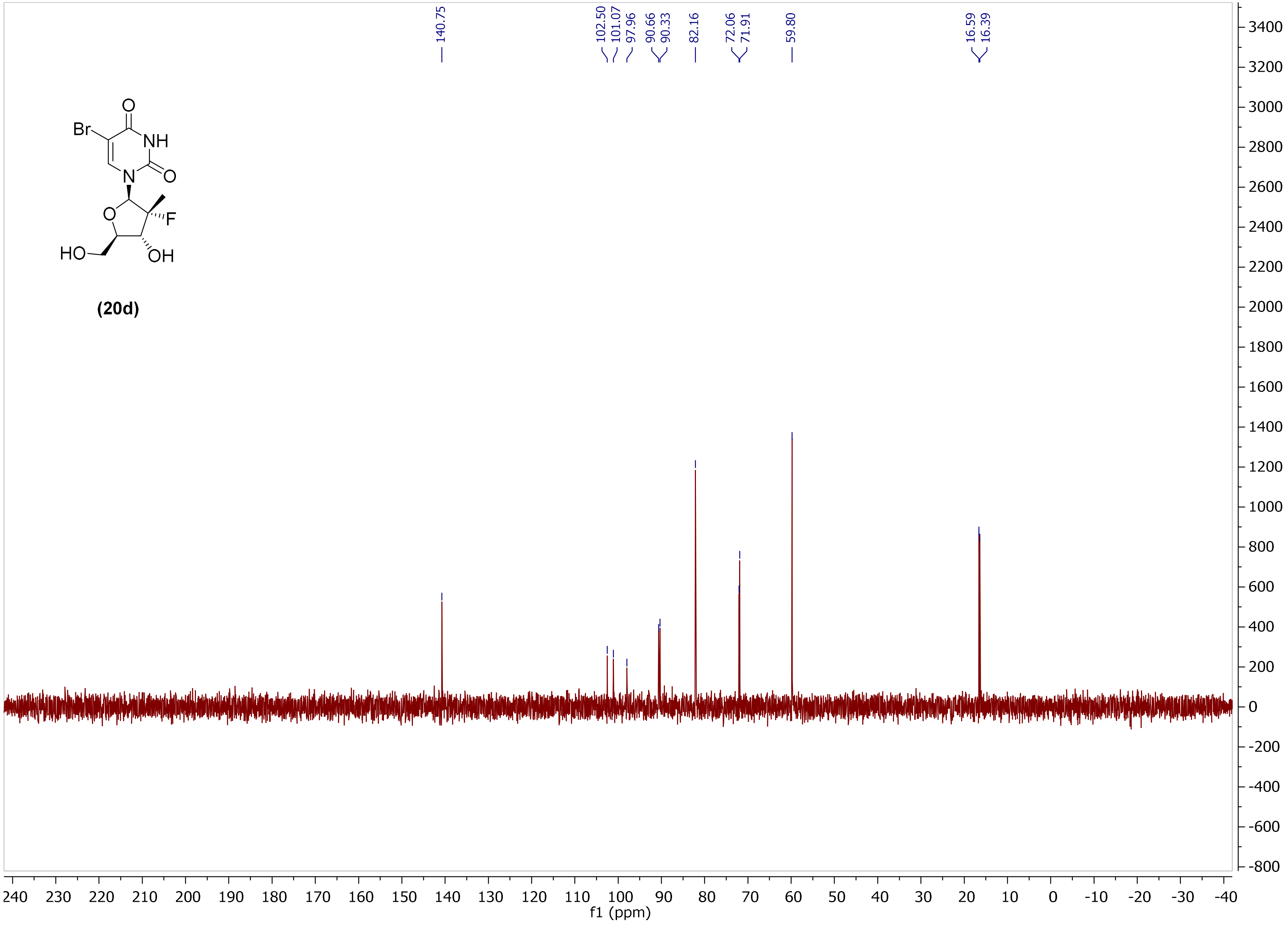

Figure S. 161 - ^{1}H-NMR Spectrum (400 MHz, D_2O) - 1-(2-Deoxy-2,2-difluoro-β-*D*-ribofuranosyl)-5-bromo-2,4-dioxo-1,2,3,4-tetrahydropyrimidine – **20e**

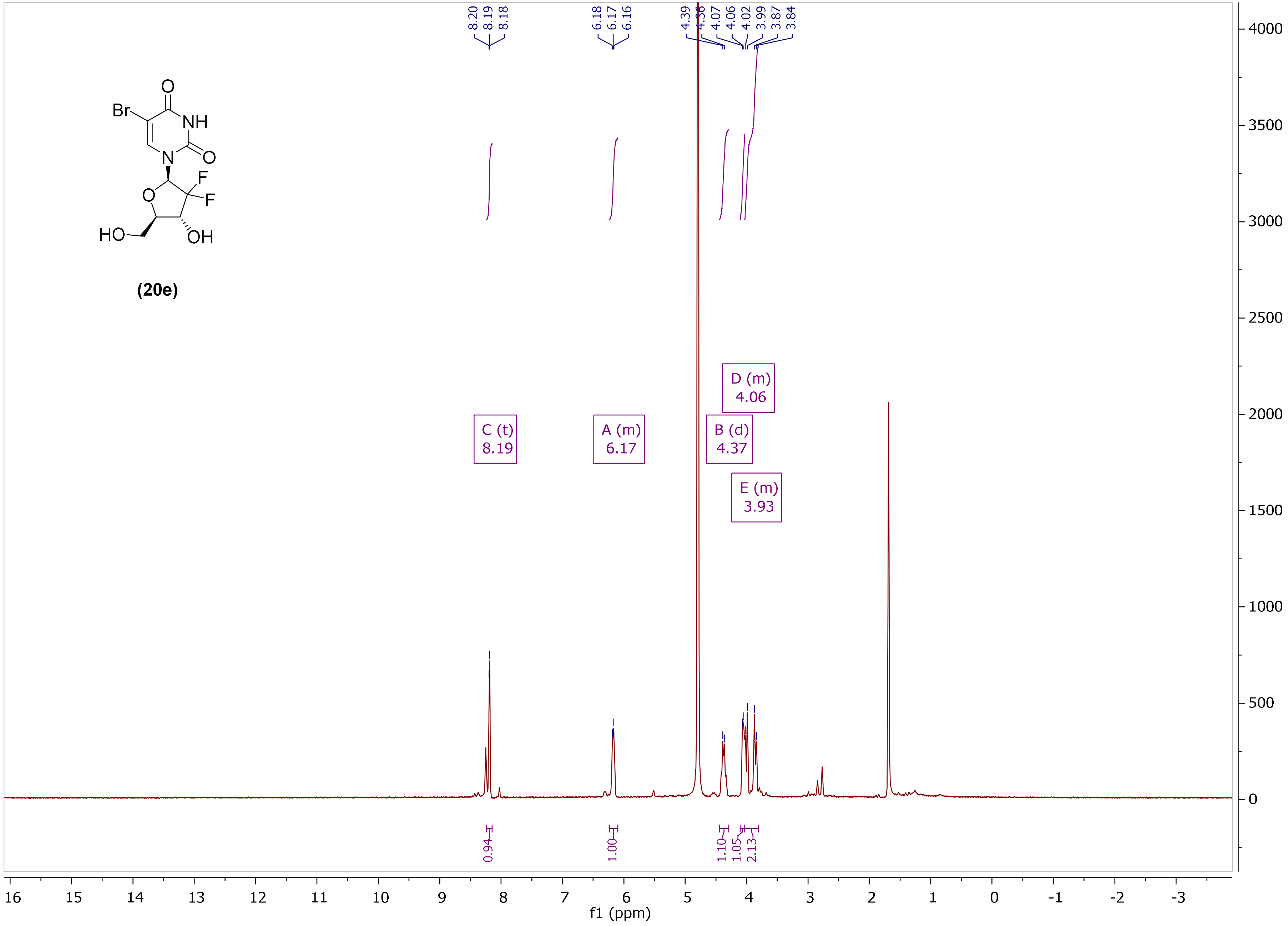

Figure S. 162 - ^{13}C NMR Spectra (101 MHz, D_2O) - 1-(2-Deoxy-2,2-difluoro-β-*D*-ribofuranosyl)-5-bromo-2,4-dioxo-1,2,3,4-tetrahydropyrimidine – **20e**

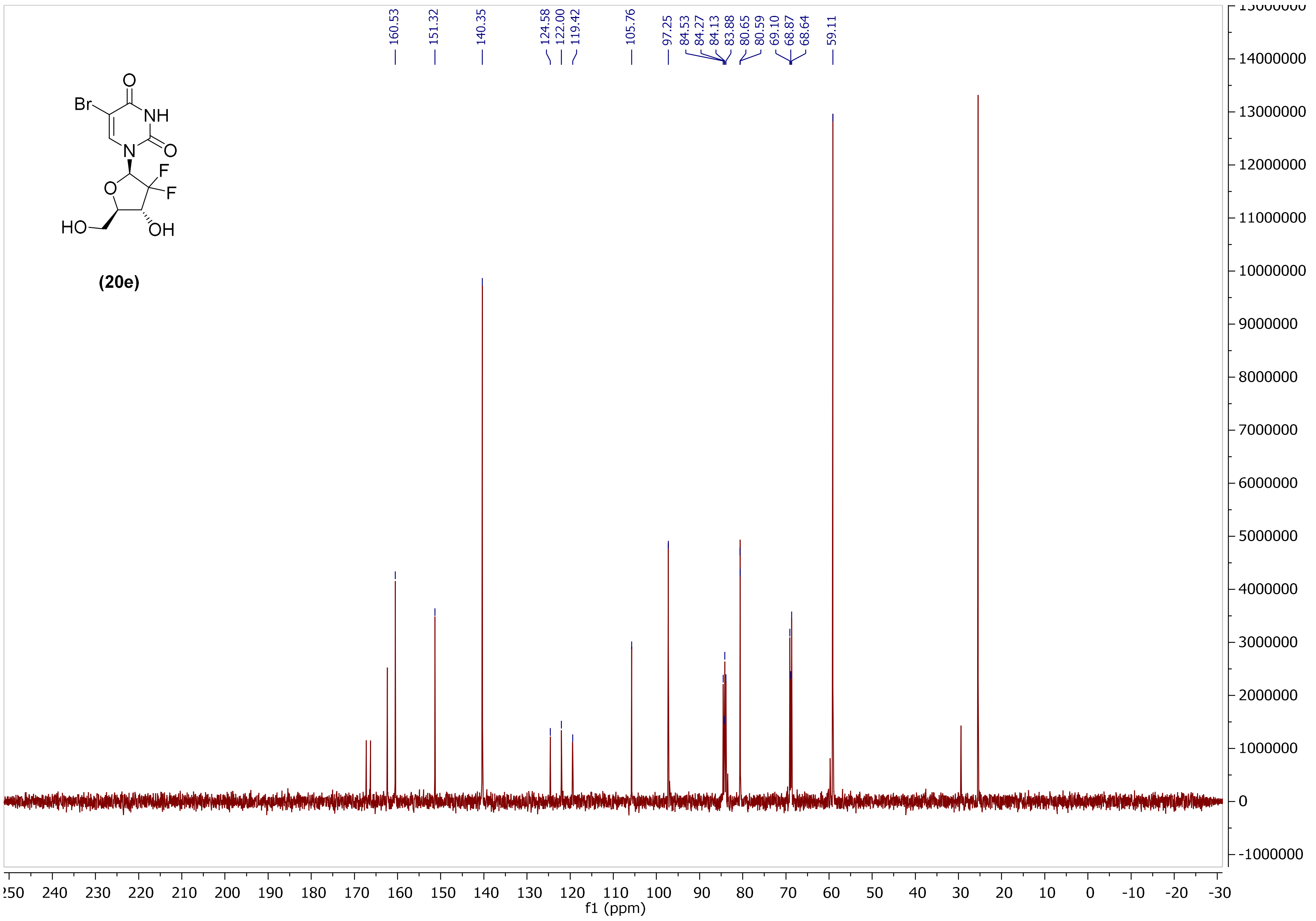

X-Ray data

Crystals suitable for X-ray crystallography were obtained through vapor diffusion of pentane into a dichloromethane or diethyl ether solution of the desired product.

Table S1. Crystal Data, Data Collection and Refinement Parameters for the structures of **14**, **15a**, **15d**, **17a**, **19a**, and **19e**.

data	14	15a	15d
formula	$C_7H_9NO_4$	$C_{18}H_{20}N_2O_4$	$C_{26}H_{23}F_2NO_9$
solvent	—	$0.5(C_4H_8O_2)$	—
formula weight	171.15	372.41	531.45
color, habit	colorless blocky needles	colorless platy needles	colorless platy needles
temperature / K	173	173	173
crystal system	monoclinic	triclinic	monoclinic
space group	$C2/c$ (no. 15)	P–1 (no. 2)	$P2_1$ (no. 4)
a / Å	9.7171(4)	6.0312(5)	5.6021(2)
b / Å	9.5873(3)	11.4502(11)	16.7688(7)
c / Å	16.7838(5)	14.4764(14)	13.3169(6)
α / deg	90	102.501(8)	90
β / deg	90.482(3)	100.716(7)	92.762(4)
γ / deg	90	104.640(8)	90
V / Å^3	1563.53(9)	913.16(15)	1249.54(9)
Z	8	2	2
D_c / g cm^{-3}	1.454	1.354	1.413
radiation used	Cu-Kα	Cu-Kα	Cu-Kα
μ / mm^{-1}	1.036	0.806	1.002
no. of unique reflns			
measured (R_{int})	1567 (0.0369)	3454 (0.0362)	3263 (0.0447)
obs, $\lvert F_o\rvert > 4\sigma(\lvert F_o\rvert)$	1352	2360	2687
completeness (%) [a]	99.9	98.0	98.0
no. of variables	120	254	350
R_1(obs), wR_2(all) [b]	0.0392, 0.1074	0.0418, 0.1126	0.0427, 0.1017

[a] Completeness to 0.84 Å resolution. [b] $R_1 = \Sigma||F_o| - |F_c||/\Sigma|F_o|$; $wR_2 = \{\Sigma[w(F_o^2 - F_c^2)^2] / \Sigma[w(F_o^2)^2]\}^{1/2}$; $w^{-1} = \sigma^2(F_o^2) + (aP)^2 + bP$.

Table S1. …part 2

data	17a	19a	19e
formula	$C_{17}H_{19}BrN_2O_2$	$C_{10}H_{12}N_2O_7$	$C_{10}H_{10}F_2N_2O_7$
solvent	—	—	H_2O
formula weight	363.25	272.22	326.22
color, habit	colorless blocky needles	colorless blocks	colorless plates
temperature / K	173	173	173
crystal system	orthorhombic	monoclinic	orthorhombic
space group	*Pbca* (no. 61)	$P2_1$ (no. 4)	$P2_12_12_1$ (no. 19)
a / Å	13.8765(3)	4.83948(11)	7.5688(2)
b / Å	13.9709(3)	11.6674(3)	9.7017(3)
c / Å	16.3037(4)	9.5352(2)	16.5450(6)
α / deg	90	90	90
β / deg	90	97.873(2)	90
γ / deg	90	90	90
V / Å^3	3160.77(12)	533.32(2)	1214.90(7)
Z	8	2	4
D_c / g cm^{-3}	1.527	1.695	1.783
radiation used	Mo-Kα	Cu-Kα	Cu-Kα
μ / mm^{-1}	2.610	1.268	1.541
no. of unique reflns			
measured (R_{int})	3670 (0.0473)	2007 (0.0202)	2294 (0.0365)
obs, $\|F_o\| > 4\sigma(\|F_o\|)$	2718	1916	1940
completeness (%) [a]	99.9	99.5	99.4
no. of variables	200	189	224
R_1(obs), wR_2(all) [b]	0.0318, 0.0636	0.0269, 0.0717	0.0371, 0.0959

Table S1 provides a summary of the crystallographic data for the structures of **14**, **15a**, **15d**, **17a**, **19a**, and **19e**. Data were collected using Agilent Xcalibur PX Ultra A (**14**, **15a**, **15d**, **19a**, and **19e**) and Xcalibur 3 E (**17a**) diffractometers, and the structures were solved and refined using the OLEX2,[X1] SHELXTL[X2] and SHELX-2013[X3] program systems. The absolute structures of **15d**, **19a** and **19e** could not be determined by use of the Flack parameter [x = –0.1(2),

0.10(12) and –0.10(19) respectively], and so were set by reference to known stereochemistries (at C4 and C5 for **15d**, and at C9 and C10 in both **19a** and **19e**). CCDC 2194811 to 2194816.

X-ray crystallography

The two N8–H hydrogen atoms in the structure of **14** were located from a Δ*F* map and refined freely subject to an N–H distance constraint of 0.90 Å. The O30-based included 1,4-dioxane solvent molecule in the structure of **15a** was found to sit across a center of symmetry, and the unique oxygen atom was found to be disordered over two sites of *ca*. 87 and 13% occupancy. The thermal parameters of the two partial occupancy atoms were restrained to be similar, and the major occupancy atom was refined anisotropically (the minor occupancy atom was refined isotropically). The O21–H hydrogen atom was located from a Δ*F* map and refined freely subject to an O–H distance constraint of 0.90 Å. The N6–H hydrogen atom in the structure of **15d** was located from a Δ*F* map and refined freely subject to an N–H distance constraint of 0.90 Å. The absolute structure of **15d** could not be determined by use of the Flack parameter [*x* = –0.1(2)], and so was set by reference to the known stereochemistries at C4 and C5. The N3–, O13–, O14– and O16–H hydrogen atoms in the structure of **19a** were all located from Δ*F* maps and refined freely subject to X–H distance constraints of 0.90 Å. The absolute structure of **19a** could not be determined by use of the Flack parameter [*x* = 0.10(12)], and so was set by reference to the known stereochemistries at C9 and C10. The N3–, O13–, O14–, O18– and O20–H hydrogen atoms in the structure of **19e** were all located from Δ*F* maps and refined freely subject to X–H distance constraints of 0.90 Å. The absolute structure of **19e** could not be determined by use of the Flack parameter [*x* = –0.10(19)], and so was set by reference to the known stereochemistries at C9 and C10.

References

[X1] O.V. Dolomanov, L.J. Bourhis, R.J. Gildea, J.A.K. Howard, H. Puschmann, *J. Appl. Cryst.*, 2009, **42**, 339-341.

[X2] SHELXTL v5.1, Bruker AXS, Madison, WI, 1998.

[X3] SHELX-2013, G.M. Sheldrick, *Acta Cryst.*, 2015, **C71**, 3-8.

[X4] L. E. da Silva, A. C. Joussef, L. L. Silva, S. Foro, B. Schmidt, *Acta Cryst.*, 2006, E**62**, o3866-o3867

Figures

Figure S. 163 The crystal structure of **14** (50% probability ellipsoids). Identical structure to compound **14** has been reported previously.[X4]

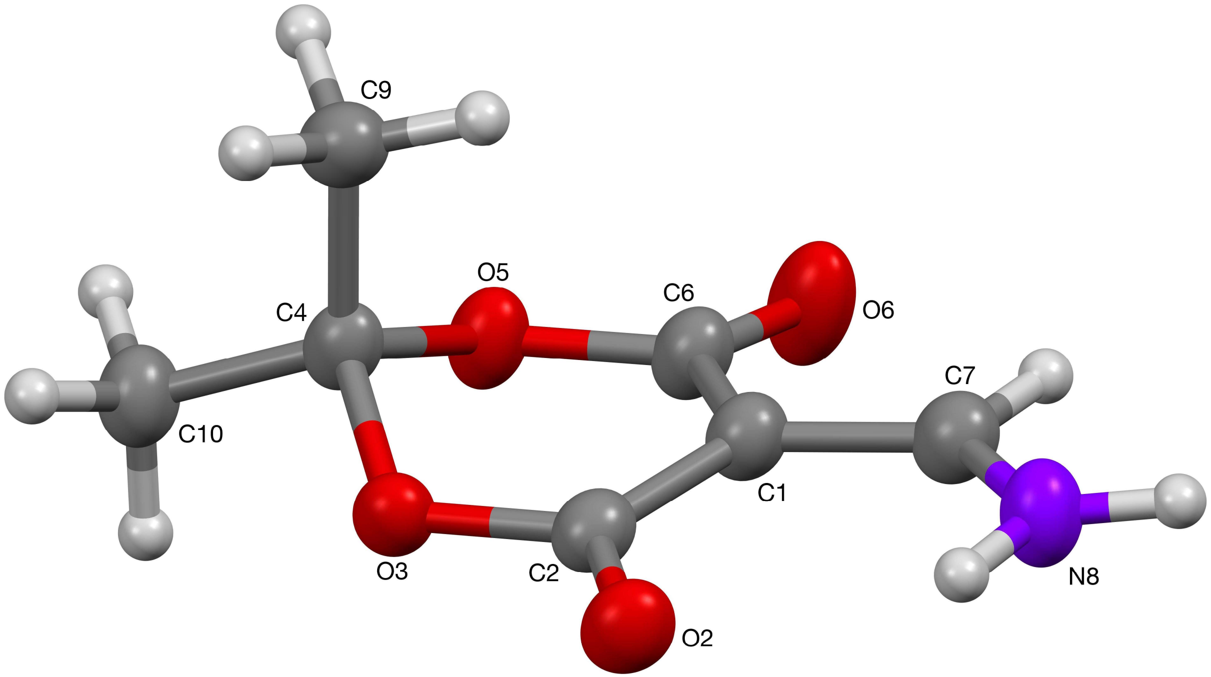

Figure S. 164 The crystal structure of **15a** (50% probability ellipsoids).

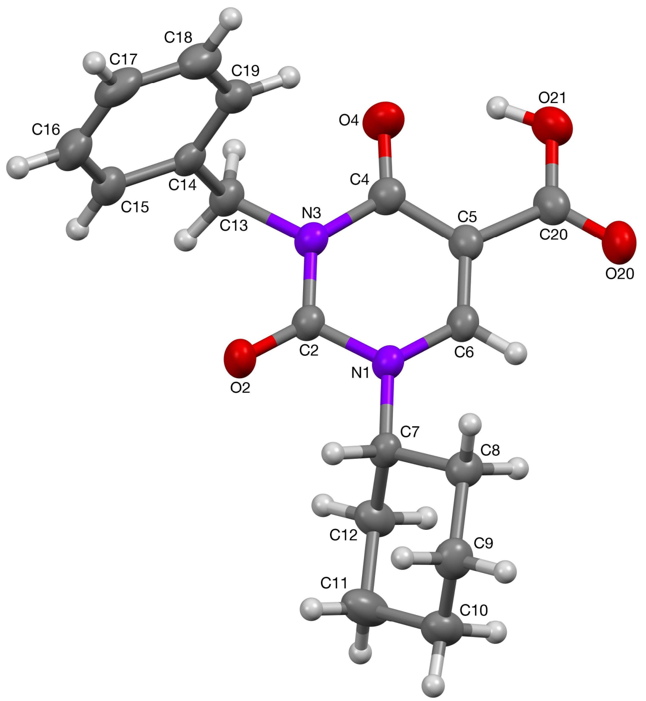

Figure S. 165 The crystal structure of **15d** (50% probability ellipsoids).

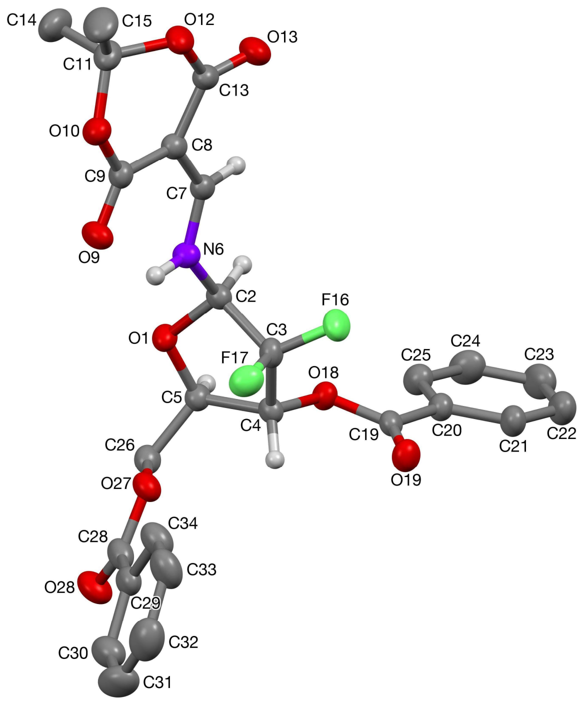

Figure S. 166 The crystal structure of **17a** (50% probability ellipsoids).

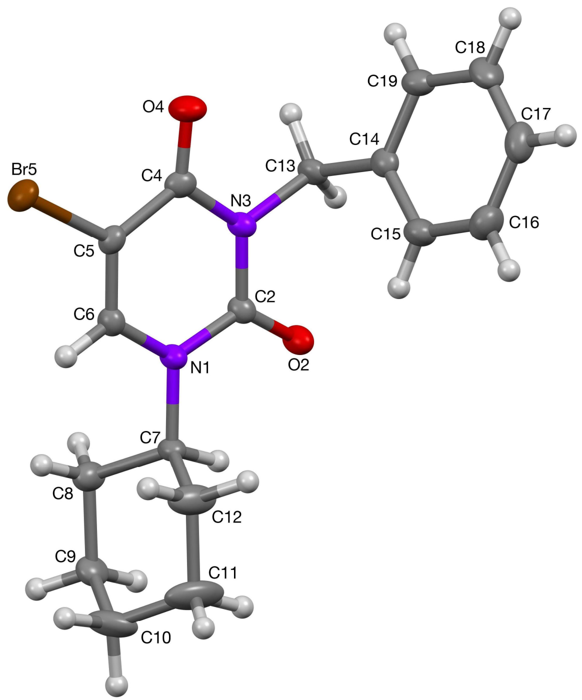

Figure S. 167 The crystal structure of **19a** (50% probability ellipsoids).

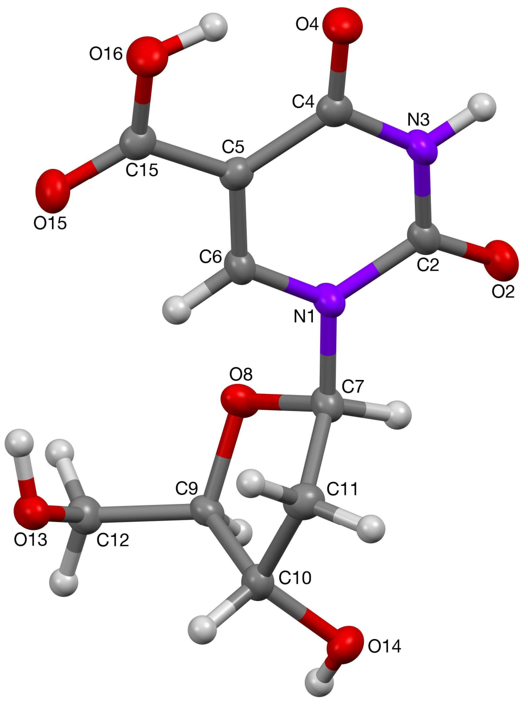

Figure S. 168 The crystal structure of **19e** (50% probability ellipsoids).

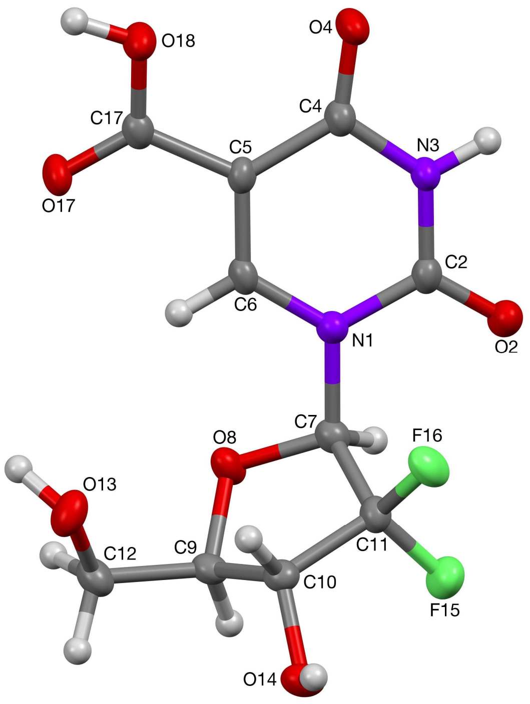

Supplement: Supplementary file 2 — ol2c03152_si_002.pdf [file ol2c03152_si_002.pdf]
